# Structure-based optimization of type III indoleamine 2,3-dioxygenase 1 (IDO1) inhibitors

**DOI:** 10.1080/14756366.2022.2089665

**Published:** 2022-06-27

**Authors:** Ute F. Röhrig, Somi Reddy Majjigapu, Pierre Vogel, Aline Reynaud, Florence Pojer, Nahzli Dilek, Patrick Reichenbach, Kelly Ascenção, Melita Irving, George Coukos, Olivier Michielin, Vincent Zoete

**Affiliations:** a SIB Swiss Institute of Bioinformatics, Molecular Modeling Group, Lausanne, Switzerland; b Laboratory of Glycochemistry and Asymmetric Synthesis, Ecole Polytechnique Fédérale de Lausanne (EPFL), Lausanne, Switzerland; c Protein Production and Structure Core Facility, School of Life Sciences, Ecole Polytechnique Fédérale de Lausanne (EPFL), Lausanne, Switzerland; d Department of Oncology UNIL-CHUV, Ludwig Lausanne Branch, Epalinges, Switzerland; e Department of Oncology, University Hospital of Lausanne (CHUV), Ludwig Cancer Research-Lausanne Branch, Lausanne, CH-1011, Switzerland

**Keywords:** Cancer immunotherapy, structure-based drug design, tryptophan metabolism, X-ray crystallography

## Abstract

The haem enzyme indoleamine 2,3-dioxygenase 1 (IDO1) catalyses the rate-limiting step in the kynurenine pathway of tryptophan metabolism and plays an essential role in immunity, neuronal function, and ageing. Expression of IDO1 in cancer cells results in the suppression of an immune response, and therefore IDO1 inhibitors have been developed for use in anti-cancer immunotherapy. Here, we report an extension of our previously described highly efficient haem-binding 1,2,3-triazole and 1,2,4-triazole inhibitor series, the best compound having both enzymatic and cellular IC_50_ values of 34 nM. We provide enzymatic inhibition data for almost 100 new compounds and X-ray diffraction data for one compound in complex with IDO1. Structural and computational studies explain the dramatic drop in activity upon extension to pocket B, which has been observed in diverse haem-binding inhibitor scaffolds. Our data provides important insights for future IDO1 inhibitor design.

## Introduction

**Figure 1.**
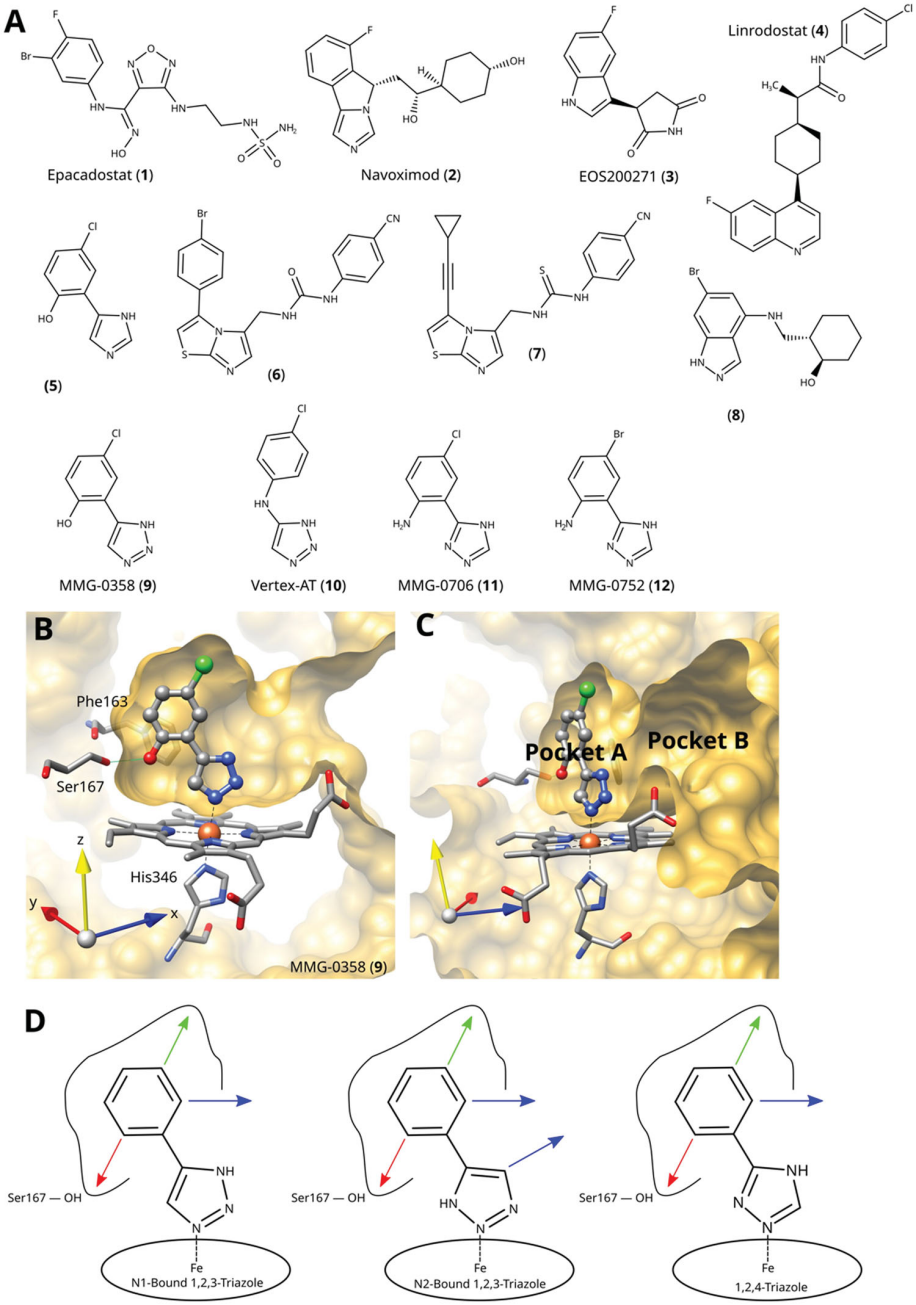
(A) Examples of clinical and other potent IDO1 inhibitors. (B) and (C) Rotated views of the binding pockets A and B in IDO1-active site (PDB ID 6r63^22^; ligand MMG-0358). (D) Main lead optimisation strategies pursued in this work. The red arrow denotes a preferentially hydrogen-bonding substituent, the green arrow a hydrophobic substituent, and the blue arrows potential access points to pocket B. As demonstrated before, the acidic hydrogens on the triazole rings are crucial for activity and therefore cannot provide access to pocket B.

Immuno oncology provides powerful therapies against cancer in the form of immune checkpoint inhibitors, adoptive cell therapies, monoclonal antibodies, oncolytic viruses, cancer vaccines, and other immuno modulators. However, low response rates due to tumoral immune suppression and resistance remain an unsolved issue^1^. L-Trp catabolism along the kynurenine pathway is an important mechanism employed by cancer cells to escape a potentially effective immune response^2^^,^^3^. The rate-limiting step in this pathway is catalysed by indoleamine 2,3-dioxygenase 1 (IDO1) and by tryptophan 2,3-dioxygenase (TDO), with the IDO1 paralogue IDO2 also potentially playing a role^4^^,^^5^ Preclinical data suggests that a combination of IDO1 inhibitors with other anticancer agents results in effective anti-tumor immunity^6–8^. However, in a phase 3 clinical trial of the IDO1 inhibitor epacadostat in combination with pembrolizumab in melanoma patients, the combination failed to increase the overall and progression-free survival when compared to pembrozilumab alone^9^. This failure highlighted the need for a better understanding of the role of the kynurenine pathway, for the development of better IDO1 inhibitors, and for improved trial design^10^^,^^11^. Additional aspects of IDO1 biology are constantly discovered and may influence its role in cancer, such as its signalling activity^12–14^, regulation by haem availability^15^, nitrite reductase activity in hypoxic tissues^16^, involvement in the redox signalling pathways of hydrogen peroxide and singlet oxygen^17^, and activation by polysulphides^18^. Based on the ongoing interest for IDO1 inhibitors capable of modulating these different pathways and processes selectively, a multitude of small-molecule IDO1 inhibitors have been described^19^^,^^20^, and more than 60 crystal structures of IDO1 have been deposited in the protein data bank (PDB)^21^. These structures with a large diversity of bound cofactors and ligands, including the clinical-stage IDO1 inhibitors epacadostat (**1**, INCB024360, Figure 1A)^23^, navoximod (**2**, NLG-919/GDC919)^24^, EOS200271 (**3**)^25^, and linrodostat (**4**, BMS-986205)^26^, yield a wealth of information for inhibitor design^27^.

We have previously classified IDO1 inhibitors into four types according to their preferential binding and inhibition mechanism^22^. Type i inhibitors (e.g. 1-methyl-L-tryptophan) preferentially bind to oxygen-bound holoIDO1, type ii inhibitors (e.g. epacadostat) to free ferrous holoIDO1, type iii inhibitors (e.g. navoximod) to free ferric holoIDO1, and type iv inhibitors (e.g. linrodostat) to apoIDO1. Lead optimisation of haem-iron binding type ii and type iii inhibitors has proven difficult due to the selectivity and sensitivity of the haem–ligand interactions to changes in the electronic structure of the ligand^28^ and due to the small size of the distal haem pocket (pocket A)^29^, crucial for inhibitor activity. The IDO1 active site further comprises Pocket B, which extends from pocket A towards the entrance of the active site (Figure 1B,C), and whose size and shape are determined by the conformation of the flexible JK-loop^27^. Although its influence on inhibitor affinity is less pronounced than pocket A, it is of interest for modulation of other compound properties such as specificity, absorption, distribution, metabolism and excretion (ADME), and pharmacokinetics/pharmacodynamics (PK/PD).

Despite the large number of published IDO1 inhibitors, there are only a very limited number of sub-micromolar type ii and type iii inhibitor scaffolds known. Epacadostat (**1**) binds to the haem iron through an unusual high-affinity hydroxyamidine scaffold, which was discovered by Incyte^23^^,^^30^ and has later been exploited by other groups^31–38^. Due to its unique tilted iron-binding conformation, it provides a straightforward access to pocket B and allowed the development of numerous nanomolar IDO1 inhibitors with a high selectivity over TDO^27^. Except for the hydroxyamidines, almost all nanomolar haem-binding IDO1 inhibitors are based on a haem-binding free or fused azole scaffold.

Imidazoles are classical haem binders, as present in the haem-binding histidine side chain and in many antifungal drugs, and 4-phenyl-imidazole^39^ was the first cocrystallized IDO1 inhibitor^40^. Early structure-based optimisation of this chemotype yielded the 2-hydroxy-substituted phenyl derivative as the most active compound^41^. Combination of the 2-hydroxy substitution with a 5-chloro substitution led to the most efficient imidazole compound (**5**) with a ligand efficiency (LE) of 0.68 kcal/mol/heavy atom (HA)^24^^,^^28^. In subsequent developments, extending the scaffold to pocket B, a drop in LE was always encountered^42^, but nanomolar activities could be obtained in some 1,5-disubstituted imidazoles^43^^,^^44^. Rigidification of the scaffold to imidazo[5,1-a]isoindole also allowed to retain nanomolar activities^45–49^ and led to the development of navoximod (**2**, Figure 1)^24^. Independently from the work on imidazo[5,1-a]isoindoles, structural and functional data of imidazo[2,1-b][1,3]thiazole based IDO1 inhibitors was disclosed in 2014 (**6**)^50^. Subsequent work on this fused imidazole scaffold led for example to compound **7** with a good enzymatic activity and a LE of 0.41 kcal/mol/HA^51^. However, compounds with this scaffold lack cellular activity^51–53^.

Indazoles are also known haem binders, and the indazol-4-amine scaffold developed by IOmet Pharma^54^ yielded selective nanomolar TDO inhibitors and dual IDO1/TDO inhibitors^55–57^. X-ray structures of the complexes between IDO1 and some indazol-4-amines such as compound **8** have recently been resolved^57^. These compounds provide access to pocket B while preserving a LE of up to 0.47 kcal/mol/HA.

We have previously discovered 1,2,3-triazoles as highly efficient IDO1 inhibitors and resolved the X-ray structure of MMG-0358 (**9**) in complex with IDO1 (Figure 1)^22^^,^^28^^,^^29^^,^^58^. Compound **9** forms a direct bond to the haem iron, a hydrogen bond with Ser167 through its hydroxy function, and hydrophobic interactions through its chloro substituent, leading to a LE of 0.76 kcal/mol/HA. A few 4,5-disubstituted 1,2,3-triazoles with nanomolar activities have been reported^59^, but as we detail below, we failed to reproduce these results. Interestingly, the N-phenyl-1,2,3-triazol-4-amine (**10**) developed by Vertex^60^ demonstrates an IDO1 inhibition mechanism distinct from the 4-phenyl1,2,3-triazoles despite a similar binding mode^22^.

1,2,4-Triazole is a common haem-binding scaffold present in many antifungal drugs such as fluconazole, and its direct iron binding in sterol 14α-demethylase (CYP51) is well documented by structural data. However, these 1-substituted 1,2,4-triazoles have been found to be inactive on IDO1, at variance with imidazole antifungals such as miconazole, which showed some activity^61^. However, we recently demonstrated that the 3-substituted 1,2,4-triazole scaffold can provide highly efficient IDO1 inhibitors. Two simple substitutions on the 3-phenyl-1,2,4-triazole scaffold improved its inhibitory activity by more than four orders of magnitude from the millimolar to the low nanomolar range, and we provided structural data for IDO1 binding of MMG-0706 (**11**) and MMG-0752 (**12**, Figure 1)^28^. These compounds feature a 2-amino, 5-halogen di-substituted phenyl ring and display a LE of 0.80 kcal/mol/HA for the bromo compound, to our knowledge the highest LE reported for IDO1 inhibitors to date. Based on molecular modelling and quantum chemical calculations, we were able to explain the improved activity of the 2-amino substituent versus the 2-hydroxy substituent in this scaffold, due to simultaneous intramolecular and intermolecular interactions^28^.

Here, we describe our efforts to improve and extend compounds comprising the 1,2,3-triazole and 1,2,4-triazole haem-binding scaffolds, the best new compound (**144**) demonstrating both enzymatic and cellular IC_50_ values of 34 nM. Although this compound and our previously described compounds such as MMG-0358 (**9**), MMG-0706 (**11**), or MMG-0752 (**12**)^28^^,^^58^ are highly efficient both in an enzymatic and in a cellular environment, they are very sensitive to even the smallest changes in their chemical structure, and therefore lack available sites to modulate their ADME and PK/PD properties. Analysing the active site structure of IDO1 and the chemical structures of other known IDO1 inhibitors, it would be natural to extend the compounds from pocket A in the haem distal site to pocket B towards the entrance of the active site (Figure 1D). However, azole ligands are not optimally suited to be extended to pocket B due to their preferred orientation, and this extension often leads to a dramatic decrease in activity for many compounds^27^. Here, we resolve one new X-ray structure of a previously reported triazole extending to pocket B (**13**, MMG-0472), which validates our docking predictions. Based on this new structural data and the measured activities of almost 100 new compounds, we give recommendations for the development of future IDO1 inhibitors.

### Chemistry

**Scheme 1.**
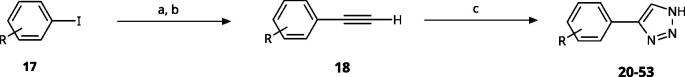
General synthesis of 4-aryl-1,2,3-triazoles. Reagents and conditions: (a) TMSA, PdCl_2_(PPh_3_)_2_, Et_3_N, CuI, dioxane, 45 °C, 5 h. (b) KF, MeOH, rt, 3 h, overall yield 48-85% for 2 steps. (c) TMSN_3_, CuI, DMF:MeOH (9:1), 100 °C, 10–12 h, yield 44–85%.

**Scheme 2.**
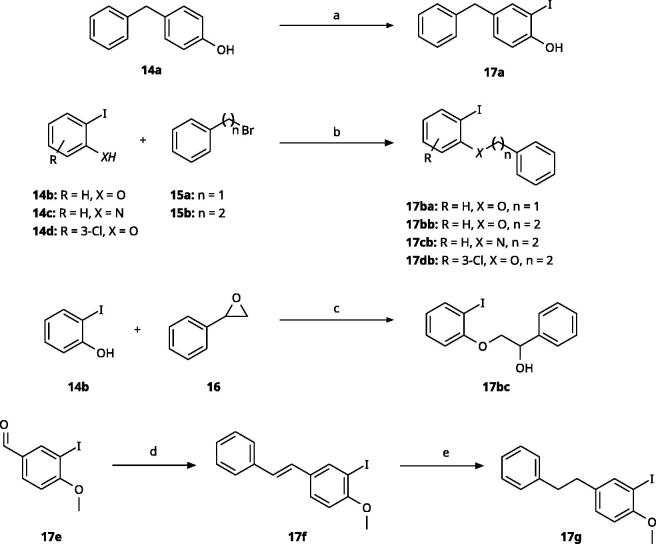
Synthesis of aryl iodides. Reagents and conditions: (a) aq. NH_3_, KI, I_2_, DMF, rt, 1 h, yield 50%. (b) K_2_CO_3_, DMF, 80 °C, overnight, yield 50–95%. (c) Cs_2_CO_3_, DMF, 100 °C, 24 h, yield 60%. (d) PhCH_2_P(Br)(Ph)_3_, [(CH_3_)_3_Si]_2_NNa, THF, rt. (e) N_2_H_4_·H_2_O, FeCl_3_·6H_2_O, EtOH, 100 °C, 24 h, overall yield 90% for 2 steps.

All compounds were synthesised using existing protocols or procedures adapted from the literature. 4-Aryl-1,2,3-triazoles (**20**–**53**, Table 1) were synthesised according to the method developed by Yamamoto and co-workers^63^, which involves reaction of ethynyl derivatives (**18**) with trimethylsilyl azide (TMSN_3_, Scheme 1). The ethynyl derivatives were synthesised from iodo substrates (**17**, Scheme 2) by the Sonogashira coupling reaction^64^. Phenols **20**, **39**, and **44** were obtained by demethylation of the corresponding methyl ethers with 48% HBr in water at 100 °C.

**Table 1.** 4-Aryl-1,2,3-triazoles with substituted phenyl groups. 
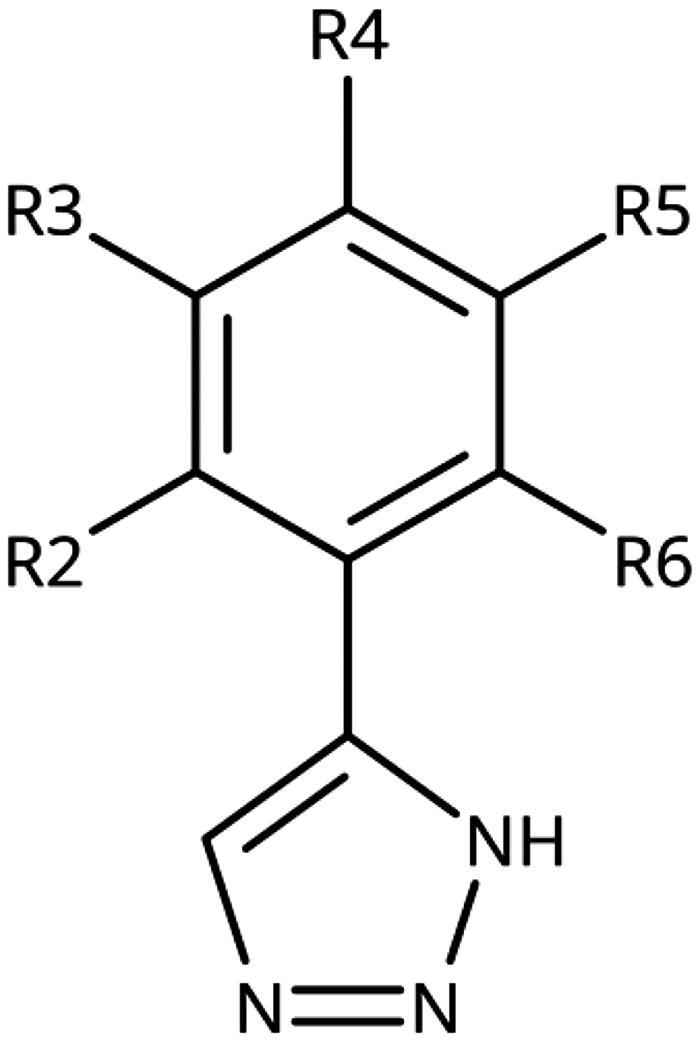

Compound	R2	R3	R4	R5	R6	IC_50_ [μM] (SD^a^)	LE^b^ [kcal/mol/HA]
**19**						10 (1)^28^	0.62
**20**			–OH			>1000	
**21**				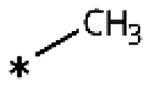		22^58^	0.53
**22**				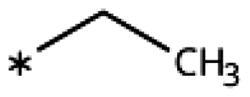		13^58^	0.51
**23**				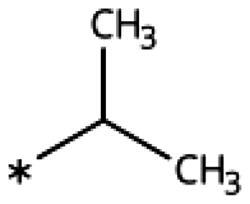		280 (20)	0.35
**24**				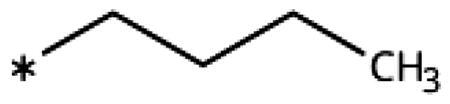		34 (–)	0.41
**25**					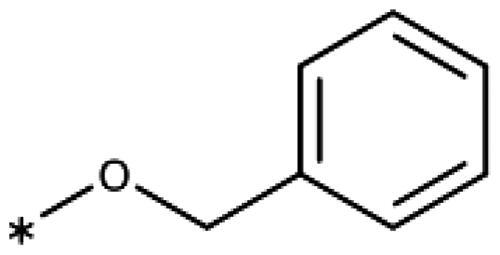	>500	
**26**					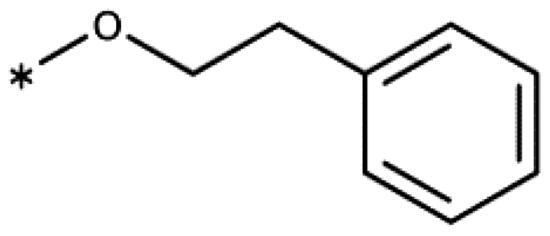	>100	
**27**					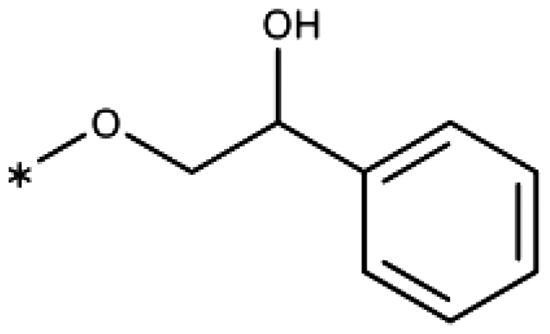	>500	
**28**					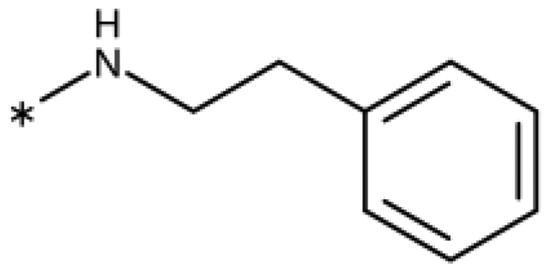	>250	
**29**	–OH					2.3 (0.8)^28^	0.64
**30**				–Cl		0.35 (0.04)^28^	0.73
**9**	–OH			–Cl		0.059 (0.003)^28^	0.76
**31**	–			–Cl		1.5 (0.2)^28^	0.61
**32**	–			–Cl		4.3^28^	0.56
**33**	–			–		>1000^28^	
**34**	–OH			–F		0.14 (0.01)	0.72
**35**	–OH			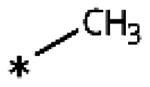		0.55 (0.05)	0.66
**36**	–OH			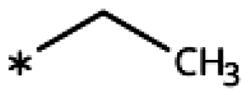		0.25 (0.02)	0.64
**37**	–OH			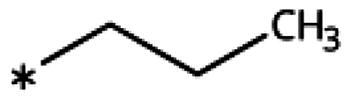		0.75 (0.03)	0.56
**38**	–OH			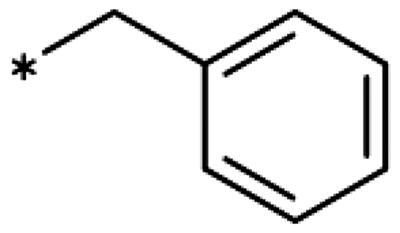		88 (–)	0.29
**39**	–OH			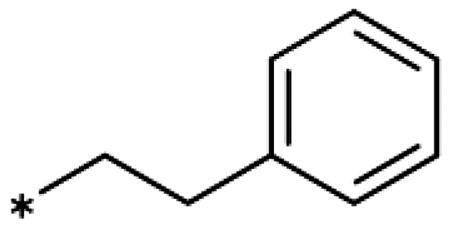		160 (–)	0.26
**40** ^62^				–Cl	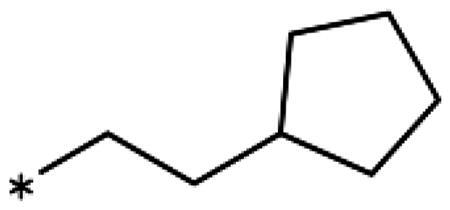	9.5 (0.8)	0.36
**13** ^62^				–Cl	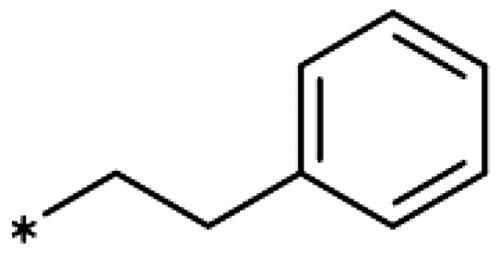	15 (1)	0.33
**41** ^62^				–Cl	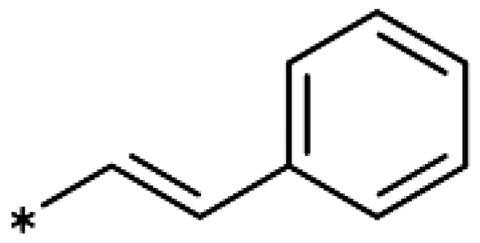	49 (9)	0.29
**42** ^62^				–Cl	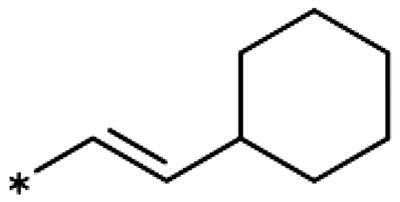	260 (50)	0.24
**43**				–Cl	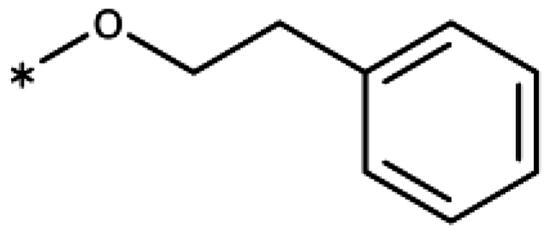	31 (0.4)	0.29
**44**	–OH				–OH	13 (3)	0.51
**45**	–				–OCH_3_	>1000	
**46**	–Cl			–Cl		6.4^58^	0.54
**47**	–Cl	–Cl				23^58^	0.49
**48**	–Cl		–Cl			31 (0.09)	0.47
**49**		–Cl	–Cl			51 (4)	0.45
**50**		–Cl		–Cl		58 (7)	0.44
**51**	–Cl				–Cl	280 (20)	0.37
**52**	–Cl		–Cl	–Cl		33 (0.5)	0.44
**53**	–Cl		–Cl		–Cl	>500	

^a^ Standard Deviation (SD). ^b^Ligand Efficiency (LE).

**Scheme 3.**
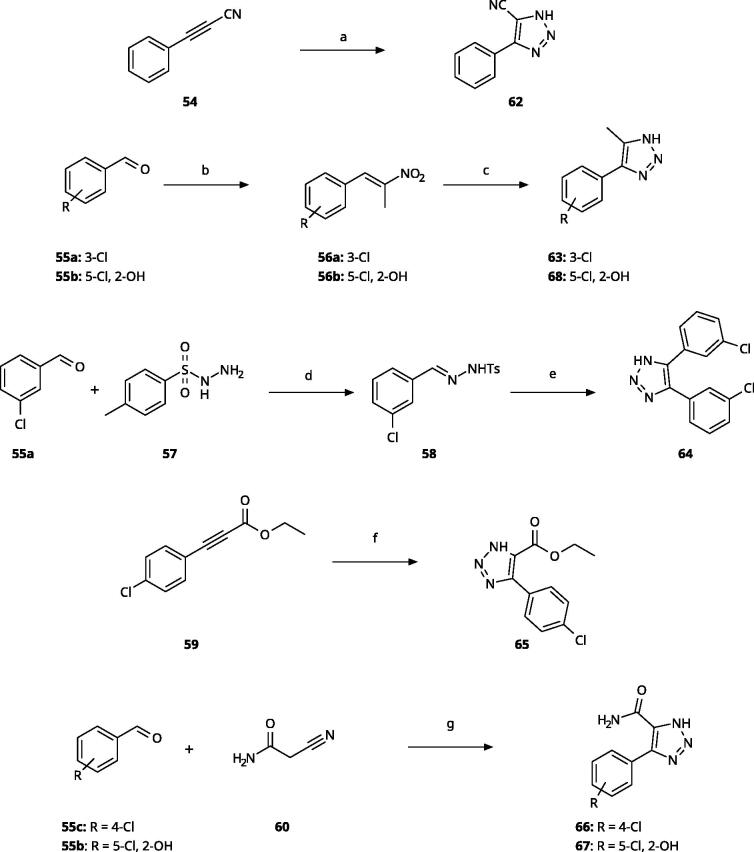
Synthesis of 5-substituted 4-aryl-(1*H*)-1,2,3-triazoles. Reagents and conditions: (a) NaN_3_, DMF, 90 °C, 3 h, yield 75%. (b) Nitroethane, NH_4_OAc, AcOH, reflux, 2 h. (c) NaN_3_, *p*TsOH, DMF, 60 °C, 14 h, overall yield 65–70% for 2 steps. (d) EtOH, rt, 2 h (e) Cs_2_CO_3_, DMF, 100 °C, 4 h, overall yield 70% for 2 steps. (f) NaN_3_, DMSO, 60 °C, 6 h, yield 60%. (g) NaN_3_, Et_3_N·HCl, DMF, 70 °C, 10 h, yield 55–65%.

5-Substituted 4-aryl-1,2,3-triazoles (Table 2) were obtained according to Scheme 3. Phenyl propynenitrile (**54**) reacted with sodium azide in DMF at 90 °C to produce **62**^65^. Compounds **63** and **68** were obtained from the corresponding aryl benzaldehyde (**55a**, **55b**), nitroethane and ammonium acetate through condensation in presence of acetic acid, followed by dipolar cycloaddition with HN_3_ engendered with sodium azide and *p*-toluenesulfonic acid in DMF at 60 °C^66^. The 4,5-diaryl derivative **64** was obtained from 3-chlorobenzaldehyde (**55a**) by reaction with tosylhydrazine (**57**) in ethanol followed by cyclisation with Cs_2_CO_3_ at 100 °C in DMF^67^. The reaction of ethyl 3–(4-chlorophenyl)propiolate (**59**) with sodium azide in DMSO at 60 °C yielded **65**^68^. The reaction of 2-cyanoacetamide (**60**) with aryl benzaldehydes (**55b**, **55c**) in the presence of sodium azide and Et_3_N·HCl allowed the formation of triazoles **66** and **67**^69^.

**Table 2.** 5-Substituted 4-Aryl-1,2,3-Triazoles. 
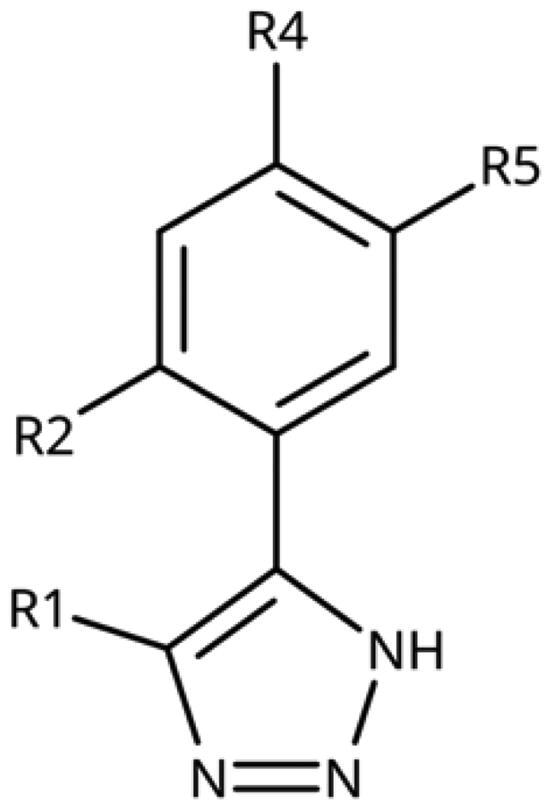

Compound	R1	R2	R4	R5	IC_50_ [μM] (SD^a^)	LE^b^ [kcal/mol/HA]
**61**	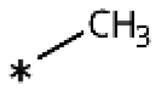				>1000^58^	
**62**	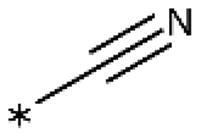				630 (70)	0.34
**63**	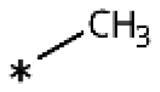			–Cl	>1000	
**64** ^59^	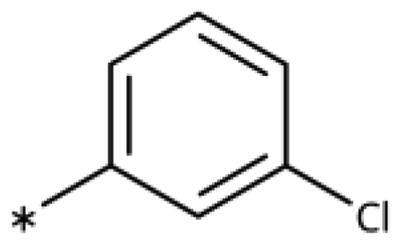			–Cl	>50	
**65** ^59^	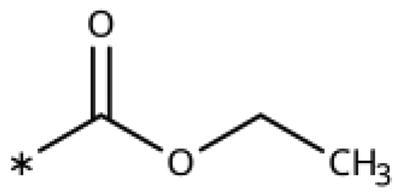		–Cl		>1000	
**66** ^59^	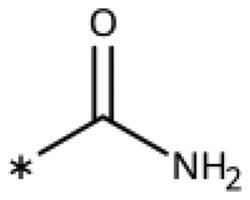		–Cl		800	0.28
**67**	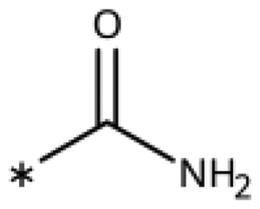	–OH		–Cl	>1000	
**68**	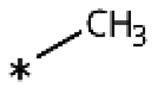	–OH		–Cl	220 (7)	0.36

^a^ Standard Deviation (SD). ^b^Ligand Efficiency (LE).

**Scheme 4.**
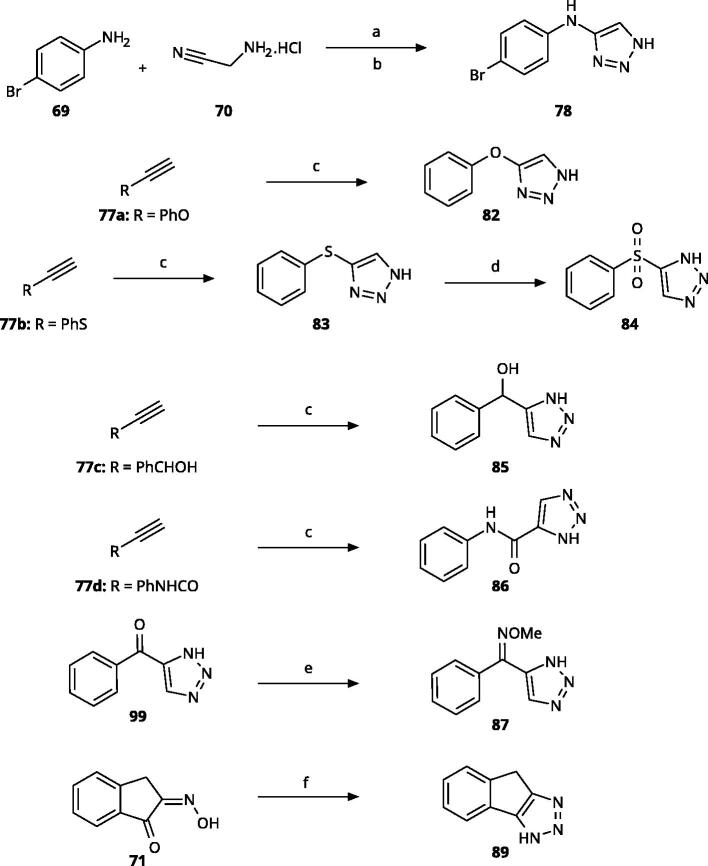
Aryl-tether-1,2,3-triazoles and annulated derivatives. Reagents and conditions: (a) NaNO_2_, 2M HCl, CH_3_COONa, 0 °C-rt, 1 h. (b) EtOH, reflux, overnight, overall yield 70% for 2 steps. (c) TMSN_3_, CuI, DMF:MeOH (9:1), 100 °C, 10–12 h, yield 50–70%. (d) H_2_O_2_, (NH_4_)_2_MoO_4_, MeOH, 0 °C- rt, 14 h, yield 80%. (e) CH_3_ONH_2_·HCl, NaOAc, H_2_O:EtOH, 70 °C, rt, 15 h, yield 55%. (f) N_2_H_4_·H_2_O, KOH, diethylene glycol, 170–190 °C, 6 h, yield 40%.

**Scheme 5.**
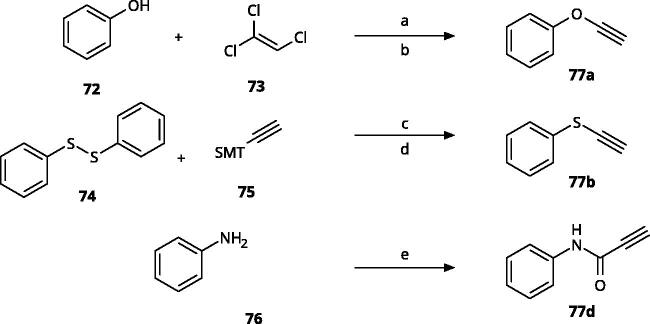
Synthesis of ethynes. Reagents and conditions: (a) NaOH, DMSO, rt, 4 h. (b) *n*-BuLi, Et_2_O, -78 °C-rt, 2h, overall yield 40% for 2 steps. (c) *n*-BuLi, THF, -78 °C-rt, overnight. (d) KF, MeOH, rt, 4 h, overall yield 85% for 2 steps. (e) propiolic acid, DCC, DMAP, Et_2_O, rt, 18 h, yield 90%.

4-Aryl-tether-1,2,3-triazoles (Table 3) were synthesised according to Scheme 4. 4-Arylamino-1,2,3-triazole **78** resulted from treatment of 4-bromoaniline (**69**) with 2-aminoacetonitrile (**70**) and sodium nitrite in 2 M HCl at rt first, then by boiling in ethanol^71^. Analogues **82**, **83**, **85**, and **86** were derived from the corresponding ethynyl derivative **77** (Scheme 5) and TMSN_3_^63^. Reaction of sulphide **83** with H_2_O_2_ in the presence of ammonium molybdate in methanol at rt produced sulphone **84**^72^. *O*-methyl oxime **87** was obtained from the corresponding ketone **99** and *O*-methylhydroxylamine hydrochloride in H_2_O:EtOH at 70 °C^73^. The annulated derivative **89** was obtained by treatment of 1,2-indandione-2-oxime (**71**) with hydrazine hydrate and potassium hydroxide at 170–190 °C^74^.

**Table 3.** 4-Aryl-tether-1,2,3-triazoles and annulated derivatives. 
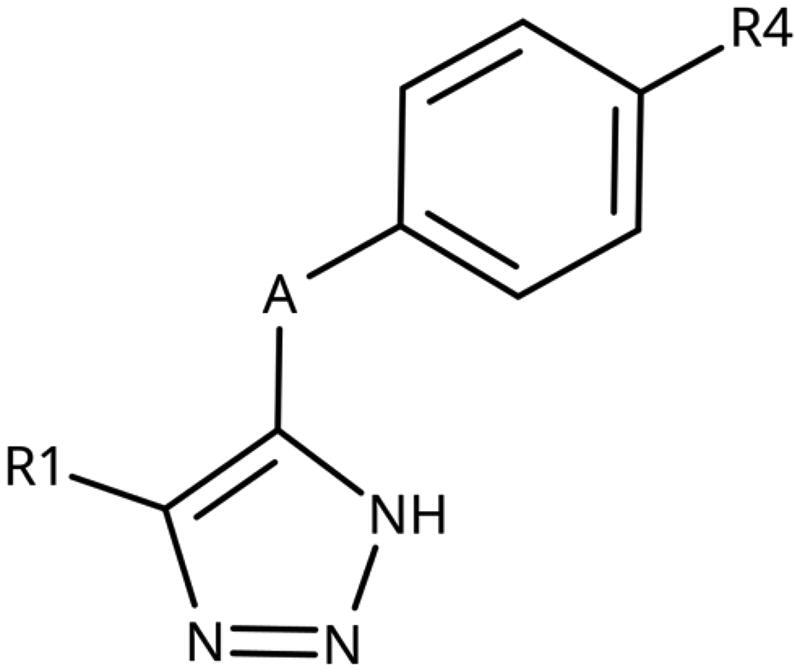

Compound	A	R1	R4	IC_50_ [μM] (SD^a^)	LE^b^ [kcal/mol/HA]
**10** ^60^	–NH–		–Cl	57 (20)	0.44
**78** ^70^	–NH–		–Br	52 (8)	0.45
**79**	–NH–			>100	
**80**	–NH–	–phenyl		>250	
**81**	–CH_2_–			>1000^58^	
**82**	–O–			>1000	
**83**	–S–			590 (20)	0.37
**84**	–SO_2_–			>1000	
**85**	–CHOH–			>1000	
**86**	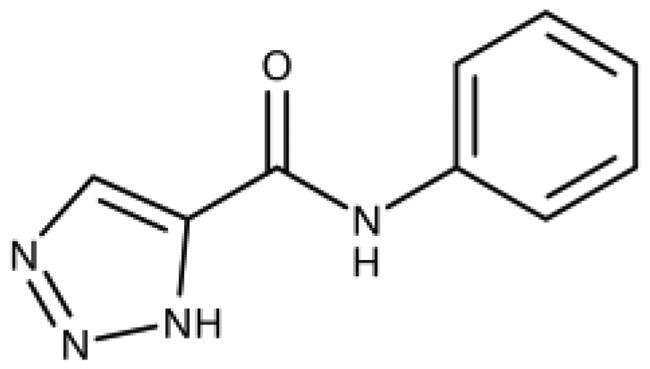			>1000	
**87**	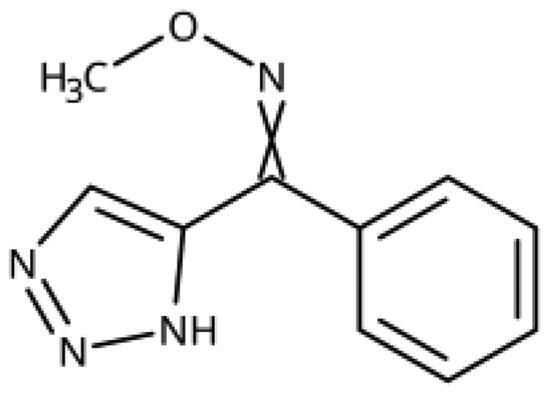			>1000	
**88**	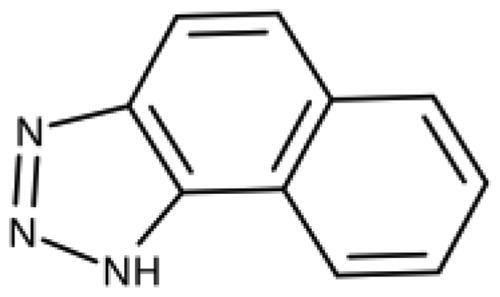			>100	
**89**	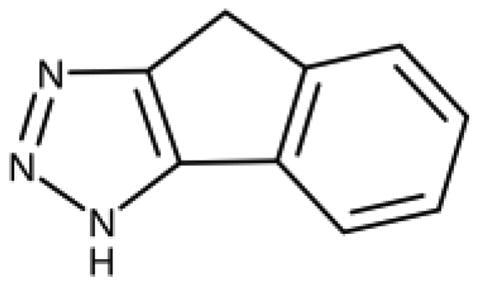			>1000	

^a^ Standard Deviation (SD). ^b^Ligand Efficiency (LE).

**Scheme 6.**
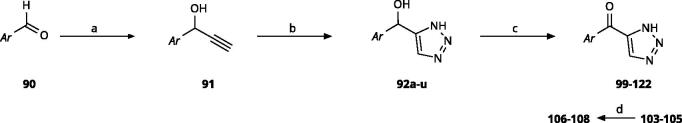
Synthesis of 5-aroyl and 5-hetaroyl-1,2,3-triazoles. Reagents and conditions: (a) ethynylmagnesium bromide, THF, 3 °C-rt, 1 h. (b) TMSN_3_, CuI, DMF:MeOH (9:1), 100 °C, 10–12 h, overall yield 55–85% for 2 steps. (c) PCC, DCM, rt, 2 h, yield 35–73%. (d) 48% HBr in H_2_O, 100 °C, 14 h, yield 45–50%.

**Scheme 7.**
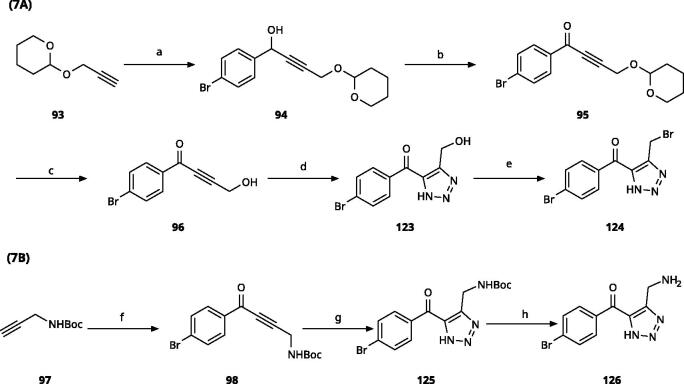
Synthesis of 4-substituted-5-aroyl-(1H)-1,2,3-triazoles. Reagents and conditions: (a) 4-Bromobenzaldehyde, *n*-BuLi, -78 °C to -38 °C, THF, 3 h, yield 88%. (b) MnO_2_, DCM, rt, 5 h, yield 97%. (c) PPTSA, EtOH, 50 °C, 4 h, yield 75%. (d) TMSN_3_, CuI, DMF/MeOH (9:1), 100 °C, 10–12 h, yield 50%. (e) CBr_4_, PPh_3_, DMF, rt, 4 h, yield 85%. (f) 4-Bromobenzoyl chloride, PdCl_2_(PPh_3_)_2_, CuI, Et_3_N,THF, rt,1 h, yield 90%. (g) TMSN_3_, CuI, DMF/MeOH (9:1), 10 °C, 10-12 h, yield 45%. (h) TFA, DCM, rt, overnight, yield 75%.

5-Aroyl-1*H*-1,2,3-triazoles (Table 4) were obtained according to Scheme 6. Alkynylation of arenecarbaldehyde **90** with ethynylmagnesiumbromide in THF produced the corresponding arylpropargyl alcohol (**91**). The latter underwent dipolar cycloaddition with TMSN_3_ in presence of CuI as catalyst (**92a**–**92u**). Oxidation with pyridinium chlorochromate (PCC) in dichloromethane gave **99**–**122**^63^^,^^75^^,^^76^. Compounds **103**–**105** were demethylated with 48% HBr in H_2_O at 100 °C to produce the corresponding phenols **106**–**108**. The alkynylation of 4-bromobenzaldehyde with 2-(prop-2-ynyloxy)tetrahydro-2*H*-pyran (**93**) in presence of *n*-BuLi in THF at −78 °C yielded the propargyl alcohol **94** after aqueous work-up. **94** was oxidised with MnO_2_, followed by hydrolysation to **96** in the presence of pyridinium tosylate (PPTSA). Dipolar cycloaddition of **96** with TMSN_3_ (CuI catalyst) in DMF yielded triazole **123**, which was brominated into **124** by reaction with triphenylphosphine and CBr_4_ in DMF at rt (Scheme 7A)^63^^,^^77^^,^^78^. Triazolomethanamine **126** was obtained from 4-bromobenzoyl chloride and *tert*-butyl prop-2-yn-1-ylcarbamate (**97**), producing ynone **98** in the presence of PdCl_2_(PPh_3_)_2_ as catalyst. **98** reacted with TMSN_3_ (CuI catalyst), yielding carbamate **125**. In the presence of trifluoroacetic acid in dichloromethane at rt, carbamate **125** provided the primary amine **126** (Scheme 7B)^63^^,^^79^.

**Table 4.** 5-Aroyl and 5-Hetaroyl-1,2,3-Triazoles. 
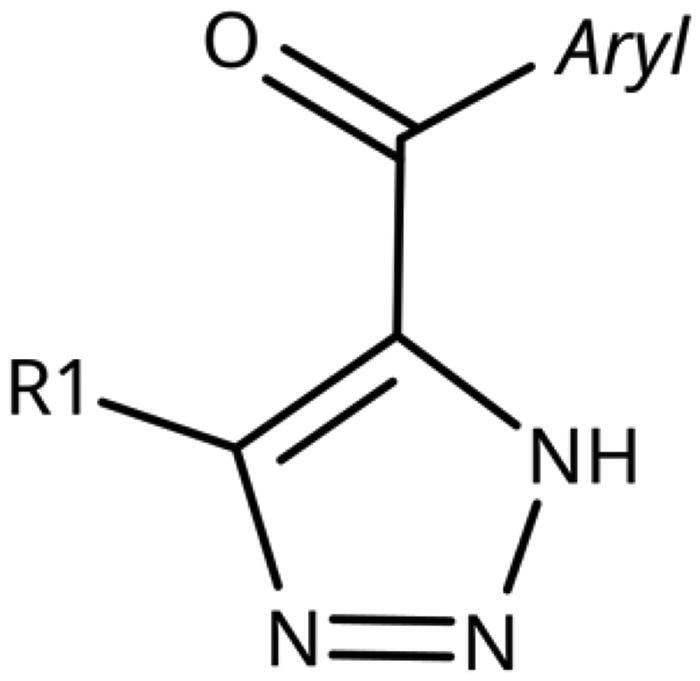

Compound	Aryl	R1	IC_50_ [μM] (SD^a^)	LE^b^ [kcal/mol/HA]
**99**	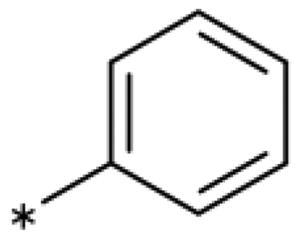		430 (60)	0.35
**100**	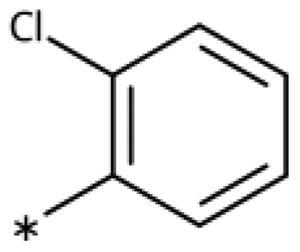		>1000	0.20
**101**	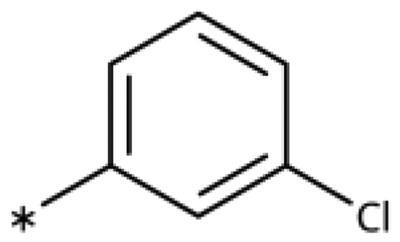		450 (40)	0.33
**102**	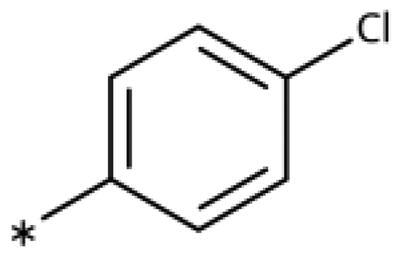		31 (6)	0.44
**103**	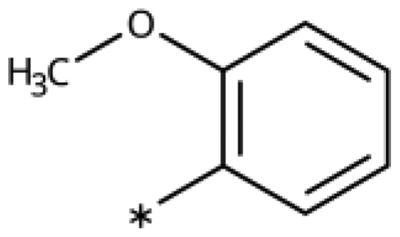		>1000	
**104**	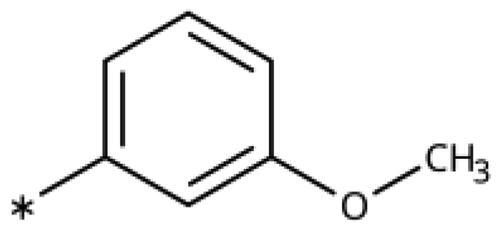		920 (200)	0.28
**105**	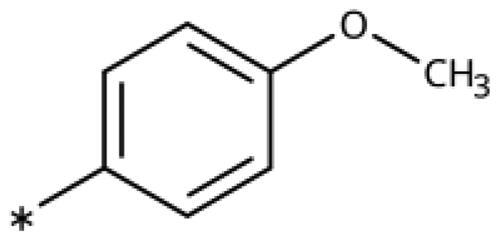		200 (20)	0.34
**106**	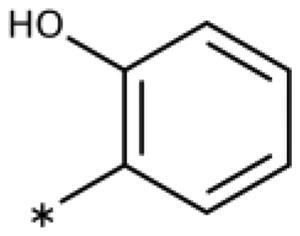		130 (30)	0.38
**107**	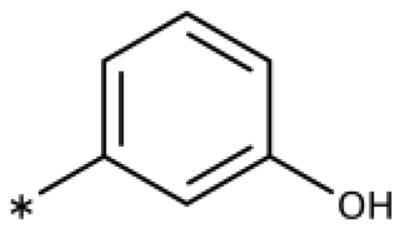		>1000	
**108**	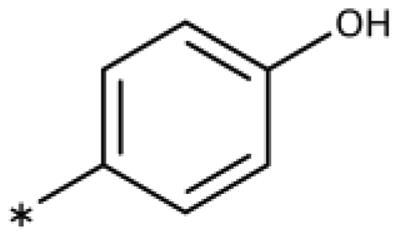		>1000	
**109**	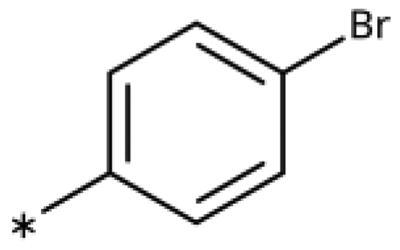		13 (0.8)	0.48
**110**	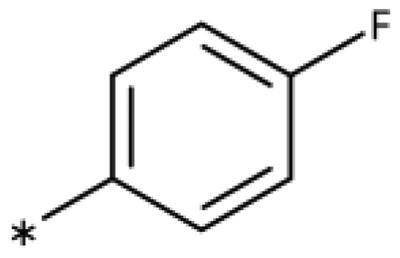		190 (10)	0.36
**111**	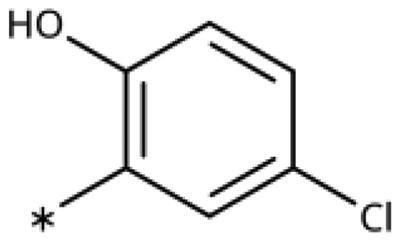		370 (30)	0.31
**112**	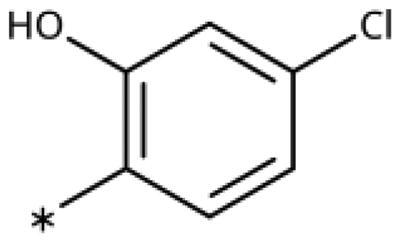		11 (0.2)	0.45
**113**	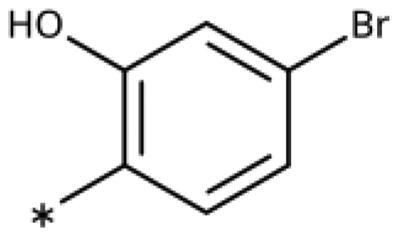		8.2 (1)	0.46
**114**	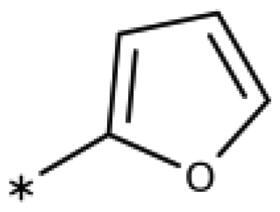		230 (20)	0.41
**115**	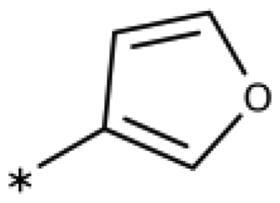		16 (0.7)	0.55
**116**	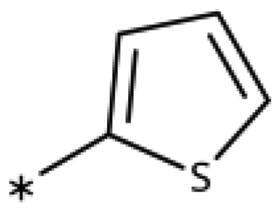		17 (1)	0.54
**117**	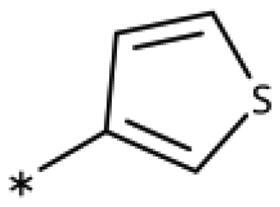		68 (3)	0.47
**118**	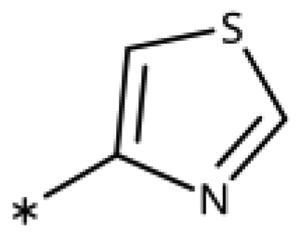		82 (6)	0.46
**119**	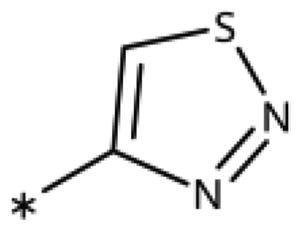		510 (100)	0.37
**120**	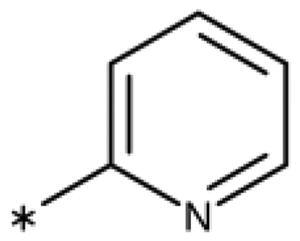		>1000	
**121**	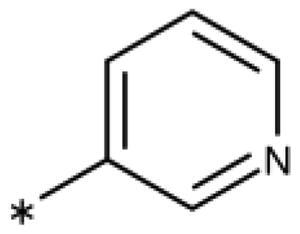		650 (100)	0.33
**122**	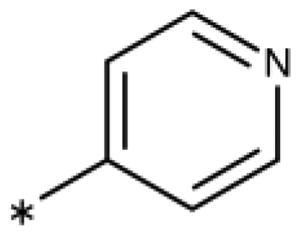		>1000	
**123**	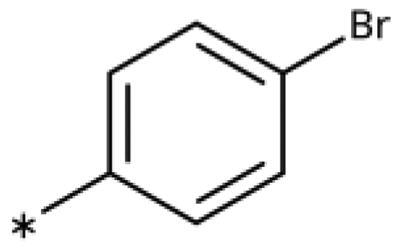	–CH_2_OH	>1000	
**124**	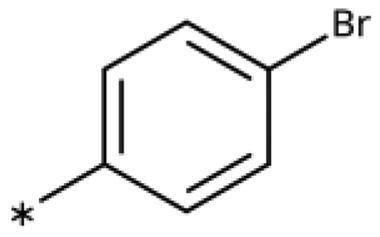	–CH_2_Br	>1000	
**126**	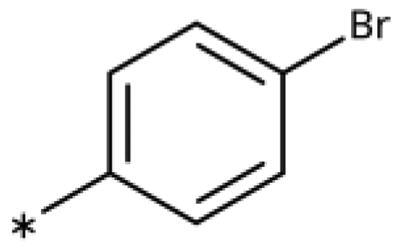	–CH_2_NH_2_	>1000	

^a^ Standard Deviation (SD). ^b^Ligand Efficiency (LE).

**Scheme 8.**
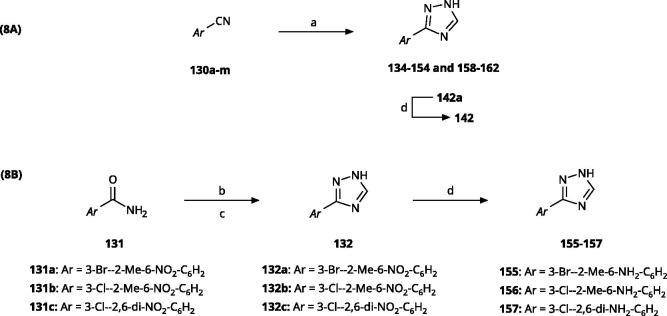
Synthesis of 3-aryl-1*H*-1,2,4-triazoles. Reagents and conditions: (a) Formic acid, N_2_H_4_·H_2_O, DMF, 90 °C, 12 h, yield 20–45%. (b) *N,N*-dimethyl formamide-dimethyl acetal (DMF-DMA), reflux, 1 h. (c) N_2_H_4_·H_2_O, AcOH, 90 °C, 1.5 h, overall yield 70-80% for 2 steps. (d) SnCl_2_·2H_2_O, EtOH, 70 °C, 1 h, yield 40–66%.

**Scheme 9.**
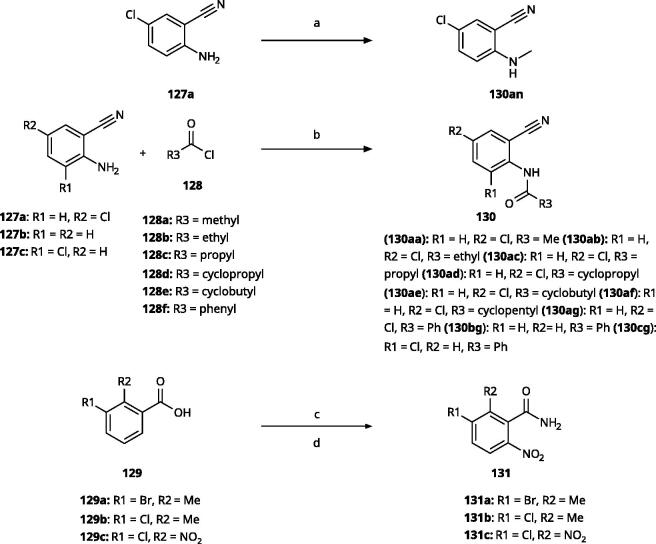
Synthesis of aryl carbonitriles and aryl carboxamides. Reagents and conditions: (a) t-BuOK, dimethyl oxalate, *N,N*-dimethyl acetamide, 140 °C, 5 h, yield 70%. (b) pyridine, DMAP, rt, 3 h, yield 75–85%. (c) HNO_3_, H_2_SO_4_, rt, 3 h (d) (COCl)_2_, NH_4_OH, DMF, DCM, reflux, overall yield 70–75% for 2 steps.

The 3-aryl-1*H*-1,2,4-triazoles (Tables 5 and 6) were prepared according to Scheme 8. Compounds **134**–**154** and **158**–**161** were prepared by reaction of the respective carbonitrile **130** with formic acid and hydrazine hydrate in DMF at 90 °C (Scheme 8A)^80^. The 1,2,4-triazoles **155**–**157** were obtained from benzamides **132a**–**132c** through condensation with *N,N*-dimethylformamide dimethyl acetal (DMF-DMA), followed by cyclisation with hydrazine hydrate in acetic acid at 90 °C (Scheme 8B)^81^^,^^82^. The corresponding carbonitriles and benzamides were prepared according to Scheme 9.

**Table 5.** 3-Aryl-1,2,4-Triazoles. 
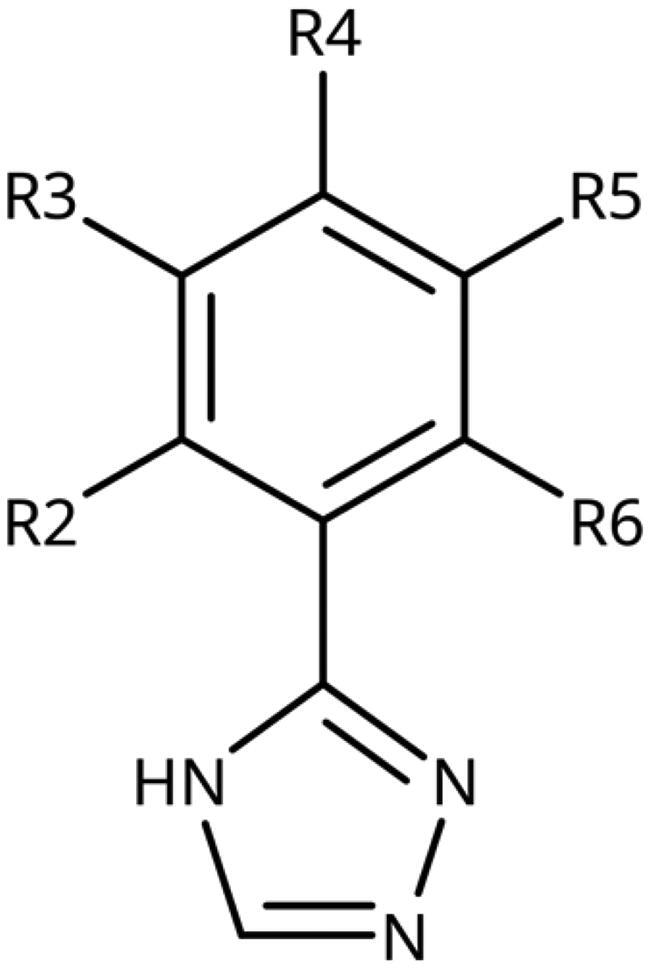

Compound	R2	R3	R4	R5	R6	IC_50_ [μM] (SD^a^)	LE^b^ [kcal/mol/HA]
**133**						>1000^28^	
**134**			–F			>1000	
**135**				–Br		16 (1)	0.55
**136**				–Cl		31 (4)^28^	0.51
**137**				–F		160 (20)	0.43
**138**	–OH					31 (1)^28^	0.51
**139**	–NH_2_					2.0 (0.7)^28^	0.65
**140**	–NH_2_	–F				0.81 (0.2)	0.64
**141**	–NH_2_	–Cl				29 (3)	0.48
**142**	–NH_2_				–NH_2_	31 (7)	0.47
**12**	–NH_2_			–Br		0.020 (0.001)^28^	0.80
**11**	–NH_2_			–Cl		0.035 (0.004)^28^	0.78
**143**	–NH_2_			–F		0.15 (0.006)	0.72
**144**	–NH_2_		–F	–Br		0.034 (0.004)	0.73
**145**	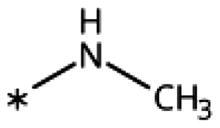			–Cl		8.7 (3)	0.49
**146**	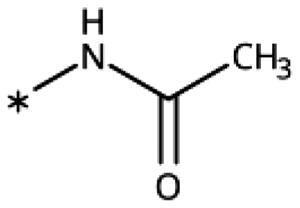			–Cl		420 (90)	0.29
**147**	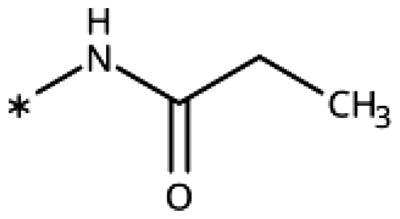			–Cl		1.2 (0.04)	0.47
**148**	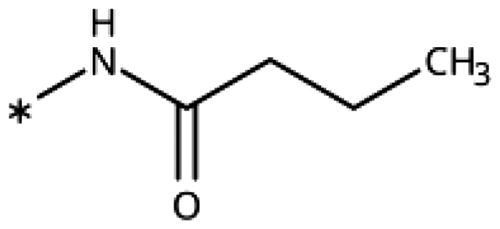			–Cl		25 (7)	0.35
**149**	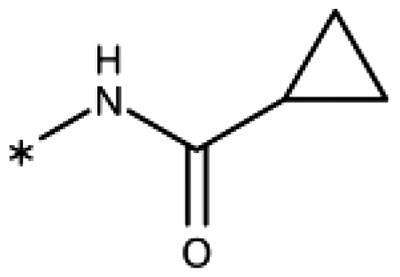			–Cl		0.49 (0.05)	0.48
**150**	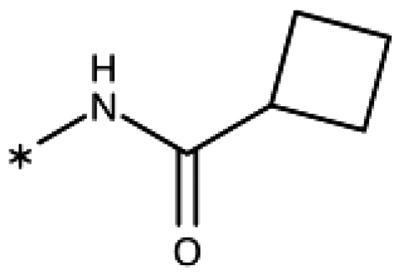			–Cl		>100	
**151**	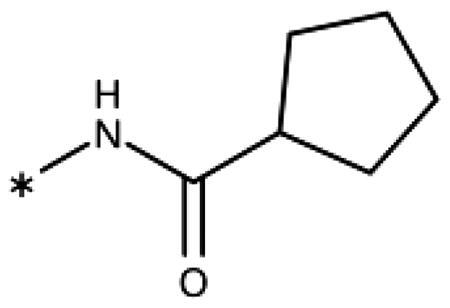			–Cl		>100	
**152**	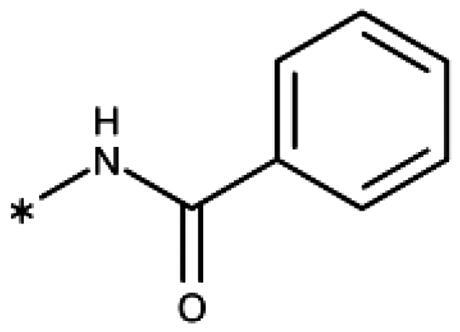					>100	
**153**	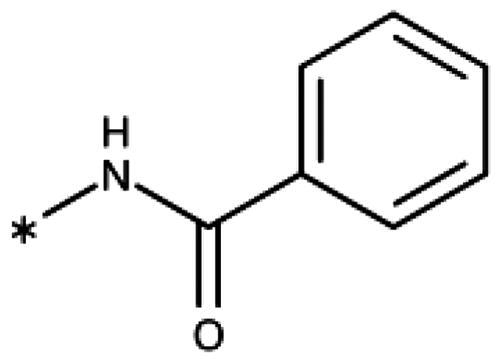			–Cl		9.3 (0.3)	0.33
**154**	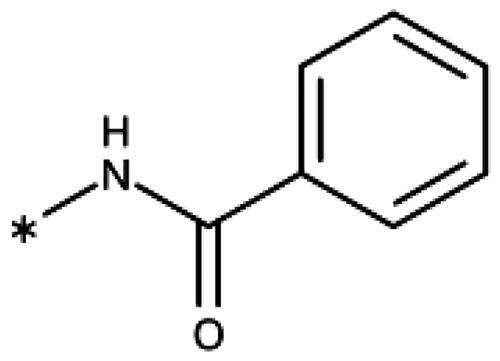	–Cl				>1000	
**155**	–NH_2_			–Br	–CH_3_	63 (10)	0.41
**156**	–NH_2_			–Cl	–CH_3_	96 (3)	0.39
**157**	–NH_2_			–Cl	–NH_2_	1.0 (0.02)	0.55

^a^ Standard Deviation (SD). ^b^Ligand Efficiency (LE).

**Table 6.** 3-Hetaryl-1,2,4-triazoles. 
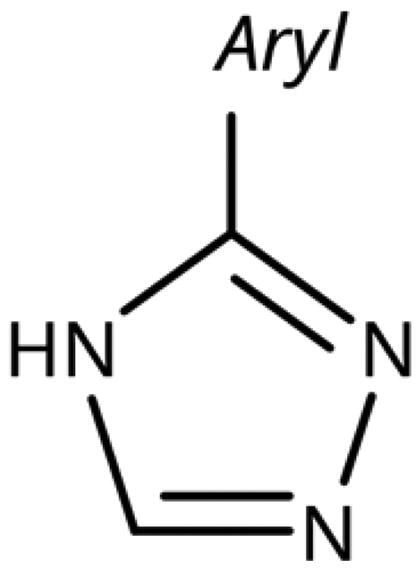

Compound	Aryl	IC_50_ [μM] (SD^a^)	LE^b^ [kcal/mol/HA]
**158**	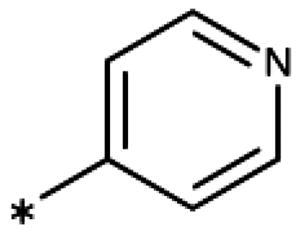	>1000	
**159**	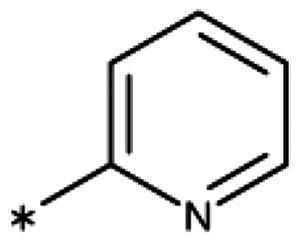	>1000	
**160**	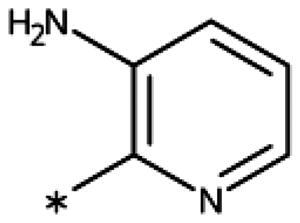	16 (7)	0.55
**161**	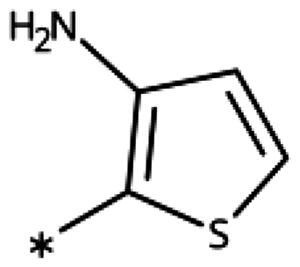	10 (2)	0.57
**162**	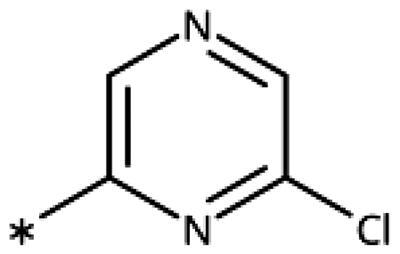	250 (10)	0.41

^a^ Standard Deviation (SD). ^b^Ligand Efficiency (LE).

**Scheme 10.**
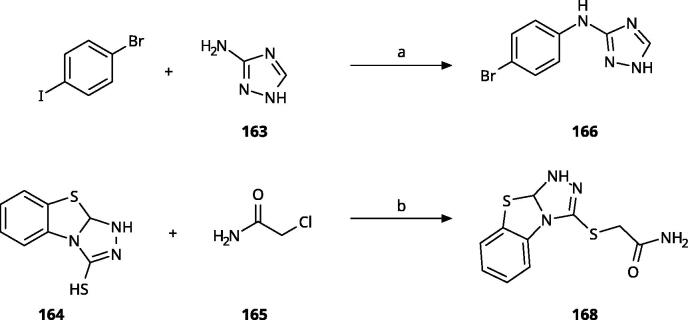
Synthesis of other 1,2,4-triazoles. Reagents and conditions: (a) K_2_CO_3_, CuI, *N,N*-dimethyl acetamide, 90 °C, 48 h, yield 39%. (b) K_2_CO_3_, acetone, reflux, 9h, yield 60%.

Other 1,2,4-triazoles (Table 7) were prepared according to Scheme 10. 3-(4Bromophenylamino)-1,2,4-triazole (**166**) was synthesised by reaction of 1-bromo-4iodobenzene (**163**) with 1*H*-1,2,4-triazole-3-amine in the presence of CuI and K_2_CO_3_ in DMA at 90 °C^83^. The annulated derivative **168** was synthesised by the S*_N_*2 displacement of 2-chloroacetamide **165** using thiol **164** in the presence of K_2_CO_3_ in acetone^84^.

**Table 7.** Other 1,2,4-triazoles.

Compound	Structure	IC_50_ [μM] (SD^a^)	LE^b^ [kcal/mol/HA]
**166**	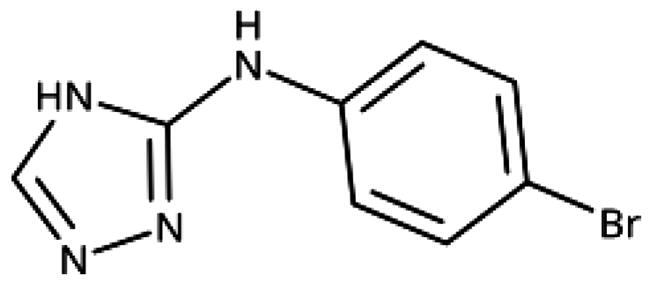	>1000	
**167**	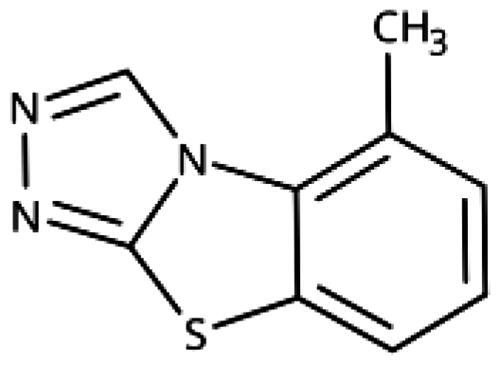	>1000	
**168**	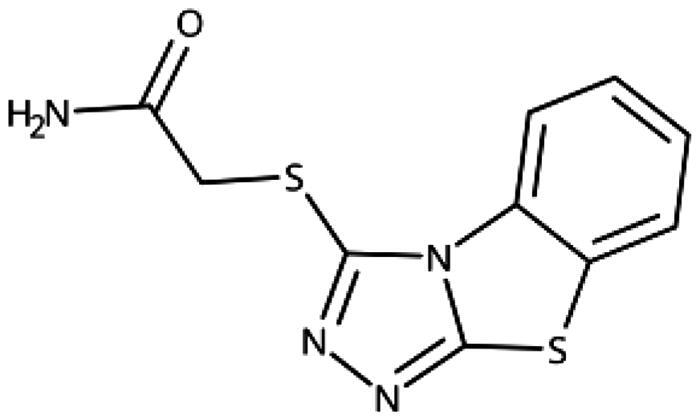	>1000	
**169**	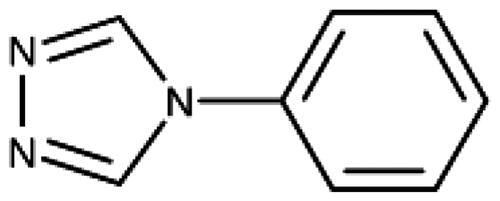	>1000	
**170**	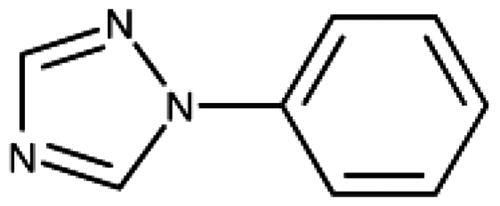	870	0.38
**171**	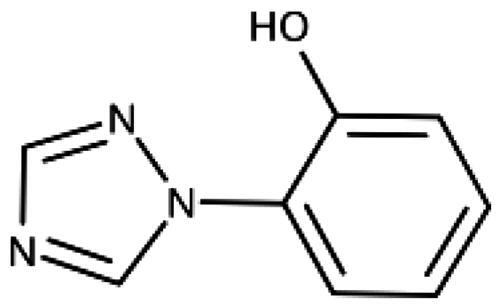	27	0.52

^a^ Standard Deviation (SD). ^b^Ligand Efficiency (LE).

Compounds **9**, **19**, **21**, **22**, **29**, **30**, **31**, **32**, **33**, **46**, **47**, **61**, **81**^58^, **13**, **40**, **41**, **42**^62^, **11**, **12**, **136**, **138**, **169**^28^, and **10**^22^ have been described previously by us. Compounds **79**, **80**, **88**, **133**, **139**, **167**, **170**, and **171** were commercially available.

## Results and discussion

### Structural data

#### MMG-0472–bound structure

**Figure 2.**
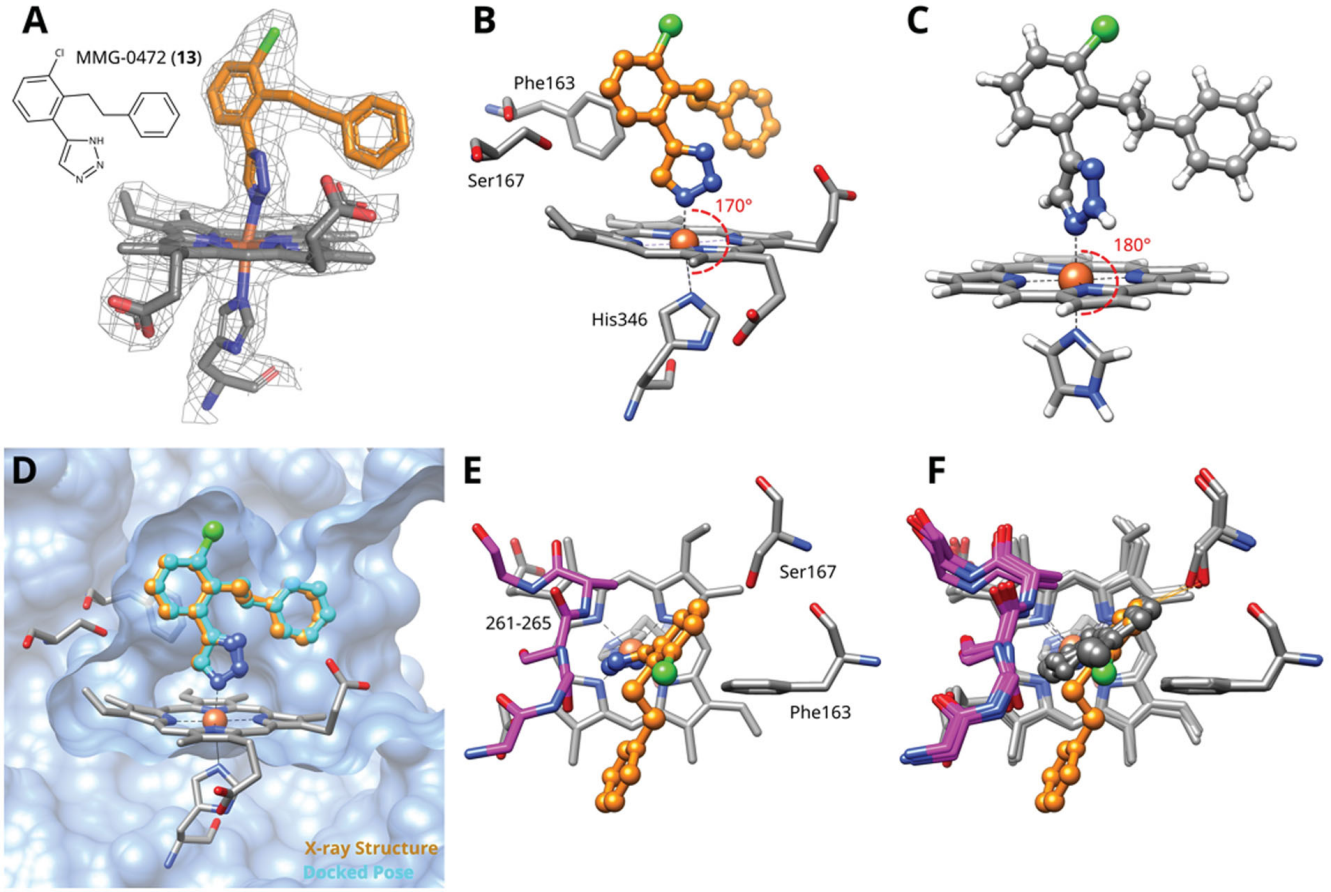
(A) Detail of X-ray structure of compound MMG-0472 (**13**, orange) bound to IDO1 (PDB ID 7zv3). The 2*F_o_*−*F_c_* map of the active site is contoured at 1.0 *σ*. (B) Same Xray structure, highlighting the His346–iron–ligand bond angle of 170°. (C) DFT-optimized model of MMG-0472 binding to haem. The imidazole–iron–ligand bond angle of 180° is highlighted. (D) Superposition of the X-ray structure of MMG-0472 (orange) and its binding pose predicted by docking (cyan). The RMSD between the structures is 0.3 Å. (E) Top view of MMG-0472 X-ray structure, showing the passage from pocket A to pocket B in-between residues Phe163 on the one side and residues 261–265 (magenta) on the other side. (F) Same view, superimposed with X-ray structures of compounds **9**, **11** and **12** (PDB ID 6r63, 7ah5, 7ah6)^22^^,^^28^.

MMG-0472 (**13**, Figure 2) is a 4-phenyl-1,2,3-triazole featuring an extension on the phenyl ring, designed to be located in pocket B. We first described this compound in 2016, when we tested it in cellular assays for hIDO1 and for mIDO2 inhibition. MMG-0472 showed a high cytotoxicity (70% at 200 *µ*M) and was not further pursued for this reason^62^. The mechanism behind this cellular toxicity remains to be clarified and could be due to solubility issues based on our experience with similar compounds. Here, we proceeded to test this compound in the enzymatic assay and found it to be one of the most potent azoles with B pocket extension with an enzymatic IC_50_ value of 14 *µ*M and a LE of 0.33 kcal/mol/HA.

We also investigated compound **13** by X-ray crystallography and obtained diffracting crystals of its complex with IDO1 (PDB ID 7zv3). The 2F*_o_*–F*_c_* map of the IDO1 bound ligand clearly shows its electron density in both pockets A and B (Figure 2A). The ligand is non-planar, with the central bond between the triazole ring and the chloro-phenyl displaying a dihedral angle of 39°. The His346–iron–ligand bond angle is 170° (Figure 2B), deviating by 10° from its optimal value of 180° as determined by density-functional theory calculations on a haem model system (Figure 2C). This deviation probably reflects some strain in the complex. The resolved ligand structure is very close to our docking prediction (Figure 2D) with a root-mean-square distance (RMSD) of 0.3 Å.

As it can be appreciated from the top view and superimposition with the X-ray structures of triazoles **9**, **11** and **12** (Figure 2D,E), the phenyl ring in compound **13** is rotated away from Ser167 and towards pocket B by about 20° with respect to the phenyl rings of the other compounds. Therefore, an additional 2-hydroxy substituent on the phenyl ring would not be favourable in these types of compounds, because they would produce a clash between the B-pocket extension and residues 261–265. The structural data for compound **13** thus confirms our previous analysis showing that azole ligands are not optimally suited to extend into pocket B^27^.

#### Enzymatic activities

Enzymatic IC_50_ values were measured with the ascorbate/methylene blue reduction system^85^ in presence of a non-ionic detergent to reduce compound aggregation as described before^28^. Kynurenine and L-Trp concentrations were determined by HPLC through UV detection. Dose-response curves and Hill slopes can be found in the Supplementary Information (Figure S2). Generally, IC_50_ values are slightly lower here than in our first work on 1,2,3-triazoles^58^, as we shortened the incubation time to stop the reaction in its linear phase. If compound solubility allowed, compounds were tested at concentrations up to 1 mM in presence of 5% of DMSO co-solvent.

#### 4-Aryl-1,2,3-Triazoles with monosubstituted phenyl groups

**Figure 3.**
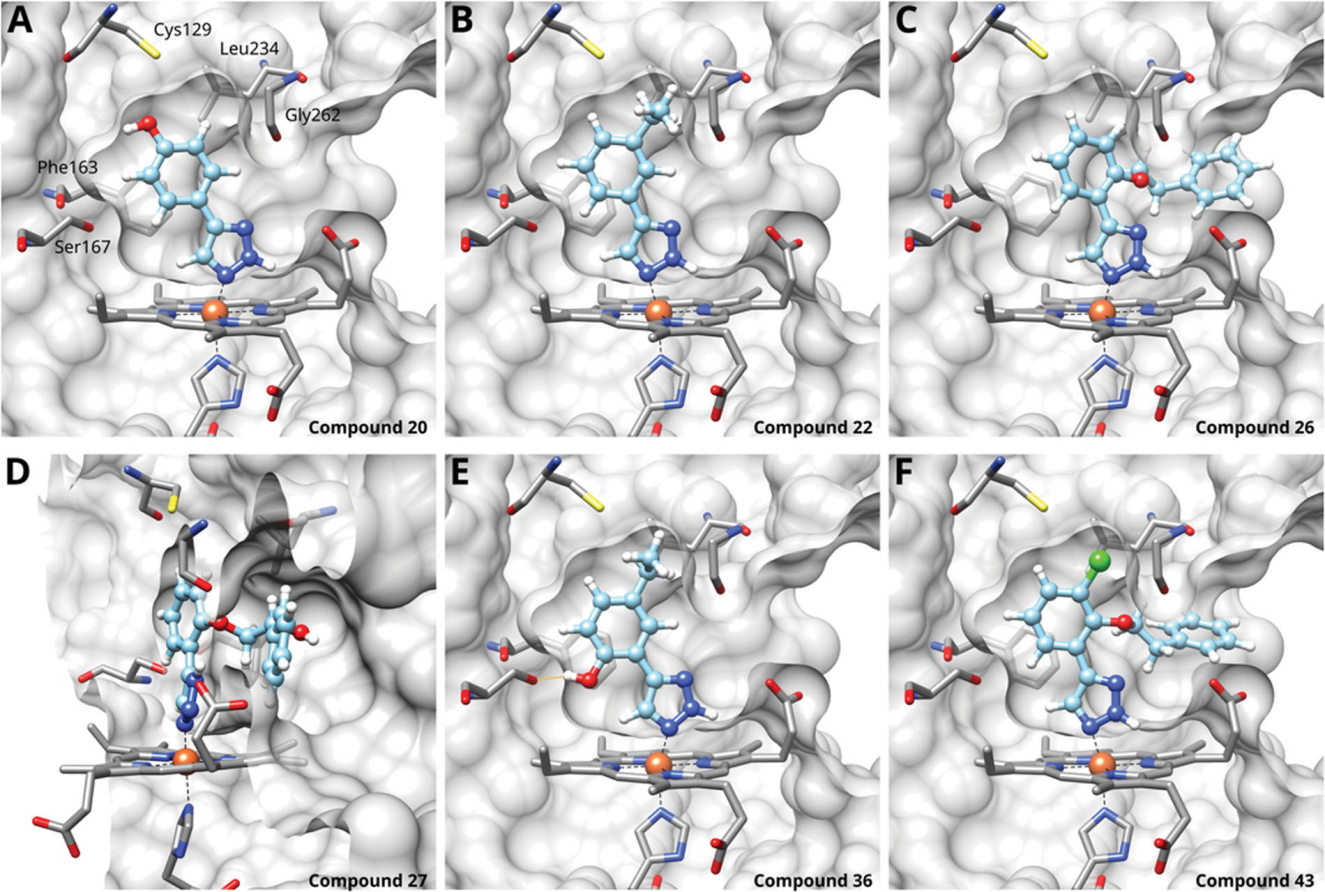
Binding poses of 4-aryl-1,2,3-triazoles predicted by docking. (A) Compound **20**: non-optimal pose, the imidazole-iron-triazole bond angle deviates from 180° due to limited space for the *p*-OH substituent. (B) Compound **22**: the *m*-ethyl substituent is of good size for the hydrophobic subpocket between Val125, Cys129, Leu234 and Gly262. (C) Compound **26**: inactive compound with larger *ortho* substituent using an ether linker. (D) Compound **27**: no favourable interactions between hydroxy group and pocket B. (E) Compound **36**. (F) Compound **43**.

We previously showed that 1,2,3-triazoles with *para*-substituted phenyl rings consistently had lower inhibitory activities on IDO1 than unsubstituted compounds, their IC_50_ values increasing from 10 *µ*M (H)^28^ to 190 *µ*M (F), 530 *µ*M (Cl), 1 mM (CH_3_) to above 1 mM (CF_3_)^58^ for non-polar substituents. Here, we synthesised and tested one compound with the polar *p*-OH substituent (**20**), which was inactive (Table 1). This result shows that, in agreement with what has been found for imidazoles^41^, also polar substituents in this position are unfavourable. This is in agreement with docking predictions, showing little space and hydrophobic groups surrounding *para* substituents (Figure 3A).

On the other hand, we have previously found that non-polar substitutions in *meta* position increased the activity and were most favourable for chloride (0.35 *µ*M)^28^, while hydroxy (310 *µ*M) and amino (*>*1 mM) substituents decreased activity^58^. Similar effects were found for substituents in this position in imidazoles^24^^,^^41^ and hydroxyamidines^30^. Here, we tested more aliphatic substitutions of different sizes and found the ethyl substituent (**22**, 13 *µ*M, Figure 3B)^58^ to show the best activity, better than methyl (**21**, 22 *µ*M)^58^, *n*-butyl (**24**, 34 *µ*M), and *i*-propyl (**23**, 280 *µ*M, Table 1). For larger substituents, there is not enough space in this position, as suggested by the structural analysis.

Earlier investigations of *ortho* substitutions in the 4-phenyl-1,2,3-triazoles showed that only the hydroxy substituent substantially increased the activity (**29**, 2.3 *µ*M), attributed to the formation of a hydrogen bond with Ser167 in the back of the binding site^28^. Especially larger substituents, designed for targeting pocket B, showed substantially decreased inhibitory activities besides lowering the solubility of the compounds^58^^,^^62^. Here, we show that usage of an ether (**25**, **26**, **27**, Figure 3C) or an amino linker (**28**) to a large substituent in this position does not yield active compounds either (Table 1). This is also true when introducing a hydroxy group (**27**), expected to increase solubility and allowing to test the inhibitor at higher concentrations. However, it should be noted that docking predicts that this hydroxy group cannot form favourable interactions within pocket B (Figure 3D).

#### 4-Aryl-1,2,3-Triazoles with double and triple substituted phenyl groups

As described previously, the 2,5-disubstituted compound MMG-0358 (**9**, Figure 1, IC_50_ = 0.059 *µ*M, LE 0.76 kcal/mol/HA) displayed a very high inhibitory activity, explained by the interactions of its hydroxy subsituent with Ser167 and of its chloride substituent with the hydrophobic subpocket around Val125, Cys129, Leu234, and Gly262 as confirmed by X-ray crystallography (Figure 1)^22^^,^^58^. Compounds **31**, **32** and **46** with the 2-hydroxy substituent replaced by amino, methyl, or chloride substituents, were less active but still displayed low micromolar IC_50_ values, while **33** with inverted polar/non-polar substituents (5-amino-2-methylphenyl instead of 2-amino-5-methylphenyl) was completely inactive^58^. Here, we extended our investigation with 2-hydroxyphenyl derivatives bearing various 5-substituents. 4-Aryl-1,2,3-triazoles with small non-polar 5-substituents up to the size of propyl were inhibitors in the nanomolar range (**34**, **35**, **36**, **37**, LE 0.72 to 0.56 kcal/mol/HA), while analogues with larger substituents such as 5–(2-benzyl) and 5–(2-phenylethyl) (**38**, **39**, LE 0.26 to 0.29 kcal/mol/HA) showed lower activities (Table 1). In our docking approach, only substituents in 5-position up to the size of ethyl docked well (Figure 3E). Other 5-chlorophenyl analogues 2-substituted with longer alkyl chains have been described previously (**13**, **40**, **41** and **42**) but were tested only in a cellular environment on IDO1 and on IDO2^62^. Compound **42** inhibited both human IDO1 and murine IDO2 with a cellular IC_50_ value of 110 µM. Here, we also measured their enzymatic activities and found them to have a low LE (0.24 to 0.36 kcal/mol/HA). We additionally synthesised and tested 4-[5-chloro-2–(2-phenylethoxy)phenyl]-1,2,3-triazole (**43**), which is also a micromolar inhibitor (31 µM, LE 0.29 kcal/mol/HA, Figure 3F). We also tested two 2,6-disubstituted compounds, **44** (13 µM) and **45** (no inhibition, NI). As in the case of imidazoles^41^, the 2,6-di-OH substituted compound is at least 2 orders of magnitude more active than the 2,6-di-OCH_3_ substituted compound, but it is less active than the single OH-substituted compound **29**.

Finally, we synthesised and tested a series of dichloro and trichloro substituted compounds. The most active compound was the 2,5-disubstituted compound **46** (6.4 *µ*M), followed by **47** (23 *µ*M), **48** (31 *µ*M), **52** (33 *µ*M), **49** (51 *µ*M), **50** (58 *µ*M), **51** (280 *µ*M), and **53** (NI). In agreement with the docking predictions, all compounds are less active than the 5-Cl single-substituted compound (**30**, 0.35 *µ*M)^28^, and the third chloride substituent does not increase activity with respect to the di-substituted compounds.

#### 5-Substituted 4-Aryl-1,2,3-Triazoles

Theoretically, access to the B-pocket could also be achieved through a substitution either of the N3 or the C5 atoms of the 1,2,3-triazole ring (Figure 1D). We explored the possibility of nitrogen substitutions before, without obtaining any active compound^58^. This finding supports the importance of an ionisable NH group in the triazole and the hypothesis that deprotonation of the 1,2,3-triazole is crucial for IDO1 inhibition. Here, we further explored substitution of the C5 atom (Table 2), although this enforces iron binding through the N2 atom (Figure 1D), which has been calculated to be less favourable than binding through the N1 atom^28^^,^^58^. We previously described one compound of this type with a 5-methyl substituent (**61**, NI)^58^. Here, we show that an electron-withdrawing 5-nitrile substitution yielded a slightly active compound (**62**, 630 *µ*M), while combination of the 5-methyl substituent with the 5-Cl substituent on the phenyl ring yielded an inactive compound (**63**, NI). Compounds with this substitution pattern have recently been further explored by Panda and co-workers^59^, who described 11 compounds with nanomolar IC_50_ values. We synthesised three of the compounds described in this work (original compound numbers: 1d, **64**; 2 g, **65**; and 3 g, **66**) but could not reproduce the published IC_50_ values. In our hands, compounds **64** and **65** showed no inhibition of IDO1, although their published IC_50_ values were 2.51 *µ*M and 0.39 *µ*M, respectively^59^. Compound **66**, which has a published IC_50_ value of 0.62 *µ*M, showed a weak activity in our tests, with an IC_50_ value of 800 *µ*M. The lower activities might be due to higher purities of our compounds and are in line with weak activities measured for other compounds of this type (Table 2). Even combination with the highly activating 2-OH,5-Cl substitutions on the phenyl ring (**67**, **68**) yielded at best a compound with an IC_50_ value in the high micromolar range.

These compounds cannot meaningfully be docked into the IDO1 active site, the best poses displaying a triazole–haem angle deviating significantly from the optimal angle of 90° (Supporting Information, Figure S1).

#### 4-Aryl-Tether-1,2,3-Triazoles and annulated derivatives

**Figure 4.**
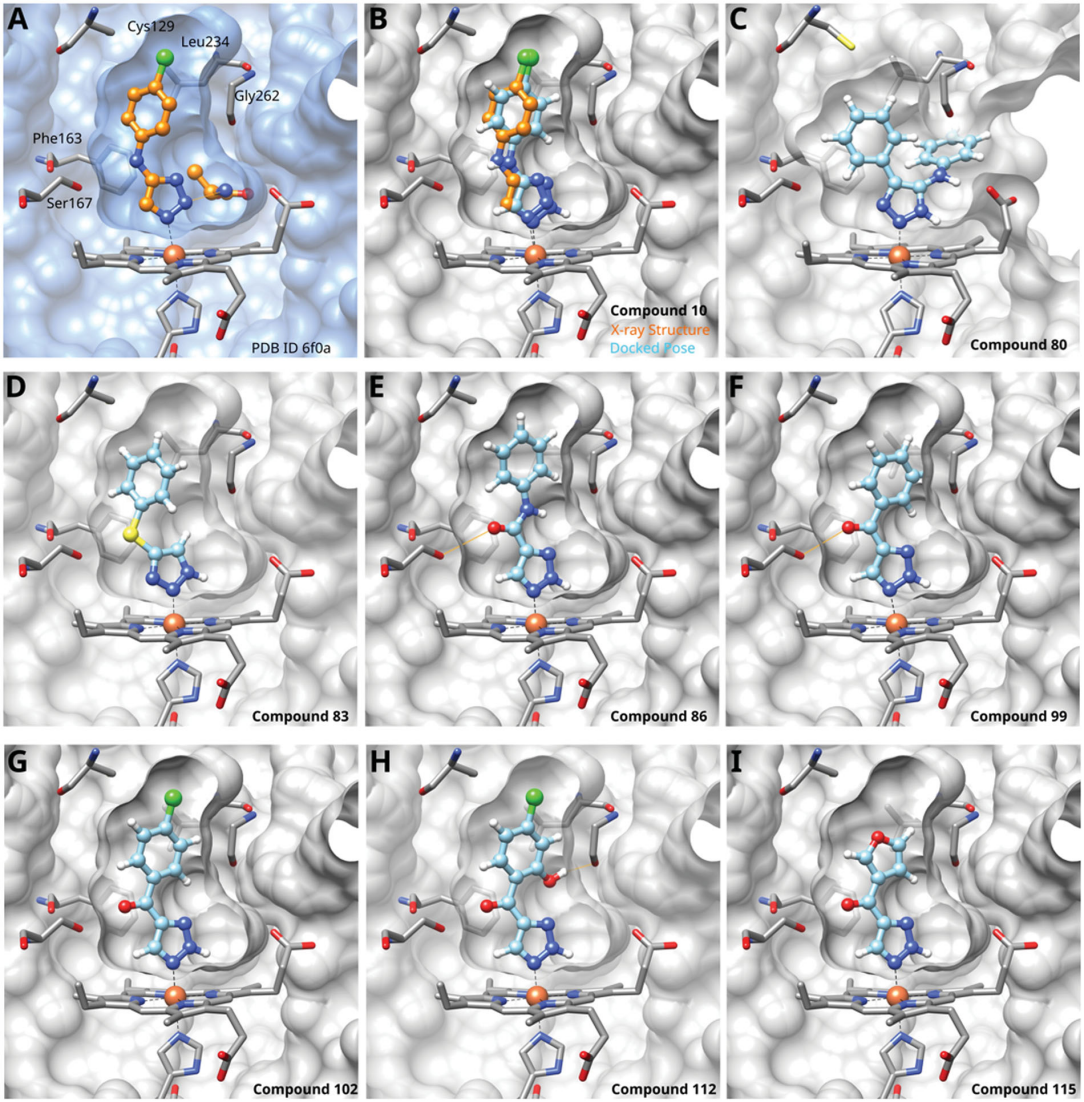
Tethered 1,2,3-triazoles. (A) X-ray structure of compound **10** (PDB ID 6f0a)^60^. (B) Superposition of X-ray structure and of docking pose of **10**. Docking poses of (C) compound **80**; (D) compound **83**; (E) compound **86**; (F) compound **99**; (G) compound **102**; (H) compound **112**; (I) compound **115**. Hydrogen bonds are shown in orange.

The 1,2,3-triazole published by Alexandre and co-workers (**10**, Figure 1), which features an amino linker between the triazole and the 4-chlorophenyl moiety, shows a peculiar behaviour^60^^,^^70^^,^^86^. X-ray data demonstrates haem-iron binding and occupancy of pocket A (Figure 4A), but a slow shift of the haem Soret peak in UV-absorption spectra under reductive conditions coupled with haem unbinding and degradation as well as the largely superior activity in cellular assays as compared to enzymatic assays is very distinct from the behaviour of the 4-aryl-1,2,3-triazoles^22^^,^^60^.

Building on these results, we synthesised and tested 1,2,3-triazoles linked to aryl moieties by a one-center tether (N, CH_2_, O, S, SO_2_, CHOH) or two-atom tether (CONH, Table 3). In our hands, compound **10** displayed an IC_50_ value of 56 *µ*M (11.3 *µ*M in the original publication). 4–(4-Bromophenylamino)-1,2,3-triazole (**78**) showed an inhibitory activity of 52 *µ*M, whereas the 4-phenylamino analogue (**79**) was not active. These 3 latter compounds dock well into the IDO1 active site, although the phenyl ring adopts a slightly rotated conformation with respect to the X-ray structure (Figure 4B). Compound **80** with an additional phenyl substituent on the C5 of the triazole ring rather docks with the amino linker pointing towards the haem propionate and pocket B but shows no activity in the enzymatic assay (NI, Table 3, Figure 4C), similar to other C5-substituted 1,2,3-triazoles (Table 2).

Replacement of the amino linker by a methylene group (**81**, NI), by an ether group (**82**, NI), by a sulphide group (**83**, 590 *µ*M, Figure 4D), by a sulfoxide moiety (**84**, NI), by a hydroxymethylene group (**85**, NI) or by a carboxamide group (**86**, NI, Figure 4E) generated inactive compounds except for **83** with the sulphur tether (590 *µ*M). Compound **87** with a MeON=C linker was synthesised and tested as another possible access point to pocket B but was inactive. The annulated 1,2,3-triazoles **88** and **89** were also inactive. More interesting results were obtained with 5-aroyl-1,2,3-triazoles (keto linker), preserving the conjugation and the planarity between the aromatic rings (Table 4). Docking results for compound **99** (430 *µ*M) suggest that this compound can form a hydrogen bond with Ser167 (Figure 4F), while the keto group simultaneously increases the acidity of the triazole ring. Starting from this 5-benzoyl-1,2,3-triazole scaffold, we explored different single and double substitutions of the phenyl ring, heterocyclic replacements of the phenyl ring, and 4-substitutions of the 1,2,3-triazole ring. Chloro substitutions in 2-position (**100**, NI), 3-position (**101**, 450 *µ*M) and 4-position (**102**, 31 *µ*M) of the phenyl ring showed that it was favourable only in the latter, in agreement with the analogous amino-1,2,3-triazole and the docking predictions (Figure 4G)^60^. Similar trends were observed for methoxy substitution in 2-position (**103**, NI), 3-position (**104**, 990 *µ*M), and 4-position (**105**, 200 *µ*M). On the other hand, hydroxy substitutions in 2-position (**106**, 125 *µ*M), 3-position (**107**, NI), and 4-position (**108**, NI) showed that it was favourable only in 2-position. In 4-position, substituent influence was the following: Br (**109**, 13 *µ*M) *>* Cl (**102**, 31 *µ*M) *>* F (**110**, 185 *µ*M) *>* OMe (**105**, 200 *µ*M) *>* OH (**108**, NI), similar to what has been found for the 5-position in the 4-phenyl-1,2,3-triazoles. Combination of 2-OH with the 5-Cl substitutions yielded only a moderately active compound (**111**, 365 *µ*M), as expected by the structure-activity relationship (SAR) of the chloro substituents, but combination of 2-OH with 4-Br (**113**, 8.2 *µ*M, LE 0.46 kcal/mol/HA) and with 4-Cl (**112**, 11 *µ*M, LE 0.45 kcal/mol/HA) substantially increased the activities of singly substituted compounds and provided the most active compounds of this type, which remained, however, only in the low micromolar range. In the docked poses, the hydroxy groups of these compounds form a hydrogen bond to Gly262 (Figure 4H). Replacement of the phenyl ring by 5 or 6-membered aromatic heterocycles was mostly beneficial with 5-membered rings (**115**, 16 *µ*M, Figure 4(I); **116**, 17 *µ*M; **117**, 67 *µ*M; **118**, 81 *µ*M; **114,** 230 *µ*M; **119**, 515 *µ*M), reaching a maximal LE of 0.55 kcal/mol/HA for compound **115**. Replacement by 2–(**120**, NI), 3–(**121**, 650 *µ*M), and 4-pyridine (**122**, 1200 *µ*M), however, was detrimental. Substitution of the C4 atom of the 1,2,3-triazole with the aim to reach pocket B were explored combining them with the 4-Br substituted phenyl ring. However, all substitutions, including hydroxymethyl (**123**, NI), aminomethyl (**126**, NI), bromomethyl (**124**, NI), or larger groups (data not shown) yielded inactive compounds, in agreement with the docking predictions, which do not find low-energy poses for these compounds inside the active site.

In summary, we were able to develop original 5-aroyl-1,2,3-triazoles with enzymatic activities in the low micromolar range. However, they did not provide access to pocket B.

#### 3-Aryl-1,2,4-Triazoles

**Figure 5.**
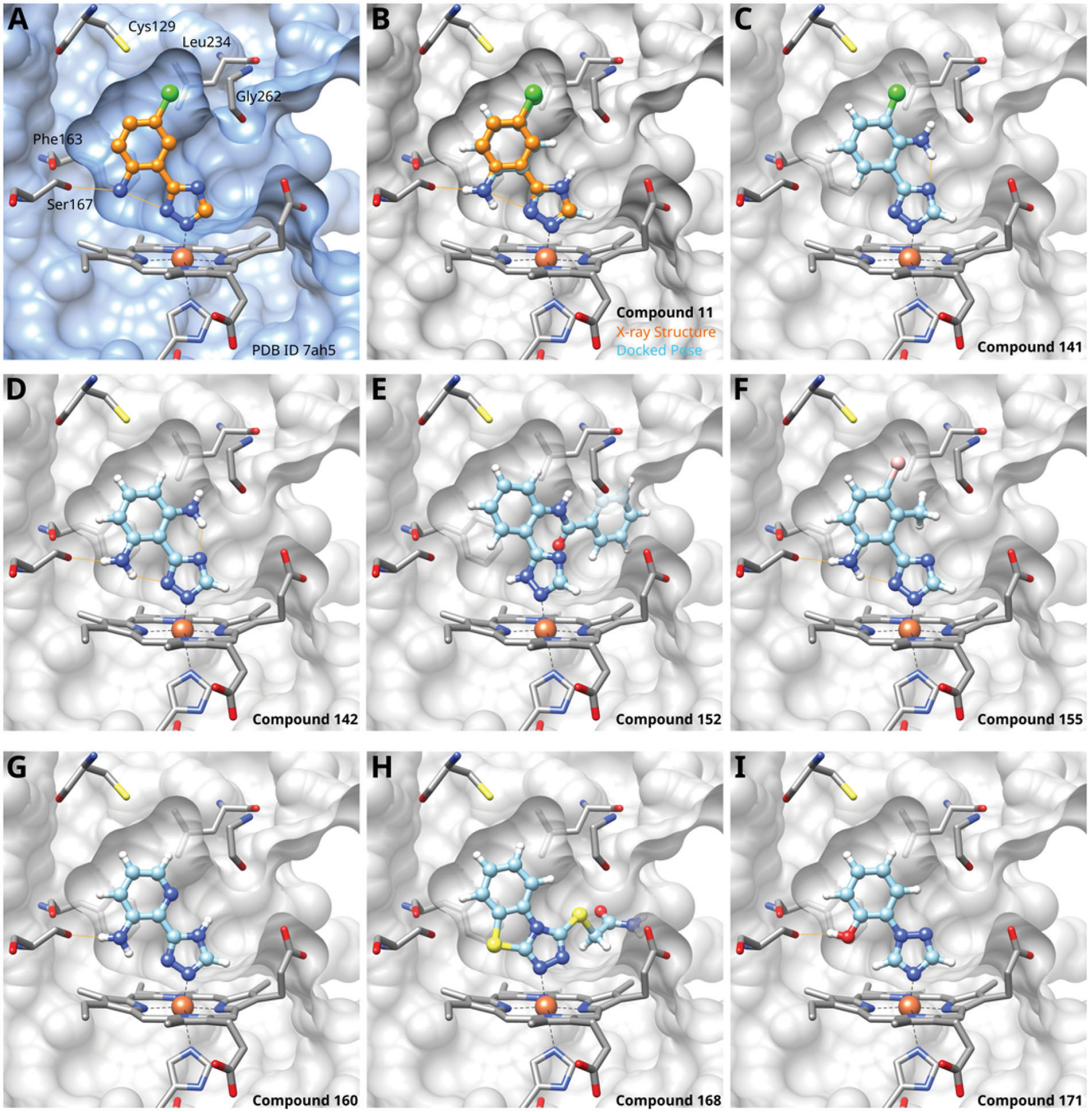
1,2,4-Triazoles. (A) X-ray structure of compound **11** (PDB ID 7ah5)^28^. (B) Superposition of X-ray structure and of docking pose of compound **11**. Docking poses of (C) compound **141**; (D) compound **142**; (E) compound **152**; (F) compound **155**; (G) compound **160**; (H) compound **168**; (I) compound **171**. Hydrogen bonds are displayed in orange.

Since we did not find a 1,2,3-triazole providing a convenient access to pocket B while preserving efficiency and potency, we turned in the following to the 1,2,4-triazoles^28^. For this scaffold, we found previously that 3–(2-aminophenyl)-1,2,4-triazole (**139**) displayed an interesting inhibitory activity of 2 *µ*M (Table 5). This was better than with the 3–(2-hydroxyphenyl) derivative (**138**, 31 *µ*M), preferred by the 1,2,3-triazoles. Based on X-ray crystallography and molecular modelling studies, we attributed these results to the simultaneous inter- and intra-molecular hydrogen bonds of the 2-amino substituent in the 1,2,4-triazole, leading to a more favourable planar conformation (Figure 5A). Most important, combination with the 5-chloro substituent (**11**, 0.035 *µ*M) or the 5-bromo substituent (**12**, 0.020 *µ*M) yielded highly active compounds with excellent LE of 0.78 and 0.80 kcal/mol/HA, respectively^28^.

Here, we started by synthesising and testing the *para*-fluorophenyl derivative (**134**), which was was found inactive and deterred us from preparing more *para*-substituted phenyl derivatives. Based on the knowledge collected for 1,2,3-triazoles, we tested halogen substitutions in *meta* position. We found that the bromo analogue (**135**, 16 *µ*M) was more active than the chloro (**136**, 29 *µ*M) and the fluoro (**137**, 160 *µ*M) substituted compounds. This was expected based on the size of the hydrophobic subpocket in this region and in agreement with the activities found for 1,2,3-triazoles. A 2,6-diamino substituted compound (**142**, 31 *µ*M, Figure 5C) showed decreased activity with respect to the singly substituted 2-amino compound (**139**, 2.0 *µ*M). The 2-NH_2_,5-F compound was consistently less active (**143**, 0.15 *µ*M) than its other halogenated counterparts (compounds **11**, **12**).

We synthesised and tested two compounds with 2-amino,3-halo disubstituted phenyl rings. As expected from our structural analysis, **140** (0.81 *µ*M) and **141** (29 *µ*M, Figure 5D) are much less active than their 2,5-disubstituted counterparts (Table 5). These compounds cannot offer any synergic effects from interactions of the amino group with Ser167 and the triazole ring on the one hand and the interactions of the halogen with the hydrophobic subpocket on the other hand. Methyl substitution of the amino group of compound **11** led to a lower inhibitory activity (**145**, 8.6 *µ*M). This is in agreement with the structural data and the finding that both hydrogen atoms of the amino group serve as hydrogen bond donors^28^.

We also synthesised and tested triple-substituted 3-phenyl-1,2,4-triazoles all having a 2-amino substitution of the phenyl ring. Adding a 4-F substitution to compound **12** only slightly perturbs its activity (**144**, 0.034 *µ*M). However, adding a 6-methyl substitution to either compound **12** (**155**, 63 *µ*M, Figure 5E) or **11** (**156**, 90 *µ*M) reduced the inhibitory activities by more than 3 orders of magnitude. Adding a 6-amino group as in compound **157** (1 *µ*M) also strongly reduced the inhibitory activity as compared to compound **11** (0.035 *µ*M). This mirrors the results obtained with 2-aminophenyl **139** and 2,6-diaminophenyl derivative **142** and leaves little chance to develop analogues of this type extending to pocket B.

Finally, we investigated amide extensions of the *ortho*-amino group with the hope that the latter could reach pocket B, although structural and functional data suggested this amino group to increase activity by being located in the back of the active site and making two simultaneous hydrogen bonds (Figure 5A). We generally found that amide extensions reduced the compound solubility. With 3–(2-benzamidophenyl)-1,2,4-triazole (**152**), we lost the inhibitory activity completely (Figure 5F). Curiously, introduction of a 5-Cl substituent restored some of the inhibitory activity (**153**, 9.4 *µ*M, Table 5) albeit at low solubility. Interestingly, the cyclopropanecarboxamide (**149**, 0.49 *µ*M) and the propenamide (**147**, 1.2 *µ*M) showed the best inhibitory activities of this series. However, the related cyclobutanecarboxamide (**150**, NI) and cyclopentanecarboxamide (**151**, NI) were inactive as inhibitors and showed strongly reduced solubilities. In the aliphatic series, the butanamide (**148**, 24 *µ*M) was better than the acetamide (**146**, 425 *µ*M). Aromatic extensions such as the benzamide (**153**, 9.4 *µ*M) led to lower solubilities. However, the latter could be improved by introducing polar groups. Unfortunately, the inhibitory activities of resulting compounds were only in the high micromolar range (data not shown). Summarising, the observed inhibitory activities of the 1,2,4-triazoles bearing 2-amido substituents cannnot be rationalised based on structural data. Investigations are rendered complicated by the low solubilities of these compounds.

#### 3-Hetaryl-1,2,4-Triazoles

Replacement of the phenyl ring of parent compound **133** by 4-pyridinyl (**158**, NI, Table 6) and by 2-pyridinyl (**159**, NI) produced inactive compounds. However, 3–(3aminopyridinyl)-1,2,4-triazole (**160**, 15 *µ*M, LE 0.55 kcal/mol/HA, Figure 5G) and 3-(3-aminothiophenyl)-1,2,4-triazole (**161**, 10 *µ*M, LE 0.57 kcal/mol/HA) were low micromolar inhibitors with a similar inhibitory activity as the aniline **139**. Some activity was also found with the chloro-pyrazine derivative **162** (250 *µ*M, LE 0.41 kcal/mol/HA). In summary, it is possible to replace the phenyl ring by 5 or 6-membered heteroaromatics, but up to now, no compounds with better activities than the 3-aryl analogues have been found.

#### Other 1,2,4-Triazoles

To complete our investigation with 1,2,4-triazoles as IDO1 inhibitors, we also tested the derivatives shown in Table 7. 3–(4-Bromophenylamino)-1,2,4-triazole (**166**, NI), an analogue of the 1,2,3-triazole derivative **78**, was found to be inactive. This is not surprising, as **78** only shows an IC_50_ value of 52 *µ*M, and the 1,2,4-triazoles are generally less active than the corresponding 1,2,3-triazoles. The annulated antifungal tryclazole (**167**, NI) and its derivative (**168**, NI, Figure 5H), designed to extend into pocket B, were inactive, although they dock well into the active site. Their inactivity is probably due to their low iron affinity. We have shown before that 4-phenyl-1,2,4-triazole (**169**, NI) was inactive^58^. However, as we predicted based on quantum chemical calculations^28^, 1-phenyl-1,2,4-triazole (**170**, 870 *µ*M) showed some activity, which could further be increased by adding an *ortho*-hydroxysubstituent to the phenyl ring (**171**, 32 *µ*M, Figure 5I), making it about equipotent to the 3-phenyl-1,2,4-triazole **138**. Interestingly, this compound binds to IDO1 in its neutral form, as the 1,2,4-triazole cannot be deprotonated, and might therefore display different properties than the derivatives of **133**, which may bind stronger in their deprotonated form to IDO1.

### Cellular activities

**Figure 6.**
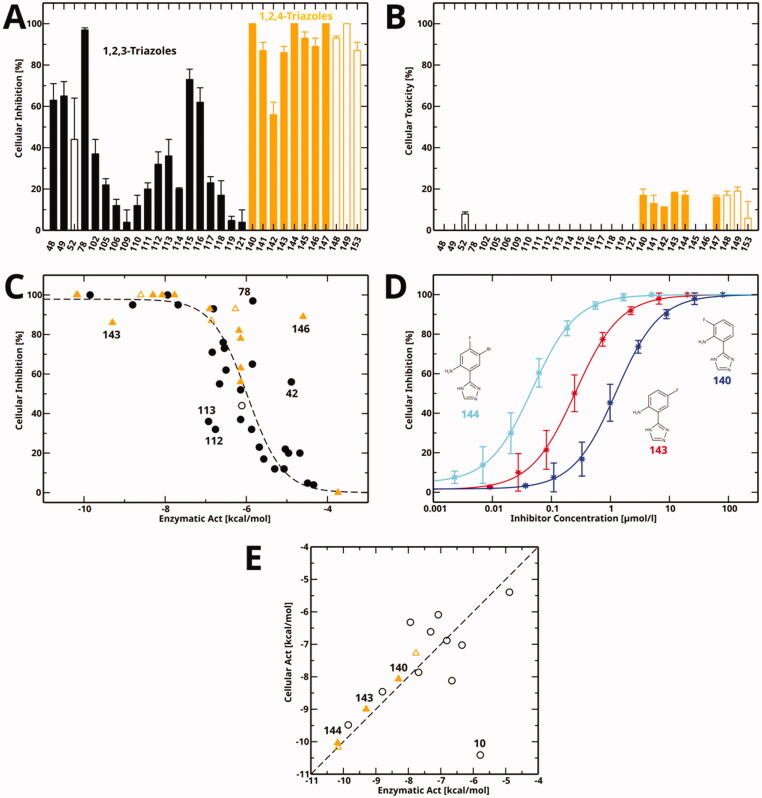
Cellular inhibition and comparison to enzymatic inhibition. (A) Cellular inhibition of kynurenine production at a single compound concentration. Data for 1,2,3-triazole compounds is shown in black, for 1,2,4-triazoles in orange. Values measured at a compound concentration of 200 *µ*M are given in filled bars, values measured at 50 *µ*M in empty bars. (B) Cellular toxicity under the same conditions and using the same colour code as part (A). (C) Cellular inhibition as a function of enzymatic activity (*Act* = *RTlog*(*IC*_50_)). The dashed line is a sigmoidal fit to all data points except for the marked outliers. Compounds measured at a concentration of 50 *µ*M are marked by empty symbols. (D) Cellular dose-response curves of compounds **140**, **143**, and **144** measured in this work. (E) Correlation between cellular activity and enzymatic activity for compounds mentioned in this manuscript. Colour code as in part (A). Filled symbols denote the newly determined data shown in part (D).

We tested the cellular hIDO1 inhibitory activity and toxicity of 30 active compounds at a single concentration (Figure 6 and Table 8). As some of the aniline derivatives reacted with Ehrlich’s reagent (*p*-dimethylaminobenzaldehyde, *p*-DMAB) used to quantify kynurenine, the kynurenine content of these samples was determined by HPLC. To be able to detect also weak inhibition, compounds were tested at the high concentration of 200 *µ*M (Figure 6A and B, filled bars), except for less soluble compounds (**52**, **148**, **149** and **153**), which were tested at a concentration of 50 *µ*M (Figure 6A and B, empty bars). Many compounds showed a good cellular inhibition (Figure 6A) and a low toxicity (Figure 6B). It is noticeable that for the compounds tested here, the 1,2,4-triazoles (orange) generally showed a better cellular inhibition than the 1,2,3-triazoles (black) but also a detectable albeit low toxicity. However, it should be kept in mind that previously tested 1,2,3-triazoles also showed a very good cellular inhibition^58^, and that the toxicity of the 1,2,4-triazoles occurs at a concentration of 200 *µ*M, far above their cellular IC_50_ value.

**Table 8.** Cellular inhibition and toxicity data determined in this work.

Compound	Conc. [μM]	Inh. [%]	Tox. [%]	Cell. IC_50_ [μM] (SD^a^)
**48**	200	63 (8)	0 (0)	
**49**	200	65 (7)	0 (0)	
**52**	50	44 (20)	7.9 (1)	
**78**	200	97 (1)	0 (0)	
**102**	200	37 (7)	0 (0)	
**105**	200	22 (3)	0 (0)	
**106**	200	12 (3)	0 (0)	
**109**	200	55 (10)	0 (0)	
**110**	200	12 (5)	0 (0)	
**111**	200	20 (3)	0 (0)	
**112**	200	32 (6)	0 (0)	
**113**	200	36 (8)	0 (0)	
**114**	200	20 (0.6)	0 (0)	
**115**	200	73 (5)	0 (0)	
**116**	200	62 (7)	0 (0)	
**117**	200	23 (3)	0 (0)	
**118**	200	17 (7)	0 (0)	
**119**	200	4.8 (2)	0 (0)	
**121**	200	3.9 (6)	0 (0)	
**140**	200	100 (0)	17 (3)	0.81 (0.2)
**141**	200	82 (7)	13 (4)	
**142**	200	56 (6)	11 (0.4)	
**143**	200	86 (3)	18 (0.5)	0.15 (0.06)
**144**	200	100 (0)	17 (2)	0.034 (0.004)
**145**	200	93 (3)	0 (0)	
**146**	200	89 (4)	0 (0)	
**147**	200	100 (0)	16 (1)	
**148**	50	93 (1)	17 (2)	
**149**	50	100 (0)	19 (2)	
**153**	50	87 (4)	5.9 (8)	

^a^ Standard Deviation (SD).

Previously determined cellular data for compounds mentioned in this work is given in the Supporting Information, Table S2. Both previously and in the present work, cellular inhibition was observed to be closely related to enzymatic activity for both 1,2,3-triazoles and 1,2,4-triazoles, showing a sigmoidal dependence (Figure 6C). Three outliers show a lower cellular activity than expected, namely two double-substituted 5-aroyl-1,2,3-triazoles (**112**, **113**) and the fluorinated 1,2,4-triazole **143**. On the other hand, three compounds show a higher cellular activity, namely compound **78** of the Vertex scaffold, for which this behaviour has already been documented^60^, the amide 1,2,4-triazole **146**, which might be hydrolized to the highly active **11** inside the cell, and the 1,2,3-triazole **42** for unknown reasons.

Here, we determined cellular IC_50_ values for a selection of three 1,2,4-triazoles (Figure 6D), and found two of them to display nanomolar activities also in a cellular context. As in the enzymatic assay, the most potent compound is **144** (cellular IC_50_ value of 0.034 *µ*M), followed by **143** (0.25 *µ*M) and **140** (1.2 *µ*M).

We previously found a good correlation between enzymatic and cellular IC_50_ values for different azole compounds^28^^,^^58^. Here, we show this correlation (Figure 6E) for all 1,2,3-triazole (black) and 1,2,4-triazole (orange) compounds mentioned in this manuscript. The newly determined cellular IC_50_ values of the 1,2,4-triazoles closely follow this correlation. The only outlier is the original Vertex compound **10**^60^.

## Conclusions

In summary, here we described almost 100 new compounds of the 1,2,3-triazole and the 1,2,4-triazole haem-binding series and tested them for their inhibitory activity on IDO1. They provide highly efficient scaffolds for inhibitors binding to pocket A, which are also very potent in a cellular environment and display low cytotoxicities. The best compound (**144**) displays both enzymatic and cellular IC_50_ values of 34 nM and is therefore more potent and efficient *in vitro* than other frequently used IDO1 inhibitors. We did not measure the activities of these compounds on IDO2 and on TDO to determine their selectivity. However, in our earlier works we found that triazoles such as MMG-0358 with high activities on IDO1 have undetectable activities on TDO (>100 μM)^58^. We also found that MMG-0358 has a more than1000-fold selectivity for IDO1 over IDO2, even though triazoles extending from pocket A to pocket B showed better activity on mouse IDO2 than on human IDO1 in a cellular environment^62^. Based on these observations, it is reasonable to assume that the potent compounds reported here, which are binding only to pocket A, are likely to be highly selective for IDO1 over TDO and IDO2.

Unfortunately, extending these highly efficient compounds into pocket B by one of the attachment points described in Figure 1(D) proved very challenging. We were able to resolve the X-ray structure of the complex of one such compound extending into pocket B (MMG-0472, PDB ID 7zv3). The experimental structure confirms our docking predictions of the binding mode of this compound. However, docking predictions in general are challenging due to the interactions with the haem cofactor in the active site, which is difficult to parameterise classically. For future design of type ii or type iii IDO1 inhibitors, we recommend tackling the issue of addressing pockets A and B simultaneously early on in hit-to-lead optimisation to provide more flexibility for rational compound modifications.

## Experimental section

### Docking

Docking was performed with our in-house docking code AttractingCavities (AC)^87^, which relies on the physical scoring function of the CHARMM27 force field^88^^,^^89^, while solvation effects are taken into account by the Fast Analytical Treatment of Solvation (FACTS) model^90^, which has been shown to allow for accurate docking results^91^. Ligand force-field parameters were derived with the SwissParam tool^92^. Standard parameters were used, i.e. a cubic search space of 20 Å^3^ around the IDO1 active site, a rotational angle of 90° for initial ligand sampling, and a *N_Thr_* value of 70 for determination of the attractive grid points. For all compounds with an acidic proton, both the neutral and the deprotonated species were docked, and different tautomers were considered. A Morse-like metal binding potential (MMBP) was used to describe the interactions between the haem iron of IDO1 and ligand atoms that display a free electron pair for iron binding^93^. The protein was kept fixed during the docking. We used seven different IDO1 X-ray structures as targets, chosen for their quality and diversity, namely PDB ID 2d0t^40^ co-crystallized with a small azole ligand in pocket A, 5whr^25^ with a resolved JK-loop*^C^* in closed conformation, 6e41^94^ with an analogue of epacadostat in pockets A and B, 6kof^51^ with a large azole ligand in pockets A and B, 6o3i^24^ with the clinical compound navoximod, 6pu7^32^ with a hydroxyamidine ligand of peculiar shape, and 7ah4^28^ with two small azole ligands in pockets A and D. All ligands were removed before docking.

### Density functional theory calculations

Quantum chemical geometry optimizations and charge calculations were carried out in the density functional theory (DFT) framework with the PBE0 hybrid functional^95^ using the Gaussian16 code^96^. Geometry optimizations were carried out with standard settings and the TZVP basis set^97^. Solvation effects were taken into account by the polarisable continuum model^98^ as implemented in Gaussian16. The histidine-bound haem complex of IDO1 was modelled by an iron–porphin–imidazole system. For the 6-fold coordinated systems, a low-spin complex was assumed, as it has been found experimentally^39^.

### Chemistry

#### General remarks

All reactions were carried out under nitrogen atmosphere unless otherwise stated. Glassware was oven-dried (120 °C), evacuated and purged with nitrogen. Any common reagents, catalysts and solvents that were obtained from commercial suppliers were used without any further purification. For extraction and chromatography, all solvents were distilled prior to use. Thin layer chromatography (TLC) for reaction monitoring was performed on silica gel plates (Merck 60 F254) with detection by UV light (254 nm). Flash chromatography (FC) was conducted using silica gel 60 Å, 230–400 mesh (Merck 9385). Mass spectra were recorded on a Nermag R10-10C instrument in chemical ionisation mode. Electrospray mass analyses were recorded on a Finnigan MAT SSQ 710 C spectrometer in positive ionisation mode. ^1^H and ^13 ^C NMR spectra were recorded with a Bruker-DPX-400 or Bruker-ARX-400 spectrometer at 400 MHz and 100.6 MHz, respectively. Data for ^1^H NMR spectra are reported as follows: chemical shift, multiplicity, apparent coupling constant, and integration. Data for ^13 ^C NMR spectra reported in terms of chemical shift. Chemical shifts are given in parts per million, relative to an internal standard such as residual solvent signals. Coupling constants are given in Hertz. Spectra were analysed with MestreNova. High resolution mass spectra were recorded via ESI-TOF-HRMS or MALDI-TOF-HRMS.

The purity of all final compounds was confirmed to exceed 95% by ^1^H NMR showing ^13 ^C-H satellite signals. Additionally, analytical HPLC purity analysis was carried out for compounds **34**, **113**, **143** and **149** with UV detection at 220 nm, using a PFP propyl column (RESTEK Allure HPLC column, particle size 5 *µ*m, pore size 60 Å, dimensions 150 × 4.6 mm) with a linear gradient of solvent B (acetonitrile, 0.1% TFA) over solvent A (H_2_O, 0.1% TFA) from 0 to 100% in 30 min at a flow rate of 1 mL/min. Typically, 25 *µ*L solution (0.5 mg/mL in 50% ACN/H_2_O) was injected for each compound.

### Procedures

#### Synthesis of iodo derivatives

4-Benzyl-2-iodophenol (**17a**)^99^: 4-Benzylphenol (**14a**) (1.1 g, 6.0 mmol) was dissolved in DMF (10 mL), diluted with concentrated aqueous ammonia (60 mL), and treated with a solution of potassium iodide (5.3 g, 32 mmol) and iodine (1.6 g, 6.3 mmol) in water (20 mL) all at once. After stirring at rt for 1 h, the ammonia was removed under reduced pressure. The remaining solution was neutralised with 1 N HCl in H_2_O. After extraction with EtOAc (50 mL), the organic phases were collected, dried over Na_2_SO_4_, filtered, and concentrated under reduced pressure. The residue was purified by FC (silica gel, EtOAc/hexane) giving **17a** (930 mg, 50% yield) as white solid. ^1^H NMR (400 MHz, MeOD) *δ* 7.50 (d, *J* = 2.1 Hz, 1H), 7.35 − 7.23 (m, 2H), 7.18 (td, *J* = 6.5, 1.7 Hz, 3H), 7.01 (dd, *J* = 8.3, 2.2 Hz, 1H), 6.76 (d, *J* = 8.2 Hz, 1H), 3.85 (s, 2H).

1-(Benzyloxy)-2-iodobenzene (**17ba**)^100^: To a solution of 2-iodophenol (**14b**) (440 mg, 2.0 mmol) in DMF (6 mL), anh. K_2_CO_3_ (1.38 g, 10.0 mmol) was added, and the mixture was stirred for 5 min before dropwise addition of benzyl bromide (**15a**) (374 mg, 2.2 mmol). The mixture was stirred at 80 °C for 24 h. After cooling to rt, CH_2_Cl_2_ (10 mL) and water (10 mL) were added and the mixture was shaken vigorously before extraction with CH_2_Cl_2_ (2 × 10 mL). The organic layers were collected, washed with brine (2 × 10 mL), dried over Na_2_SO_4_, filtered, and concentrated under reduced pressure. The crude residue of **17ba** (527 mg, 85% yield) was used directly in the next step. ^1^H NMR (400 MHz, CDCl_3_) *δ* 5.12 (s, 2H), 6.75 (dt, *J* = 7.6, 1.3 Hz,1H, H-5), 6.87 (dd, *J* = 8.2, 1.1 Hz, 1H, H-6), 7.29 (ddd, *J* = 8.2, 7.4,1.5 Hz, 1H, H-4), 7.33–7.46 (m, 3H), 7.55 (d, *J* = 7.3 Hz, 2H), 7.85 (dd, *J* = 7.8, 1.6 Hz).

1-Iodo-2-phenethoxybenzene (**17bb**) (CAS [1104274–16-9]): Compound **17bb** was prepared the same way as **17ba** from 2-iodophenol (**14b**) (440 mg, 2.0 mmol) and (2-bromoethyl)benzene (**15b**) (405 mg, 2.2 mmol) in DMF (6 mL), giving **17bb** (583 mg, 90% yield) as colourless oil. ^1^H NMR (400 MHz, CDCl_3_) *δ* 7.77 (dd, *J* = 7.8, 1.6 Hz, 1H), 7.42 − 7.30 (m, 4H), 7.34 − 7.21 (m, 2H), 6.78 (dd, *J* = 8.3, 1.3 Hz, 1H), 6.70 (td, *J* = 7.5, 1.4 Hz, 1H), 4.21 (t, *J* = 6.9 Hz, 2H), 3.18 (t, *J* = 6.9 Hz, 2H).

2-Iodo-*N*-phenethylaniline (**17cb**)^101^: Compound **17cb** was prepared the same way as **17ba** from 2-iodoaniline (**14c**) (440 mg, 2.0 mmol) and (2-bromoethyl)benzene (**15b**) (405 mg, 2.2 mmol) in DMF (6 mL), giving **17cb** (323 mg, 50% yield) as colourless oil. ^1^H NMR (400 MHz, CDCl_3_) *δ* 7.65 (dt, *J* = 7.9, 1.5 Hz, 2H), 7.39 − 7.18 (m, 1H), 7.18 − 7.10 (m, 2H), 6.76 (dd, *J* = 8.0, 1.5 Hz, 2H), 6.53 − 6.41 (m, 2H), 4.45 − 4.30 (m, 2H), 2.99 (q, *J* = 6.8 Hz, 2H).

1-Chloro-3-iodo-2-phenethoxybenzene (**17db**): Compound **17db** was prepared the same way as **17ba** from 2-chloro-6-iodophenol (**14d**) (508 mg, 2.0 mmol) and (2-bromoethyl)benzene (**15b**) (405 mg, 2.2 mmol) in DMF (6 mL), giving **17db** (680 mg, 95% yield) as colourless oil. ^1^H NMR (400 MHz, CDCl_3_) *δ* 7.68 (dd, *J* = 7.9, 1.5 Hz, 1H), 7.40 − 7.29 (m, 4H), 7.27 (s, 1H), 7.24 (ddt, *J* = 8.5, 5.3, 2.5 Hz, 1H), 6.78 (t, *J* = 7.9 Hz, 1H), 4.21 (t, *J* = 7.4 Hz, 2H), 3.25 (t, *J* = 7.4 Hz, 2H).

2–(2-Iodophenoxy)-1-phenylethan-1-ol (**17bc**)^102^: 2-Phenyloxirane (**16**) (580 mg, 4.8 mmol, 1.2 eq), 2-iodophenol (**14b**) (880 mg, 4.0 mmol, 1.0 eq), and Cs_2_CO_3_ (31.9 g, 12 mmol, 3.0 eq) were added to a 100-mL 2-neck round-bottom flask equipped with a condenser and a septum. DMF (8 mL) was added, and the mixture was heated under reflux (110 °C) for 24 h. After cooling to rt, water (10 mL) was added, followed by extraction with EtOAc (3 × 15 mL). The organic layers were collected, dried over anh. Na_2_SO_4_, filtered, and concentrated under reduced pressure. The residue was purified by FC (silica gel, EtOAc/hexane) to give **17bc** (0.98 g, 60% yield) as colourless oil. ^1^H NMR (400 MHz, CDCl_3_) *δ* 7.80 (dd, *J* = 7.8, 1.6 Hz, 1H), 7.54 − 7.47 (m, 2H), 7.46 7.25 (m, 3H), 6.84 − 6.72 (m, 2H), 5.21 (dt, *J* = 8.7, 2.9 Hz, 1H), 4.21 (dd, *J* = 9.3, 3.3 Hz, 1H), 4.02 (t, *J* = 9.0 Hz, 1H), 3.14 (d, *J* = 2.6 Hz, 1H).

2-Iodo-1-methoxy-4-phenethylbenzene (**17g**): To a stirred solution of methyl(triphenyl)phosphoniumbromide (1.23 g, 6.7 mmol) in freshly distilled THF (9 mL), sodiumbis(trimethylsilyl)amide (2.19 g, 6.1 mmol) was added under cooling (ice bath), causing the solution to turn yellow. After 1.5 h of stirring at rt, 3-iodo-4-methoxybenzaldehyde (**17e**) (786 mg, 3.0 mmol) was added to the ylide solution and stirring was continued for 4 h. The mixture was acidified using H_2_SO_4_ (0.1 M, 5 mL) and extracted with CH_2_Cl_2_ (50 mL). The combined organic phases were dried over Na_2_SO_4_, filtered, and concentrated under reduced pressure to give 1.84 g (90% yield) of a mixture of (E/Z)-2-iodo-1-methoxy-4-styrylbenzene (**17f**) as a oily solid. ^1^H NMR (400 MHz, MeOD) *δ* 7.99 (d, *J* = 2.2 Hz, 1H), 7.62 (d, *J* = 2.2 Hz, 1H), 7.55 (td, *J* = 8.3, 1.6 Hz, 3H), 7.35 (dd, *J* = 8.4, 6.9 Hz, 2H), 7.30 − 7.17 (m, 7H), 7.06 (d, *J* = 1.2 Hz, 2H), 6.96 (d, *J* = 8.5 Hz, 1H), 6.80 (d, *J* = 8.5 Hz, 1H), 6.58 (d, *J* = 12.1 Hz, 1H), 6.49 (d, *J* = 12.1 Hz, 1H), 3.90 (s, 3H), 3.84 (s, 3H). The crude olefin mixture (710 mg, 2.1 mmol) was dissolved in ethanol (12 mL) together with FeCl_3_ ·6H_2_O (29 mg, 0.11 mmol), immediately followed by the addition of aqueous hydrazine hydrate (1.0 mL, 21.0 mmol). The reaction mixture was stirred at rt for 24 h under air before extraction with CH_2_Cl_2_ (3 × 5 mL). The organic phased were collected, dried (Na_2_SO_4_), filtered, and concentrated under reduced pressure. The residue was purified by FC (SiO_2_, 10% EtOAc/petroleum ether), affording **17g** (640 mg, 90% yield) as oily solid. ^1^H NMR (400 MHz, MeOD) *δ* 7.55 (d, *J* = 2.1 Hz, 1H), 7.29 − 7.03 (m, 6H), 6.82 (d, *J* = 8.4 Hz, 1H), 3.82 (s, 3H), 2.93 − 2.82 (m, 2H), 2.85 − 2.77 (m, 2H).

#### General procedure (I) for the preparation of arylethynes

To a stirred solution of commercially available or synthetically prepared iodo derivatives (**17**) (Scheme 1 and 2) (1 eq) mixed with Et_3_N (4 eq) in dioxane (4 mL), trimethylsilylacetylene (1.3 eq), PdCl_2_(PPh_3_)_2_ (0.01 eq), and CuI (0.02 eq) were added. The reaction mixture was stirred at 45 °C for 5 h under nitrogen atmosphere. After cooling to rt, the reaction mixture was diluted with Et_2_O (5 mL) and washed with brine (5 mL). The organic layer was dried (Na_2_SO_4_), filtered, and concentrated under reduced pressure. KF (3.6 eq) was added to the residue and the mixture was dissolved in MeOH (5 mL) and stirred for 3 h at rt before concentration under reduced pressure. After addition of CH_2_Cl_2_ (5 mL) and water (3 mL), the organic layer was collected, dried over MgSO_4_, and filtered through a short silica plug to afford the corresponding arylethynes **18** (Scheme 1). Overall yields 65–93%.

#### Synthesis of arylethynes

1-Butyl-3-ethynylbenzene (**18a**, CAS [2243191–48-0]): Synthesised from 1-butyl-3-iodobenzene (520 mg, 2 mmol) according to the general procedure (**I**) to afford the title compound as a yellowish oil (269 mg) in 85% yield, ^1^H NMR (400 MHz, CDCl_3_) *δ* 7.41 − 7.29 (m, 2H), 7.25 (td, *J* = 7.4, 0.8 Hz, 1H), 7.19 (dt, *J* = 7.7, 1.6 Hz, 1H), 3.07 (s, 1H), 2.66 − 2.57 (m, 2H), 1.68 − 1.56 (m, 2H), 1.37 (h, *J* = 7.4 Hz, 2H), 0.95 (t, *J* = 7.3 Hz, 3H).

1-(Benzyloxy)-2-ethynylbenzene (**18b**)^103^: Synthesised from 1-(benzyloxy)-2-iodobenzene (620 mg, 2 mmol) according to the general procedure (**I**) to afford the title compound as an oil (291 mg) in 70% yield. ^1^H NMR (400 MHz, CDCl_3_) *δ* 7.59 7.47 (m, 3H), 7.41 − 7.15 (m, 5H), 6.99 − 6.86 (m, 1H), 5.23 (s, 2H).

1-Ethynyl-2-phenethoxybenzene (**18c**): Synthesised from 1-iodo-2-phenethoxybenzene (648 mg, 2 mmol) according to the general procedure (**I**) to afford the title compound as an oil (333 mg) in 75% yield. ^1^H NMR (400 MHz, CDCl_3_) *δ* 7.46 (dd, *J* = 7.6, 1.7 Hz, 1H), 7.39 − 7.31 (m, 4H), 7.31 − 7.23 (m, 1H), 6.95 − 6.83 (m, 3H), 4.24 (t, *J* = 7.1 Hz, 2H), 3.30 (s, 1H), 3.17 (t, *J* = 7.1 Hz, 2H).

2–(2-Ethynylphenoxy)-1-phenylethan-1-ol (**18d**): Synthesised from 2–(2-iodophenoxy)-1-phenylethan-1-ol (680 mg, 2 mmol) according to the general procedure (**I**) to afford the title compound as an oil (309 mg) with 65% yield. ^1^H NMR (400 MHz, CDCl_3_) *δ* 7.53 − 7.26 (m, 7H), 6.97 (td, *J* = 7.5, 1.0 Hz, 1H), 6.88 (dd, *J* = 8.4, 1.0 Hz, 1H), 5.18 (dt, *J* = 9.1, 2.8 Hz, 1H), 4.24 (dd, *J* = 9.5, 3.1 Hz, 2H), 4.02 (t, *J* = 9.2 Hz, 2H), 3.34 (s, 1H).

2-Ethynyl-*N*-phenethylaniline (**18e**): Synthesised from 2-iodo-*N*-phenethylaniline (646 mg, 2 mmol) according to the general procedure (**I**) to afford the title compound as an oil (331 mg) in 75% yield. ^1^H NMR (400 MHz, CDCl_3_) *δ* 7.40 − 7.18 (m, 7H), 6.69 − 6.59 (m, 2H), 4.72 (s, 1H), 3.46 (td, *J* = 7.1, 5.6 Hz, 2H), 3.34 (s, 1H), 2.96 (t, *J* = 7.2 Hz, 2H).

2-Ethynyl-4-fluorophenol (**18f**)^104^: Synthesised from 4-fluoro-2-iodophenol (476 mg, 2 mmol) according to the general procedure (**I**) to afford the title compound as an oil (231 mg) in 85% yield. ^1^H NMR (400 MHz, CDCl_3_) *δ* 7.13 − 6.97 (m, 2H), 6.91 (dd, *J* = 9.0, 4.6 Hz, 1H), 5.65 (s, 1H), 3.51 (s, 1H).

2-Ethynyl-4-methylphenol (**18g**)^104^: Synthesised from 2-iodo-4-methylphenol (468 mg, 2 mmol) according to the general procedure (**I**) to afford the title compound as an oil (211 mg) in 80% yield. ^1^H NMR (400 MHz, CDCl_3_) *δ* 7.20 (d, *J* = 2.2 Hz, 1H), 7.10 (dd, *J* = 8.4, 2.2 Hz, 1H), 6.87 (d, *J* = 8.4 Hz, 1H), 5.65 (s, 1H), 3.46 (s, 1H), 2.27 (s, 3H).

4-Ethynyl-2-methylphenol (**18h**): Synthesised from 4-ethyl-2-iodophenol (496 mg, 2 mmol) according to the general procedure (**I**) to afford the title compound as an oil (238 mg) in 90% yield. ^1^H NMR (400 MHz, CDCl_3_) *δ* 7.35 − 7.20 (m, 1H), 7.13 (dd, *J* = 8.6, 2.2 Hz, 1H), 6.89 (d, *J* = 8.4 Hz, 1H), 5.66 (s, 1H), 3.46 (s, 1H), 2.58 (q, *J* = 7.7 Hz, 2H), 1.30 − 1.15 (t, *J* = 7.7 Hz, 3H).

2-Ethynyl-4-propylphenol (**18i**)^105^: Synthesised from 2-iodo-4-propylphenol (524 mg, 2 mmol) according to the general procedure (**I**) to afford the title compound as an oil (202 mg) in 88% yield. ^1^H NMR (400 MHz, CDCl_3_) *δ* 7.35 − 7.17 (m, 1H), 7.10 (dd, *J* = 8.4, 2.4 Hz, 1H), 6.88 (d, *J* = 8.4 Hz, 1H), 5.66 (s, 1H), 3.46 (s, 1H), 2.50 (t, *J* = 7.5 Hz, 2H), 1.75 − 1.54 (m, 2H), 0.94 (t, *J* = 7.5 Hz, 3H).

4-Benzyl-2-ethynylphenol (**18j**): Synthesised from 4-benzyl-2-iodophenol (620 mg, 2 mmol) according to the general procedure (**I**) to afford the title compound as an oil (307 mg) in 93% yield. ^1^H NMR (400 MHz, MeOD) *δ* 7.27 (dd, *J =* 8.7, 6.5 Hz, 2H), 7.21 − 7.12 (m, 4H), 7.03 (dd, *J* = 8.4, 2.3 Hz, 1H), 6.77 (d, *J* = 8.4 Hz, 1H), 4.63 (s, 1H), 3.85 (s, 2H), 3.57 (s, 1H).

2-Ethynyl-1-methoxy-4-phenethylbenzene (**18k**): Synthesised from 2-iodo-1methoxy-4-phenethylbenzene (676 mg, 2 mmol) according to the general procedure (**I**) to afford the title compound as an oil (378 mg) in 80% yield. ^1^H NMR (400 MHz, MeOD) *δ* 7.29 − 7.03 (m, 7H), 6.88 (d, *J* = 8.5 Hz, 1H), 3.83 (s, 3H), 3.55 (s, 1H), 3.33 (p, *J* = 1.6 Hz, 2H), 2.93 − 2.78 (m, 2H).

1-Chloro-2-ethynyl-3-phenethoxybenzene (**18 l**): Synthesised from 1-chloro-2-iodo-3-phenethoxybenzene (676 mg, 2 mmol) according to the general procedure (**I**) to afford the title compound as an oil (476 mg) in 83% yield. ^1^H NMR (400 MHz, CDCl_3_) *δ* 7.42 − 7.30 (m, 6H), 7.33 − 7.20 (m, 1H), 7.00 (t, *J* = 7.9 Hz, 1H), 4.39–4.31 (m, 2H), 3.26–3.16 (m, 3H).

1,2,4-Trichloro-5-ethynylbenzene (**18m**, CAS [6546–87-8]): Synthesised from 1,2,4-trichloro-5-iodobenzene (612 mg, 2 mmol) according to the general procedure (**I**) to afford the title compound as an oil (310 mg) in 76% yield. ^1^H NMR (400 MHz, CDCl_3_) *δ* 7.63 (s, 1H), 7.55 (s, 1H), 7.29 (s, 1H), 3.46 (s, 1H).

1,3,5-Trichloro-2-ethynylbenzene (**18n**)^106^: Synthesised from 1,3,5-trichloro-5-iodobenzene (612 mg, 2 mmol) according to the general procedure (**I**) to afford the title compound as an oil (326 mg) in 76% yield. ^1^H NMR (400 MHz, CDCl_3_) *δ* 7.40 (s, 2H), 3.73 (s, 1H).

#### General procedure (II) for the preparation of 4-Aryl-1,2,3-Triazoles:

To a stirred solution of commercially available or synthetically prepared arylethynes (**18**) (1 equiv) and CuI (0.05 equiv) in DMF/MeOH solution (2 mL, 9:1) under an argon atmosphere, TMSN_3_ (1.5 equiv) was added. The resulting solution was stirred at 100 °C for 10–12 h. After consumption of the ethynyl substrate, the mixture was cooled to rt, the precipitate was filtered off, and the remaining solution was concentrated under reduced pressure. The crude residue was purified by FC (SiO_2_, EtOAc/petroleum ether) to obtain the desired 4-aryl-1,2,3-triazole.

#### General procedure (III) for the demethylation of aryl methyl ethers:

The methyl ether (1 eq) was dissolved in 48% HBr in water (4 mL), and the orange solution was heated to 100 °C for 14 h under nitrogen atmosphere. The mixture was cooled to rt, diluted with water, and neutralised by the addition of a saturated aq. soln. of NaHCO_3_ until the evolution of CO_2_ ceased. The organic layer was extracted with EtOAc (2 × 5 mL), dried over Na_2_SO_4_, filtered, and concentrated under reduced pressure to afford a residue that was purified by FC (SiO_2_, EtOAc/petroleum ether) to give the desired phenol.

#### Synthesis of 4-Aryl-1,2,3-Triazoles

4–(1,2,3-Triazol-4-yl)phenol (**20**)^107^: Synthesised from 1-ethynyl-4-methoxybenzene (264 mg, 2.0 mmol) and TMSN_3_ (345 mg, 3.0 mmol) using the general procedure (**II**) to give 4–(4-methoxyphenyl)-1,2,3-triazole (245 mg, 70%) as a white solid. ^1^H NMR (400 MHz, CDCl_3_) *δ* 7.92 (s, 1H), 7.76 − 7.68 (m, 2H), 7.02 − 6.93 (m, 2H), 3.85 (s, 3H). Using the general procedure (**III**), 4–(4-methoxyphenyl)-1,2,3-triazole (166 mg, 1.0 mmol) dissolved in 48% HBr in water (4 mL) gave **20** (115 mg, 75%) as a white solid. ^1^H NMR (400 MHz, MeOD) *δ* 8.00 (s, 1H), 7.72 − 7.59 (m, 2H), 6.95 − 6.82 (m, 2H), ^13 ^C NMR (101 MHz, MeOD) *δ* 157.82, 127.06, 115.40, HRMS (ESI/QTOF) *m/z*: calcd for [M + H]^+^ C_8_H_8_N_3_O^+^ 162.0662; found, 162.0661.

4–(3-Isopropylphenyl)-1,2,3-triazole (**23**, CAS [2192187–16-7]): Synthesised from 1-ethynyl-3-isopropylbenzene (144 mg, 1.0 mmol) and TMSN_3_ (173 mg, 1.5 mmol) using the general procedure (**II**) to give **23** (84 mg, 45%) as a white solid. ^1^H NMR (400 MHz, CDCl_3_) *δ* 7.98 (s, 1H), 7.71 (t, *J* = 1.8 Hz, 1H), 7.63 (dt, *J* = 7.7, 1.5 Hz, 1H), 7.39 (t, *J* = 7.7 Hz, 1H), 7.31 − 7.23 (m, 1H), 2.99 (p, *J* = 6.9 Hz, 1H), 1.31 (d, *J* = 6.9 Hz, 6H), ^13 ^C NMR (101 MHz, CDCl_3_) *δ* 149.77, 129.65, 128.99, 127.00, 124.30, 123.71, 34.18, 23.95, HRMS (ESI/QTOF) *m/z*: calcd for [M + H]^+^ C_11_H_13_N_3_^+^ 188.1188, found: 188.1185.

4–(3-Butylphenyl)-1,2,3-triazole (**24**, CAS [2192187–22-5]): Synthesised from 1-butyl-3-ethynylbenzene (**18a**) (150 mg, 0.95 mmol) and TMSN_3_ (164 mg, 1.43 mmol) using the general procedure (**II**) to give **24** (153 mg, 80%) as a white solid. ^1^H NMR (400 MHz, MeOD) *δ* 8.15 (s, 1H), 7.68 (d, *J* = 1.9 Hz, 1H), 7.64 (dt, *J* = 7.8, 1.5 Hz, 1H), 7.36 (t, *J* = 7.7 Hz, 1H), 7.21 (dt, *J* = 7.6, 1.5 Hz, 1H), 2.74 − 2.66 (m, 2H), 1.67 (tt, *J* = 9.2, 6.9 Hz, 2H), 1.41 (h, *J* = 7.4 Hz, 2H), 0.98 (t, *J* = 7.4 Hz, 3H), ^13 ^C NMR (101 MHz, MeOD) *δ* 143.49, 128.57, 128.34, 125.61, 123.03, 35.23, 33.44, 29.42, 22.01, 13.00, HRMS (ESI/QTOF) *m/z*: calcd for [M + H]^+^ C_12_H_16_N_3_^+^ 202.1339; found, 202.1342.

4–(2-Benzyloxyphenyl)-1,2,3-triazole (**25**)^108^: Synthesised from 1-(benzyloxy)-2-ethynylbenzene (**18b**) (104 mg, 0.5 mmol) and TMSN_3_ (87 mg, 0.75 mmol) using the general procedure (**II**) to give **25** (90 mg, 72%) as a white solid. ^1^H NMR (400 MHz, MeOD) *δ* 8.08 (s, 1H), 7.51 − 7.42 (m, 2H), 7.43 − 7.28 (m, 5H), 7.18 (dd, *J* = 8.4, 1.1 Hz, 1H), 7.06 (td, *J* = 7.5, 1.1 Hz, 1H), 5.23 (s, 2H), ^13 ^C NMR (101 MHz, CDCl_3_) *δ* 155.20, 135.99, 130.49, 129.97, 128.98, 128.61, 128.22, 127.86, 121.64, 112.64, 70.91, HRMS (ESI/QTOF) *m/z*: calcd for [M + H]^+^ C_15_H_14_N_3_O^+^ 252.1131; found, 252.1131.

4–(2-Phenethoxyphenyl)-1,2,3-triazole (**26**): Synthesised from 1-ethynyl-2-phenethoxybenzene (**18c**) (111 mg, 0.5 mmol) and TMSN_3_ (87 mg, 0.75 mmol) using the general procedure (**II**) to give **26** (93 mg, 70%) as a white solid, m.p. 91–93 °C. ^1^H NMR (400 MHz, CDCl_3_) *δ* 12.03 (s, 1H), 7.98 (s, 1H), 7.81 (s, 1H), 7.46 − 7.28 (m, 6H), 7.12 − 7.02 (m, 2H), 4.44 (t, *J* = 6.6 Hz, 2H), 3.25 (t, *J* = 6.6 Hz, 2H), ^13 ^C NMR (101 MHz, CDCl_3_) *δ* 155.12, 137.75, 129.92, 129.01, 128.74, 128.28, 127.04, 121.41, 112.15, 69.00, 35.61, HRMS (ESI/QTOF) *m/z*: calcd for [M + H]^+^ C_16_H_16_N_3_O^+^ 266.1288; found, 266.1290.

2–(2-(1*H*-1,2,3-triazol-5-yl)phenoxy)-1-phenylethan-1-ol (**27**): Synthesised from 2(2-ethynylphenoxy)-1-phenylethan-1-ol (**18d**) (120 mg, 0.5 mmol) and TMSN_3_ (87 mg, 0.75 mmol) using the general procedure (**II**) to give **27** (91 mg, 65%) as a white solid, m.p. 153–155 °C. ^1^H NMR (400 MHz, CDCl_3_) *δ* 8.01 (s, 1H), 7.69 (dd, *J* = 7.7, 1.6 Hz, 1H), 7.50 − 7.42 (m, 2H), 7.42 − 7.28 (m, 4H), 7.07 (td, *J* = 7.6, 1.1 Hz, 1H), 6.99 (dd, *J* = 8.3, 1.1 Hz, 1H), 5.20 (dd, *J* = 9.0, 3.1 Hz, 1H), 4.38 (dd, *J* = 9.5, 3.2 Hz, 1H), 4.08 (t, *J* = 9.3 Hz, 1H), ^13 ^C NMR (101 MHz, MeOD) *δ* 156.02, 142.09, 130.21, 128.94, 128.37, 126.99, 121.79, 113.38, 73.96, 72.68, HRMS (ESI/QTOF) *m/z*: calcd for [M + Na]^+^ C_16_H_15_N_3_O_2_Na^+^ 304.1056; found, 304.1064.

*N*-Phenyl-2-(1*H*-1,2,3-triazol-5-yl)aniline (**28**): Synthesised from 2-ethynyl-*N-*phenethylaniline (**18e**) (165 mg, 0.75 mmol) and TMSN_3_ (130 mg, 1.13 mmol) using the general procedure (**II**) to give **28** (80 mg, 45%) as a white solid, m.p. 129–132 °C. ^1^H NMR (400 MHz, MeOD) *δ* 8.03 (s, 1H), 7.60 (d, *J* = 7.6 Hz, 1H), 7.31 − 7.13 (m, 6H), 6.83 (dd, *J* = 8.4, 1.1 Hz, 1H), 6.70 (td, *J* = 7.5, 1.1 Hz, 1H), 3.49 (t, *J* = 7.0 Hz, 3H), 2.96 (t, *J* = 7.0 Hz, 3H), ^13 ^C NMR (101 MHz, MeOD) *δ* 145.50, 139.57, 130.69, 129.11, 128.48, 128.03, 127.78, 125.85, 115.73, 111.04, 44.81, 35.03, HRMS (ESI/QTOF) *m/z*: calcd for [M + H]^+^ C_16_H_17_N_4_^+^ 265.1448; found, 265.1441.

4-Fluoro-2-(1*H*-1,2,3-triazol-5-yl)phenol (**34**): Synthesised from 2-ethynyl-4-fluorophenol (**18f**) (204 mg, 1.5 mmol) and TMSN_3_ (259 mg, 2.25 mmol) using the general procedure (**II**) to give **34** (201 mg, 75%) as a white solid, m.p. 186–188 °C. ^1^H NMR (400 MHz, MeOD) *δ* 8.31 (s, 1H), 7.62 (dd, *J* = 9.6, 2.8 Hz, 1H), 7.04 − 6.86 (m, 2H), ^13 ^C NMR (101 MHz, MeOD) *δ* 157.53, 155.20, 150.77, 116.82, 116.73, 115.37, 115.14, 112.71, 112.46, HRMS (ESI/QTOF) *m/z*: calcd for [M + H]^+^ C_8_H_7_FN_3_O^+^ 180.0568; found, 180.0567.

4-Methyl-2-(1*H*-1,2,3-triazol-5-yl)phenol (**35**): Synthesised from 2-ethynyl-4-methylphenol (**18g**) (170 mg, 1.3 mmol) and TMSN_3_ (225 mg, 1.95 mmol) using the general procedure (**II**) to give **35** (171 mg, 75%) as a white solid, m.p. 160–162 °C. ^1^H NMR (400 MHz, MeOD) *δ* 8.24 (s, 1H), 7.69 − 7.58 (m, 1H), 7.04 (dd, *J* = 8.3, 2.3 Hz, 1H), 6.85 (d, *J* = 8.3 Hz, 1H), 2.32 (s, 3H), ^13 ^C NMR (101 MHz, MeOD) *δ* 152.36, 129.91, 128.66, 127.09, 115.80, 114.63, 19.18, HRMS (ESI/QTOF) *m/z*: calcd for [M + H]^+^ C_9_H_10_N_3_O^+^ 176.0818; found, 176.0817.

4-Ethyl-2-(1*H*-1,2,3-triazol-5-yl)phenol (**36**): Synthesised from 4-ethyl-2-methylphenol (**18h**) (146 mg, 1.0 mmol) and TMSN_3_ (173 mg, 1.5 mmol) using the general procedure (**II**) to give 36 (151 mg, 80%) as a white solid, m.p. 71–73 °C. ^1^H NMR (400 MHz, MeOD) δ 8.24 (s, 1H), 7.66 (s, 1H), 7.07 (dd, *J* = 8.3, 2.2 Hz, 1H), 6.88 (d, *J* = 8.5 Hz, 1H), 2.63 (q, *J* = 7.6 Hz, 2H), 1.26 (td, *J* = 7.7, 1.2 Hz, 3H), ^13 ^C NMR (101 MHz, MeOD) *δ* 152.56, 135.37, 128.73, 126.00, 115.83, 114.74, 114.68, 27.63, 15.08, HRMS (ESI/QTOF) *m/z*: calcd for [M + H]^+^ C_10_H_12_N_3_O^+^ 190.0975; found, 190.0977.

4-Propyl-2-(1H-1,2,3-triazol-5-yl)phenol (**37**): Synthesised from 2-ethynyl-4-propylphenol (**18i**) (160 mg, 1.0 mmol) and TMSN_3_ (173 mg, 1.5 mmol) using the general procedure (**II**) to give **37** (152 mg, 75%) as a white solid, m.p. 138–140 °C. ^1^H NMR (400 MHz, MeOD) δ 8.24 (s, 1H), 7.64 (s, 1H), 7.05 (dd, *J* = 8.3, 2.2 Hz, 1H), 6.88 (d, *J* = 8.2 Hz, 1H), 2.65 − 2.50 (m, 2H), 1.67 (h, *J* = 7.4 Hz, 2H), 0.97 (t, *J* = 7.3 Hz, 3H), ^13 ^C NMR (101 MHz, MeOD) *δ* 152.60, 133.70, 129.38, 126.63, 115.78, 114.68, 36.80, 24.52, 12.72, HRMS (ESI/QTOF) *m/z*: calcd for [M + H]^+^ C_11_H_14_N_3_O^+^ 204.1131; found, 204.1130.

4-Benzyl-2-(1H-1,2,3-triazol-5-yl)phenol (**38**): Synthesised from 4-benzyl-2-ethynylphenol (**18j**) (460 mg, 2.2 mmol) and TMSN_3_ (379 mg, 3.3 mmol) using the general procedure (**II**) to give **38** (441 mg, 80%) as a white solid, m.p. 148–151 °C. ^1^H NMR (400 MHz, MeOD) *δ* 8.23 (s, 1H), 7.75 − 7.65 (m, 1H), 7.32 − 7.13 (m, 5H), 7.05 (dd, *J* = 8.4, 2.3 Hz, 1H), 6.88 (d, *J* = 8.3 Hz, 1H), 3.95 (s, 2H), ^13 ^C NMR (101 MHz, MeOD) *δ* 152.92, 141.64, 132.63, 129.81, 128.40, 128.04, 127.20, 125.58, 115.97, 115.01, 40.57, HRMS (ESI/QTOF) *m/z*: calcd for [M + H]^+^ C_15_H_14_N_3_O^+^ 252.1131; found, 252.1137.

4-Phenylethyl-2-(1*H*-1,2,3-triazol-5-yl)phenol (**39**): Synthesised from 2-ethynyl-1-methoxy-4-phenethylbenzene (**18k**) (544 mg, 2.3 mmol) and TMSN_3_ (398 mg, 3.45 mmol) using the general procedure (**II**) to give 5–(2-methoxy-5-phenethylphenyl)-1*H*-1,2,3-triazole (282 mg, 44%) as a white solid. ^1^H NMR (400 MHz, MeOD) *δ* 8.15 (s, 1H), 7.79 (s, 1H), 7.30 − 7.22 (m, 2H), 7.22 − 7.11 (m, 4H), 7.02 (d, *J* = 8.5 Hz, 1H), 3.95 (s, 3H), 2.96–2-90 (m, 4H). Using the general procedure (**III**), 5–(2-methoxy-5phenethylphenyl)-1*H*-1,2,3-triazole (246 mg, 1.2 mmol) dissolved in 48% HBr in water (5 mL) gave **39** (238 mg, 75%) as a white solid, m.p. 157–159 °C. ^1^H NMR (400 MHz, MeOD) *δ* 8.21 (brs, 1H), 7.64 (brs, 1H), 7.32 − 7.12 (m, 5H), 7.03 (dd, *J* = 8.3, 2.3 Hz, 1H), 6.85 (d, *J* = 8.3 Hz, 1H), 2.98 − 2.81 (m, 4H), ^13 ^C NMR (101 MHz, MeOD) *δ* 152.76, 141.71, 132.93, 129.45, 128.21, 127.87, 126.78, 125.45, 115.73, 37.93, 36.90, HRMS (ESI/QTOF) *m/z*: calcd for [M + H]^+^ C_16_H_16_N_3_O^+^ 266.1288; found, 266.1293.

4–(3-Chloro-2-phenethoxyphenyl)-1,2,3-triazole (**43**): Synthesised from 1-chloro-2-ethynyl-3-phenethoxybenzene (**18 l**) (150 mg, 0.59 mmol) and TMSN_3_ (102 mg, 0.89 mmol) using the general procedure (**II**) to give **43** (120 mg, 68%) as a white solid, m.p. 96–99 °C. ^1^H NMR (400 MHz, CDCl_3_) *δ* 7.86 − 7.71 (m, 1H), 7.46 − 7.24 (m, 6H), 7.16 (t, *J* = 7.9 Hz, 2H), 4.10 (t, *J* = 6.8 Hz, 2H), 3.13 (t, *J* = 6.8 Hz, 2H), ^13 ^C NMR (101 MHz, MeOD) *δ* 151.89, 138.17, 129.96, 128.90, 128.53, 128.13, 126.77, 126.31, 125.06, 73.32, 36.16, HRMS (ESI/QTOF) *m/z*: calcd for [M + Na]^+^ C_16_H_14_ClN_3_ONa^+^ 322.0718; found, 322.0722.

2-(1*H*-1,2,3-triazol-5-yl)benzene-1,3-diol (**44**): Synthesised from **45** (130 mg, 0.75 mmol) dissolved in 48% HBr in water (3 mL) using the general procedure (**III**) to give **44** (93 mg, 70%) as white solid, m.p. 257–259 °C. ^1^H NMR (400 MHz, MeOD) *δ* 8.38 (s, 1H), 7.02 (t, *J* = 8.2 Hz, 1H), 6.46 (d, *J* = 8.1 Hz, 2H), ^13 ^C NMR (101 MHz, MeOD) *δ* 156.24, 129.03, 106.57, 102.41, HRMS (ESI/QTOF) *m/z*: calcd for [M + H]^+^ C_8_H_7_N_3_O_2_^+^ 178.0616; found, 178.0606.

4–(2,6-Dimethoxyphenyl)-1,2,3-triazole (**45**, CAS [2385145–91-3]): Synthesised from 2-ethynyl-1,3-dimethoxybenzene (324 mg, 2.0 mmol) and TMSN_3_ (345 mg, 3.0 mmol) using the general procedure (**II**) to give **45** (197 mg, 48%) as a white solid. ^1^H NMR (400 MHz, CDCl_3_) *δ* 8.34 (s, 1H), 7.35 (t, *J* = 8.4 Hz, 1H), 6.74 (d, *J* = 8.5 Hz, 2H), 4.02 (s, 9H), ^13 ^C NMR (101 MHz, MeOD) *δ* 157.60, 132.66, 132.57, 130.45, 103.87, 55.08, HRMS (ESI/QTOF) *m/z*: calcd for [M + H]^+^ C_10_H_11_N_3_O_2_^+^ 206.0930; found, 206.0940.

4–(2,4-Dichlorophenyl)-1,2,3-triazole (**48**)^107^: Synthesised from 2,4-dichloro-1-ethynylbenzene (250 mg, 1.5 mmol) and TMSN_3_ (259 mg, 2.25 mmol) using the general procedure (**II**) to give **48** (272 mg, 85%) as a yellow solid. ^1^H NMR (400 MHz, CDCl_3_) *δ* 8.24 (d, *J* = 8.2 Hz, 1H), 7.93 (d, *J* = 8.4 Hz, 1H), 7.52 (t, *J* = 2.5 Hz, 1H), 7.36 (dt, *J* = 8.5, 2.5 Hz, 1H), ^13 ^C NMR (101 MHz, MeOD) *δ* 134.29, 132.40, 130.97, 129.62, 127.30, 56.06, HRMS (ESI/QTOF) *m/z*: calcd for [M + H]^+^ C_8_H_6_Cl_2_N_3_^+^ 213.9933; found, 213.9931.

4–(3,4-Dichlorophenyl)-1,2,3-triazole (**49**)^109^: Synthesised from 1,2-dichloro-4-ethynylbenzene (170 mg, 1.0 mmol) and TMSN_3_ (173 mg, 1.5 mmol) using the general procedure (**II**) to give **49** (187 mg, 88%) as a yellow solid. ^1^H NMR (400 MHz, MeOD) *δ* 8.25 (s, 1H), 8.06 (d, *J* = 2.0 Hz, 1H), 7.81 (dd, *J* = 8.4, 2.0 Hz, 1H), 7.62 (d, *J* = 8.4 Hz, 1H), ^13 ^C NMR (101 MHz, MeOD) *δ* 144.15, 132.55, 131.53, 130.64, 127.19, 125.05, 47.45, 47.24, 47.03, HRMS (ESI/QTOF) *m/z*: calcd for [M + H]^+^ C_8_H_6_Cl_2_N_3_^+^ 213.9933; found, 213.9928.

4–(3,5-Dichlorophenyl)-1,2,3-triazole (**50**, CAS [55751–17-2]): Synthesised from 1,3-dichloro-5-ethynylbenzene (170 mg, 1.0 mmol) and TMSN_3_ (173 mg, 1.5 mmol) using the general procedure (**II**) to give **50** (175 mg, 82%) as a yellow solid. ^1^H NMR (400 MHz, MeOD) *δ* 8.29 (s, 1H), 7.84 (d, *J* = 2.0 Hz, 2H), 7.42 (q, *J* = 1.6 Hz, 1H), ^13 ^C NMR (101 MHz, MeOD) *δ* 143.94, 135.32, 133.74, 127.46, 123.88, HRMS (ESI/QTOF) *m/z*: calcd for [M + H]^+^ C_8_H_6_Cl_2_N_3_^+^ 213.9933; found, 213.9932.

4–(2,6-Dichlorophenyl)-1,2,3-triazole (**51**, CAS [2385230–51-1]): Synthesised from 1,3-dichloro-2-ethynylbenzene (170 mg, 1.0 mmol) and TMSN_3_ (173 mg, 1.5 mmol) using the general procedure (**II**) to give **51** (160 mg, 75%) as a yellow solid. ^1^H NMR (400 MHz, MeOD) *δ* 7.95 (s, 1H), 7.58 − 7.49 (m, 2H), 7.45 (dd, *J* = 8.9, 7.2 Hz, 1H), ^13 ^C NMR (101 MHz, MeOD) *δ* 135.87, 130.82, 128.08, HRMS (ESI/QTOF) *m/z*: calcd for [M + H]^+^ C_8_H_6_Cl_2_N_3_^+^ 213.9933; found, 213.9927.

4–(2,4,5-Trichlorophenyl)-1,2,3-triazole (**52**): Synthesised from 1,2,4-trichloro-5-ethynylbenzene (**18m**) (340 mg, 2.0 mmol) and TMSN_3_ (345 mg, 3.0 mmol) using the general procedure (**II**) to give **52** (395 mg, 80%) as a yellow solid, m.p. 190–192 °C. ^1^H NMR (400 MHz, CDCl_3_) *δ* 8.20 (s, 1H), 8.09 (s, 1H), 7.56 (s, 1H), ^13 ^C NMR (101 MHz, DMSO) *δ* 140.90, 131.74, 131.59, 130.85, 130.49, 130.18, 129.72, HRMS (ESI/QTOF) *m/z*: calcd for [M + H]^+^ C_8_H_5_Cl_3_N_3_^+^ 247.9544; found, 247.9536.

4–(2,4,6-Trichlorophenyl)-1,2,3-triazole (**53**): Synthesised from 1,3,5-trichloro-2-ethynylbenzene (**18n**) (170 mg, 1.0 mmol) and TMSN_3_ (173 mg, 1.5 mmol) using the general procedure (**II**) to give **53** (198 mg, 80%) as a yellow solid, m.p. 236–238 °C. ^1^H NMR (400 MHz, MeOD) *δ* 7.97 (s, 1H), 7.66 (s, 2H), ^13 ^C NMR (101 MHz, DMSO) *δ* 136.31, 135.32, 128.67, HRMS (ESI/QTOF) *m/z*: calcd for [M + H]^+^ C_8_H_5_Cl_3_N_3_^+^ 247.9544; found, 247.9538.

#### Synthesis of 5-Substituted 4-Aryl-1,2,3-Triazoles

4-Phenyl-1*H*-1,2,3-triazole-5-carbonitrile (**62**)^65^: To a strongly stirred solution of NaN_3_ (195 mg, 1.5 mmol) suspended in DMF (4 mL) at 90 °C, a second solution of phenylpropynenitrile (**54**) (255 mg, 2.0 mmol) in DMF (2 mL) was added dropwise through an addition funnel over 10 min. The mixture was stirred for 1 h at 90 °C before cooling to rt and removing the solvent under reduced pressure. Water (6 mL) was added, and the aqueous layer was extracted with CH_2_Cl_2_ to remove the oil and to give a transparent light colour solution. Finally, the aqueous solution was acidified with 10% HCl resulting in a light yellow precipitate which was washed with iced water to yield the crude product. The residue was purified by FC (SiO_2_, CH_2_Cl_2_/MeOH) to obtain **62** (255 mg, 75% yield) as white solid. ^1^H NMR (400 MHz, MeOD) *δ* 7.93 − 7.77 (m, 2H), 7.52 − 7.39 (m, 3H). 5.38 (brs, 1H), ^13 ^C NMR (101 MHz, MeOD) *δ* 147.13, 130.16, 128.97, 126.41, 126.16, 116.65, 112.66, HRMS (ESI/QTOF) *m/z*: calcd for [M + H]^+^ C_9_H_6_N_4_^+^ 171.0671; found, 171.0667.

4–(3-Chlorophenyl)-5-methyl-1,2,3-triazole (**63**): 3-Chlorobenzaldehyde (280 mg, 2.0 mmol), nitromethane (180 mg, 2.4 mmol) and ammonium acetate (93 mg, 1.2 mmol) were added to 2 mL of glacial acetic acid. The resulting solution was heated under reflux for 2 h before pouring the reaction mixture into ice water. The yellow solid thus formed was collected by filtration to give the crude product 1-chloro-3–(2-nitroprop-1-en-1-yl)benzene (**56a**)^66^^,^^110^. The crude product (197 mg, 1.0 mmol) and NaN_3_ (98 mg, 1.5 mmol) were stirred in DMF (3 mL), and *p*-TsOH (87 mg, 0.5 mmol) was added to the mixture at rt. The mixture was stirred at 60 °C under air for 1 h. After completion of the reaction, the mixture was cooled to rt, quenched with H_2_O (5 mL) and extracted with EtOAc (3 × 10 mL). The organic layers were collected, dried over anh. Na_2_SO_4_, filtered, and concentrated under reduced pressure. The residue was purified by FC (silica gel, EtOAc/hexane) to afford **63** (135 mg, 70%) as white solid, m.p. 161–163 °C. ^1^H NMR (400 MHz, CDCl_3_) *δ* 11.42 (brs, 1H), 7.73 (t, *J* = 1.8 Hz, 1H), 7.62 (dt, *J* = 7.4, 1.6 Hz, 1H), 7.46 − 7.31 (m, 2H), 2.55 (s, 3H), ^13 ^C NMR (101 MHz, MeOD) *δ* 134.37, 132.79, 130.00, 127.53, 126.48, 124.94, 8.94, HRMS (ESI/QTOF) *m/z*: calcd for [M + H]^+^ C_9_H_8_ClN_3_^+^ 194.0485; found, 194.0482.

4,5-Bis(3-chlorophenyl)-1,2,3-triazole (**64**)^67^: To a stirred solution of 3-chlorobenzyaldehyde (420 mg, 3 mmol) in ethanol (6 mL) *p*-toluenesulfonyl hydrazide (**57**) (615 mg, 3.3 mmol) was added, and the solution was stirred for 2 h at rt. After completion of the reaction, the solvent was removed under reduced pressure. The obtained solid residue was washed with ethanol and dried under reduced pressure to afford N’-(3-chlorobenzylidene)-4-methylbenzenesulfonohydrazide (**58**)^77^, ^1^H NMR (400 MHz, CDCl_3_) *δ* 8.36 (bs, 1H), 7.91 (s, 1H), 7.89 (s, 1H), 7.73 (s, 1H), 7.59 (t, *J* = 1.8 Hz, 1H), 7.49 − 7.25 (m, 5H), 2.44 (s, 3H). The residue (**58**) (440 mg, 1.4 mmol) was dissolved in DMF (5 mL), Cs_2_CO_3_ (764 mg, 2.2 mmol) was added, and the mixture was heated to 100 °C, monitoring the progress of the reaction by TLC. The reaction mixture was cooled to rt and diluted with cold water. After extraction with EtOAc, the organic layers were collected, dried (anh. Na_2_SO_4_), filtered, and concentrated under reduced pressure. The residue was purified by FC (silica gel, EtOAc/hexane) to give **64** (285 mg, 70%) as white solid. ^1^H NMR (400 MHz, CDCl_3_) *δ* 8.62 (s, 2H), 7.90 (t, *J* = 1.9 Hz, 2H), 7.72 (dt, *J* = 7.4, 1.5 Hz, 2H), 7.52 − 7.35 (m, 4H), ^13 ^C NMR (101 MHz, MeOD) *δ* 134.38, 131.99, 130.02, 129.30, 128.40, 127.74, 127.38, 126.22, HRMS (ESI/QTOF) *m/z*: calcd for [M + H]^+^ C_14_H_10_Cl_2_N_3_^+^ 290.0246; found, 290.0246.

Ethyl 4–(3-chlorophenyl)-1*H*-1,2,3-triazole-5-carboxylate (**65**)^59^: To a stirred solution of ethyl-3–(4-chlorophenyl)propiolate (**59**) (209 mg, 1.0 mmol) in DMSO (2 mL), NaN_3_ (182 mg, 2.8 mmol) was added, and the solution was stirred under reflux at 70 °C for 6 h. After completion of the reaction, the mixture was cooled to rt and quenched with water. After extraction with EtOAc, the organic layer was collected, washed with brine, dried (anh. Na_2_SO_4_), filtered, and concentrated under reduced pressure. Purification by FC (silica gel, EtOAc/hexane) produced **65** (150 mg, 60%) as white solid. ^1^H NMR (400 MHz, MeOD) *δ* 7.83 (dd, *J* = 8.4, 1.7 Hz, 2H), 7.51 (dd, *J* = 8.5, 1.6 Hz, 2H), 4.39 (qd, *J* = 7.1, 1.4 Hz, 2H), 1.36 (td, *J* = 7.2, 1.4 Hz, 3H), ^13 ^C NMR (101 MHz, MeOD) *δ* 160.72, 135.17, 130.52, 128.08, 61.10, 13.00, HRMS (ESI/QTOF) *m/z*: calcd for [M + H]^+^ C_11_H_11_ClN_3_O_2_^+^ 252.0534; found, 252.0535.

4–(4-Chlorophenyl)-1*H*-1,2,3-triazole-5-carboxamide (**66**)^69^: To a stirred solution of 4-chlorobenzaldehyde (**55c**) (280 mg, 2.0 mmol), 2-cyanoacetamide (**60**) (169 mg, 2.0 mmol), and Et_3_N·HCl (566 mg, 5.0 mmol) in DMF (3 mL), NaN_3_ (390 mg, 6.0 mmol) was added. The mixture was stirred at 70 °C for 10 h before cooling to rt and adding water (20 mL) and 10% HCl solution (1 mL). After extraction with EtOAc (2 × 30 mL), the organic layer was collected, washed with water (3 × 50 mL) and brine solution (1 × 50 mL), dried (anh. Na_2_SO_4_), filtered, and concentrated under reduced pressure. The residue was subject to FC (SiO_2_, CH_2_Cl_2_/MeOH) to obtain **66** (288 mg, 65% yield) as white solid, ^1^H NMR (400 MHz, MeOD) *δ* 7.92 (d, *J* = 8.3 Hz, 2H), 7.47 (d, *J* = 8.3 Hz, 2H), ^13 ^C NMR (101 MHz, MeOD) *δ* 163.78, 134.64, 130.54, 128.25, HRMS (nanochip-ESI/LTQ-Orbitrap) *m/z*: calcd for [M + Na]^+^ C_9_H_7_ClN_4_ONa^+^ 245.0201; found, 245.0196.

4–(4-Chloro-2-hydroxyphenyl)-1*H*-1,2,3-triazole-5-carboxamide (**67**): Synthesised from 5-chloro-2-hydroxybenzaldehyde (**55b**) (314 mg, 2.0 mmol), 2-cyanoacetamide (**60**) (169 mg, 2.0 mmol), and Et_3_N·HCl (566 mg, 5.0 mmol), and NaN_3_ (390 mg, 6.0 mmol) using the same procedure as for **66** to give **67** (260 mg, 55% yield) as a white solid, m.p. 260–262 °C. ^1^H NMR (400 MHz, MeOD) *δ* 7.68 − 7.57 (m, 1H), 7.31 (ddd, *J* = 8.7, 2.7, 1.4 Hz, 1H), 6.96 (dd, *J* = 8.7, 1.5 Hz, 1H), ^13 ^C NMR (101 MHz, MeOD) *δ* 164.84, 154.03, 137.63, 130.39, 130.37, 124.01, 118.10, 116.86, HRMS (ESI/QTOF) *m/z*: calcd for [M + Na]^+^ C_9_H_7_ClN_4_O_2_Na^+^ 261.0150; found, 261.0159.

4-Chloro-2–(5-methyl-1,2,3-triazol-4-yl)phenol (**68**): Synthesised from 5-chloro-2-hydroxybenzaldehyde (**55b)** (312 mg, 2.0 mmol), nitromethane (180 mg, 2.4 mmol), and ammonium acetate (93 mg, 1.2 mmol) according to the same procedure as used for **63** to afford **68** (136 mg, 65%) as white solid, m.p. 149–151 °C. ^1^H NMR (400 MHz, CDCl_3_) *δ* 7.56 (d, *J* = 2.5 Hz, 1H), 7.25 (dd, *J* = 8.8, 2.6 Hz, 1H), 7.05 (d, *J* = 8.7 Hz, 1H), 2.69 (s, 3H), ^13 ^C NMR (101 MHz, MeOD) *δ* 170.80, 158.40, 149.45, 145.46, 138.72, 120.68, 19.07, HRMS (ESI/QTOF) *m/z*: calcd for [M + H]^+^ C_9_H_9_ClN_3_O^+^ 210.0429; found, 210.0431.

#### Synthesis of 4-Aryl-Tether-1,2,3-Triazoles and annulated derivatives

*N*-(4-Bromophenyl)-1,2,3-triazol-4-amine (**78**)^71^: 4-Bromo aniline (335 mg, 2.62 mmol) was dissolved in hydrochloric acid (6.03 mL of 2 M, 12.06 mmol), diluted with water (8 mL) and cooled to 0 °C. Sodium nitrite (181 mg, 2.62 mmol) was added and the mixture was stirred at 0 °C for 20 min before slowly adding a solution of 2-aminoacetonitrile monohydrochloride (**70**) (243 mg, 2.62 mmol) in water (3 mL). The mixture was stirred for 10 min at 0 °C before adding sodium acetate (3.02 g, 37.0 mmol) and allowing the mixture to warm to rt, where it was stirred for 1 h. The precipitate was collected by filtration and washed with water to afford 2–(3-(4-chlorophenyl)triaz-2-en-1yl)acetonitrile. The residue (238 mg, 1.0 mmol) was dissolved in EtOH (7 mL) and heated under reflux for 4 h. The mixture was allowed to cool to rt and concentrated under reduced pressure. The crude compound was triturated with CH_2_Cl_2_ to produce a cream solid which was purified by FC (40% EtOAc in petroleum ether) to afford **78** (167 mg, 70%) as a white solid. ^1^H NMR (400 MHz, MeOD) *δ* 7.39 (s, 1H), 7.35 − 7.25 (m, 2H), 7.19 (m, 2H), ^13 ^C NMR (101 MHz, MeOD) *δ* 148.34, 142.56, 131.44, 121.80, 116.57, 110.61, HRMS (ESI/QTOF) *m/z*: calcd for [M + H]^+^ C_8_H_8_BrN_4_^+^ 238.9927; found, 238.9933.

4-(Phenoxy)-1,2,3-triazole (**82**): Synthesised from (ethynyloxy)benzene (**77a**) (130 mg, 1.1 mmol) and TMSN_3_ according to the general procedure (**II**) to afford **82** (124 mg, 70%) as a white solid. ^1^H NMR (400 MHz, CDCl_3_) *δ* 11.08 (bs, 1H), 7.44 − 7.34 (m, 3H), 7.29 (s, 1H), 7.24 − 7.14 (m, 2H), ^13 ^C NMR (101 MHz, MeOD) *δ* 158.33, 156.97, 129.48, 123.62, 117.16, HRMS (ESI/QTOF) *m/z*: calcd for [M + H]^+^ C_8_H_8_N_3_O^+^ 162.0662; found, 162.0666.

4-(Phenylthio)-1,2,3-triazole (**83**): Synthesised from ethynyl(phenyl)sulfane (**77b**) (176 mg, 1.3 mmol) and TMSN_3_ according to the general procedure (**II**) to afford **83** (154 mg, 67%) as a white solid. ^1^H NMR (400 MHz, CDCl_3_) *δ* 7.72 (s, 1H), 7.42 − 7.24 (m, 5H), 3.42 (s, 2H), ^13 ^C NMR (101 MHz, MeOD) *δ* 135.18, 128.91, 128.52, 126.57, HRMS (ESI/QTOF) *m/z*: calcd for [M + H]^+^ C_8_H_8_N_3_S^+^ 178.0433; found, 178.0436.

4-(Phenylsulfonyl)-1*H*-1,2,3-triazole (**84**)^111^: To a stirred solution of compound **83** (60 mg 0.34 mmol) in methanol (2 mL), H_2_O_2_ (0.1 mL, 2.7 mmol) and a catalytic amount of ammonium molybdate (7 mg, 0.04 mmol) were added and allowed to stir at rt for 14 h, monitoring the progress of the reaction by TLC. After completion of the reaction, the solvent was removed under reduced pressure. The obtained solid products were dissolved in CH_2_Cl_2_ (2 mL) and water (2 mL), and the aqueous phase was extracted with CH_2_Cl_2_ (2 × 4 mL). The combined organic layers were washed with brine, dried over Na_2_SO_4_, filtered, and concentrated under reduced pressure. The residue was purified by FC to give **84** (57 mg, 80%) as a white solid. ^1^H NMR (400 MHz, CDCl_3_) *δ* 8.17 (s, 1H), 8.09 (dd, *J* = 7.3, 1.8 Hz, 2H), 7.69 − 7.61 (m, 1H), 7.57 (dd, *J* = 8.5, 6.9 Hz, 2H), ^13 ^C NMR (101 MHz, MeOD) *δ* 148.22, 140.70, 133.77, 129.19, 127.44, HRMS (ESI/QTOF) *m/z*: calcd for [M + Na]^+^ C_8_H_7_N_3_O_2_SNa^+^ 232.0151; found, 232.0154.

Phenyl(1,2,3-triazol-4-yl)methanol (**85**)^112^: Synthesised from 1-phenylprop-2-yn-1ol (**77c**) (198 mg, 1.5 mmol) and TMSN_3_ according to the general procedure (**II**) to afford **85** (184 mg, 70%) as a white solid. ^1^H NMR (400 MHz, CDCl_3_) *δ* 7.57 (s, 1H), 7.51 − 7.31 (m, 5H), 6.08 (s, 1H), ^13 ^C NMR (101 MHz, MeOD) *δ* 142.64, 128.16, 127.49, 126.22, 68.31, HRMS (ESI/QTOF) *m/z*: calcd for [M + Na]^+^ C_9_H_9_N_3_ONa^+^ 198.0640; found, 198.0638.

*N*-Phenyl-1*H*-1,2,3-triazole-4-carboxamide (**86**): Synthesised from *N-*phenylpropiolamide (**77d**) (145 mg, 1.0 mmol) and TMSN_3_ according to the general procedure (**II**) to afford **86** (94 mg, 50%) as a white solid. ^1^H NMR (400 MHz, CDCl_3_) *δ* 8.28 (s, 1H), 7.71 (d, *J* = 8.0 Hz, 2H), 7.44 − 7.35 (m, 2H), 7.17 (td, *J* = 7.3, 1.1 Hz, 1H), ^13 ^C NMR (101 MHz, MeOD) *δ* 159.40, 142.22, 137.70, 128.50, 124.37, 120.71, 120.59, HRMS (ESI/QTOF) *m/z*: calcd for [M + Na]^+^ C_9_H_8_N_4_ONa^+^ 211.0590; found, 211.0588.

Phenyl(1*H*-1,2,3-triazol-4-yl)methanone *O*-methyl oxime (**87**): To a stirred solution of phenyl(1*H*-1,2,3-triazol-4-yl)methanone (**99**)^113^ (50 mg, 0.29 mmol) in H_2_O (2 mL), and EtOH (1 mL), *O*-methylhydroxylamine hydrochloride (65 mg, 0.82 mmol) and NaOAc (104 mg, 1.28 mmol) were added. The flask was equipped with a reflux condenser and heated to 70 °C for 2 h. After cooling to rt, the mixture was extracted with EtOAc (3 × 5 mL). The organic layers were combined, dried over Na_2_SO_4_, filtered, and concentrated under reduced pressure. Purification by FC gave **87** (32 mg, 55%) as amorphous solid. ^1^H NMR (400 MHz, CDCl_3_) *δ* 8.16 (s, 1H), 7.67 − 7.59 (m, 2H), 7.54 − 7.42 (m, 3H), 4.20 (s, 3H), ^13 ^C NMR (101 MHz, DMSO) *δ* 148.52, 138.55, 137.27, 135.12, 129.65, 129.08, 128.47, 62.86, HRMS (ESI/QTOF) *m/z*: calcd for [M + H]^+^ C_10_H_11_N_4_O^+^ 203.0927; found, 203.0926.

3,8-Dihydroindeno[1,2-d][1,2,3]triazole (**89**)^74^: 1,2-Indandione-2-oxime (**71**) (161 mg, 1.0 mmol), hydrazine hydrate (97 mg, 3.0 mmol) and potassium hydroxide (168 mg, 3.0 mmol) were dissolved in diethyleneglycol (3 mL) under nitrogen atmosphere. The reactions mixture was heated to 170–190 °C and maintained at this temperature for 6 h. Upon completion of the reaction, the mixture was cooled to rt, and water (5 mL) and EtOAc (10 mL) were added. EtOAc (10 mL) was added, the phases were separated, and the aqueous phase was further extracted with EtOAc (3 × 10 mL). The combined organic phases were washed with brine, dried over Na_2_SO_4_, filtered, and concentrated under reduced pressure. The residue was purified by FC to yield **89** (63 mg, 40%) as a white solid. ^1^H NMR (400 MHz, CDCl_3_) *δ* 7.84 (dt, *J* = 7.6, 1.1 Hz, 1H), 7.57 (dp, *J* = 7.3, 1.0 Hz, 1H), 7.47 − 7.33 (m, 2H), 3.83 (d, *J* = 0.8 Hz, 3H), ^13 ^C NMR (101 MHz, MeOD) *δ* 153.40, 146.94, 131.79, 127.05, 126.00, 120.16, 27.32 HRMS (ESI/QTOF) *m/z*: calcd for [M + H]^+^ C_9_H_8_N_3_^+^ 158.0713; found, 158.0716.

#### Synthesis of ethynes 77a, 77b, 77d

(Ethynyloxy)benzene (**77a**)^114^: A mixture of phenol (**72**) (940 mg, 10.0 mmol) and NaOH powder (400 mg, 10 mmol) in DMSO (10 mL) was stirred at rt for 2 h before slowly adding trichloroethylene (**73**) (0.91 mL, 10.0 mmol). The mixture was stirred at rt until completion of the reaction. Then, water (25 mL) and CH_2_Cl_2_ (50 mL) were added, the phases were separated, and the aqueous phase was further extracted with CH_2_Cl_2_ (3 × 50 mL). The combined organic phases were washed with brine, dried over Na_2_SO_4_, filtered, and concentrated under reduced pressure. The crude residue was used in the next step without purification. To a solution of the residue (752 mg, 4.0 mmol) in dry Et_2_O (32 mL) at −78 °C, a solution of *n*-BuLi (2.5 M in hexane, 6.4 mL 16.0 mmol) was added under nitrogen atmosphere. After addition, the mixture was warmed to −20 °C over 1 h and stirred at this temperature for 1 h, before adding water (10 mL) and warming the mixture to rt. The phases were separated and the aqueous phase was extracted with CH_2_Cl_2_ (3 × 15 mL). The combined organic phases were washed with brine, dried over Na_2_SO_4_, filtered, and concentrated under reduced pressure. The residue was purified by FC (silica gel, EtOAc/hexane) to provide **77a** (189 mg, 40%) as a brown oil. ^1^H NMR (400 MHz, CDCl_3_) *δ* 7.45 − 7.36 (m, 2H), 7.40 − 7.26 (m, 2H), 7.19 (m, 1H), 2.12 (s, 1H).

Ethynyl(phenyl)sulfane (**77b**)^115^: To a solution of TMS-acetylene (**75**) (655 mg, 3.0 mmol) in 10 mL THF at −78 °C, *n*-BuLi (2.5 M in hexane, 1.1 mL, 3.0 mmol) was added dropwise and stirred for 1.5 h. A solution of diphenyl disulphide (**74**) (324 mg, 3.3 mmol) in 2 mL THF was added dropwise, and the mixture was warmed to rt over 2 h before adding H_2_O (5 mL). After extraction with Et_2_O (3 × 10 mL), the organic layer was collected, washed with brine, dried over Na_2_SO_4_), filtered, and concentrated under reduced pressure to yield the TMS-protected thioethyne. For deprotection, the thioethyne (412 mg 2.0 mmol) was stirred for 3 h at rt with KF (441 mg, 7.6 mmol) in MeOH (10 mL). After concentration under reduced pressure, CH_2_Cl_2_ (5 mL) and water (3 mL) were added. The organic layer was collected, dried (MgSO_4_) and filtered through a short silica plug to afford **77b** (192 mg, 85%) as a brown oil. ^1^H NMR (400 MHz, CDCl_3_) *δ* 7.51 − 7.44 (m, 2H), 7.42 − 7.33 (m, 2H), 7.30 − 7.22 (m, 1H), 3.28 (s, 1H).

*N*-Phenylpropiolamide (**77d**)^116^: Propiolic acid (350 mg, 5.0 mmol) and DCC (1.03 g, 5.0 mmol) were combined in 15 mL of CH_2_Cl_2_. The solution was stirred for 10 min before adding aniline (**76**) (466 mg, 5.0 mmol) and DMAP (7.5 mg, 0.06 mmol) in dry CH_2_Cl_2_ at 0 °C under nitrogen atmosphere. After complete addition, the mixture was stirred at rt for 18h. The reaction mixture was filtered and washed with 1 N HCl (5 mL) and a saturated solution of sodium chloride (2 × 50 mL). The organic phase was separated, dried over Na_2_SO_4_, filtered, and concentrated under reduced pressure. The residue was purified by FC (silica gel, EtOAc/hexane) to provide **77d** (652 mg, 90%) as a colourless oil. ^1^H NMR (400 MHz, CDCl_3_) *δ* 7.64 (bs, 1H), 7.58 − 7.51 (m, 2H), 7.42 − 7.33 (m, 2H), 7.18 (td, *J* = 7.3, 1.2 Hz, 1H), 2.95 (s, 1H).

#### General procedure (IV) for the synthesis of (aryl)(1,2,3-Triazolyl)methanol

Ethynylmagnesium bromide solution (1.2 eq, 0.5 M solution in THF) was added dropwise to a stirred solution of aldehydes (**90**) (1 eq) in anhydrous THF (6 mL) at 0 °C. The mixture was kept stirring at 0 °C for 20 min, then warmed to rt and stirred for an additional 2 h. The reaction mixture was quenched with a saturated NH_4_Cl solution (6 mL) and extracted with EtOAc (3 × 8 mL). The organic layers were combined, dried over anh. Na_2_SO_4_, filtered, and concentrated under reduced pressure to give the intermediate 1-aryl propargyl alcohols (**91**), which were directly used in next step. Alcohols (**91**) (1 eq), TMSN_3_ (1.5 eq), and CuI (0.05 eq) in DMF/MeOH solution (9:1) were stirred at 100 °C for 10–12 h. After consumption of the ethynyl substrate, the mixture was cooled to rt, the precipitate was filtered off, and the solution was concentrated under reduced pressure. The crude residue was purified by FC (SiO_2_, EtOAc/petroleum ether) to afford compounds **92a**–**92u** in 55–85% yields.

#### Synthesis of (aryl)(1,2,3-Triazolyl)methanol (92a–92u)

Phenyl(1,2,3-triazol-4-yl)methanol (**92a**)^112^: Synthesised from benzaldehyde (212 mg, 2.0 mmol), ethynylmagnesium bromide solution (4.8 mL, 0.5 M in THF, 2.4 mmol), and TMSN_3_ (345 mg, 3.0 mmol) according to the general procedure (**IV**) to afford **92a** (297 mg, 85%) as a white solid. ^1^H NMR (400 MHz, CDCl_3_) *δ* 7.57 (s, 1H), 7.51 − 7.31 (m, 5H), 6.08 (s, 1H).

(2-Chlorophenyl)(1,2,3-triazol-4-yl)methanol (**92b**, CAS [1511220–63-5]): Synthesised from 2-chlorobenzaldehyde (422 mg, 3.0 mmol) ethynylmagnesium bromide solution (7.2 mL, 0.5 M in THF, 3.6 mmol) and TMSN_3_ (518 mg, 4.5 mmol) according to the general procedure (**IV**) to afford **92b** (470 mg, 75%) as a white solid. ^1^H NMR (400 MHz, MeOD) *δ* 7.74 (dd, *J* = 8.0, 1.7 Hz, 1H), 7.57 (s, 1H), 7.44 − 7.37 (m, 2H), 7.41 − 7.27 (m, 1H), 6.36 (s, 1H).

(3-Chlorophenyl)(-1,2,3-triazol-4-yl)methanol (**92c**, CAS [1511815–17-0]): Synthesised from 3-chlorobenzaldehyde (422 mg, 3.0 mmol), ethynylmagnesium bromide solution (7.2 mL, 0.5 M in THF, 3.6 mmol) and TMSN_3_ (518 mg, 4.5 mmol) according to the general procedure (**IV**) to afford **92c** (501 mg, 75%) as a white solid. ^1^H NMR (400 MHz, CDCl_3_) *δ* 7.48 (s, 1H), 7.42 (q, *J* = 2.0 Hz, 1H), 7.27 (ddd, *J* = 9.5, 5.1, 1.5 Hz, 4H), 5.95 (t, *J* = 3.2 Hz, 1H).

(4-Chlorophenyl)(1,2,3-triazol-4-yl)methanol (**92d**, CAS [1429056–35-8]): Synthesised from 4-chlorobenzaldehyde (422 mg, 3.0 mmol), ethynylmagnesium bromide solution (7.2 mL, 0.5 M in THF, 3.6 mmol) and TMSN_3_ (518 mg, 4.5 mmol) according to the general procedure (**IV**) to afford **92d** (501 mg, 70%) as a white solid. ^1^H NMR (400 MHz, MeOD) *δ* 7.66 (s, 1H), 7.47 − 7.33 (m, 4H), 5.98 (s, 1H).

(2-Methoxyphenyl)(1,2,3-triazol-4-yl)methanol (**92e**, CAS [1518311–97-1]): Synthesised from 2-methoxybenzaldehyde (408 mg, 3.0 mmol), ethynylmagnesium bromide solution (7.2 mL, 0.5 M in THF, 3.6 mmol) and TMSN_3_ (518 mg, 4.5 mmol) according to the general procedure (**IV**) to afford **92e** (399 mg, 65%) as a white solid. ^1^H NMR (400 MHz, CDCl_3_) *δ* 7.52 (s, 1H), 7.40 − 7.27 (m, 2H), 7.04 − 6.90 (m, 2H), 6.21 (s, 1H), 3.84 (s, 3H).

(3-Methoxyphenyl)(1,2,3-triazol-4-yl)methanol (**92f**, CAS [1541589–58-5]): Synthesised from 3-methoxybenzaldehyde (408 mg, 3.0 mmol), ethynylmagnesium bromide solution (7.2 mL, 0.5 M in THF, 3.6 mmol) and TMSN_3_ (518 mg, 4.5 mmol) according to the general procedure (**IV**) to afford **92f** (338 mg, 55%) as a white solid. ^1^H NMR (400 MHz, MeOD) *δ* 7.63 (s, 1H), 7.27 (t, *J* = 7.9 Hz, 1H), 7.07 − 6.96 (m, 2H), 6.86 (dd, *J* = 8.3, 2.6 Hz, 1H), 5.95 (s, 1H), 3.80 (s, 3H).

(4-Methoxyphenyl)(1,2,3-triazol-4-yl)methanol (**92g**, CAS [1526953–98-9]): Synthesised from 4-methoxybenzaldehyde (408 mg, 3.0 mmol), ethynylmagnesium bromide solution (7.2 mL, 0.5 M in THF, 3.6 mmol) and TMSN_3_ (518 mg, 4.5 mmol) according to the general procedure (**IV**) to afford **92g** (338 mg, 55%) as a white solid. ^1^H NMR (400 MHz, MeOD) *δ* 7.62 (s, 1H), 7.38 − 7.30 (m, 2H), 6.99 − 6.88 (m, 2H), 5.93 (s, 1H), 3.80 (s, 3H).

(4-Bromophenyl)(1,2,3-triazol-4-yl)methanol (**92 h**, CAS [1934702–39-2]): Synthesised from 4-bromobenzaldehyde (555 mg, 3.0 mmol), ethynylmagnesium bromide solution (7.2 mL, 0.5 M in THF, 3.6 mmol) and TMSN_3_ (518 mg, 4.5 mmol) according to the general procedure (**IV**) to afford **92h** (546 mg, 72%) as a white solid. ^1^H NMR (400 MHz, MeOD) *δ* 7.66 (s, 1H), 7.56 − 7.49 (m, 2H), 7.42 − 7.34 (m, 2H), 5.96 (s, 1H).

(4-Fluorophenyl)(1,2,3-triazol-4-yl)methanol (**92i**, CAS [1517879–10-5]): Synthesised from 4-fluorobenzaldehyde (372 mg, 3.0 mmol), ethynylmagnesium bromide solution (7.2 mL, 0.5 M in THF, 3.6 mmol) and TMSN_3_ (518 mg, 4.5 mmol) according to the general procedure (**IV**) to afford **92i** (359 mg, 62%) as a white solid. ^1^H NMR (400 MHz, MeOD) *δ* 7.66 (s, 1H), 7.50 − 7.41 (m, 2H), 7.15 − 7.04 (m, 2H), 5.98 (s, 1H).

(5-Chloro-2-hydroxyphenyl)(1,2,3-triazol-4-yl)methanol (**92j**): Synthesised from 5-chloro-2-methoxybenzaldehyde (513 mg, 3.0 mmol), ethynylmagnesium bromide solution (7.2 mL, 0.5 M in THF, 3.6 mmol) and TMSN_3_ (518 mg, 4.5 mmol) according to the general procedure (**IV**) to afford **92j** (499 mg, 74%) as a white solid. ^1^H NMR (400 MHz, MeOD) *δ* 7.59 (s, 1H), 7.43 (d, *J* = 2.7 Hz, 1H), 7.11 (dd, *J* = 8.6, 2.7 Hz, 1H), 6.77 (d, *J* = 8.6 Hz, 1H), 6.24 (s, 1H).

(4-Chloro-2-hydroxyphenyl)(1,2,3-triazol-4-yl)methanol (**92k**): Synthesised from 4-chloro-2-methoxybenzaldehyde (513 mg, 3.0 mmol), ethynylmagnesium bromide solution (7.2 mL, 0.5 M in THF, 3.6 mmol) and TMSN_3_ (518 mg, 4.5 mmol) according to the general procedure (**IV**) to afford **92k** (466 mg, 69%) as a white solid. ^1^H NMR (400 MHz, MeOD) *δ* 7.58 − 7.48 (m, 2H), 7.02 (dd, *J* = 6.0, 2.2 Hz, 2H), 6.25 (s, 1H), 3.82 (s, 3H).

(4-Bromo-2-hydroxyphenyl)(1,2,3-triazol-4-yl)methanol (**92l**): Synthesised from 4-bromo-2-methoxybenzaldehyde (403 mg, 2.0 mmol), ethynylmagnesium bromide solution (4.8 mL, 0.5 M in THF, 2.4 mmol), and TMSN_3_ (345 mg, 3.0 mmol) according to the general procedure (**IV**) to afford **92l** (371 mg, 69%) as a white solid. ^1^H NMR (400 MHz, CDCl_3_) *δ* 7.66 (s, 1H), 7.11 − 6.96 (m, 3H), 5.72 (s, 1H).

Furan-2-yl(1,2,3-triazol-4-yl)methanol (**92 m**, CAS [1519476–28-8]): Synthesised from 2-furanal (289 mg, 3.0 mmol), ethynylmagnesium bromide solution (7.2 mL, 0.5 M in THF, 3.6 mmol) and TMSN_3_ (518 mg, 4.5 mmol) according to the general procedure (**IV**) to afford **92 m** (297 mg, 60%) as a white solid. ^1^H NMR (400 MHz, CDCl_3_) *δ* 8.05 (s, 1H), 7.94 − 7.74 (m, 1H), 7.51 − 7.37 (m, 1H), 6.45 − 6.25 (m, 1H), 6.12 − 5.99 (m, 1H).

Furan-3-yl(1,2,3-triazol-4-yl)methanol (**92n**, CAS [1500898–43-0]): Synthesised from 3-furanal (289 mg, 3.0 mmol), ethynylmagnesium bromide solution (7.2 mL, 0.5 M in THF, 3.6 mmol) and TMSN_3_ (518 mg, 4.5 mmol) according to the general procedure (**IV**) to afford **92n** (352 mg, 71%) as a white solid. ^1^H NMR (400 MHz, MeOD) *δ* 7.73 (s, 1H), 7.49 (d, *J* = 1.5 Hz, 2H), 6.47 (d, *J* = 2.9 Hz, 1H), 5.96 (s, 1H).

Thiophen-2-yl(1,2,3-triazol-4-yl)methanol (**92o**, CAS [1542859–11-9]): Synthesised from thiophene-2-carbaldehyde (224 mg, 2.0 mmol), ethynylmagnesium bromide solution (4.8 mL, 0.5 M in THF, 2.4 mmol), and TMSN_3_ (345 mg, 3.0 mmol) according to the general procedure (**IV**) to afford **92o** (239 mg, 66%) as a white solid. ^1^H NMR (400 MHz, MeOD) *δ* 7.75 − 7.70 (s, 1H), 7.37 (dd, *J* = 5.0, 1.4 Hz, 1H), 7.08 − 6.95 (m, 2H), 6.24 (s, 1H).

Thiophen-3-yl(1,2,3-triazol-4-yl)methanol (**92p**, CAS [1522175–89-8]): Synthesised from thiophene-3-carbaldehyde (336 mg, 3.0 mmol), ethynylmagnesium bromide solution (7.2 mL, 0.5 M in THF, 3.6 mmol) and TMSN_3_ (518 mg, 4.5 mmol) according to the general procedure (**IV**) to afford **92p** (396 mg, 73%) as a white solid. ^1^H NMR (400 MHz, MeOD) *δ* 7.68 (s, 1H), 7.40 (dd, *J* = 5.1, 3.0 Hz, 12H), 7.36 − 7.31 (m, 1H), 7.11 (dd, *J* = 5.0, 1.3 Hz, 1H), 6.07 (s, 1H).

Thiazol-4-yl(1,2,3-triazol-4-yl)methanol (**92q**, CAS [1935872–98-2]): Synthesised from thiazole-4-carbaldehyde (326 mg, 2.0 mmol), ethynylmagnesium bromide solution (4.8 mL, 0.5 M in THF, 2.4 mmol), and TMSN_3_ (345 mg, 3.0 mmol) according to the general procedure (**IV**) to afford **92q** (272 mg, 73%) as a white solid. ^1^H NMR (400 MHz, MeOD) *δ* 8.99 (s, 1H), 8.00 (s, 1H), 7.81 (s, 1H), 6.35 (s, 1H).

(1,2,3-Thiadiazol-4-yl)(1,2,3-triazol-4-yl)methanol (**92r**): Synthesised from 1,2,3thiadiazole-4-carbaldehyde (228 mg, 2.0 mmol), ethynylmagnesium bromide solution (4.8 mL, 0.5 M in THF, 2.4 mmol), and TMSN_3_ (345 mg, 3.0 mmol) according to the general procedure (**IV**) to afford **92r** (260 mg, 71%) as a white solid. ^1^H NMR (400 MHz, MeOD) *δ* 8.95 (s, 1H), 7.84 (s, 1H), 6.60 (s, 1H).

Pyridin-2-yl(1,2,3-triazol-4-yl)methanol (**92 s**): Synthesised from picolinaldehyde (322 mg, 3.0 mmol), ethynylmagnesium bromide solution (7.2 mL, 0.5 M in THF, 3.6 mmol) and TMSN_3_ (518 mg, 4.5 mmol) according to the general procedure (**IV**) to afford **92 s** (370 mg, 70%) as a yellow solid. ^1^H NMR (400 MHz, CDCl_3_) *δ* 8.68 − 8.58 (m, 1H), 8.24 (dt, *J* = 7.8, 1.1 Hz, 1H), 7.99 (s, 1H), 7.89 (td, *J* = 7.7, 1.8 Hz, 1H), 7.48 (ddd, *J* = 7.6, 4.8, 1.3 Hz, 1H), 5.60 (s, 1H).

Pyridin-3-yl(1,2,3-triazol-4-yl)methanol (**92t**, CAS [1508769–16-1]): Synthesised from nicotinaldehyde (322 mg, 3.0 mmol), ethynylmagnesium bromide solution (7.2 mL, 0.5 M in THF, 3.6 mmol) and TMSN_3_ (518 mg, 4.5 mmol) according to the general procedure (**IV**) to afford **92t** (396 mg, 75%) as a yellow solid. ^1^H NMR (400 MHz, MeOD) *δ* 8.64 (d, *J* = 2.2 Hz, 1H), 8.49 (dd, *J* = 4.9, 1.6 Hz, 1H), 7.93 (dt, *J* = 8.1, 2.0 Hz, 1H), 7.75 (s, 1H), 7.46 (dd, *J* = 8.0, 4.9 Hz, 1H), 6.08 (s, 1H)

Pyridin-4-yl(1,2,3-triazol-4-yl)methanol (**92 u**): Synthesised from isonicotinaldehyde (322 mg, 3.0 mmol), ethynylmagnesium bromide solution (7.2 mL, 0.5 M in THF, 3.6 mmol) and TMSN_3_ (518 mg, 4.5 mmol) according to the general procedure (**IV**) to afford **92 u** (359 mg, 68%) as a yellow solid. ^1^H NMR (400 MHz, CDCl_3_) *δ* 8.67 − 8.51 (m, 2H), 7.57 − 7.49 (m, 2H), 7.34 (s, 1H), 5.51 (s, 1H).

#### Synthesis of 5-Aroyl and 5-Hetaroyl-1,2,3-Triazoles

Phenyl(1*H*-1,2,3-triazol-5-yl)methanone (**99**)^113^: A solution of phenyl(1,2,3-triazol-4yl)methanol (**92a**) (88 mg, 0.5 mmol) in CH_2_Cl_2_ (3 mL) was treated with PCC (162 mg, 0.75 mmol). After stirring at rt for 2 h, the reaction was complete as determined by TLC. Excess PCC was removed by filtration of the reaction mixture through a pad of celite. The filtrate was sequentially washed with brine, dried over Na_2_SO_4_, filtered, and concentrated under reduced pressure. The residue as purified by FC (SiO_2_, EtOAc/petroleum ether) giving **99** (63 mg, 73%) as a oily solid. ^1^H NMR (400 MHz, CDCl_3_) *δ* 8.39 (s, 1H), 8.33 (d, *J* = 7.7 Hz, 1H), 7.71 − 7.64 (m, 1H), 7.57 (tt, *J* = 6.6, 1.2 Hz, 3H), ^13 ^C NMR (101 MHz, MeOD) *δ* 186.20, 136.83, 133.07, 129.76, 128.16, HRMS (ESI/QTOF) *m/z*: calcd for [M + Na]^+^ C_9_H_7_N_3_ONa^+^ 196.0481; found, 196.0481.

(2-Chlorophenyl)(1*H*-1,2,3-triazol-5-yl)methanone (**100**)^117^: Prepared the same way as **99** from (2-chlorophenyl)(1,2,3-triazol-4-yl)methanol (**92b**) (250 mg, 1.2 mmol) and PCC (389 mg, 1.8 mmol) in CH_2_Cl_2_ (8 mL), giving **100** (137 mg, 55% yield) as a white solid. ^1^H NMR (400 MHz, CDCl_3_) *δ* 8.32 (s, 1H), 7.64 − 7.58 (m, 1H), 7.55 − 7.46 (m, 2H), 7.43 (ddd, *J* = 7.6, 6.2, 2.3 Hz, 1H), ^13 ^C NMR (101 MHz, MeOD) *δ* 187.13, 145.63, 137.95, 131.76, 130.96, 129.95, 129.37, 126.64, HRMS (ESI/QTOF) *m/z*: calcd for [M + Na]^+^ C_9_H_6_ClN_3_ONa^+^ 230.0092; found, 230.0090.

(3-Chlorophenyl)(1*H*-1,2,3-triazol-5-yl)methanone (**101**)^118^: Prepared the same way as **99** from (3-chlorophenyl)(1,2,3-triazol-4-yl)methanol (**92c**) (320 mg, 1.5 mmol) and PCC (486 mg, 2.25 mmol) in CH_2_Cl_2_ (10 mL), giving **101** (186 mg, 60% yield) as white solid. ^1^H NMR (400 MHz, CDCl_3_) *δ* 8.47 − 8.21 (m, 3H), 7.64 (ddd, *J* = 8.0, 2.2, 1.1 Hz, 1H), 7.51 (t, *J* = 7.9 Hz, 1H), ^13 ^C NMR (101 MHz, MeOD) *δ* 184.44, 145.54, 138.36, 134.22, 132.76, 129.74, 129.58, 128.20, HRMS (ESI/QTOF) *m/z*: calcd for [M + Na]^+^ C_9_H_6_ClN_3_ONa 230.0097; found, 230.0096.

(4-Chlorophenyl)(1*H*-1,2,3-triazol-5-yl)methanone (**102**)^118^: Prepared the same way as **99** from (4-chlorophenyl)(1,2,3-triazol-4-yl)methanol (**92d**) (105 mg, 0.5 mmol) and PCC (162 mg, 0.75 mmol) in CH_2_Cl_2_ (3 mL) giving **102** (68 mg, 65% yield) as white solid. ^1^H NMR (400 MHz, MeOD) *δ* 8.48 (s, 1H), 8.38 − 8.27 (m, 2H), 7.66 − 7.54 (m, 2H), ^13 ^C NMR (101 MHz, MeOD) *δ* 184.67, 139.36, 135.21, 131.49, 128.38, HRMS (ESI/QTOF) *m/z*: calcd for [M + Na]^+^ C_9_H_6_ClN_3_ONa^+^ 230.0097; found, 230.0105.

(2-Methoxyphenyl)(1*H*-1,2,3-triazol-5-yl)methanone (**103**, CAS [1541622–39-2]): Prepared the same way as **99** from (2-methoxyphenyl)(1,2,3-triazol-5-yl)methanol (**92e**) (205 mg, 1.0 mmol) and PCC (324 mg, 1.5 mmol) in CH_2_Cl_2_ (5 mL) giving **103** (142 mg, 70% yield) as white solid. ^1^H NMR (400 MHz, CDCl_3_) *δ* 8.21 (s, 1H), 7.64 − 7.49 (m, 2H), 7.16 − 6.99 (m, 2H), 3.83 (s, 1H), ^13 ^C NMR (101 MHz, MeOD) *δ* 187.99, 157.89, 132.87, 129.44, 128.22, 120.16, 111.72, 54.77, HRMS (ESI/QTOF) *m/z*: calcd for [M + Na]^+^ C_10_H_9_N_3_O_2_Na^+^ 226.0587; found, 226.0589.

(3-Methoxyphenyl)(1*H*-1,2,3-triazol-5-yl)methanone (**104**)^118^: Prepared the same way as **99** from (3-methoxyphenyl)(1,2,3-triazol-5-yl)methanol (**92f**) (205 mg, 1.0 mmol) and PCC (324 mg, 1.5 mmol) in CH_2_Cl_2_ (5 mL) giving **104** (122 mg, 60% yield) as white solid. ^1^H NMR (400 MHz, MeOD) *δ* 8.46 (s, 1H), 7.87 (d, *J* = 7.6 Hz, 1H), 7.79 (d, *J* = 2.7 Hz, 1H), 7.49 (t, *J* = 8.0 Hz, 1H), 7.26 (dd, *J* = 8.1, 2.6 Hz, 1H), 3.90 (s, 3H), ^13 ^C NMR (101 MHz, MeOD) *δ* 185.82, 159.76, 138.00, 129.23, 122.35, 119.20, 114.25, 54.52, HRMS (ESI/QTOF) *m/z*: calcd for [M + Na]^+^ C_10_H_9_N_3_O_2_Na^+^ 226.0587; found, 226.0584.

(4-Methoxyphenyl)(1*H*-1,2,3-triazol-5-yl)methanone (**105**)^117^: Prepared the same way as **99** from (4-methoxyphenyl)(1,2,3-triazol-5-yl)methanol (**92g**) (240 mg, 1.2 mmol) and PCC (389 mg, 1.8 mmol) in CH_2_Cl_2_ (8 mL) giving **105** (171 mg, 70% yield) as white solid. ^1^H NMR (400 MHz, MeOD) *δ* 8.48 − 8.28 (m, 3H), 7.15 − 7.01 (m, 2H), 3.93 (s, 3H), ^13 ^C NMR (101 MHz, MeOD) *δ* 184.65, 164.23, 132.35, 129.34, 113.45, 54.70, HRMS (ESI/QTOF) *m/z*: calcd for [M + Na]^+^ C_10_H_9_N_3_O_2_Na^+^ 226.0587; found, 226.0590.

(2-Hydroxyphenyl)(1*H*-1,2,3-triazol-5-yl)methanone (**106**, CAS [2294215–75-9]): Synthesised from compound **103** (102 mg, 0.5 mmol) and 48% HBr in water (2 mL) using the general procedure (**III**) to give **106** (42 mg, 45%) as a yellow solid. ^1^H NMR (400 MHz, MeOD) *δ* 8.82 (d, *J* = 8.1 Hz, 1H), 8.52 (s, 1H), 7.59 (ddd, *J* = 8.7, 7.2, 1.7 Hz, 1H), 7.07 − 6.99 (m, 2H), ^13 ^C NMR (101 MHz, MeOD) *δ* 189.62, 163.06, 136.31, 132.91, 119.17, 118.80, 117.52, HRMS (ESI/QTOF) *m/z*: calcd for [M + Na]^+^ C_9_H_7_N_3_O_2_Na^+^ 212.0436; found, 212.0439.

(3-Hydroxyphenyl)(1*H*-1,2,3-triazol-5-yl)methanone (**107**, CAS [2294564–32-0]): Synthesised from compound **104** (102 mg, 0.5 mmol) and 48% HBr in water (2 mL) using the general procedure (**III**) to give **107** (46 mg, 49%) as a fluffy yellow powder. ^1^H NMR (400 MHz, MeOD) *δ* 8.43 (s, 1H), 7.72 (d, *J* = 7.7 Hz, 1H), 7.65 (s, 1H), 7.39 (t, *J* = 7.9 Hz, 1H), 7.10 (ddd, *J* = 8.1, 2.6, 1.0 Hz, 1H), ^13 ^C NMR (101 MHz, MeOD) *δ* 186.16, 157.44, 138.09, 129.24, 121.09, 120.24, 115.99, HRMS (ESI/QTOF) *m/z*: calcd for [M + Na]^+^ C_9_H_7_N_3_O_2_Na^+^ 212.0430; found, 212.0429.

(4-Hydroxyphenyl)(1*H*-1,2,3-triazol-5-yl)methanone (**108**, CAS [2296580–04-4]): Synthesised from compound **105** (102 mg, 0.5 mmol) and 48% HBr in water (2 mL) using the general procedure (**III**) to give **108** (49 mg, 50%) as a yellow solid. ^1^H NMR (400 MHz, MeOD) *δ* 8.39 (s, 1H), 8.24 (d, *J* = 8.4 Hz, 2H), 6.99 − 6.82 (m, 2H), ^13 ^C NMR (101 MHz, MeOD) *δ* 162.87, 132.64, 128.22, 114.86, HRMS (ESI/QTOF) *m/z*: calcd for [M + Na]^+^ C_9_H_7_N_3_O_2_Na^+^ 212.0430; found, 212.0438.

(4-Bromophenyl)(1*H*-1,2,3-triazol-5-yl)methanone (**109**)^118^: Prepared the same way as **99** from (4-bromophenyl)(1,2,3-triazol-4-yl)methanol (**92h**) (253 mg, 1.0 mmol) and PCC (324 mg, 1.5 mmol) in CH_2_Cl_2_ (5 mL) giving **109** (148 mg, 59% yield) as white solid. ^1^H NMR (400 MHz, CDCl_3_) *δ* 8.33 (s, 1H), 8.24 (d, *J* = 8.2 Hz, 2H), 7.76 − 7.61 (m, 2H), ^13 ^C NMR (101 MHz, MeOD) *δ* 184.88, 135.64, 131.56, 131.45, 127.99, HRMS (ESI/QTOF) *m/z*: calcd for [M + Na]^+^ C_9_H_6_BrN_3_ONa^+^ 273.9586; found, 273.9587.

(4-Fluorophenyl)(1*H*-1,2,3-triazol-5-yl)methanone (**110**)^113^: Prepared the same way as **99** from (4-fluorophenyl)(1,2,3-triazol-4-yl)methanol (**92i**) (193 mg, 1.0 mmol) and PCC (324 mg, 1.5 mmol) in CH_2_Cl_2_ (5 mL) giving **110** (86 mg, 45% yield) as white solid. ^1^H NMR (400 MHz, MeOD) *δ* 8.48 (s, 1H), 8.45 − 8.37 (m, 2H), 7.35 − 7.27 (m, 2H), ^13 ^C NMR (101 MHz, MeOD) *δ* 184.37, 167.18, 164.66, 145.64, 133.18, 133.15, 132.84, 132.74, 115.20, 114.98, HRMS (ESI/QTOF) *m/z*: calcd for [M + Na]^+^ C_9_H_6_FN_3_ONa^+^ 214.0387; found, 214.0386.

(5-Chloro-2-hydroxyphenyl)(1*H*-1,2,3-triazol-5-yl)methanone (**111**): Prepared the same way as **99** from (5-chloro-2-hydroxyphenyl)(1,2,3-triazol-4-yl)methanol (**92j**) (125 mg, 0.6 mmol) and PCC (194 mg, 1.5 mmol) in CH_2_Cl_2_ (4 mL) giving **111** (48 mg, 39% yield) as white solid, m.p. 186–188 °C. ^1^H NMR (400 MHz, MeOD) *δ* 8.95 (s, 1H), 8.55 (s, 1H), 7.56 (dd, *J* = 9.0, 2.7 Hz, 1H), 7.04 (d, *J* = 8.9 Hz, 1H), ^13 ^C NMR (101 MHz, MeOD) *δ* 188.32, 161.52, 135.86, 131.84, 123.48, 119.95, 119.34, HRMS (ESI/QTOF) *m/z*: calcd for [M + Na]^+^ C_9_H_6_ClN_3_O_2_Na^+^ 246.0046; found, 246.0051.

(4-Chloro-2-hydroxyphenyl)(1*H*-1,2,3-triazol-5-yl)methanone (**112**): Prepared the same way as **99** from (4-chloro-2-hydroxyphenyl)(1,2,3-triazol-4-yl)methanol (**92k**) (96 mg, 0.5 mmol) and PCC (162 mg, 0.75 mmol) in CH_2_Cl_2_ (3 mL) giving **112** (53 mg, 55% yield) as oily solid. ^1^H NMR (400 MHz, MeOD) *δ* 8.92 (d, *J* = 8.8 Hz, 1H), 8.55 (s, 1H), 7.12 − 6.99 (m, 2H), ^13 ^C NMR (101 MHz, MeOD) *δ* 188.61, 163.74, 141.90, 134.34, 119.32, 118.00, 117.42, HRMS (ESI/QTOF) *m/z*: calcd for [M + Na]^+^ C_9_H_6_ClN_3_O_2_Na^+^ 246.0041; found, 246.0038.

(4-Bromo-2-hydroxyphenyl)(1*H*-1,2,3-triazol-5-yl)methanone (**113**): Prepared the same way as **99** from (4-bromo-2-hydroxyphenyl)(1,2,3-triazol-4-yl)methanol (**92l**) (200 mg, 0.75 mmol) and PCC (243 mg, 1.13 mmol) in CH_2_Cl_2_ (5 mL) giving **113** (100 mg, 50% yield) as yellow solid, m.p. 192–194 °C. ^1^H NMR (400 MHz, MeOD) *δ* 8.79 (d, *J* = 8.7 Hz, 1H), 8.52 (s, 1H), 7.21 (d, *J* = 1.9 Hz, 1H), 7.16 (dd, *J* = 8.7, 2.0 Hz, 1H), ^13 ^C NMR (101 MHz, MeOD) *δ* 188.84, 163.46, 145.87, 134.20, 132.23, 130.50, 122.24, 120.58, 118.23, HRMS (ESI/QTOF) *m/z*: calcd for [M–H]^−^ C_9_H_6_BrN_3_O_2_^–^ 267.9545; found, 267.9543.

Furan-2-yl(1*H*-1,2,3-triazol-5-yl)methanone (**114**)^118^: Prepared the same way as **99** from furan-2-yl(1,2,3-triazol-4-yl)methanol (**92 m**) (165 mg, 1.0 mmol) and PCC (324 mg, 1.5 mmol) in CH_2_Cl_2_ (6 mL) giving **114** (57 mg, 35% yield) as white solid. ^1^H NMR (400 MHz, MeOD) *δ* 8.57 (s, 1H), 8.01 (s,1H), 7.97 − 7.93 (m, 1H), 6.77 (dd, *J* = 3.6, 1.7 Hz, 1H), ^13 ^C NMR (101 MHz, MeOD) *δ* 175.43, 151.23, 148.25, 121.80, 112.39, HRMS (ESI/QTOF) *m/z*: calcd for [M + Na]^+^ C_7_H_5_N_3_O_2_Na^+^ 186.0274; found, 186.0271.

Furan-3-yl(1*H*-1,2,3-triazol-5-yl)methanone (**115**, CAS [1522300–51-1]): Prepared the same way as **99** from furan-3-yl(1,2,3-triazol-4-yl)methanol (**92n**) (165 mg, 1.0 mmol) and PCC (324 mg, 1.5 mmol) in CH_2_Cl_2_ (6 mL) giving **115** (83 mg, 51% yield) as white solid. ^1^H NMR (400 MHz, MeOD) *δ* 8.89 (s, 1H), 8.43 (s, 1H), 7.68 (t, *J* = 1.7 Hz, 1H), 7.05 (d, *J* = 1.8 Hz, 1H), ^13 ^C NMR (101 MHz, MeOD) *δ* 179.64, 150.59, 143.92, 125.78, 108.79, HRMS (ESI/QTOF) *m/z*: calcd for [M + Na]^+^ C_7_H_5_N_3_O_2_Na^+^ 186.0274; found, 186.0274.

Thiophen-2-yl(1*H*-1,2,3-triazol-5-yl)methanone (**116**, CAS [1540541–16-9]): Prepared the same way as **99** from thiophen-2-yl(1,2,3-triazol-4-yl)methanol (**92o**) (200 mg, 1.1 mmol) and PCC (356 mg, 1.65 mmol) in CH_2_Cl_2_ (6 mL) giving **116** (69 mg, 35% yield) as white solid. ^1^H NMR (400 MHz, MeOD) *δ* 8.60 (d, *J* = 3.8 Hz, 1H), 8.47 (s, 1H), 7.97 (dd, *J* = 4.9, 1.2 Hz, 1H), 7.30 (dd, *J* = 5.0, 3.9 Hz, 1H), ^13 ^C NMR (101 MHz, MeOD) *δ* 177.70, 145,71, 142.24, 135.64, 135.19, 128.14, HRMS (ESI/QTOF) *m/z*: calcd for [M + Na]^+^ C_7_H_5_N_3_OSNa^+^ 202.0046; found, 202.0042.

Thiophen-3-yl(1*H*-1,2,3-triazol-5-yl)methanone (**117**)^118^: Prepared the same way as **99** from thiophen-3-yl(1,2,3-triazol-4-yl)methanol (**92p**) (200 mg, 1.1 mmol) and PCC (356 mg, 1.65 mmol) in CH_2_Cl_2_ (6 mL) giving **117** (81 mg, 41% yield) as white solid. ^1^H NMR (400 MHz, MeOD) *δ* 8.99 (s, 1H), 8.46 (s, 1H), 7.86 (d, *J* = 5.1 Hz, 1H), 7.55 (dd, *J* = 5.2, 2.9 Hz, 1H), ^13 ^C NMR (101 MHz, MeOD) *δ* 179.25, 147.86, 140.32, 135.87, 127.67, 125.82, HRMS (ESI/QTOF) *m/z*: calcd for [M + Na]^+^ C_7_H_5_N_3_OSNa^+^ 202.0046; found, 202.0044.

Thiazol-4-yl(1*H*-1,2,3-triazol-5-yl)methanone (**118**, CAS [1935572–44-3]): Prepared the same way as **99** from thiazol-4-yl(1,2,3-triazol-4-yl)methanol (**92q**) (225 mg, 1.25 mmol) and PCC (405 mg, 1.88 mmol) in CH_2_Cl_2_ (8 mL) giving **118** (143 mg, 64% yield) as white solid. ^1^H NMR (400 MHz, MeOD) *δ* 9.35 (s, 1H), 9.23 (s, 1H), 8.53 (s, 1H), ^13 ^C NMR (101 MHz, DMSO) *δ* 177.42, 162.18, 149.95, 145.47, 137.72, 131.16, HRMS (ESI/QTOF) *m/z*: calcd for [M + H]^+^ C_6_H_5_N_4_OS^+^ 181.0179; found, 181.0175.

(1,2,3-Thiadiazol-4-yl)(1*H*-1,2,3-triazol-5-yl)methanone (**119**): Prepared the same way as **99** from (1,2,3-thiadiazol-4-yl(1,2,3-triazol-4-yl)methanol (**92r**) (275 mg, 1.5 mmol) and PCC (486 mg, 2.25 mmol) in CH_2_Cl_2_ (8 mL) giving **119** (117 mg, 43% yield) as white solid, m.p. 170–172 °C. ^1^H NMR (400 MHz, MeOD) *δ* 10.10 (s, 1H), 8.99 (s, 1H), ^13 ^C NMR (101 MHz, MeOD) *δ* 175.58, 160.36, 145.03, HRMS (ESI/QTOF) *m/z*: calcd for [M + Na]^+^ C_5_H_3_N_5_NaOSNa^+^ 203.9951; found, 203.9947.

Pyridin-2-yl(1*H*-1,2,3-triazol-5-yl)methanone (**120**): Prepared the same way as **99** from pyridin-2-yl(1,2,3-triazol-4-yl)methanol (**92 s**) (240 mg, 1.2 mmol) and PCC (389 mg, 1.8 mmol) in CH_2_Cl_2_ (8 mL) giving **120** (114 mg, 55% yield) as white solid, m.p. 161–163 °C. ^1^H NMR (400 MHz, MeOD) *δ* 9.00 (s, 1H), 8.85 − 8.74 (m, 1H), 8.27 (dt, *J* = 7.8, 1.2 Hz, 1H), 8.07 (td, *J* = 7.7, 1.7 Hz, 1H), 7.69 (ddd, *J* = 7.6, 4.8, 1.2 Hz, 1H), ^13 ^C NMR (101 MHz, MeOD) *δ* 182.94, 153.16, 148.91, 137.36, 133.15, 127.47, 123.57, HRMS (ESI/QTOF) *m/z*: calcd for [M + Na]^+^ C_8_H_6_N_4_ONa^+^ 197.0434; found, 197.0432.

Pyridin-3-yl(1*H*-1,2,3-triazol-5-yl)methanone (**121**, CAS [1523140–26-2]): Prepared the same way as **99** from pyridin-3-yl(1,2,3-triazol-4-yl)methanol (**92t**) (270 mg, 1.5 mmol) and PCC (486 mg, 2.25 mmol) in CH_2_Cl_2_ (10 mL) giving **121** (104 mg, 40% yield) as white solid. ^1^H NMR (400 MHz, MeOD) *δ* 9.45 (d, *J* = 2.2 Hz, 1H), 8.81 (dd, *J* = 5.0, 1.7 Hz, 1H), 8.73 (d, *J* = 8.1 Hz, 1H), 8.51 (brs, 1H), 7.66 (dd, *J* = 8.0, 4.9 Hz, 1H), ^13 ^C NMR (101 MHz, MeOD) *δ* 184.19, 152.35, 150.31, 138.15, 132.93, 123.80, HRMS (ESI/QTOF) *m/z*: calcd for [M + H]^+^ C_8_H_7_N_4_O^+^ 175.0614; found, 175.0613.

Pyridin-4-yl(1*H*-1,2,3-triazol-5-yl)methanone (**122**): Prepared the same way as **99** from pyridin-4-yl(1,2,3-triazol-4-yl)methanol (**92 u**) (165 mg, 1.0 mmol) and PCC (324 mg, 1.5 mmol) in CH_2_Cl_2_ (6 mL) giving **122** (96 mg, 52% yield) as white solid, m.p. 221–223 °C. ^1^H NMR (400 MHz, MeOD) *δ* 8.89 − 8.76 (m, 2H), 8.57 (s, 1H), 8.25 − 8.12 (m, 2H), ^13 ^C NMR (101 MHz, MeOD) *δ* 184.76, 149.70, 145.27, 143.91, 132.09, 123.40, HRMS (ESI/QTOF) *m/z*: calcd for [M + H]^+^ C_8_H_7_N_4_O^+^ 175.0614; found, 175.0613.

(4-Bromophenyl)(4-(hydroxymethyl)-1*H*-1,2,3-triazol-5-yl)methanone (**123**) and (4-(Bromomethyl)-1*H*-1,2,3-triazol-5-yl)(4-bromophenyl)methanone (**124**): To a solution of 2-(prop-2-ynyloxy)-tetrahydro-2*H*-pyran (**93**) (1.43 g, 7.7 mmol) in THF (15 mL), *n*-BuLi (1.6 M, 4.82 mL, 7.7 mmol) was added at −78 °C. The reaction mixture was kept at −78 °C for 1 h, then 4-bromobenzaldehyde (982 mg, 7.0 mmol) was added at −78 °C. The reaction mixture was stirred for 2 h, then slowly warmed to −30 °C before being poured into an aqueous solution of NaHCO_3_ (10 mL). After extraction with EtOAc (3 × 100 mL), the organic layer was collected, dried over anh. Na_2_SO_4_, filtered, and concentrated under reduced pressure to afford (1–(4-bromophenyl)-4-((tetrahydro-2*H*-pyran-2-yl)oxy)but-2-yn-1-ol (**94**) (2.1 g, 88%) as colourless oil. ^1^H NMR (400 MHz, CDCl_3_) *δ* 7.56 − 7.41 (m, 4H), 5.50 (dd, *J* = 4.8, 3.0 Hz, 1H), 4.83 (t, *J* = 3.4 Hz, 1H), 4.37 (qt, *J* = 15.7, 1.6 Hz, 2H), 3.86 (ddd, *J* = 11.5, 9.0, 3.1 Hz, 1H), 3.55 (ddt, *J* = 12.6, 5.3, 2.8 Hz, 1H), 2.30 (d, *J* = 6.0 Hz, 1H), 1.93 − 1.59 (m, 4H), 1.56 (ddd, *J* = 11.6, 5.3, 3.0 Hz, 2H). To a solution of compound **94** (1.3 g, 4.0 mmol) in CH_2_Cl_2_ (50 mL), MnO_2_ (3.6 g, 41.2 mmol) was added. The suspension was stirred at rt for 5 h and then filtered over a pad of celite. After removing the solvent under reduced pressure, the crude product was filtered over a pad of silica gel and washed with petroleum ether:EtOAc (10:1) to afford (1–(4-bromophenyl)-4-((tetrahydro-2*H*-pyran-2-yl)oxy)but-2-yn-1-one (**95**) (1.25 g, 97%) as yellow oil. ^1^H NMR (400 MHz, CDCl_3_) *δ* 8.14–8.17 (m, 2H), 7.61–7.66 (m, 1H), 7.48–7.53 (m, 1H), 4.90–4.92 (m, 1H), 4.57 (s, 2H), 3.86–3.92 (m, 1H), 3.58–3.61 (m, 1H), 1.55–1.86 (m, 6H). To a solution of compound **95** (970 mg, 3.0 mmol) in EtOH (10 mL), pyridinium *p*-toluenesulfonate (158 mg, 0.63 mmol) was added. The resulting solution was stirred for 4 h at 50 °C before being poured into water (30 mL). After extraction with Et_2_O (3 × 50 mL), the organic layer was collected, dried over anh. Na_2_SO_4_, filtered, and concentrated under reduced pressure. The residue was purified by FC to afford (1–(4-bromophenyl)-4-hydroxybut-2-yn-1-one (**96**) (534 mg, 75%) as white solid. ^1^H NMR (400 MHz, CDCl_3_) *δ* 8.06 − 7.99 (m, 2H), 7.71 − 7.63 (m, 2H), 4.60 (d, *J* = 6.4 Hz, 2H). Compound **123** was synthesised from **96** (400 mg, 1.7 mmol) and TMSN_3_ according to the general procedure (**II**) to give **123** (239 mg, 50%) as a brown fluffy powder, m.p. 240–242 °C. ^1^H NMR (400 MHz, MeOD) *δ* 8.26 (d, *J* = 8.3 Hz, 2H), 7.81 − 7.68 (m, 2H), 5.02 (s, 2H), ^13 ^C NMR (101 MHz, MeOD) *δ* 186.07, 135.94, 131.74, 131.27, 127.73, 55.27, HRMS (ESI/QTOF) *m/z*: calcd for [M + Na]^+^ C_10_H_8_BrN_3_O_2_Na^+^ 303.9698; found, 303.9713. A solution of PPh_3_ (135 mg, 0.52 mmol) and CBr_4_ (173 mg, 0.52 mmol) in dry DMF (4 mL) was strirred for 10 min before adding compound **123** (121 mg, 0.43 mmol) and monitoring the reaction by TLC. After completion, the reaction was quenched with an aq. solution of sodium metabisulfite and extracted with EtOAc (3 × 5 mL). The organic layers were collected, dried over Na_2_SO_4_, filtered, and concentrated under reduced pressure. The crude product was purified by FC (silica gel, EtOAc/hexane, 1:3) to afford **124** (125 mg, 85%) as white solid, m.p. 121–123 °C. ^1^H NMR (400 MHz, CDCl_3_) *δ* 8.23 (d, *J* = 8.3 Hz, 2H), 7.69 (d, *J* = 8.5 Hz, 2H), 4.93 (s, 2H), ^13 ^C NMR (101 MHz, MeOD) *δ* 185.65, 135.72, 131.70, 131.34, 127.99, 22.80, HRMS (ESI/QTOF) *m/z*: calcd for [M + Na]^+^ C_10_H_8_BrN_3_O_2_Na^+^ 367.8833; found, 367.8830.

(4-(Aminomethyl)-1*H*-1,2,3-triazol-5-yl)(4-bromophenyl)methanone (**126**): To a solution of 4-bromobenzoyl chloride (1.32 g, 6.0 mmol) and *t*-butyl prop-2-yn-1-ylcarbamate (**97**) (621 mg, 4.0 mmol) in anhydrous THF (10 mL), PdCl_2_(PPh_3_)_2_ (26 mg, 0.04 mmol) and CuI (23 mg, 0.12 mmol) were added under a nitrogen atmosphere. After 1 min of stirring, Et_3_N (0.6 mL, 5.0 mmol) was added, and the reaction was stirred for 40 min at rt. During this time, Et_3_NHCl precipitated, and the solution assumed a dark orange/brown colour. The reaction was diluted with Et_2_O (30 mL) and washed with H_2_O (30 mL). After extraction with CH_2_Cl_2_ (3 × 50 mL), the organic layers were combined, dried over Na_2_SO_4_, filtered, and concentrated under reduced pressure. The residue was purified by FC to afford (*tert*-butyl (4–(4-bromophenyl)-4-oxobut-2-yn-1-yl)carbamate (**98**) (1.82 g, 90%) as colourless oil. ^1^H NMR (400 MHz, CDCl_3_) *δ* 8.05 − 7.92 (m, 2H), 7.74 − 7.58 (m, 2H), 4.90 (bs, 1H), 4.24 (d, *J* = 5.9 Hz, 2H), 1.51 (s, 9H). *Tert*-butyl ((5–(4-bromobenzoyl)-1*H*-1,2,3-triazol-5-yl)methyl)carbamate (**125**) was synthesised from **98** (500 mg, 1.5 mmol) and TMSN_3_ according to the general procedure (**II**) to afford **125** (257 mg, 45%) as solid. ^1^H NMR (400 MHz, CDCl_3_) *δ* 8.31 (d, *J* = 8.3 Hz, 2H), 7.73 − 7.65 (m, 2H), 5.57 (s, 1H), 4.73 (d, *J* = 6.2 Hz, 2H), 1.48 (s, 9H). To a solution of compound **125** (190 mg, 0.5 mmol) in CH_2_Cl_2_ (4 mL), trifluoroacetic acid (0.37 mL, 5 mmol) was added and the mixture was stirred overnight at rt. The solution was concentrated, basified with NaHCO_3_ solution, and extracted with EtOAc. The organic layer was dried over Na_2_SO_4_, filtered, concentrated under reduced pressure and triturated with Et_2_O to afford **126** (105 mg, 75%) as white solid, m.p. 190–192 °C. ^1^H NMR (400 MHz, MeOD) *δ* 8.25 − 8.18 (m, 2H), 7.75 − 7.69 (m, 2H), 4.36 (s, 2H), ^13 ^C NMR (101 MHz, DMSO) *δ* 185.98, 138.22, 132.82, 131.42, 126.27, HRMS (ESI/QTOF) *m/z*: calcd for [M + H]^+^ C_10_H_10_BrN_4_O^+^ 281.0032; found, 281.0038.

#### General procedure (V) for the synthesis of 3-Aryl-1H-1,2,4-Triazoles

The commercially available or synthetically prepared aryl nitriles (**130**) (1 mmol) were dissolved in DMF (3 mL), and formic acid (1.0 mL/mmol) and hydrazine hydrate (1.0 mL) were added. The reaction mixture was heated under reflux (90 °C) for 12 h. Upon completion of the reaction as determined by TLC, the reaction mixture was cooled to rt and poured into ice-water slurry (5 mL). After extraction with EtOAc (3 × 10 mL), the organic phases were collected, washed with brine solution (2 × 5 mL), dried over Na_2_SO_4_, filtered and concentrated under reduced pressure. The residue was purified by FC (SiO_2_, EtOAc/petroleum ether) to afford aryl 1*H*-1,2,4-triazoles (**134**–**154** and **158**–**161**). Overall yields 20–45%.

#### Synthesis of 3-Aryl-1H-1,2,4-Triazoles (134–154 and 158–161)

3–(4-Fluorophenyl)-1*H*-1,2,4-triazole (**134**)^81^: Synthesised from 4-fluorobenzonitrile (**130a**) (123 mg, 1 mmol) according to the general procedure (**V**) to afford **134** (43 mg, 30%) as a white solid. ^1^H NMR (400 MHz, CDCl_3_) *δ* 8.35 (s, 1H), 8.11 − 8.00 (m, 2H), 7.22 − 7.13 (m, 2H), ^13 ^C NMR (101 MHz, MeOD) *δ* 164.98, 162.51, 128.28, 128.20, 125.72, 115.51, 115.29, HRMS (ESI/QTOF) *m/z*: calcd for [M + H]^+^ C_8_H_7_FN_3_^+^ 164.0619; found, 164.0619.

3–(3-Bromophenyl)-1*H*-1,2,4-triazole (**135**)^119^: Synthesised from 3-bromobenzonitrile (**130 b**) (181 mg, 1 mmol) according to the general procedure (**V**) to afford **135** (55 mg, 25%) as a white solid. ^1^H NMR (400 MHz, MeOD) *δ* 8.46 (s, 1H), 8.22 (t, *J* = 1.8 Hz, 1H), 8.02 (dt, *J* = 7.9, 1.3 Hz, 1H), 7.63 (dt, *J* = 8.1, 1.3 Hz, 1H), 7.43 (t, *J* = 7.9 Hz, 1H), ^13 ^C NMR (101 MHz, MeOD) *δ* 134.46, 132.22, 130.32, 128.90, 124.69, 122.40, HRMS (ESI/QTOF) *m/z*: calcd for [M + H]^+^ C_8_H_7_BrN_3_^+^ 223.9818; found, 223.9815.

3–(3-Fluorophenyl)-1*H*-1,2,4-triazole (**137**)^120^: Synthesised from 3-fluorobenzonitrile (**130c**) (121 mg, 1 mmol) according to the general procedure (**V**) to afford **137** (49 mg, 30%) as white solid. ^1^H NMR (400 MHz, CDCl_3_) *δ* 8.33 (d, *J* = 8.9 Hz, 1H), 7.84 (dt, *J* = 7.4, 1.3 Hz, 1H), 7.75 (ddd, *J* = 9.7, 2.7, 1.5 Hz, 1H), 7.44 (td, *J* = 8.0, 5.8 Hz, 1H), 7.13 (tdd, *J* = 8.4, 2.6, 1.1 Hz, 1H), ^13 ^C NMR (101 MHz, MeOD) *δ* 164.28, 161.85, 130.47, 130.39, 121.87, 121.84, 112.85, 112.61, HRMS (ESI/QTOF) *m/z*: calcd for [M + H]^+^ C_8_H_7_FN_3_^+^ 164.0619; found, 164.0620.

2-Fluoro-6-(1*H*-1,2,4-triazol-3-yl)aniline (**140**, CAS [1503176–05-3]): Synthesised from 2-amino-3-fluorobenzonitrile (**130d**) (136 mg, 1 mmol) according to the general procedure (**V**) to afford **140** (44 mg, 25%) as white solid. ^1^H NMR (400 MHz, MeOD) *δ* 8.46 (brs, 1H), 7.71 (brs, 1H), 7.12 − 6.94 (m, 1H), 6.69 (td, *J* = 8.0, 5.2 Hz, 1H), ^13 ^C NMR (101 MHz, MeOD) *δ* 153.20, 150.84, 115.43, 115.35, HRMS (ESI/QTOF) *m/z*: calcd for [M + H]^+^ C_8_H_8_FN_4_^+^ 179.0728; found, 179.0732.

2-Chloro-6-(1*H*-1,2,4-triazol-3-yl)aniline (**141**, CAS [1698578–50-5]): Synthesised from 2-amino-3-chlorobenzonitrile (**130e**) (152 mg, 1 mmol) according to the general procedure (**V**) to afford **141** (77 mg, 40%) as white solid. ^1^H NMR (400 MHz, MeOD) *δ* 8.44 (s, 1H), 7.99 (d, *J* = 2.6 Hz, 1H), 7.31 (dd, *J* = 8.8, 2.6 Hz, 1H), 6.99 (d, *J* = 8.8 Hz, 1H), ^13 ^C NMR (101 MHz, MeOD) *δ* 155.11, 130.65, 125.97, 123.92, 118.28, HRMS (ESI/QTOF) *m/z*: calcd for [M + H]^+^ C_8_H_8_ClN_4_^+^ 195.0432; found, 195.0431.

2-(1*H*-1,2,4-triazol-3-yl)benzene-1,3-diamine (**142**): Synthesised from 2,6-dinitrobenzonitrile (**130f**) (193 mg, 1 mmol) according to the general procedure (**V**) to afford 3–(2,6-dinitrophenyl)-1*H*-1,2,4-triazole **142a** (77 mg, 40%) as yellow solid. ^1^H NMR (400 MHz, MeOD) *δ* 8.60 (s, 1H), 8.33 (d, *J* = 8.2 Hz, 2H), 7.98 (t, *J* = 8.2 Hz, 1H). Following the same procedure as used for **155**, 3–(2,6-dinitrophenyl)-1*H*-1,2,4-triazole (**142a**) (60 mg, 0.25 mmol) was mixed with SnCl_2_ ·2H_2_O (281 mg, 1.25 mmol) to obtain **142** (20 mg, 45%) as white solid, m.p. 152–154 °C. ^1^H NMR (400 MHz, MeOD) *δ* 8.50 (s, 1H), 6.89 (t, *J* = 7.9 Hz, 1H), 6.22 (d, *J* = 7.9 Hz, 2H), ^13 ^C NMR (101 MHz, MeOD) *δ* 147.47, 147.41, 142.49, 129.70, 129.59, 105.78, 100.52, HRMS (ESI/QTOF) *m/z*: calcd for [M + H]^+^ C_8_H_10_N_5_^+^ 176.0931; found, 176.0936.

4-Fluoro-2-(1*H*-1,2,4-triazol-3-yl)aniline (**143**, CAS [147004–95-3]): Synthesised from 2-amino-5-fluorobenzonitrile (**130g**) (136 mg, 1 mmol) according to the general procedure (**V**) to afford **143** (44 mg, 25%) as white solid. ^1^H NMR (400 MHz, MeOD) *δ* 8.46 (brs, 1H), 7.63 (brs, 1H), 6.95 (td, *J* = 8.5, 3.0 Hz, 1H), 6.85 (dd, *J* = 8.9, 4.9 Hz, 1H), ^13 ^C NMR (101 MHz, MeOD) *δ* 156.18, 153.86, 142.73, 117.67, 117.59, 113.12, HRMS (ESI/QTOF) *m/z*: calcd for [M + H]^+^ C_8_H_8_FN_4_^+^ 179.0728; found, 179.0732.

4-Bromo-5-fluoro-2-(1*H*-1,2,4-triazol-3-yl)aniline (**144**): Synthesised from 2-amino5-bromo-4-fluorobenzonitrile (**130h**) (214 mg, 1 mmol) according to the general procedure (**V**) to afford **144** (72 mg, 28%) as a white solid, m.p. 243–246 °C. ^1^H NMR (400 MHz, CDCl_3_) *δ* 8.47 (s, 1H), 8.05 (dd, *J* = 7.5, 2.0 Hz, 1H), 6.58 (dd, *J* = 10.4, 1.9 Hz, 1H), ^13 ^C NMR (101 MHz, MeOD) *δ* 160.95, 148.13, 132.07, 102.62, 102.37, 93.00, 92.79, HRMS (ESI/QTOF) *m/z*: calcd for [M + H]^+^ C_8_H_7_BrFN_4_^+^ 256.9833; found, 256.9834.

4-Chloro-*N*-methyl-2-(1*H*-1,2,4-triazol-3-yl)aniline (**145**): Synthesised from 5chloro-2-(methylamino)benzonitrile (**130an**) (166 mg, 1 mmol) according to the general procedure (**V**) to afford **145** (52 mg, 25%) as white solid, m.p. 207–209 °C. ^1^H NMR (400 MHz, CDCl_3_) *δ* 8.24 (s, 1H), 7.89 (s, 1H), 7.54 (s, 1H), 6.70 (d, *J* = 8.9 Hz, 1H), 2.98 (s, 3H), ^13 ^C NMR (101 MHz, MeOD) *δ* 150.42, 129.22, 125.32, 121.42, 120.07, 117.27, 29.27, HRMS (ESI/QTOF) *m/z*: calcd for [M + H]^+^ C_9_H_10_ClN_4_^+^ 209.0589; found, 209.0587.

*N*-(4-Chloro-2-(1*H*-1,2,4-triazol-3-yl)phenyl)acetamide (**146**): Synthesised from *N*-(4-chloro-2-cyanophenyl)acetamide (**130aa**) (194 mg, 1 mmol) according to the general procedure (**V**) to afford **146** (62 mg, 26%) as yellow amorphous solid. ^1^H NMR (400 MHz, MeOD) *δ* 8.63 − 8.46 (m, 2H), 8.15 (d, *J* = 2.5 Hz, 1H), 7.39 (dd, *J* = 9.0, 2.5 Hz, 1H), 2.25 (s, 3H), ^13 ^C NMR (101 MHz, MeOD) *δ* 169.95, 135.41, 129.27, 128.19, 127.03, 121.97, 23.47, HRMS (ESI/QTOF) *m/z*: calcd for [M + Na]^+^ C_10_H_9_ClN_4_ONa^+^ 259.0357; found, 259.035.

*N*-(4-Chloro-2-(1*H*-1,2,4-triazol-3-yl)phenyl)propionamide (**147**): Synthesised from *N*-(4-chloro-2-cyanophenyl)propionamide (**130ab**) (208 mg, 1 mmol) according to the general procedure (**V**) to afford **147** (75 mg, 30%) as yellow solid, m.p. 168–171 °C. ^1^H NMR (400 MHz, DMSO) *δ* 8.66 (s, 1H), 8.54 (d, *J* = 8.9 Hz, 1H), 8.11 (d, *J* = 2.6 Hz, 1H), 7.45 (dd, *J* = 9.1, 2.6 Hz, 1H), 2.43 (q, *J* = 7.5 Hz, 2H), 1.15 (t, *J* = 7.5 Hz, 2H), ^13 ^C NMR (101 MHz, MeOD) *δ* 173.64, 135.49, 131.91, 129.26, 128.03, 127.01, 121.90, 30.85, 8.63, HRMS (ESI/QTOF) *m/z*: calcd for [M + Na]^+^ C_11_H_11_ClN_4_ONa^+^ 273.0514; found, 273.0521.

*N*-(4-Chloro-2-(1*H*-1,2,4-triazol-3-yl)phenyl)butyramide (**148**): Synthesised from *N*-(4-chloro-2-cyanophenyl)butyramide (**130ac**) (222 mg, 1 mmol) according to the general procedure (V) to afford **148** (76 mg, 29%) as white solid, m.p. 156–158 °C. ^1^H NMR (400 MHz, MeOD) *δ* 8.59 (d, *J* = 8.9 Hz, 2H), 8.18 (s, 1H), 7.41 (dd, *J* = 9.0, 2.6 Hz, 1H), 2.48 (dd, *J* = 8.4, 6.6 Hz, 2H), 1.87 − 1.74 (m, 2H), 1.04 (dd, *J* = 8.4, 6.5 Hz, 3H), ^13 ^C NMR (101 MHz, MeOD) *δ* 172.85, 135.44, 129.25, 128.09, 127.04, 121.93, 39.78, 18.68, 12.59, HRMS (ESI/QTOF) *m/z*: calcd for [M + Na]^+^ C_12_H_13_ClN_4_ONa^+^ 287.0670; found, 287.0675.

*N*-(4-Chloro-2-(1*H*-1,2,4-triazol-3-yl)phenyl)cyclopropanecarboxamide (**149**): Synthesised from *N*-(4-chloro-2-cyanophenyl)cyclopropanecarboxamide (**130ad**) (220 mg, 1 mmol) according to the general procedure (**V**) to afford **149** (71 mg, 27%) as white solid, m.p. 183–186 °C. ^1^H NMR (400 MHz, DMSO) *δ* 8.60 (brs, 1H), 8.43 (d, *J* = 9.0 Hz, 1H), 8.08 (d, *J* = 2.6 Hz, 1H), 7.42 (dd, *J* = 8.9, 2.6 Hz, 1H), 1.72 (td, *J* = 7.4, 3.8 Hz, 1H), 0.89 (dt, *J* = 8.6, 3.0 Hz, 6H), ^13 ^C NMR (101 MHz, MeOD) *δ* 173.32, 143.18, 134.08, 135.61, 129.13, 127.87, 127.09, 121.82, 15.74, 7.02, HRMS (ESI/QTOF) *m/z*: calcd for [M + Na]^+^ C_12_H_11_ClN_4_ONa^+^ 285.0514; found, 285.0518.

*N*-(4-Chloro-2-(1*H*-1,2,4-triazol-3-yl)phenyl)cyclobutanecarboxamide (**150**): Synthesised from *N*-(4-chloro-2-cyanophenyl)cyclobutanecarboxamide (**130ae**) (234 mg, 1 mmol) according to the general procedure (**V**) to afford **150** (69 mg, 25%) as a white solid, m.p. 201–203 °C. ^1^H NMR (400 MHz, MeOD) *δ* 8.68 − 8.52 (m, 1H), 8.18 (d, *J* = 2.6 Hz, 1H), 7.41 (dd, *J* = 9.0, 2.6 Hz, 1H), 3.39 (qd, *J* = 8.7, 1.1 Hz, 1H), 2.50 2.27 (m, 4H), 2.18 − 2.00 (m, 1H), 1.99 − 1.88 (m, 1H), ^13 ^C NMR (101 MHz, MeOD) *δ* 174.81, 138.39, 128.01, 121.80, 41.39, 25.06, 17.41, HRMS (ESI/QTOF) *m/z*: calcd for [M + Na]^+^ C_13_H_13_ClN_4_ONa^+^ 299.0670; found, 299.0677.

*N*-(4-Chloro-2-(1*H*-1,2,4-triazol-3-yl)phenyl)cyclopentanecarboxamide (**151**): Synthesised from *N*-(4-chloro-2-cyanophenyl)cyclopentanecarboxamide (**130af**) (248 mg, 1 mmol) according to the general procedure (**V**) to afford **151** (96 mg, 33%) as white solid, m.p. 197–199 °C. ^1^H NMR (400 MHz, DMSO) *δ* 11.63 (brs, 1H), 8.84 (brs, 1H), 8.63 (d, *J* = 9.0 Hz, 1H), 8.14 (d, *J* = 2.6 Hz, 1H), 7.48 (dd, *J* = 8.9, 2.6 Hz, 1H), 2.85 (p, *J* = 8.1 Hz, 1H), 2.06 − 1.89 (m, 2H), 1.80 (dq, *J* = 11.4, 6.9 Hz, 2H), 1.74 − 1.61 (m, 2H), 1.68 − 1.54 (m, 2H), ^13 ^C NMR (101 MHz, MeOD) *δ* 175.97, 135.64, 129.28, 127.95, 127.03, 121.90, 29.99, 25.48, HRMS (ESI/QTOF) *m/z*: calcd for [M + Na]^+^ C_14_H_15_ClN_4_ONa^+^ 313.0827; found, 313.0825.

*N*-(2-(1*H*-1,2,4-triazol-3-yl)phenyl)benzamide (**152**, CAS [2428213–63-0]): Synthesised from *N*-(2-cyanophenyl)benzamide (**130bg**) (222 mg, 1 mmol) according to the general procedure (**V**) to afford **152** (92 mg, 35%) as a white solid. ^1^H NMR (400 MHz, MeOD) *δ* 8.81 (d, *J* = 8.4 Hz, 1H), 8.59 (s, 1H), 8.21 (s, 1H), 8.15 (dd, *J* = 8.1, 1.6 Hz, 2H), 7.70 − 7.54 (m, 3H), 7.55 − 7.44 (m, 1H), 7.27 (t, *J* = 7.6 Hz, 1H), ^13 ^C NMR (101 MHz, MeOD) *δ* 166.35, 137.00, 134.84, 131.72, 129.91, 128.47, 127.47, 127.19, 123.36, 120.51, HRMS (ESI/QTOF) *m/z*: calcd for [M + Na]^+^ C_15_H_12_N_4_ONa^+^ 287.0903; found, 287.0903.

*N*-(4-Chloro-2-(1*H*-1,2,4-triazol-3-yl)phenyl)benzamide (**153**): Synthesised from *N*-(4-chloro-2-cyanophenyl)benzamide (**130ag**) (256 mg, 1 mmol) according to the general procedure (**V**) to afford **153** (95 mg, 32%) as white solid, m.p. 213–216 °C. ^1^H NMR (400 MHz, MeOD) *δ* 8.85 (d, *J* = 9.0 Hz, 1H), 8.67 (s, 1H), 8.27 (s, 1H), 8.18 − 8.11 (m, 2H), 7.68 − 7.55 (m, 3H), 7.48 (dd, *J* = 9.0, 2.5 Hz, 1H), ^13 ^C NMR (101 MHz, MeOD) *δ* 166.29, 143.40, 134.54, 131.88, 129.30, 128.54, 128.30, 127.22, 121.84, HRMS (ESI/QTOF) *m/z*: calcd for [M + Na]^+^ C_15_H_11_ClN_4_ONa^+^ 321.0514; found, 321.0517.

*N*-(2-Chloro-6-(1*H*-1,2,4-triazol-3-yl)phenyl)benzamide (**154**): Synthesised from *N*-(2-chloro-6-cyanophenyl)-benzamide (**130cg**) (256 mg, 1 mmol) according to the general procedure (**V**) to afford **154** (101 mg, 34%) as amorphous solid. ^1^H NMR (400 MHz, CDCl_3_) *δ* 10.16 (s, 1H), 8.18 − 8.03 (m, 3H), 7.79 − 7.46 (m, 5H), 7.10 (t, *J* = 8.0 Hz, 1H), ^13 ^C NMR (101 MHz, CDCl_3_) *δ* 166.58, 133.46, 133.01, 132.90, 132.48, 131.58, 128.84, 127.89, 127.10, 127.00, HRMS (ESI/QTOF) *m/z*: calcd for [M + Na]^+^ C_15_H_11_ClN_4_ONa^+^ 321.0519; found, 321.0524.

4-(1*H*-1,2,4-Triazol-3-yl)pyridine (**158**)^81^: Synthesised from isonicotinonitrile (**130i**) (104 mg, 1 mmol) according to the general procedure (**V**) to afford **158** (59 mg, 40%) as white solid. ^1^H NMR (400 MHz, CDCl_3_) *δ* 8.71 − 8.58 (m, 1H), 8.15 (d, *J* = 6.8 Hz, 2H), 7.88 (tt, *J* = 7.8, 1.8 Hz, 1H), 7.40 (t, *J* = 6.3 Hz, 1H), ^13 ^C NMR (101 MHz, MeOD) *δ* 149.41, 137.37, 124.59, 121.31, HRMS (ESI/QTOF) *m/z*: calcd for [M + H]^+^ C_7_H_7_N_4_^+^ 147.0665; found, 147.0660.

2-(1*H*-1,2,4-Triazol-3-yl)pyridine (**159**)^81^: Synthesised from picolinonitrile (**130j**) (104 mg, 1 mmol) according to the general procedure (**V**) to afford **159** (61 mg, 41%) as white solid. ^1^H NMR (400 MHz, CDCl_3_) *δ* 8.71 − 8.62 (m, 2H), 8.29 (s, 1H), 8.03 − 7.93 (m, 2H), ^13 ^C NMR (101 MHz, MeOD) *δ* 170.82, 149.45, 145.46, 120.68, HRMS (ESI/QTOF) *m/z*: calcd for [M + H]^+^ C_7_H_7_N_4_^+^ 147.0665; found, 147.0665.

2-(1*H*-1,2,4-Triazol-3-yl)pyridine-3-amine (**160**, CAS [53511–60-7]): Synthesised from 3-aminopicolinonitrile (**130k**) (119 mg, 1 mmol) according to the general procedure (**V**) to afford **160** (64 mg, 40%) as white solid. ^1^H NMR (400 MHz, MeOD) *δ* 9.28 (s, 1H), 8.76 (s, 1H), 8.57 (brs, 1H), ^13 ^C NMR (101 MHz, MeOD) *δ* 148.91, 147.21, 140.28, HRMS (ESI/QTOF) *m/z*: calcd for [M + H]^+^ C_6_H_5_ClN_5_^+^ 182.0228; found, 182.0223.

2-(1*H*-1,2,4-Triazol-3-yl)thiophen-3-amine (**161**, CAS [1250372–48-5]): Synthesised from 3-aminothiophene-2-carbonitrile (**130l**) (124 mg, 1 mmol) according to the general procedure (**V**) to afford **161** (66 mg, 40%) as yellow solid. ^1^H NMR (400 MHz, MeOD) *δ* 8.70 (s, 1H), 7.66 (d, *J* = 5.4 Hz, 1H), 7.14 (d, *J* = 5.4 Hz, 1H), ^13 ^C NMR (101 MHz, MeOD) *δ* 154.71, 143.76, 129.26, 127.55, 123.03, 121.36, HRMS (ESI/QTOF) *m/z*: calcd for [M + H]^+^ C_6_H_7_N_4_S^+^ 167.0386; found, 167.0379.

2-Chloro-6-(1*H*-1,2,4-triazol-3-yl)pyrazine (**162**, CAS [1472615–55-6]): Synthesised from 6-chloropyrazine-2-carbonitrile (**130m**) (139 mg, 1 mmol) according to the general procedure (**V**) to afford **162** (76 mg, 42%) as white solid. ^1^H NMR (400 MHz, MeOD) *δ* 8.15 (s, 1H), 7.96 (dd, *J* = 4.3, 1.5 Hz, 1H), 7.28 − 7.13 (m, 2H), ^13 ^C NMR (101 MHz, MeOD) *δ* 143.31, 137.23, 125.05, 123.33, HRMS (ESI/QTOF) *m/z*: calcd for [M + H]^+^ C_7_H_8_N_5_^+^ 162.0774; found, 162.0770.

#### Synthesis of 3-Aryl-1H-1,2,4-Triazoles (132a–132c) and (155–157)

3–(3-Bromo-2-methyl-6-nitrophenyl)-1*H*-1,2,4-triazole (**132a**)^81^: A solution of 3-bromo-2-methyl-6-nitrobenzamide (**131a**) (387 mg, 1.5 mmol) and *N,N-*dimethylformamide-dimethyl acetal (DMF-DMA) (3.0 mL) was heated under reflux for 1 h under a nitrogen atmosphere. The mixture was cooled, concentrated under reduced pressure, and the residue was washed with hexane. After drying *in vacuo*, a white solid was obtained and used in the next step without purification. The crude product was dissolved in acetic acid (2 mL), and hydrazine hydrate (90 mg, 1.65 mmol) was added dropwise to the stirred solution. The mixture was stirred at 90 °C for 2 h. After cooling to rt, the solvent was removed under reduced pressure. The mixture was dissolved in diethyl ether (5 mL) and stirred at 0 °C for 30 min. The precipitate was collected, dried under reduced pressure, and purified by FC (SiO_2_, EtOAc/petroleum ether) to give **132a** (304 mg, 72%) as white solid. ^1^H NMR (400 MHz, MeOD) *δ* 8.70 (s, 1H), 7.99 (d, *J* = 8.3 Hz, 1H), 7.84 (m, 1H), 2.31 (s, 3H).

3–(3-Chloro-2-methyl-6-nitrophenyl)-1*H*-1,2,4-triazole (**132b**): Synthesised from 3-chloro-2-methyl-6-nitrobenzamide (**131b**) (214 mg, 1.0 mmol) according to the procedure used for **132a** to afford **132b** (167 mg, 70%) as white solid. ^1^H NMR (400 MHz, MeOD) *δ* 9.19 (s, 1H), 8.09 (d, *J* = 8.8 Hz, 1H), 7.91 (d, *J* = 8.8 Hz, 1H), 2.30 (s, 3H).

3–(3-Chloro-2,6-dinitrophenyl)-1*H*-1,2,4-triazole (**132c**): Synthesised from 3-chloro-2,6-dinitrobenzamide (**131c**) (244 mg, 1.0 mmol) according to the procedure used for **132a** to afford **132c** (214 mg, 80%) as white solid. ^1^H NMR (400 MHz, MeOD) *δ* 8.60 (s, 1H), 8.24 (d, *J* = 8.9 Hz, 1H), 8.06 (d, *J* = 8.8 Hz, 1H).

4-Bromo-3-methyl-2-(1*H*-1,2,4-triazol-3-yl)aniline (**155**)^82^: A mixture of **132a** (141 mg, 0.5 mmol) and SnCl_2_ ·2H_2_O (560 mg, 2.5 mmol) in absolute EtOH (3 mL) was heated to 70 °C under a nitrogen atmosphere. The progress of the reaction was monitored by TLC. After 1.5 h the starting material has disappeared. The solution was cooled to rt and poured into ice. The pH was adjusted to a slightly basic value (7–8) by addition of 5% NaHCO_3_ before extraction with EtOAc (3 × 5 mL). The collected organic phases were washed with brine, treated with charcoal and filtered. After drying over Na_2_SO_4_, the solvent was removed under reduced pressure and the residue was purified by FC (silica gel, EtOAc/hexanes) to give **155** (83 mg, 66%) as white solid, m.p. 201–204 °C. ^1^H NMR (400 MHz, MeOD) *δ* 8.45 (brs, 1H), 7.37 (d, *J* = 8.8 Hz, 1H), 6.63 (d, *J* = 8.6 Hz, 1H), ^13 ^C NMR (101 MHz, MeOD) δ 146.79, 137.07, 133.60, 114.57, 19.63, HRMS (ESI/QTOF) *m/z*: calcd for [M + H]+ C9H9BrN4 + 253.0089; found, 253.0092.

4-Chloro-3-methyl-2-(1*H*-1,2,4-triazol-3-yl)aniline (**156**): Prepared from **132b** (119 mg, 0.5 mmol) and SnCl_2_ ·2H_2_O (560 mg, 2.5 mmol) according to the procedure used for **155** to afford **156** (62 mg, 59%) as white solid, m.p. 208–210 °C. ^1^H NMR (400 MHz, MeOD) *δ* 8.46 (brs, 1H), 7.20 (d, *J* = 8.8 Hz, 1H), 6.69 (d, *J* = 8.7 Hz, 1H), ^13 ^C NMR (101 MHz, MeOD) *δ* 146.21, 130.54, 135.42, 114.22, 16.55. HRMS (ESI/QTOF) *m/z*: calcd for [M + H]^+^ C_9_H_9_ClN_4_^+^ 209.0594; found, 209.0597.

4-Chloro-2-(1*H*-1,2,4-triazol-3-yl)benzene-1,3-diamine (**157**): Prepared from **132c** (135 mg, 0.5 mmol) and SnCl_2_ ·H_2_O (560 mg, 2.5 mmol) according to the procedure used for **155** to afford **157** (63 mg, 60%) as brown solid, m.p. 212–214 °C. ^1^H NMR (400 MHz, MeOD) *δ* 7.96 (s, 1H), 7.46 (d, *J* = 8.9 Hz, 1H), 6.98 (d, *J* = 8.9 Hz, 1H).

#### Synthesis of aryl carbonitriles

5-Chloro-2-(methylamino)benzonitrile (**130an**)^121^: A mixture of 2-amino-5-chlorobenzonitrile (**127a**) (458 mg, 3.0 mmol), dimethyl oxalate (532 mg, 4.5 mmol) and *t*-BuOK (421 mg, 3.75 mmol) in DMA (6 mL) was heated under a nitrogen atmosphere to 130–140 °C for 5 h. The mixture was poured into ice water and extracted with EtOAc (3 × 10 mL). After concentration of the organic layers under reduced pressure, the crude product was purified by FC (silica gel, EtOAc/hexane) to afford **130an** (345 mg, 70%) as white solid. ^1^H NMR (400 MHz, CDCl_3_) *δ* 7.42 7.34 (m, 2H), 6.65 − 6.58 (m, 1H), 4.68 (brs, 1H), 2.94 (dd, *J* = 5.1, 1.8 Hz, 3H).

*N*-(4-Chloro-2-cyanophenyl)acetamide (**130aa**)^122^: To a stirred mixture of 2-amino-5-chlorobenzonitrile (**127a**) (456 mg, 3.0 mmol) in CH_2_Cl_2_ (6 mL), pyridine (260 mg, 3.3 mmol), DMAP (18 mg, 0.15 mmol), and acetyl chloride (**128a**) (351 mg, 4.5 mmol) were added. The reaction mixture was stirred at rt for 3 h, then diluted with CH_2_Cl_2_ (10 mL) and washed with H_2_O (2 × 5 mL) and brine (2 × 5 mL). The combined organic phases were dried over Na_2_SO_4_, filtered, and concentrated under reduced pressure. The residue was triturated with pentane and dried under air to afford **130aa** (436 mg, 75%) as an oil. ^1^H NMR (400 MHz, DMSO) *δ* 10.28 (bs, 1H), 7.97 (d, *J* = 2.5 Hz, 1H), 7.74 (dd, *J* = 8.8, 2.6 Hz, 1H), 7.58 (d, *J* = 8.8 Hz, 2H), 2.09 (s, 3H).

*N*-(4-Chloro-2-cyanophenyl)propionamide (**130ab**)^123^: Synthesised the same way as **130aa** from 2-amino-5-chlorobenzonitrile (**127a**) (456 mg, 3.0 mmol) and propionyl chloride (**128 b**) (304 mg, 3.3 mmol) to afford **130ab** (530 mg, 85%) as a colourless oil. ^1^H NMR (400 MHz, CDCl_3_) *δ* 8.51 − 8.43 (m, 1H), 7.61 − 7.53 (m, 2H), 2.52 (q, *J* = 7.5 Hz, 2H), 1.30 (td, *J* = 7.5, 1.1 Hz, 3H).

*N*-(4-Chloro-2-cyanophenyl)butyramide (**130ac**, CAS [53313–24-9]): Synthesised the same way as **130aa** from 2-amino-5-chlorobenzonitrile (**127a**) (456 mg, 3.0 mmol) and butyryl chloride (**128c**) (350 mg, 3.3 mmol) to afford **130ac** (566 mg, 85%) as a colourless oil. ^1^H NMR (400 MHz, CDCl_3_) *δ* 8.50 − 8.42 (m, 1H), 7.57 (dq, *J* = 4.6, 2.5 Hz, 2H), 2.46 (t, *J* = 7.4 Hz, 2H), 1.81 (h, *J* = 7.4 Hz, 2H), 1.06 (t, *J* = 7.4 Hz, 3H).

*N*-(4-Chloro-2-cyanophenyl)cyclopropanecarboxamide (**130ad**): Synthesised the same way as **130aa** from 2-amino-5-chlorobenzonitrile (**127a**) (456 mg, 3.0 mmol) and cyclopropanecarbonyl chloride (**128d**) (343 mg, 3.3 mmol) to afford **130ad** (528 mg, 80%) as a colourless oil. ^1^H NMR (400 MHz, CDCl_3_) *δ* 8.50 − 8.40 (m, 1H), 7.61 7.53 (m, 1H), 7.56 (bs, 1H), 7.50 (s, 1H), 3.22 (pd, *J* = 8.5, 1.1 Hz, 1H), 2.15 − 1.85 (m, 4H).

*N*-(4-Chloro-2-cyanophenyl)cyclobutanecarboxamide (**130ae**, CAS [2147559–95-1]): Synthesised the same way as **130aa** from 2-amino-5-chlorobenzonitrile (**127a**) (456 mg, 3.0 mmol) and cyclobutanecarbonyl chloride (**128e**) (390 mg, 3.3 mmol) to afford **130ae** (597 mg, 80%) as an oil. ^1^H NMR (400 MHz, CDCl_3_) *δ* 8.52 − 8.45 (m, 1H), 7.61 − 7.53 (m, 1H), 7.56 (s, 1H), 7.50 (s, 1H), 3.27 (pd, *J* = 8.5, 1.1 Hz, 1H), 2.50 − 2.25 (m, 4H), 2.18 − 1.91 (m, 2H).

*N*-(4-chloro-2-cyanophenyl)cyclopentanecarboxamide (**130af**, CAS [2242379–82-2]): Synthesised the same way as **130aa** from 2-amino-5-chlorobenzonitrile (**127a**) (456 mg, 3.0 mmol) and cyclopentanecarbonyl chloride (**128f**) (436 mg, 3.3 mmol) to afford **130af** (632 mg, 85%) as a colourless oil. ^1^H NMR (400 MHz, CDCl_3_) *δ* 8.52 − 8.45 (m, 1H), 7.61 − 7.53 (m, 1H), 7.56 (s, 1H), 7.50 (s, 1H), 3.27 (pd, *J* = 8.5, 1.1 Hz, 1H), 2.50 − 2.25 (m, 4H), 2.18 − 1.91 (m, 4H).

*N*-(4-chloro-2-cyanophenyl)benzamide (**130ag**)^124^: Synthesised the same way as **130aa** from 2-amino-5-chlorobenzonitrile (**127a**) (456 mg, 3.0 mmol) and benzoyl chloride (**128g**) (462 mg, 3.3 mmol) to afford **130ag** (600 mg, 78%) as an amorphous solid. ^1^H NMR (400 MHz, CDCl_3_) *δ* 8.64 (d, *J* = 8.8 Hz, 1H), 8.40 (s, 1H), 7.99 − 7.92 (m, 2H), 7.75 − 7.52 (m, 5H).

*N*-(2-cyanophenyl)benzamide (**130bg**)^125^: Synthesised the same way as **130aa** from 2-aminobenzonitrile (**127b**) (354 mg, 3.0 mmol) and benzoyl chloride (**128g**) (462 mg, 3.3 mmol) to afford **130bg** (566 mg, 85%) as an oil. ^1^H NMR (400 MHz, CDCl_3_) *δ* 8.65 (d, *J* = 8.5 Hz, 1H), 8.45 − 8.40 (m, 1H), 8.01 − 7.93 (m, 2H), 7.73 − 7.52 (m, 5H), 7.25 (td, *J* = 7.7, 1.1 Hz, 1H).

*N*-(2-chloro-6-cyanophenyl)-benzamide (**130cg**): Synthesised the same way as **130aa** from 2-amino-3-chloro-benzonitrile (**127c**) (456 mg, 3.0 mmol) and benzoyl chloride (**128g**) (462 mg, 3.3 mmol) to afford **130cg** (614 mg, 80%) as a colourless oil. ^1^H NMR (400 MHz, CDCl_3_) *δ* 8.04 − 7.97 (m, 2H), 7.93 (bs, 1H), 7.77–7.68 (m, 2H), 7.67 − 7.62 (m, 1H), 7.59–7.52 (m, 2H), 7.37 (t, *J* = 8.0 Hz, 1H).

#### Synthesis of aryl carboxamides

3-Bromo-2-methyl-6-nitrobenzamide (**131a**, CAS [110127–08-7]): A solution of 3-bromo-2-methylbenzoic acid (**129a**) (1.07 g, 5 mmol) in H_2_SO_4_ (98%, 1.1 mL) was treated with fuming nitric acid (1.1 mL) at rt. The reaction mixture was stirred for 3 h, cooled and poured into ice water (10 mL). The white precipitate was collected and dried. The aqueous filtrate was extracted with EtOAc (3 × 10 mL) and concentrated under reduced pressure. The residue was combined with the previously collected preciputate and used in the next step without further purification. To a slurry of crude compound in CH_2_Cl_2_ (20 mL) at 0 °C, oxalyl chloride (661 mg, 5.25 mmol) was added under a nitrogen atmosphere, followed by dropwise addition of DMF (2.5 mmol). The mixture was stirred 5 min at 0 °C, 15 min at rt, and then heated to reflux under N_2_ for 1 h. The mixture was cooled and poured into NH_4_OH (15 mL, ca. 30% NH_3_). Precipitated solids were collected by filtration and purified by FC to afford **131a** (903 mg, 70%) as a yellow oil. ^1^H NMR (400 MHz, MeOD) *δ* 7.96 (d, *J* = 8.8 Hz, 1H), 7.89 (d, *J* = 8.8 Hz, 1H), 2.55 (s, 3H).

3-Chloro-2-methyl-6-nitrobenzamide (**131b**, CAS [51123–60-5]): Synthesised the same way as **131a** from 3-chloro-2-methylbenzoic acid (**129b**) (850 mg, 5 mmol) to afford **131b** (760 mg, 71%) as a yellow oil. ^1^H NMR (400 MHz, MeOD) *δ* 8.06 (d, *J* = 8.8 Hz, 1H), 7.70 (d, *J* = 8.8 Hz, 1H), 2.51 (s, 3H).

3-Chloro-2–6-dinitrobenzamide (**131c**): Synthesised the same way as **131a** from 3-chloro-2-nitrobenzoic acid (**129c**) (1 g, 5 mmol) to afford **131c** (920 mg, 75%) as an oil. ^1^H NMR (400 MHz, MeOD) *δ* 8.38 (d, *J* = 8.9 Hz, 1H), 8.00 (d, *J* = 8.9 Hz, 1H).

#### Synthesis of other 1,2,4-Triazoles

*N*-(4-Bromophenyl)-4*H*-1,2,4-triazol-3-amine (**166**): 1-Bromo-4-iodobenzene (283 mg, 1.0 mmol) was dissolved in *N*,*N*-dimethylacetamide and 4*H*-1,2,4-triazol-3-amine (**163**) (168 mg, 2.0 mmol), K_2_CO_3_ (207 mg, 1.5 mmol), and CuI (19.1 mg, 0.1 mmol) were added under a nitrogen atmosphere. The mixture was stirred at 90 °C for 48 h. After cooling to rt, the solids were filtered and washed with CH_2_Cl_2_ (2 × 10 mL). The combined organic phases were concentrated under reduced pressure and the residue was purified by FC (silica gel, EtOAc/hexane) to give **166** (93 mg, 39%) as a white solid, m.p. 190–192 °C. ^1^H NMR (400 MHz, CDCl_3_) *δ* 7.88 (s, 1H), 7.73 (d, *J* = 8.4 Hz, 2H), 7.44 (d, *J* = 8.4 Hz, 2H), ^13 ^C NMR (101 MHz, MeOD) *δ* 148.90, 135.93, 132.49, 125.22, 121.28, HRMS (ESI/QTOF) *m/z*: calcd for [M + H]^+^ C_8_H_8_BrN_4_^+^ 238.9927; found, 238.9929.

2-((1,9a-Dihydrobenzo[4,5]thiazolo[2,3-c][1,2,4]triazol-3-yl)thio)acetamide(**168**): To the solution of (1,9a-dihydrobenzo[4,5]thiazolo[2,3-c][1,2,4]triazol-3-yl)thiol (**164**) (207 mg, 1.0 mmol) in acetone (5 mL), K_2_CO_3_ (138 mg, 1.0 mmol) and 2-chloroacetamide (**165**) (191 mg, 1.0 mmol) were added. The reaction mixture was heated under reflux for 9 h, then cooled to rt, filtered, and acidified with aq. 2 N HCl solution. The precipitate was filtered, washed with cold water (2 mL), and recrystallized from aqueous ethanol to afford **168** (160 mg, 60%) as white solid. ^1^H NMR (400 MHz, CDCl_3_) *δ* 7.92 − 7.77 (m, 2H), 7.10 (td, *J* = 8.5, 4.3 Hz, 2H), 6.91 − 6.73 (m, 2H), 4.54 (brs, 1H), ^13 ^C NMR (101 MHz, CDCl_3_) *δ* 154.81, 152.73, 132.28, 132.26, 127.32, 127.30, 124.37, 118.59, 118.56, 110.64, HRMS (ESI/QTOF) *m/z*: calcd for [M + Na]^+^ C_10_H_8_N_4_OS_2_Na^+^ 287.0032; found, 287.0037.

### Protein expression and purification

Full-length human IDO1 gene was cloned into the pET-28a vector and expressed in E. coli Rosetta (DE3) cells. Protein was produced in LB medium supplemented with 0.5 mM dALA (5-aminolevulinic acid) and was induced by addition of 0.1 mM isopropylthio-*β*-galactoside overnight at 16 °C. The culture was harvested and frozen at −80 °C until further use. The frozen cells were lysed by sonication in 20 mM Tris pH 7, 400 mM NaCl, 1 mM MgCl_2_, 20 mM imidazole, 1 mM DTT, 0.5 mg/mL Lysozyme and Protease inhibitor. After centrifugation, the supernatant was loaded into a HisTrap HP column pre-equilibrated with buffer containing 20 mM Tris pH 7, 500 mM NaCl and 20 mM imidazole, and eluted with the same buffer containing 500 mM imidazole. Fractions containing IDO1 were pooled, concentrated, and injected on a size exclusion chromatography column (HiLoad Superdex S75 16/60) to increase purity. hIDO1 fractions were concentrated to 20 mg/mL in 20 mM Tris pH 7 and 150 mM NaCl.

### Protein crystallisation, data collection, structure determination and model refinement

hIDO1 protein at 20 mg/mL was mixed with compound MMG-0472 at a final concentration of 5 mM, and co-crystallized by sitting drop vapour diffusion method. Crystals formed in a couple of days in 15% PEG 4000, 0.2 M lithium sulphate, and 0.1 M Tris pH 7.5. The crystals were cryoprotected with 25% glycerol. Diffraction data were collected at the Paul Scherrer Institute (SLS, Villigen) at PXII–X06DA beamline. Data were processed with the XDS Program Package^126^. Structures were solved by molecular replacement using Phaser-MR and chain A of PDB entry 2d0t^40^ as the model. Manual model building and structure refinement were carried out in Phenix Suite^127^ using coot software^128^ and phenix-refine, respectively. After validation, the ligand bound IDO1 model was deposited in the PDB database under PDB code 7zv3. Data collection and refinement statistics are summarised in the Supplementary Information (Table S1). The structures were displayed with PyMOL (http://www.pymol.org/) and UCSF Chimera^129^. Authors will release the atomic coordinates and experimental data upon article publication.

### Enzymatic assays

The enzymatic inhibition assays were performed as described by Takikawa et al.^130^ with some modifications. Briefly, the reaction mixture (100 *µ*L) contained potassium phosphate buffer (100 mM, pH 6.5) ascorbic acid (20 mM), catalase (400 units/mL), methylene blue (10 *µ*M), purified recombinant IDO1 (2.5 ng/*µ*L), L-Trp (100 *µ*M), Triton X-100 (0.01%) and DMSO (5 *µ*L). The inhibitors were serially diluted 3-fold for at least 8 different concentrations. After incubation at room temperature for 20 min, the reaction was stopped in its linear phase by addition of trichloroacetic acid solution (40 mL, 30% w/v), and the samples were incubated at 50 °C for 30 min. After centrifugation, 80 *µ*l of supernatant from each well were used for HPLC analysis. The mobile phase for HPLC analysis was composed of 50% (v/v) of methanol and 50% of sodium citrate buffer (40 mM, pH 2.4) containing 400 *µ*M sodium dodecyl sulphate. An Agilent Zorbax Eclipse XDB C18 column (150 × 4.6 mm) was used at 23 °C with a flow rate of 1 mL/min and an injection volume of 20 *µ*L. For detection of kynurenine, absorption at 365 nm was measured, while remaining L-Trp was detected at a wavelength of 280 nm. All assays were carried out in duplicates. The activity (Act) of each compound was calculated as *Act* = *RT* log(*IC*_50_) in analogy to the relation between the binding free energy and the K*_i_* value. The ligand efficiency (LE) was calculated as *LE* = −*Act/N_HA_*, where *N_HA_* is the number of non-hydrogen atoms (heavy atoms) in the compound.

### Cellular assays

The cDNA encoding hIDO1 (NCBI, NM 0021464.3) was purchased from Origene as pCMV6-Entry vector (RC206592). The full ORF of hIDO1 was cloned into the mammalian expression vector pcDNA6/myc-His from Invitrogen. Human embryonic kidney 293 T (HEK 293 T) cells were transiently transfected with the plasmid construct encoding hIDO1. A 75 cm^2^ flask containing HEK 293 T cells at a confluency of 70% was transfected with jetPEI DNA transfection reagent (Polyplus-transfection), according to manufacturer’s protocol. A total of 20 *µ*g of pcDNA-IDO1 along with 5 *µ*g of pcDNA-GFP plasmid were used for the transfection; GFP was used to evaluate transfection efficiency prior to the cellular assays. 16–18 h post-transfection the 293 T cells were detached with trypsin, centrifuged, and re-suspended in 25 mL of DMEM medium without phenol red, supplemented with penicillin and streptomycin, 10% foetal bovine serum and 1 mM pyruvate (Gibco). Subsequently, 100 *µ*L of cells were distributed to each well of two 96-well round-bottom plates that had been pre-loaded with 100 *µ*L DMEM and the small organic test molecule in DMSO (for a final DMSO concentration of 2%). DMEM medium contains 78 *µ*M L-Trp and was supplemented with 500 *µ*M of L-Trp. Cells transfected with hIDO1 were incubated for 7 h at 37 °C in a CO_2_ incubator. The reaction was stopped in its linear phase by adding trichloroacetic acid (TCA) to the medium at a final concentration of 4%. Plates were centrifuged for 15 min at 4,000 rpm. 100 *µ*L of supernatant was added to 100 *µ*L of 2% (w/v) DMAB in glacial acetic acid. After 10 min the absorbance was measured at a wavelength of 480 nm to detect kynurenine formation^85^. Alternatively, kynurenine content was determined by HPLC as done for the enzymatic assay.

### Cell viability

Following DMSO (negative control) or inhibitor treatments, apoptotic cells were detected by 4′,6-diamidino-2-phenylindole (DAPI) staining. Briefly, after 7 h of incubation, cells were washed with PBS, resuspended in PBS with 1 *µ*g/mL DAPI and immediately analysed using a BD LSR II cytometer. A 405 nm laser with 450/50 nm bandpass filter was used to collect data.

## Supplementary Material

Supplemental Material

## Supporting information

X-ray data collection and refinement statistics, previously determined cellular inhibition and toxicity data, binding poses of 5-substituted 4-aryl-1,2,3-triazoles, enzymatic dose-response curves of all compounds, HPLC purity traces, NMR spectra.

## Accession codes

Compound MMG-0472, PDB ID 7zv3. Authors will release the atomic coordinates upon article publication.

## References

[1] Kraehenbuehl L, Weng C-H, Eghbali S, et al. Enhancing immunotherapy in cancer by targeting emerging immunomodulatory pathways. Nat Rev Clin Oncol 2022;19:37–50. 34580473 10.1038/s41571-021-00552-7

[2] Uyttenhove C, Pilotte L, Théate I, et al. Evidence for a tumoral immune resistance mechanism based on tryptophan degradation by indoleamine 2,3-dioxygenase. Nat Med 2003;9:1269–74. 14502282 10.1038/nm934

[3] Platten M, Nollen EAA, Röhrig UF, et al. Tryptophan metabolism as a common therapeutic target in cancer, neurodegeneration and beyond. Nat Rev Drug Discov 2019;18:379–401. 30760888 10.1038/s41573-019-0016-5

[4] Mondanelli G, Mandarano M, Belladonna ML, et al. Current challenges for IDO2 as target in cancer immunotherapy. Front Immunol 2021;12:679953. 33968089 10.3389/fimmu.2021.679953 PMC8097162

[5] Yuasa HJ, Stocker R. Methylene blue and ascorbate interfere with the accurate determination of the kinetic properties of IDO2. Febs J 2021;288:4892–904. 33686747 10.1111/febs.15806

[6] Munn DH, Mellor AL. Indoleamine 2,3-dioxygenase and tumor-induced tolerance. J Clin Invest 2007;117:1147–54. 17476344 10.1172/JCI31178 PMC1857253

[7] Spranger S, Spaapen RM, Zha Y, et al. Up-regulation of PD-L1, IDO, and Tregs in the melanoma tumor microenvironment is driven by CD^8+^ T cells. Sci Transl Med 2013;5:200–ra116. 10.1126/scitranslmed.3006504 PMC4136707 23986400

[8] Spranger S, Koblish HK, Horton B, et al. Mechanism of tumor rejection with doublets of CTLA-4, PD-1/PD-L1, or IDO blockade involves restored IL-2 production and proliferation of CD8(+) T cells directly within the tumor microenvironment. J Immunother Cancer 2014;2:3. 24829760 10.1186/2051-1426-2-3 PMC4019906

[9] Long GV, Dummer R, Hamid O, et al. Epacadostat plus pembrolizumab versus placebo plus pembrolizumab in patients with unresectable or metastatic melanoma (ECHO-301/KEYNOTE-252): a phase 3, randomised, double-blind study. Lancet Oncol 2019;20:1083–97. 31221619 10.1016/S1470-2045(19)30274-8

[10] Muller AJ, Manfredi MG, Zakharia Y, Prendergast GC. Inhibiting IDO pathways to treat cancer: lessons from the ECHO-301 trial and beyond. Semin Immunopathol 2019;41:41–8. 30203227 10.1007/s00281-018-0702-0

[11] Van den Eynde BJ, van Baren N, Baurain J-f. Is there a clinical future for IDO1 inhibitors after the failure of epacadostat in Melanoma? Annu Rev Cancer Biol 2020;4:241–256.

[12] Pallotta MT, Orabona C, Volpi C, et al. Indoleamine 2,3-dioxygenase is a signaling protein in long-term tolerance by dendritic cells. Nat Immunol 2011;12:870–8. 21804557 10.1038/ni.2077

[13] Albini E, Rosini V, Gargaro M, et al. Distinct roles of immunoreceptor tyrosine-based motifs in immunosuppressive indoleamine 2,3-dioxygenase 1. J Cell Mol Med 2017;21:165–76. 27696702 10.1111/jcmm.12954 PMC5192792

[14] Albini E, Coletti A, Greco F, et al. Identification of a 2-propanol analogue modulating the nonenzymatic function of indoleamine 2,3-dioxygenase 1. Biochem Pharmacol 2018;158:286–97. 30391205 10.1016/j.bcp.2018.10.033

[15] Nelp MT, Kates PA, Hunt JT, et al. Immune-modulating enzyme indoleamine 2,3-dioxygenase is effectively inhibited by targeting its apo-form. Proc Natl Acad Sci USA 2018;115:3249–54. 29531094 10.1073/pnas.1719190115 PMC5879690

[16] Lim YJ, Foo TC, Yeung AWS, et al. Human indoleamine 2,3-dioxygenase 1 is an efficient Mammalian nitrite reductase. Biochemistry 2019;58:974–86. 30585477 10.1021/acs.biochem.8b01231

[17] Stanley CP, Maghzal GJ, Ayer A, et al. Singlet molecular oxygen regulates vascular tone and blood pressure in inflammation. Nature 2019;566:548–52. 30760924 10.1038/s41586-019-0947-3

[18] Nelp MT, Zheng V, Davis KM, et al. Potent activation of indoleamine 2,3-dioxygenase by polysulfides. J Am Chem Soc 2019;141:15288–300. 31436417 10.1021/jacs.9b07338

[19] Feng X, Liao D, Liu D, et al. Development of indoleamine 2,3-dioxygenase 1 inhibitors for cancer therapy and beyond: a recent perspective. J Med Chem 2020;63:15115–39. 33215494 10.1021/acs.jmedchem.0c00925

[20] Tang K, Wu Y-H, Song Y, Yu B. Indoleamine 2,3-dioxygenase 1 (IDO1) inhibitors in clinical trials for cancer immunotherapy. J Hematol Oncol 2021;14:68. 33883013 10.1186/s13045-021-01080-8 PMC8061021

[21] wwPDB c. Protein Data Bank: the single global archive for 3D macromolecular structure data. Nucleic Acids Res 2019;47:D520–D528. 30357364 10.1093/nar/gky949 PMC6324056

[22] Röhrig UF, Reynaud A, Majjigapu SR, et al. Inhibition mechanisms of indoleamine 2,3-Dioxygenase 1 (IDO1). J Med Chem 2019;62:8784–95. 31525930 10.1021/acs.jmedchem.9b00942

[23] Yue EW, Sparks R, Polam P, et al. INCB24360 (Epacadostat), a highly potent and selective indoleamine-2,3dioxygenase 1 (IDO1) inhibitor for immuno-oncology. ACS Med Chem Lett 2017;8:486–91. 28523098 10.1021/acsmedchemlett.6b00391 PMC5430407

[24] Kumar S, Waldo JP, Jaipuri FA, et al. Discovery of clinical candidate (1R,4r)-4-((R)-2-((S)-6-Fluoro-5H-imidazo[5,1-a]isoindol-5-yl)-1-hydroxyethyl)cyclohexan-1-ol (Navoximod), a potent and selective inhibitor of indoleamine 2,3-dioxygenase 1. J Med Chem 2019;62:6705–33. 31264862 10.1021/acs.jmedchem.9b00662

[25] Crosignani S, Bingham P, Bottemanne P, et al. Discovery of a Novel and selective indoleamine 2,3-dioxygenase (IDO-1) inhibitor 3-(5-Fluoro-1 H-indol-3-yl)pyrrolidine-2,5-dione (EOS200271/PF-06840003) and its characterization as a potential clinical candidate. J Med Chem 2017;60:9617–29. 29111717 10.1021/acs.jmedchem.7b00974

[26] Balog A, Lin T-a, Maley D, et al. Preclinical characterization of linrodostat mesylate, a Novel, potent, and selective oral indoleamine 2,3-dioxygenase 1 inhibitor. Mol Cancer Ther 2021;20:467–76. 33298590 10.1158/1535-7163.MCT-20-0251

[27] Röhrig UF, Michielin O, Zoete V. Structure and plasticity of indoleamine 2,3-dioxygenase 1 (IDO1). J Med Chem 2021;64:17690–705. 34907770 10.1021/acs.jmedchem.1c01665

[28] Röhrig UF, Majjigapu SR, Reynaud A, et al. Azole-based indoleamine 2,3-dioxygenase 1 (IDO1) inhibitors. J Med Chem 2021;64:2205–27. 33557523 10.1021/acs.jmedchem.0c01968

[29] Röhrig UF, Awad L, Grosdidier A, et al. Rational design of indoleamine 2,3-dioxygenase inhibitors. J Med Chem 2010;53:1172–89. 20055453 10.1021/jm9014718

[30] Yue EW, Douty B, Wayland B, et al. Discovery of potent competitive inhibitors of indoleamine 2,3-dioxygenase with *in vivo* pharmacodynamic activity and efficacy in a mouse melanoma model. J Med Chem 2009;52:7364–7. 19507862 10.1021/jm900518f

[31] Paul S, Roy A, Deka SJ, et al. Nitrobenzofurazan derivatives of N′-hydroxyamidines as potent inhibitors of indoleamine-2,3dioxygenase 1. Eur J Med Chem 2016;121:364–75. 27267006 10.1016/j.ejmech.2016.05.061

[32] Zhang H, Liu K, Pu Q, et al. Discovery of amino-cyclobutarene-derived indoleamine2,3-dioxygenase 1 (IDO1) inhibitors for cancer immunotherapy. ACS Med Chem Lett 2019;10:1530–6. 31749906 10.1021/acsmedchemlett.9b00344 PMC6862341

[33] Du Q, Feng X, Wang Y, et al. Discovery of phosphonamidate IDO1 inhibitors for the treatment of non-small cell lung cancer. Eur J Med Chem 2019;182:111629. 31445231 10.1016/j.ejmech.2019.111629

[34] Chen S, Guo W, Liu X, et al. Design, synthesis and antitumor study of a series of N-Cyclic sulfamoylaminoethyl substituted 1,2,5-oxadiazol-3-amines as new indoleamine 2, 3-dioxygenase 1 (IDO1) inhibitors. Eur J Med Chem 2019;179:38–55. 31233921 10.1016/j.ejmech.2019.06.037

[35] Liu C, Nan Y, Xia Z, et al. Discovery of novel hydroxyamidine derivatives as indoleamine 2,3-dioxygenase 1 inhibitors with *in vivo* anti-tumor efficacy. Bioorg Med Chem Lett 2020;30:127038. 32088128 10.1016/j.bmcl.2020.127038

[36] Steeneck C, Kinzel O, Anderhub S, et al. Discovery of hydroxyamidine based inhibitors of IDO1 for cancer immunotherapy with reduced potential for glucuronidation. ACS Med Chem Lett 2020;11:179–87. 32071686 10.1021/acsmedchemlett.9b00572 PMC7025390

[37] Song X, Sun P, Wang J, et al. Design, synthesis, and biological evaluation of 1,2,5-oxadiazole-3-carboximidamide derivatives as Novel indoleamine-2,3-dioxygenase 1 inhibitors. Eur J Med Chem 2020;189:112059. 31981851 10.1016/j.ejmech.2020.112059

[38] Jin F, Hu Q, Fei H, et al. Discovery of hydroxyamidine derivatives as highly potent, selective indoleamine-2,3-dioxygenase 1 Inhibitors. ACS Med Chem Lett 2021;12:195–201. 33603965 10.1021/acsmedchemlett.0c00443 PMC7883375

[39] Sono M, Cady SG. Enzyme kinetic and spectroscopic studies of inhibitor and effector interactions with indoleamine 2,3-dioxygenase. 1. Norharman and 4-phenylimidazole binding to the enzyme as inhibitors and heme ligands. Biochemistry 1989;28:5392–9. 2789076 10.1021/bi00439a012

[40] Sugimoto H, Oda S-i, Otsuki T, et al. Crystal structure of human indoleamine 2,3-dioxygenase: catalytic mechanism of O2 incorporation by a heme-containing dioxygenase. Proc Natl Acad Sci USA 2006;103:2611–6. 16477023 10.1073/pnas.0508996103 PMC1413787

[41] Kumar S, Jaller D, Patel B, et al. Structure based development of phenylimidazole-derived inhibitors of indoleamine 2,3-dioxygenase. J Med Chem 2008;51:4968–77. 18665584 10.1021/jm800512z PMC3159384

[42] Fallarini S, Massarotti A, Gesù A, et al. In silico-driven multicomponent synthesis of 4,5- and 1,5-disubstituted imidazoles as indoleamine 2,3-dioxygenase inhibitors. MedChemComm 2016;7:409–19.

[43] Brant MG, Goodwin-Tindall J, Stover KR, et al. Identification of potent indoleamine 2,3-dioxygenase 1 (IDO1) inhibitors based on a phenylimidazole scaffold. ACS Med Chem Lett 2018;9:131–6. 29456801 10.1021/acsmedchemlett.7b00488 PMC5807880

[44] Zheng Y, Stafford PM, Stover KR, et al. A series of 2-((1-Phenyl-1H-imidazol-5-yl)methyl)-1H-indoles as Indoleamine 2,3-Dioxygenase 1 (IDO1) Inhibitors. ChemMedChem 2021;16:2195–205. 33759400 10.1002/cmdc.202100107

[45] Peng Y-H, Ueng S-H, Tseng C-T, et al. Important hydrogen bond networks in indoleamine 2,3-Dioxygenase 1 (IDO1) inhibitor design revealed by crystal structures of imidazoleisoindole derivatives with IDO1. J Med Chem 2016;59:282–93. 26642377 10.1021/acs.jmedchem.5b01390

[46] Zou Y, Wang F, Wang Y, et al. Discovery of imidazoleisoindole derivatives as potent IDO1 inhibitors: design, synthesis, biological evaluation and computational studies. Eur J Med Chem 2017;140:293–304. 28963992 10.1016/j.ejmech.2017.09.025

[47] Tu W, Yang F, Xu G, et al. Discovery of imidazoisoindole derivatives as highly potent and orally active indoleamine-2,3-dioxygenase Inhibitors. ACS Med Chem Lett 2019;10:949–53. 31223453 10.1021/acsmedchemlett.9b00114 PMC6580535

[48] Parr BT, Pastor R, Sellers BD, et al. Implementation of the CYP index for the design of selective tryptophan-2,3-dioxygenase inhibitors. ACS Med Chem Lett 2020;11:541–9. 32292562 10.1021/acsmedchemlett.0c00004 PMC7153281

[49] Crescenzi C, Fuchss T, Ippoliti D, et al. Reiterative chiral resolution/racemization/recycle (RRR synthesis) for an effective and scalable process for the enantioselective synthesis of a dual IDO1/TDO2 inhibitor imidazoisoindole derivative. Org Process Res Dev 2020;24:1018–23.

[50] Tojo S, Kohno T, Tanaka T, et al. Crystal structures and structure–activity relationships of imidazothiazole derivatives as IDO1 inhibitors. ACS Med Chem Lett 2014;5:1119–23. 25313323 10.1021/ml500247w PMC4190630

[51] Peng Y-h, Liao F-y, Tseng C-t, et al. Unique sulfur–aromatic interactions contribute to the binding of potent imidazothiazole indoleamine 2,3-dioxygenase inhibitors. J Med Chem 2020;63:1642–59. 31961685 10.1021/acs.jmedchem.9b01549

[52] Griglio A, Torre E, Serafini M, et al. A multicomponent approach in the discovery of indoleamine 2,3-dioxygenase 1 inhibitors: synthesis, biological investigation and docking studies. Bioorg Med Chem Lett 2018;28:651–7. 29398544 10.1016/j.bmcl.2018.01.032

[53] Serafini M, Torre E, Aprile S, et al. Synthesis, docking and biological evaluation of a novel class of imidazothiazoles as IDO1 inhibitors. Molecules 2019;24:1874. 10.3390/molecules24101874 PMC6572114 31096672

[54] Cowley P, Wise A, Pharmaceutical compound. 2016; Patent WO 2016/071293.

[55] Qian S, He T, Wang W, et al. Discovery and preliminary structure–activity relationship of 1H-indazoles with promising indoleamine-2,3-dioxygenase 1 (IDO1) inhibition properties. Bioorg Med Chem 2016;24:6194–205. 27769672 10.1016/j.bmc.2016.10.003

[56] Yang L, Chen Y, He J, et al. 4,6-Substituted-1H-indazoles as potent IDO1/TDO dual inhibitors. Bioorg Med Chem 2019;27:1087–98. 30773421 10.1016/j.bmc.2019.02.014

[57] Ning X-L, Li Y-Z, Huo C, et al. X-ray structure-guided discovery of a potent, orally bioavailable, dual human indoleamine/tryptophan 2,3-dioxygenase (hIDO/hTDO) inhibitor that shows activity in a mouse model of Parkinson’s Disease. J Med Chem 2021;64:8303–32. 34110158 10.1021/acs.jmedchem.1c00303

[58] Röhrig UF, Majjigapu SR, Grosdidier A, et al. Rational design of 4-aryl-1,2,3-triazoles for indoleamine 2,3-dioxygenase 1 inhibition. J Med Chem 2012;55:5270–90. 22616902 10.1021/jm300260v

[59] Panda S, Pradhan N, Chatterjee S, et al. Manna, D. 4,5-disubstituted 1,2,3-triazoles: effective inhibition of indoleamine 2,3-dioxygenase 1 enzyme regulates t cell activity and mitigates tumor growth. Sci Rep 2019;9:18455. 31804586 10.1038/s41598-019-54963-9 PMC6895048

[60] Alexandre JAC, Swan MK, Latchem MJ, et al. New 4-Amino-1,2,3-triazole inhibitors of indoleamine 2,3-dioxygenase form a long-lived complex with the enzyme and display exquisite cellular potency. ChemBioChem 2018;19:552–61. 29240291 10.1002/cbic.201700560

[61] Röhrig UF, Majjigapu SR, Chambon M, et al. Detailed analysis and follow-up studies of a high-throughput screening for indoleamine 2,3-dioxygenase 1 (IDO1) inhibitors. Eur J Med Chem 2014;84:284–301. 25036789 10.1016/j.ejmech.2014.06.078

[62] Röhrig UF, Majjigapu SR, Caldelari D, et al. Michielin, O. 1,2,3-triazoles as inhibitors of indoleamine 2,3-dioxygenase 2 (IDO2). Bioorg Med Chem Lett 2016;26:4330–3. 27469130 10.1016/j.bmcl.2016.07.031

[63] Jin T, Kamijo S, Yamamoto Y. Copper-catalyzed synthesis of N-unsubstituted 1,2,3-triazoles from nonactivated terminal alkynes. Eur J Org Chem 2004;2004:3789–91.

[64] Arcadi A, Cacchi S, Del Rosario M, et al. Palladium catalyzed reaction of o-ethynylphenols, o-((trimethylsilyl)ethynyl)phenyl acetates, and o-alkynylphenols with unsaturated triflates or halides: a route to 2-substituted-, 2,3-disubstituted-, and 2-substituted-3-acylbenzo[b]furans. J Org Chem 1996;61:9280–8.

[65] Cheng Z-y, Li W-j, He F, et al. Synthesis and biological evaluation of 4-aryl-5-cyano-2H-1,2,3-triazoles as inhibitor of HER2 tyrosine kinase. Bioorg Med Chem 2007;15:1533–8. 17174554 10.1016/j.bmc.2006.09.041

[66] Quan X; Ren Z-h, Wang Y-y, Guan Z-h. p-toluenesulfonic acid mediated 1,3-dipolar cycloaddition of nitroolefins with NaN_3_ for synthesis of 4Aryl-NH-1,2,3-triazoles. Org Lett 2014;16:5728–31. 25343314 10.1021/ol5027975

[67] Panda S, Maity P, Manna D. Transition metal, azide, and oxidant-free homo- and heterocoupling of ambiphilic tosylhydrazones to the regioselective triazoles and pyrazoles. Org Lett 2017;19:1534–7. 28339210 10.1021/acs.orglett.7b00313

[68] Lai Q, Liu Q, He Y, et al. Triazoleimidazole (TA-IM) derivatives as ultrafast fluorescent probes for selective Ag + detection. Org Biomol Chem 2018;16:7801–5. 30328458 10.1039/c8ob02482k PMC6493330

[69] Ponpandian T, Muthusubramanian S. Tandem Knoevenagel-[3 + 2] cycloaddition-elimination reactions: one-pot synthesis of 4,5-disubstituted 1,2,3-(NH)-triazoles. Tetrahedron Lett 2012;53:59–63.

[70] Boyall D, Davis C, Dodd J, et al. Compounds useful as inhibitors of indoleamine 2,3-dioxygenase. 2014; Patent WO 2014/081689.

[71] Boyall D, Davis C, Dodd J, et al. Compounds useful as inhibitors of indoleamine 2,3-dioxygenase, WO/2014/081689. 2014.

[72] Zoller T, Uguen D, De Clan A, Flscher J. Efficient preparation of E-*β-*iodovinyl phenylsulfone by Finkelstein reaction at a vinylic center. Tetrahedron Lett 1998;39:8089–92.

[73] Tsai AS, Brasse M, Bergman RG, Ellman JA. Rh(III)-catalyzed oxidative coupling of unactivated alkenes via C–H activation. Org Lett 2011;13:540–2. 21175143 10.1021/ol102890k PMC3031716

[74] Tomé AC, Science of synthesis, 13: category 2, hetarenes and related ring systems. Stuttgart, New York, Delhi, Rio: Thieme Verlagsgruppe; 2004:415–601.

[75] Liu Y-L, Xu X-H, Qing F-L. Regioselective dehydroxytrifluoromethylthiolation of allylic and propargylic alcohols with AgSCF_3_. Tetrahedron Lett 2019;60:953–6.

[76] Koniev O, Leriche G, Nothisen M, et al. Selective irreversible chemical tagging of cysteine with 3-arylpropiolonitriles. Bioconjug Chem 2014;25:202–6. 24410136 10.1021/bc400469d

[77] Pauli L, Tannert R, Scheil R, Pfaltz A. Asymmetric hydrogenation of furans and benzofurans with iridium-pyridine-phosphinite catalysts. Chem- A Eur J 2015;21:1482–7. 10.1002/chem.201404903 25394881

[78] Lanni TB, Greene KL, Kolz CN, et al. Design and synthesis of phenethyl benzo[1,4]oxazine-3-ones as potent inhibitors of PI3Kinase*γ*. Bioorg Med Chem Lett 2007;17:756–60. 17095227 10.1016/j.bmcl.2006.10.080

[79] Cox RJ, Ritson DJ, Dane TA, et al. Room temperature palladium catalysed coupling of acyl chlorides with terminal alkynes. Chem Commun 2005;1037–9. 10.1039/b414826f 15719108

[80] Shechter S, Kauffmann M, Sandanyaka VP, Shacham S, Nuclear transport modulators and uses thereof, WO/2011/109799. 2011.

[81] Sun X, Hong Z, Liu M, et al. Design, synthesis, and biological activity of novel tetrahydropyrazolopyridone derivatives as FXa inhibitors with potent anticoagulant activity. Bioorg Med Chem 2017;25:2800–10. 28389110 10.1016/j.bmc.2017.03.055

[82] Bellamy F, Ou K. Selective reduction of aromatic nitro compounds with stannous chloride in non acidic and non aqueous medium. Tetrahedron Lett 1984;25:839–42.

[83] Panigrahi R, Panda S, Behera PK, et al. Recyclable bimetallic CuMoO4 nanoparticles for C–N cross-coupling reaction under mild conditions. New J Chem 2019;43:19274–8.

[84] Aboelmagd A, Ali IA, Salem EM, Abdel-Razik M. Synthesis and antifungal activity of some S-mercaptotriazolobenzothiazolyl amino acid derivatives. Eur J Med Chem 2013;60:503–11. 23376218 10.1016/j.ejmech.2012.10.033

[85] Littlejohn TK, Takikawa O, Skylas D, et al. Expression and purification of recombinant human indoleamine 2,3-dioxygenase. Protein Expr Purif 2000;19:22–9. 10833386 10.1006/prep.2000.1214

[86] Boyall D, Davis C, Dodd J, et al. Compounds useful as inhibitors of indoleamine 2,3-dioxygenase. 2015; Patent US 2015/0336903.

[87] Zoete V, Schuepbach T, Bovigny C, et al. Attracting cavities for docking: replacing the rough energy landscape of the protein by a smooth attracting landscape. J Comput Chem 2016;37:437–47. 26558715 10.1002/jcc.24249 PMC4738475

[88] MacKerell AD, Bashford D, Bellott M, et al. All-atom empirical potential for molecular modeling and dynamics studies of proteins. J Phys Chem B 1998;102:3586–616. 24889800 10.1021/jp973084f

[89] Mackerell AD, Feig M, Brooks CL. Extending the treatment of backbone energetics in protein force fields: limitations of gas-phase quantum mechanics in reproducing protein conformational distributions in molecular dynamics simulations. J Comput Chem 2004;25:1400–15. 15185334 10.1002/jcc.20065

[90] Haberthür U, Caflisch A. FACTS: fast analytical continuum treatment of solvation. J Comput Chem 2008;29:701–15. 17918282 10.1002/jcc.20832

[91] Zoete V, Grosdidier A, Cuendet M, Michielin O. Use of the FACTS solvation model for protein-ligand docking calculations: application to EADock. J Mol Recognit 2010;23:457–61. 20101644 10.1002/jmr.1012

[92] Zoete V, Cuendet MA, Grosdidier A, Michielin O. SwissParam: a fast force field generation tool for small organic molecules. J Comput Chem 2011;32:2359–68. 21541964 10.1002/jcc.21816

[93] Röhrig UF, Grosdidier A, Zoete V, Michielin O. Docking to heme proteins. J Comput Chem 2009;30:2305–15. 19288474 10.1002/jcc.21244

[94] Luo S, Xu K, Xiang S, et al. High-resolution structures of inhibitor complexes of human indoleamine 2,3dioxygenase 1 in a new crystal form. Acta Crystallogr Sect F Struct Biol Commun 2018;74:717–24. 30387777 10.1107/S2053230X18012955 PMC6213978

[95] Adamo C, Cossi M, Barone V. An accurate density functional method for the study of magnetic properties: the PBE0 model. J Mol Struct Theochem 1999;493:145–57.

[96] Frisch MJ, Trucks GW, Schlegel HB, et al. Gaussian 16 Revision C.01. 2016; Gaussian Inc. Wallingford CT.

[97] Schäfer A, Huber C, Ahlrichs R. Fully optimized contracted Gaussian basis sets of triple zeta valence quality for atoms Li to Kr. J Chem Phys 1994;100:5829–35.

[98] Tomasi J, Mennucci B, Cammi R. Quantum mechanical continuum solvation models. Chem Rev 2005;105:2999–3094. 16092826 10.1021/cr9904009

[99] Cowart M, Bennani YL, Faghih R, et al. Novel amines as Histamine-3 receptor ligands and their therapeutic applications, WO/2002/074758. 2002.

[100] Chao MN, Lorenzo-Ocampo MV, Szajnman SH, et al. Further insights of selenium-containing analogues of WC-9 against *Trypanosoma cruzi*. Bioorg Med Chem 2019;27:1350–61. 30808607 10.1016/j.bmc.2019.02.039 PMC6421105

[101] Zhu X, Li W, Luo X, et al. A catalyst-free and additive-free method for the synthesis of benzothiazolethiones from oiodoanilines, DMSO and potassium sulfide. Green Chem 2018;20:1970–4.

[102] Naidu A, Ganapathy D, Sekar G. Copper(I)-catalyzed intramolecular C_aryl_-O bond-forming cyclization for the synthesis of 1,4-benzodioxines and its application in the total synthesis of sweetening isovanillins. Synthesis 2010;2010:3509–19.

[103] Xie S, Li Y, Liu P, Sun P. Visible light-induced radical addition/annulation to construct phenylsulfonyl-functionalized dihydrobenzofurans involving an intramolecular 1,5-hydrogen atom transfer process. Org Lett 2020;22:8774–9. 33147046 10.1021/acs.orglett.0c03038

[104] Rong Z, Hu W, Dai N, Qian G. A Hg(OTf)_2_-catalyzed enolate umpolung reaction enables the synthesis of coumaran-3-ones and Indolin-3-ones. Org Lett 2020;22:3286–90. 32249582 10.1021/acs.orglett.0c01096

[105] Lee H, Torres J, Truong L, et al. Reducing agents affect inhibitory activities of compounds: results from multiple drug targets. Anal Biochem 2012;423:46–53. 22310499 10.1016/j.ab.2012.01.006 PMC3299889

[106] Miller D, Bailey C, Sammelson R. Synthesis of isoxazolines and isoxazoles inspired by fipronil. Synthesis 2015;47:2791–8.

[107] Li D, Liu L, Tian Y, et al. A flow strategy for the rapid, safe and scalable synthesis of N-H 1,2,3-triazoles via acetic acid mediated cycloaddition between nitroalkene and NaN_3_. Tetrahedron 2017;73:3959–65.

[108] Kallander LS, Ryan MD, Thompson SK, Compounds and Methods, WO/2003/031434. 2003.

[109] Lee H, Lee JK, Min S-J, et al. Copper(I)-catalyzed synthesis of 1,4-disubstituted 1,2,3-triazoles from azidoformates and aryl terminal alkynes. J Org Chem 2018;83:4805–11. 29600859 10.1021/acs.joc.8b00022

[110] Yan S, Gao Y, Xing R, et al. An efficient synthesis of (E)-nitroalkenes catalyzed by recoverable diamino-functionalized mesostructured polymers. Tetrahedron 2008;64:6294–9.

[111] Maiorana S, Pocar D, Dalla Croce P. Dalla Croce, P. Studies in the enamine field reactions of sulfonyl- and nitro-enamines with azides. Tetrahedron Lett 1966;7:6043–5.

[112] Adibekian A, Martin BR, Wang C, et al. Click-generated triazole ureas as ultrapotent *in vivo*–active serine hydrolase inhibitors. Nat Chem Biol 2011;7:469–78. 21572424 10.1038/nchembio.579 PMC3118922

[113] Efimov I, Bakulev V, Beliaev N, et al. Reactions of *β*-azolylenamines with sulfonyl azides as an approach to N-unsubstituted 1,2,3-triazoles and ethene-1,2-diamines. Eur J Org Chem 2014;2014:3684–9.

[114] Polo EC, Wang MF, Angnes RA, et al. Enantioselective heck arylation of acyclic alkenol aryl ethers: synthetic applications and DFT investigation of the stereoselectivity. Adv Synth Catal 2020;362:884–92.

[115] Eller C, Kehr G, Daniliuc CG, et al. Facile 1,1-carboboration reactions of acetylenic thioethers. Organometallics 2013;32:384–6.

[116] Al-Awadi NA, Mohamed AS, Habib OM, et al. Flash vacuum pyrolysis of acetylenic amides: a mechanistic study. J Anal Appl Pyrolysis 2020;150:104894.

[117] Blass BE, Coburn K, Lee W, et al. Synthesis and evaluation of (2-phenethyl-2H-1,2,3-triazol-4yl)(phenyl)methanones as Kv1.5 channel blockers for the treatment of atrial fibrillation. Bioorg Med Chem Lett 2006;16:4629–32. 16793267 10.1016/j.bmcl.2006.06.001

[118] Yang L, Wu Y, Yang Y, et al. Catalyst-free synthesis of 4-acyl-NH-1,2,3-triazoles by water-mediated cycloaddition reactions of enaminones and tosyl azide. Beilstein J Org Chem 2018;14:2348–53. 30254699 10.3762/bjoc.14.210 PMC6142726

[119] Cowan DJ, Larkin AL, Zhang C, et al. Novel compounds as antagonists or inverse agonists at opioid receptors, WO/2008/021849. 2008.

[120] Guirado A, López-Caracena L, López-Sánchez JI, et al. A new, high-yield synthesis of 3-aryl-1,2,4-triazoles. Tetrahedron 2016;72:8055–60.

[121] Bergman J, Brynolf A, Vuorinen E. A new synthesis of 4-amino-2-quinolinones. Tetrahedron 1986;42:3689–96.

[122] Aradi K, Novák Z. Copper-catalyzed oxidative ring closure of ortho cyanoanilides with hypervalent iodonium salts: arylation-ring closure approach to iminobenzoxazines. Adv Synth Catal 2015;357:371–6.

[123] Yoshida M, Sakauchi N, Sato A, Iminopyridine derivatives and use thereof, WO/2009/131245. 2009.

[124] Sturnio C, Deroy P, Duplessis M, et al. Inhibitors of HIV replication, WO/2010/115264. 2010.

[125] O’Broin CQ, Guiry PJ. Synthesis of 2-Amino-1,3-dienes from propargyl carbonates via palladium-catalyzed carbon–nitrogen bond formation. Org Lett 2020;22:879–83. 31939672 10.1021/acs.orglett.9b04413

[126] Kabsch W. Acta crystallographica Section D. Biol Crystallogr. 2010;66:125–132. 10.1107/S0907444909047337 PMC2815665 20124692

[127] Adams PD, Afonine PV, Bunkóczi G, et al. PHENIX: a comprehensive Python-based system for macromolecular structure solution. Acta Crystallogr Sect D Biol Crystallogr 2010;66:213–21. 20124702 10.1107/S0907444909052925 PMC2815670

[128] Emsley P, Lohkamp B, Scott WG, Cowtan K. Features and development of Coot. Acta Crystallogr Sect D Biol Crystallogr 2010;66:486–501. 20383002 10.1107/S0907444910007493 PMC2852313

[129] Pettersen EF, Goddard TD, Huang CC, et al. UCSF Chimera–A visualization system for exploratory research and analysis. J Comput Chem 2004;25:1605–12. 15264254 10.1002/jcc.20084

[130] Takikawa O, Kuroiwa T, Yamazaki F, Kido R. Mechanism of interferon-gamma action. Characterization of indoleamine 2,3-dioxygenase in cultured human cells induced by interferon-gamma and evaluation of the enzyme-mediated tryptophan degradation in its anticellular activity. J Biol Chem 1988;263:2041–8. 3123485

