# Supplementary material for: Structure-based optimization of type III indoleamine 2,3-dioxygenase 1 (IDO1) inhibitors
Source: J Enzyme Inhib Med Chem. 2022 Jun 27;37(1):1773–811. doi: 10.1080/14756366.2022.2089665 (PMC9246256; doi:10.1080/14756366.2022.2089665)
Supplement: Supplemental Material [file IENZ_A_2089665_SM3606.pdf]

Supporting Information: Structure-Based Optimization of Type III Indoleamine 2,3-Dioxygenase 1 (IDO1) Inhibitors

Ute F. Röhrig,[†,‡] Somi Reddy Majjigapu,[†,‡,¶] Pierre Vogel,[¶] Aline Reynaud,[§] Florence Pojer,[§] Nahzli Dilek,[‡] Patrick Reichenbach,[ǁ] Kelly Ascenção,[‡] Melita Irving,[ǁ] George Coukos,[ǁ] Olivier Michielin,[*,‡,⊥] and Vincent Zoete[*,‡,ǁ]

[†] Both authors contributed equally to this work (U.F.R. and S.R.M.)
[‡] SIB Swiss Institute of Bioinformatics, Molecular Modeling Group, CH-1015 Lausanne, Switzerland
[¶] Laboratory of Glycochemistry and Asymmetric Synthesis,
Ecole Polytechnique Fédérale de Lausanne (EPFL), CH-1015 Lausanne, Switzerland
[§] Protein Production and Structure Core Facility, School of Life Sciences,
Ecole Polytechnique Fédérale de Lausanne (EPFL), CH-1015 Lausanne, Switzerland
[ǁ] Department of Oncology UNIL-CHUV, Ludwig Lausanne Branch, 1066 Epalinges, Switzerland
[⊥] Department of Oncology, University Hospital of Lausanne (CHUV),
Ludwig Cancer Research-Lausanne Branch, 1011 Lausanne, Switzerland

E-mail: Olivier.Michielin@chuv.ch; Vincent.Zoete@unil.ch

Table of Contents

X-ray data collection and refinement statistics S2
Previously determined cellular data S3
Binding poses of 5-substituted 4-aryl-1,2,3-triazoles S4
Enzymatic dose-response curves of all compounds S5
HPLC purity traces S11
NMR spectra S15
References S107

**Structure**	**MMG-0472 (Compound 13)**
PDB entry	7zv3
**Data collection**	
Space group	P 21 21 21
Cell dimensions	
a, b, c (Å)	86.65 97.42 132.00
α, β, γ (°)	90 90 90
Resolution (Å)	48.71 – 2.55 (2.70–2.55)
R-meas (%)	8.2 (76.8)
Mean I/sigma(I)	15.20 (1.89)
Completeness (%)	99.5 (97.1)
**Refinement**	
Resolution (Å)	48.71 – 2.55 (2.62–2.55)
No. unique reflections	36929 (3506)
R-work/R-free	0.19/0.24
No. non-hydrogen atoms	6109
Protein	5915
Ligand/ion	142
Water	52
Average *B*-factors	60.76
Protein	60.80
Ligand/ion	60.92
Water	55.60
R.m.s. deviations	
Bond lengths (Å)	0.009
Bond angles (°)	1.05
Ramachandran	
Favored (%)	95.39
Allowed (%)	4.61
Outliers (%)	0.00

Table S1: X-ray data collection and refinement statistics. Statistics for the highest-resolution shell are shown in parentheses.

Compound	Conc. [μM]	Inh. [%]	Tox. [%]	IC_{50} [μM]
**9**	200	100	4[1]	0.11[1]
**10**				0.023[2]
**11**	200	100	0[1]	0.034[1]
**13**	200	76	71[3]	
**19**	200	93	3[1]	8.9[4]
**21**				7.0[4]
**22**				1.1[4]
**29**	200	95	0[1]	1.7[4]
**30**	200	95	0[1]	0.62[4]
**31**	200	100	26[1]	23[1]
**32**				14[4]
**40**	200	71	22[3]	
**41**	200	32	58[3]	
**42**	200	56	5[3]	110[3]
**46**				34[4]
**47**				>100[4]
**133**	200	0	0[1]	
**136**	200	78	0[1]	
**138**	200	52	6[1]	
**139**	200	100	0[1]	4.6[1]

Table S2: Previously determined cellular inhibition and toxicity data shown in Figure 6.

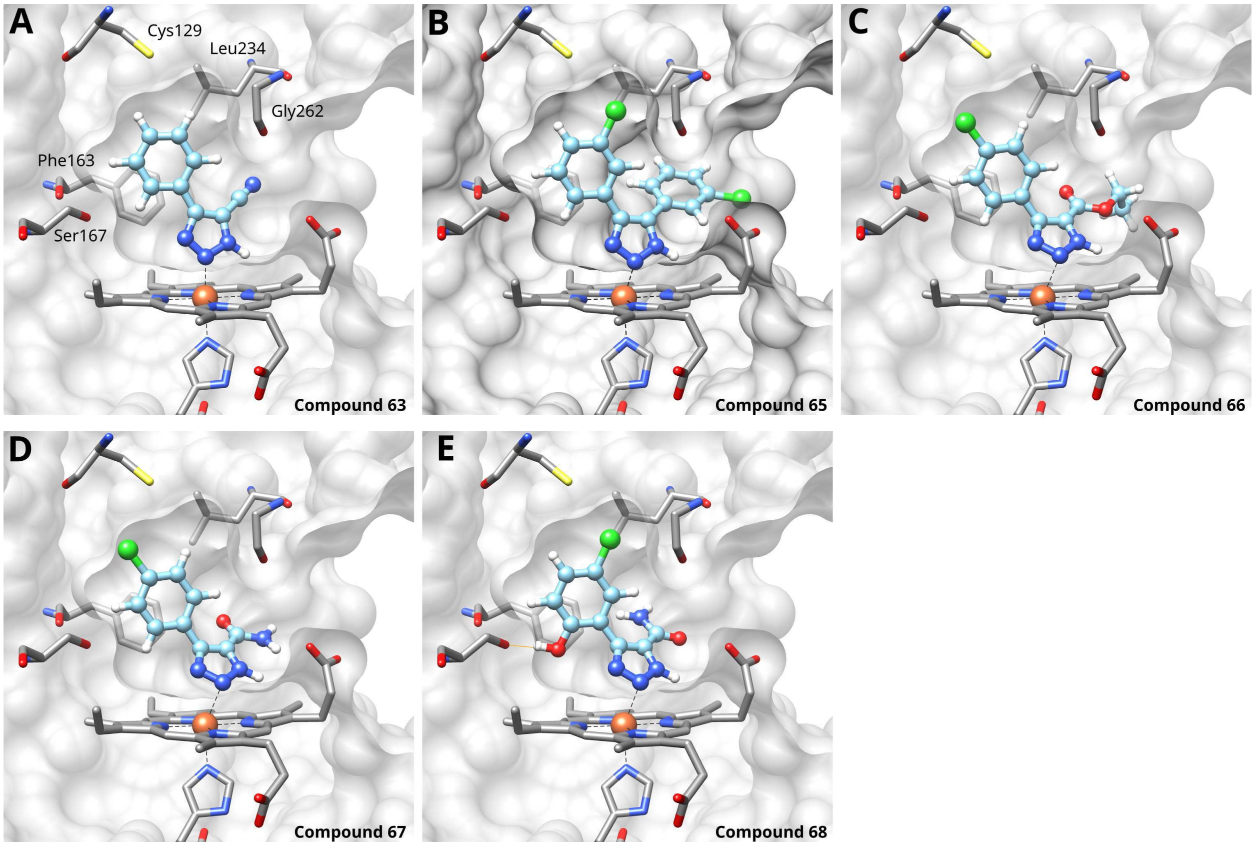

Figure S1: Binding poses predicted by docking for 5-substituted 4-aryl-1,2,3-triazoles. A. Compound **62**; B. Compound **64**; C. Compound **65**; D. Compound **66**; E. Compound **67**. The triazole ring does not assume a binding mode perpendicular to the heme plane.

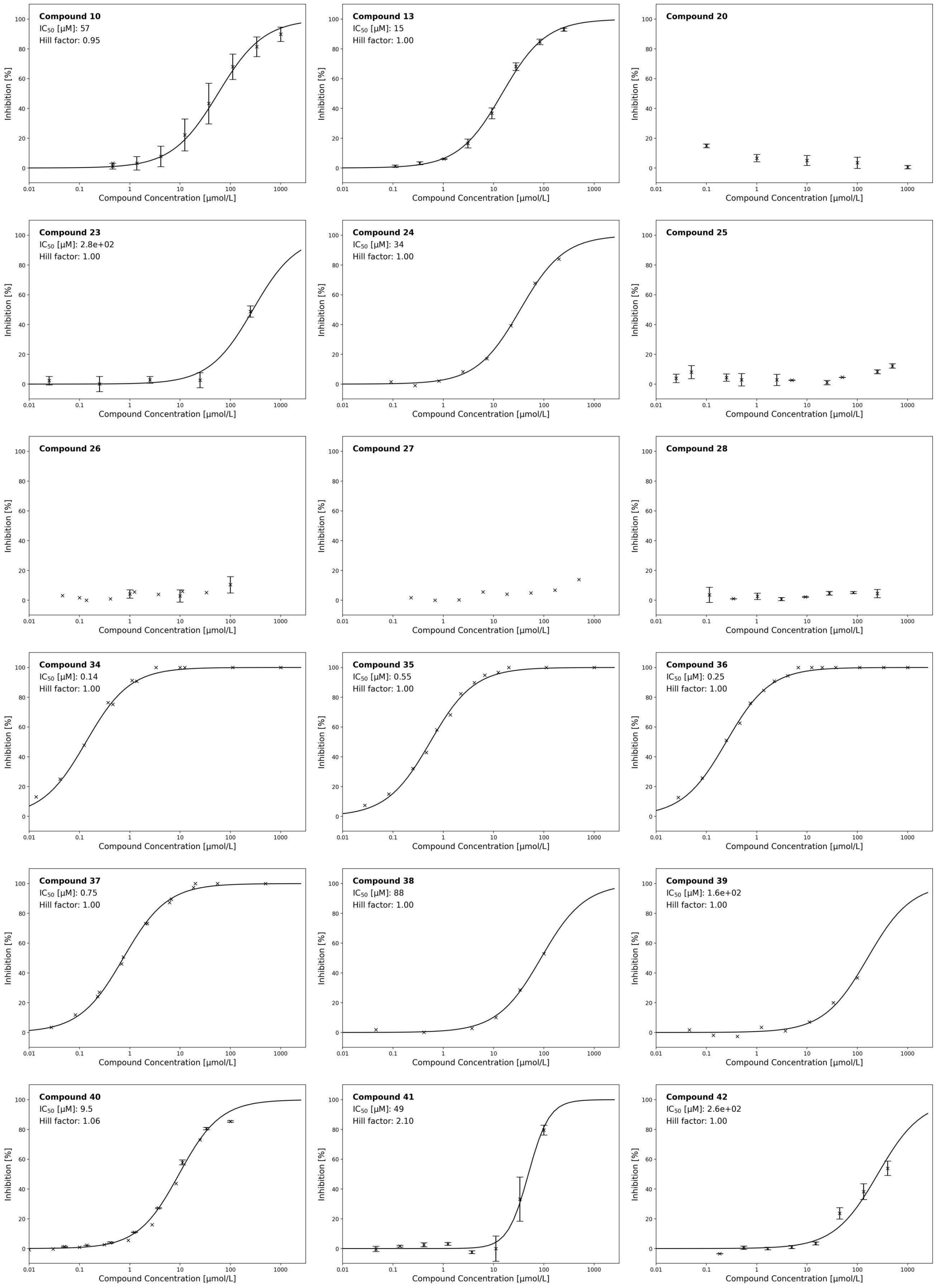

Figure S2: Enzymatic Dose-Response Curves of all Compounds (cont.).

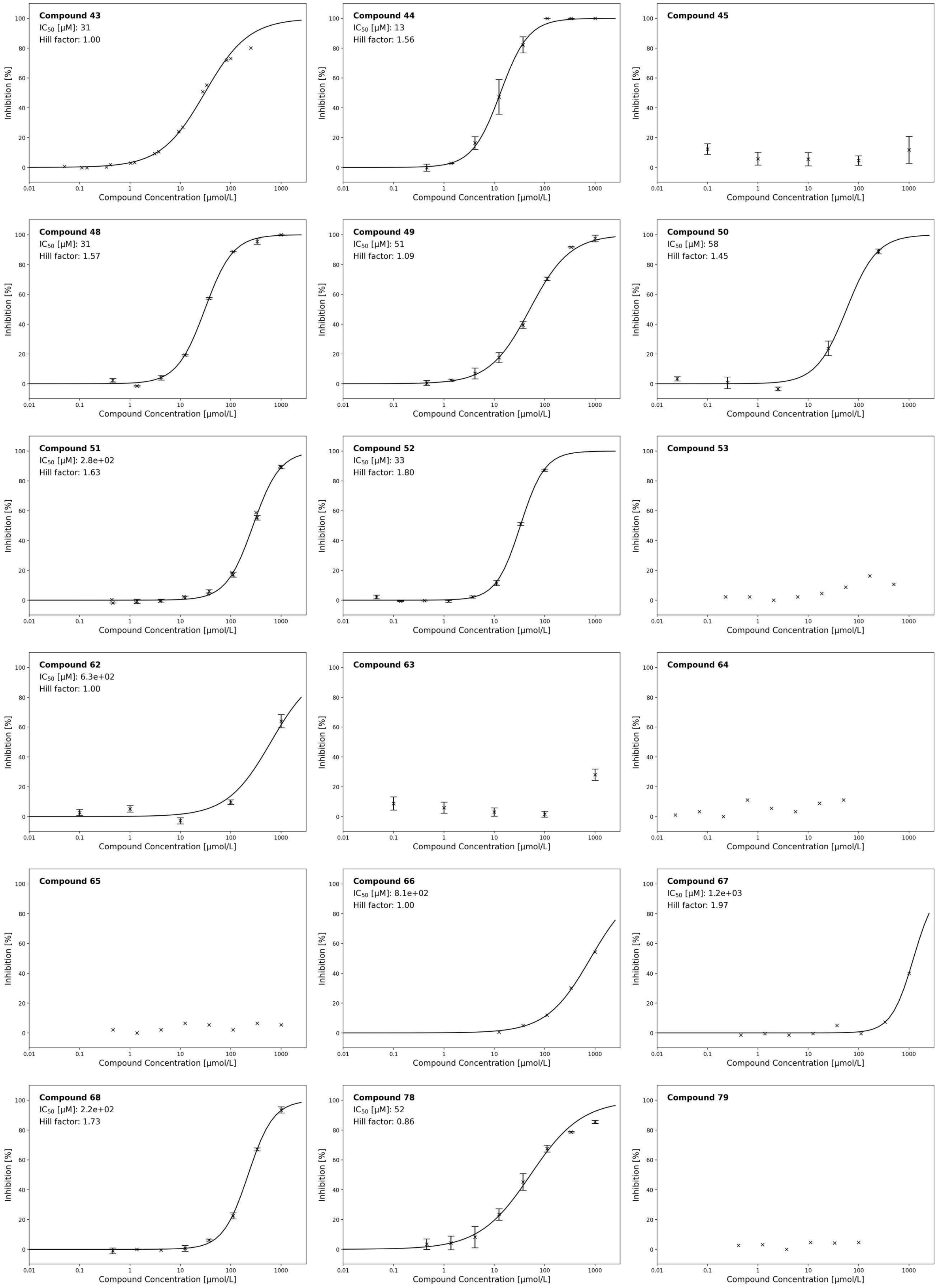
Compound 43
IC50 [μM]: 31
Hill factor: 1.00
Compound 44
IC50 [μM]: 13
Hill factor: 1.56
Compound 45
Compound 48
IC50 [μM]: 31
Hill factor: 1.57
Compound 49
IC50 [μM]: 51
Hill factor: 1.09
Compound 50
IC50 [μM]: 58
Hill factor: 1.45
Compound 51
IC50 [μM]: 2.8e+02
Hill factor: 1.63
Compound 52
IC50 [μM]: 33
Hill factor: 1.80
Compound 53
Compound 62
IC50 [μM]: 6.3e+02
Hill factor: 1.00
Compound 63
Compound 64
Compound 65
Compound 66
IC50 [μM]: 8.1e+02
Hill factor: 1.00
Compound 67
IC50 [μM]: 1.2e+03
Hill factor: 1.97
Compound 68
IC50 [μM]: 2.2e+02
Hill factor: 1.73
Compound 78
IC50 [μM]: 52
Hill factor: 0.86
Compound 79
Inhibition [%]
Compound Concentration [μmol/L]

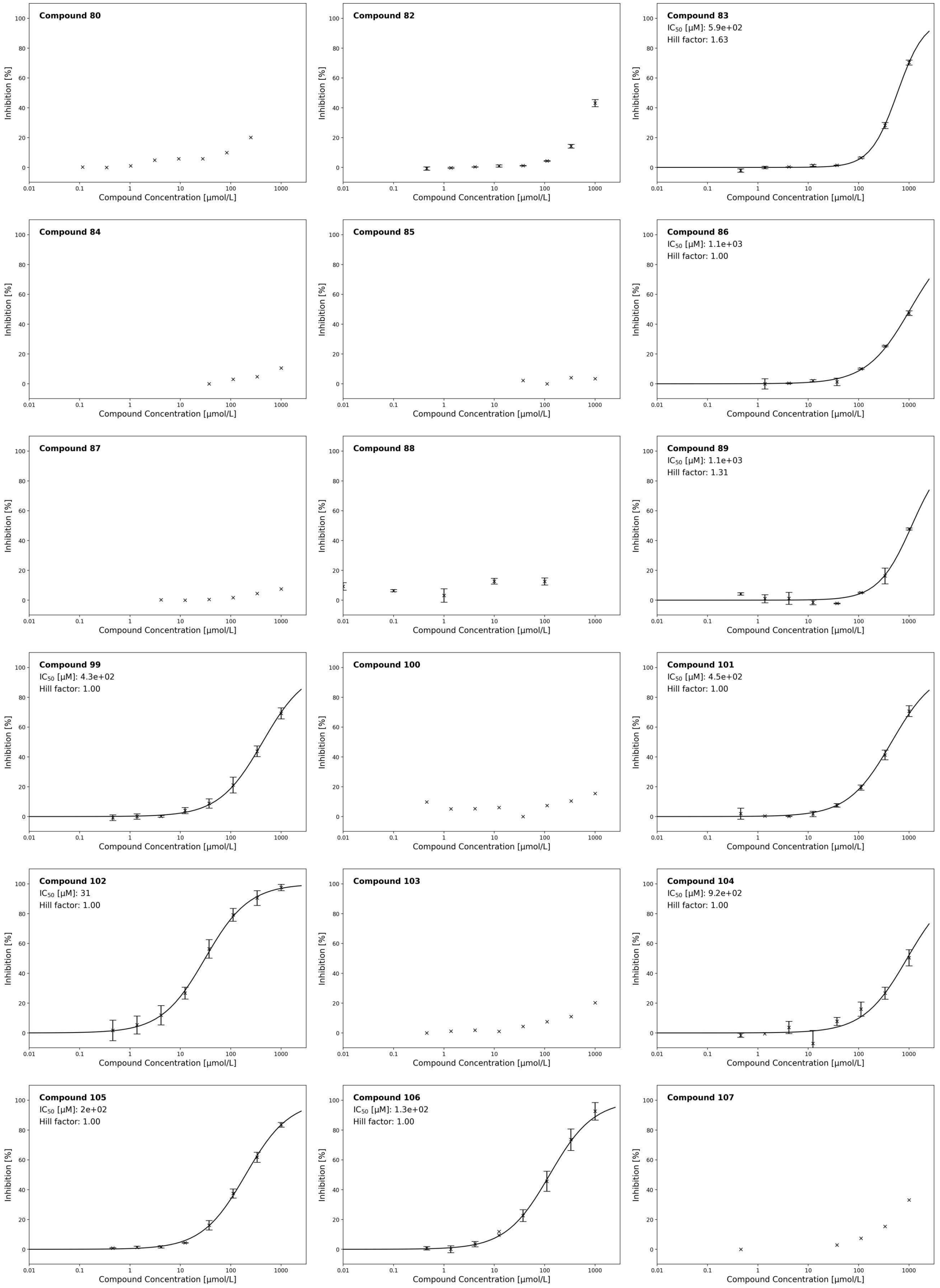
Compound 80
Compound 82
Compound 83
IC50 [μM]: 5.9e+02
Hill factor: 1.63
Compound 84
Compound 85
Compound 86
IC50 [μM]: 1.1e+03
Hill factor: 1.00
Compound 87
Compound 88
Compound 89
IC50 [μM]: 1.1e+03
Hill factor: 1.31
Compound 99
IC50 [μM]: 4.3e+02
Hill factor: 1.00
Compound 100
Compound 101
IC50 [μM]: 4.5e+02
Hill factor: 1.00
Compound 102
IC50 [μM]: 31
Hill factor: 1.00
Compound 103
Compound 104
IC50 [μM]: 9.2e+02
Hill factor: 1.00
Compound 105
IC50 [μM]: 2e+02
Hill factor: 1.00
Compound 106
IC50 [μM]: 1.3e+02
Hill factor: 1.00
Compound 107
Inhibition [%]
Compound Concentration [μmol/L]

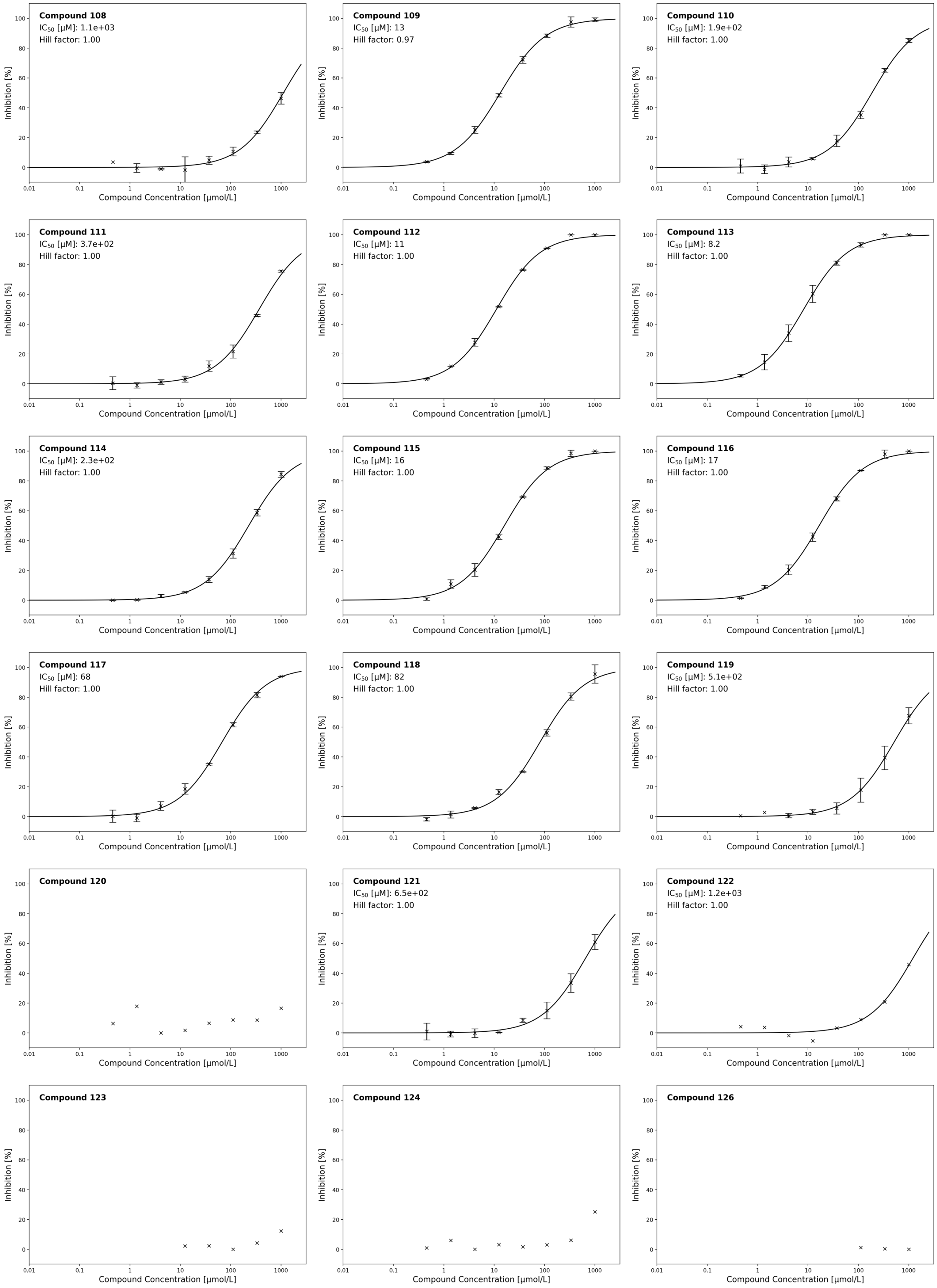
Compound 108
IC$_{50}$ [μM]: 1.1e+03
Hill factor: 1.00
Compound 109
IC$_{50}$ [μM]: 13
Hill factor: 0.97
Compound 110
IC$_{50}$ [μM]: 1.9e+02
Hill factor: 1.00
Compound 111
IC$_{50}$ [μM]: 3.7e+02
Hill factor: 1.00
Compound 112
IC$_{50}$ [μM]: 11
Hill factor: 1.00
Compound 113
IC$_{50}$ [μM]: 8.2
Hill factor: 1.00
Compound 114
IC$_{50}$ [μM]: 2.3e+02
Hill factor: 1.00
Compound 115
IC$_{50}$ [μM]: 16
Hill factor: 1.00
Compound 116
IC$_{50}$ [μM]: 17
Hill factor: 1.00
Compound 117
IC$_{50}$ [μM]: 68
Hill factor: 1.00
Compound 118
IC$_{50}$ [μM]: 82
Hill factor: 1.00
Compound 119
IC$_{50}$ [μM]: 5.1e+02
Hill factor: 1.00
Compound 120
Compound 121
IC$_{50}$ [μM]: 6.5e+02
Hill factor: 1.00
Compound 122
IC$_{50}$ [μM]: 1.2e+03
Hill factor: 1.00
Compound 123
Compound 124
Compound 126
Inhibition [%]
Compound Concentration [μmol/L]

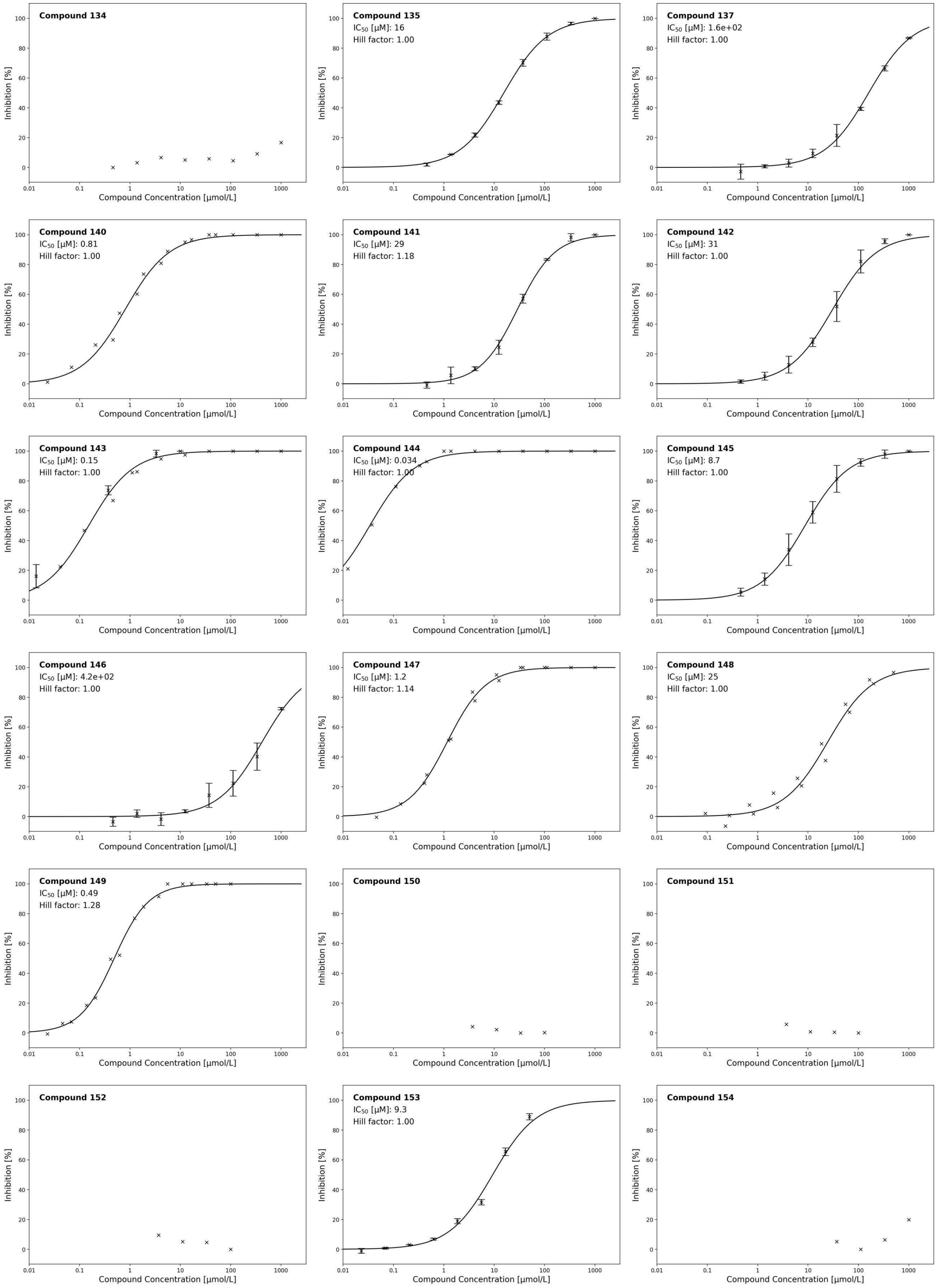
Compound 134
Compound 135
IC$_{50}$ [μM]: 16
Hill factor: 1.00
Compound 137
IC$_{50}$ [μM]: 1.6e+02
Hill factor: 1.00
Compound 140
IC$_{50}$ [μM]: 0.81
Hill factor: 1.00
Compound 141
IC$_{50}$ [μM]: 29
Hill factor: 1.18
Compound 142
IC$_{50}$ [μM]: 31
Hill factor: 1.00
Compound 143
IC$_{50}$ [μM]: 0.15
Hill factor: 1.00
Compound 144
IC$_{50}$ [μM]: 0.034
Hill factor: 1.00
Compound 145
IC$_{50}$ [μM]: 8.7
Hill factor: 1.00
Compound 146
IC$_{50}$ [μM]: 4.2e+02
Hill factor: 1.00
Compound 147
IC$_{50}$ [μM]: 1.2
Hill factor: 1.14
Compound 148
IC$_{50}$ [μM]: 25
Hill factor: 1.00
Compound 149
IC$_{50}$ [μM]: 0.49
Hill factor: 1.28
Compound 150
Compound 151
Compound 152
Compound 153
IC$_{50}$ [μM]: 9.3
Hill factor: 1.00
Compound 154
Inhibition [%]
Compound Concentration [μmol/L]

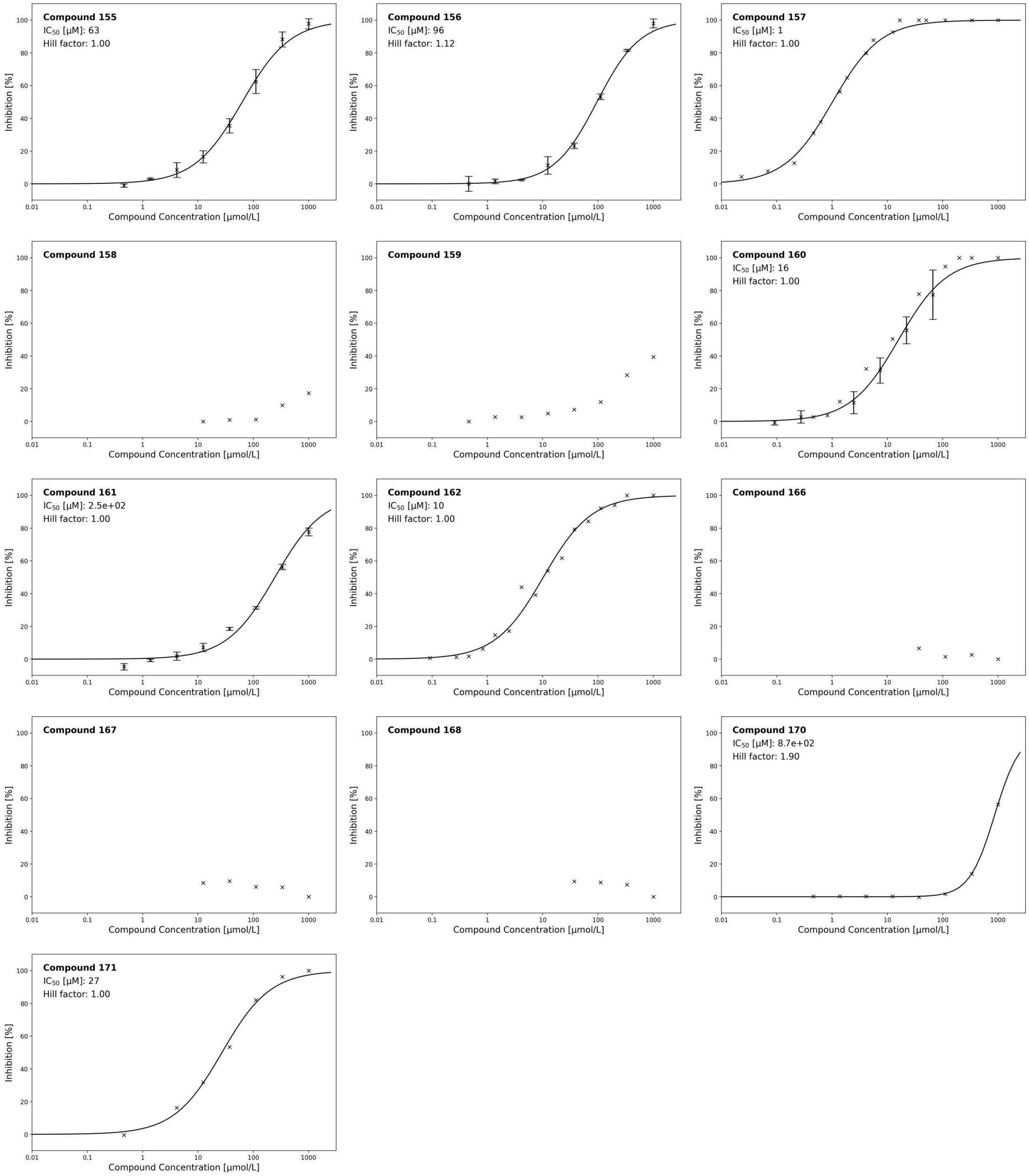
Compound 155
IC50 [μM]: 63
Hill factor: 1.00
Compound 156
IC50 [μM]: 96
Hill factor: 1.12
Compound 157
IC50 [μM]: 1
Hill factor: 1.00
Compound 158
Compound 159
Compound 160
IC50 [μM]: 16
Hill factor: 1.00
Compound 161
IC50 [μM]: 2.5e+02
Hill factor: 1.00
Compound 162
IC50 [μM]: 10
Hill factor: 1.00
Compound 166
Compound 167
Compound 168
Compound 170
IC50 [μM]: 8.7e+02
Hill factor: 1.90
Compound 171
IC50 [μM]: 27
Hill factor: 1.00
Inhibition [%]
Compound Concentration [μmol/L]

Chromatogram and Results

Injection Details

Injection Name:	**1532**	*Run Time (min):*	**41.00**
Vial Number:	**RB4**	*Injection Volume:*	**25.00**
Injection Type:	**Unknown**	*Channel:*	**UV_VIS_1**
Calibration Level:		*Wavelength:*	**220**
Instrument Method:	**0-100% 1mL 30min Somi**	*Bandwidth:*	**4**
Processing Method:	**Basic Quantitative**	*Dilution Factor:*	**1.0000**
Injection Date/Time:	**27.août.21 11:56**	*Sample Weight:*	**1.0000**

Chromatogram

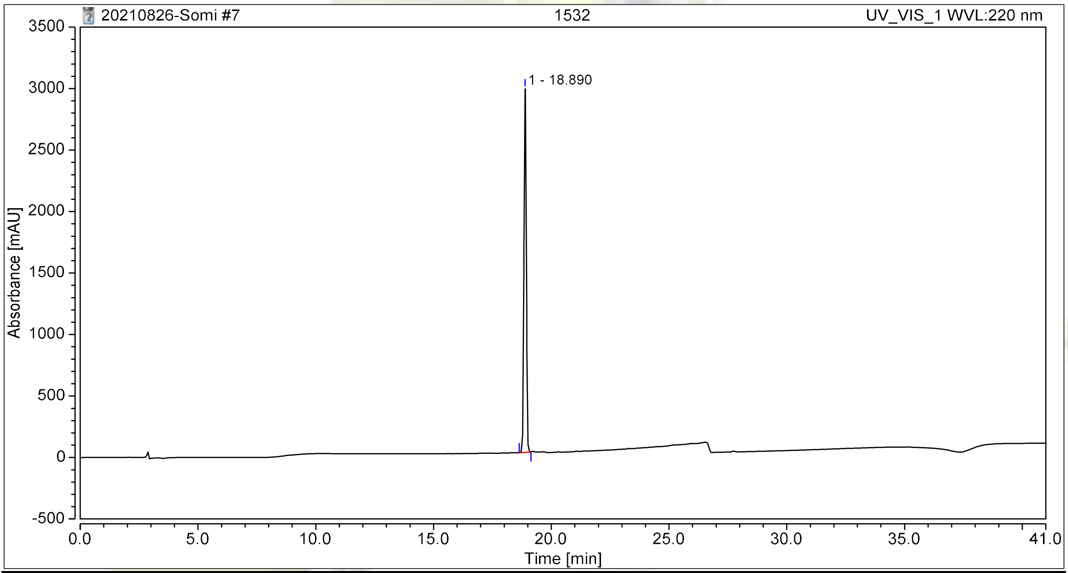

Integration Results

No.	Peak Name	Retention Time min	Area mAU*min	Height mAU	Relative Area %	Relative Height %	Amount n.a.
1		18.890	358.680	2956.073	100.00	100.00	n.a.
**Total:**			**358.680**	**2956.073**	**100.00**	**100.00**	

Default DAD/Integration

Chromeleon (c) Dionex
Version 7.2.6.10049

Figure S3: HPLC purity trace of compound **34**.

Chromatogram and Results

Injection Details

Injection Name:	**1019**	*Run Time (min):*	**41.00**
Vial Number:	**RB6**	*Injection Volume:*	**25.00**
Injection Type:	**Unknown**	*Channel:*	**UV_VIS_1**
Calibration Level:		*Wavelength:*	**220**
Instrument Method:	**0-100% 1mL 30min Somi**	*Bandwidth:*	**4**
Processing Method:	**Basic Quantitative**	*Dilution Factor:*	**1.0000**
Injection Date/Time:	**27.août.21 13:20**	*Sample Weight:*	**1.0000**

Chromatogram

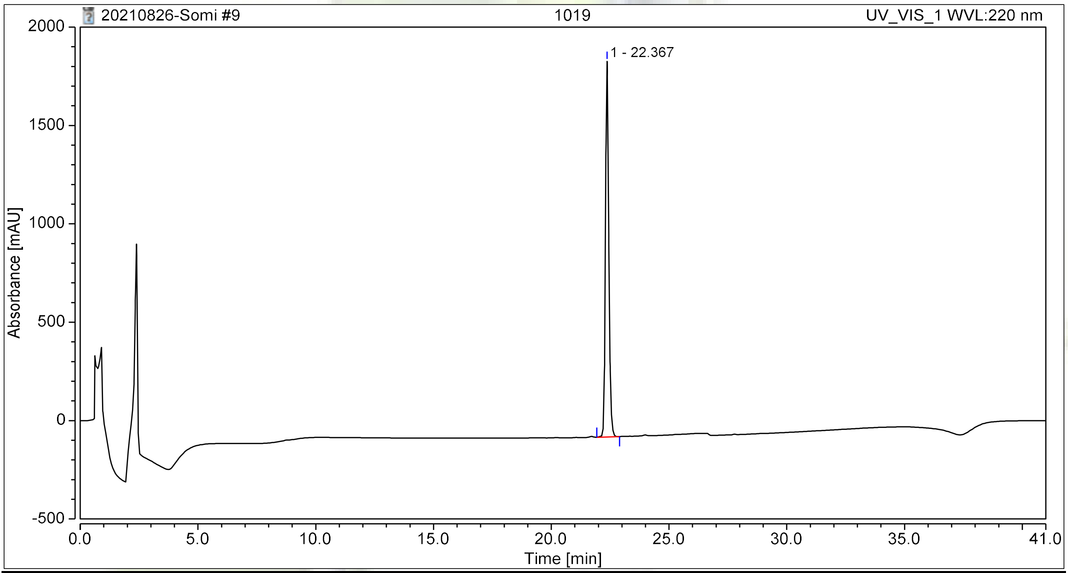

Integration Results

No.	Peak Name	Retention Time min	Area mAU*min	Height mAU	Relative Area %	Relative Height %	Amount n.a.
1		22.367	315.093	1907.890	100.00	100.00	n.a.
**Total:**			**315.093**	**1907.890**	**100.00**	**100.00**	

Default DAD/Integration

Chromeleon (c) Dionex
Version 7.2.6.10049

Figure S4: HPLC purity trace of compound **113**.

Chromatogram and Results

Injection Details

Injection Name:	**1395**	*Run Time (min):*	**41.00**
Vial Number:	**RB7**	*Injection Volume:*	**25.00**
Injection Type:	**Unknown**	*Channel:*	**UV_VIS_1**
Calibration Level:		*Wavelength:*	**220**
Instrument Method:	**0-100% 1mL 30min Somi**	*Bandwidth:*	**4**
Processing Method:	**Basic Quantitative**	*Dilution Factor:*	**1.0000**
Injection Date/Time:	**27.août.21 14:02**	*Sample Weight:*	**1.0000**

Chromatogram

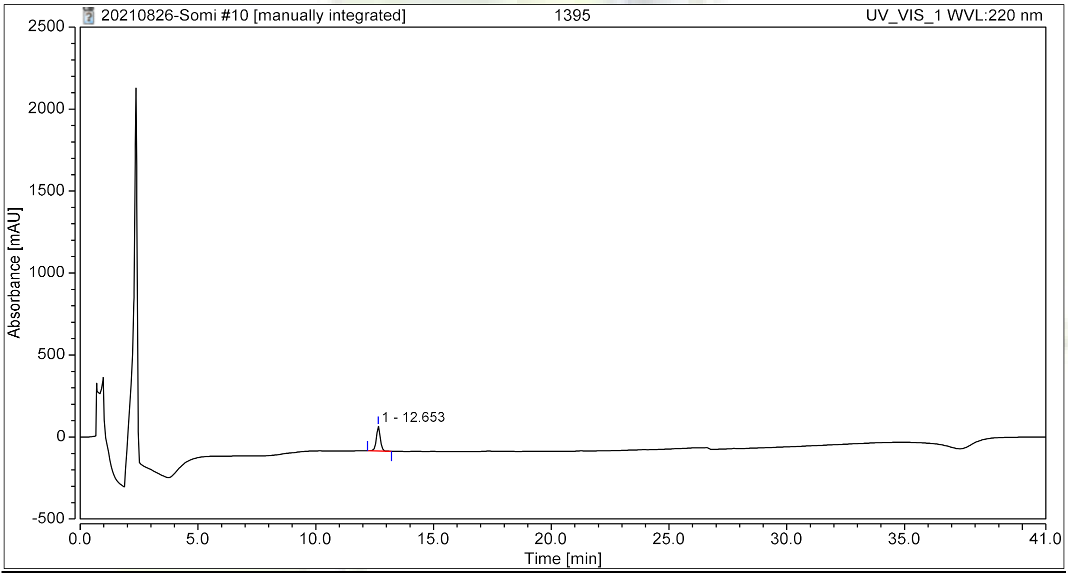

Integration Results

No.	Peak Name	Retention Time min	Area mAU*min	Height mAU	Relative Area %	Relative Height %	Amount n.a.
1		12.653	29.276	150.719	100.00	100.00	n.a.
**Total:**			**29.276**	**150.719**	**100.00**	**100.00**	

Default DAD/Integration

Chromeleon (c) Dionex
Version 7.2.6.10049

Figure S5: HPLC purity trace of compound **143**.

Chromatogram and Results

Injection Details

Injection Name:	**1363-2**	*Run Time (min):*	**41.00**
Vial Number:	**RB3**	*Injection Volume:*	**25.00**
Injection Type:	**Unknown**	*Channel:*	**UV_VIS_1**
Calibration Level:		*Wavelength:*	**220**
Instrument Method:	**0-100% 1mL 30min Somi**	*Bandwidth:*	**4**
Processing Method:	**Basic Quantitative**	*Dilution Factor:*	**1.0000**
Injection Date/Time:	**27.août.21 10:47**	*Sample Weight:*	**1.0000**

Chromatogram

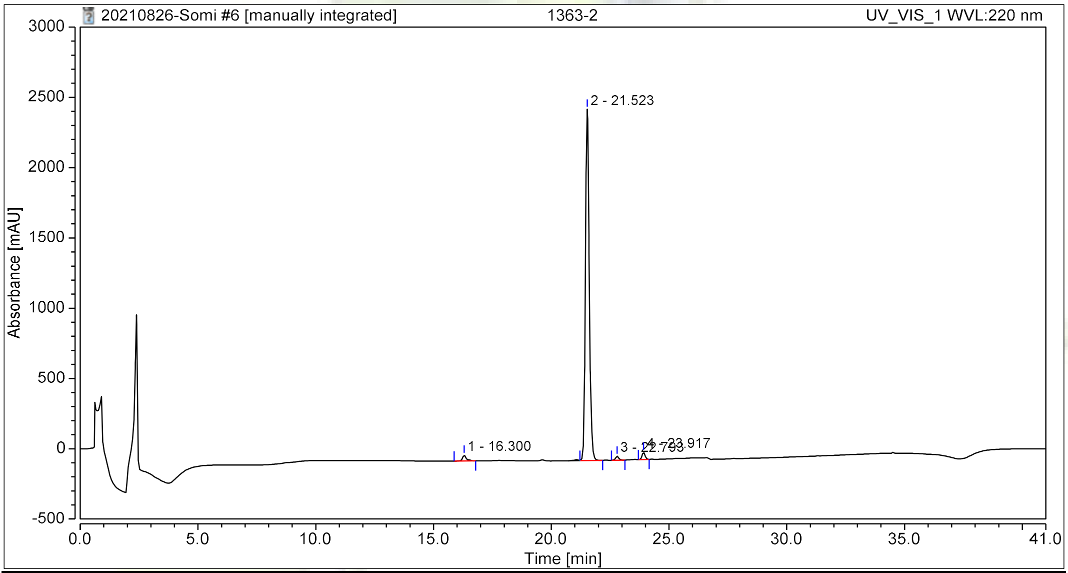

Integration Results

No.	Peak Name	Retention Time min	Area mAU*min	Height mAU	Relative Area %	Relative Height %	Amount n.a.
1		16.300	8.128	41.164	1.68	1.57	n.a.
2		21.523	464.352	2499.596	95.84	95.34	n.a.
3		22.793	4.528	28.990	0.93	1.11	n.a.
4		23.917	7.496	51.920	1.55	1.98	n.a.
**Total:**			**484.504**	**2621.670**	**100.00**	**100.00**	

Default DAD/Integration

Chromeleon (c) Dionex
Version 7.2.6.10049

Figure S6: HPLC purity trace of compound **149**.

4-(1,2,3-triazol-4-yl)phenol (20) : ^{1}H NMR (400 MHz, $CDCl_3$)

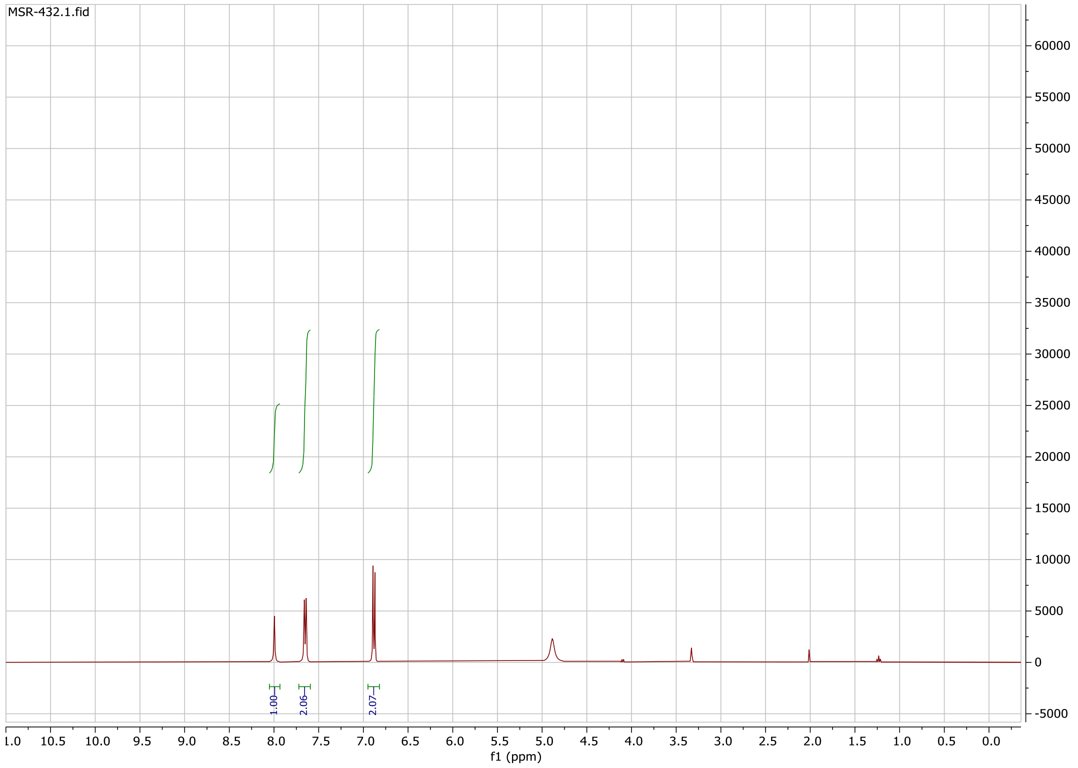

4-(1,2,3-triazol-4-yl)phenol (20) : ^{13}C NMR (101 MHz, CD_3OD)

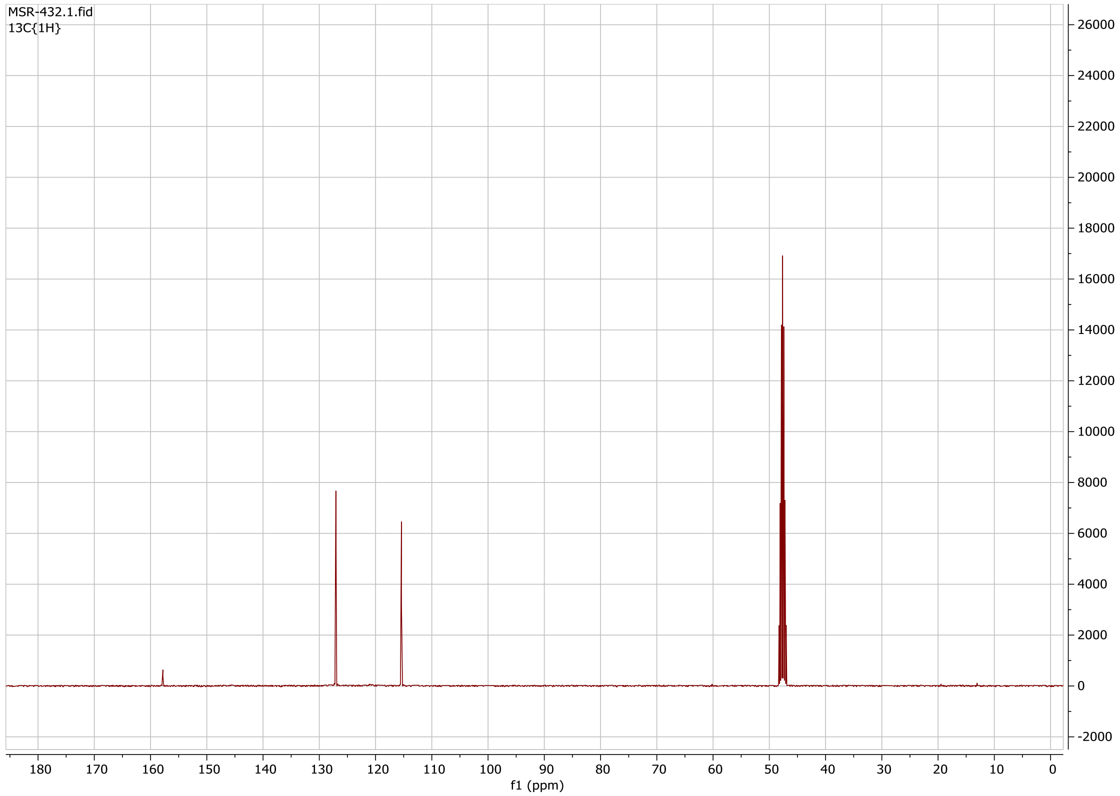

4-(3-Isopropylphenyl)-1,2,3-triazole (23) : ^{1}H NMR (400 MHz, $CDCl_3$)

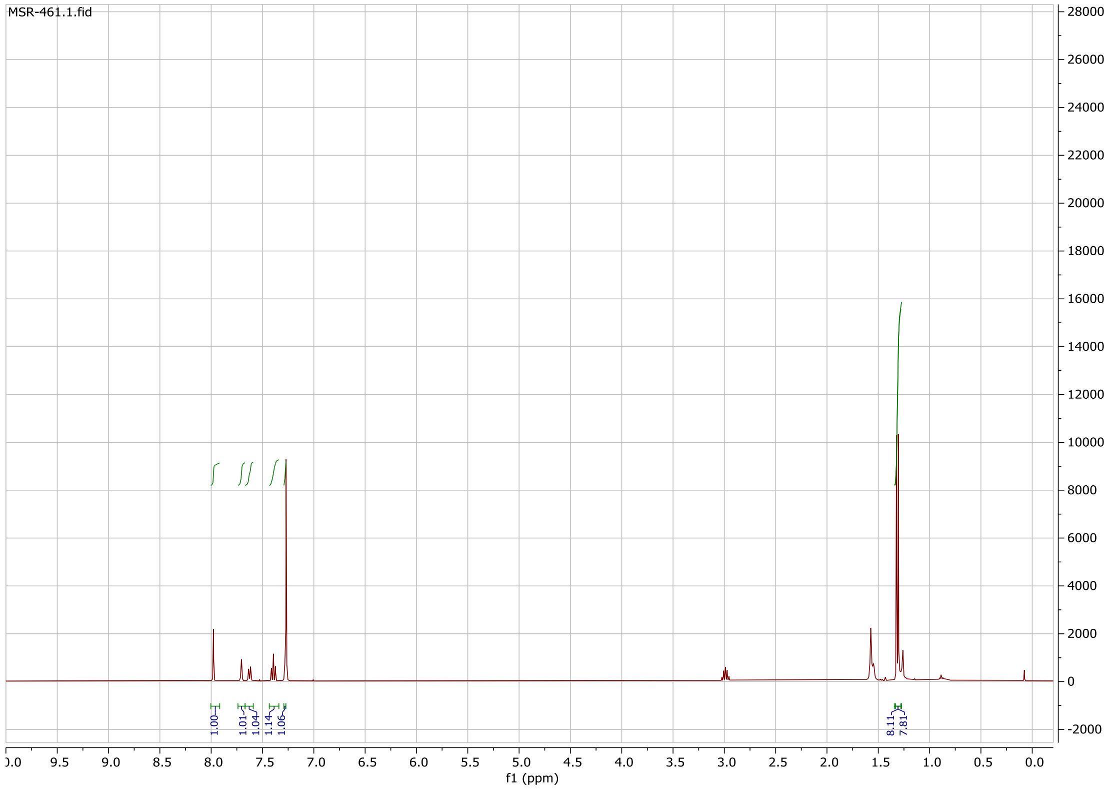

4-(3-Isopropylphenyl)-1,2,3-triazole (23) : ^{13}C NMR (101 MHz, $CDCl_3$)

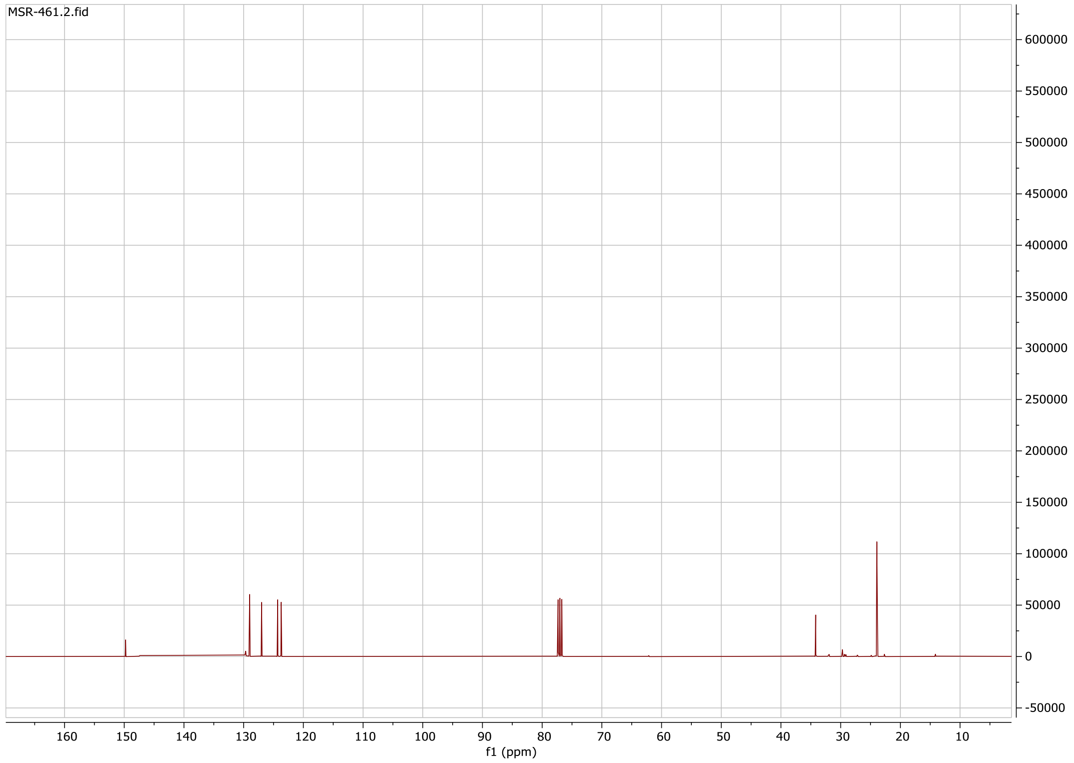

4-(3-Butylphenyl)-1,2,3-triazole (24):^{1}H NMR (400 MHz, CD_3OD)

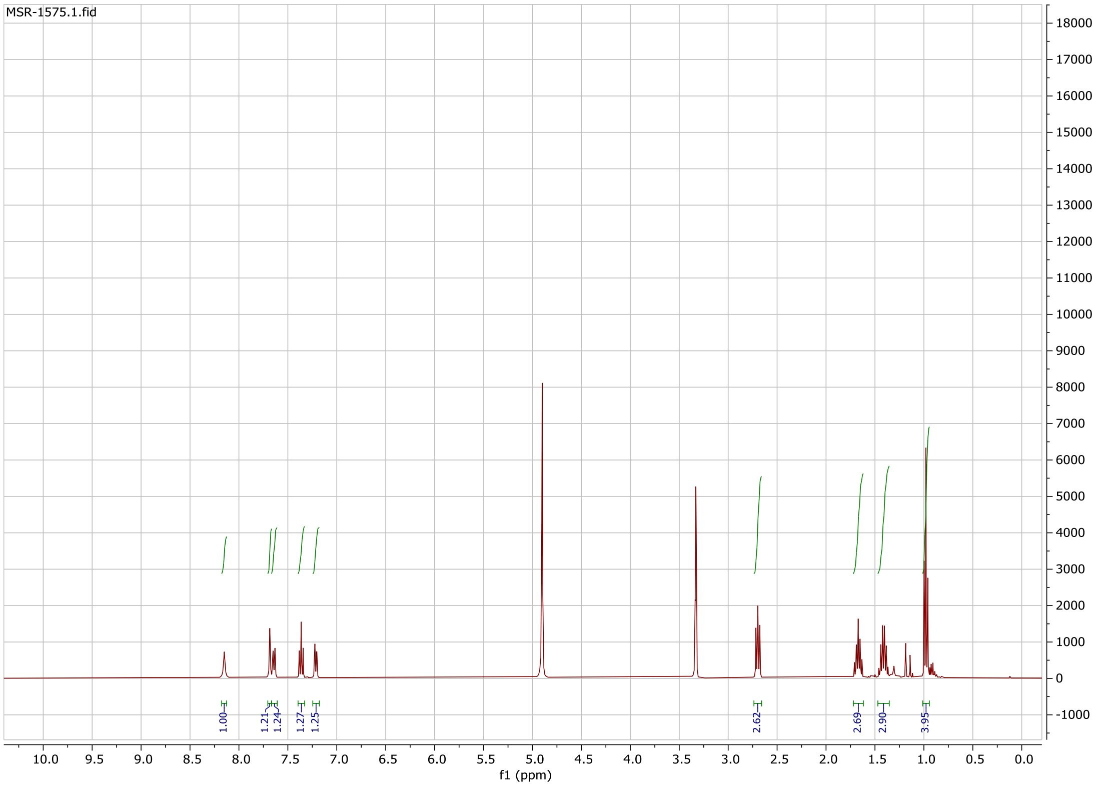

4-(3-Butylphenyl)-1,2,3-triazole (24):^{13}C NMR (101 MHz, CD_3OD)

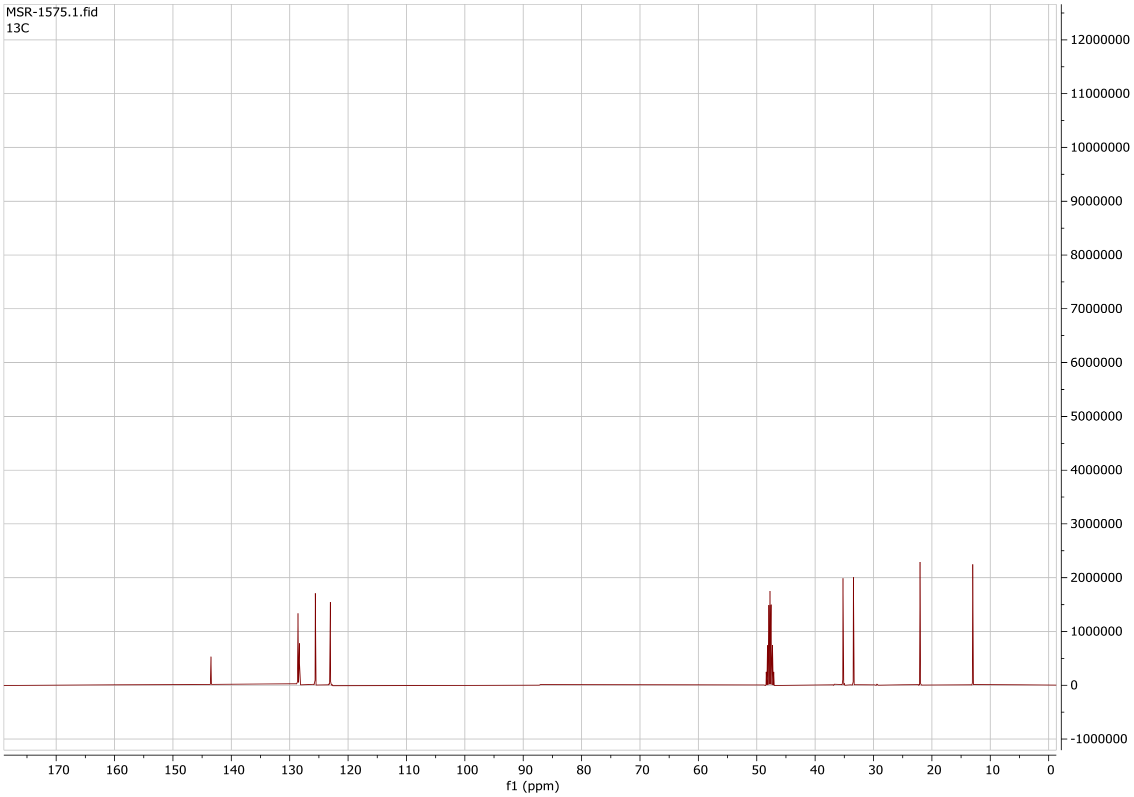

4-(2-Benzyloxyphenyl)-1,2,3-triazole (25) : ^{1}H NMR (400 MHz, CD_3OD)

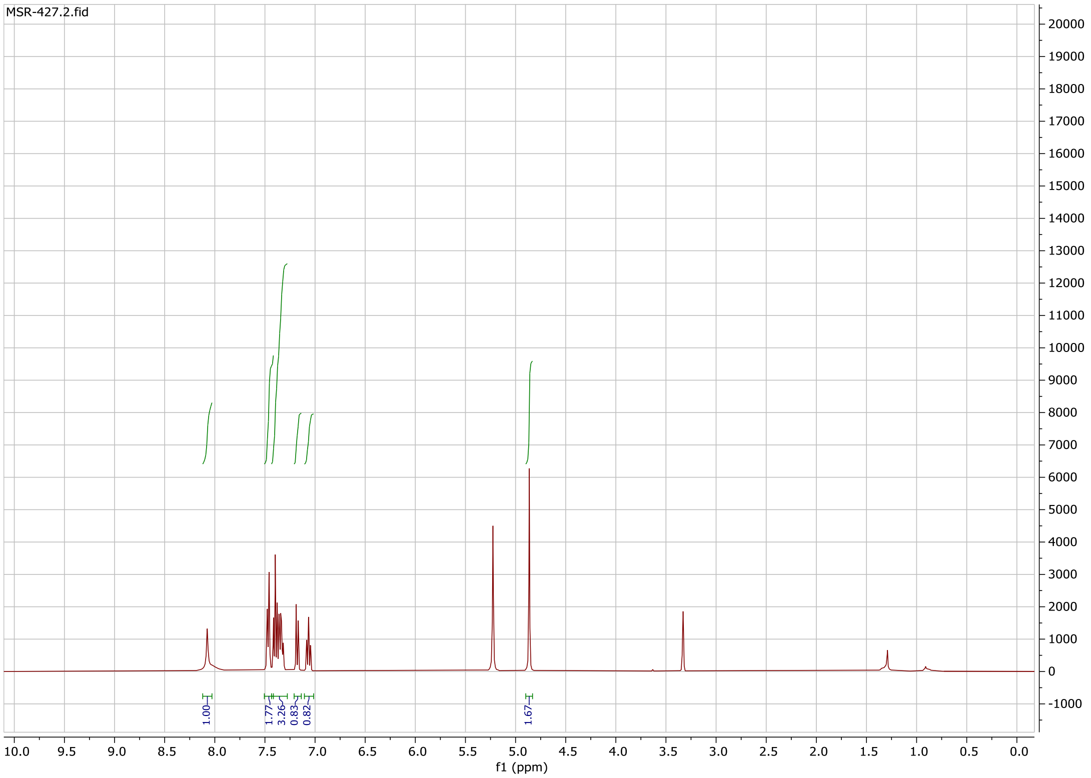

4-(2-Benzyloxyphenyl)-1,2,3-triazole (25) : ^{13}C NMR (101 MHz, CD_3OD)

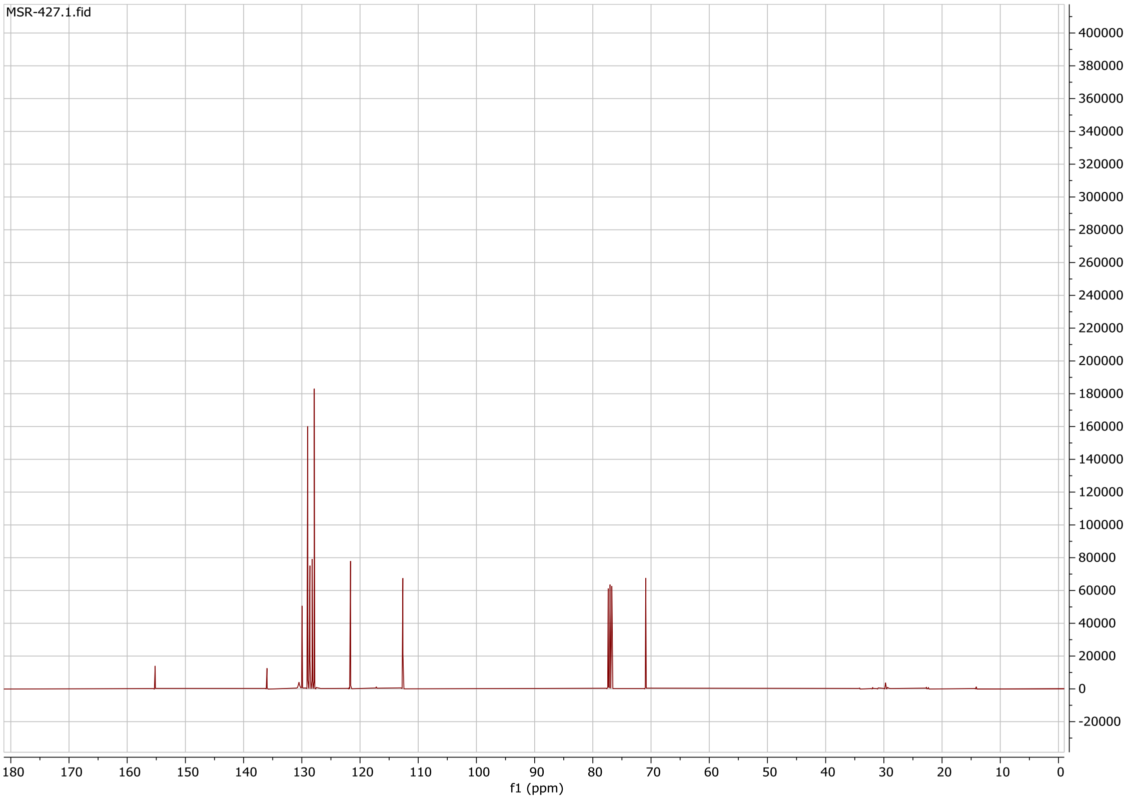

4-(2-Phenethoxyphenyl)-1,2,3-triazole (26) : ^{1}H NMR (400 MHz, $CDCl_3$)

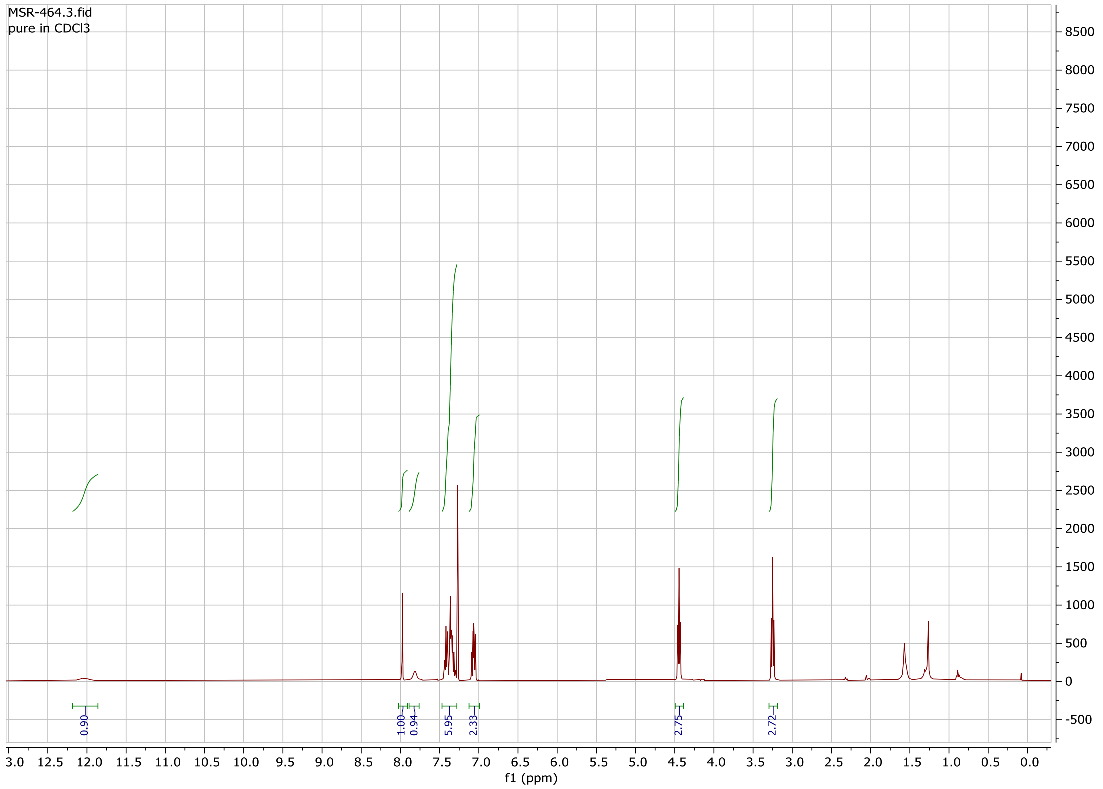

4-(2-Phenethoxyphenyl)-1,2,3-triazole (26) : ^{13}C NMR (101 MHz, $CDCl_3$)

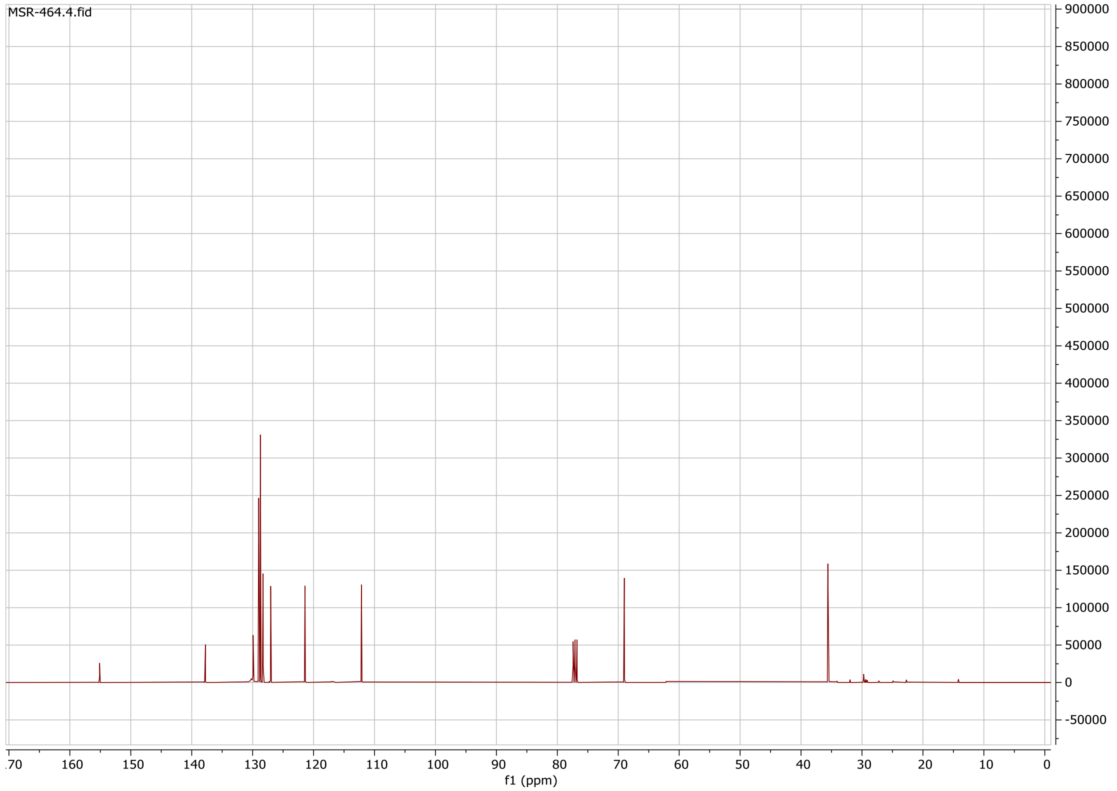

2-(2-(1H-1,2,3-triazol-5-yl)phenoxy)-1-phenylethan-1-ol (27) : ^{1}H NMR (400 MHz, $CDCl_3$)

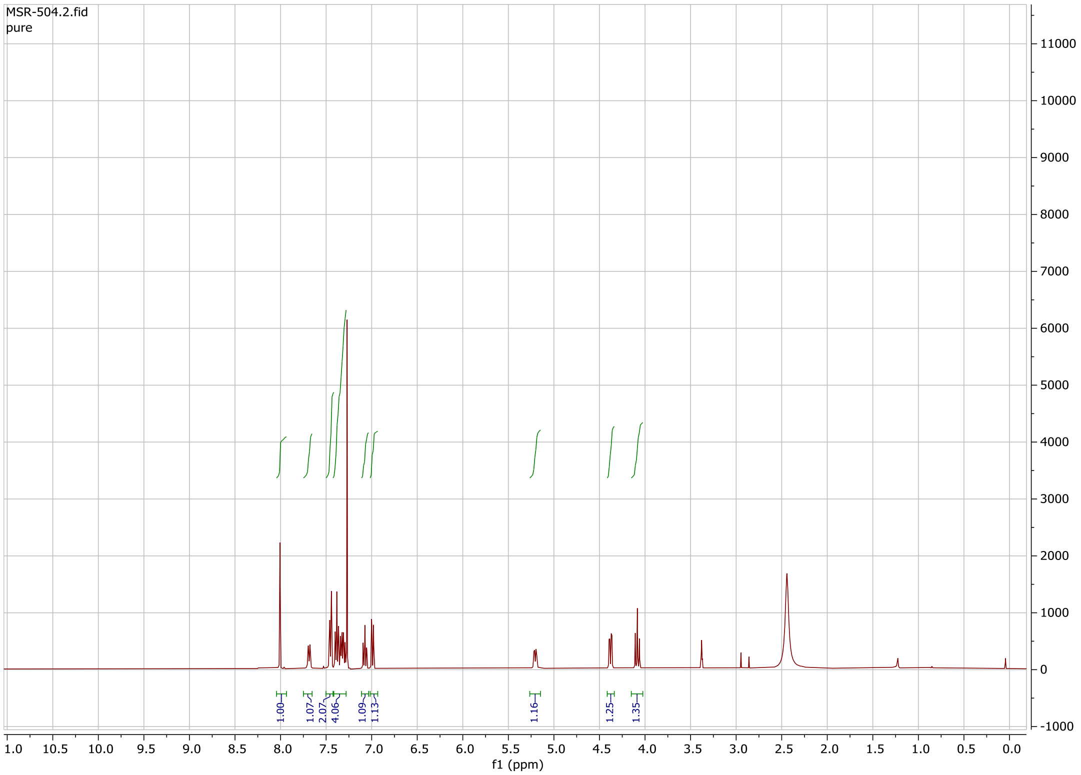

2-(2-(1H-1,2,3-triazol-5-yl)phenoxy)-1-phenylethan-1-ol (27) : ^{13}C NMR (101 MHz, CD_3OD)

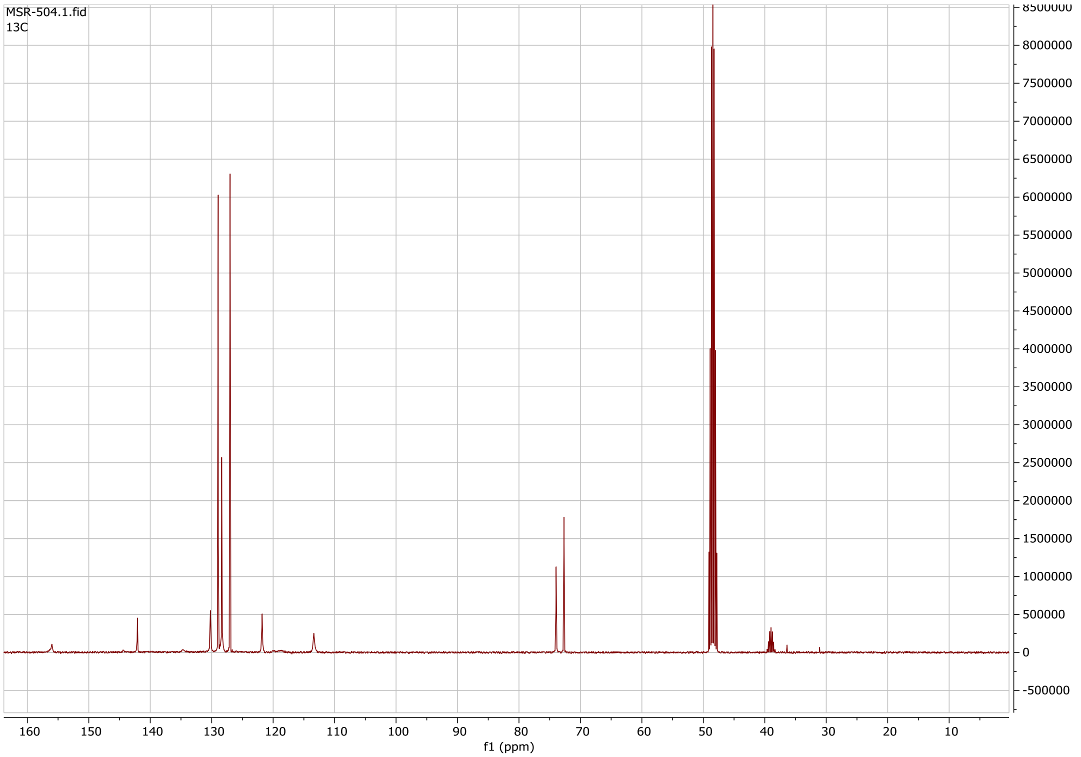

N-Phenyl-2-(1H-1,2,3-triazol-5-yl)aniline (28) : ^{1}H NMR (400 MHz, CD_3OD)

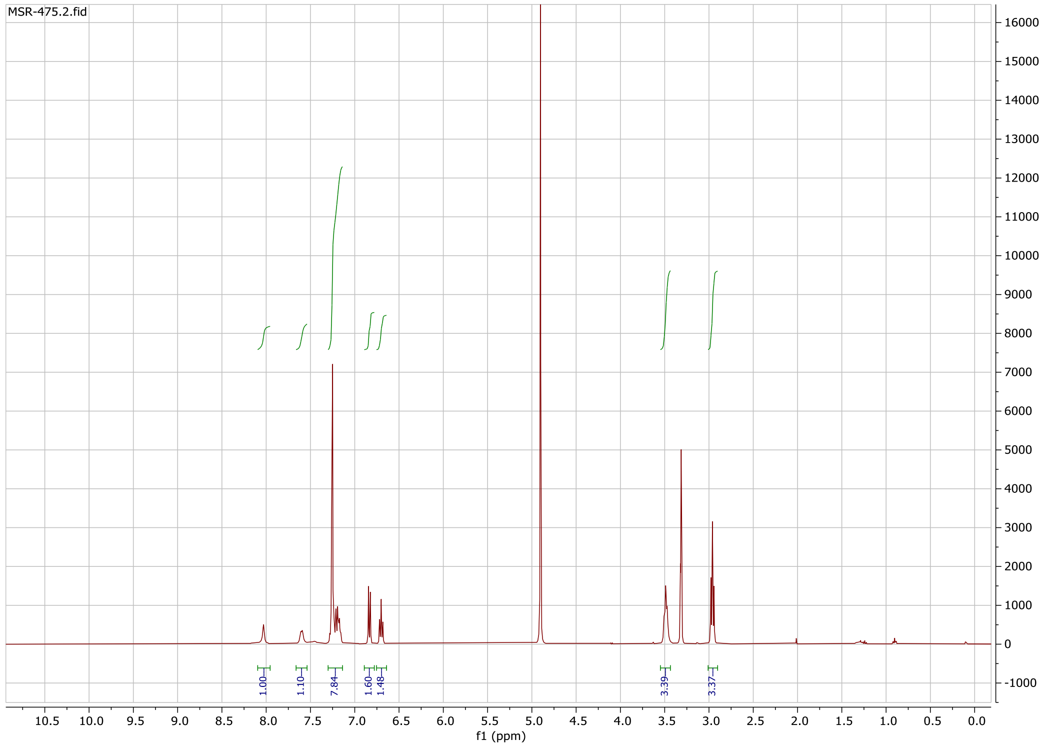

N-Phenyl-2-(1H-1,2,3-triazol-5-yl)aniline (28) : ^{13}C NMR (101 MHz, CD_3OD)

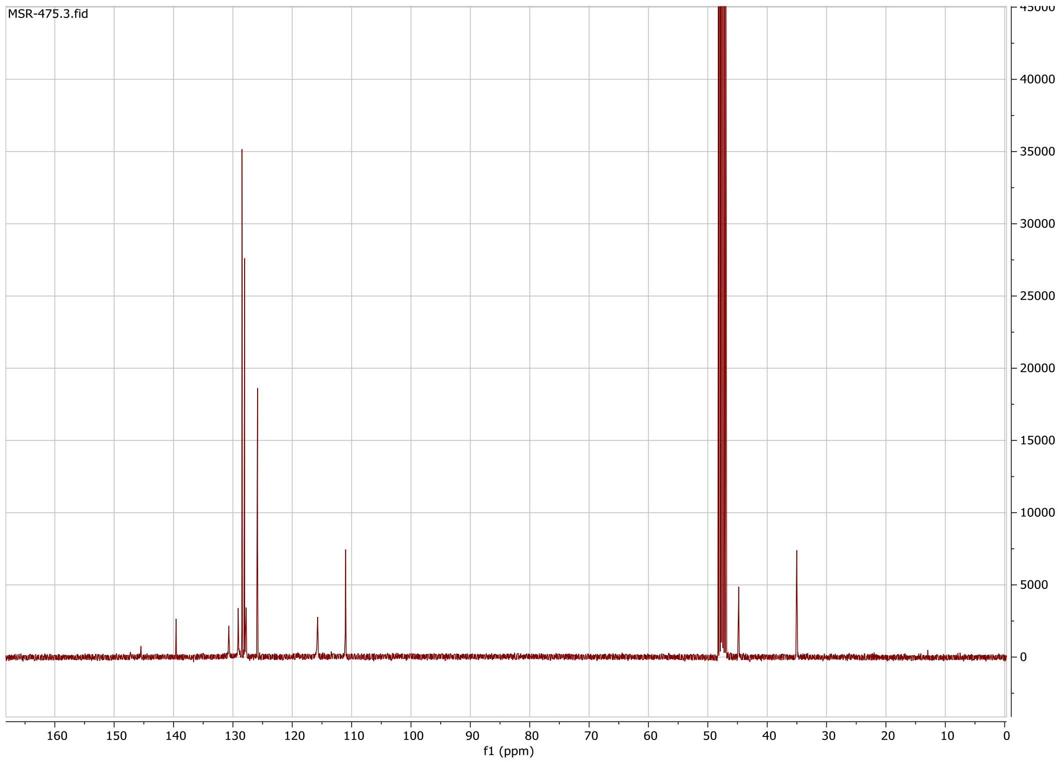

4-Fluoro-2-(1H-1,2,3-triazol-5-yl)phenol (34): ^{1}H NMR (400 MHz, CD_3OD)

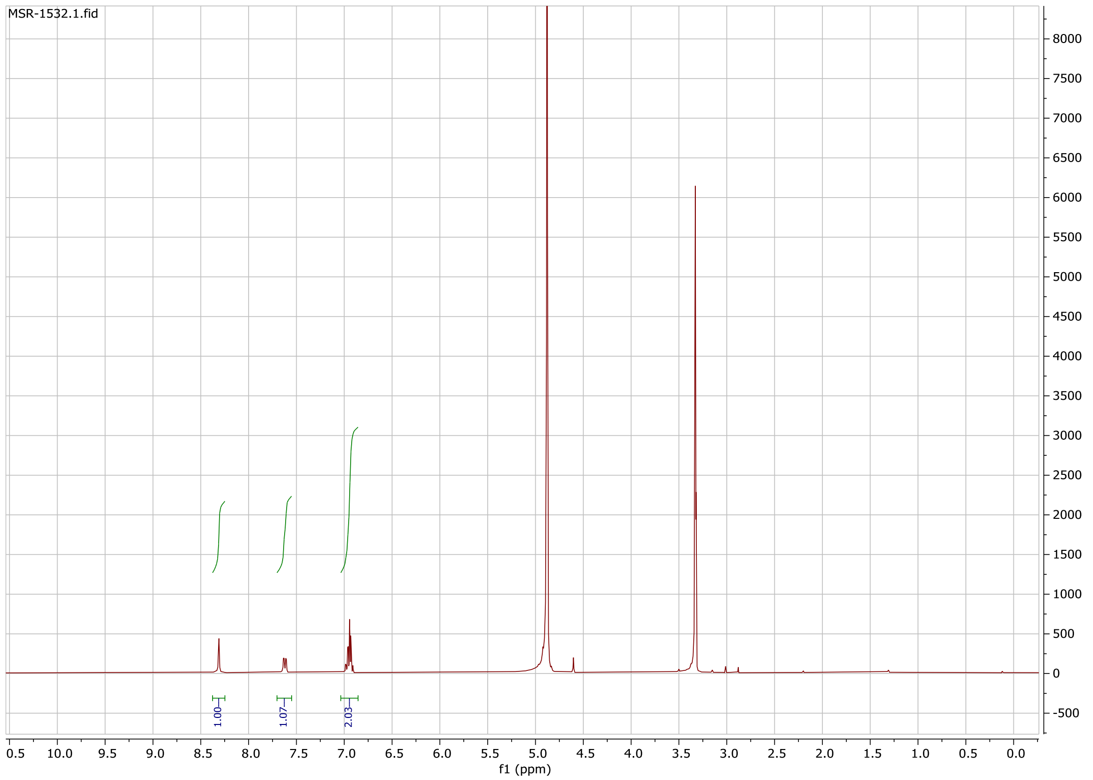

4-Fluoro-2-(1H-1,2,3-triazol-5-yl)phenol (34): ^{13}C NMR (101 MHz, CD_3OD)

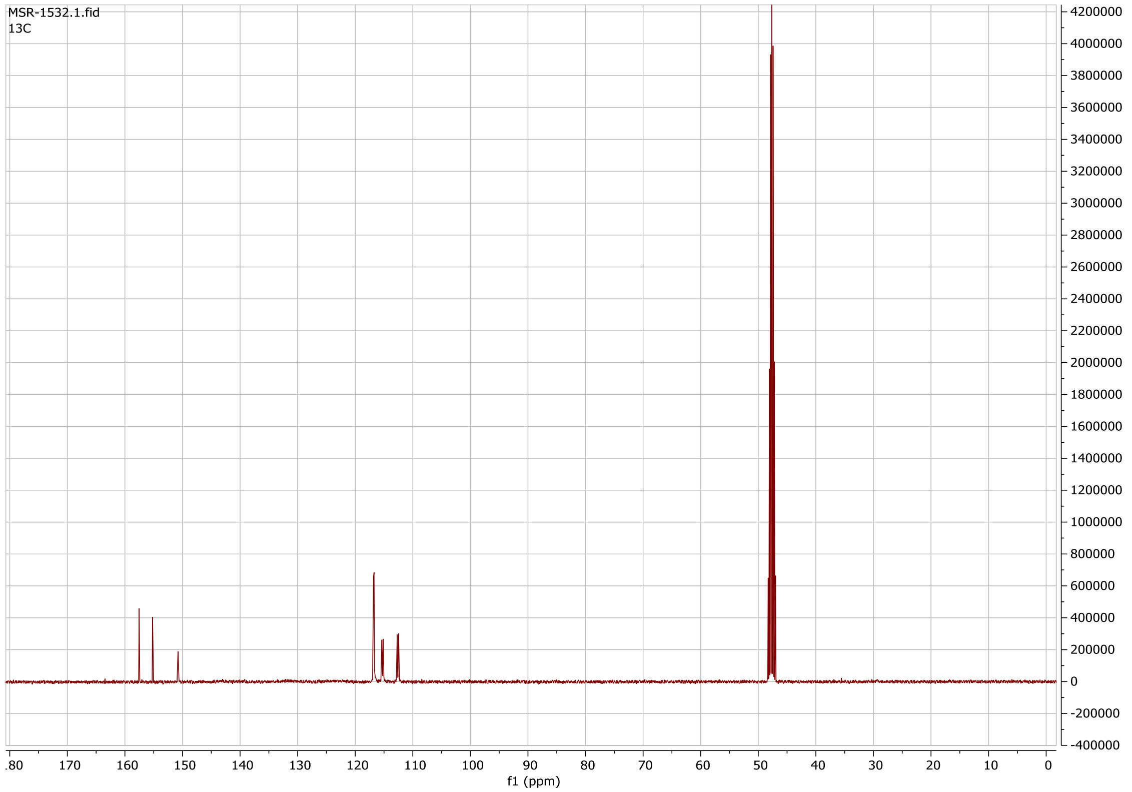

4-Methyl-2-(1H-1,2,3-triazol-5-yl)phenol (35): ^{1}H NMR (400 MHz, CD_3OD)

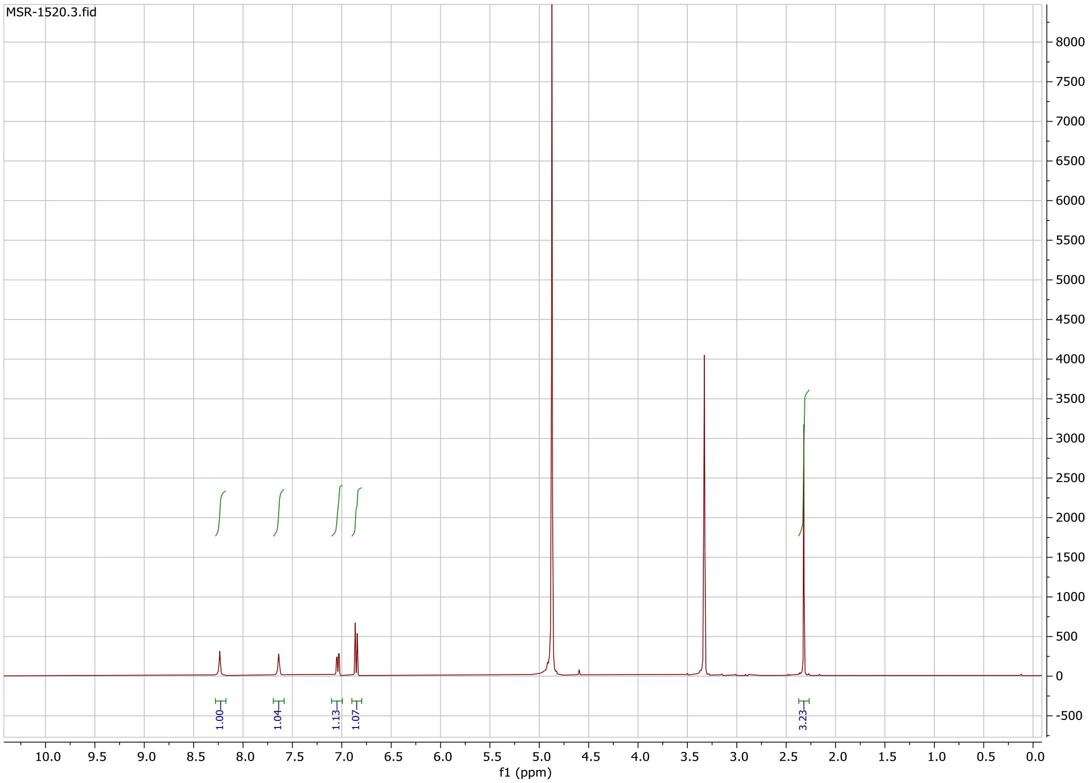

4-Methyl-2-(1H-1,2,3-triazol-5-yl)phenol (35): ^{13}C NMR (101 MHz, CD_3OD)

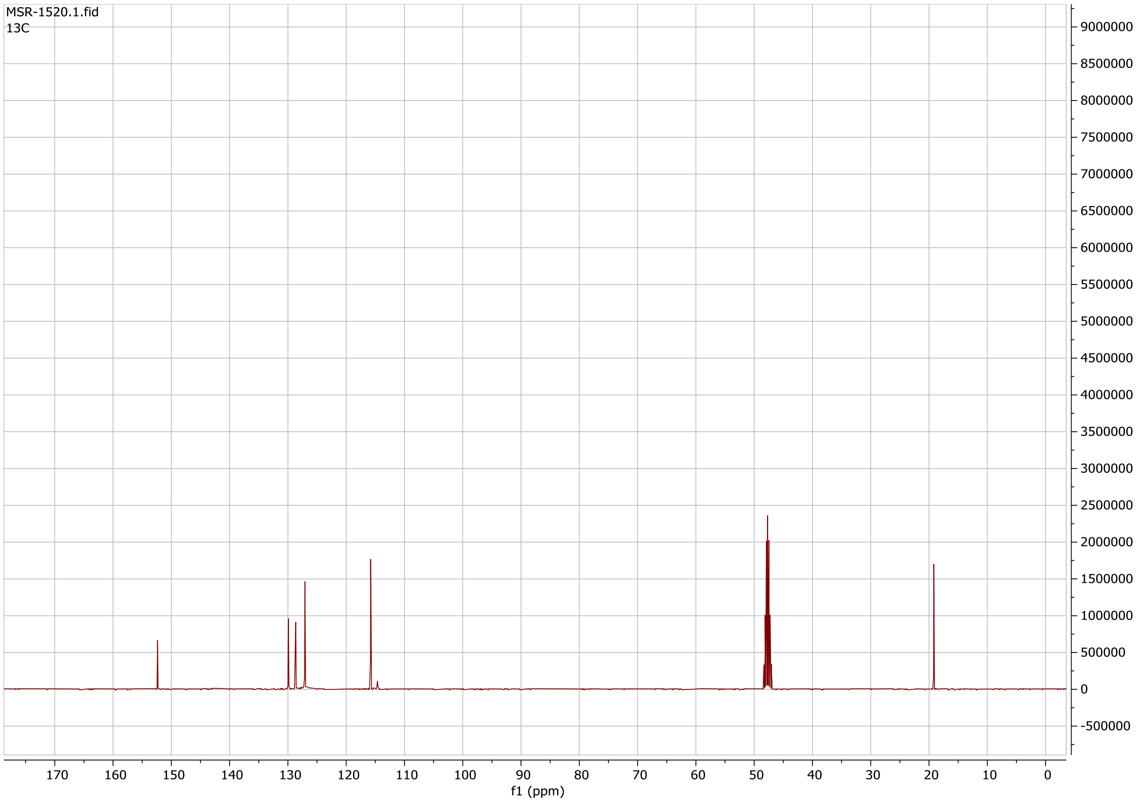

4-Ethyl-2-(1H-1,2,3-triazol-5-yl)phenol (36):^{1}H NMR (400 MHz, CD_3OD)

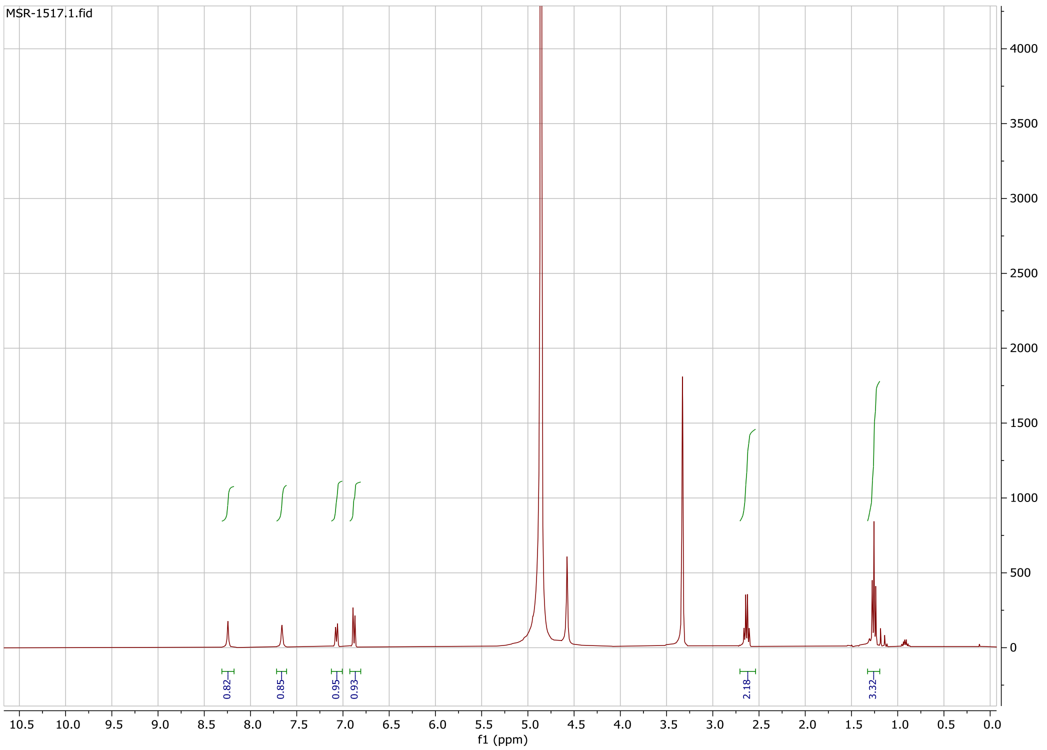

4-Ethyl-2-(1H-1,2,3-triazol-5-yl)phenol (36):^{13}C NMR (101 MHz, CD_3OD)

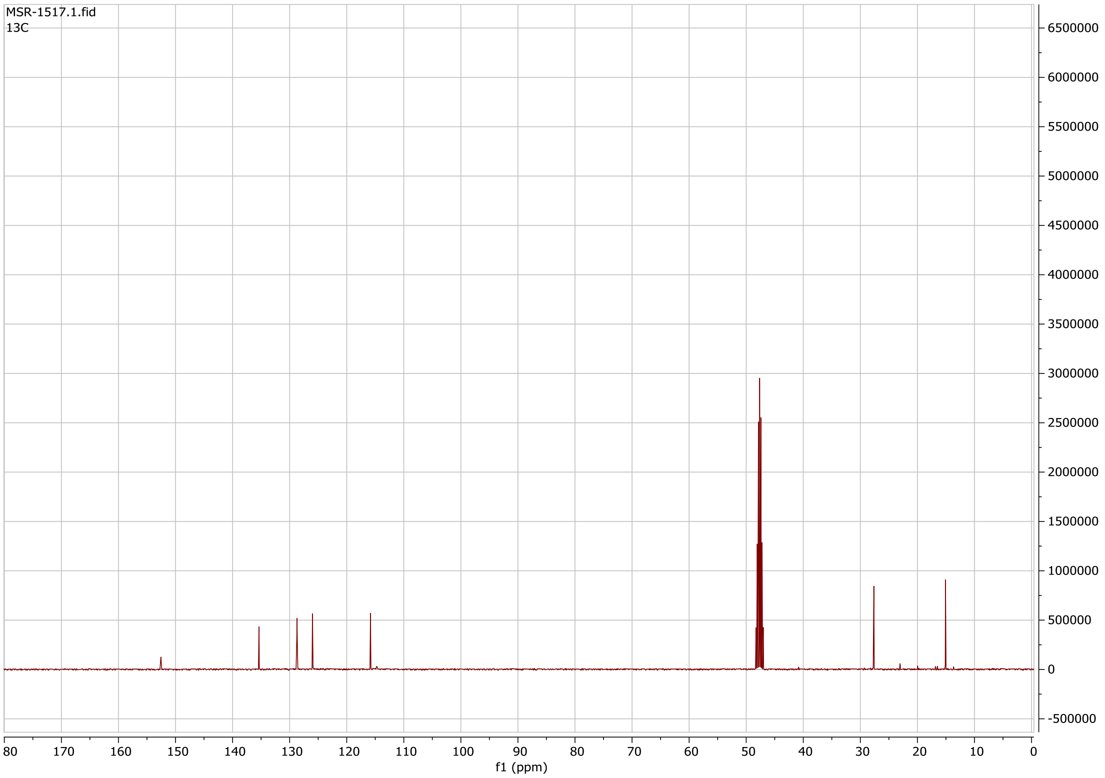

4-Propyl-2-(1H-1,2,3-triazol-5-yl)phenol (37): ^{1}H NMR (400 MHz, CD_3OD)

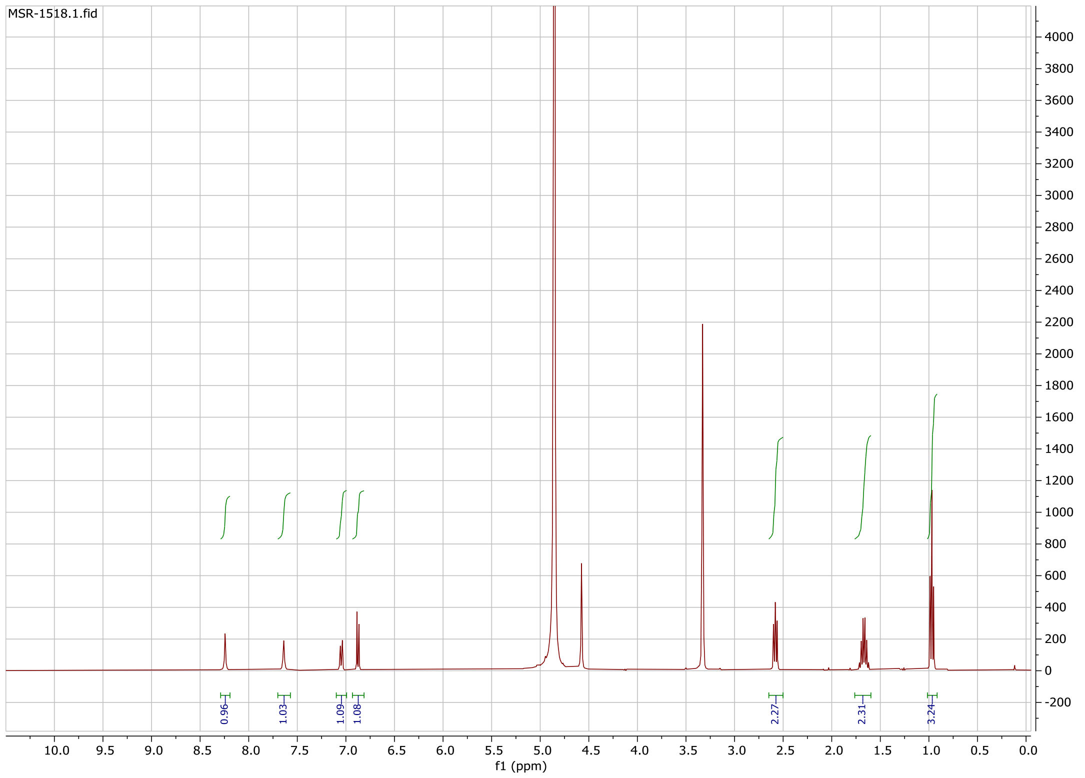

4-Propyl-2-(1H-1,2,3-triazol-5-yl)phenol (37): ^{13}C NMR (101 MHz, CD_3OD)

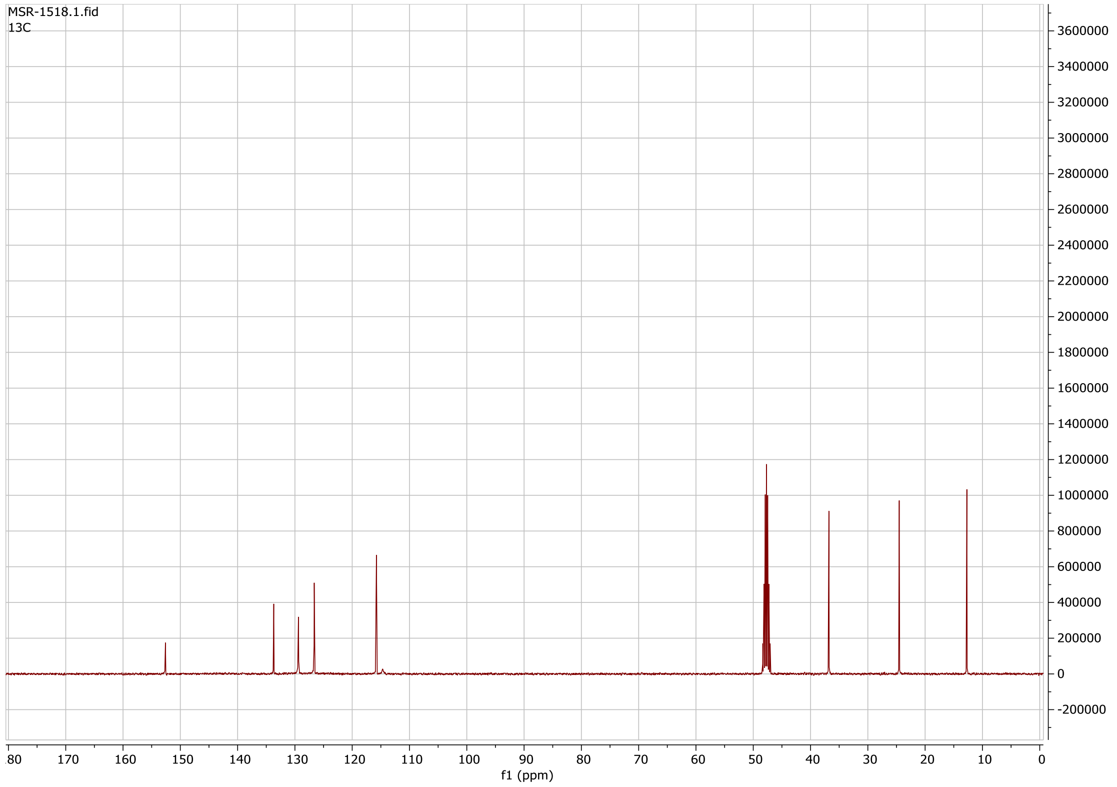

4-Benzyl-2-(1H-1,2,3-triazol-5-yl)phenol (38): ^{1}H NMR (400 MHz, CD_3OD)

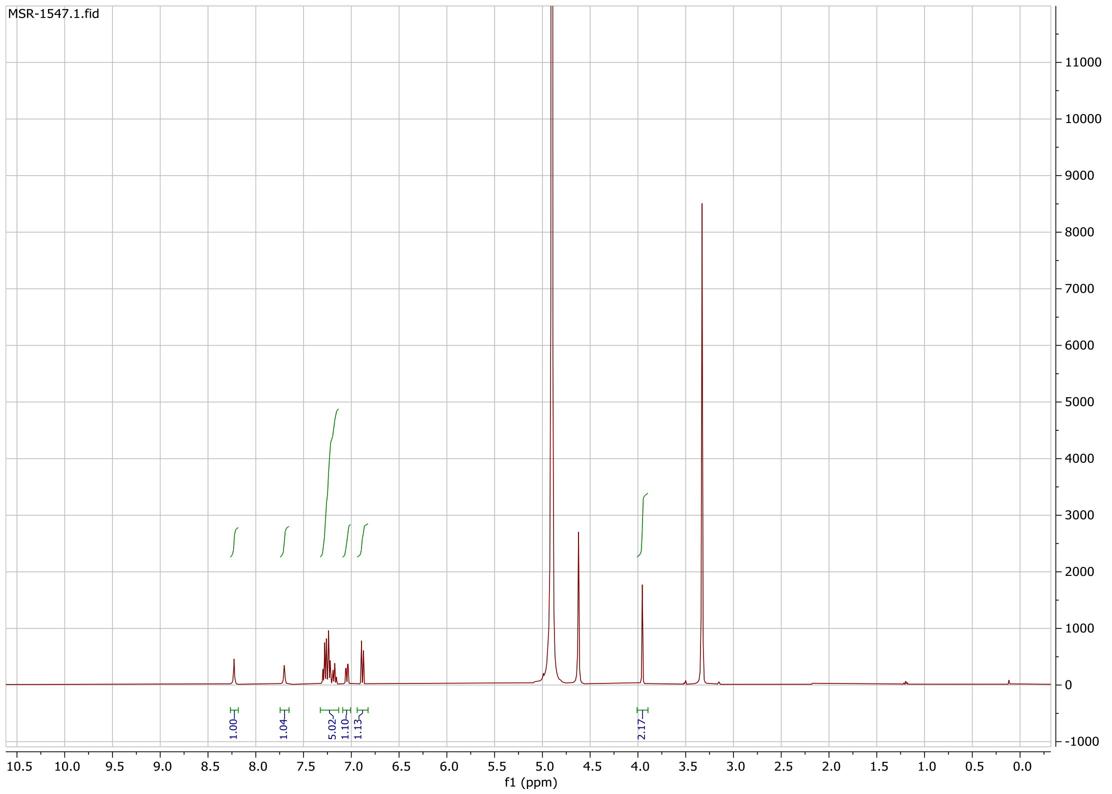

4-Benzyl-2-(1H-1,2,3-triazol-5-yl)phenol (38): ^{13}C NMR (101 MHz, CD_3OD)

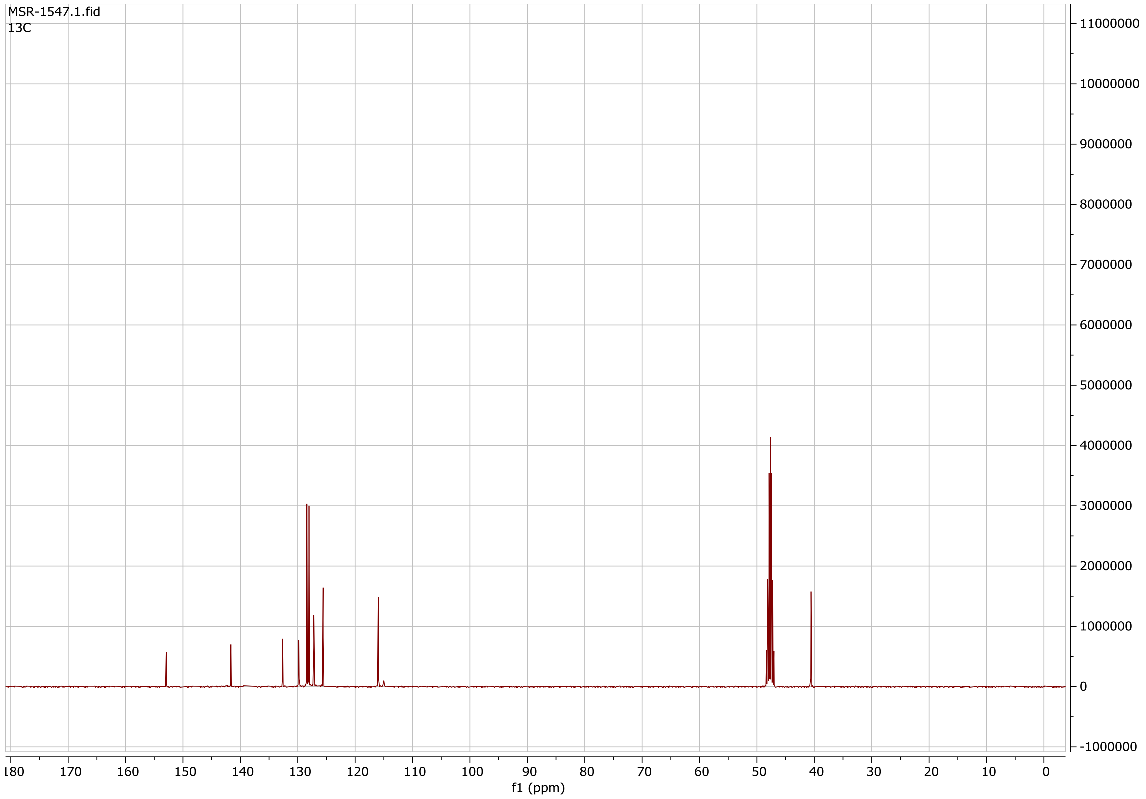

4-Phenylethyl-2-(1H-1,2,3-triazol-5-yl)phenol (39): ^{1}H NMR (400 MHz, CD_3OD)

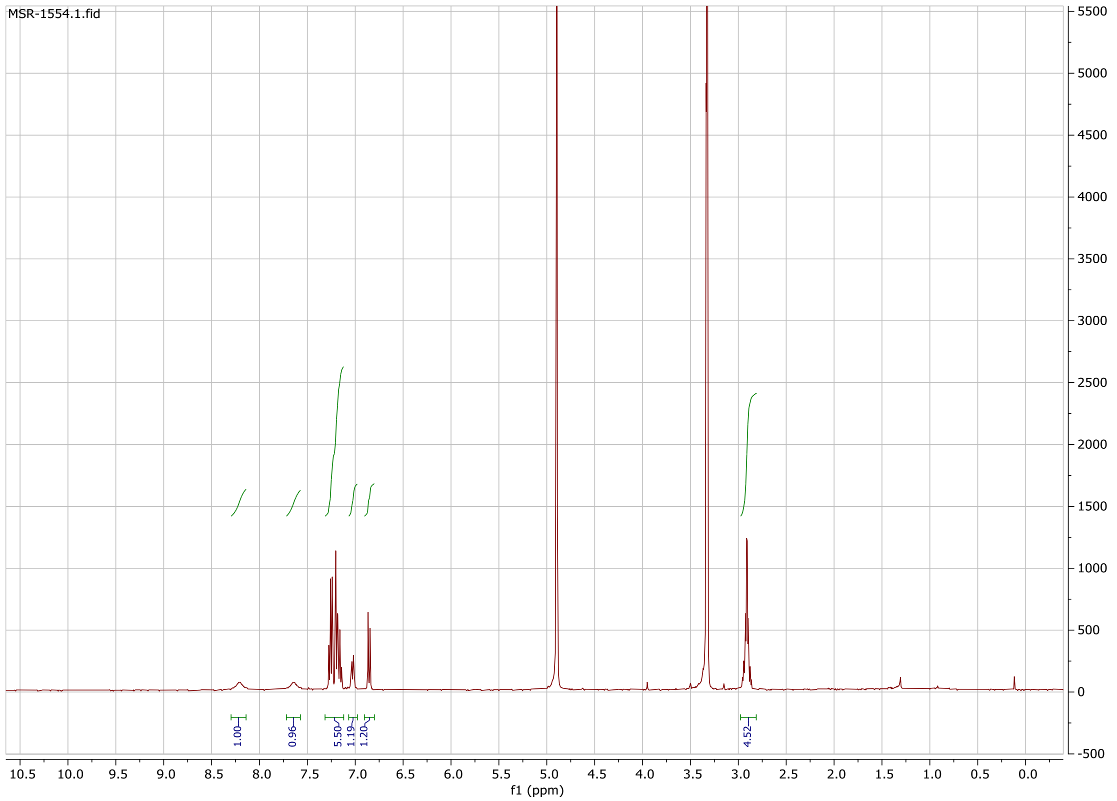

4-Phenylethyl-2-(1H-1,2,3-triazol-5-yl)phenol (39): ^{13}C NMR (101 MHz, CD_3OD)

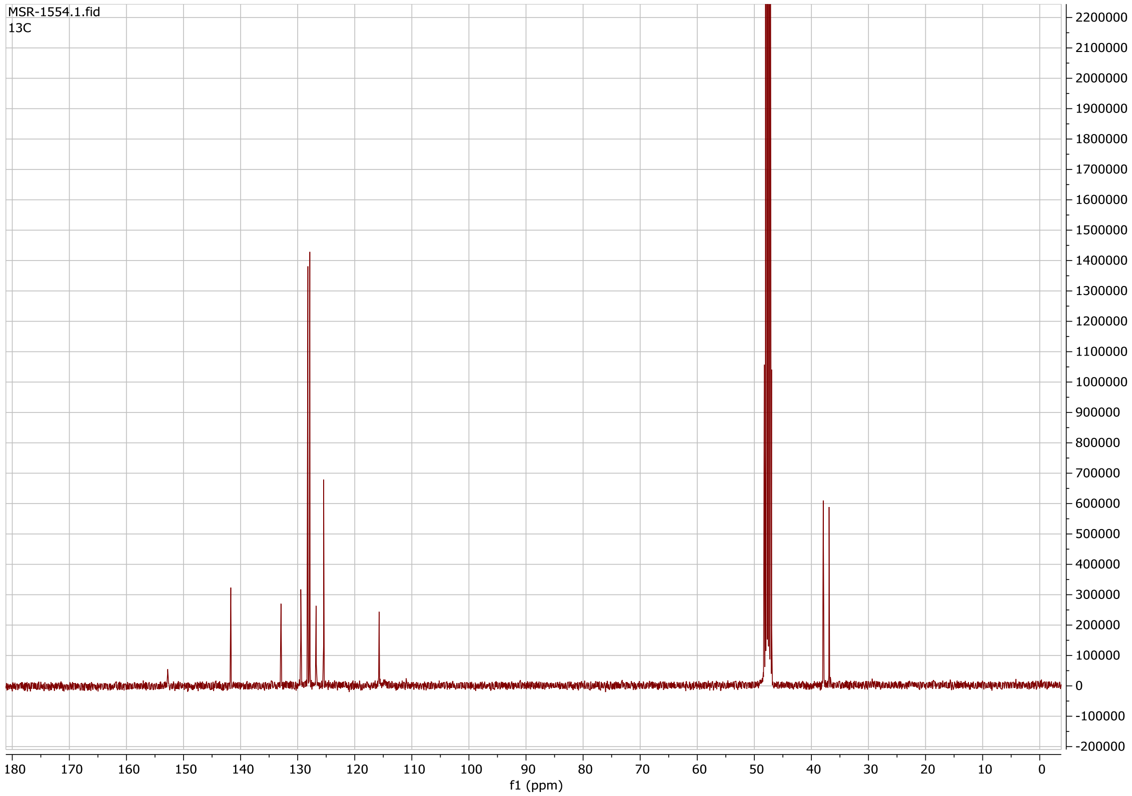

4-(3-Chloro-2-phenethoxyphenyl)-1,2,3-triazole (43) : ^{1}H NMR (400 MHz, $CDCl_3$)

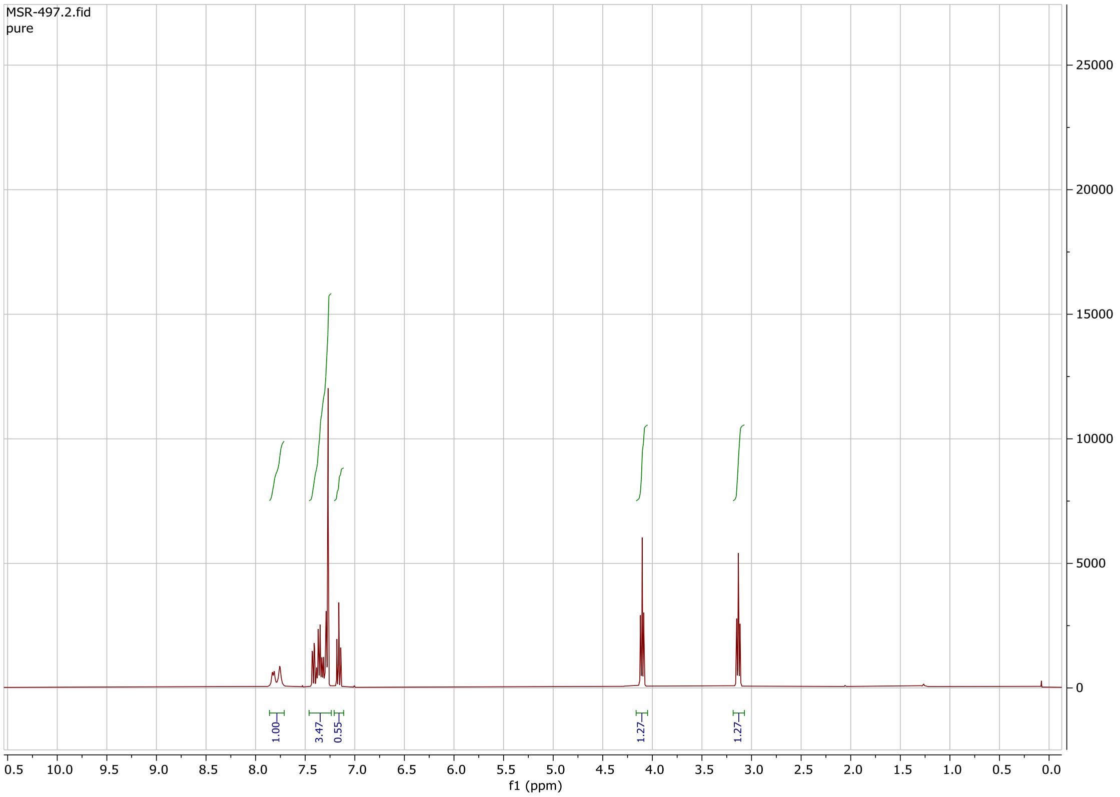

4-(3-Chloro-2-phenethoxyphenyl)-1,2,3-triazole (43) : ^{13}C NMR (101 MHz, CD_3OD)

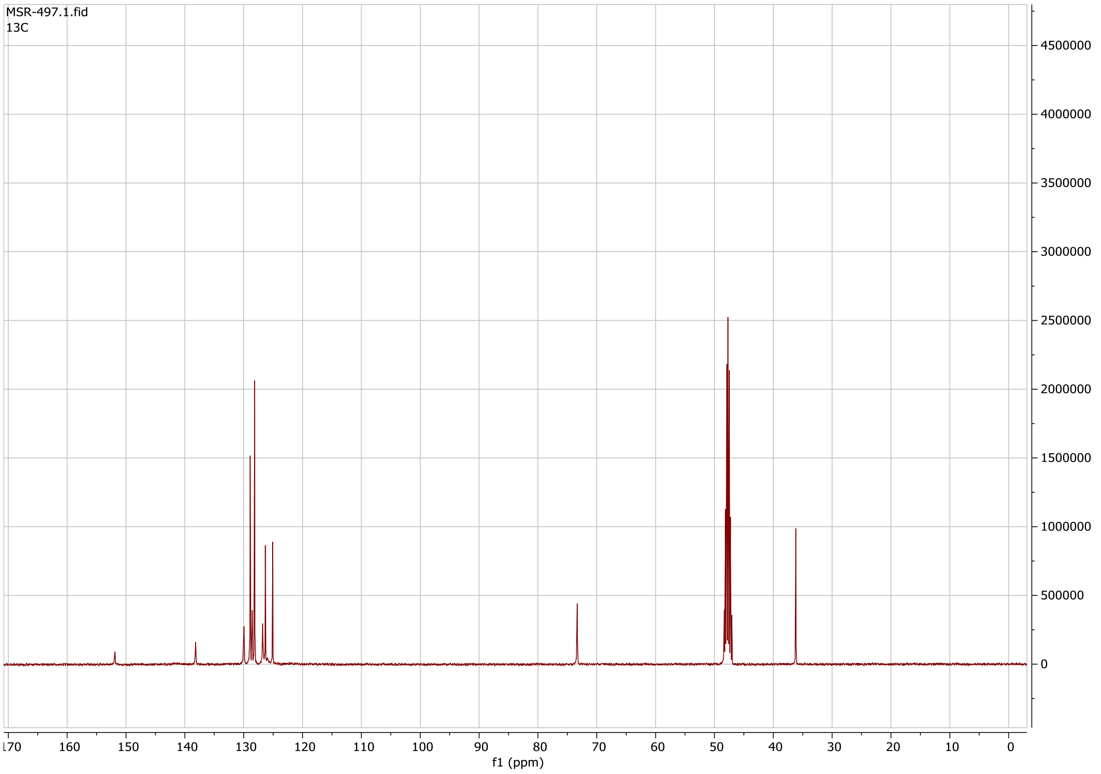

2-(1H-1,2,3-triazol-5-yl)benzene-1,3-diol (44) : ^{1}H NMR (400 MHz, CD_3OD)

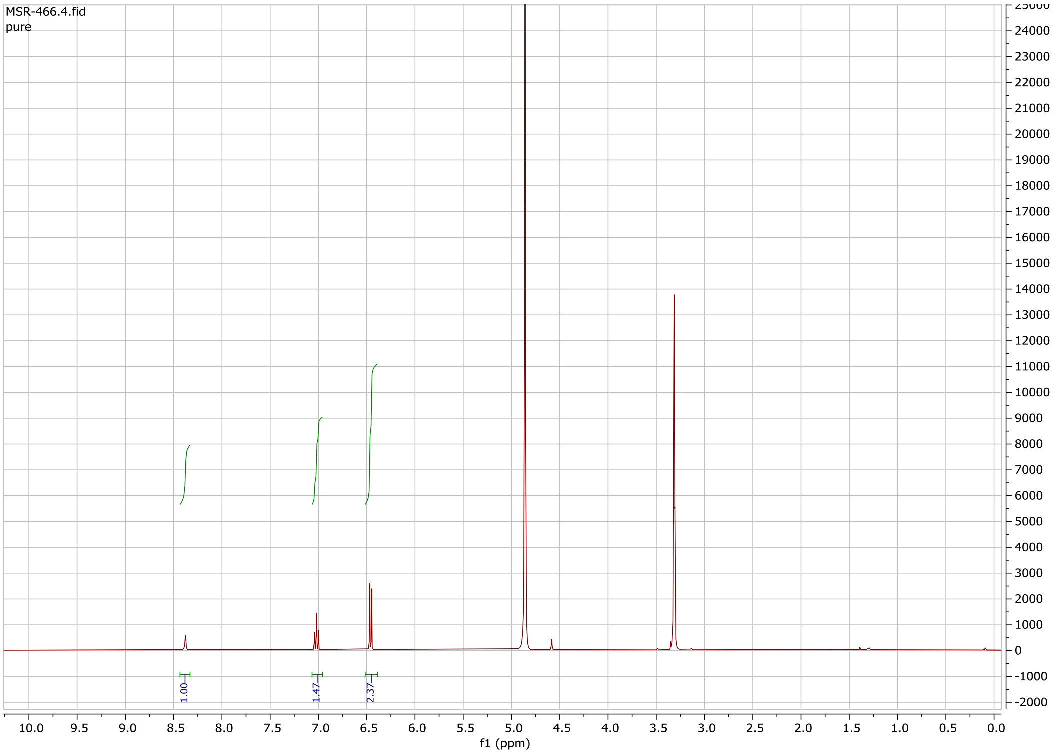

2-(1H-1,2,3-triazol-5-yl)benzene-1,3-diol (44) : ^{13}C NMR (101 MHz, CD_3OD)

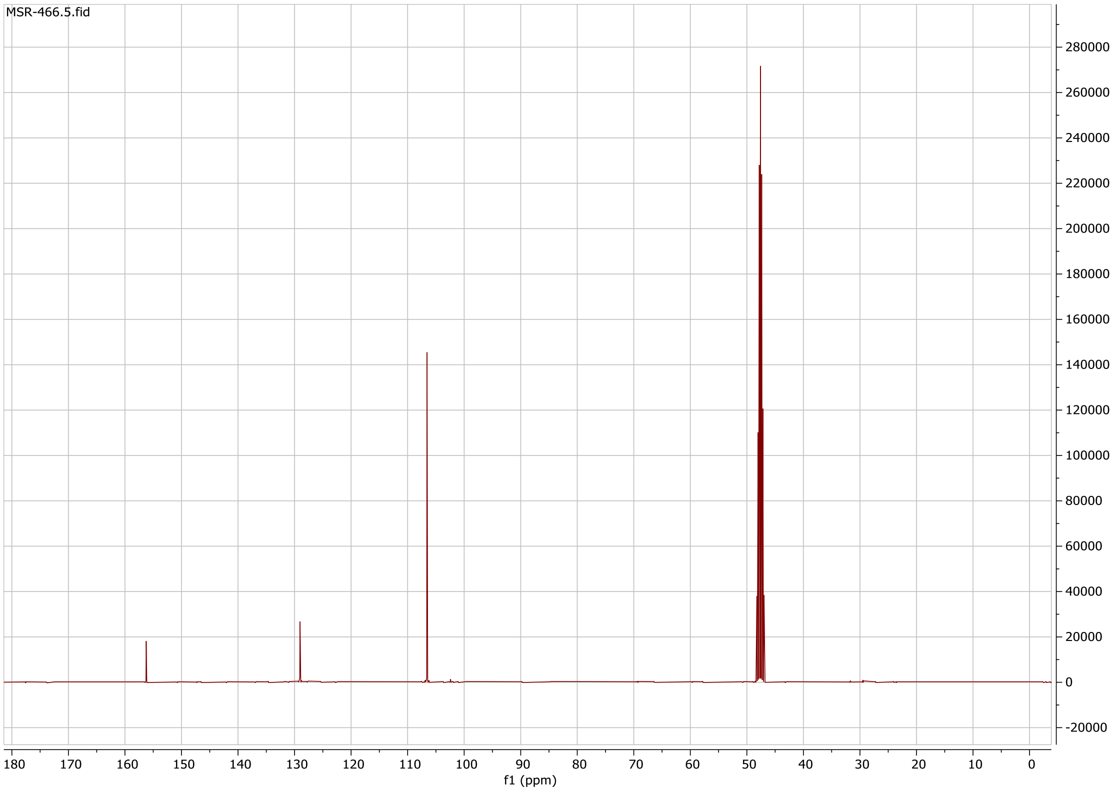

4-(2,6-Dimethoxyphenyl)-1,2,3-triazole (45) : ^{1}H NMR (400 MHz, $CDCl_3$)

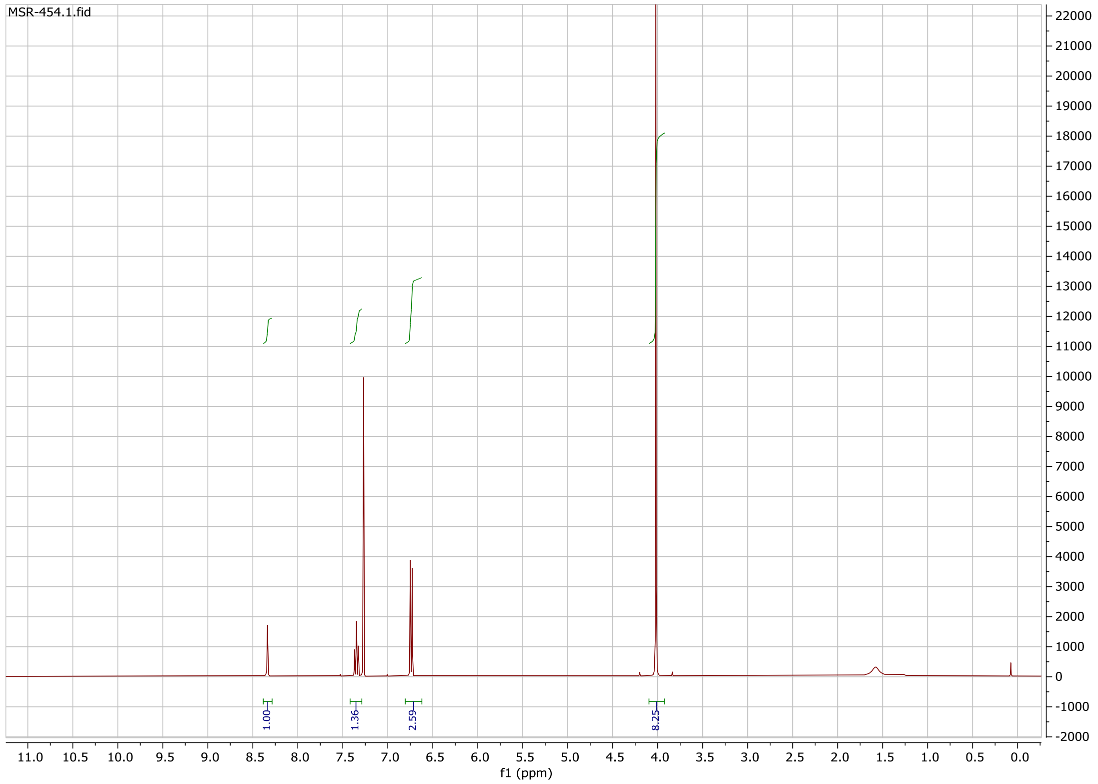

4-(2,6-Dimethoxyphenyl)-1,2,3-triazole (45) : ^{13}C NMR (101 MHz, CD_3OD)

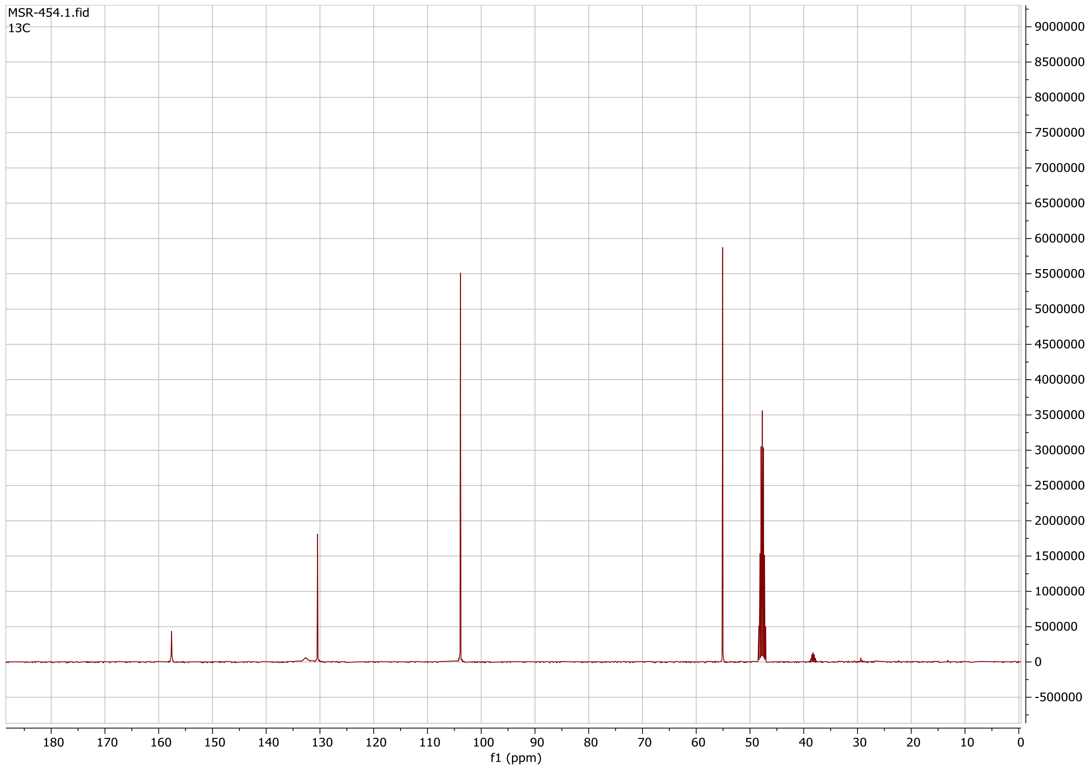

4-(2,4-Dichlorophenyl)-1,2,3-triazole (48):^{1}H NMR (400 MHz, $CDCl_3$)

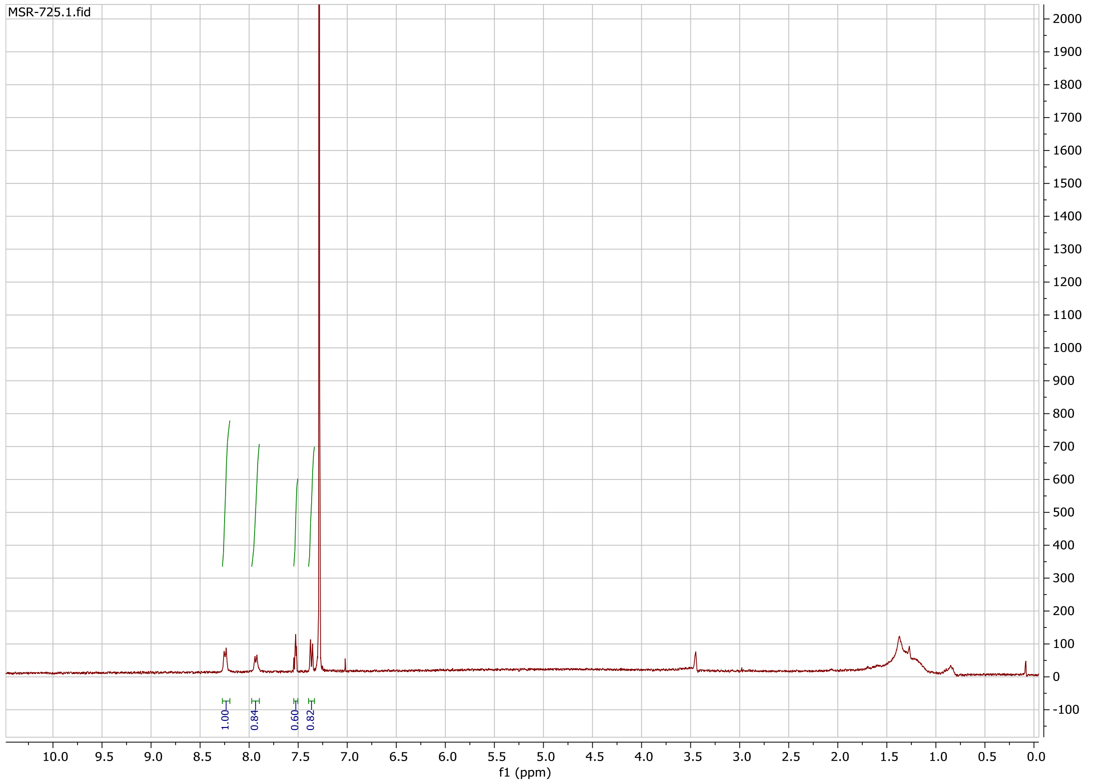

4-(2,4-Dichlorophenyl)-1,2,3-triazole (48):^{13}C NMR (101 MHz, CD_3OD)

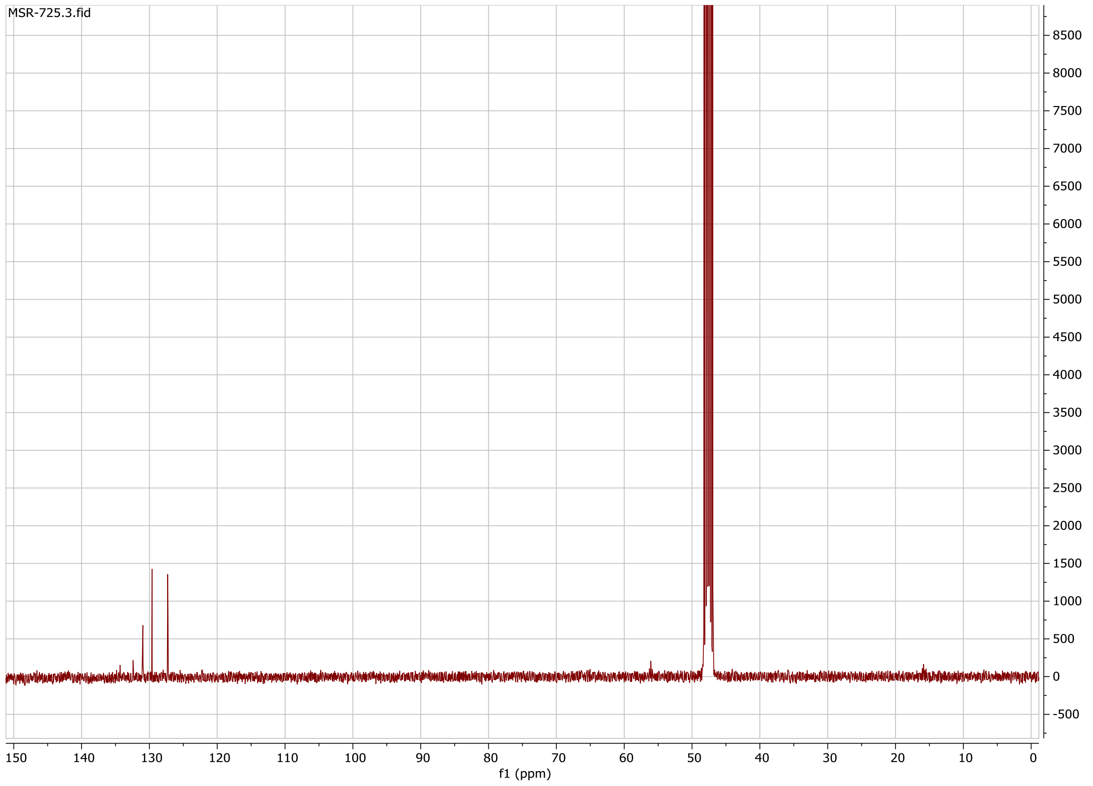

4-(3,4-Dichlorophenyl)-1,2,3-triazole (49): ^{1}H NMR (400 MHz, CD_3OD)

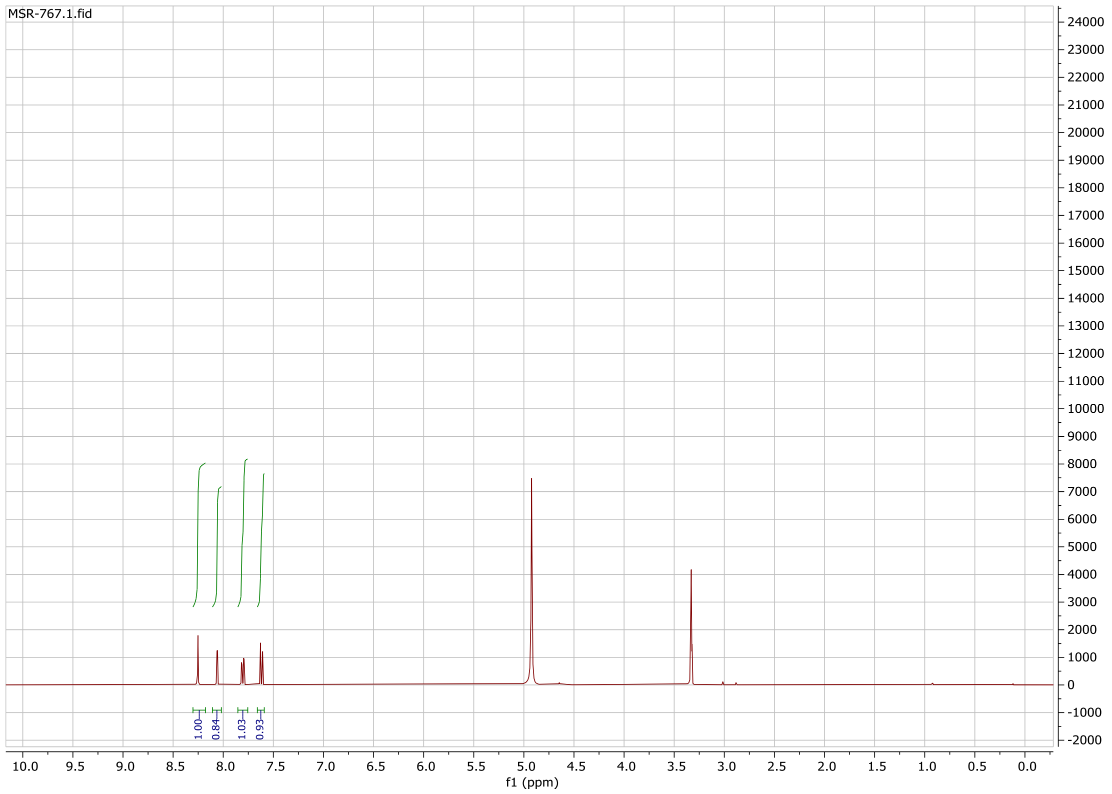

4-(3,4-Dichlorophenyl)-1,2,3-triazole (49): ^{13}C NMR (101 MHz, CD_3OD)

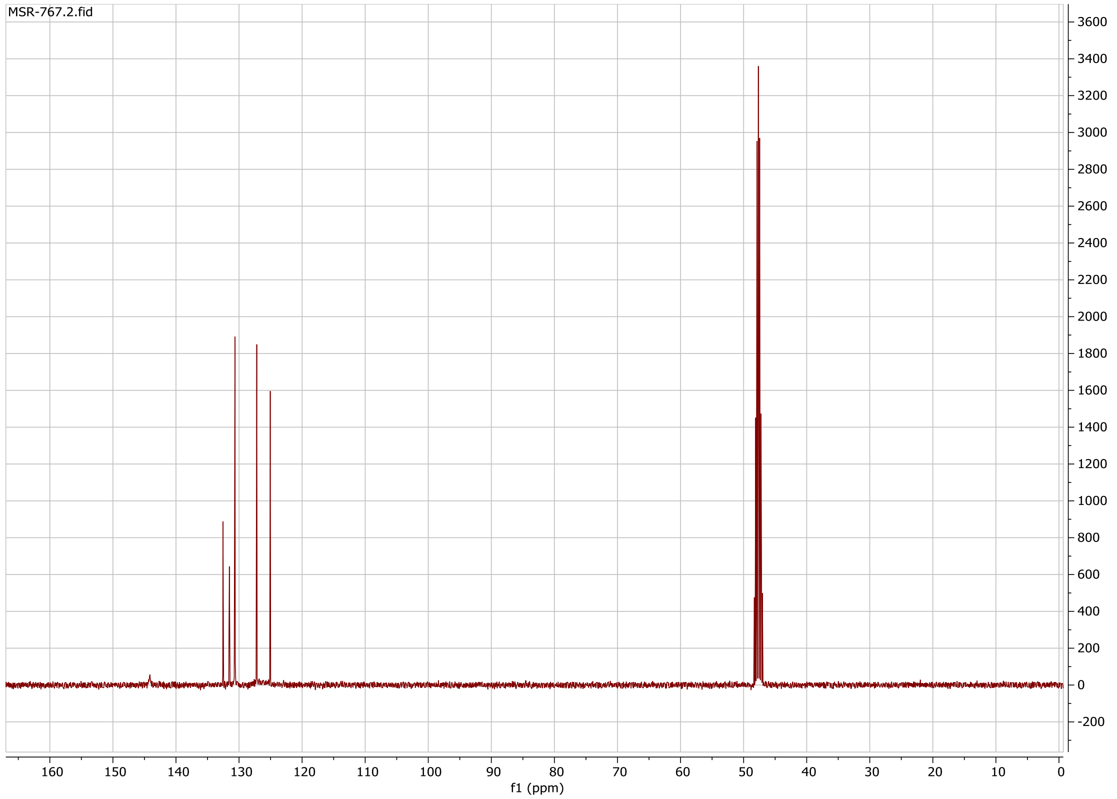

4-(3,5-Dichlorophenyl)-1,2,3-triazole (50) : ^{1}H NMR (400 MHz, CD_3OD)

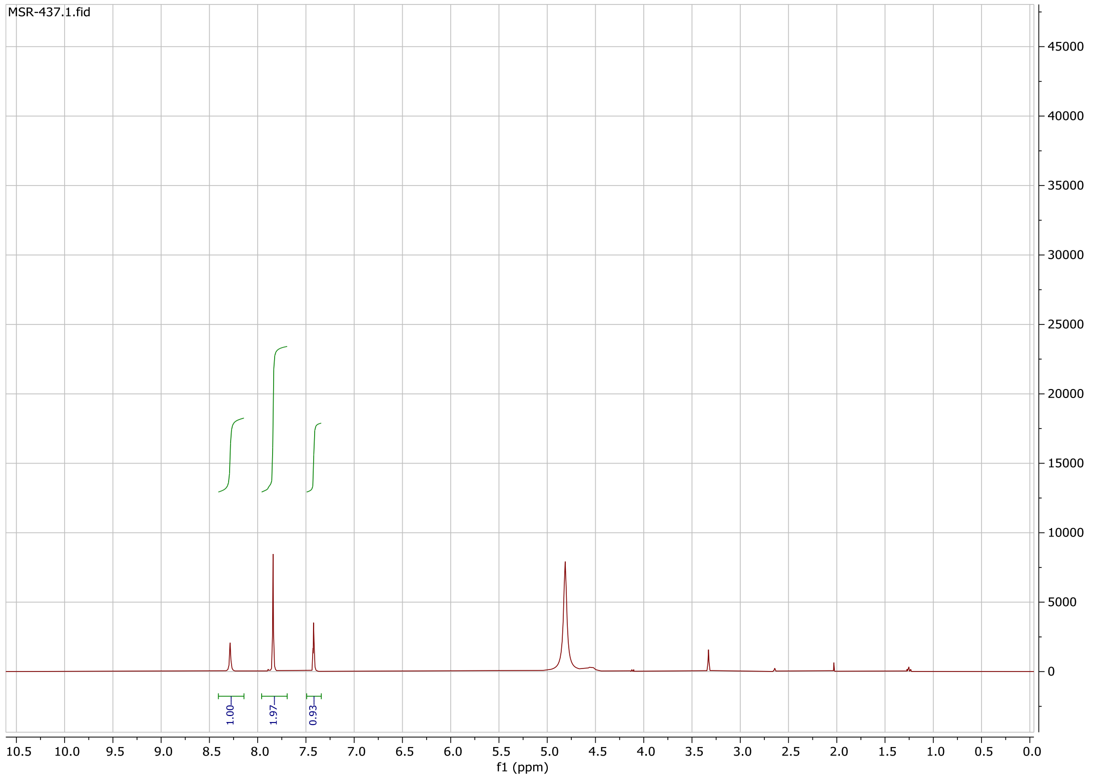

4-(3,5-Dichlorophenyl)-1,2,3-triazole (50) : ^{13}C NMR (101 MHz, CD_3OD)

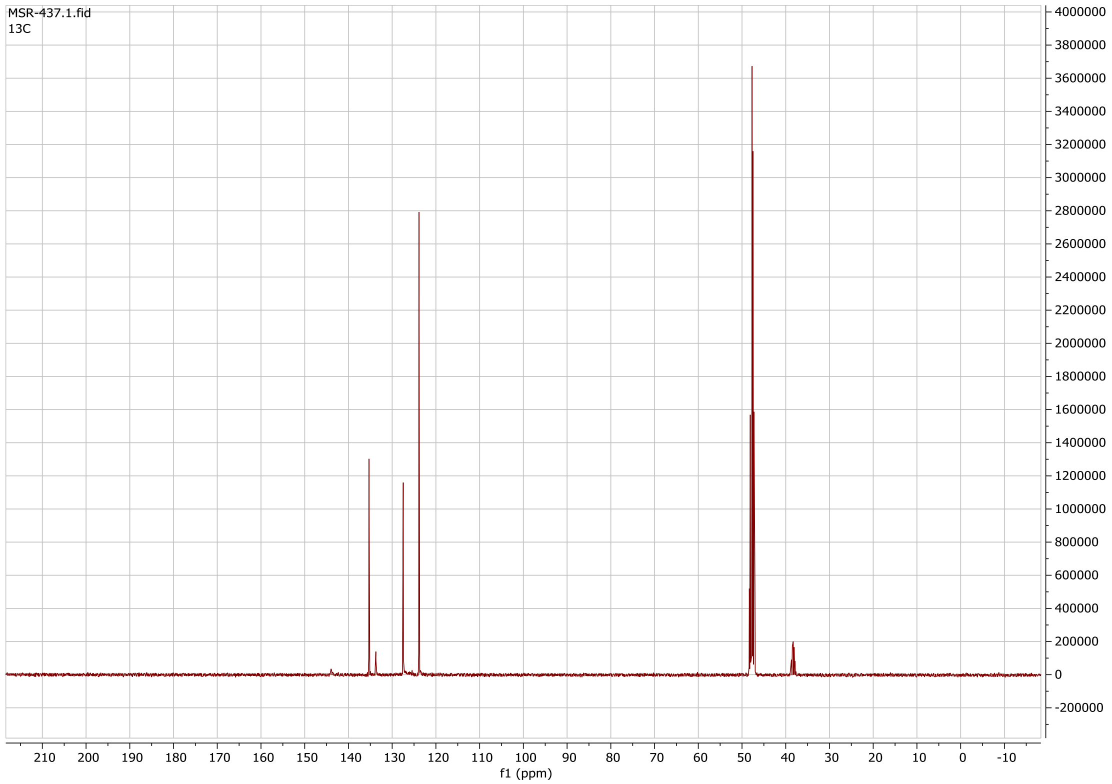

4-(2,6-Dichlorophenyl)-1,2,3-triazole (51): ^{1}H NMR (400 MHz, CD_3OD)

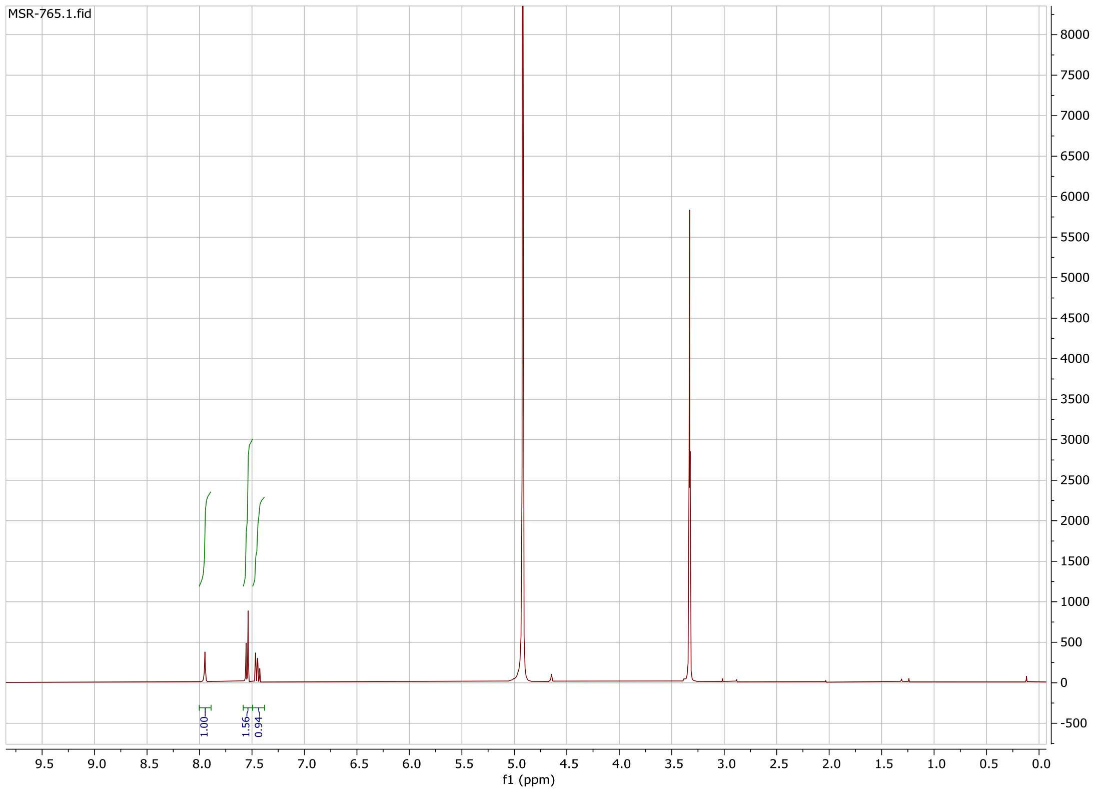

4-(2,6-Dichlorophenyl)-1,2,3-triazole (51): ^{13}C NMR (101 MHz, CD_3OD)

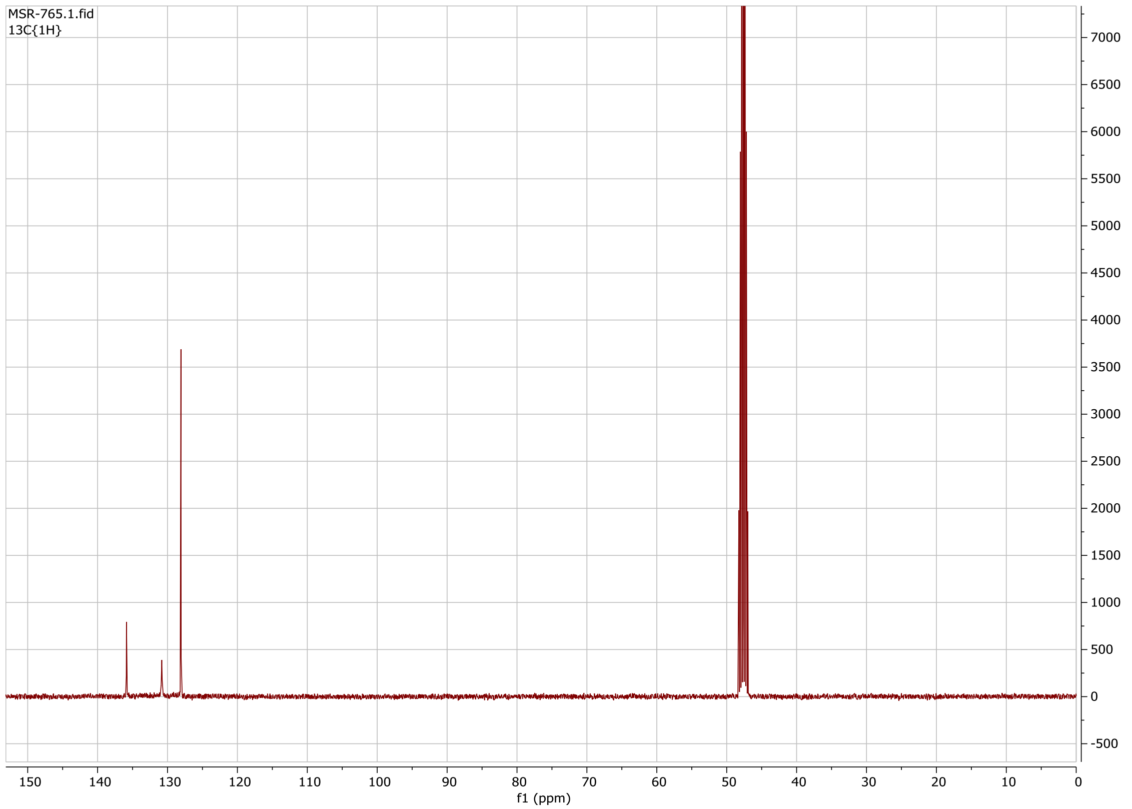

4-(2,4,5-Trichlorophenyl)-1,2,3-triazole (52):^{1}H NMR (400 MHz, $CDCl_3$)

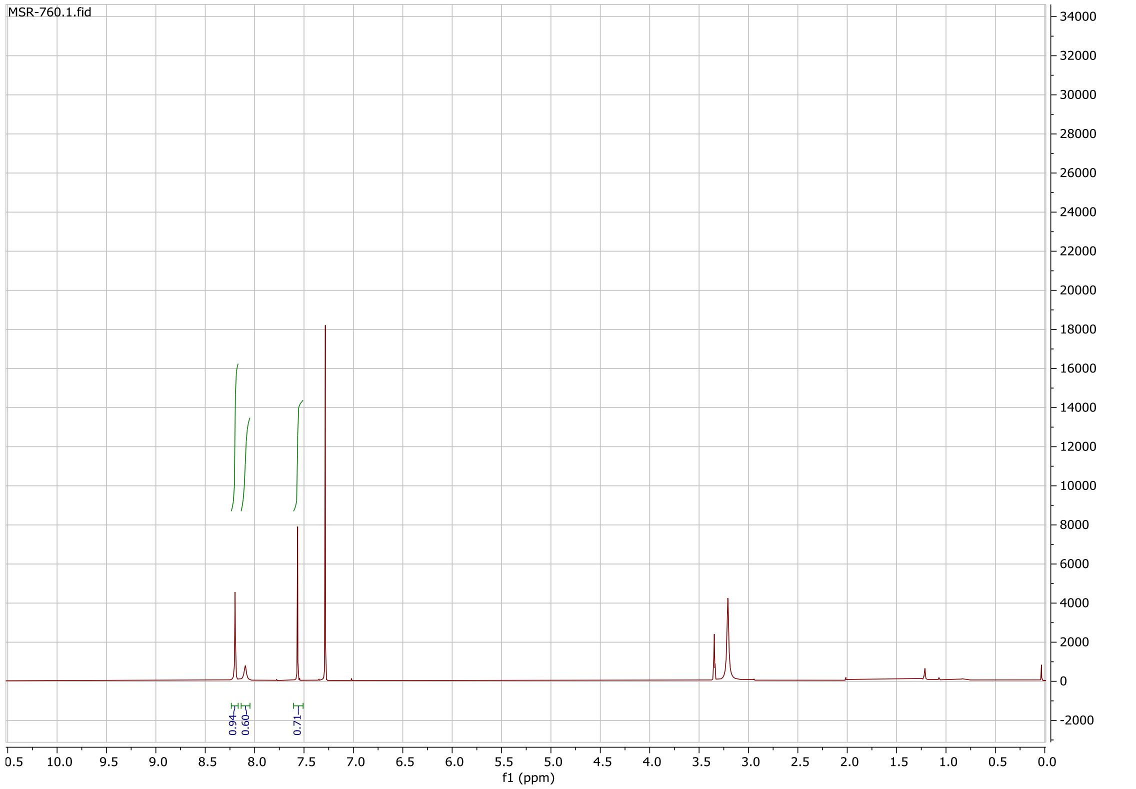

4-(2,4,5-Trichlorophenyl)-1,2,3-triazole (52):^{13}C NMR (101 MHz, CD_3OD)

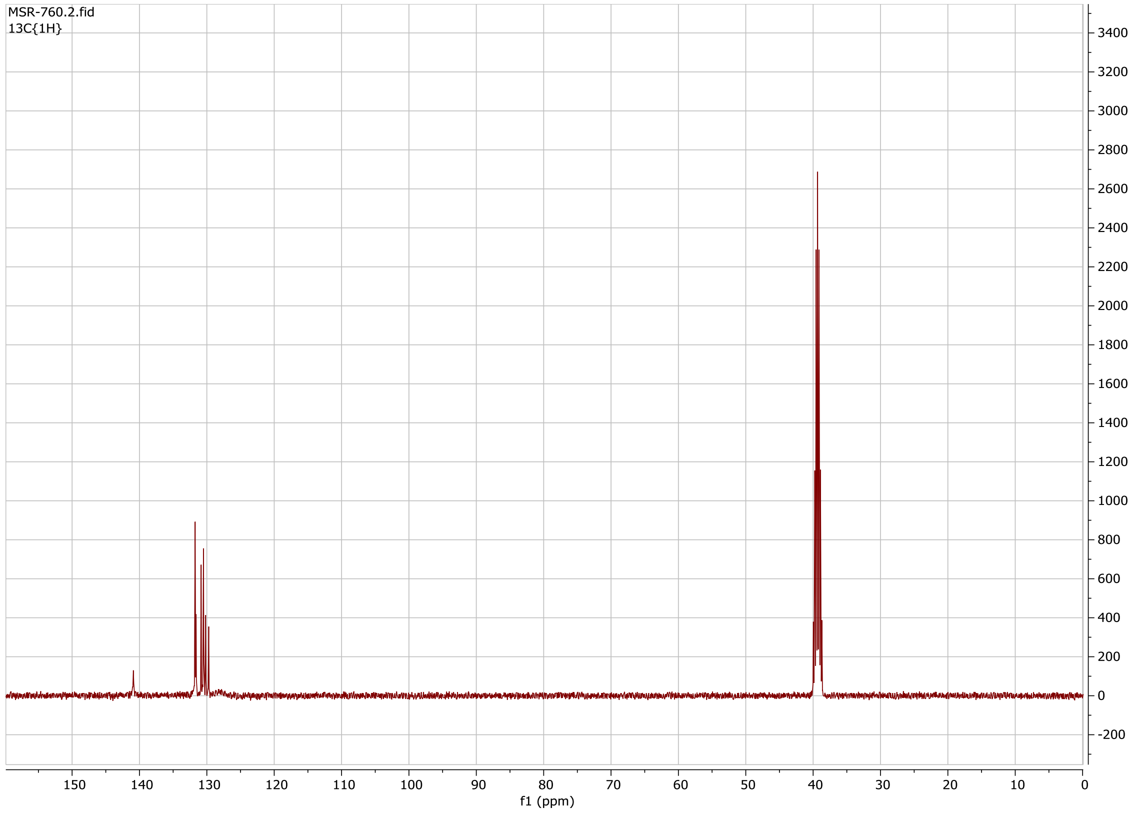

4-(2,4,6-Trichlorophenyl)-1,2,3-triazole (53):^{1}H NMR (400 MHz, CD_3OD)

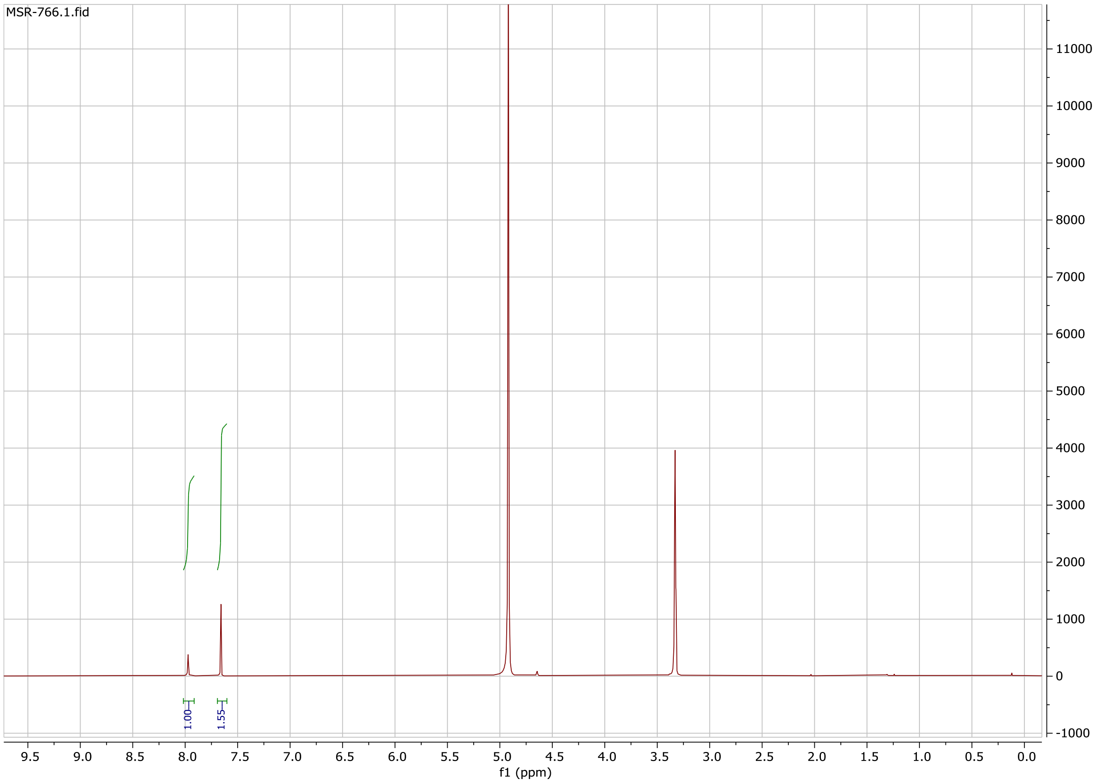

4-(2,4,6-Trichlorophenyl)-1,2,3-triazole (53):^{13}C NMR (101 MHz, CD_3OD)

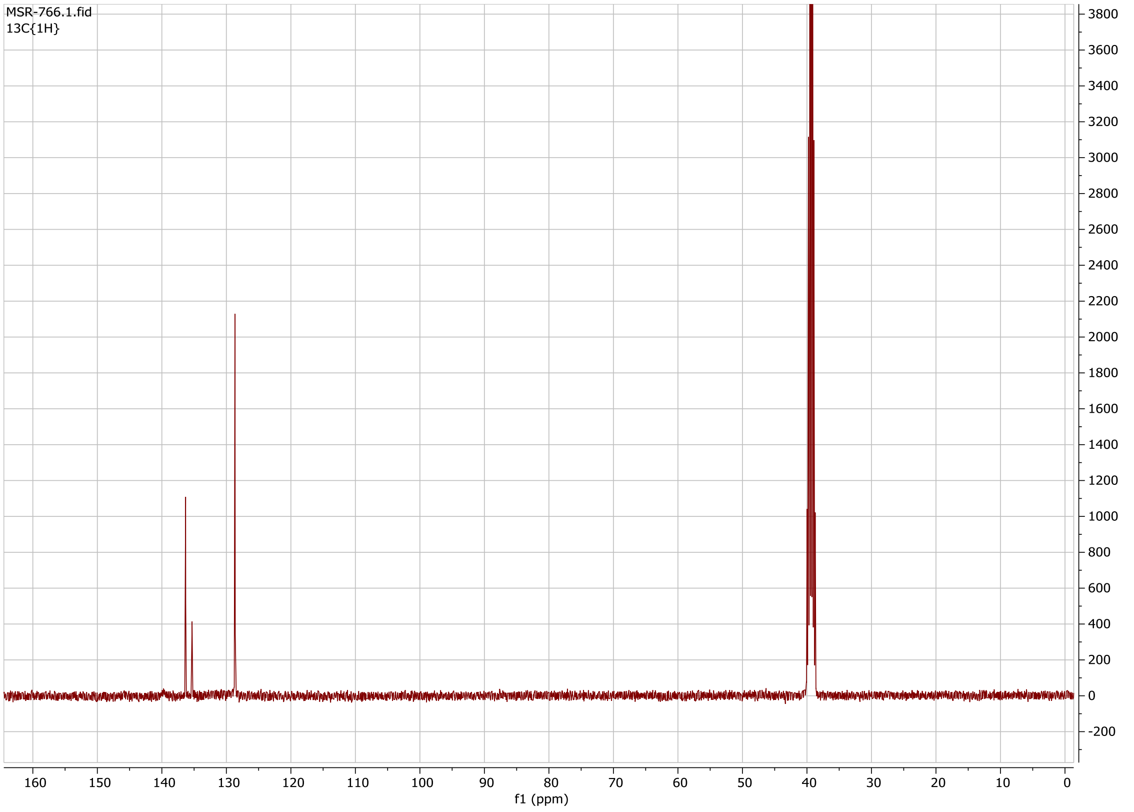

4-Phenyl-1*H*-1,2,3-triazole-5-carbonitrile (62) : ^{1}H NMR (400 MHz, CD_3OD)

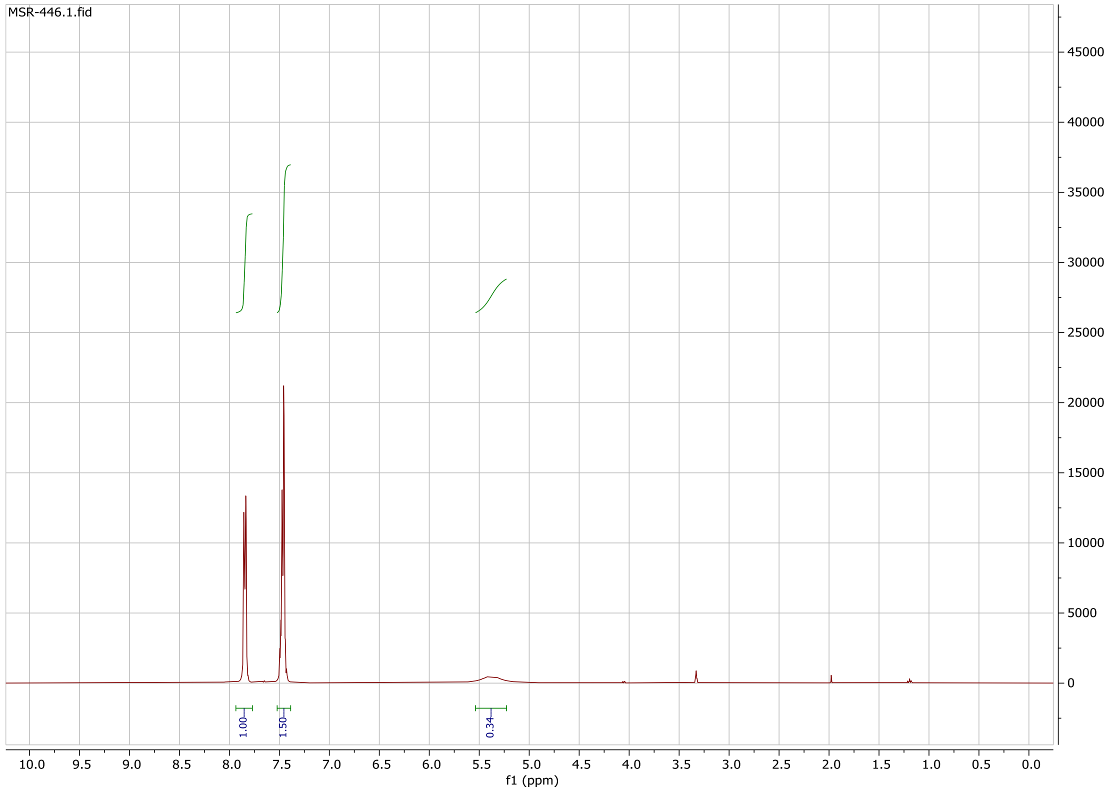

4-Phenyl-1*H*-1,2,3-triazole-5-carbonitrile (62) : ^{13}C NMR (101 MHz, CD_3OD)

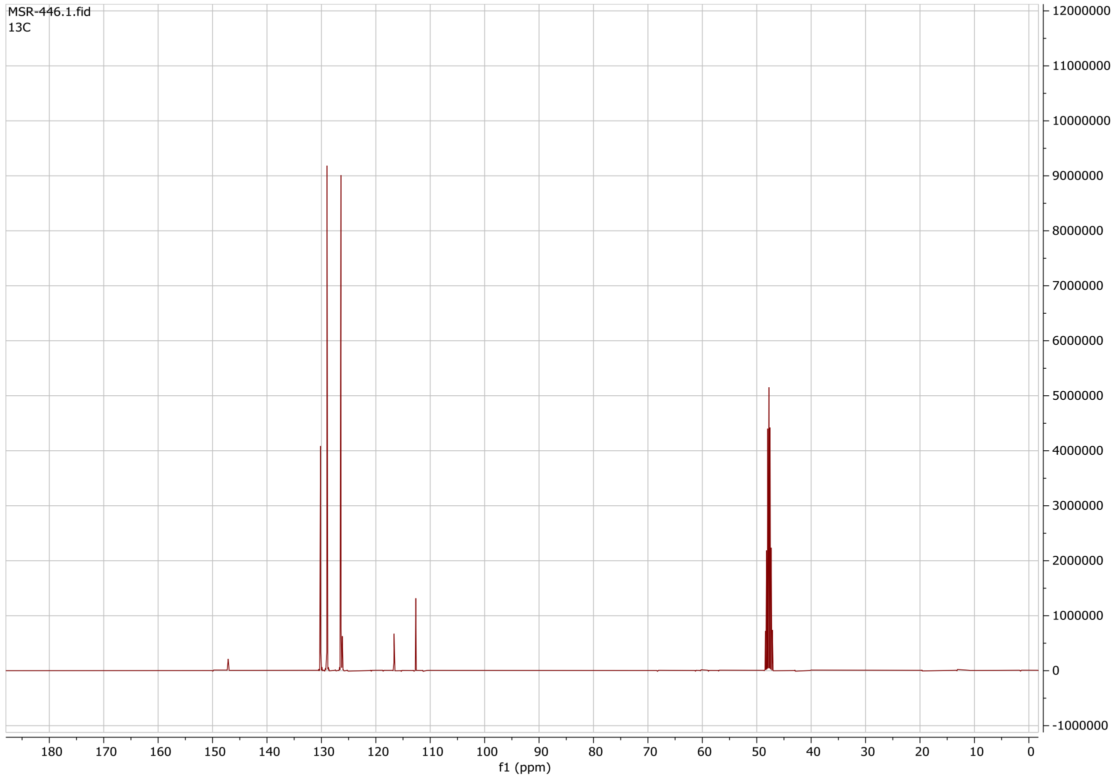

4-(3-Chlorophenyl)-5-methyl-1,2,3-triazole (63) : ^{1}H NMR (400 MHz, $CDCl_3$)

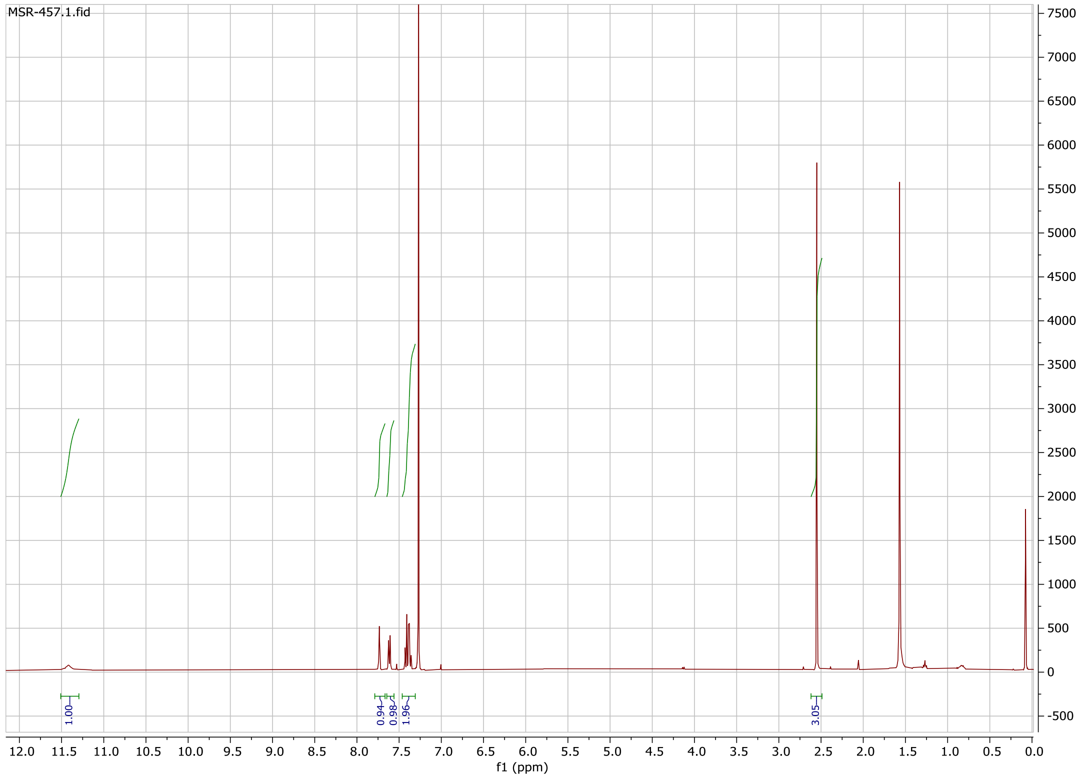

4-(3-Chlorophenyl)-5-methyl-1,2,3-triazole (63) : ^{13}C NMR (101 MHz, CD_3OD)

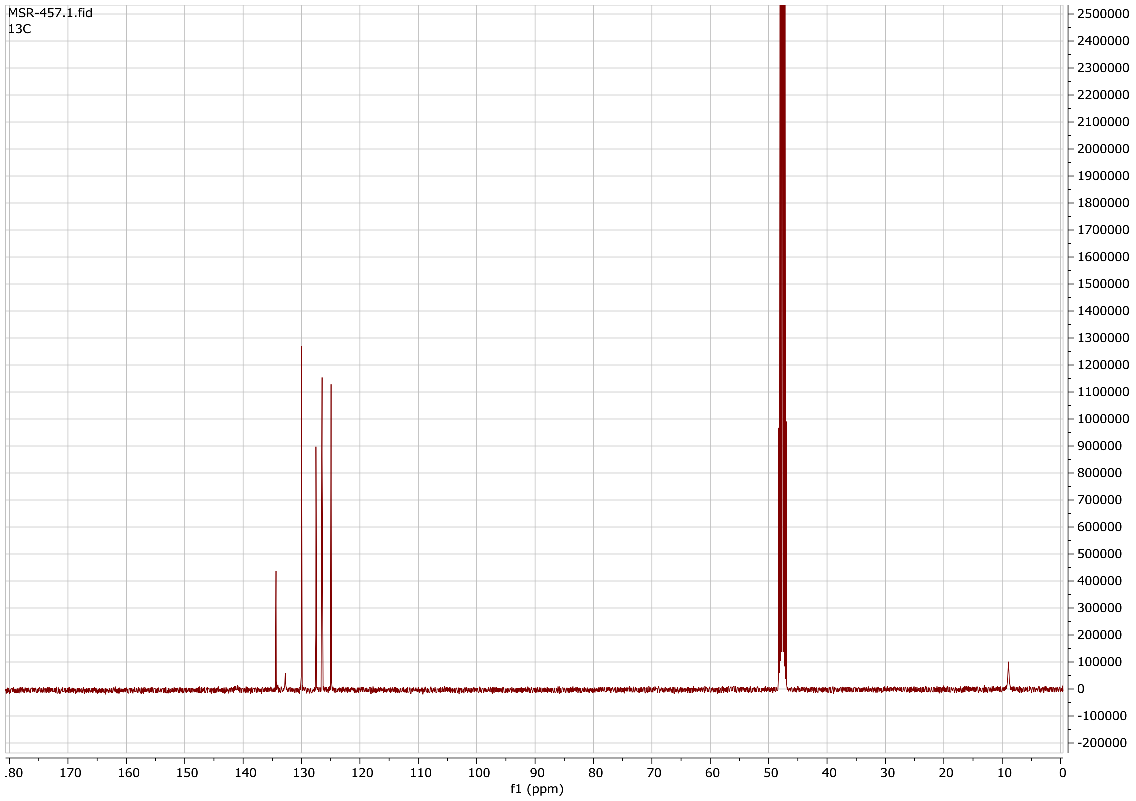

4,5-Bis(3-chlorophenyl)-1,2,3-triazole (64):^{1}H NMR (400 MHz, $CDCl_3$)

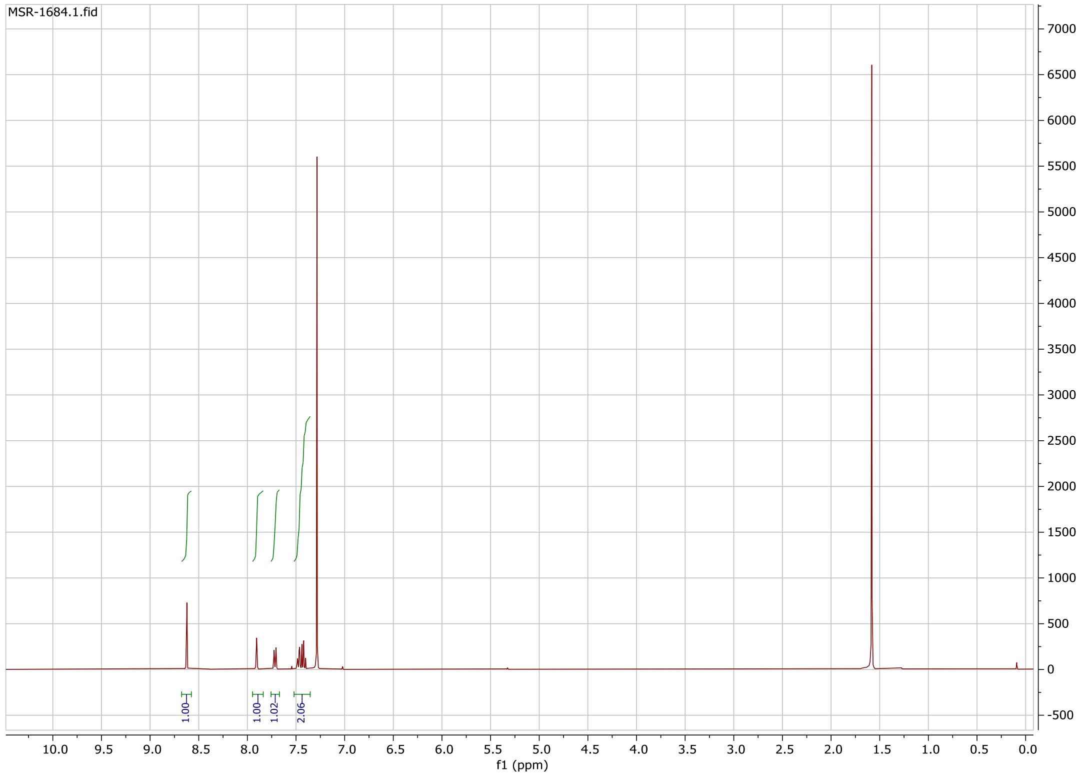

4,5-Bis(3-chlorophenyl)-1,2,3-triazole (64):^{13}C NMR (101 MHz, CD_3OD)

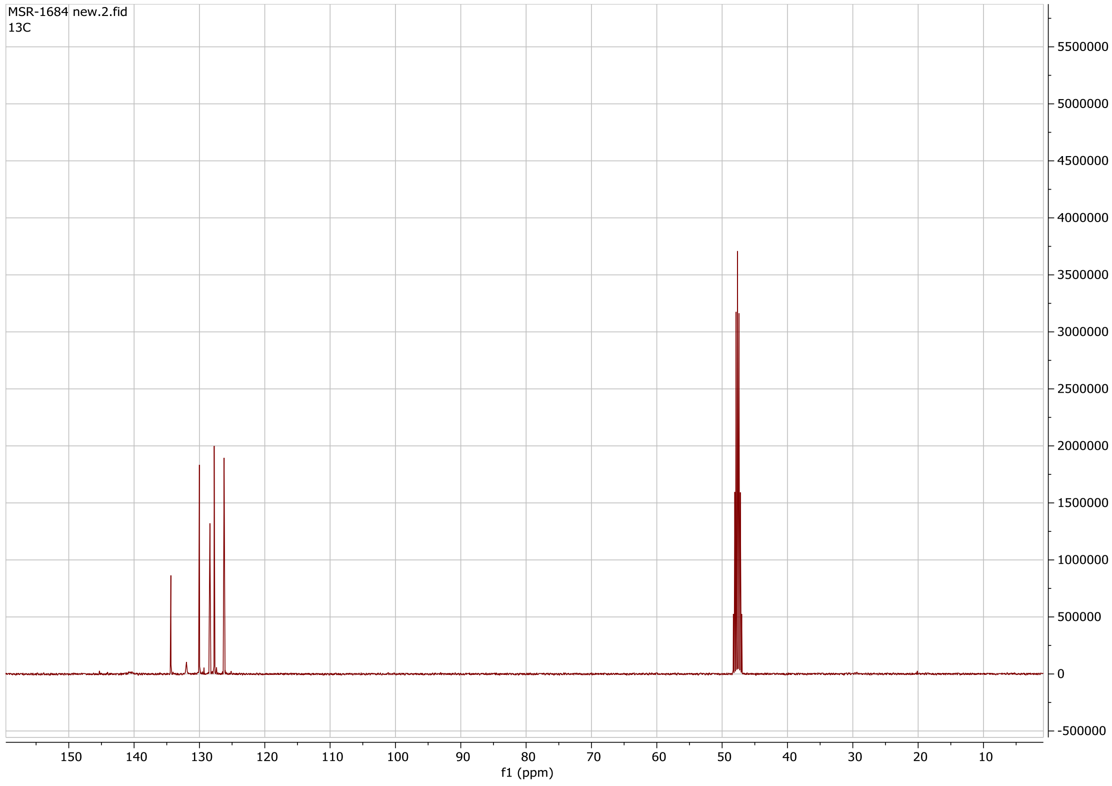

Ethyl 4-(3-chlorophenyl)-1*H*-1,2,3-triazole-5-carboxylate (65):^{1}H NMR (400 MHz, CD_3OD)

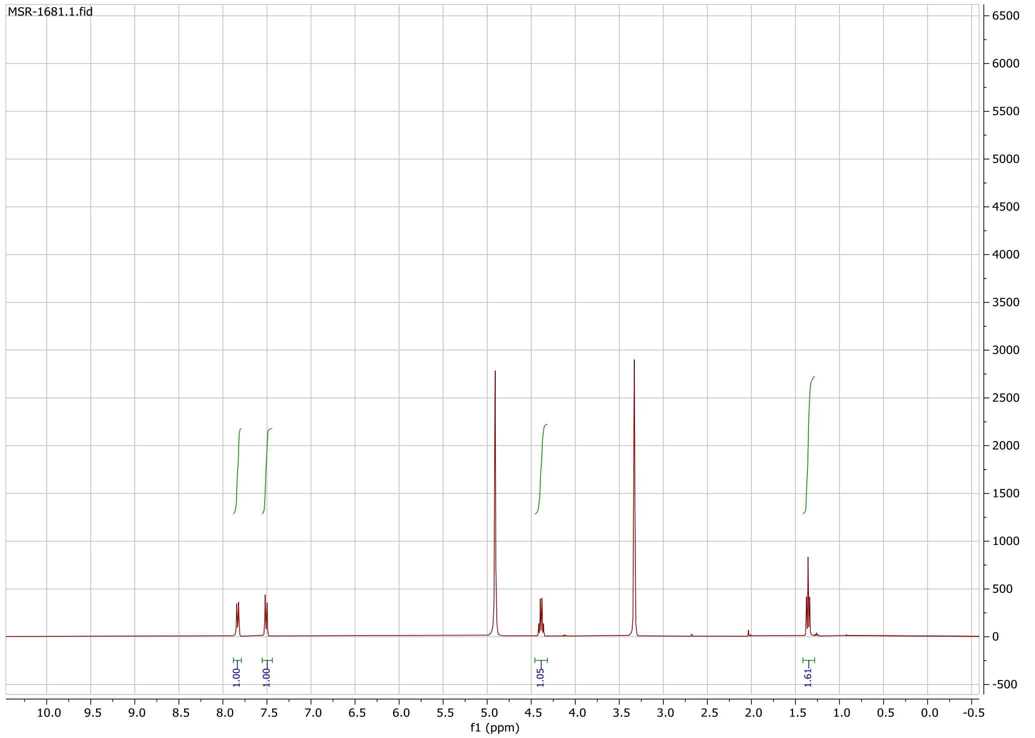

Ethyl 4-(3-chlorophenyl)-1*H*-1,2,3-triazole-5-carboxylate (65):^{13}C NMR (101 MHz, CD_3OD)

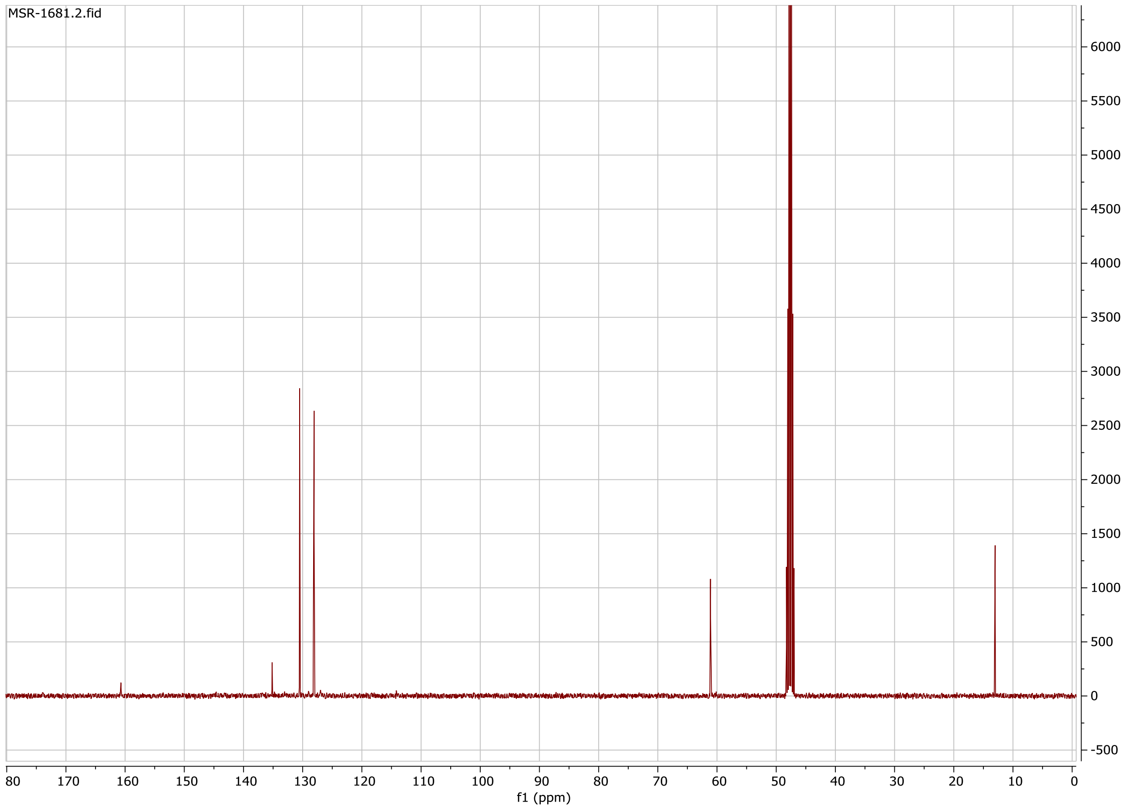

4-(4-Chlorophenyl)-1*H*-1,2,3-triazole-5-carboxamide (66): ^{1}H NMR (400 MHz, CD_3OD)

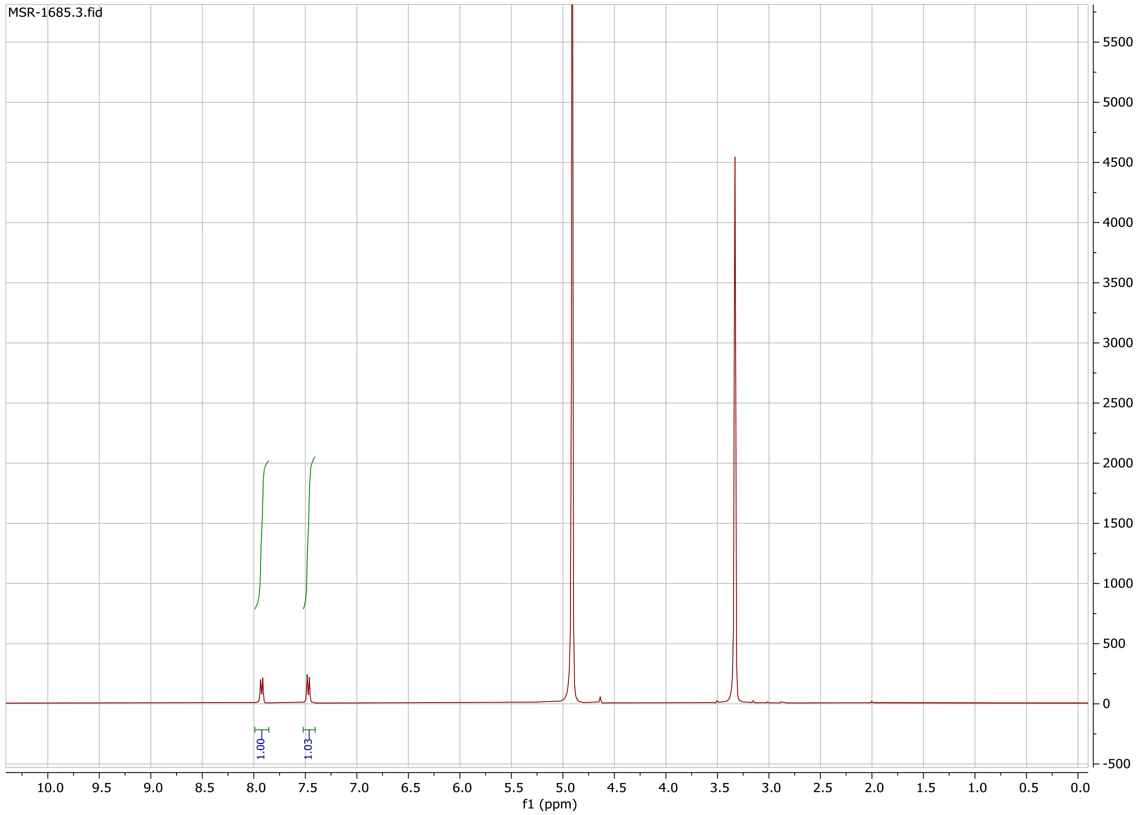

4-(4-Chlorophenyl)-1*H*-1,2,3-triazole-5-carboxamide (66): ^{13}C NMR (101 MHz, CD_3OD)

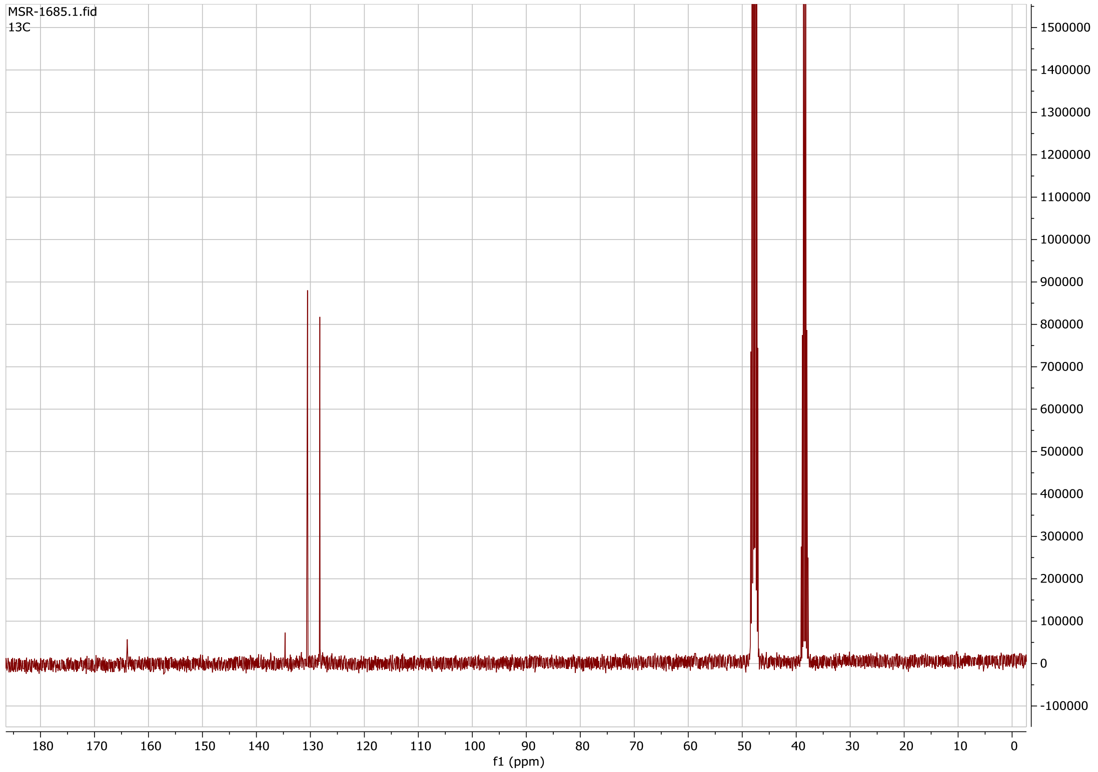

4-(4-Chloro-2-hydroxyphenyl)-1*H*-1,2,3-triazole-5-carboxamide (67):^{1}H NMR (400 MHz, CD_3OD)

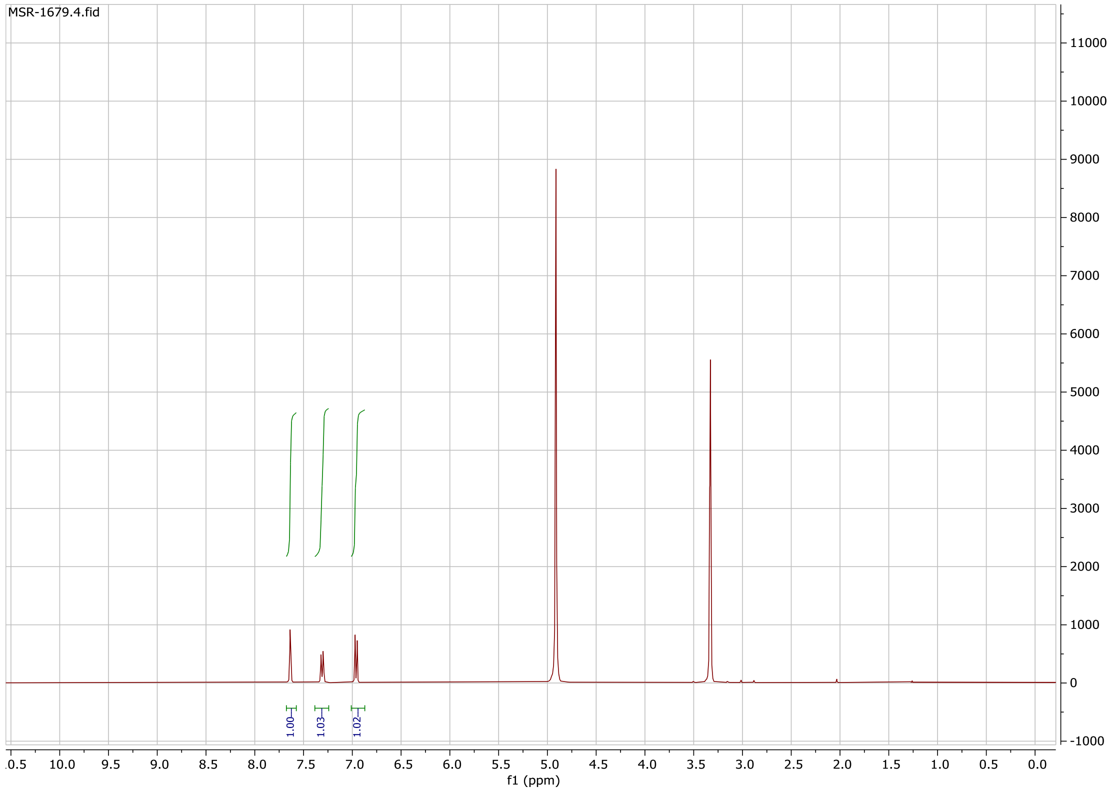

4-(4-Chloro-2-hydroxyphenyl)-1*H*-1,2,3-triazole-5-carboxamide (67):^{13}C NMR (101 MHz, CD_3OD)

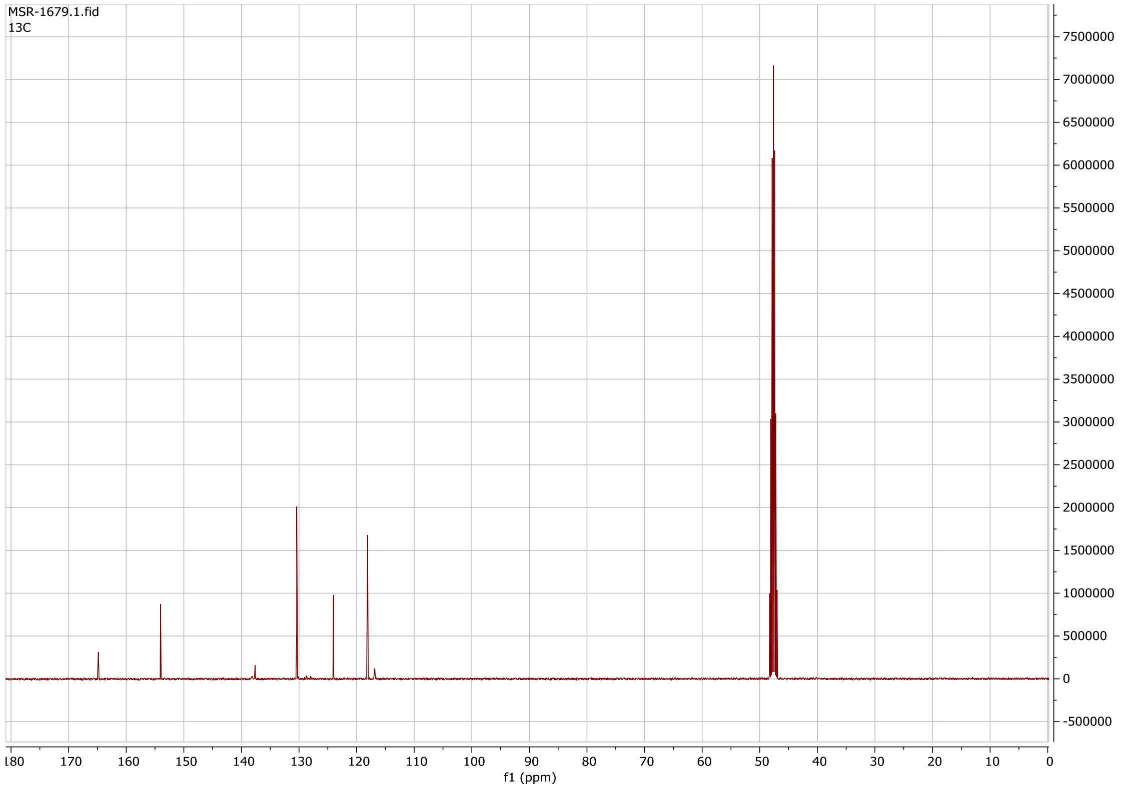

4-Chloro-2-(5-methyl-1,2,3-triazol-4-yl)phenol (68): ^{1}H NMR (400 MHz, $CDCl_3$)

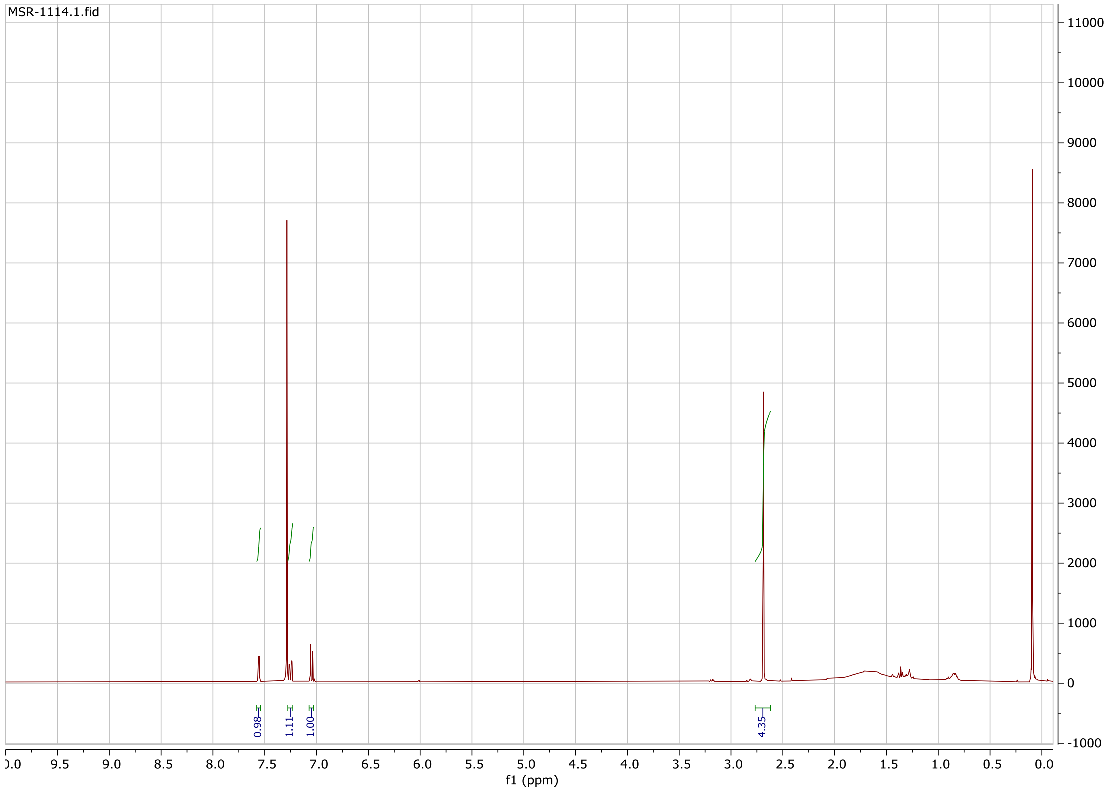

4-Chloro-2-(5-methyl-1,2,3-triazol-4-yl)phenol (68): ^{13}C NMR (101 MHz, CD_3OD)

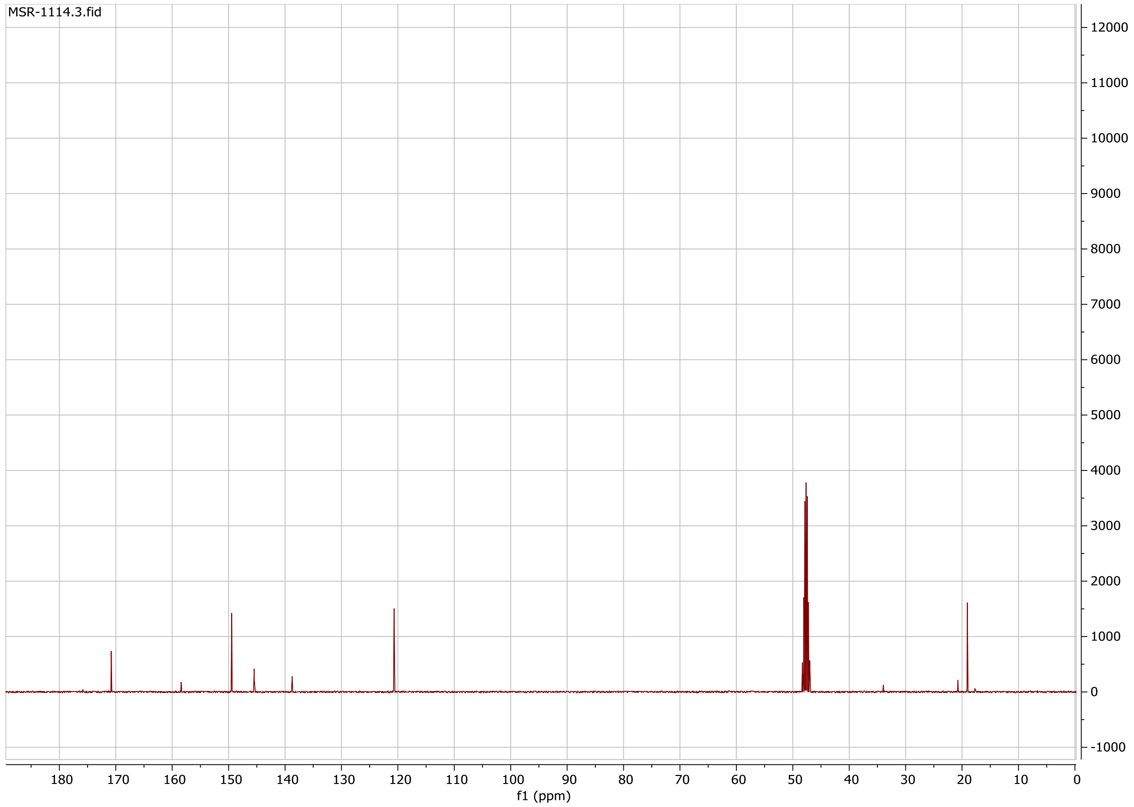

***N*-(4-Bromophenyl)-1,2,3-triazol-4-amine (78):**^{1}H NMR (400 MHz, CD_3OD)

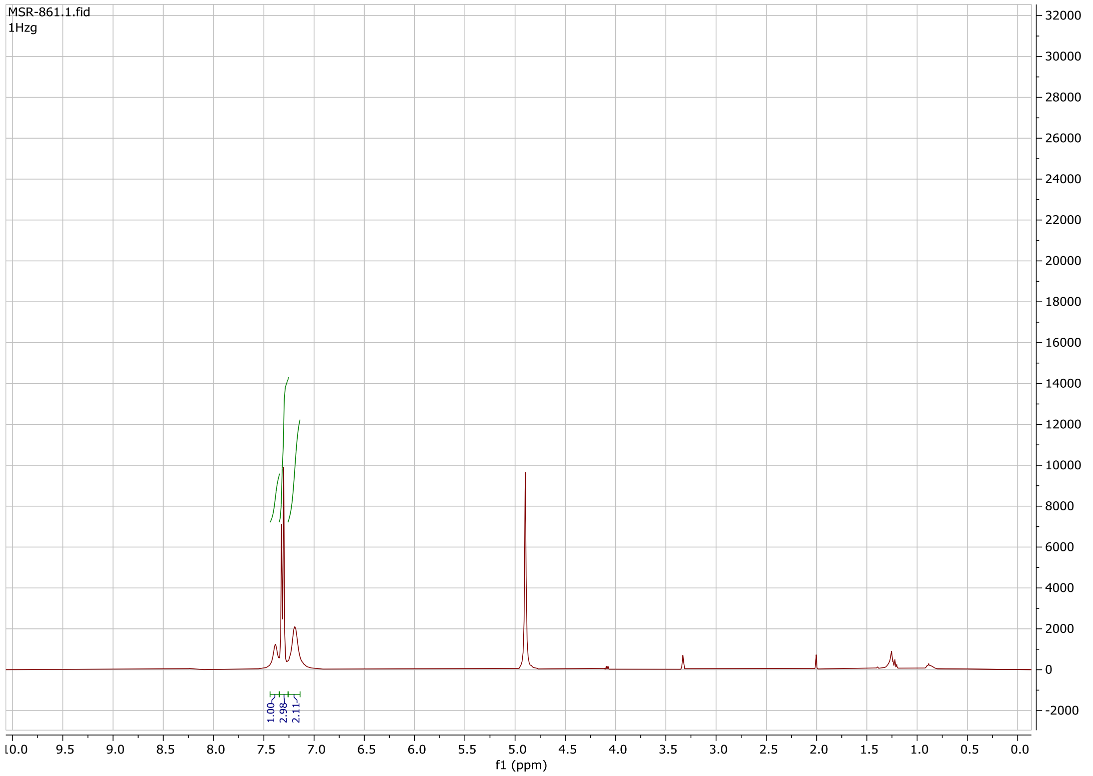

***N*-(4-Bromophenyl)-1,2,3-triazol-4-amine (78):**^{13}C NMR (101 MHz, CD_3OD)

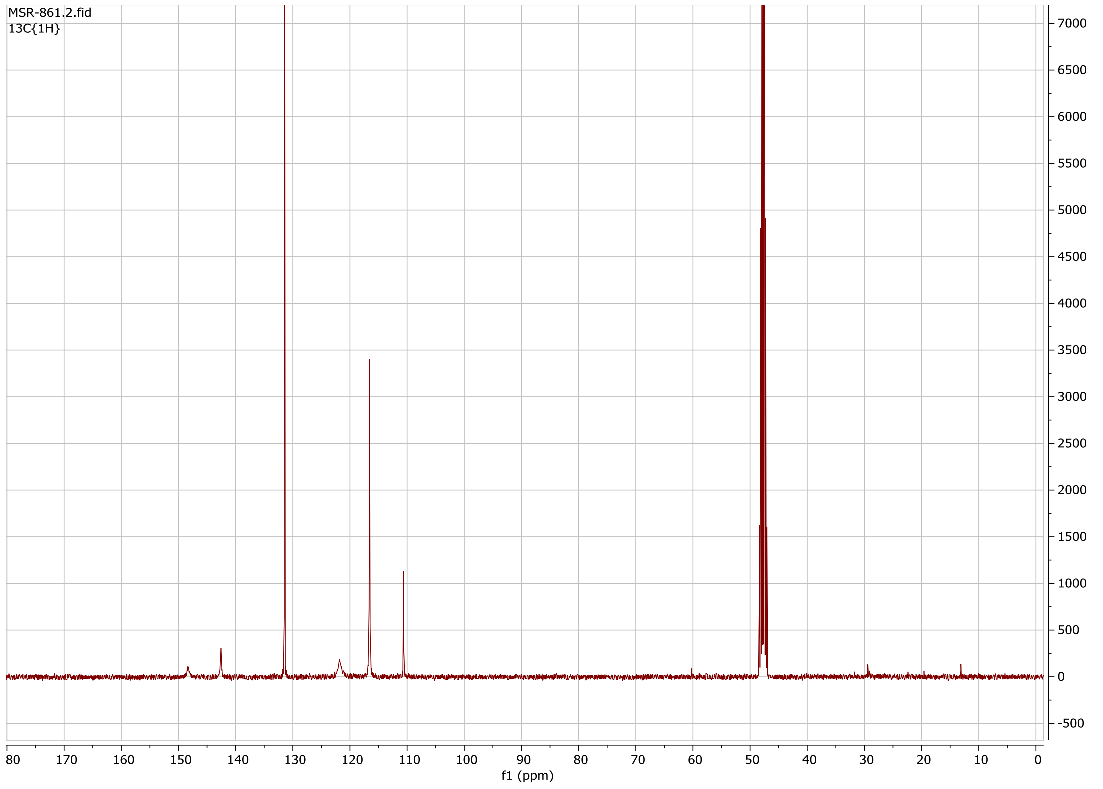

4-(Phenoxy)-1,2,3-triazole (82): ^{1}H NMR (400 MHz, $CDCl_3$)

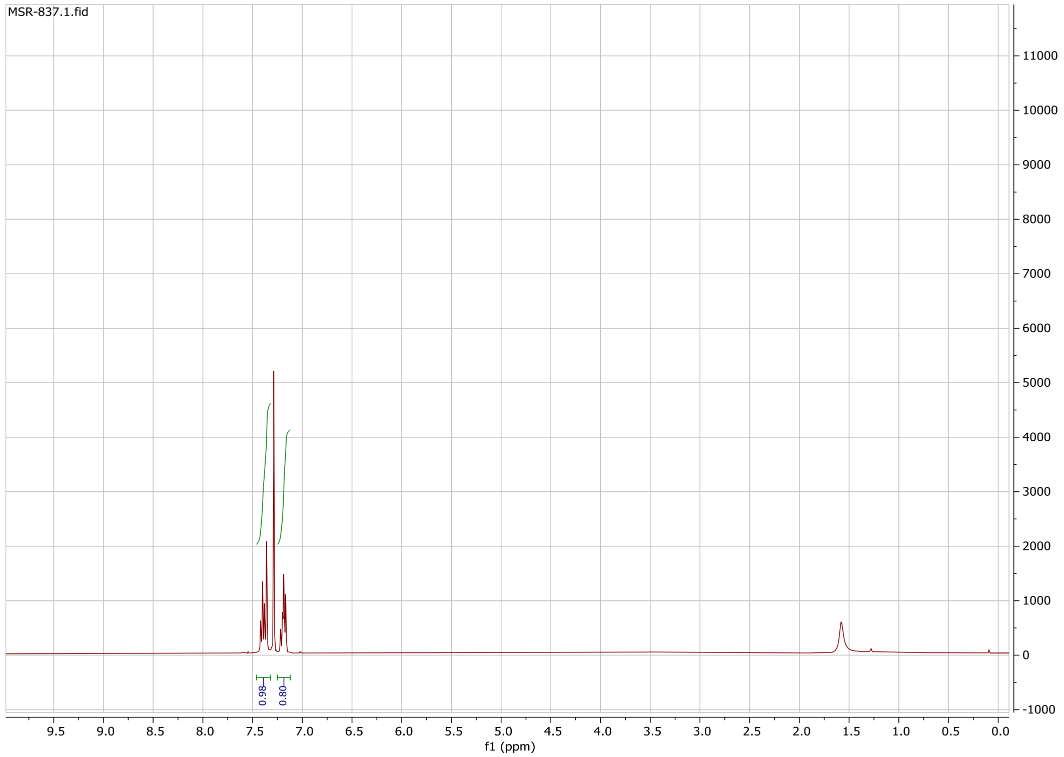

4-(Phenoxy)-1,2,3-triazole (82): ^{13}C NMR (101 MHz, CD_3OD)

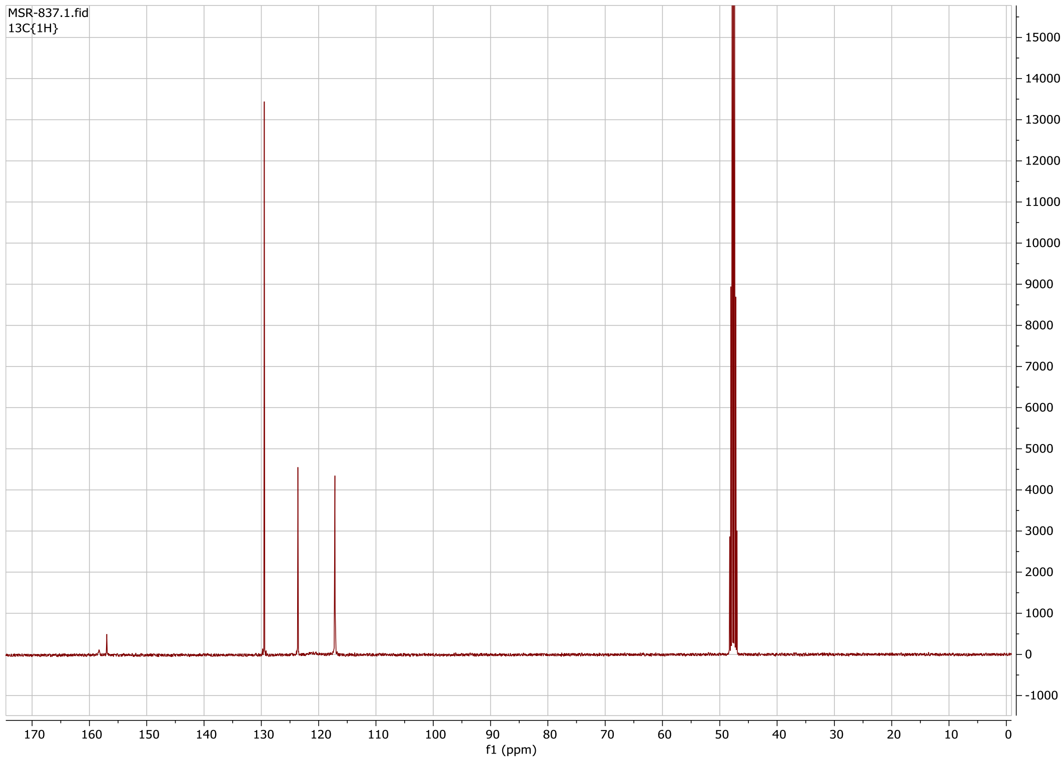

4-(Phenylthio)-1,2,3-triazole (83): ^{1}H NMR (400 MHz, $CDCl_3$)

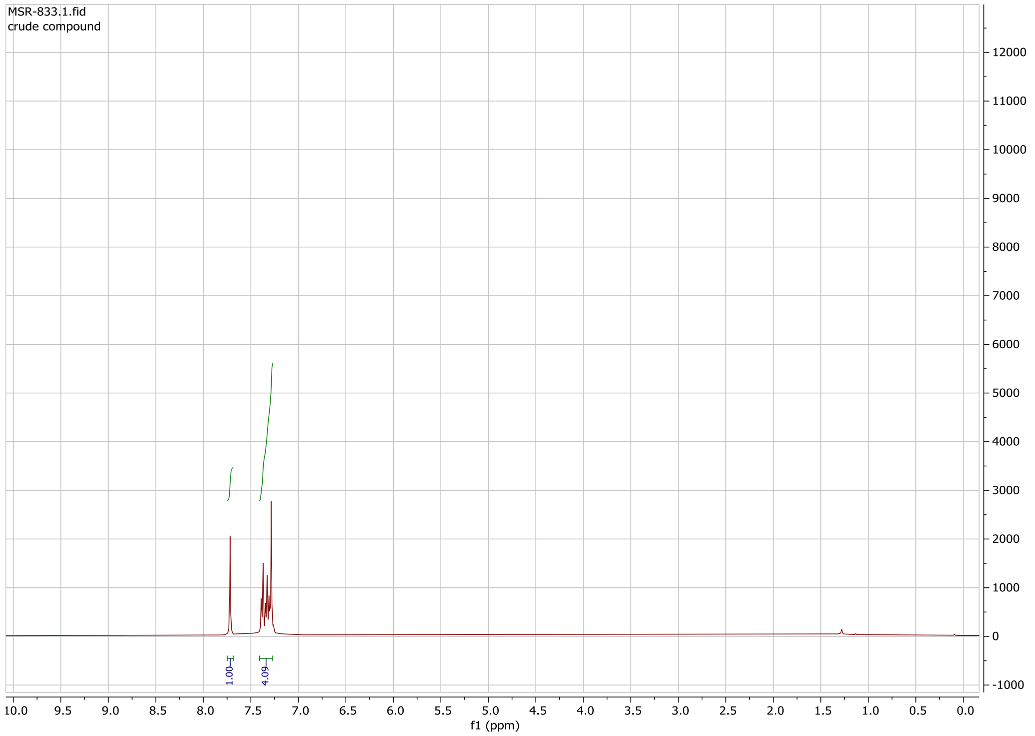

4-(Phenylthio)-1,2,3-triazole (83): ^{13}C NMR (101 MHz, CD_3OD)

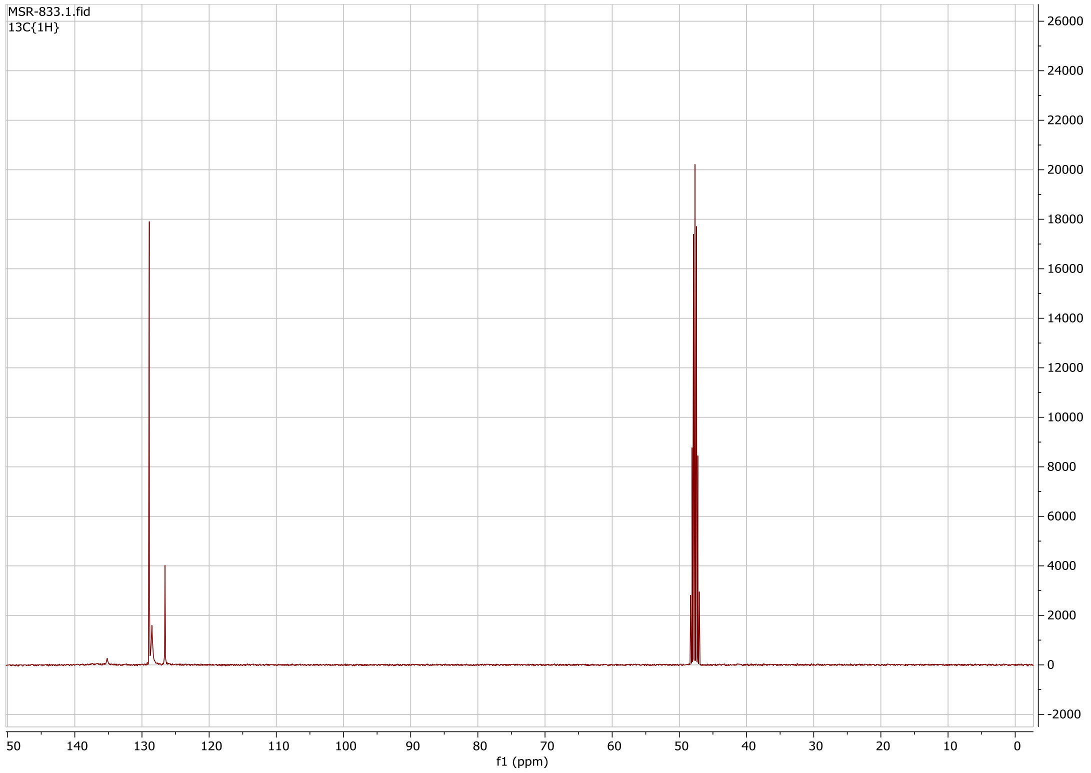

4-(Phenylsulfonyl)-1*H*-1,2,3-triazole (84): ^{1}H NMR (400 MHz, $CDCl_3$)

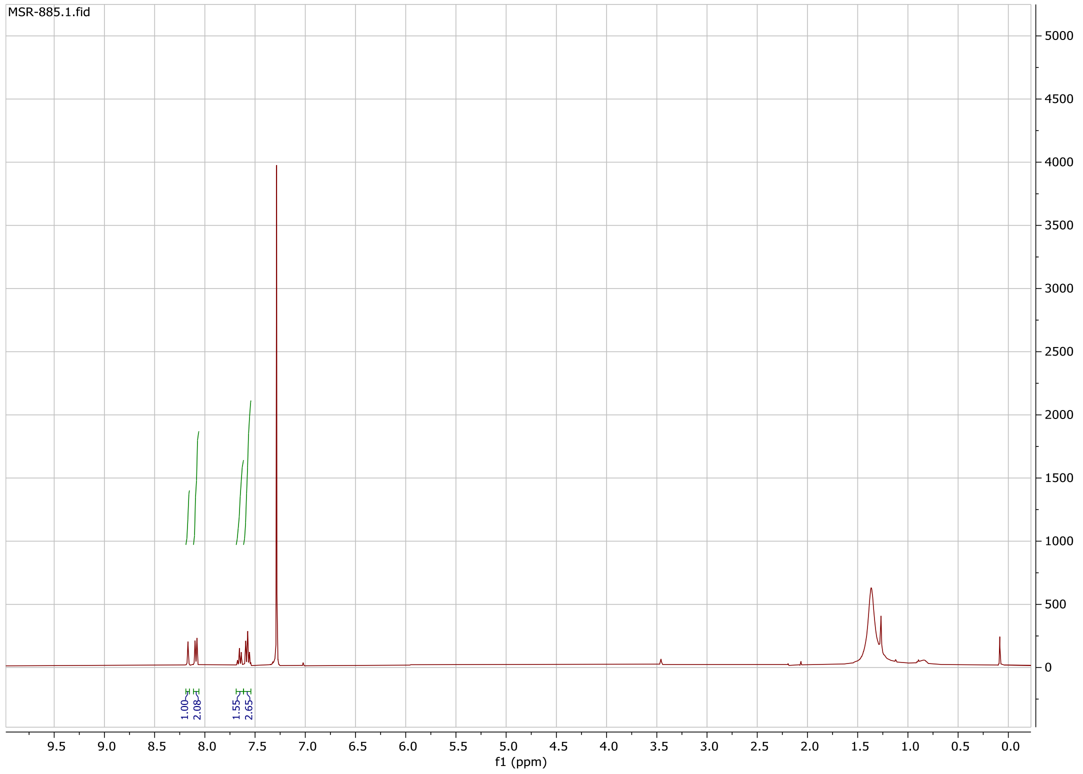

4-(Phenylsulfonyl)-1*H*-1,2,3-triazole (84): ^{13}C NMR (101 MHz, CD_3OD)

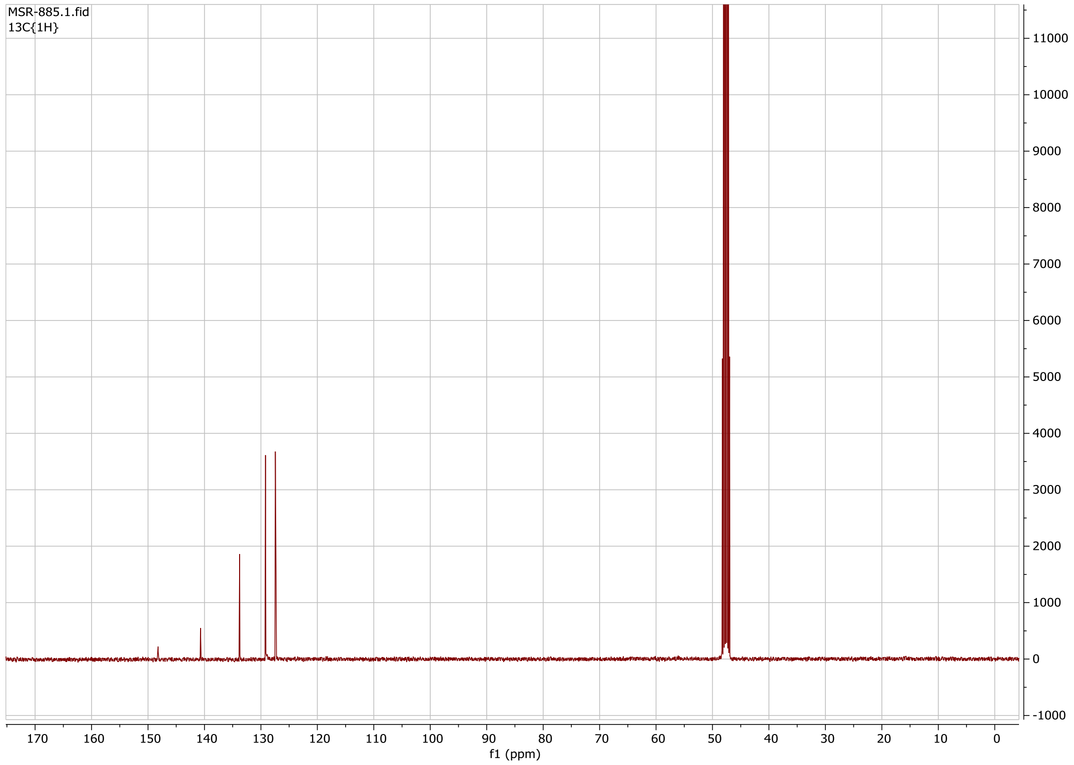

Phenyl(1,2,3-triazol-4-yl)methanol (85): ^{1}H NMR (400 MHz, $CDCl_3$)

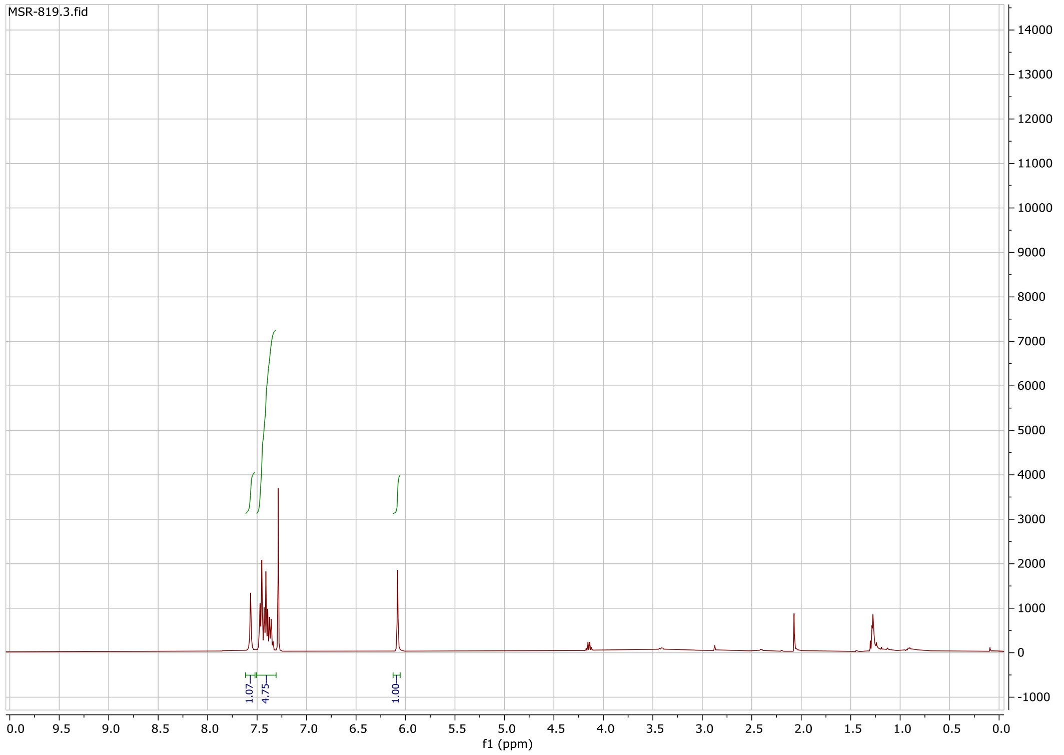

Phenyl(1,2,3-triazol-4-yl)methanol (85): ^{13}C NMR (101 MHz, CD_3OD)

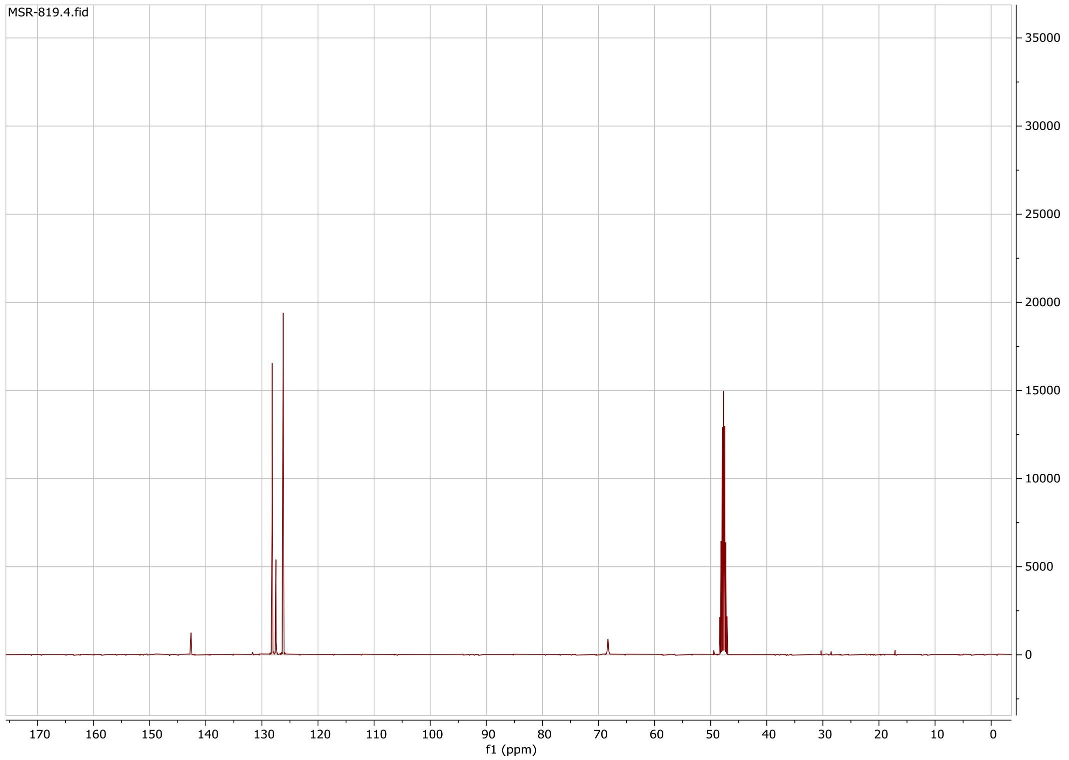

***N*-Phenyl-1*H*-1,2,3-triazole-4-carboxamide (86):**^{1}H NMR (400 MHz, $CDCl_3$)

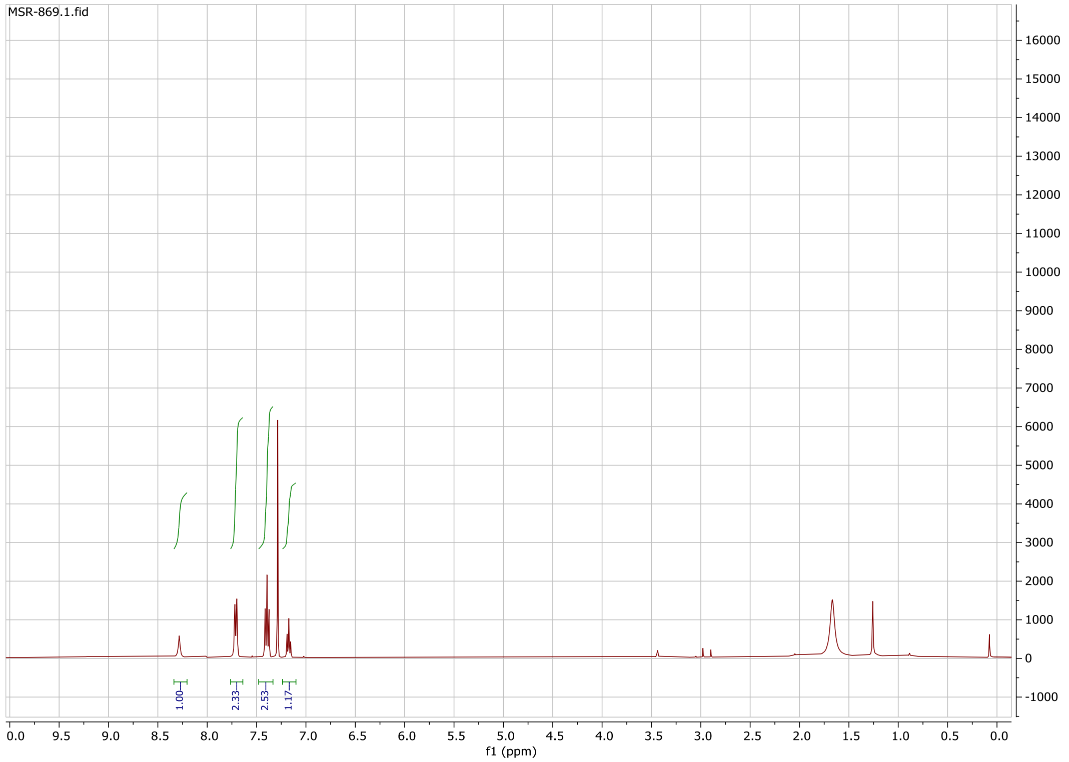

***N*-Phenyl-1*H*-1,2,3-triazole-4-carboxamide (86):**^{13}C NMR (101 MHz, CD_3OD)

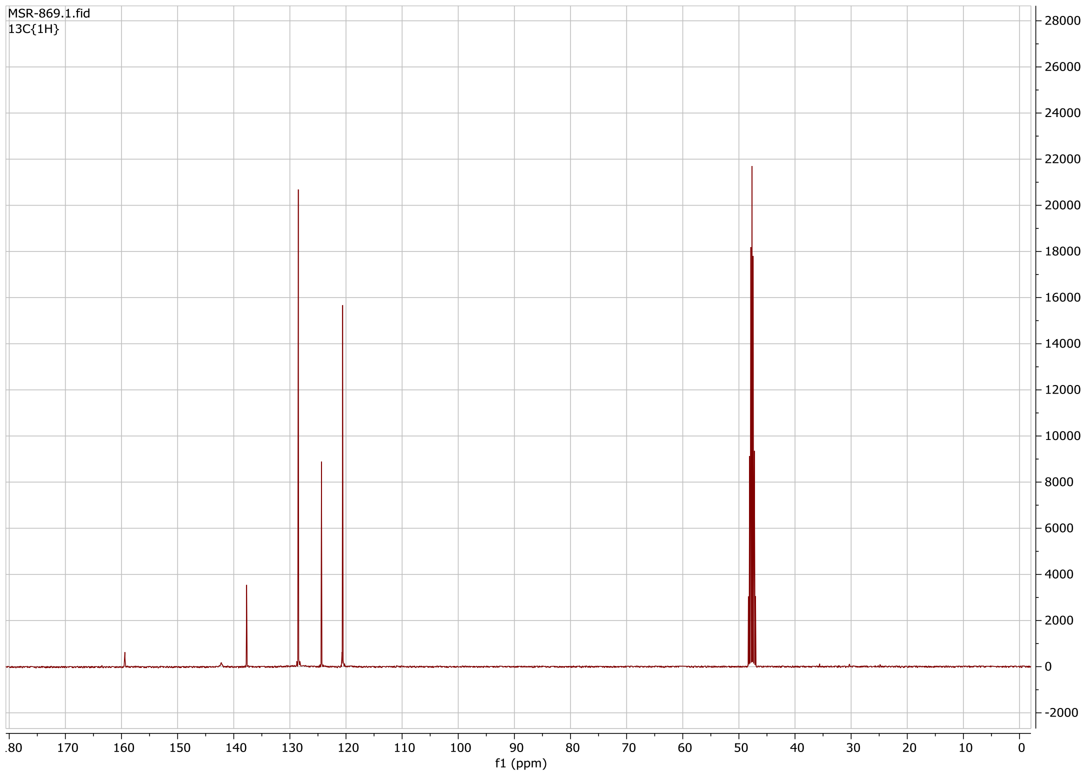

Phenyl(1*H*-1,2,3-triazol-4-yl)methanone O-methyl oxime (87):^{1}H NMR (400 MHz, $CDCl_3$)

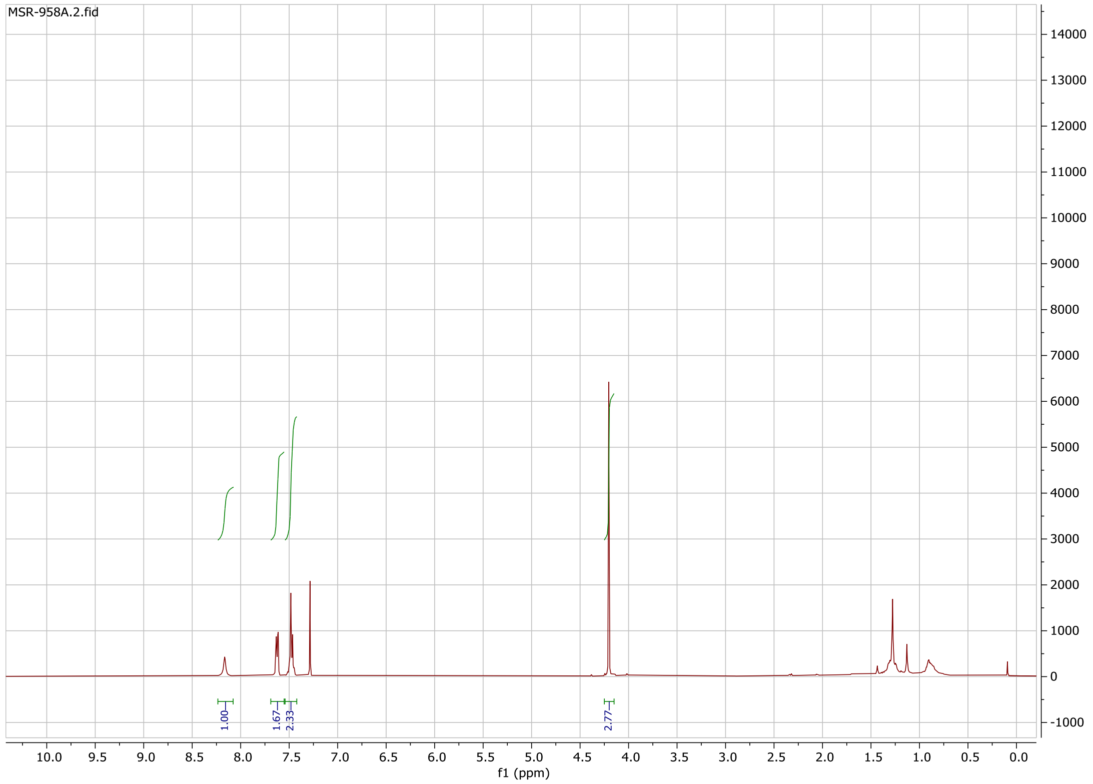

Phenyl(1*H*-1,2,3-triazol-4-yl)methanone O-methyl oxime (87):^{13}C NMR (101 MHz, CD_3OD)

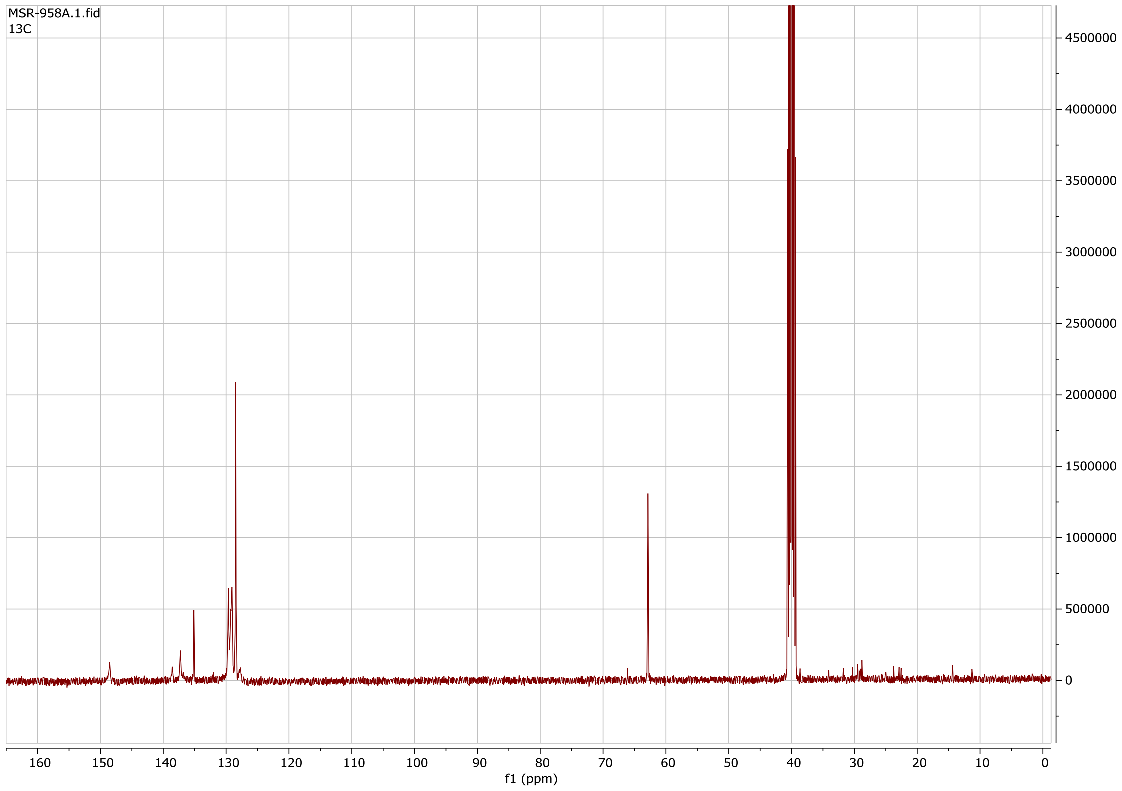

3,8-Dihydroindeno[1,2-*d*][1,2,3]triazole (89): ^{1}H NMR (400 MHz, $CDCl_3$)

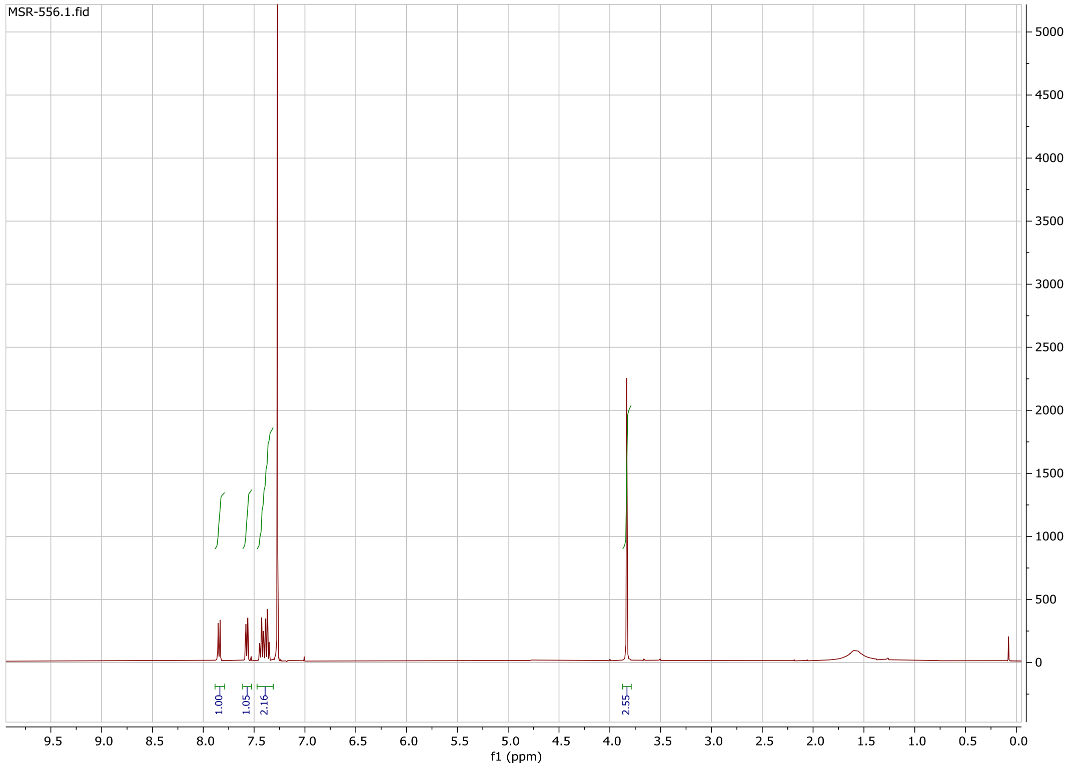

3,8-Dihydroindeno[1,2-*d*][1,2,3]triazole (89): ^{13}C NMR (101 MHz, CD_3OD)

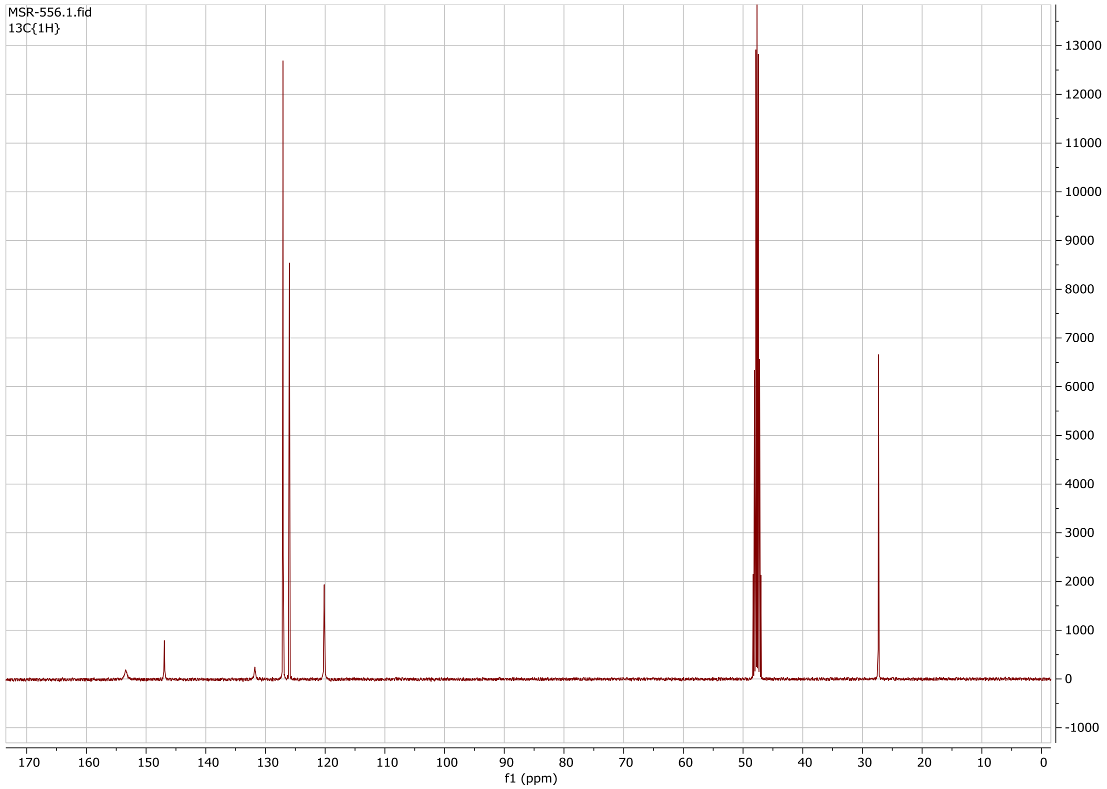

Phenyl(1*H*-1,2,3-triazol-5-yl)methanone (99): ^{1}H NMR (400 MHz, $CDCl_3$)

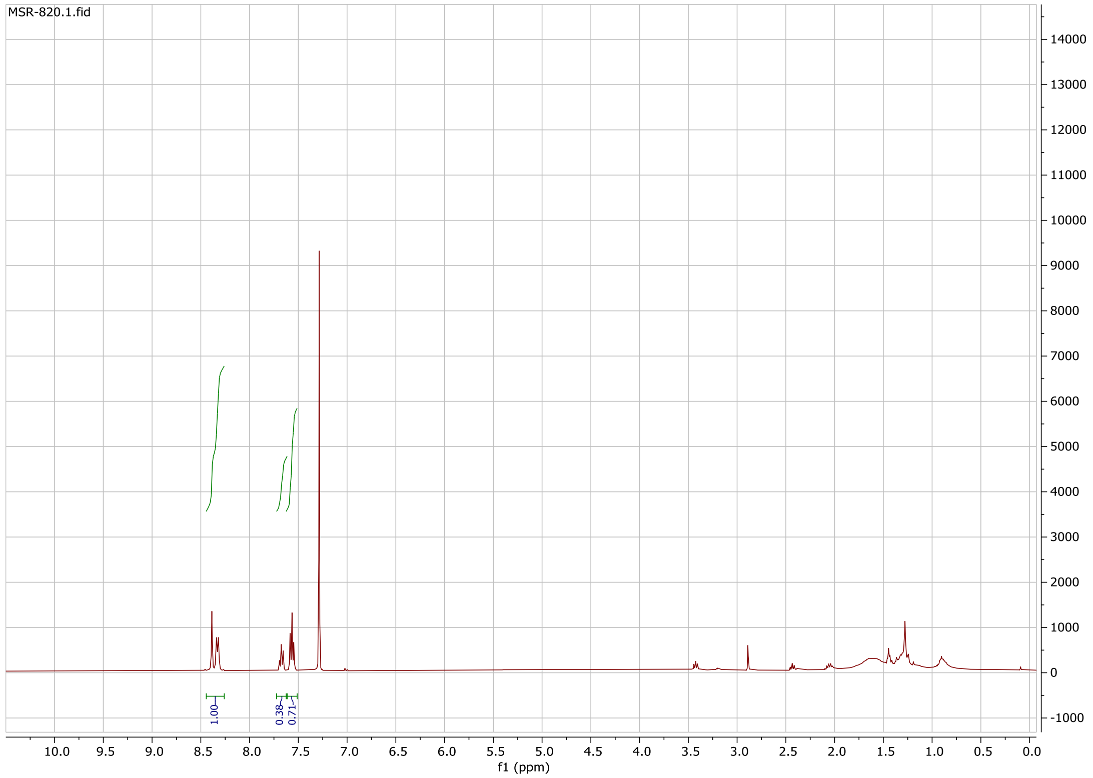

Phenyl(1*H*-1,2,3-triazol-5-yl)methanone (99): ^{13}C NMR (101 MHz, CD_3OD)

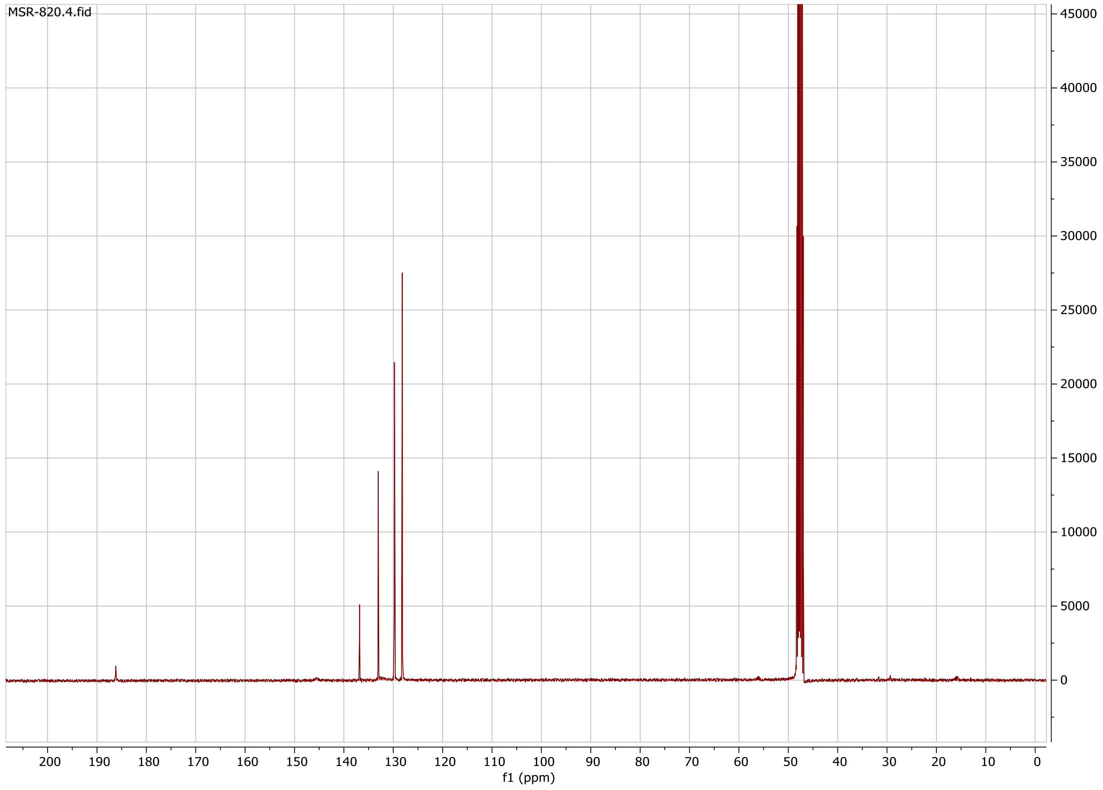

(2-Chlorophenyl)(1*H*-1,2,3-triazol-5-yl)methanone (100): ^{1}H NMR (400 MHz, $CDCl_3$)

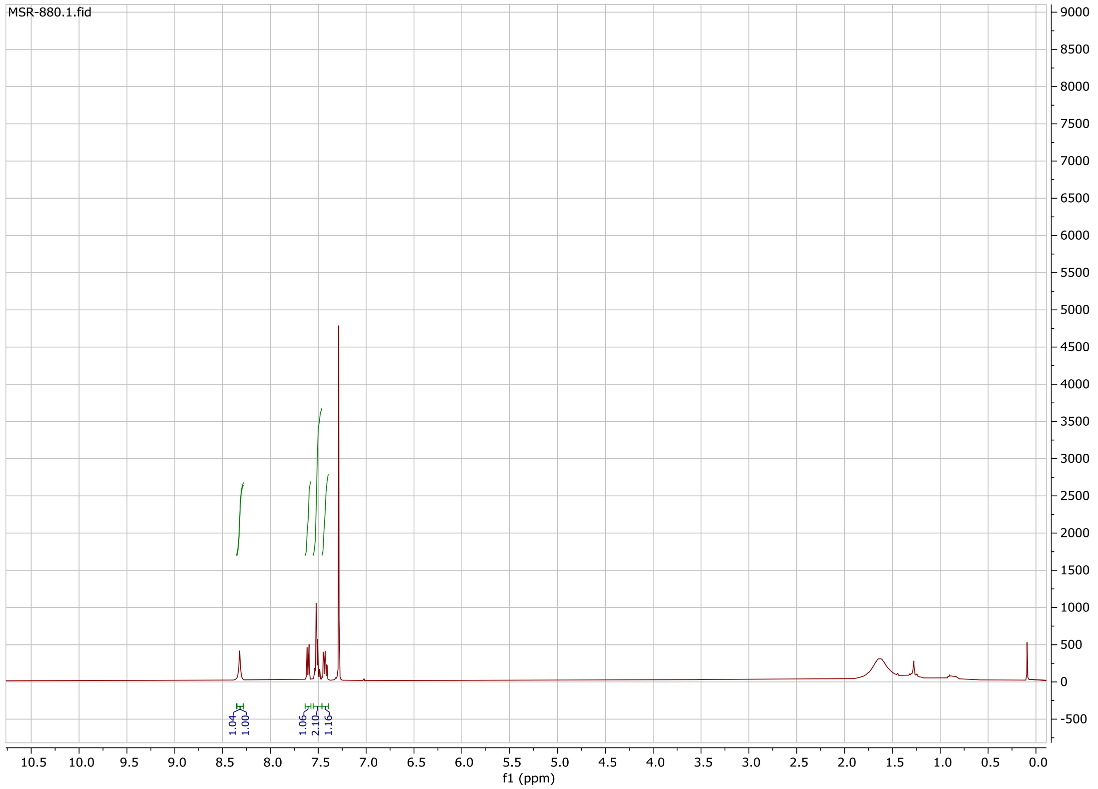

(2-Chlorophenyl)(1*H*-1,2,3-triazol-5-yl)methanone (100): ^{13}C NMR (101 MHz, CD_3OD)

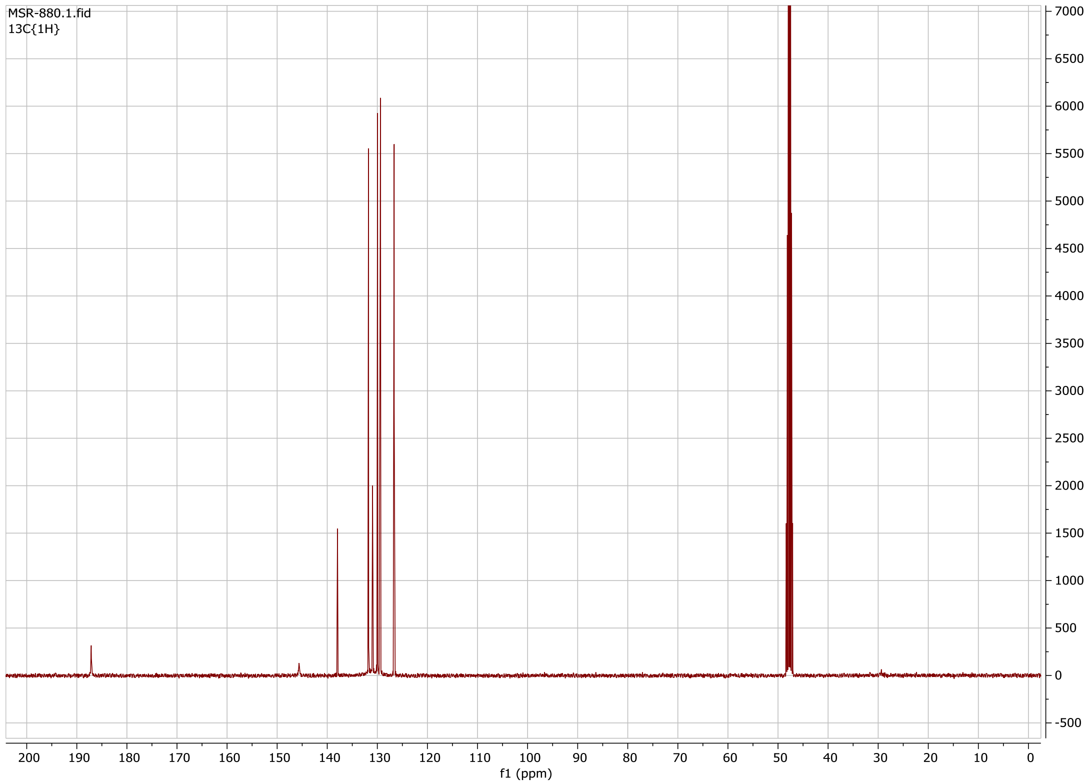

(3-Chlorophenyl)(1*H*-1,2,3-triazol-5-yl)methanone (101): ^{1}H NMR (400 MHz, $CDCl_3$)

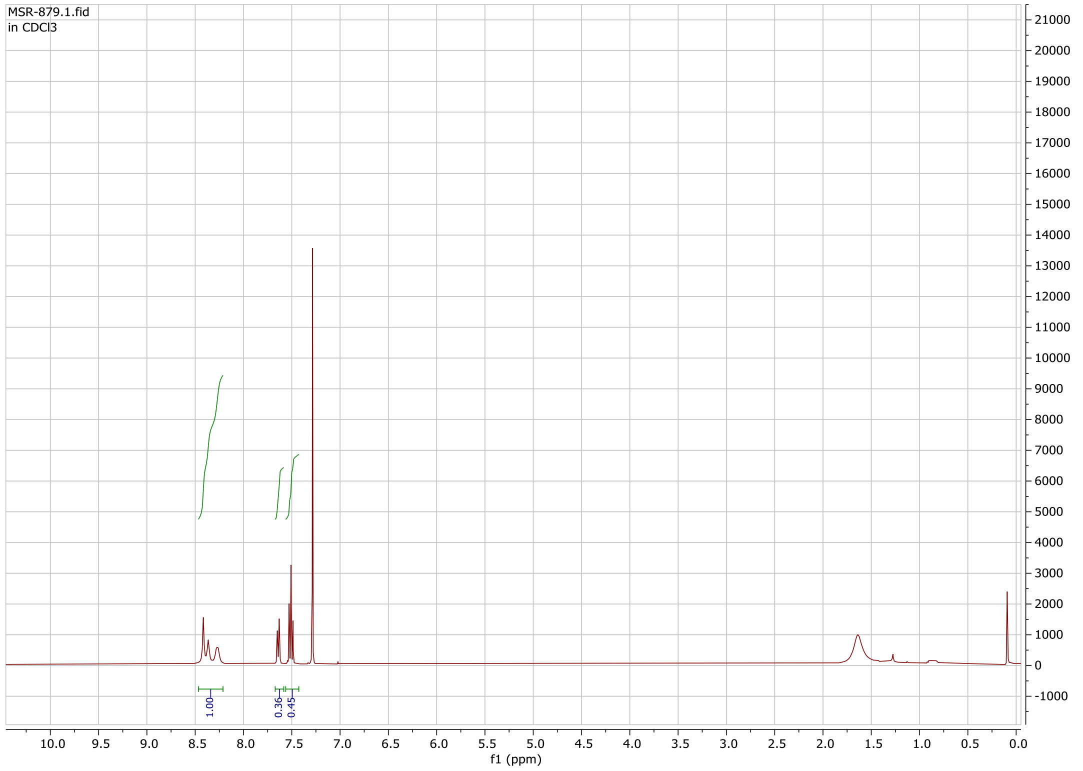

(3-Chlorophenyl)(1*H*-1,2,3-triazol-5-yl)methanone (101): ^{13}C NMR (101 MHz, CD_3OD)

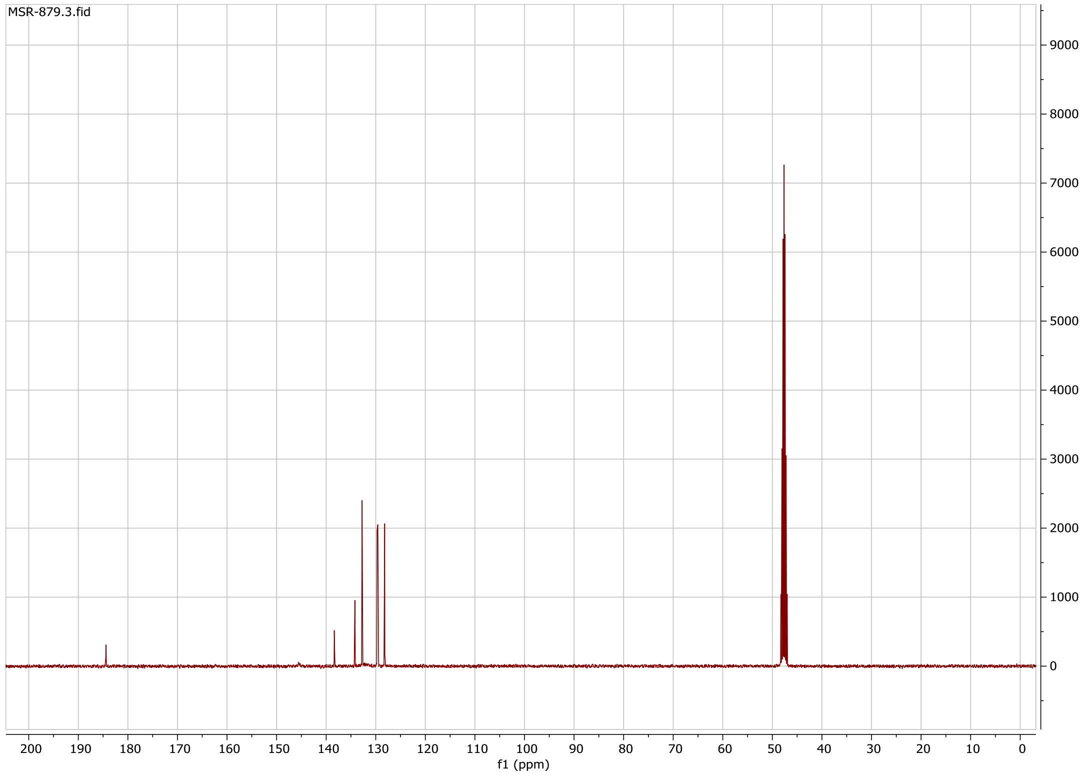

(4-Chlorophenyl)(1*H*-1,2,3-triazol-5-yl)methanone (102): ^{1}H NMR (400 MHz, CD_3OD)

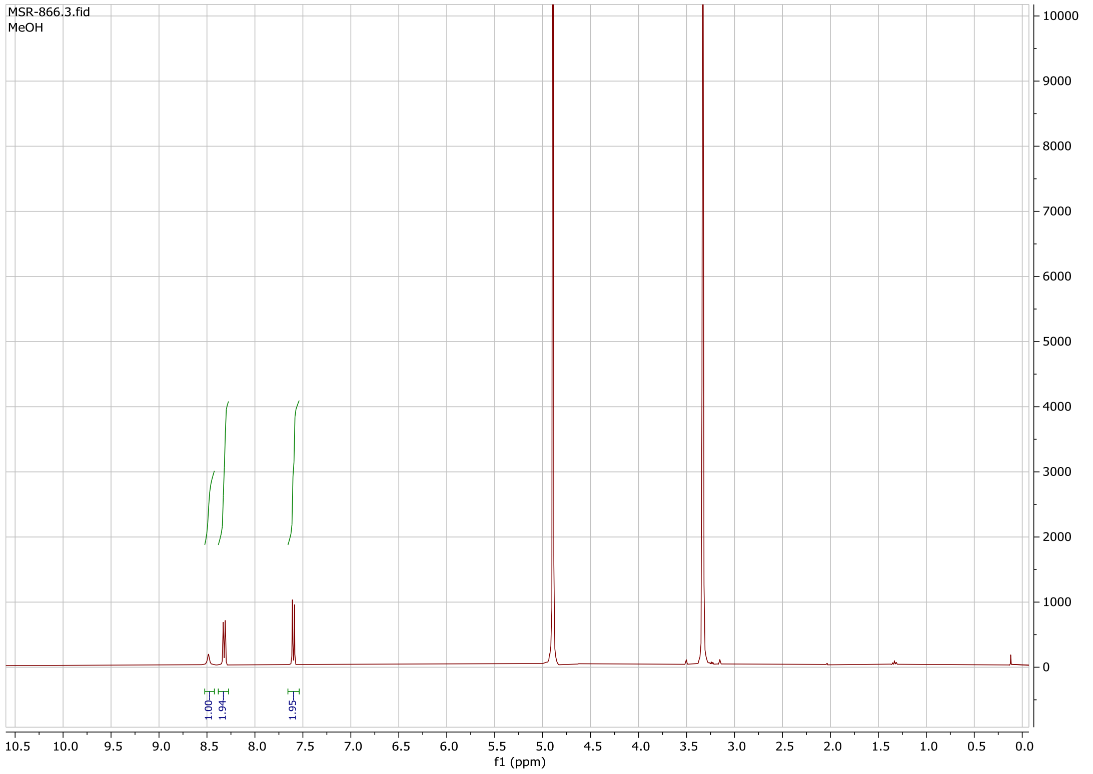

(4-Chlorophenyl)(1*H*-1,2,3-triazol-5-yl)methanone (102): ^{13}C NMR (101 MHz, CD_3OD)

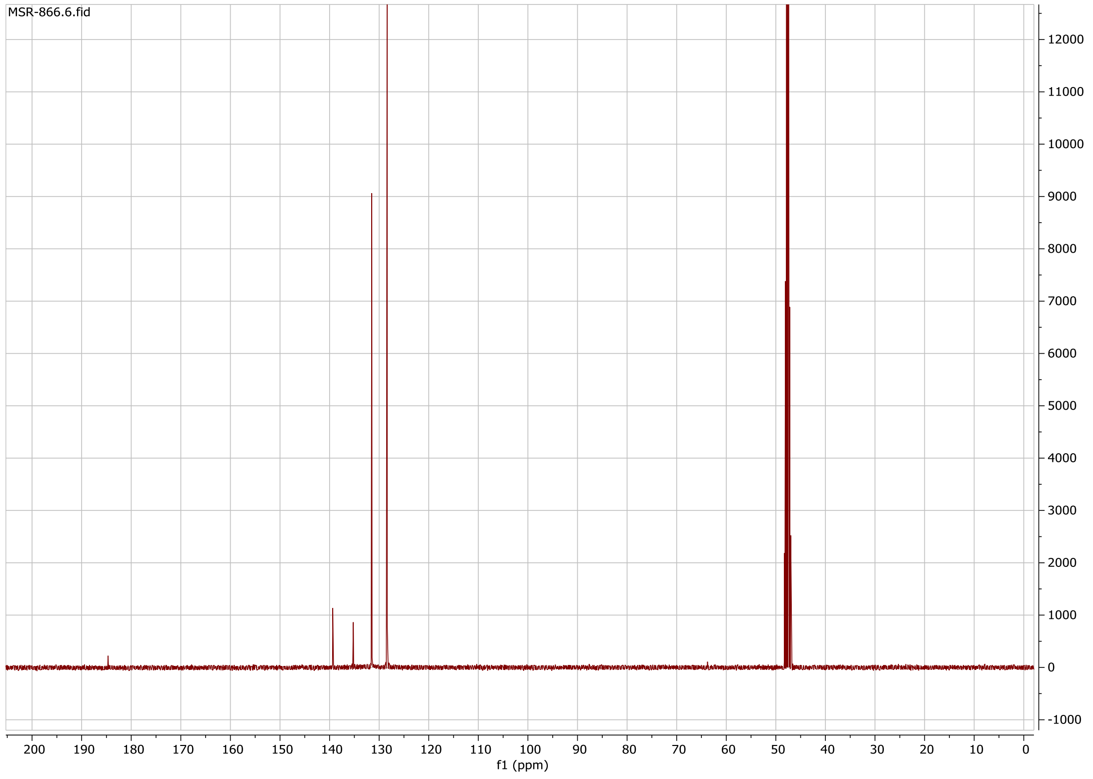

(2-Methoxyphenyl)(1*H*-1,2,3-triazol-5-yl)methanone (103): ^{1}H NMR (400 MHz, $CDCl_3$)

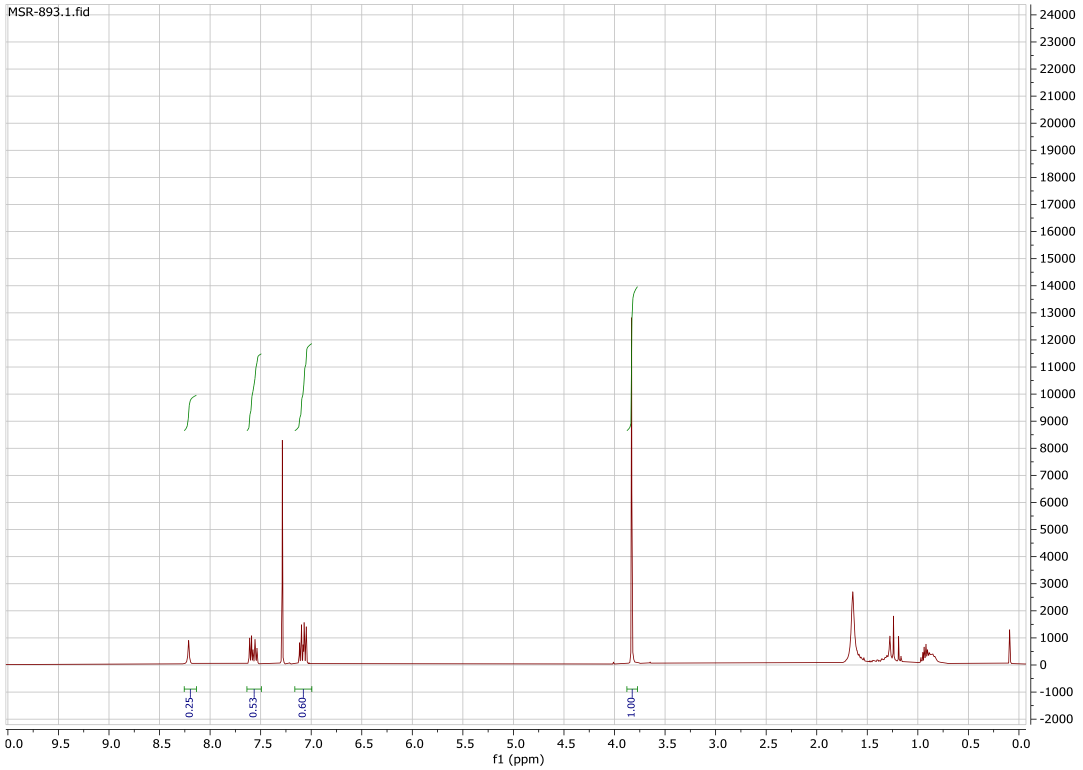

(2-Methoxyphenyl)(1*H*-1,2,3-triazol-5-yl)methanone (103): ^{13}C NMR (101 MHz, CD_3OD)

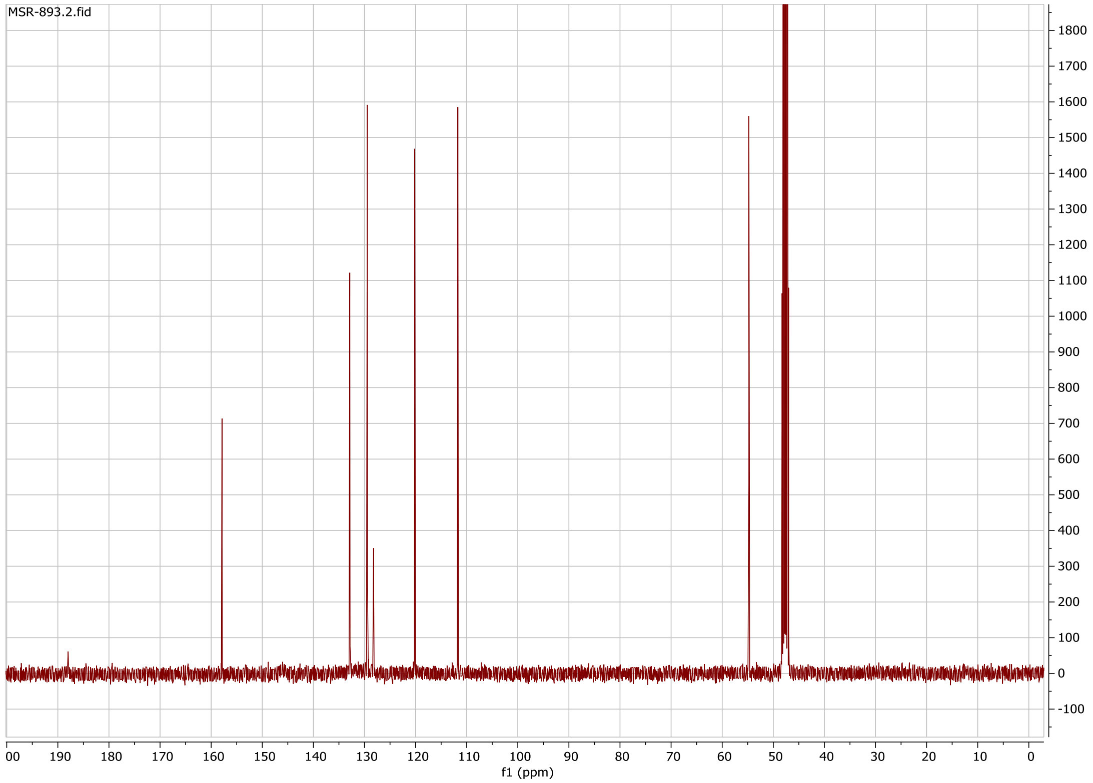

(3-Methoxyphenyl)(1*H*-1,2,3-triazol-5-yl)methanone (104): ^{1}H NMR (400 MHz, CD_3OD)

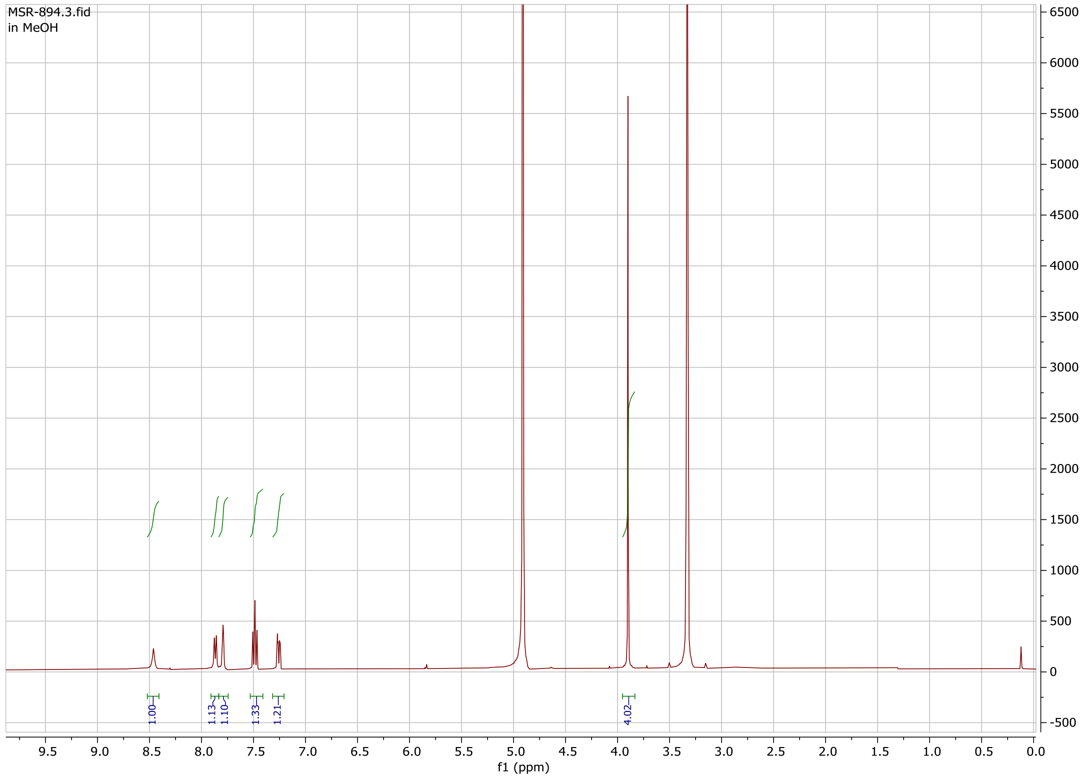

(3-Methoxyphenyl)(1*H*-1,2,3-triazol-5-yl)methanone (104): ^{13}C NMR (101 MHz, CD_3OD)

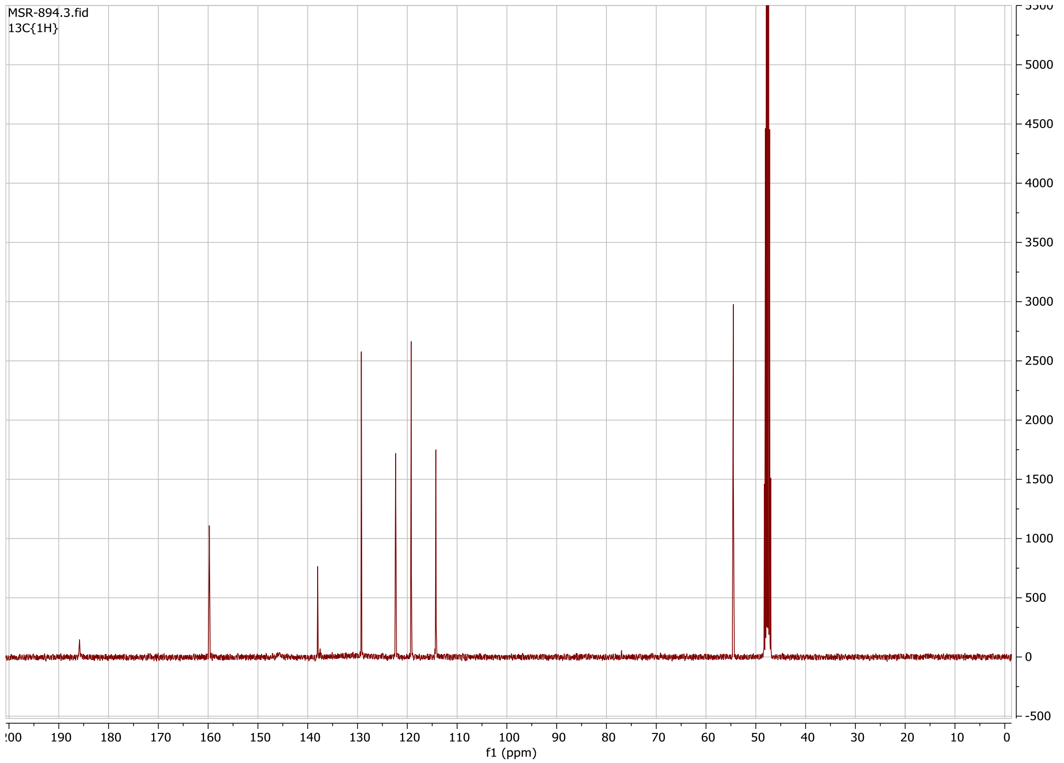

(4-Methoxyphenyl)(1*H*-1,2,3-triazol-5-yl)methanone (105): ^{1}H NMR (400 MHz, CD_3OD)

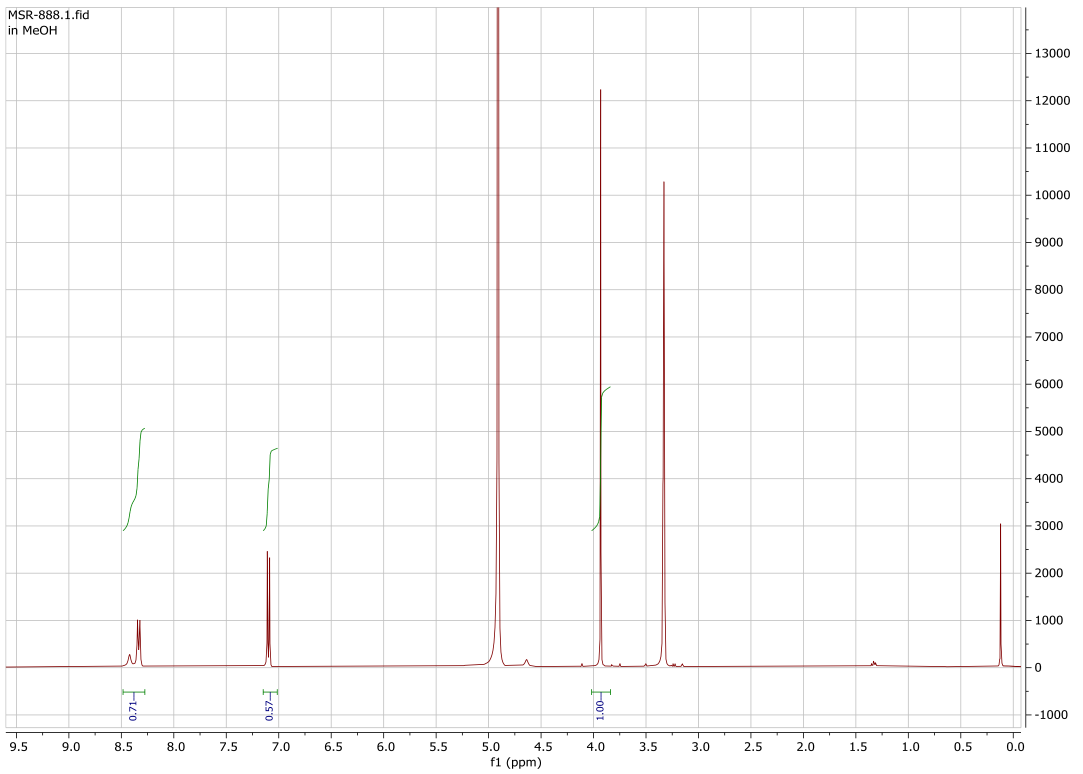

(4-Methoxyphenyl)(1*H*-1,2,3-triazol-5-yl)methanone (105): ^{13}C NMR (101 MHz, CD_3OD)

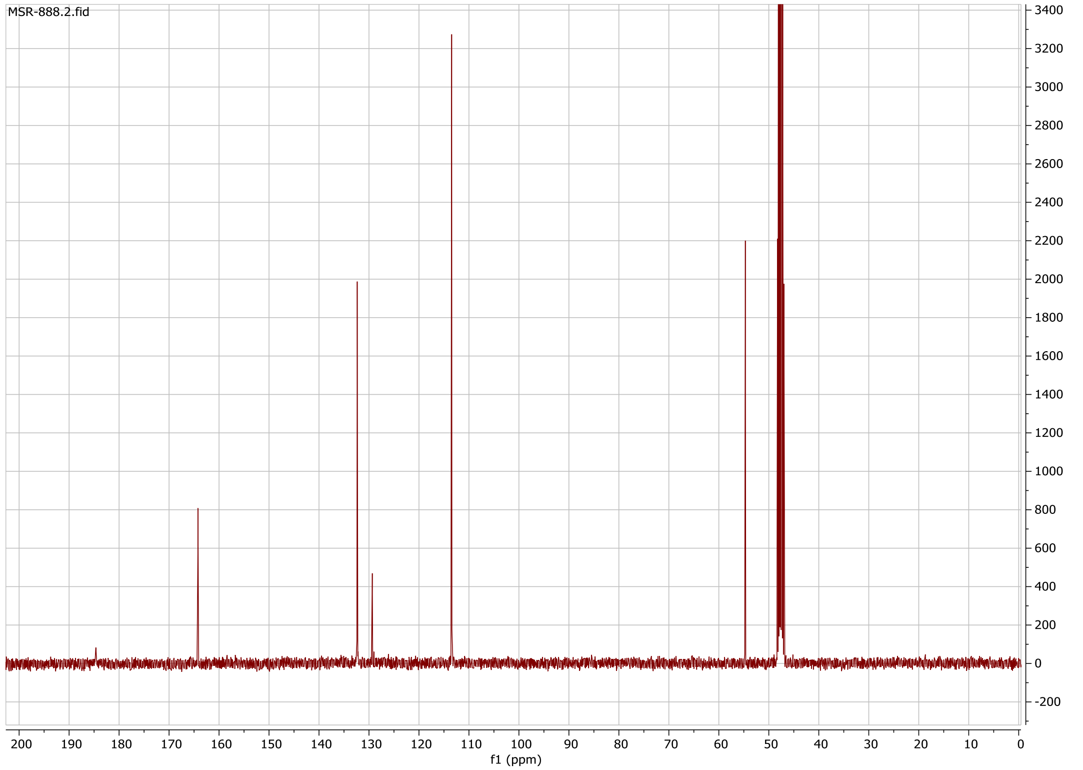

(2-Hydroxyphenyl)(1*H*-1,2,3-triazol-5-yl)methanone (106): ^{1}H NMR (400 MHz, CD_3OD)

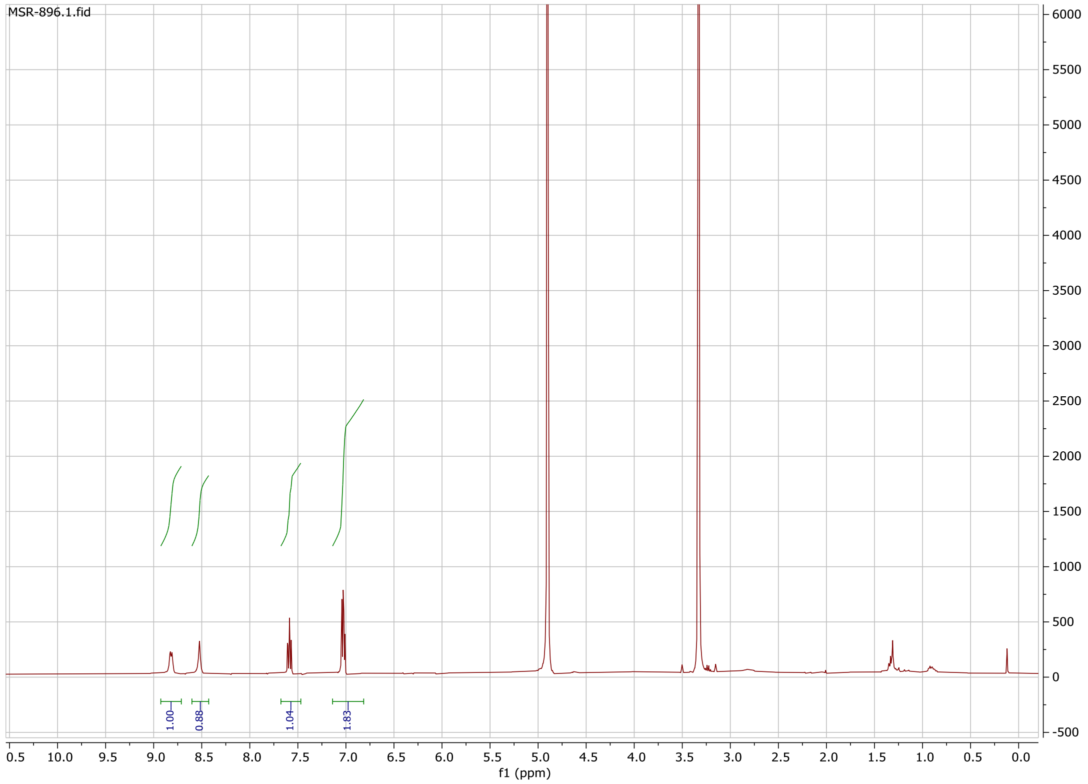

(2-Hydroxyphenyl)(1*H*-1,2,3-triazol-5-yl)methanone (106): ^{13}C NMR (101 MHz, CD_3OD)

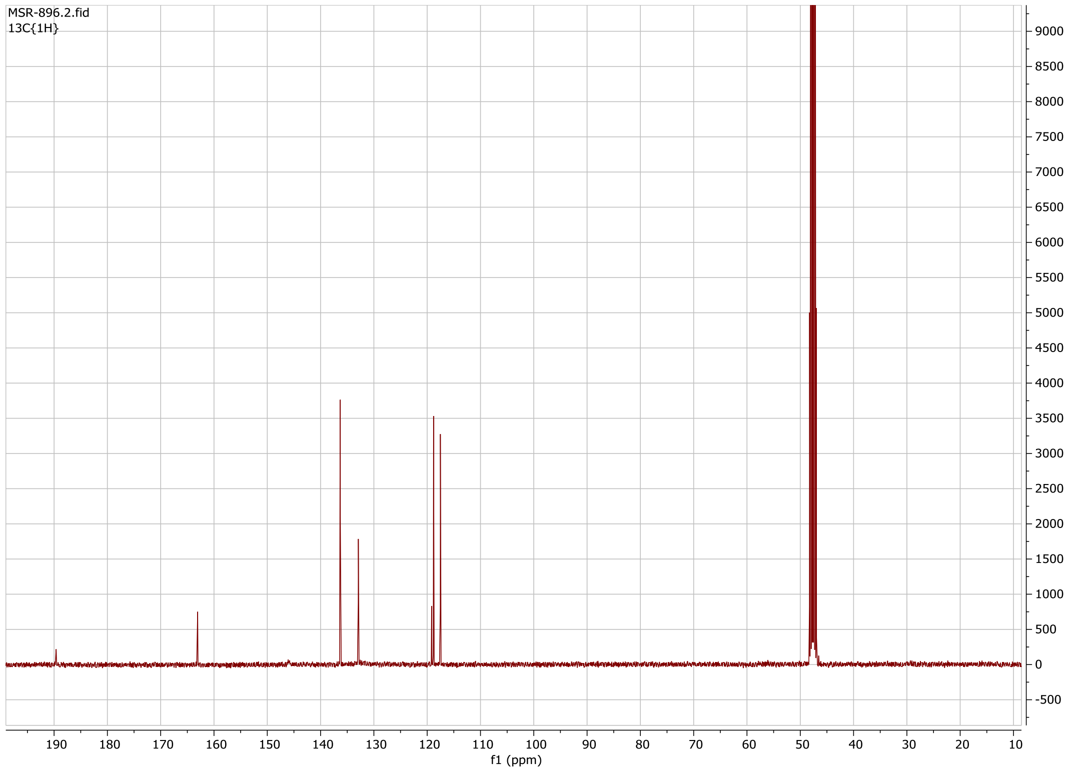

(3-Hydroxyphenyl)(1*H*-1,2,3-triazol-5-yl)methanone (107):^{1}H NMR (400 MHz, CD_3OD)

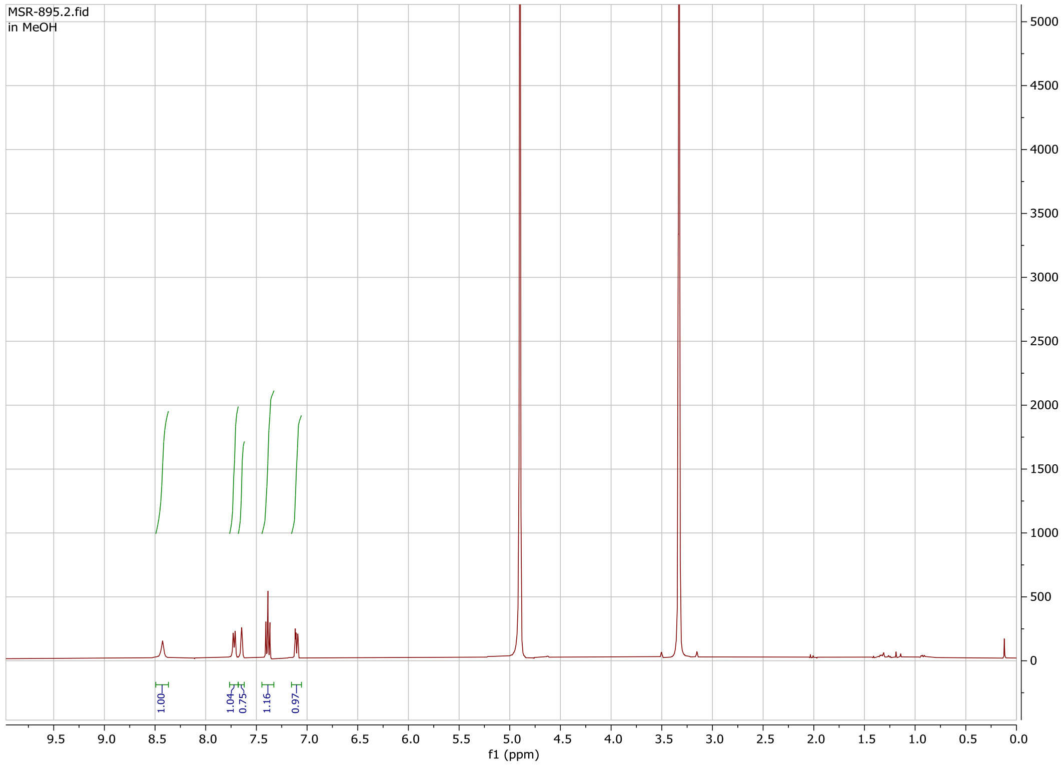

(3-Hydroxyphenyl)(1*H*-1,2,3-triazol-5-yl)methanone (107):^{13}C NMR (101 MHz, CD_3OD)

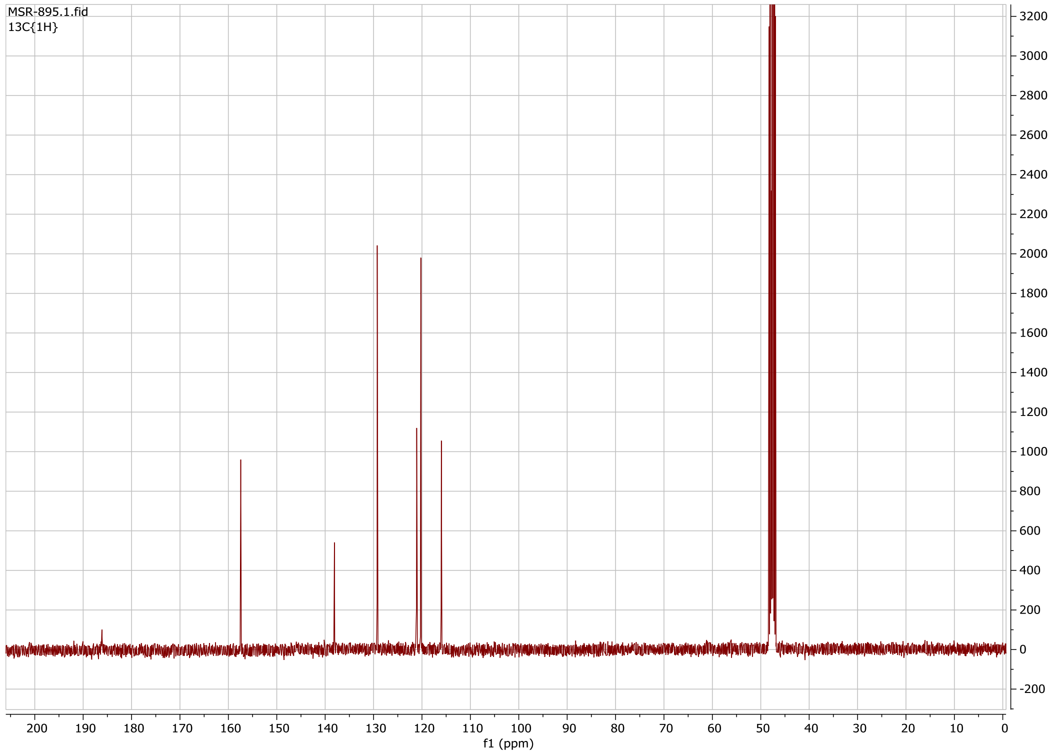

(4-Hydroxyphenyl)(1*H*-1,2,3-triazol-5-yl)methanone (108):^{1}H NMR (400 MHz, CD_3OD)

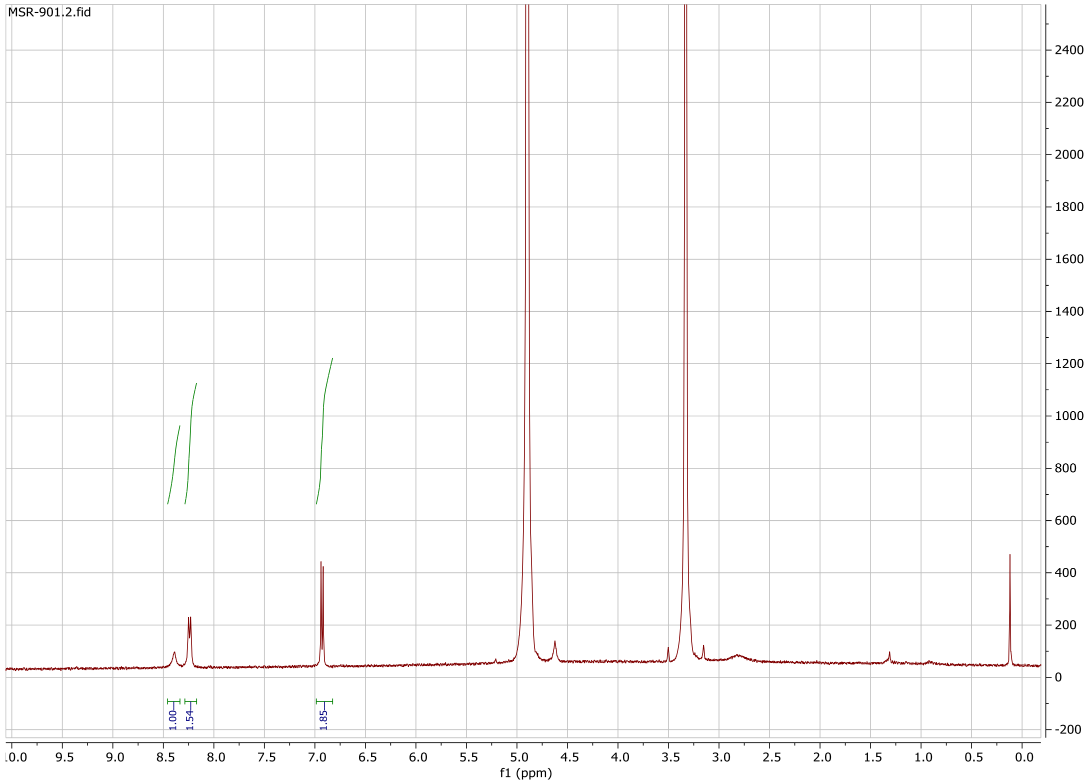

(4-Hydroxyphenyl)(1*H*-1,2,3-triazol-5-yl)methanone (108):^{13}C NMR (101 MHz, CD_3OD)

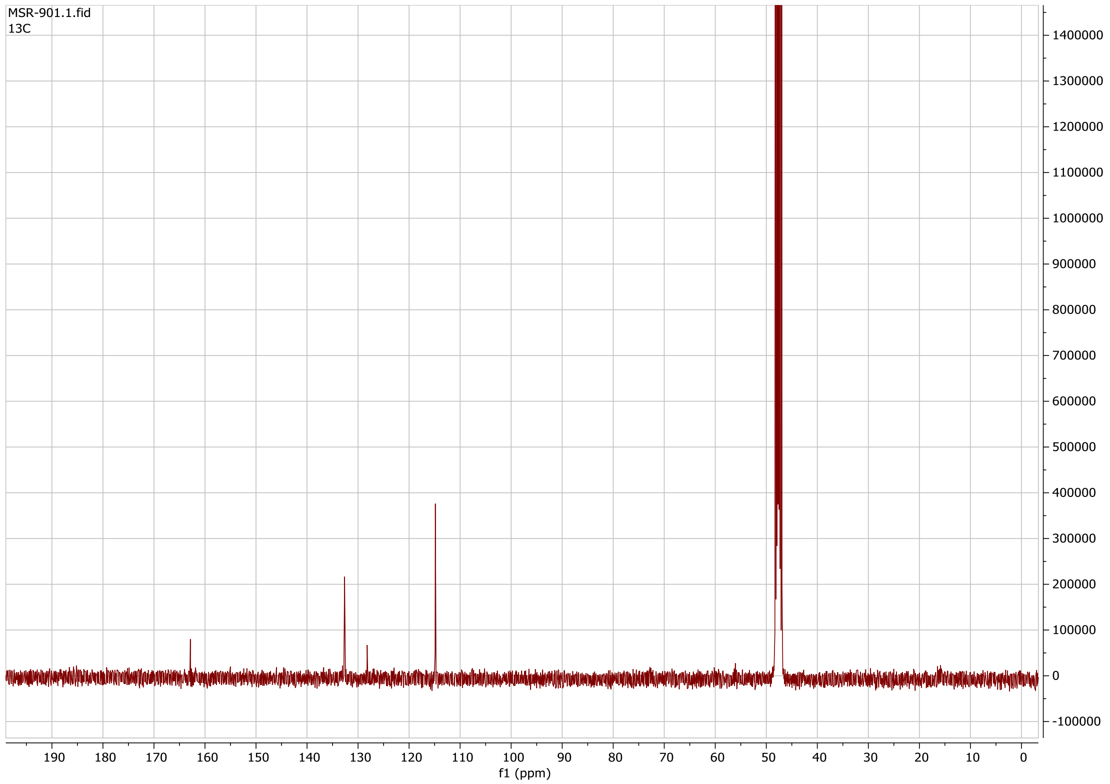

(4-Bromophenyl)(1*H*-1,2,3-triazol-5-yl)methanone (109): ^{1}H NMR (400 MHz, $CDCl_3$)

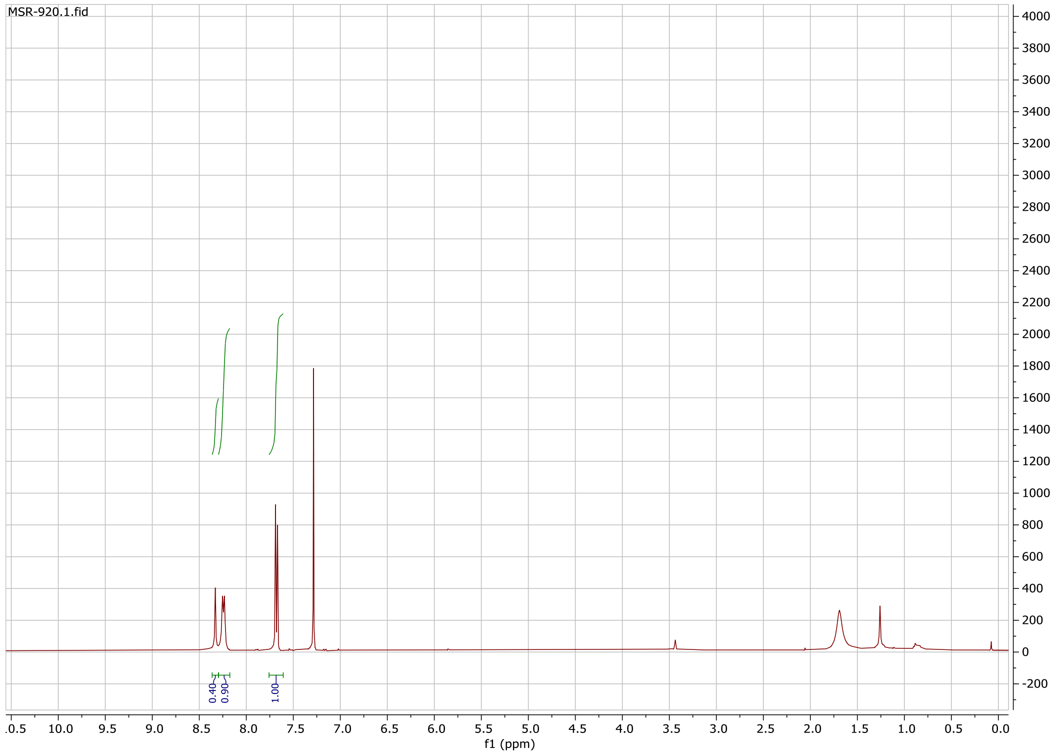

(4-Bromophenyl)(1*H*-1,2,3-triazol-5-yl)methanone (109): ^{13}C NMR (101 MHz, CD_3OD)

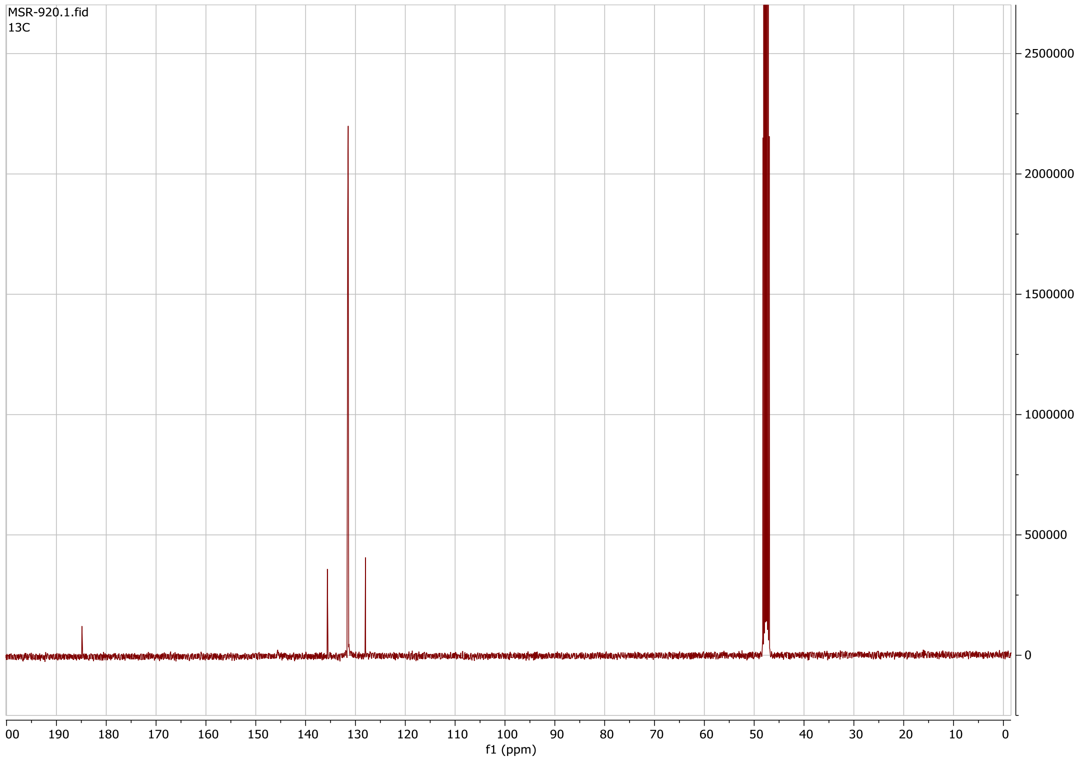

(4-Fluorophenyl)(1*H*-1,2,3-triazol-5-yl)methanone (110):^{1}H NMR (400 MHz, CD_3OD)

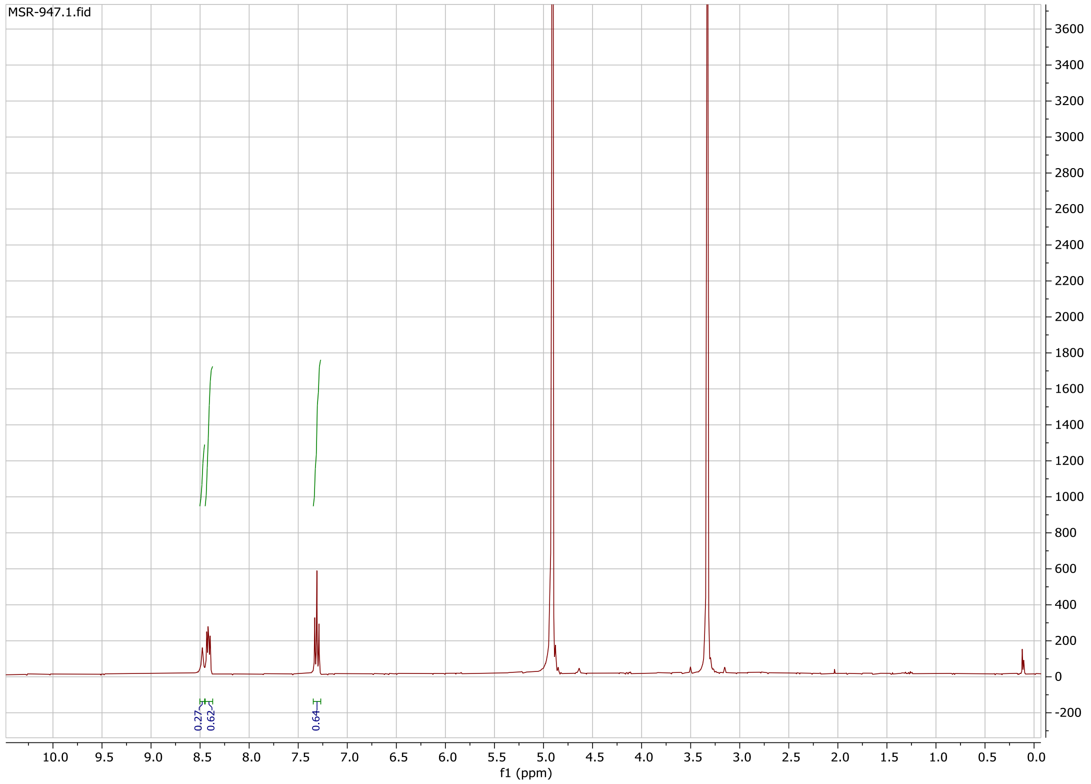

(4-Fluorophenyl)(1*H*-1,2,3-triazol-5-yl)methanone (110):^{13}C NMR (101 MHz, CD_3OD)

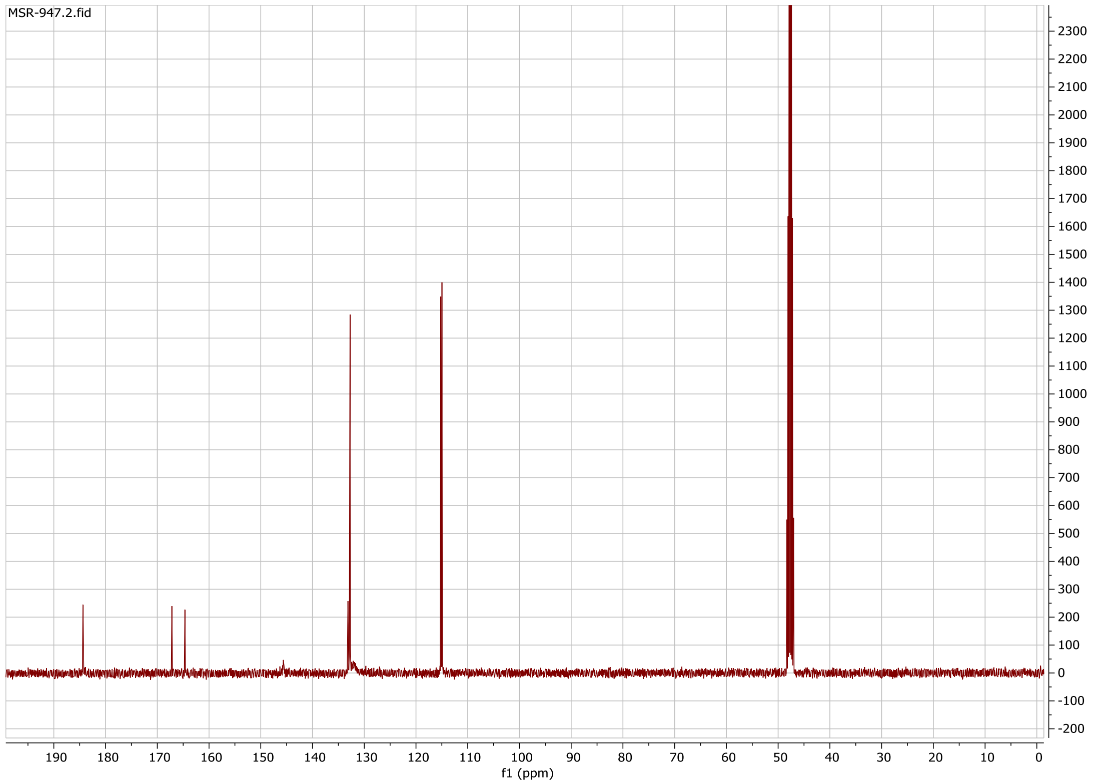

(5-Chloro-2-hydroxyphenyl)(1*H*-1,2,3-triazol-5-yl)methanone (111):^{1}H NMR (400 MHz, CD_3OD)

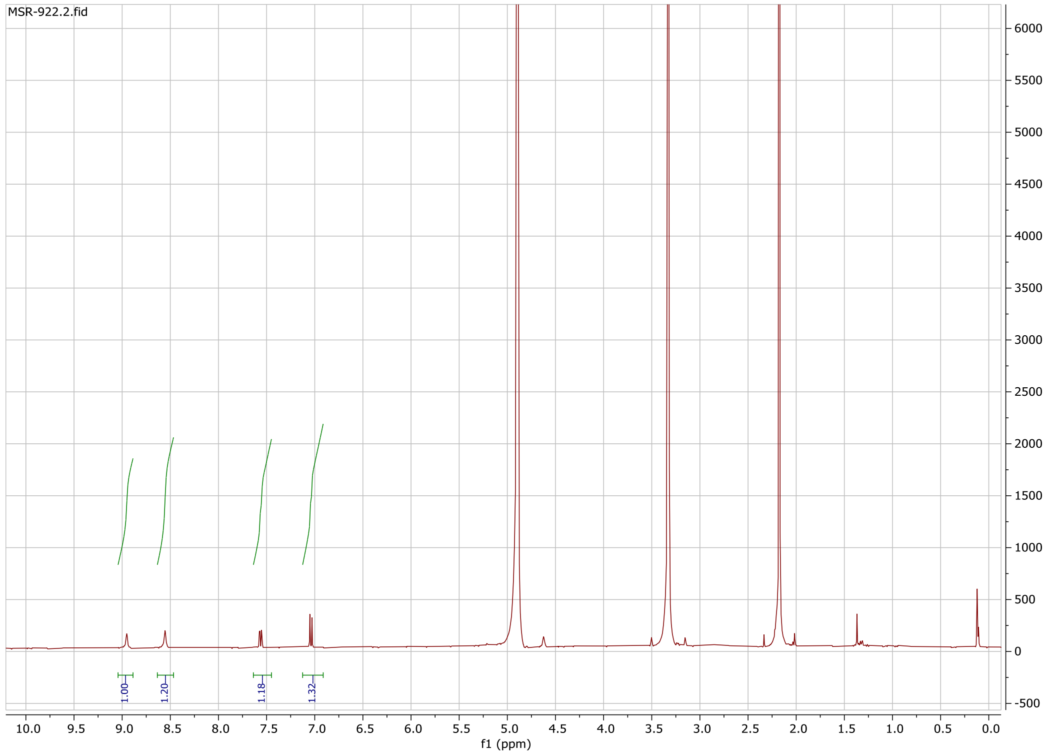

(5-Chloro-2-hydroxyphenyl)(1*H*-1,2,3-triazol-5-yl)methanone (111):^{13}C NMR (101 MHz, CD_3OD)

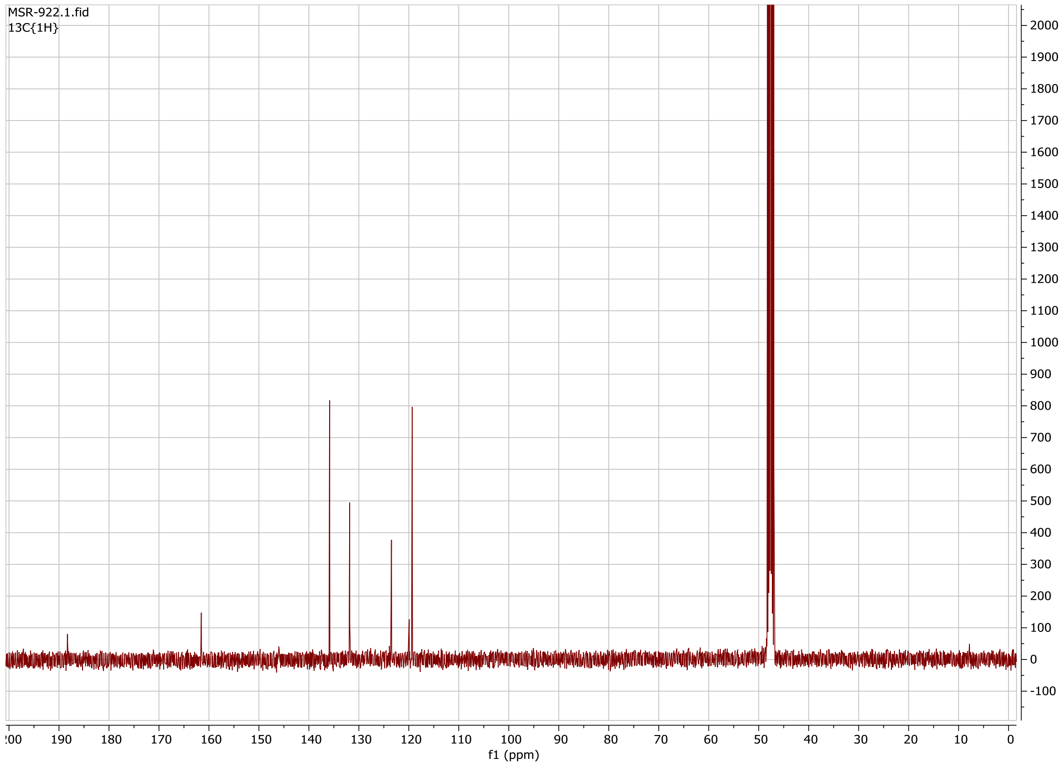

(4-Chloro-2-hydroxyphenyl)(1*H*-1,2,3-triazol-5-yl)methanone (112):^{1}H NMR (400 MHz, CD_3OD)

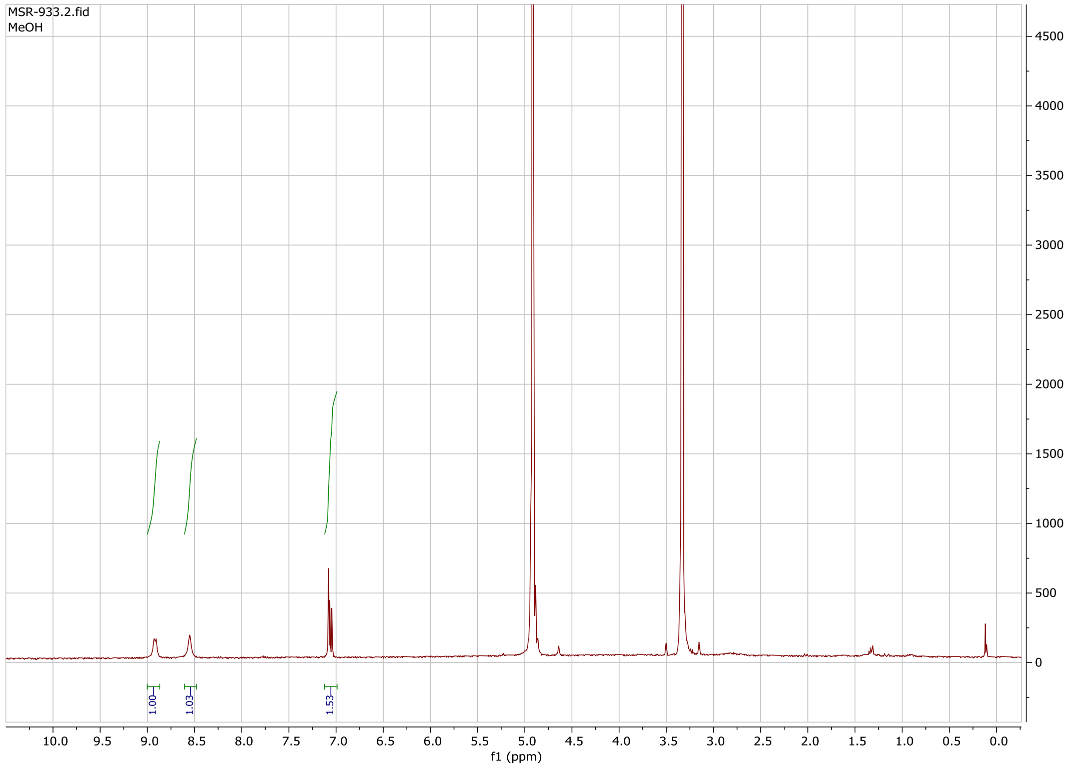

(4-Chloro-2-hydroxyphenyl)(1*H*-1,2,3-triazol-5-yl)methanone (112):^{13}C NMR (101 MHz, CD_3OD)

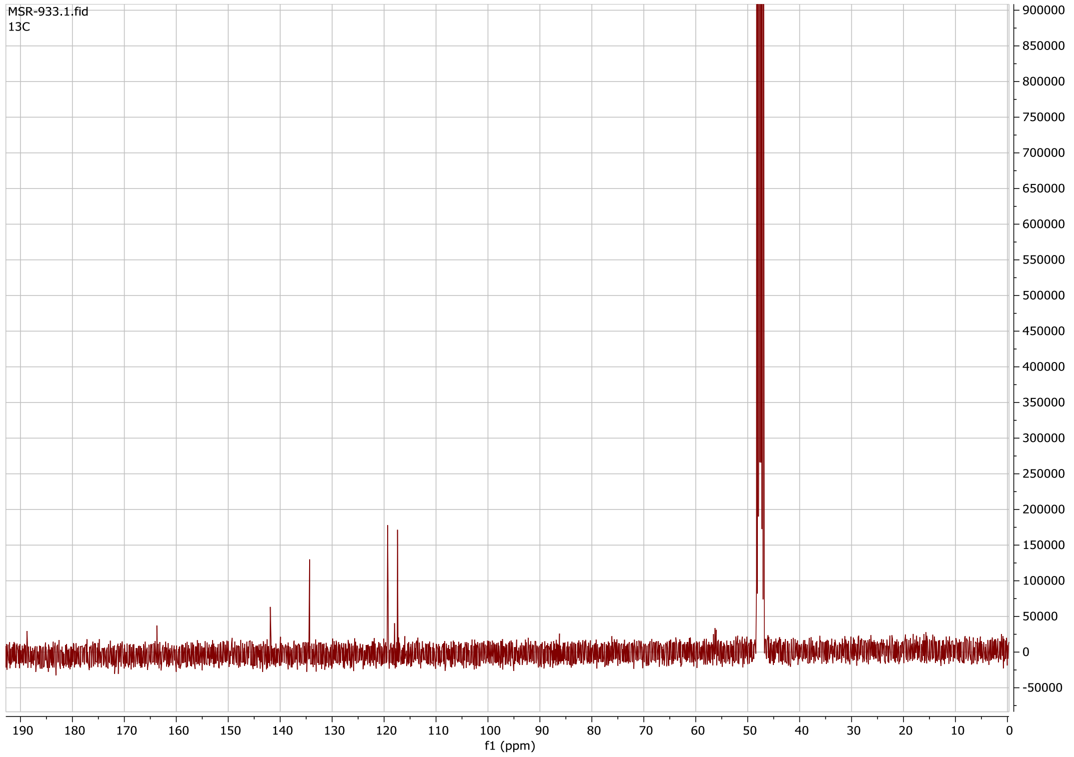

(4-Bromo-2-hydroxyphenyl)(1*H*-1,2,3-triazol-5-yl)methanone (113):^{1}H NMR (400 MHz, CD_3OD)

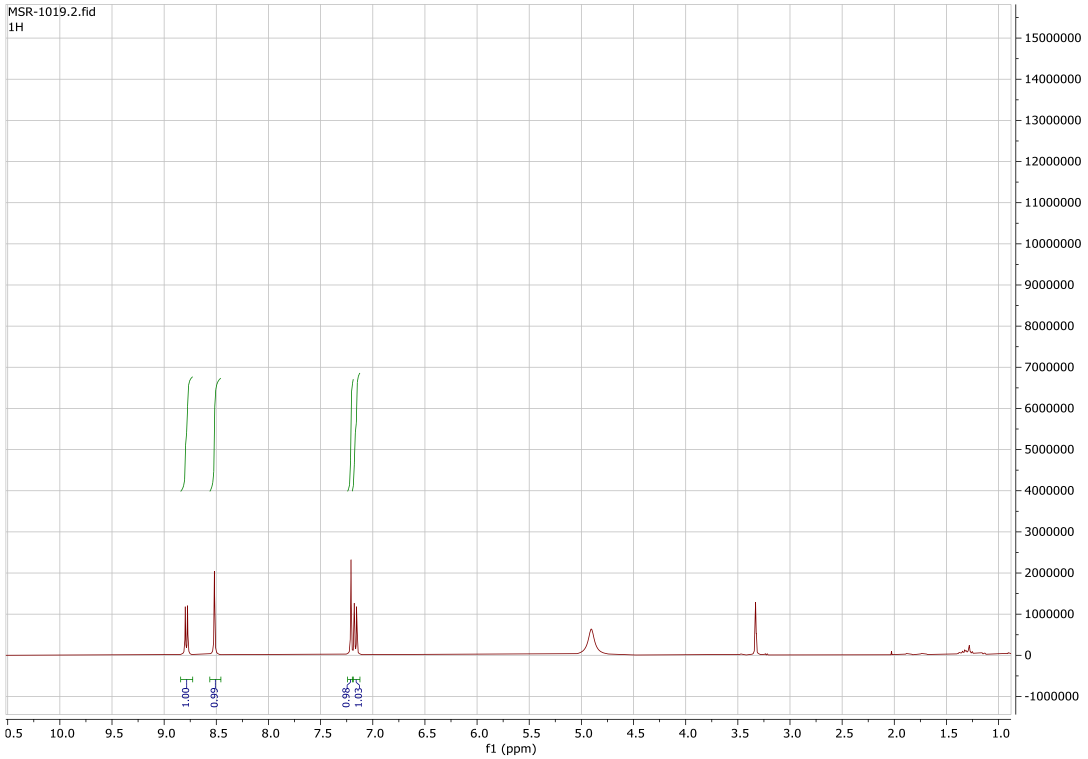

(4-Bromo-2-hydroxyphenyl)(1*H*-1,2,3-triazol-5-yl)methanone (113): ^{13}C NMR (101 MHz, CD_3OD)

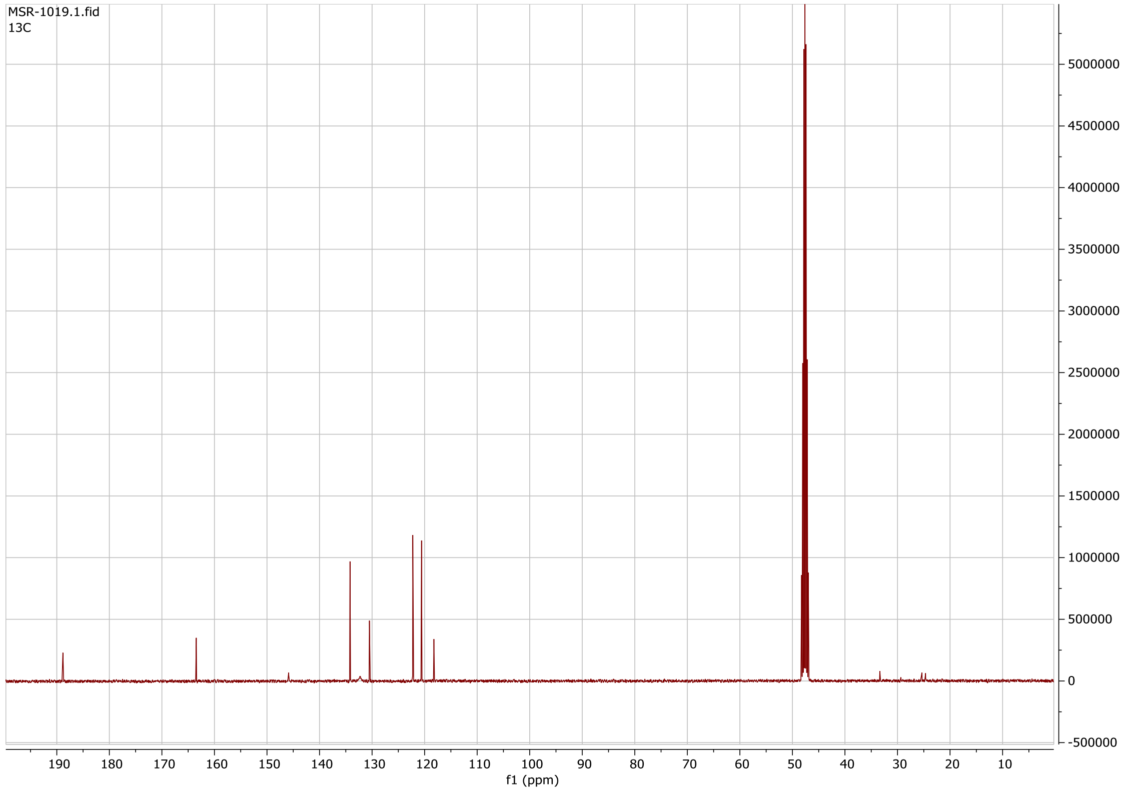

Furan-2-yl(1*H*-1,2,3-triazol-5-yl)methanone (114):^{1}H NMR (400 MHz, CD_3OD)

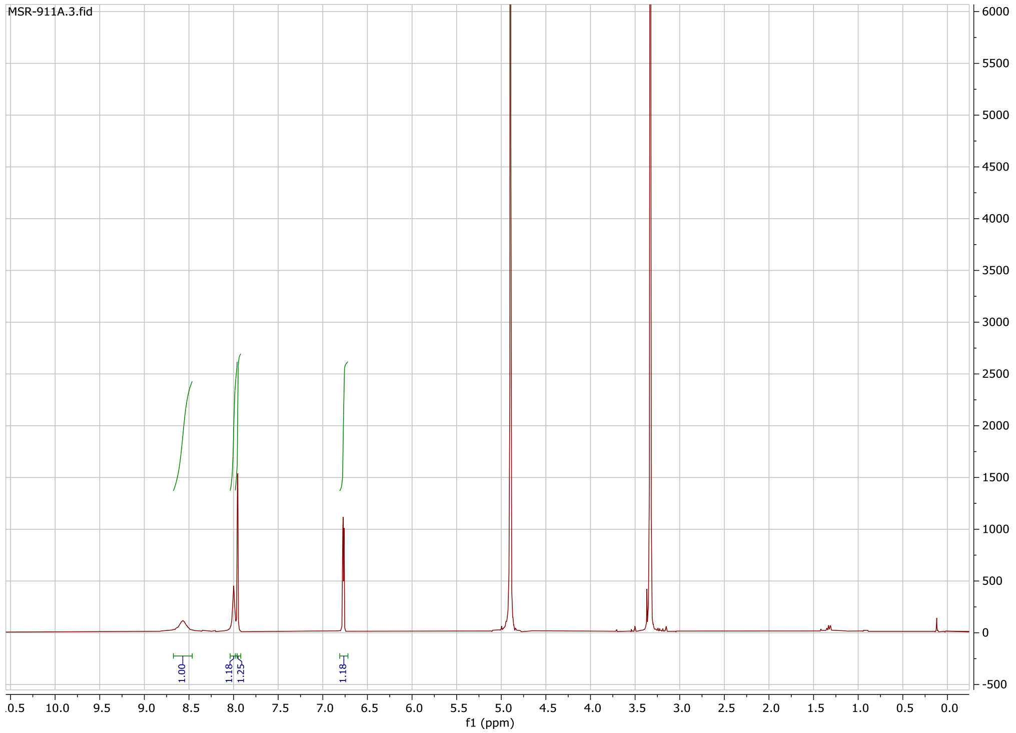

Furan-2-yl(1*H*-1,2,3-triazol-5-yl)methanone (114):^{13}C NMR (101 MHz, CD_3OD)

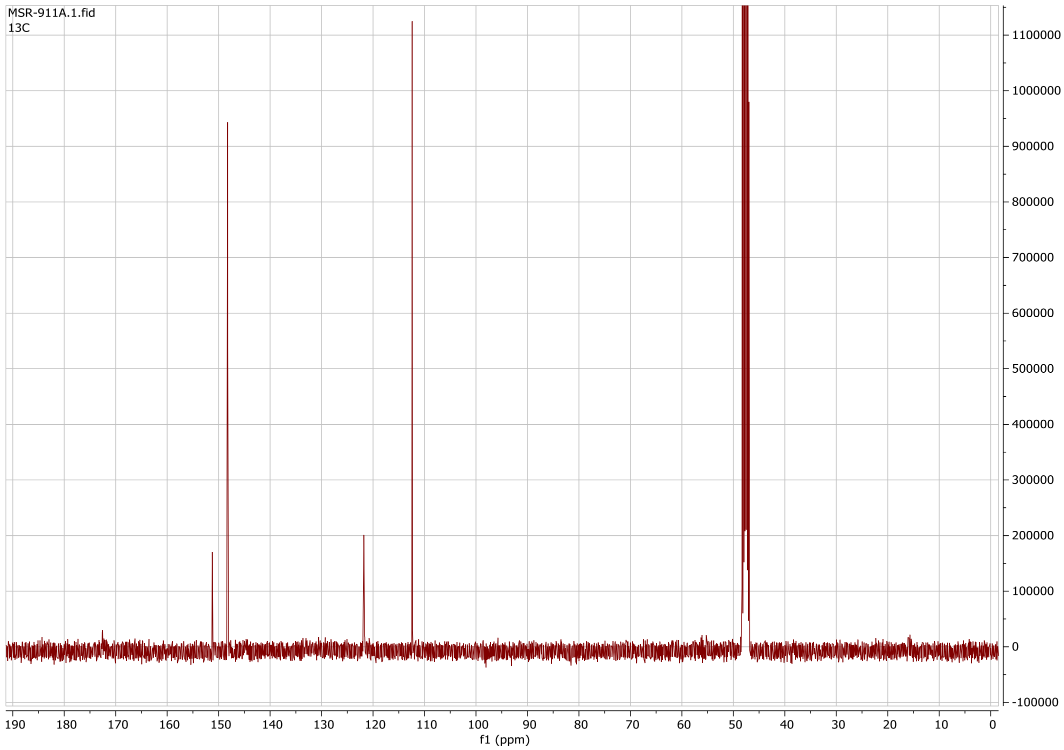

Furan-3-yl(1*H*-1,2,3-triazol-5-yl)methanone (115): ^{1}H NMR (400 MHz, CD_3OD)

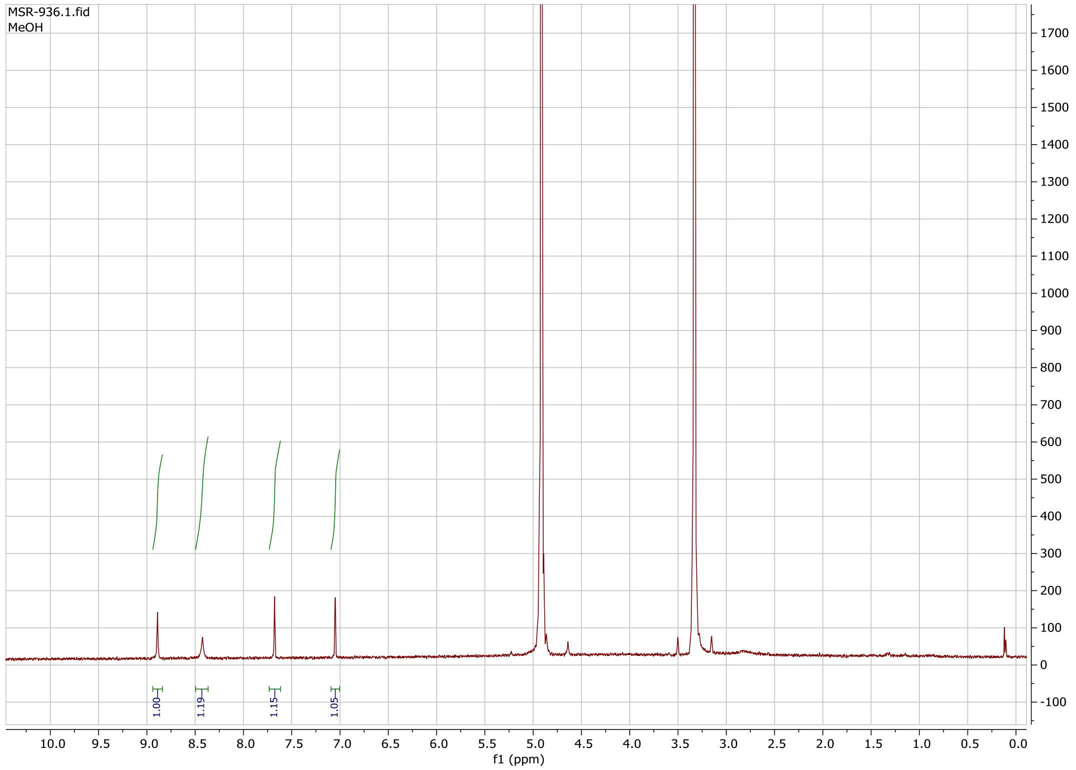

Furan-3-yl(1*H*-1,2,3-triazol-5-yl)methanone (115): ^{13}C NMR (101 MHz, CD_3OD)

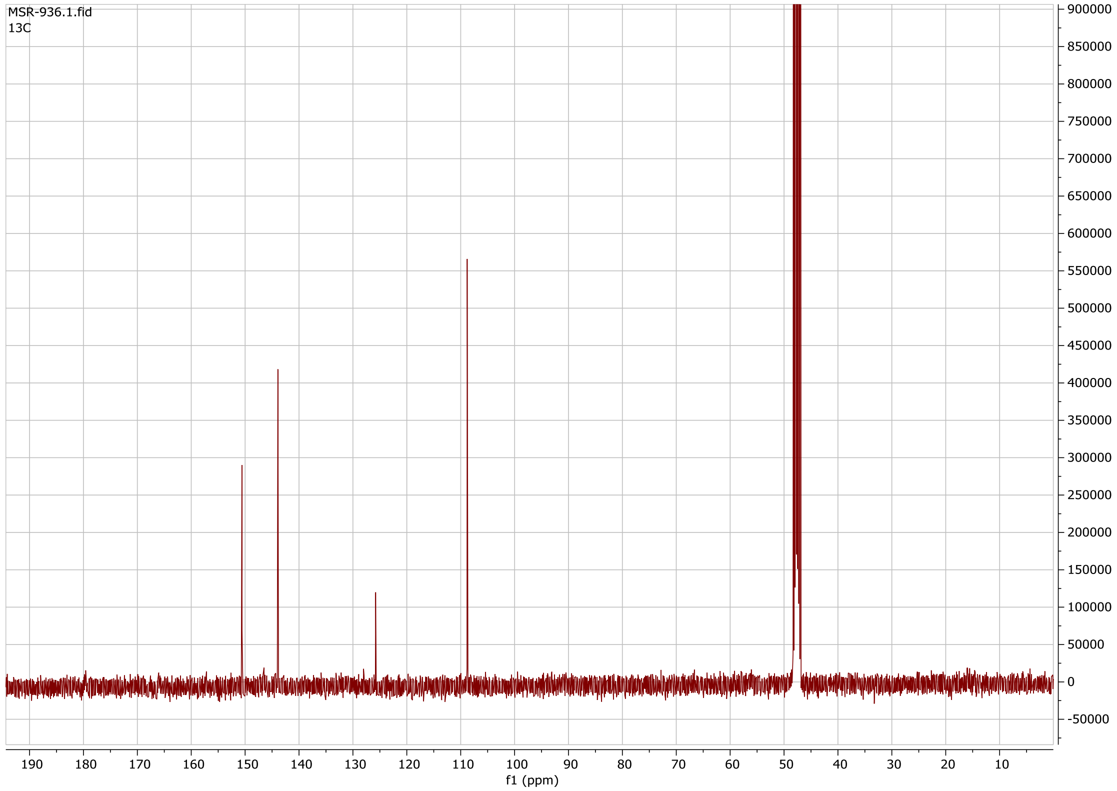

Thiophen-2-yl(1*H*-1,2,3-triazol-5-yl)methanone (116):^{1}H NMR (400 MHz, CD_3OD)

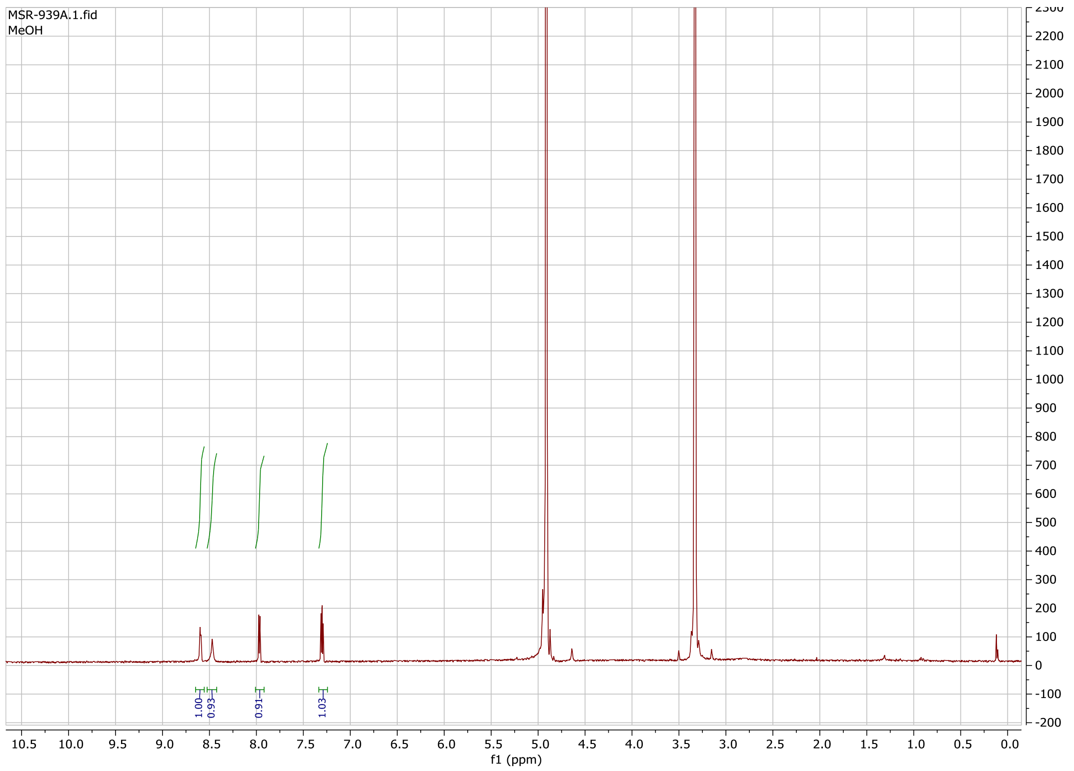

Thiophen-2-yl(1*H*-1,2,3-triazol-5-yl)methanone (116):^{13}C NMR (101 MHz, CD_3OD)

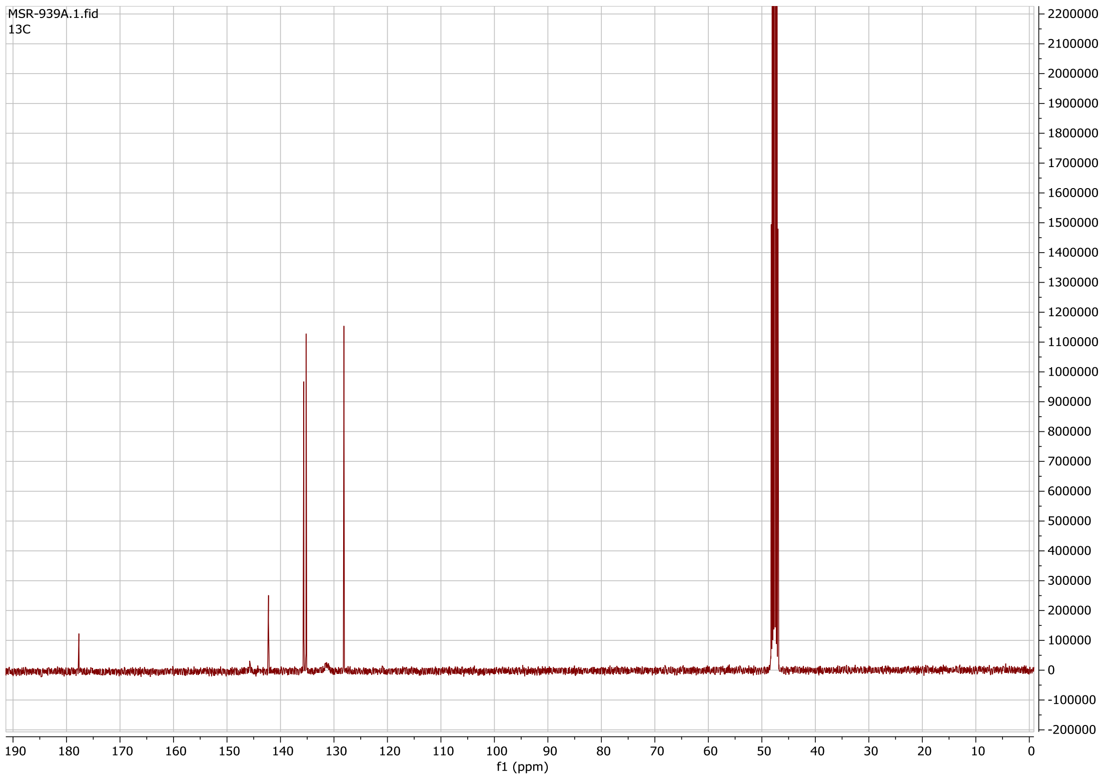

Thiophen-3-yl(1*H*-1,2,3-triazol-5-yl)methanone (117):^{1}H NMR (400 MHz, CD_3OD)

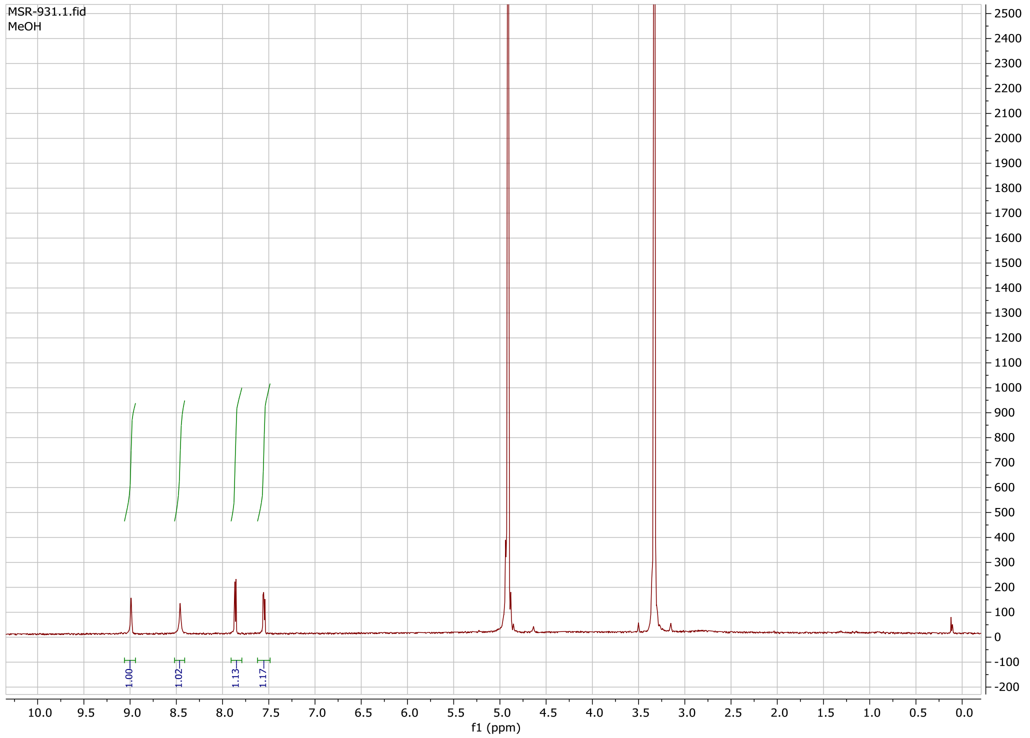

Thiophen-3-yl(1*H*-1,2,3-triazol-5-yl)methanone (117):^{13}C NMR (101 MHz, CD_3OD)

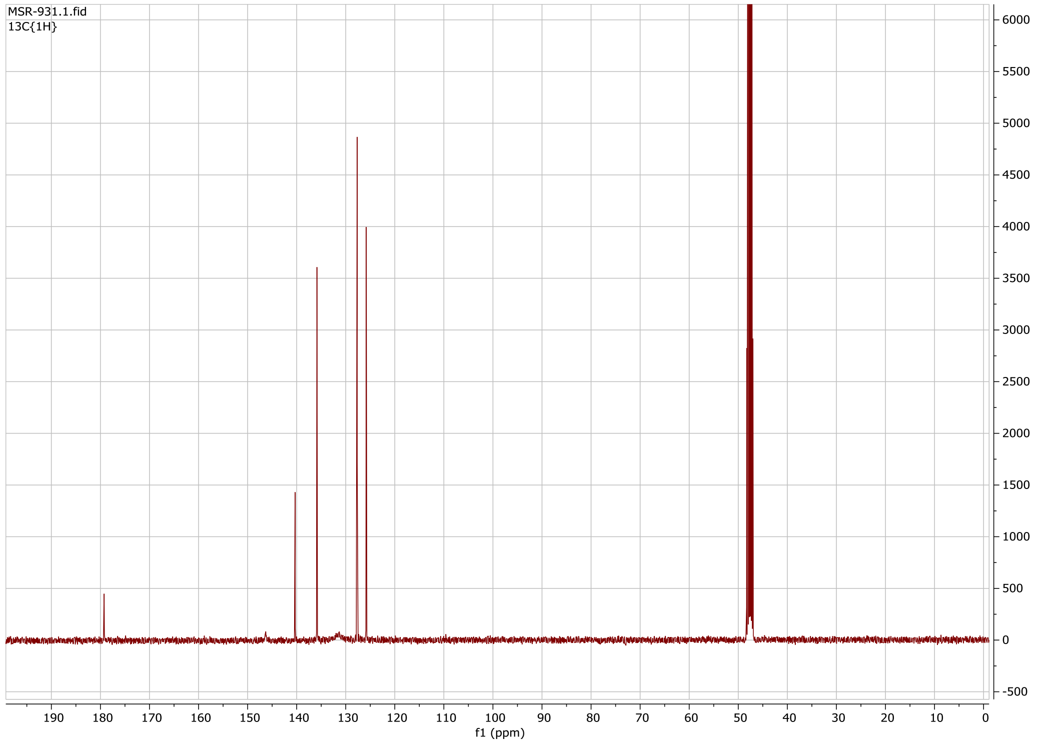

Thiazol-4-yl(1*H*-1,2,3-triazol-5-yl)methanone (118): ^{1}H NMR (400 MHz, CD_3OD)

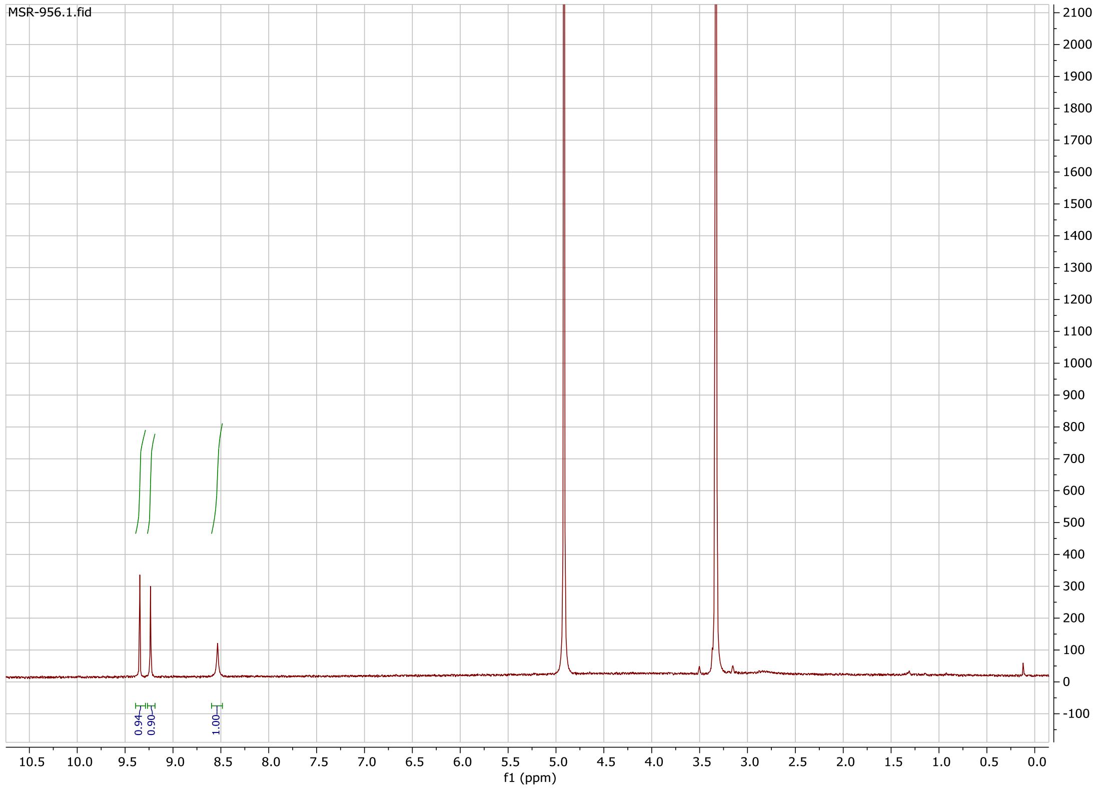

Thiazol-4-yl(1*H*-1,2,3-triazol-5-yl)methanone (118): ^{13}C NMR (101 MHz, CD_3OD)

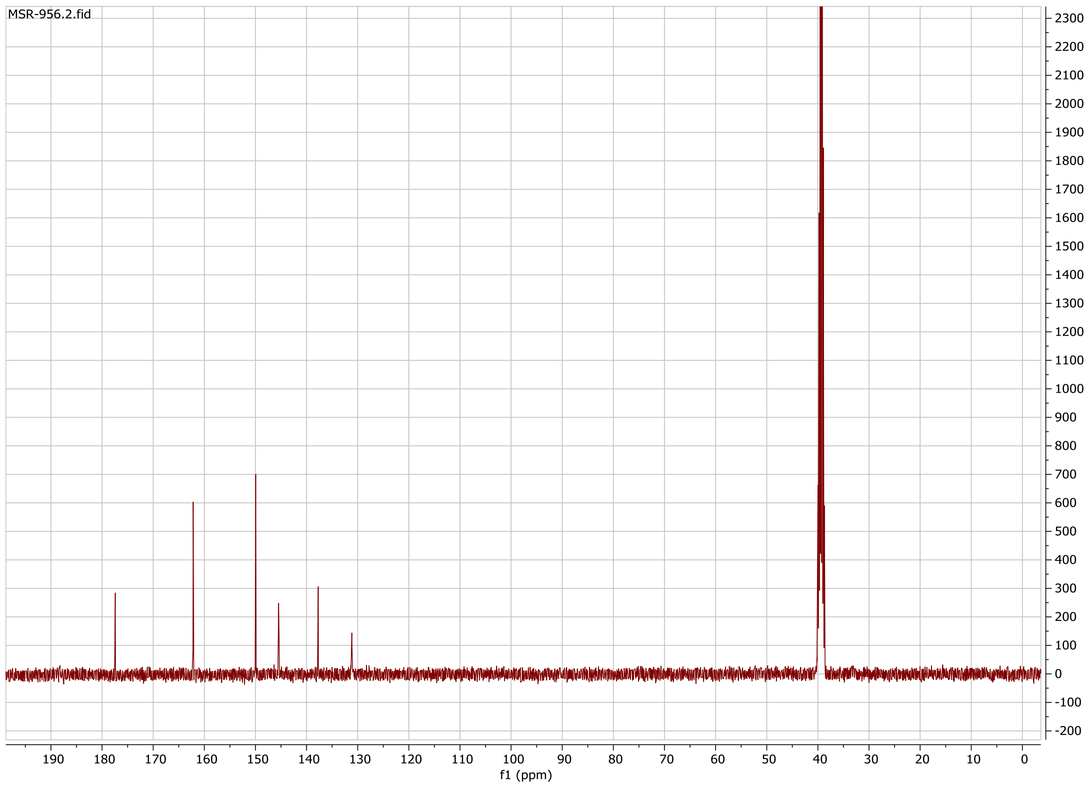

(1,2,3-Thiadiazol-4-yl)(1*H*-1,2,3-triazol-5-yl)methanone (119): ^{1}H NMR (400 MHz, CD_3OD)

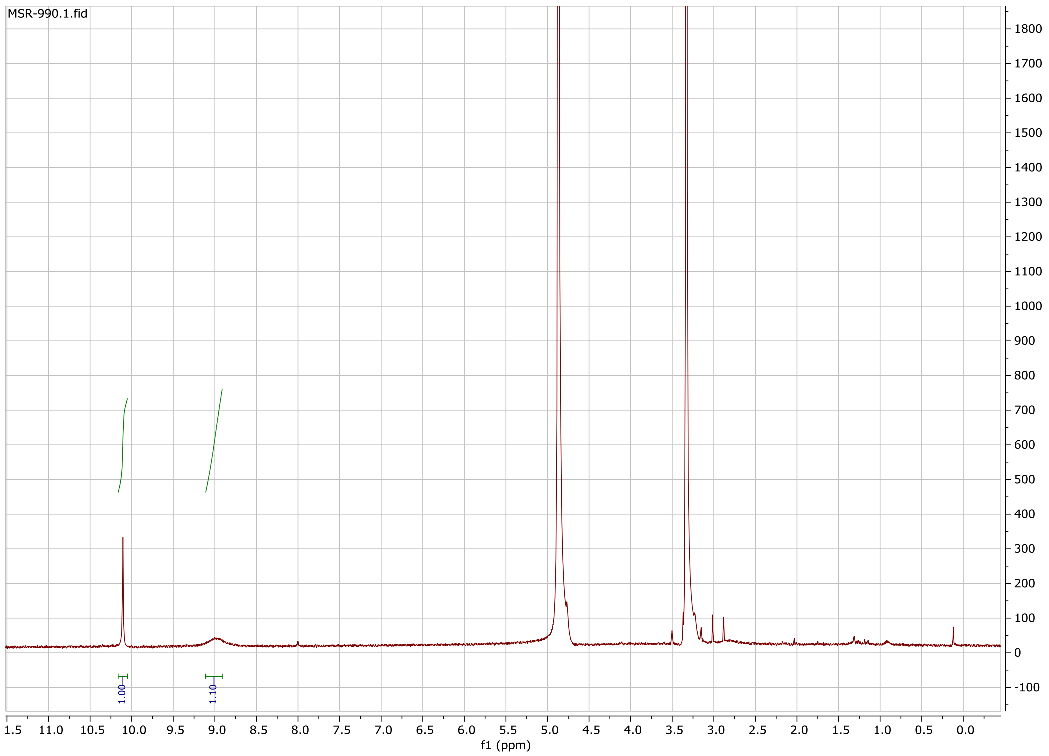

(1,2,3-Thiadiazol-4-yl)(1*H*-1,2,3-triazol-5-yl)methanone (119): ^{13}C NMR (101 MHz, CD_3OD)

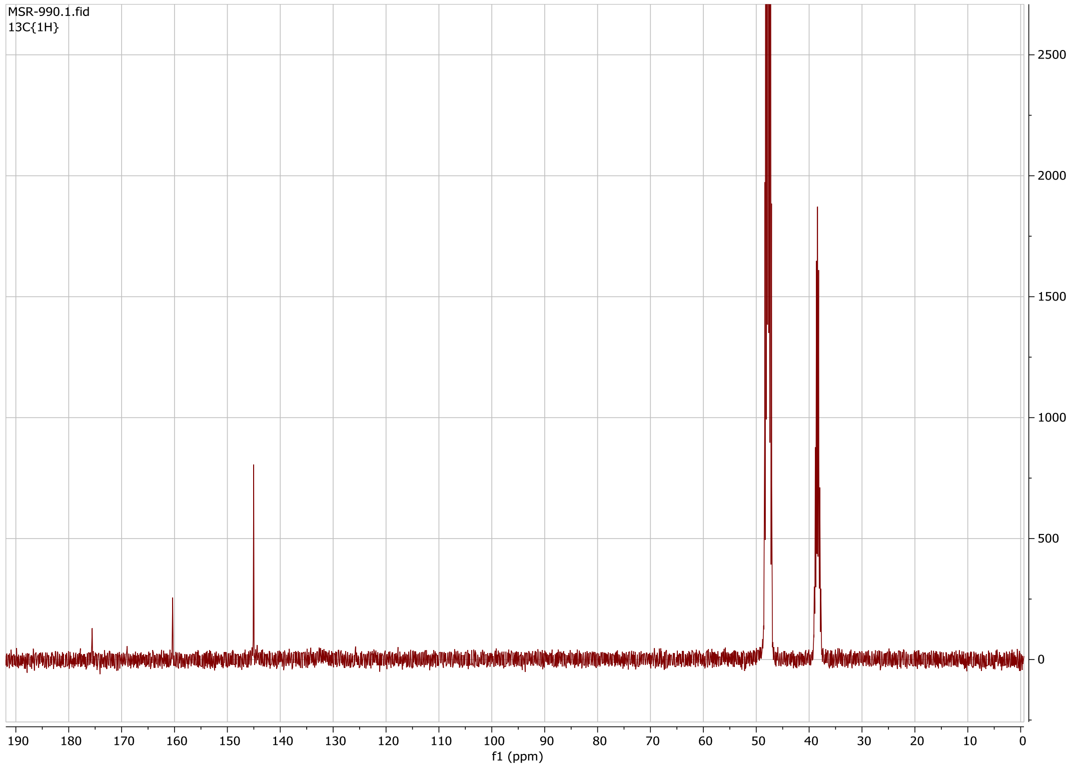

Pyridin-2-yl(1*H*-1,2,3-triazol-5-yl)methanone (120):^{1}H NMR (400 MHz, CD_3OD)

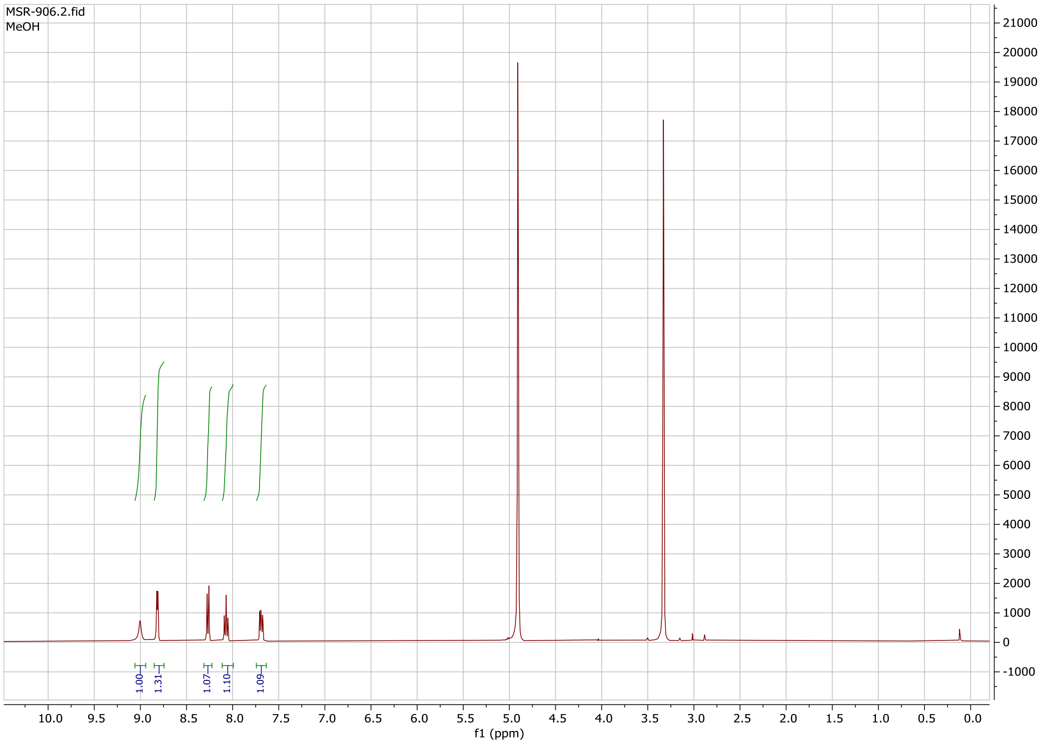

Pyridin-2-yl(1*H*-1,2,3-triazol-5-yl)methanone (120):^{13}C NMR (101 MHz, CD_3OD)

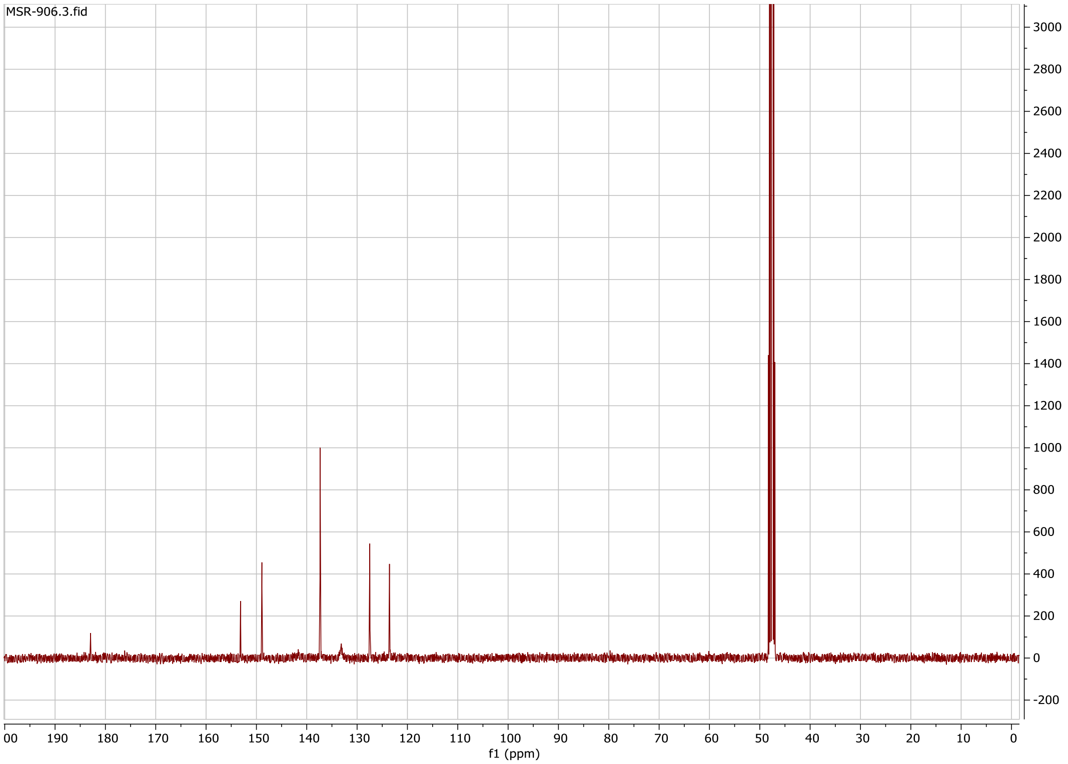

Pyridin-3-yl(1*H*-1,2,3-triazol-5-yl)methanone (121): ^{1}H NMR (400 MHz, CD_3OD)

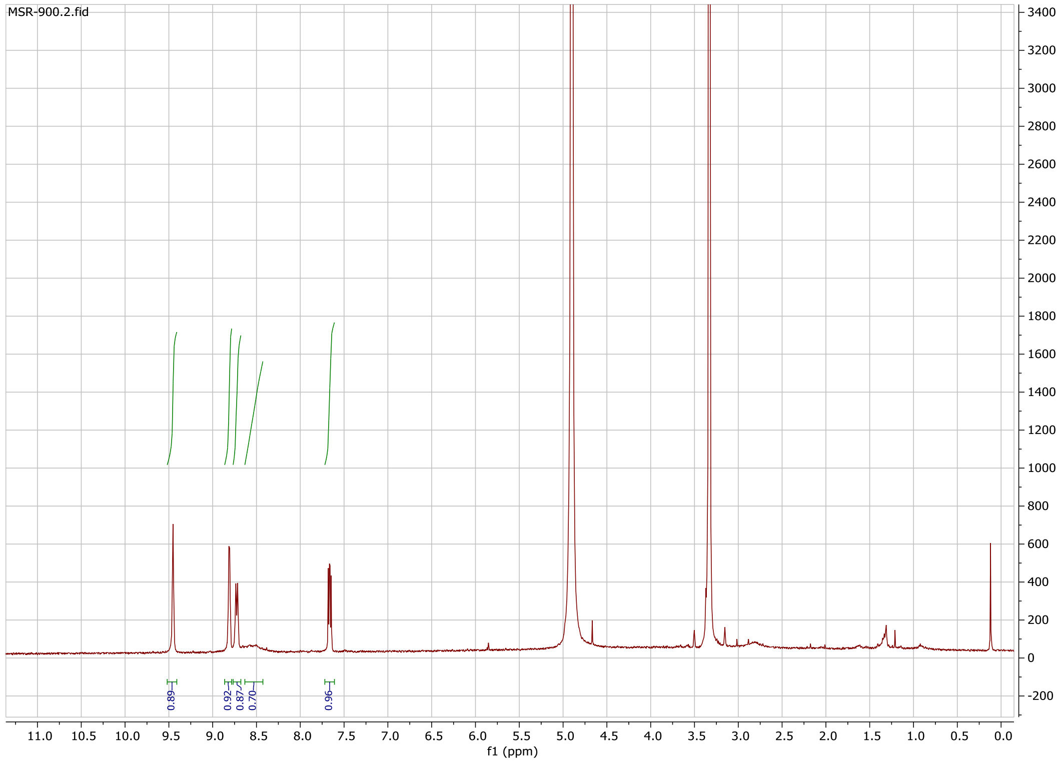

Pyridin-3-yl(1*H*-1,2,3-triazol-5-yl)methanone (121): ^{13}C NMR (101 MHz, CD_3OD)

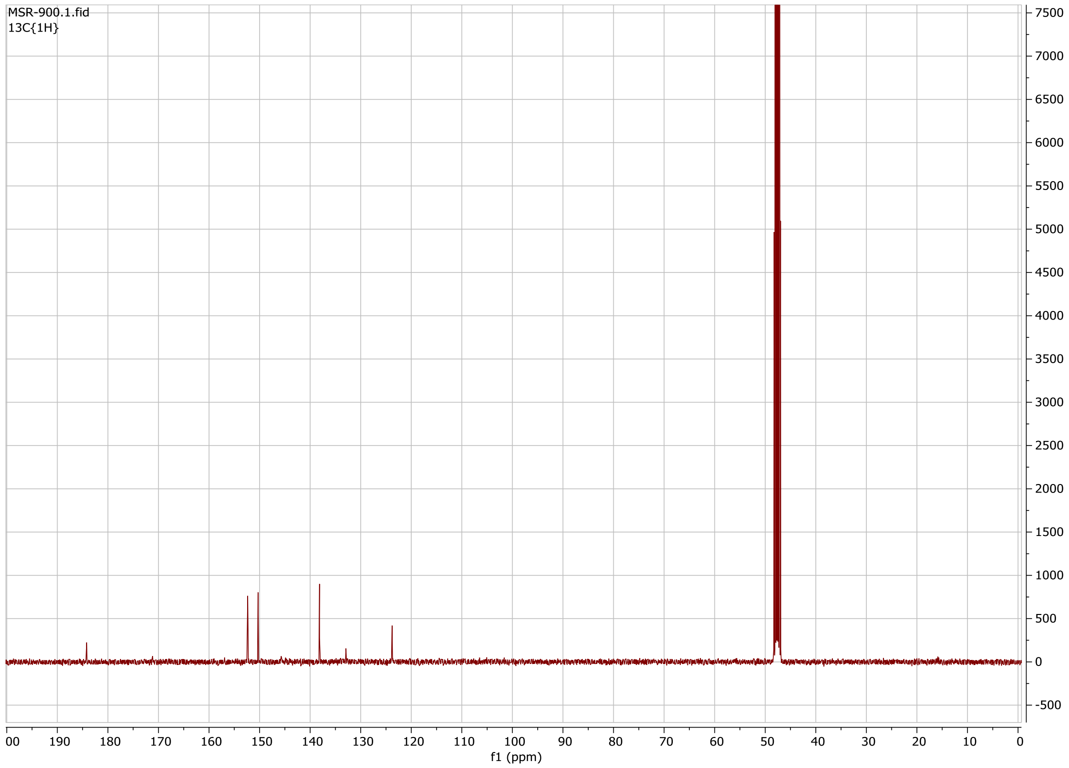

Pyridin-4-yl(1*H*-1,2,3-triazol-5-yl)methanone (122):^{1}H NMR (400 MHz, CD_3OD)

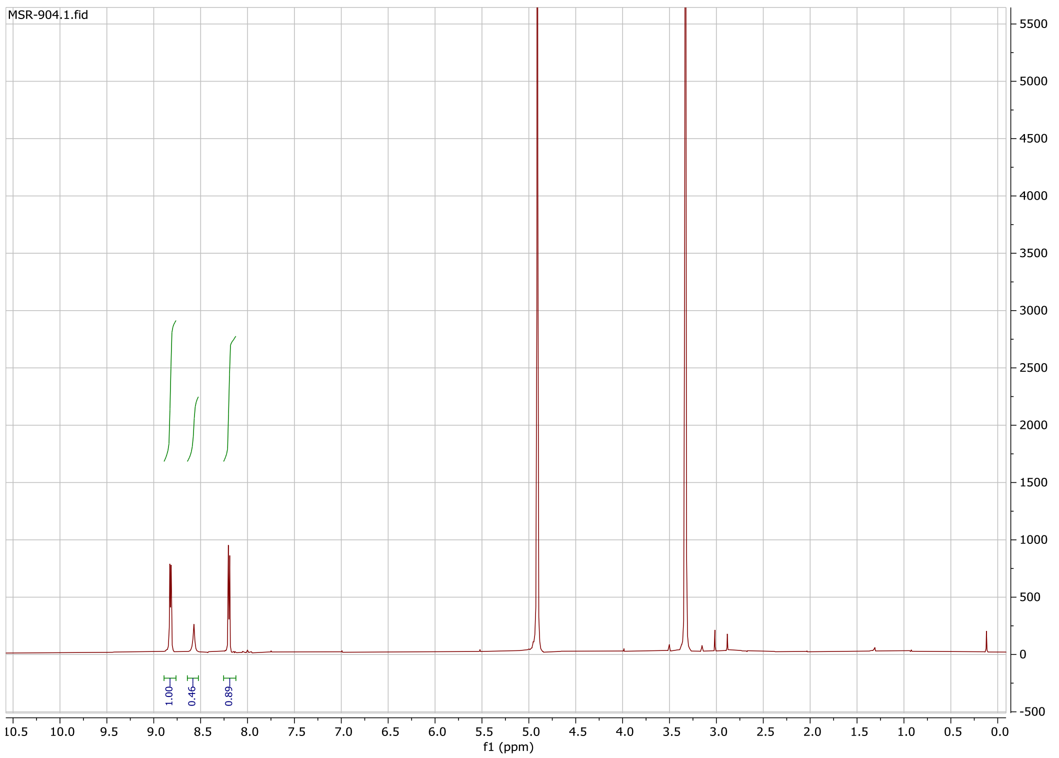

Pyridin-4-yl(1*H*-1,2,3-triazol-5-yl)methanone (122):^{13}C NMR (101 MHz, CD_3OD)

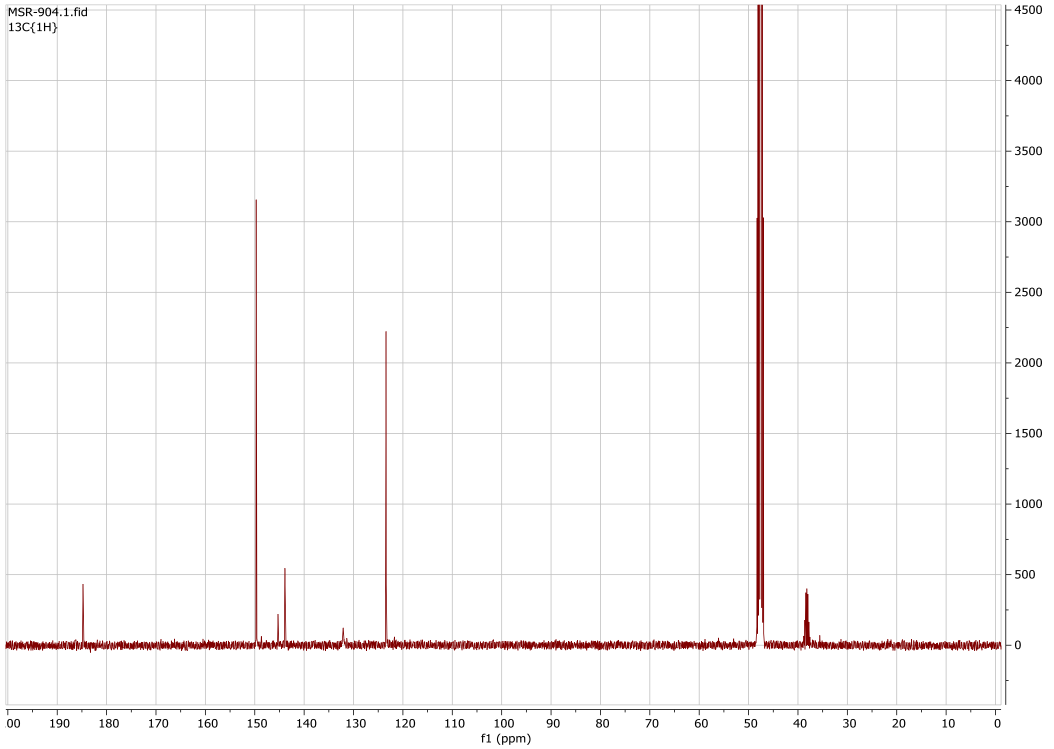

(4-Bromophenyl)(4-(hydroxymethyl)-1*H*-1,2,3-triazol-5-yl)methanone (123):^{1}H NMR (400 MHz, CD_3OD)

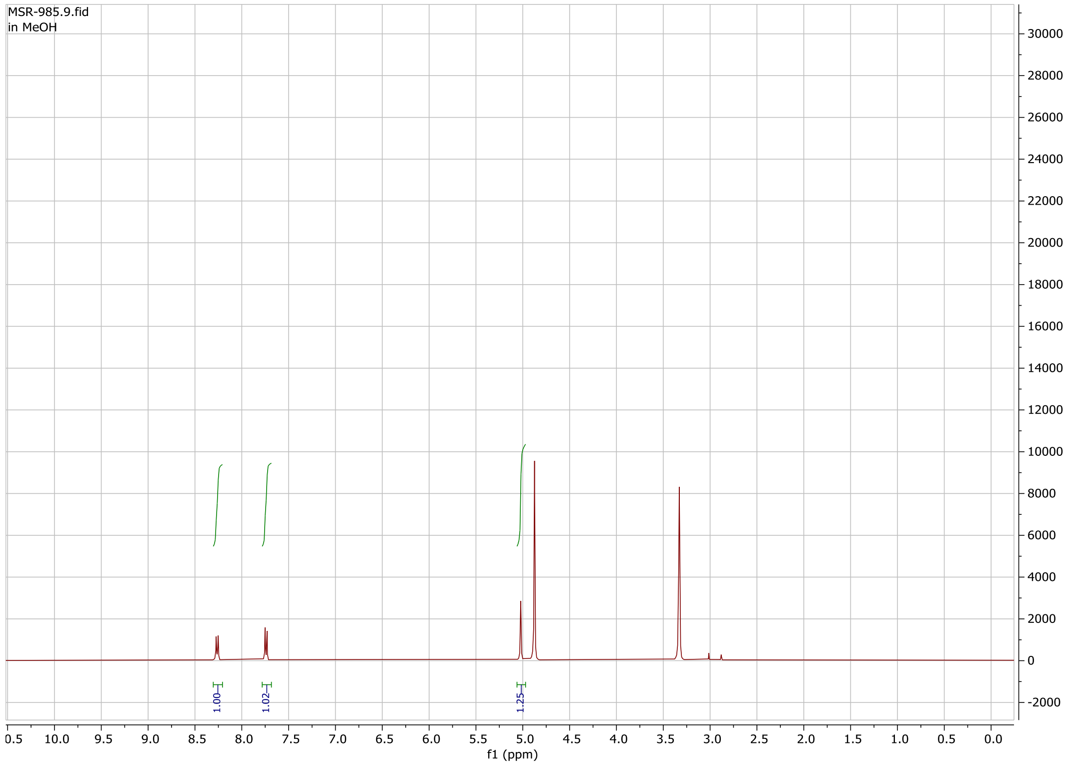

(4-Bromophenyl)(4-(hydroxymethyl)-1*H*-1,2,3-triazol-5-yl)methanone (123):^{13}C NMR (101 MHz, CD_3OD)

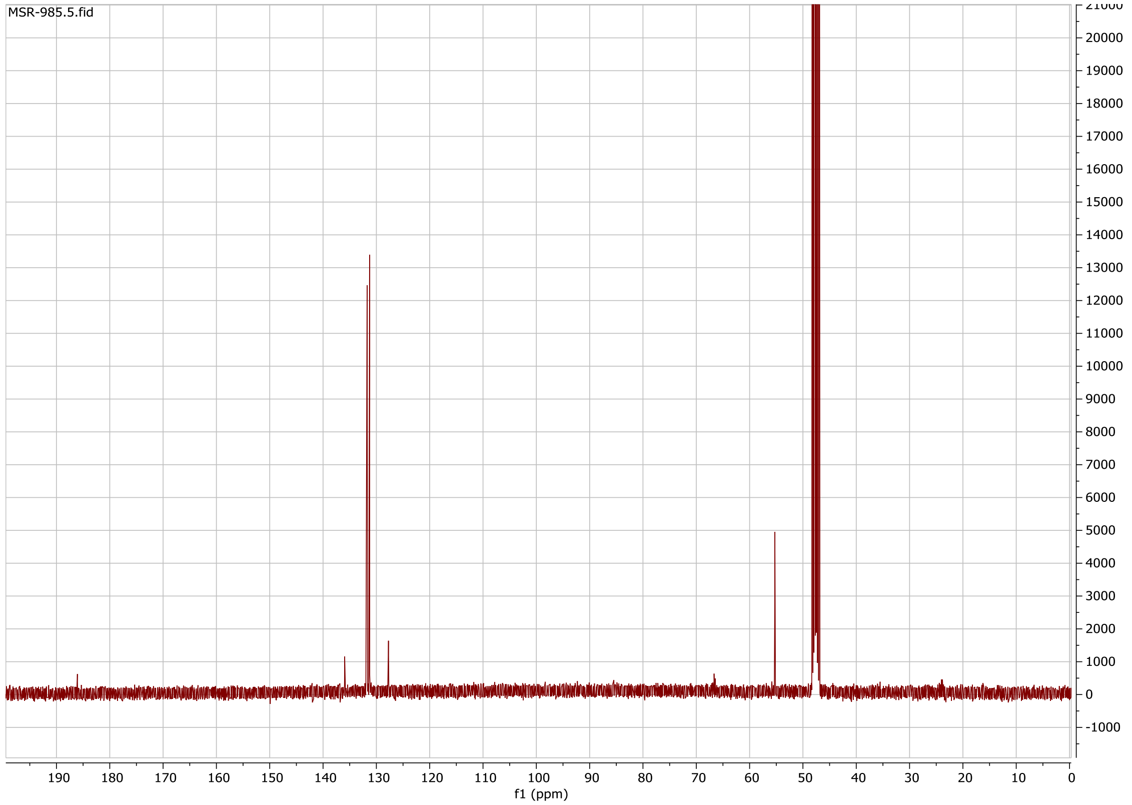

(4-(Bromomethyl)-1*H*-1,2,3-triazol-5-yl)(4-bromophenyl)methanone (124): ^{1}H NMR (400 MHz, $CDCl_3$)

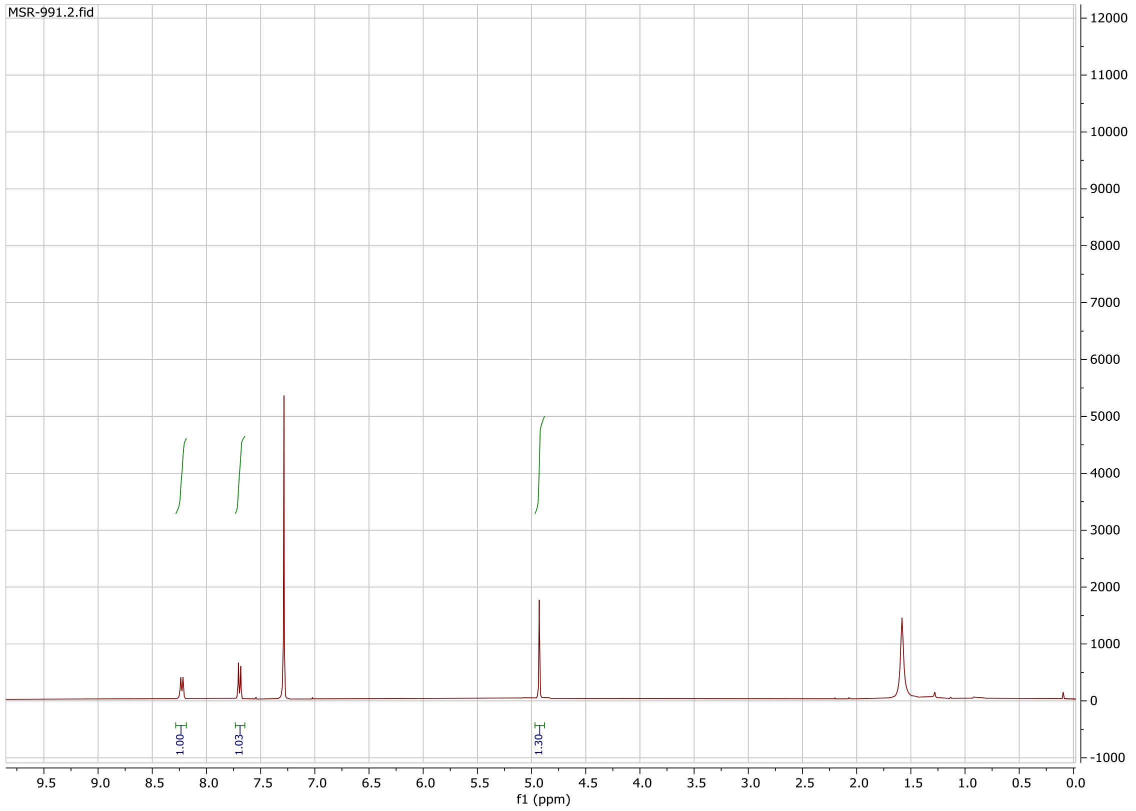

(4-(Bromomethyl)-1*H*-1,2,3-triazol-5-yl)(4-bromophenyl)methanone (124): ^{13}C NMR (101 MHz, CD_3OD)

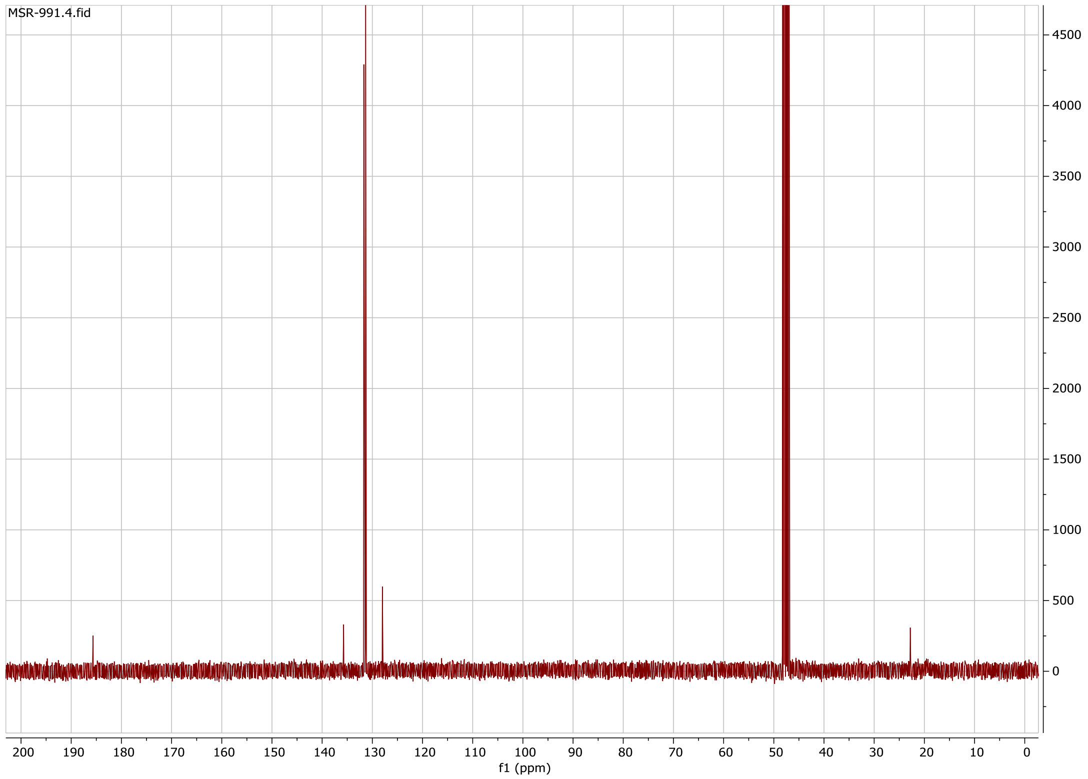

(4-(aminomethyl)-1*H*-1,2,3-triazol-5-yl)(4-bromophenyl)methanone (126):^{1}H NMR (400 MHz, CD_3OD)

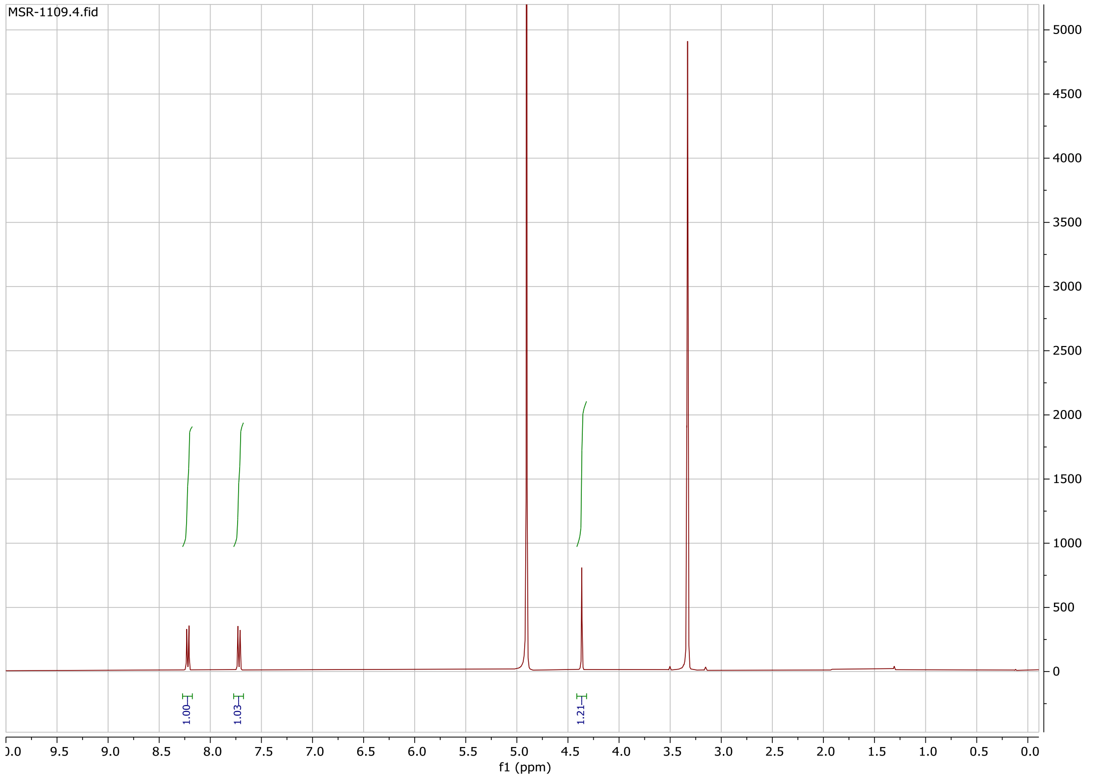

(4-(aminomethyl)-1*H*-1,2,3-triazol-5-yl)(4-bromophenyl)methanone (126):^{13}C NMR (101 MHz, CD_3OD)

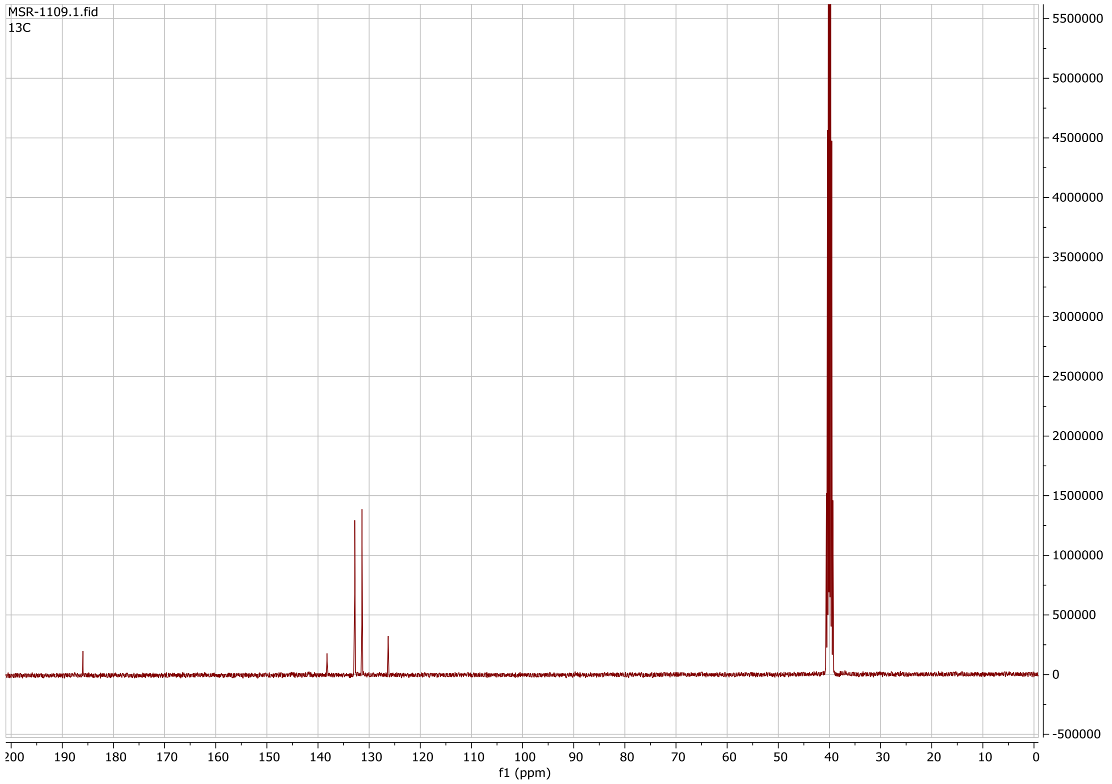

3-(4-Fluorophenyl)-1*H*-1,2,4-triazole (134):^{1}H NMR (400 MHz, $CDCl_3$)

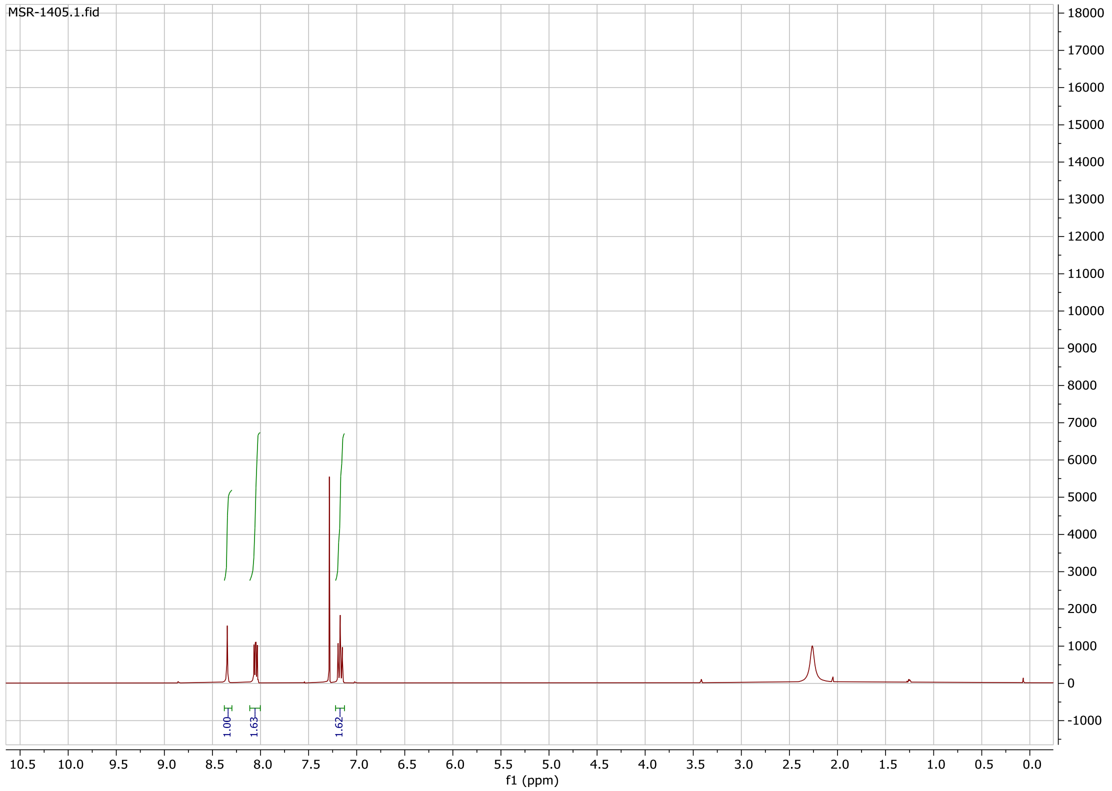

3-(4-Fluorophenyl)-1*H*-1,2,4-triazole (134):^{13}C NMR (101 MHz, CD_3OD)

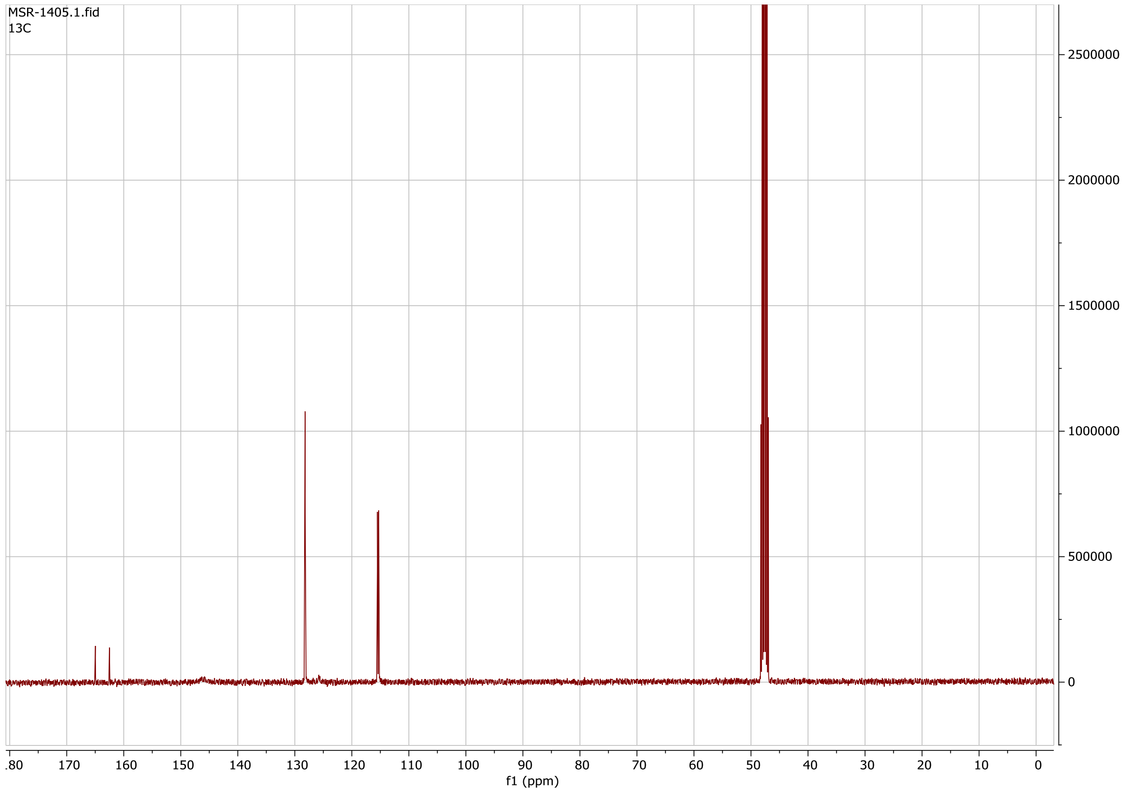

3-(3-Bromophenyl)-1*H* -1,2,4-triazole (135): ^{1}H NMR (400 MHz, CD_3OD)

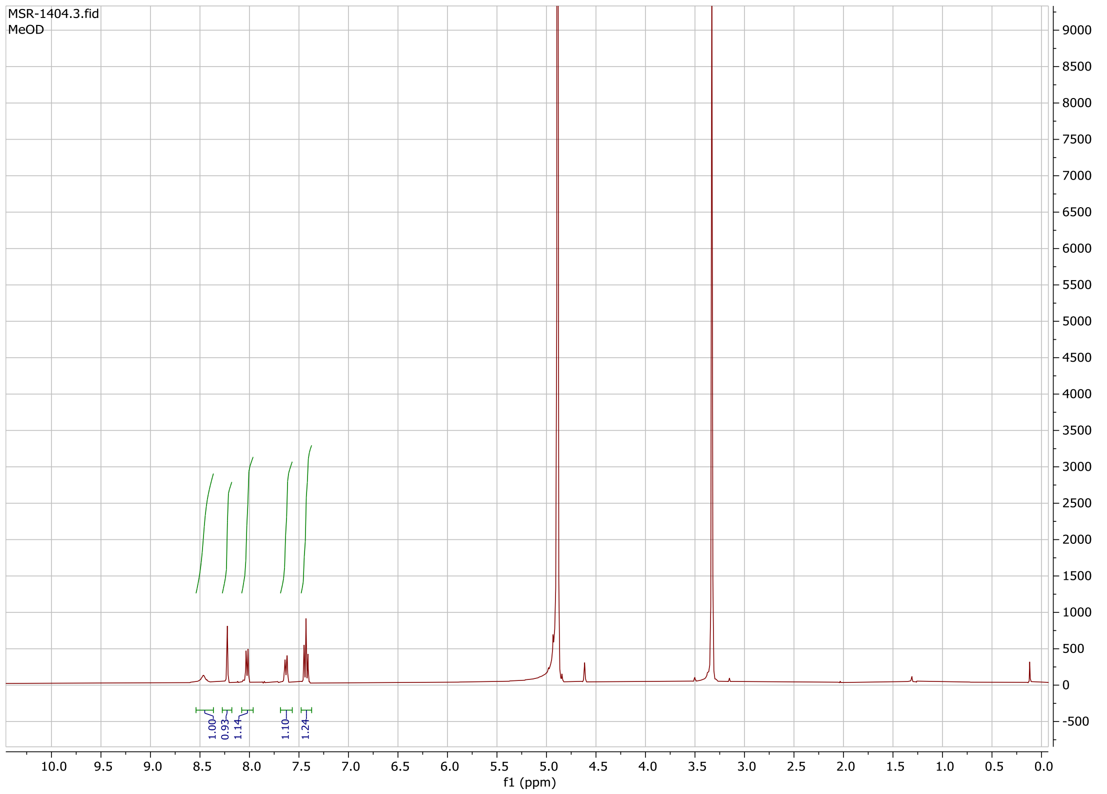

3-(3-Bromophenyl)-1*H* -1,2,4-triazole (135): ^{13}C NMR (101 MHz, CD_3OD)

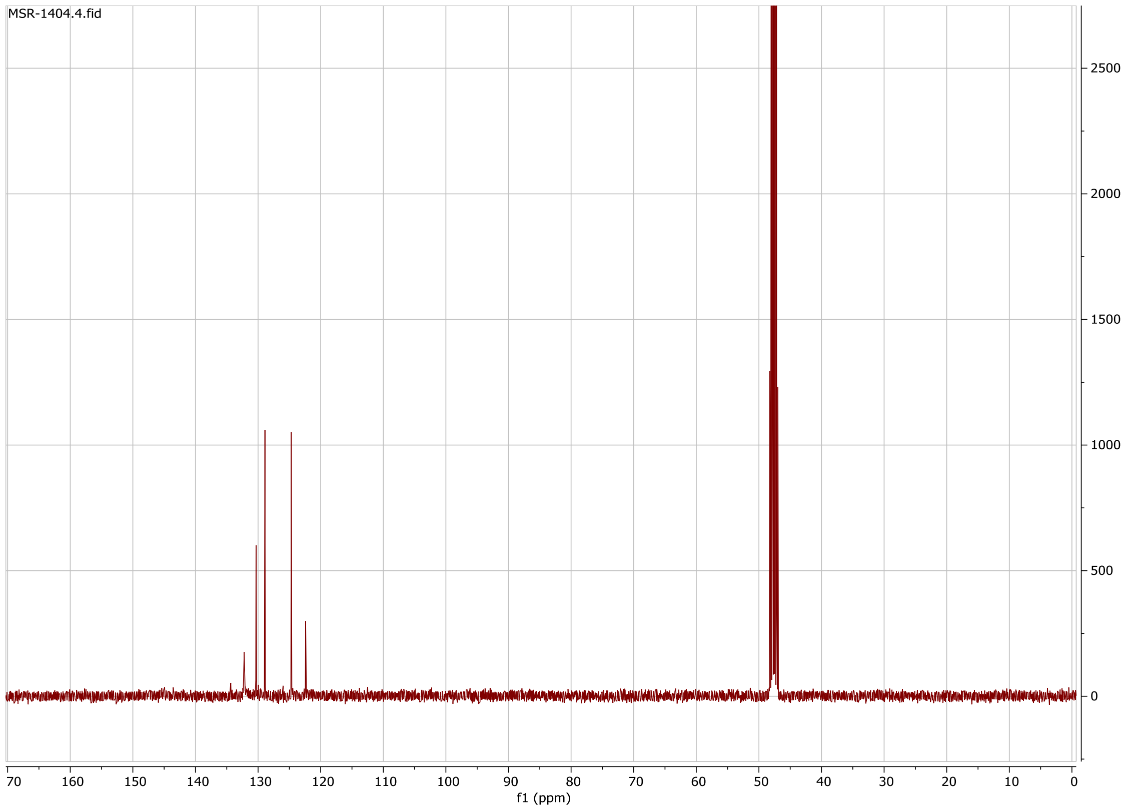

3-(3-Fluorophenyl)-1*H*-1,2,4-triazole (137):^{1}H NMR (400 MHz, $CDCl_3$)

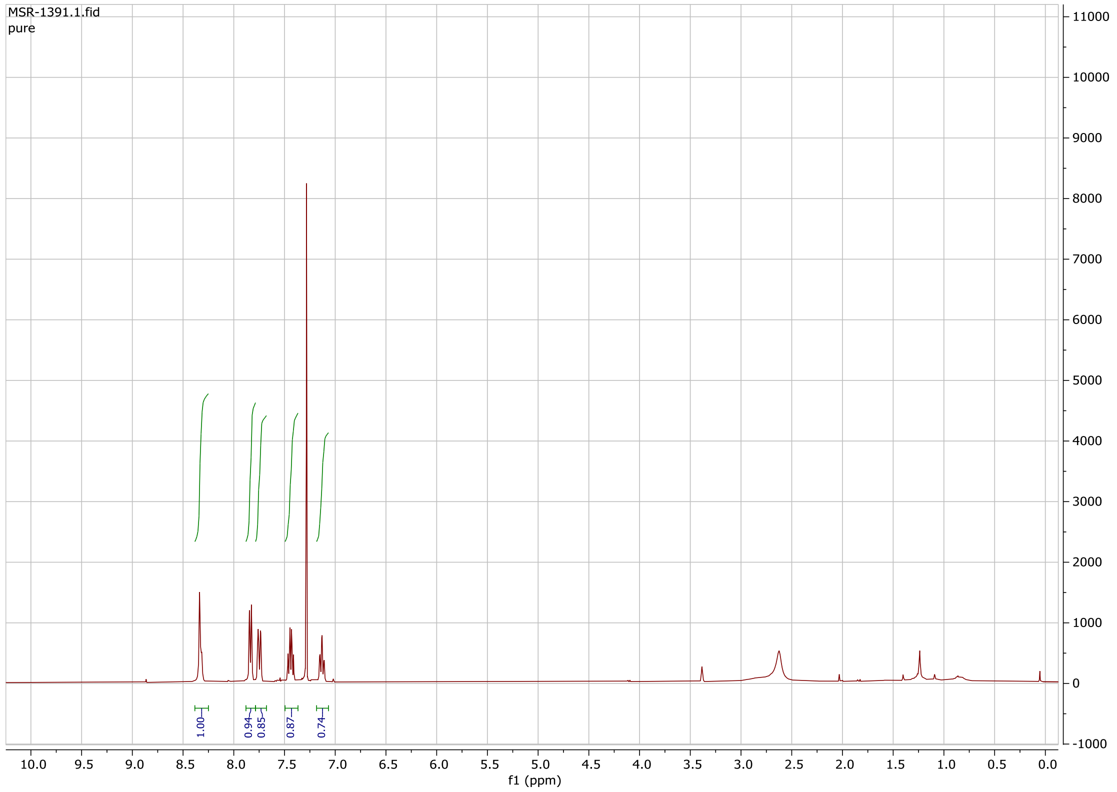

3-(3-Fluorophenyl)-1*H*-1,2,4-triazole (137):^{13}C NMR (101 MHz, CD_3OD)

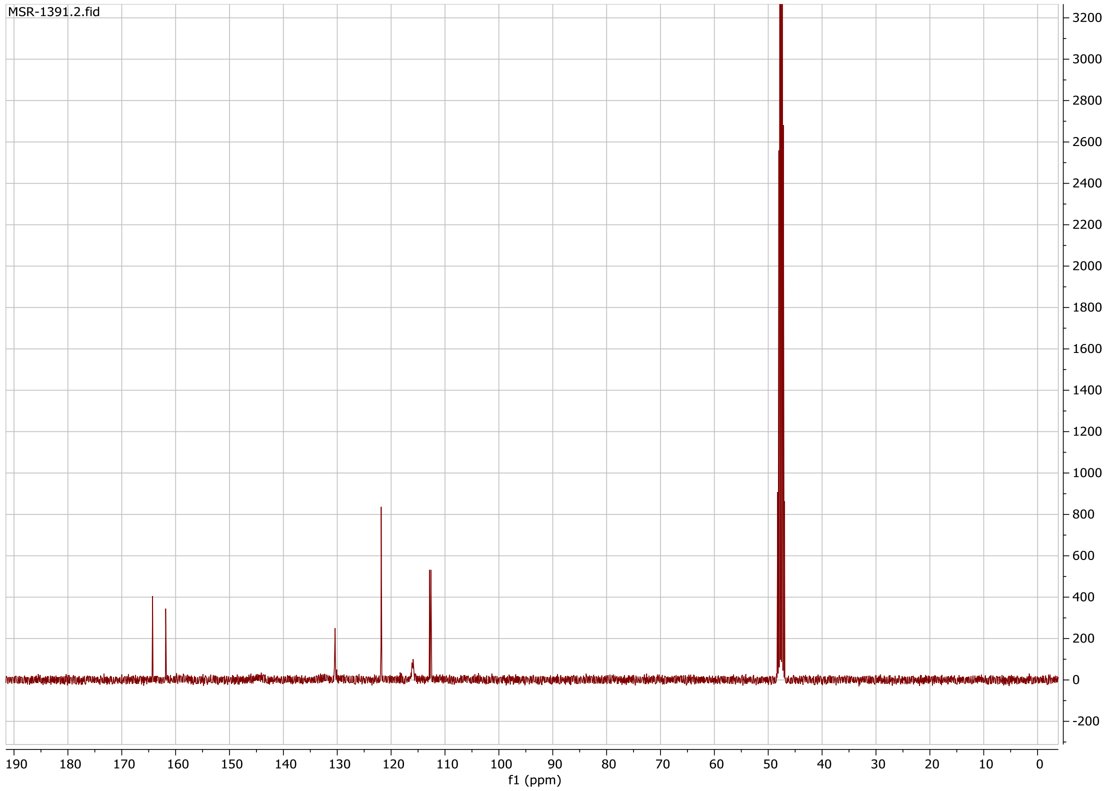

2-Fluoro-6-(1*H*-1,2,4-triazol-3-yl)aniline (140):^{1}H NMR (400 MHz, CD_3OD)

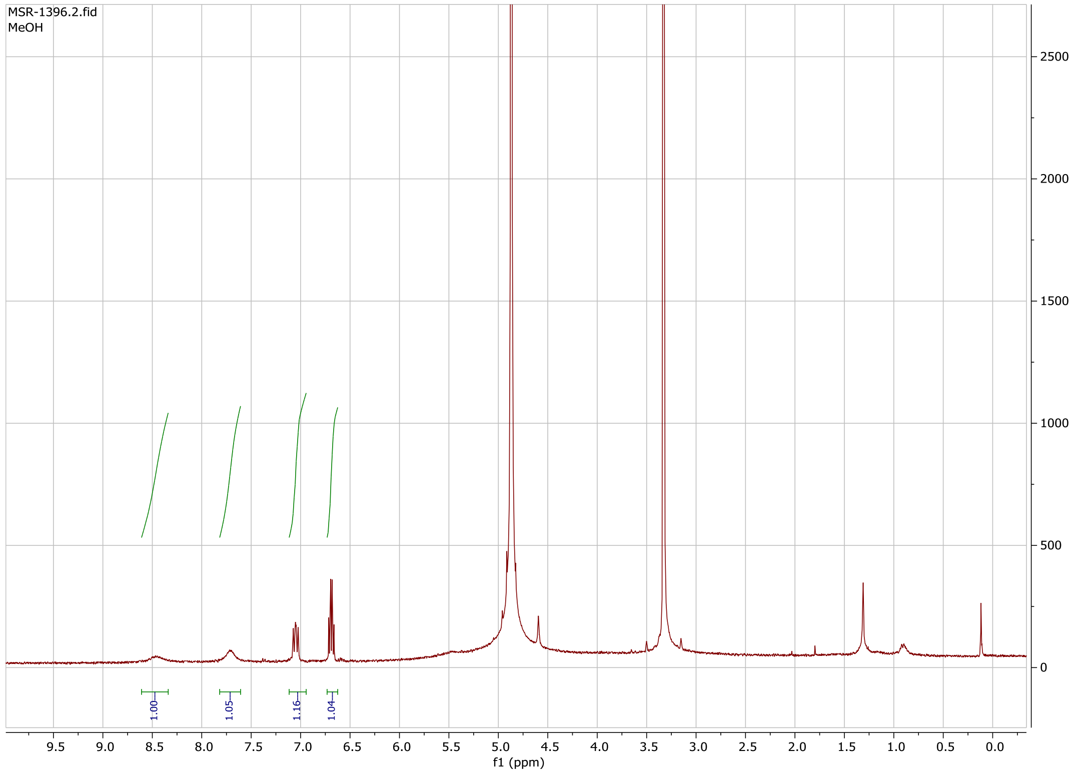

2-Fluoro-6-(1*H*-1,2,4-triazol-3-yl)aniline (140):^{13}C NMR (101 MHz, CD_3OD)

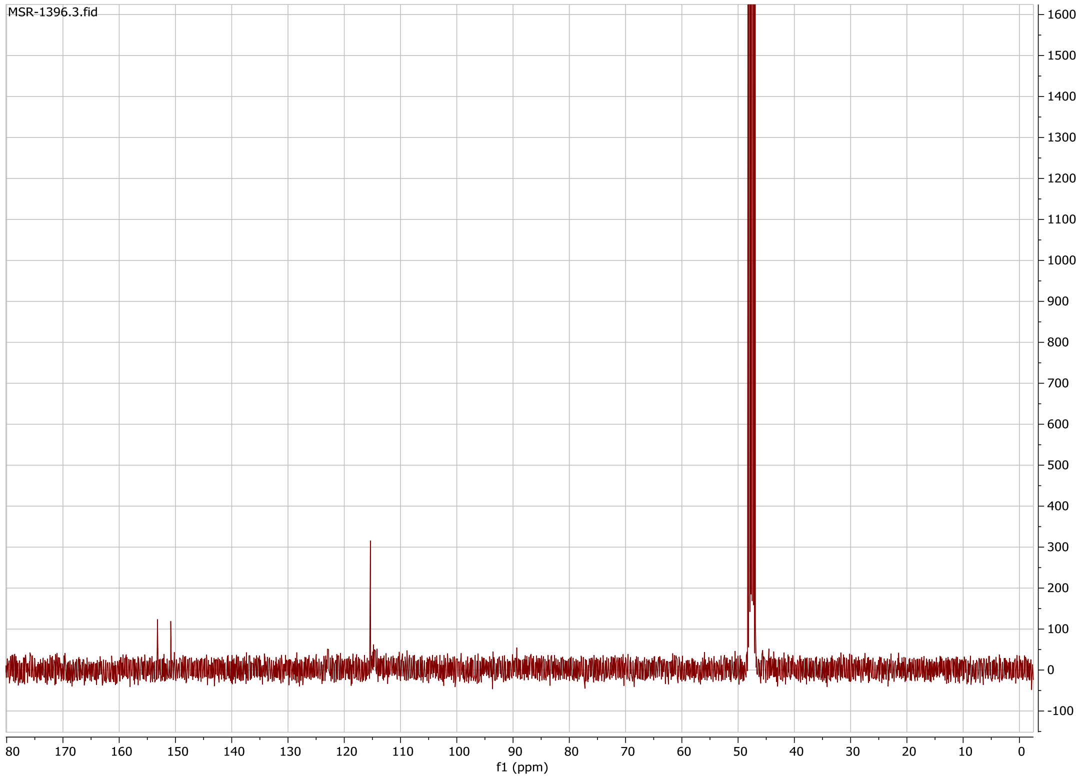

2-Chloro-6-(1*H*-1,2,4-triazol-3-yl)aniline (141):^{1}H NMR (400 MHz, CD_3OD)

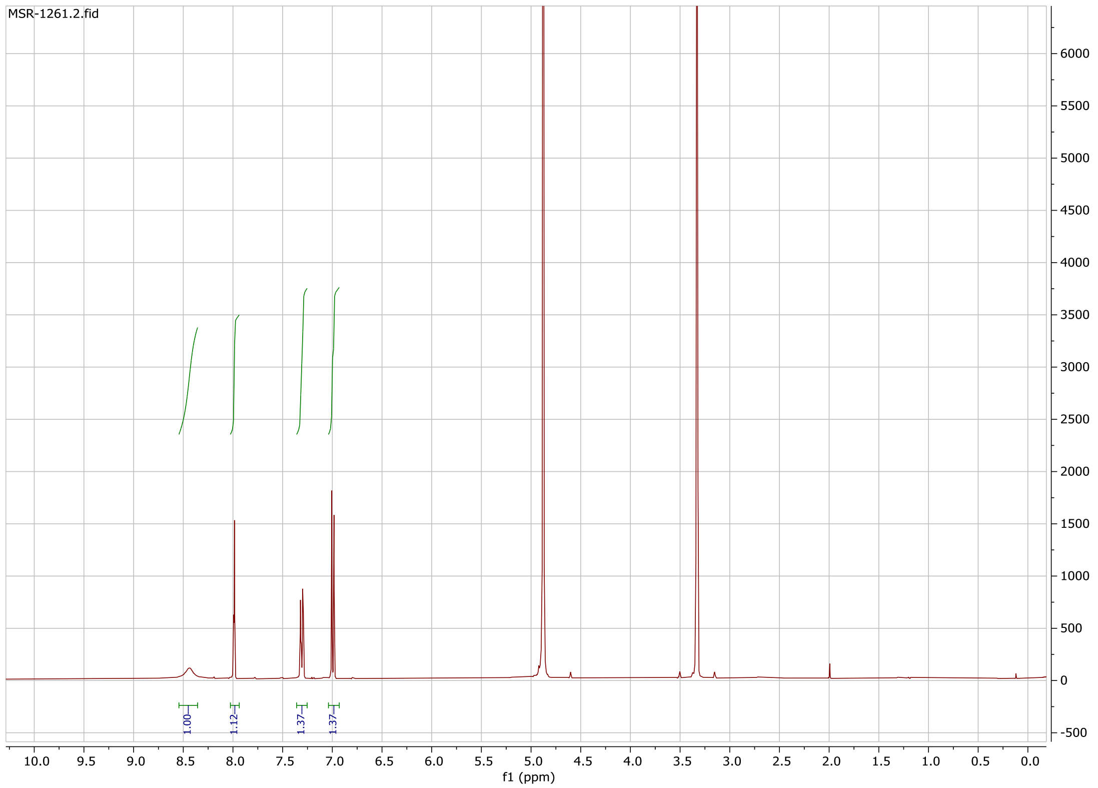

2-Chloro-6-(1*H*-1,2,4-triazol-3-yl)aniline (141):^{13}C NMR (101 MHz, CD_3OD)

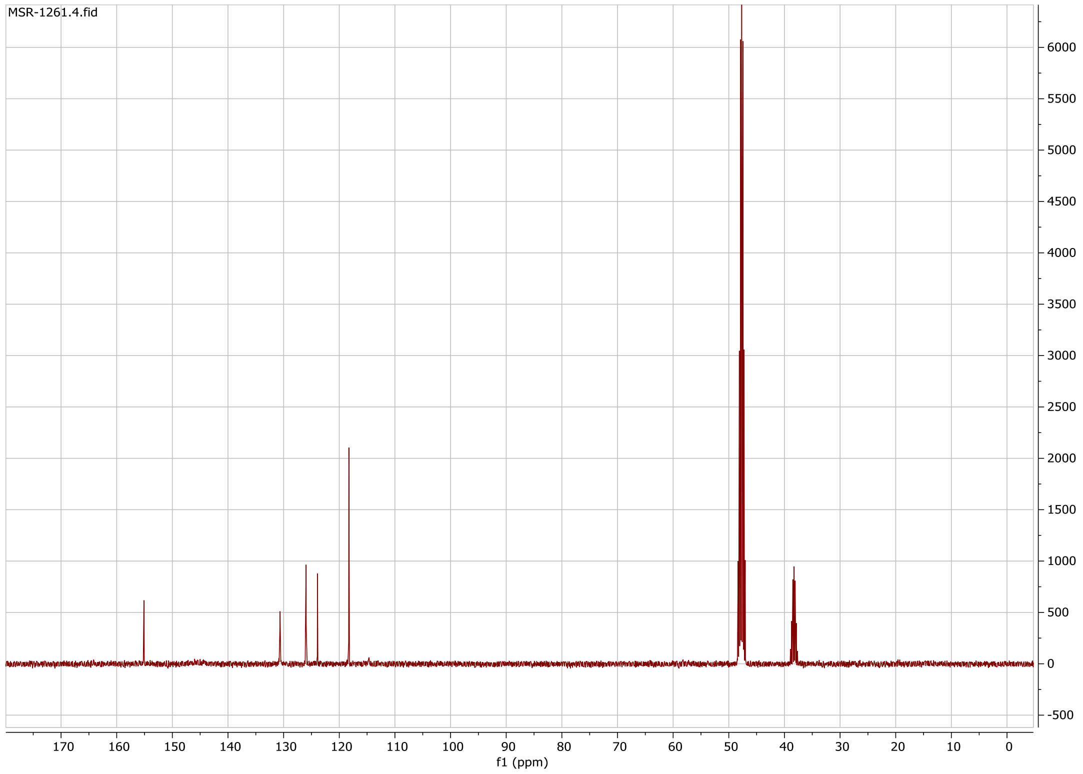

2-(1*H*-1,2,4-Triazol-3-yl)benzene-1,3-diamine (142): ^{1}H NMR (400 MHz, CD_3OD)

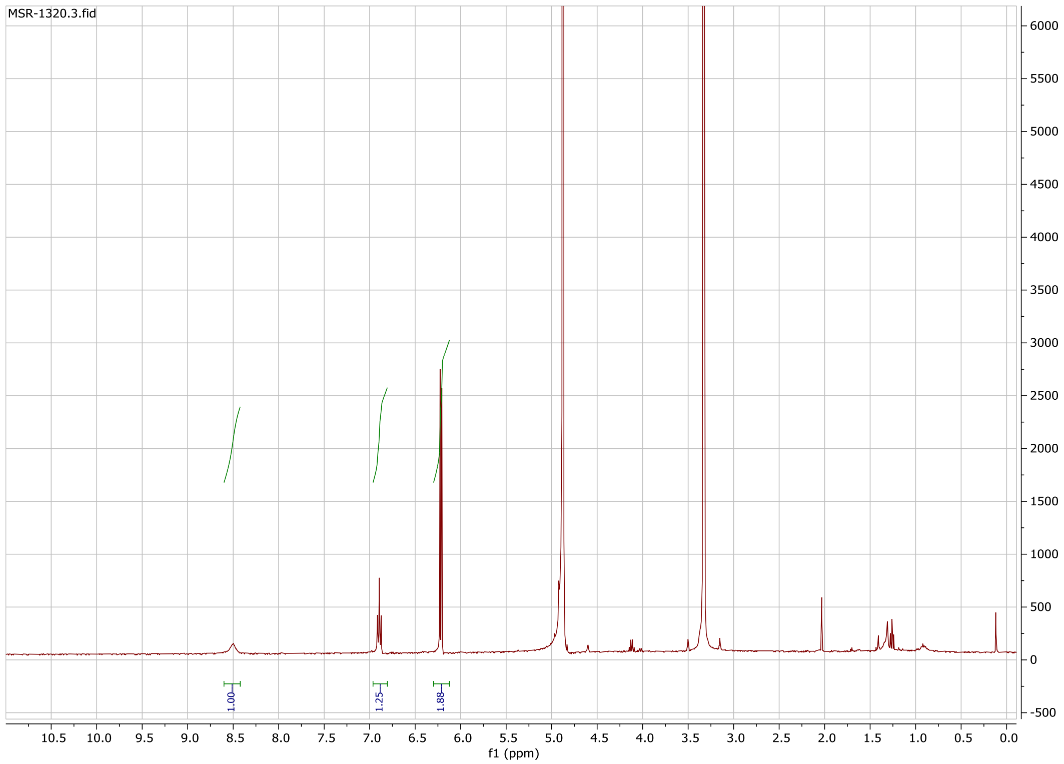

2-(1*H*-1,2,4-Triazol-3-yl)benzene-1,3-diamine (142): ^{13}C NMR (101 MHz, CD_3OD)

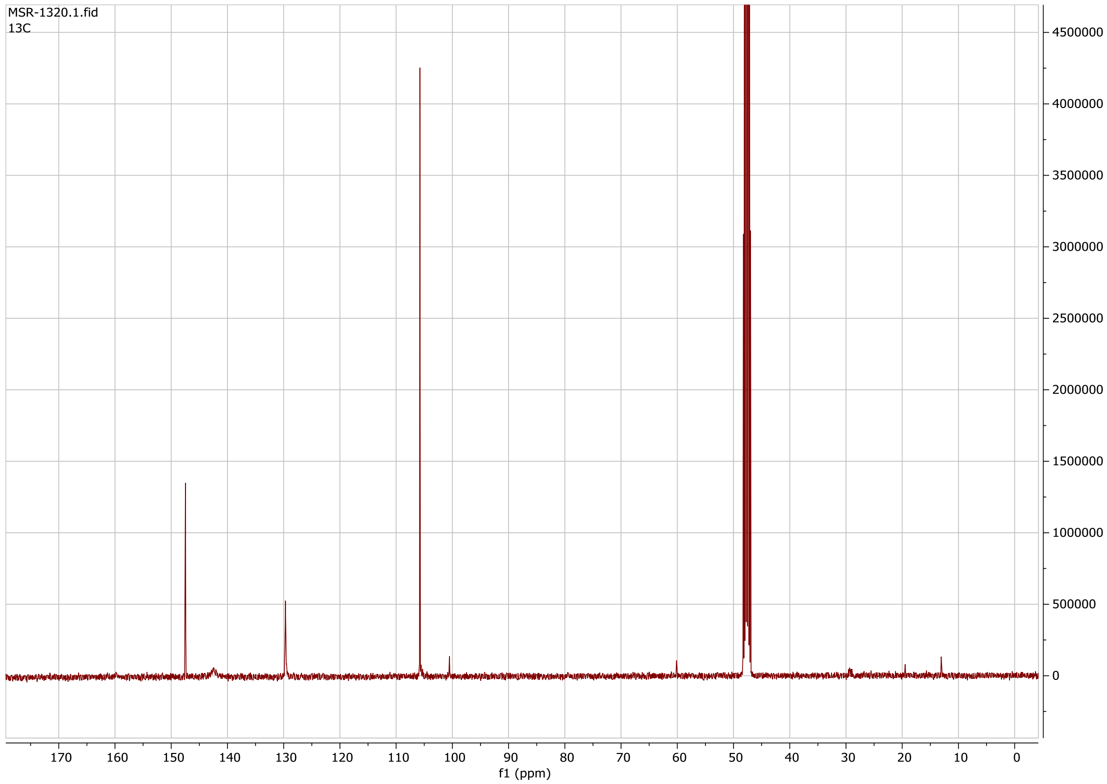

4-Fluoro-2-(1*H*-1,2,4-triazol-3-yl)aniline (143): ^{1}H NMR (400 MHz, CD_3OD)

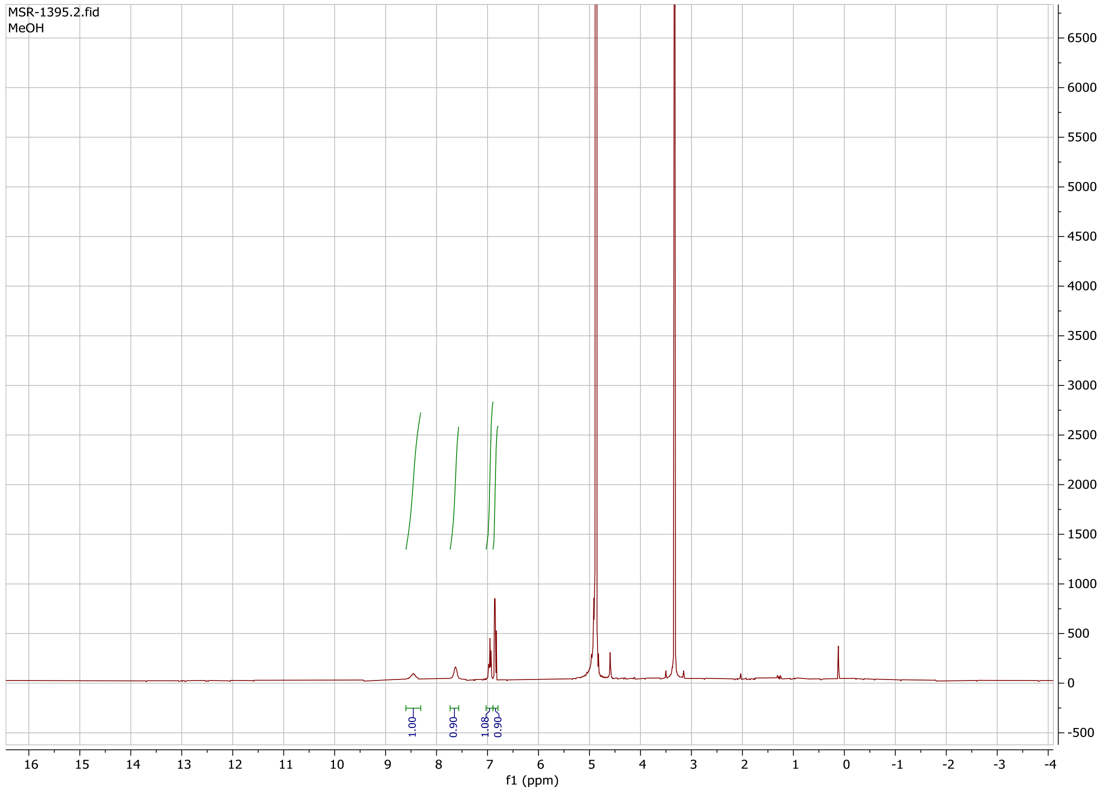

4-Fluoro-2-(1*H*-1,2,4-triazol-3-yl)aniline (143): ^{13}C NMR (101 MHz, CD_3OD)

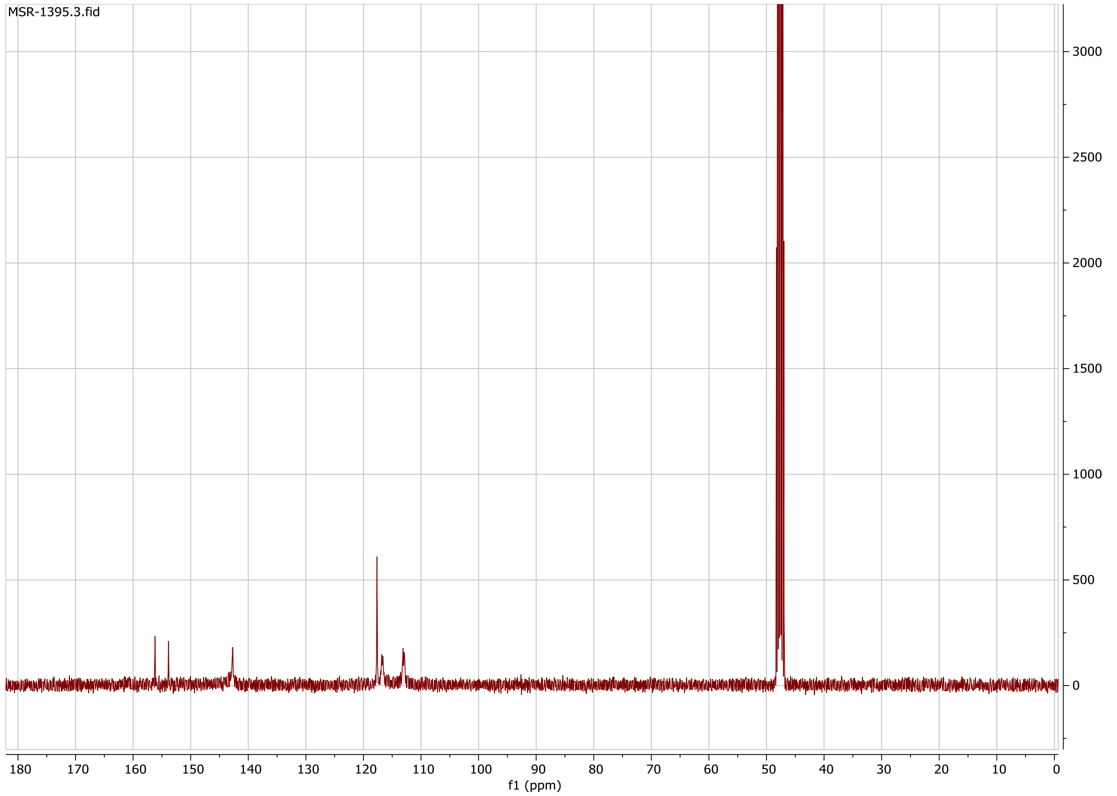

4-Bromo-5-fluoro-2-(1*H*-1,2,4-triazol-3-yl)aniline (144): ^{1}H NMR (400 MHz, $CDCl_3$)

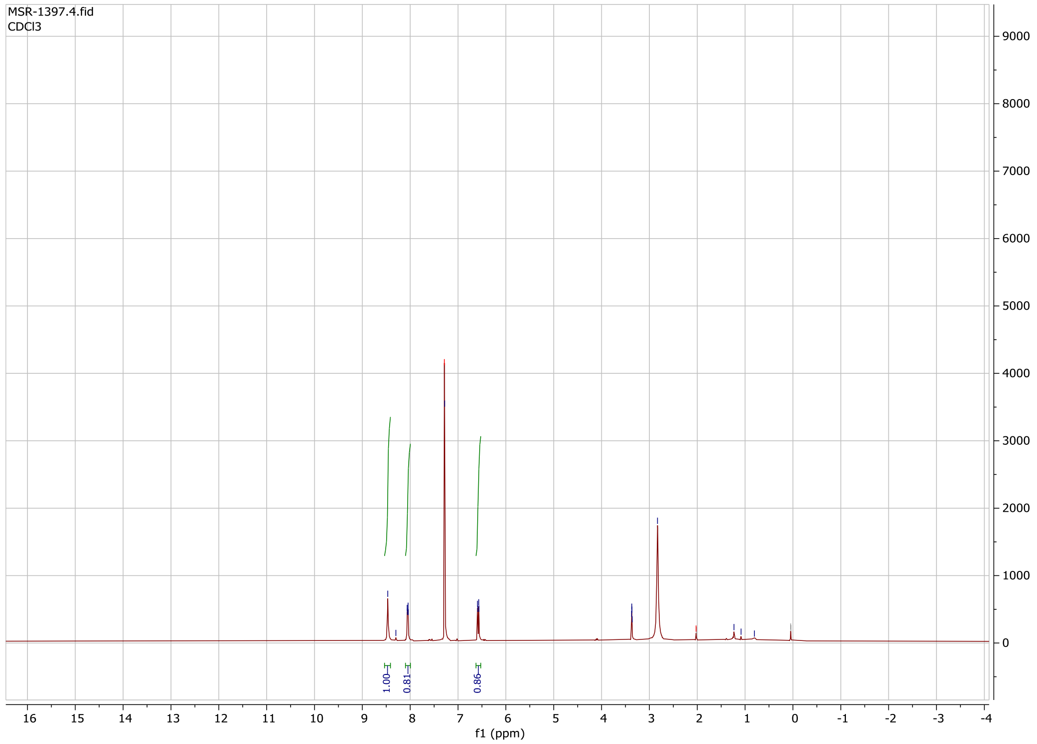

4-Bromo-5-fluoro-2-(1*H*-1,2,4-triazol-3-yl)aniline (144): ^{13}C NMR (101 MHz, CD_3OD)

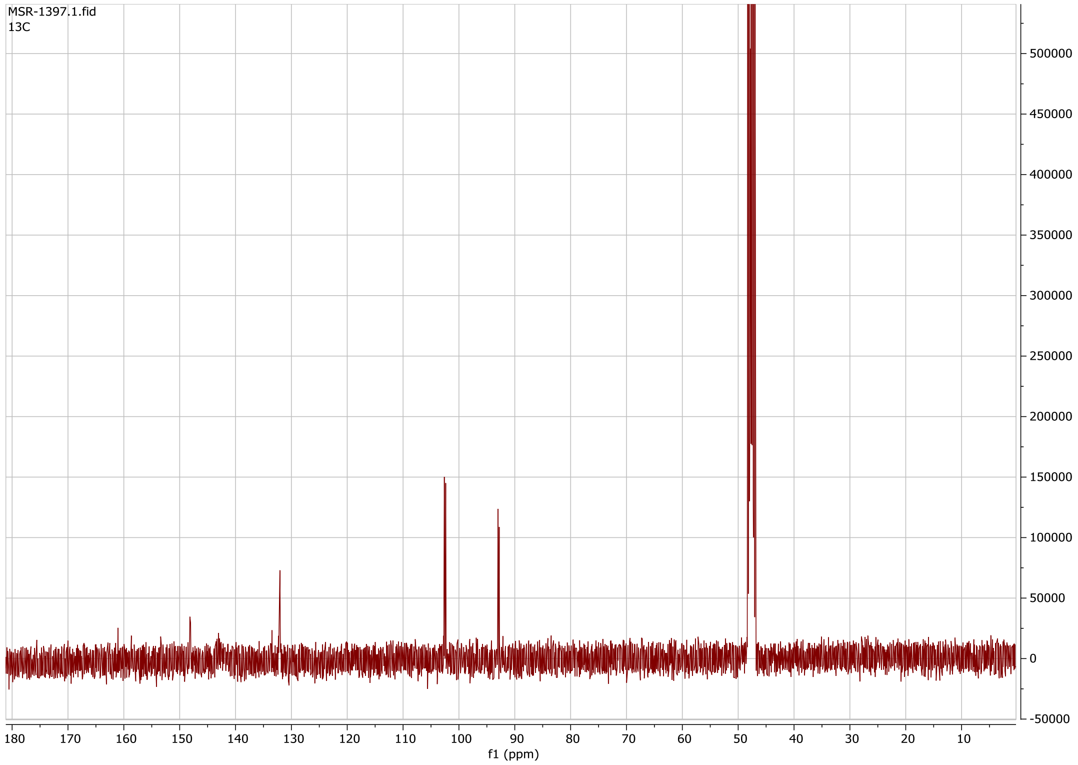

4-Chloro-*N*-methyl-2-(1*H*-1,2,4-triazol-3-yl)aniline (145): ^{1}H NMR (400 MHz, $CDCl_3$)

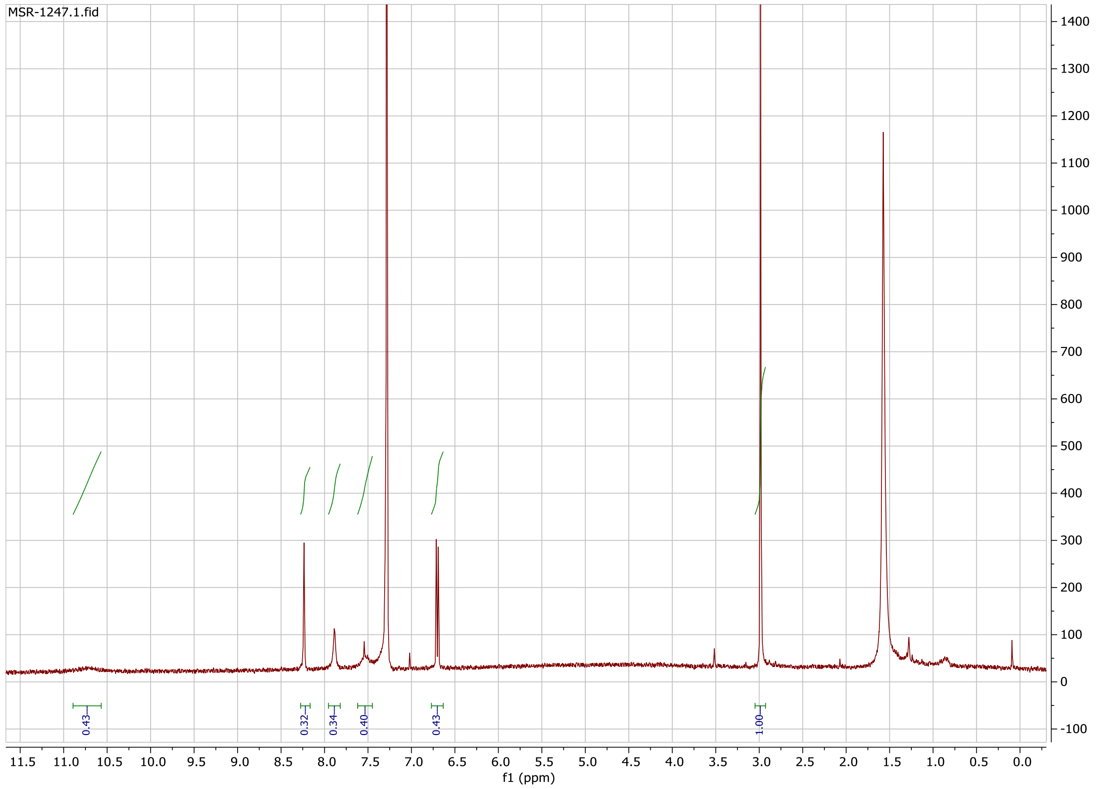

4-Chloro-*N*-methyl-2-(1*H*-1,2,4-triazol-3-yl)aniline (145): ^{13}C NMR (101 MHz, $CDCl_3$:CD_3OD)

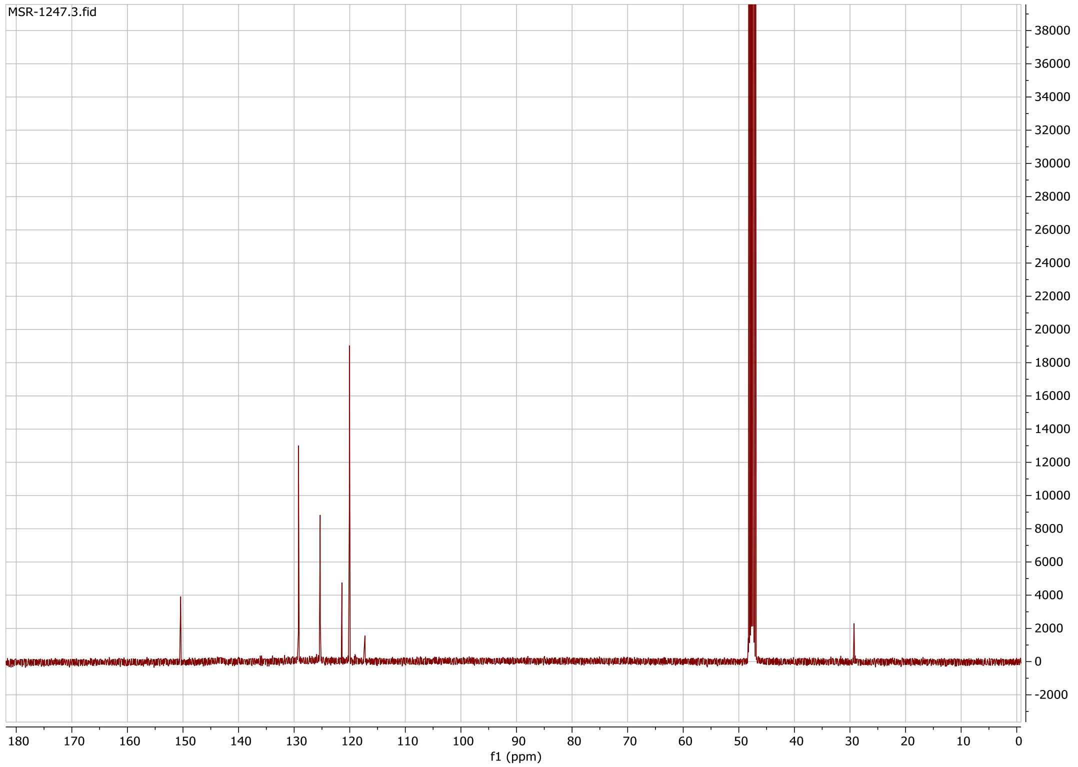

***N*-(4-Chloro-2-(1*H*-1,2,4-triazol-3-yl)phenyl)acetamide (146):** ^{1}H NMR (400 MHz, CD_3OD)

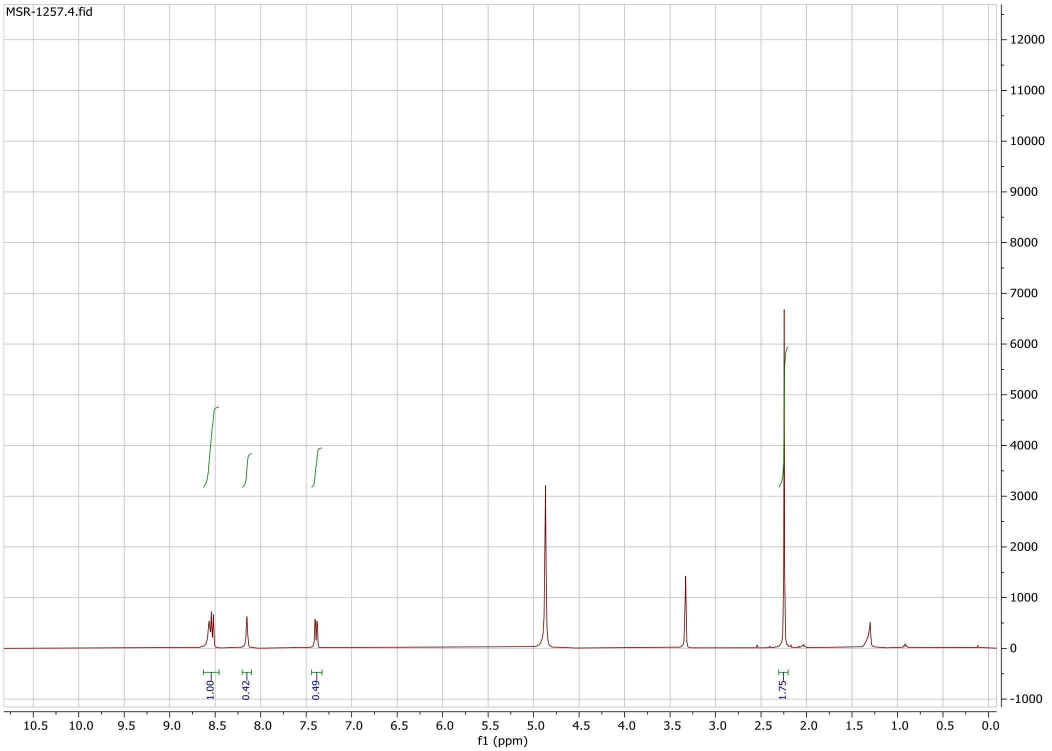

***N*-(4-Chloro-2-(1*H*-1,2,4-triazol-3-yl)phenyl)acetamide (146):** ^{13}C NMR (101 MHz, CD_3OD)

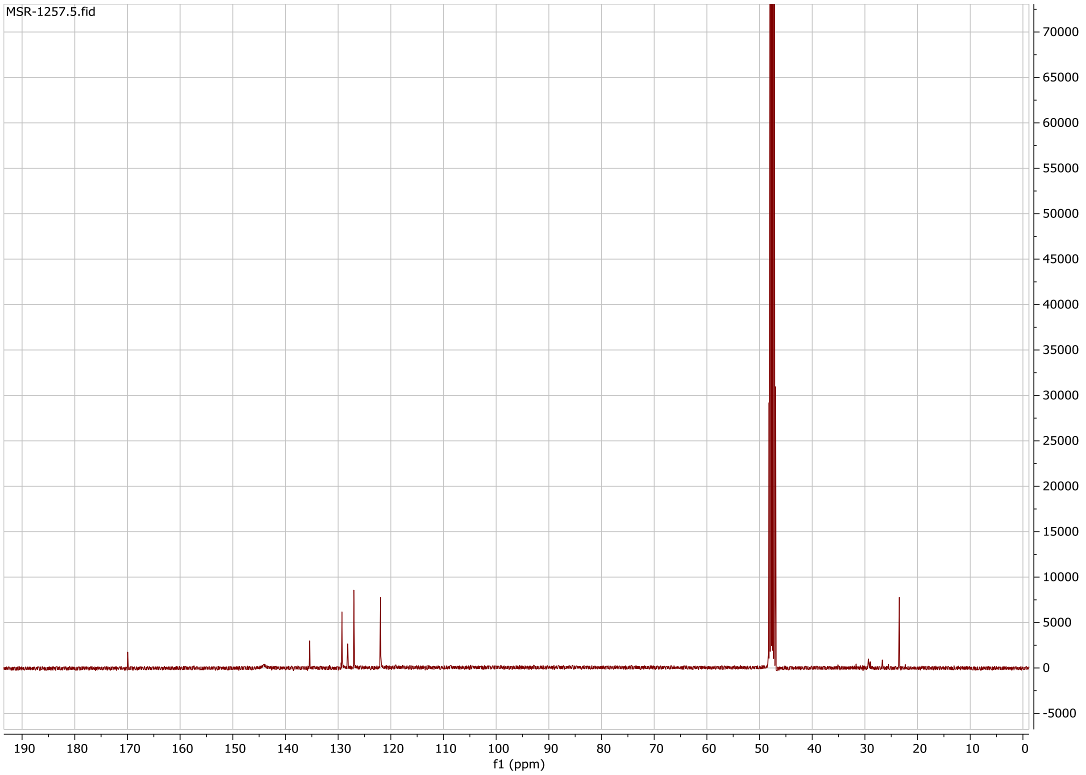

***N*-(4-Chloro-2-(1*H*-1,2,4-triazol-3-yl)phenyl)propionamide (147):**^{1}H NMR (400 MHz, DMSO+D_2O)

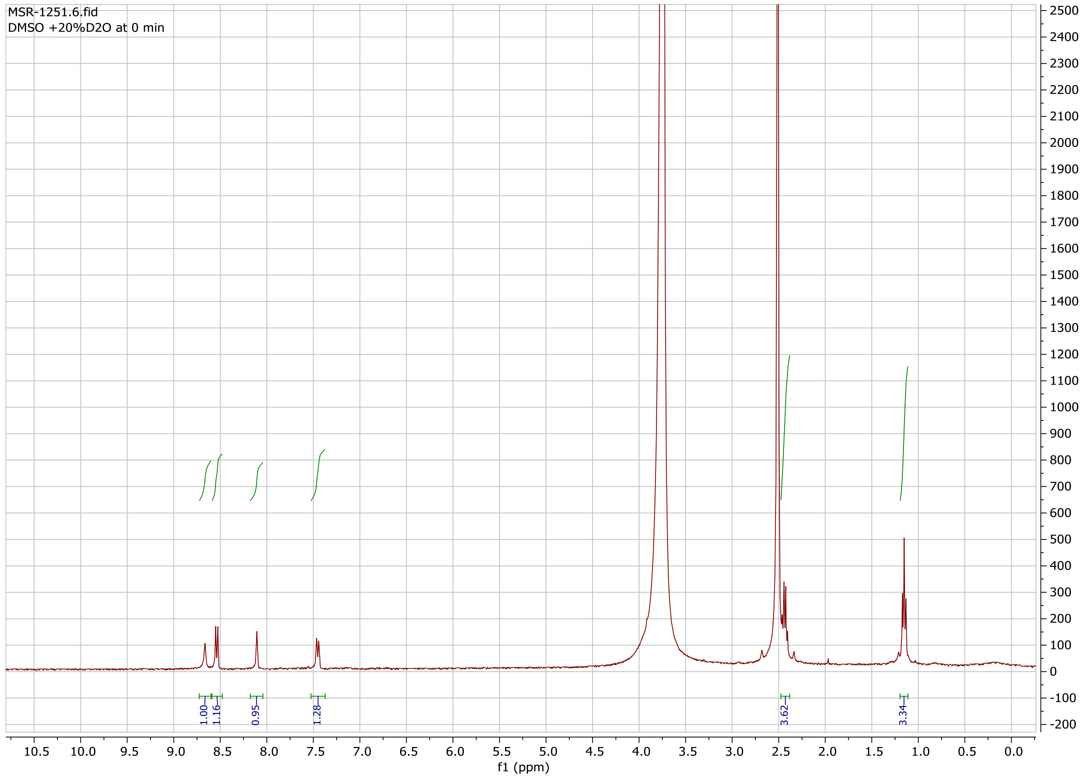

***N*-(4-Chloro-2-(1*H*-1,2,4-triazol-3-yl)phenyl)propionamide (147):**^{13}C NMR (101 MHz, CD_3OD)

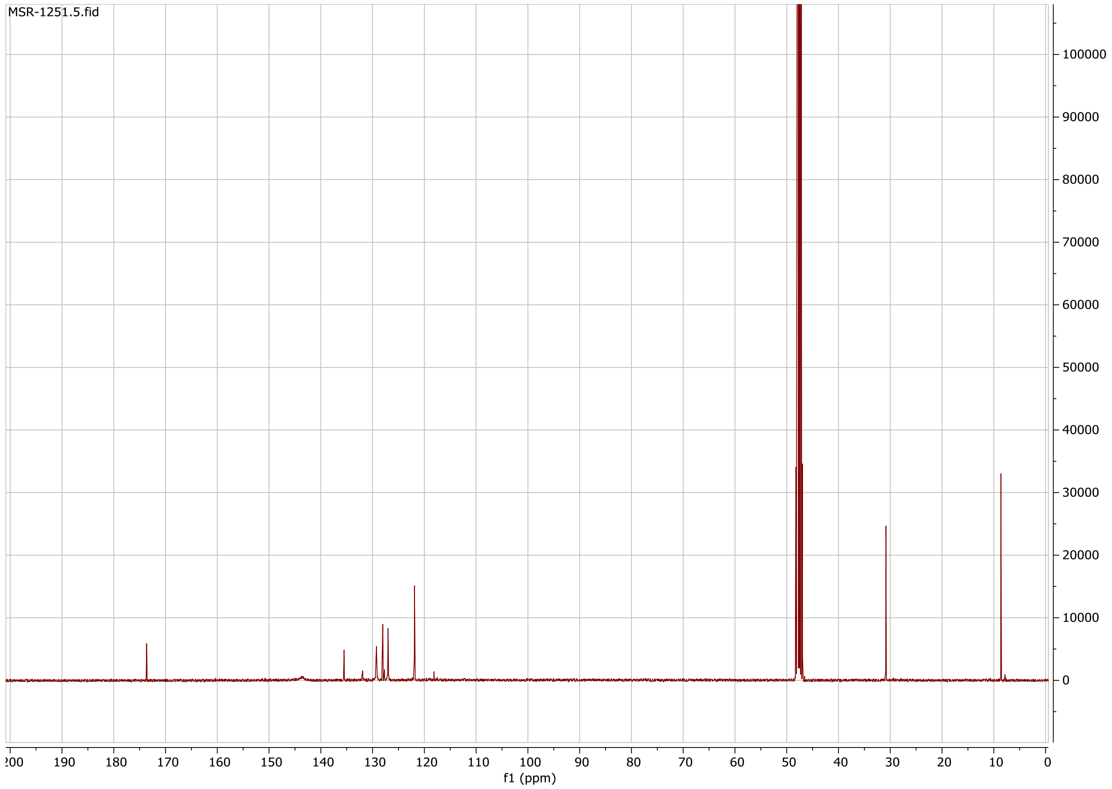

***N*-(4-Chloro-2-(1*H*-1,2,4-triazol-3-yl)phenyl)butyramide (148):** ^{1}H NMR (400 MHz, CD_3OD)

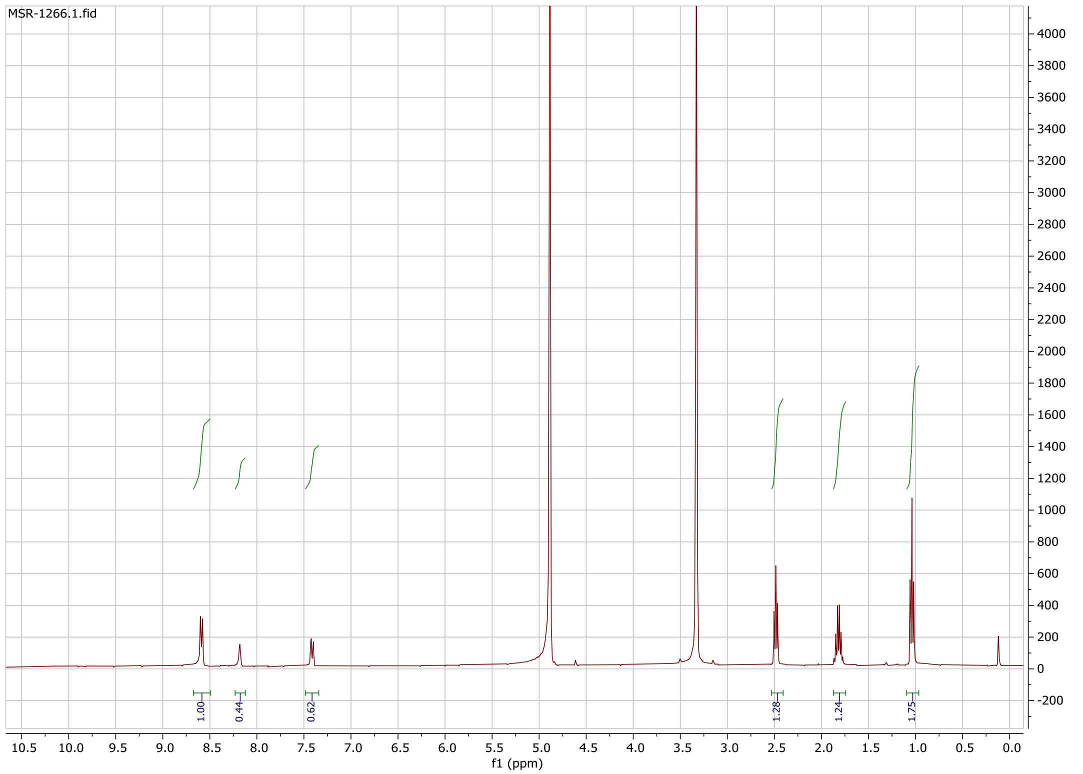

***N*-(4-Chloro-2-(1*H*-1,2,4-triazol-3-yl)phenyl)butyramide (148):** ^{13}C NMR (101 MHz, CD_3OD)

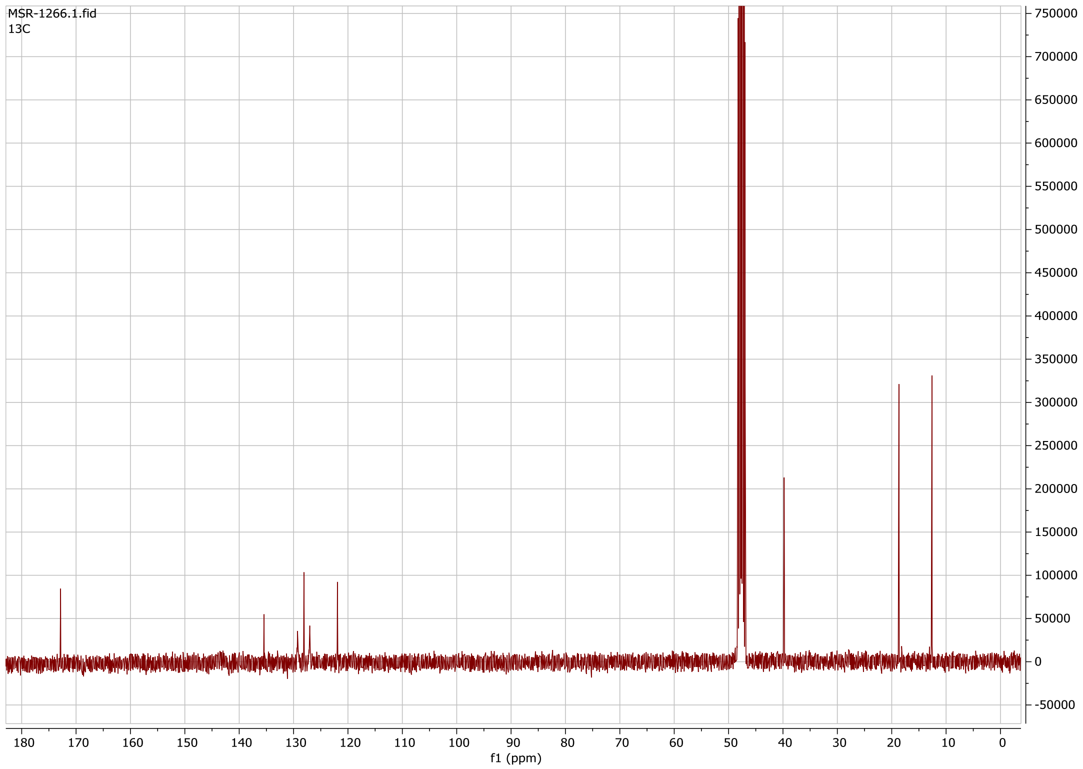

***N*-(4-Chloro-2-(1*H*-1,2,4-triazol-3-yl)phenyl)cyclopropanecarboxamide (149):**^{1}H NMR (400 MHz, DMSO+D_2O)

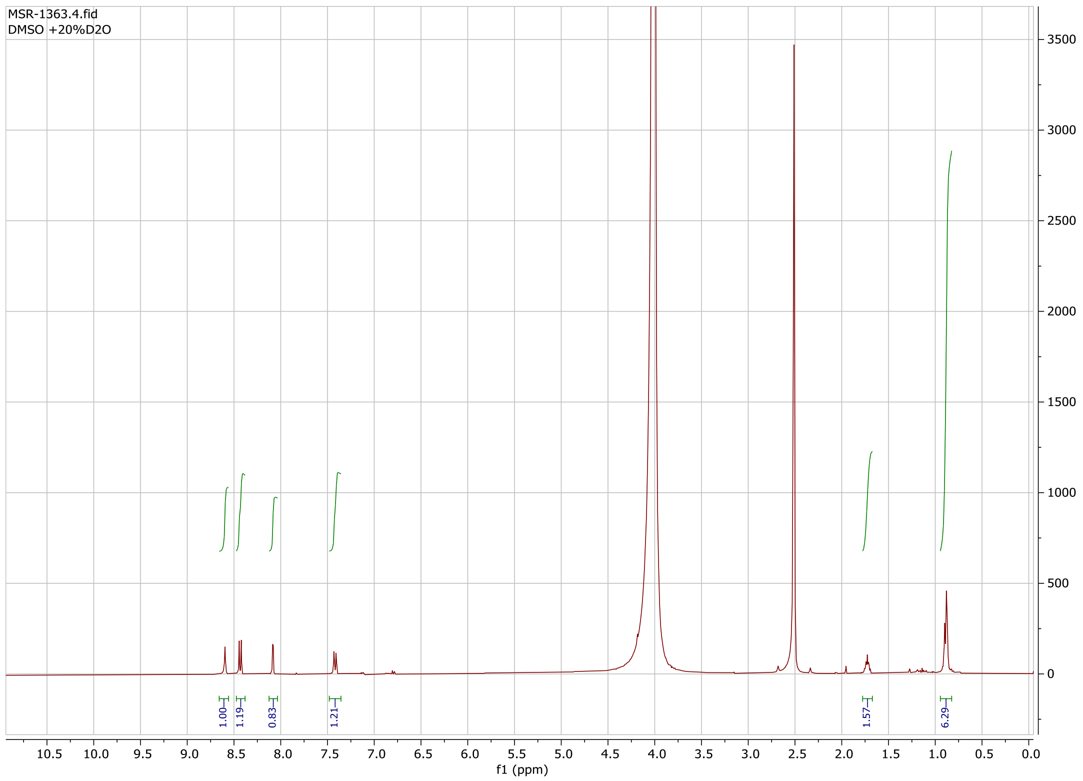

***N*-(4-Chloro-2-(1*H*-1,2,4-triazol-3-yl)phenyl)cyclopropanecarboxamide (149):**^{13}C NMR (101 MHz, CD_3OD)

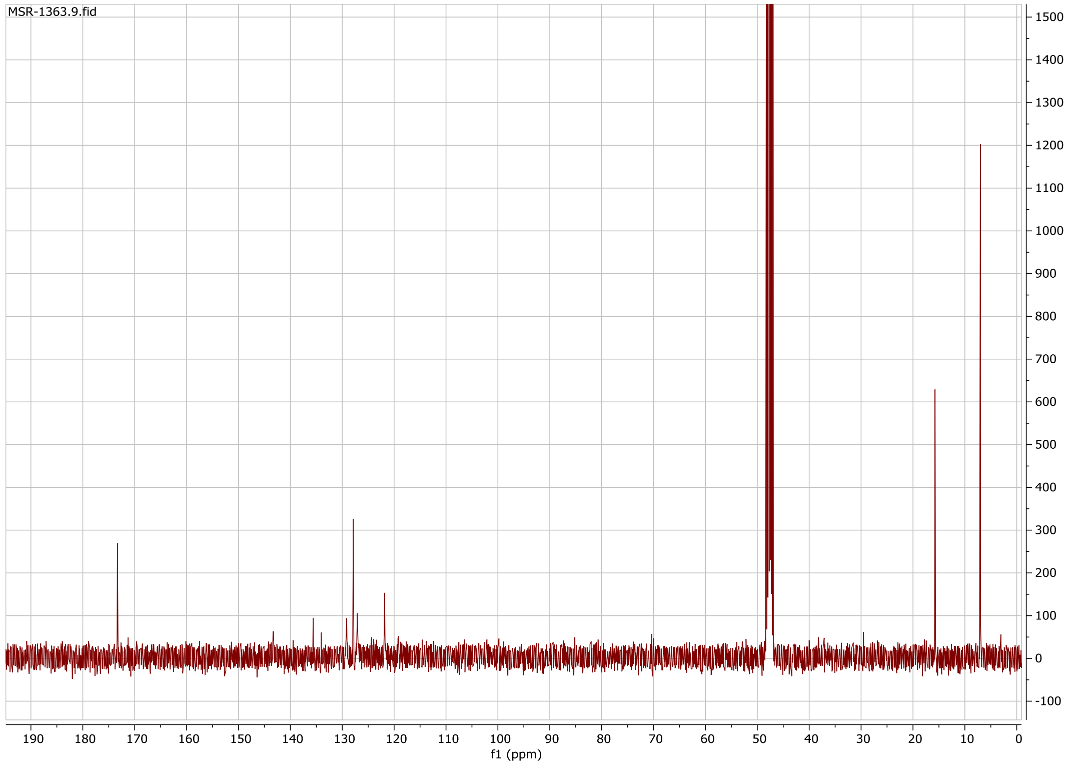

***N*-(4-Chloro-2-(1*H*-1,2,4-triazol-3-yl)phenyl)cyclobutanecarboxamide (150):**^{1}H NMR (400 MHz, DMSO)

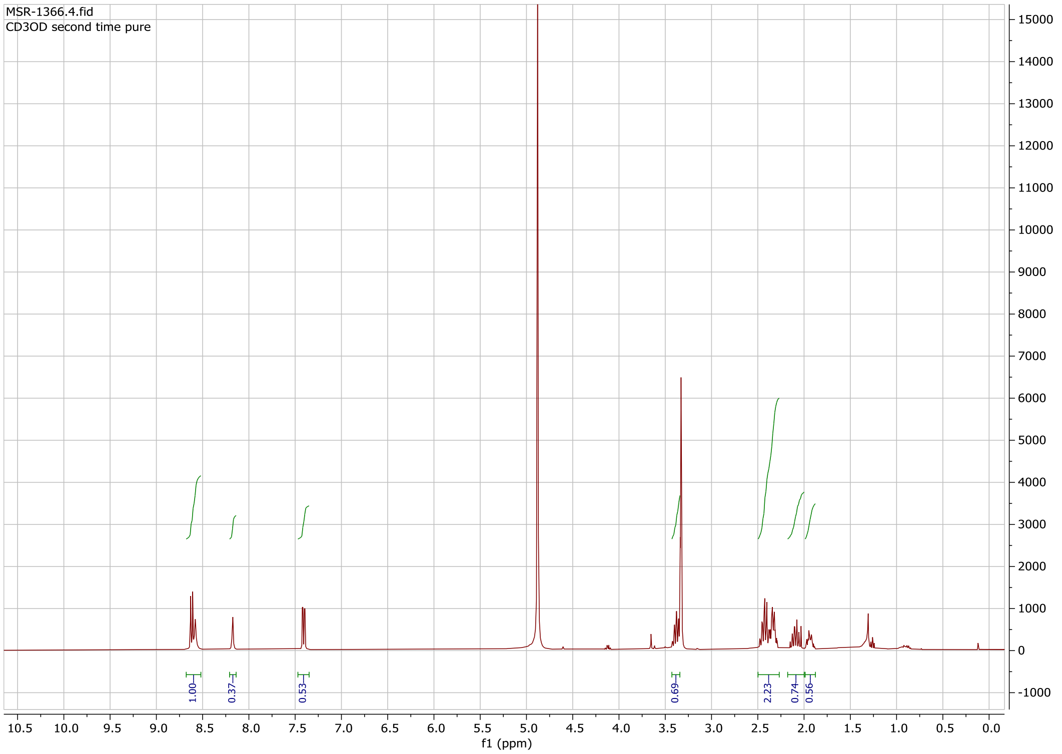

***N*-(4-Chloro-2-(1*H*-1,2,4-triazol-3-yl)phenyl)cyclobutanecarboxamide (150):**^{13}C NMR (101 MHz, CD_3OD)

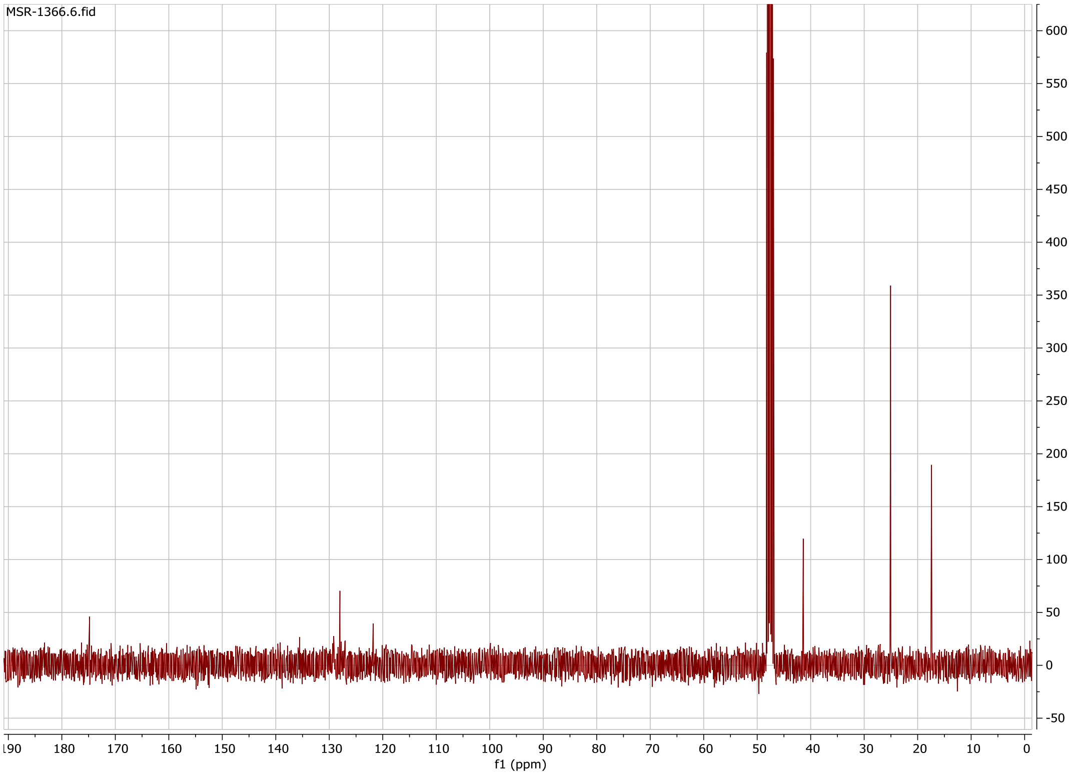

***N*-(4-Chloro-2-(1*H*-1,2,4-triazol-3-yl)phenyl)cyclopentanecarboxamide (151):** ^{1}H NMR (400 MHz, DMSO)

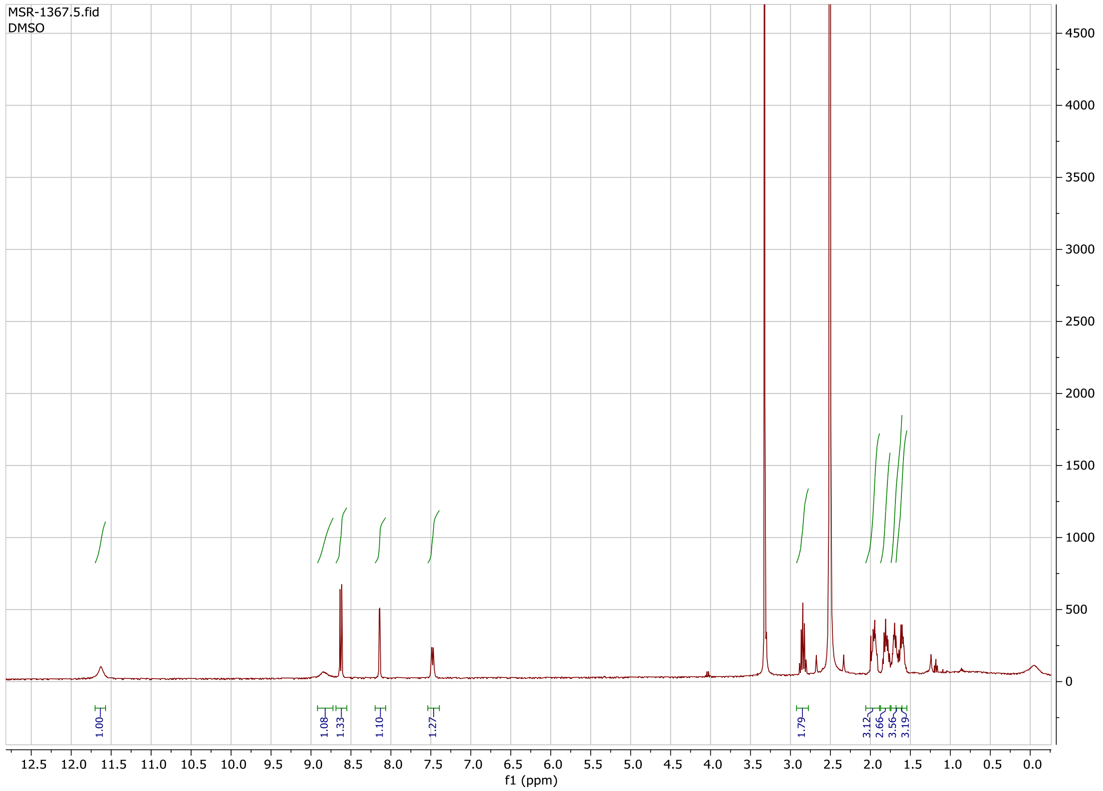

***N*-(4-Chloro-2-(1*H*-1,2,4-triazol-3-yl)phenyl)cyclopentanecarboxamide (151):** ^{13}C NMR (101 MHz, CD_3OD)

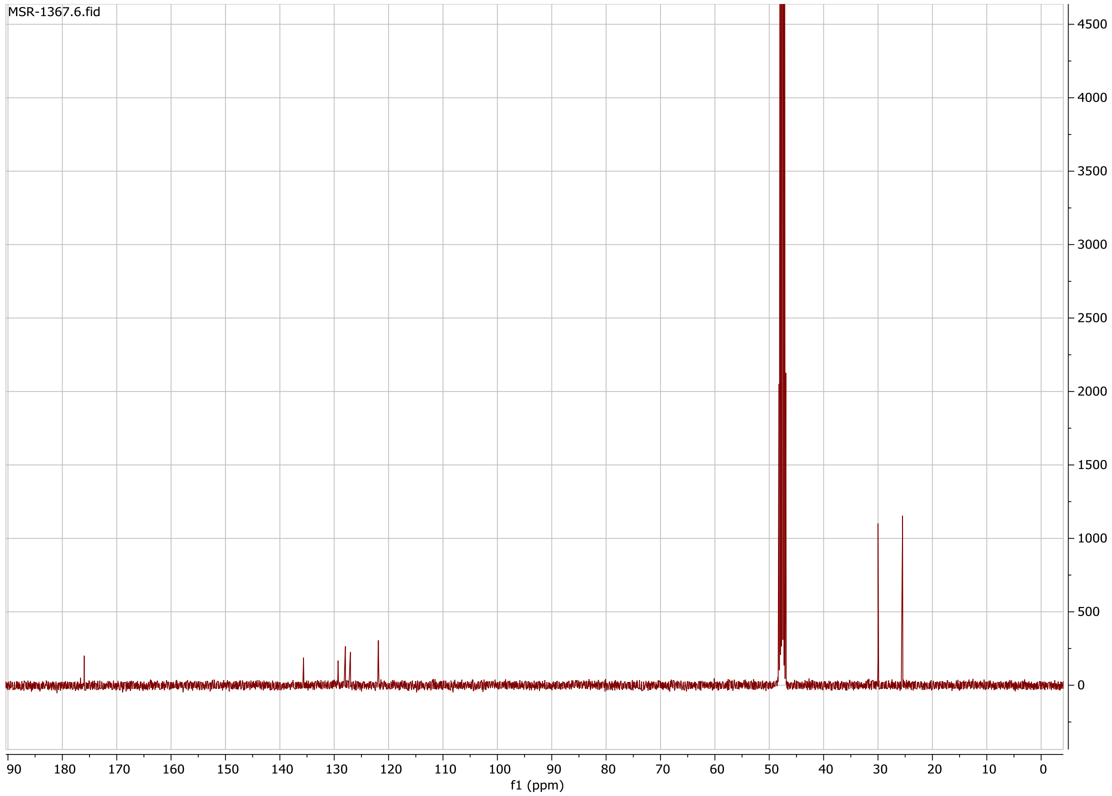

***N*-(2-(1*H*-1,2,4-Triazol-3-yl)phenyl)benzamide (152):**^{1}H NMR (400 MHz, CD_3OD)

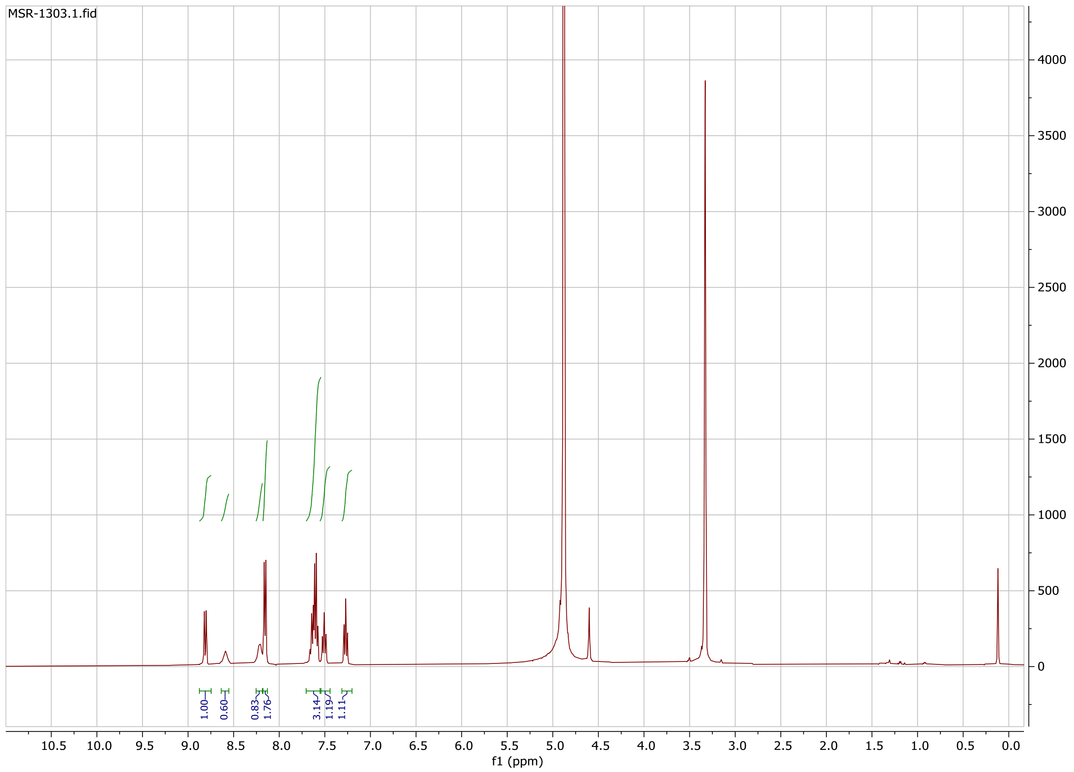

***N*-(2-(1*H*-1,2,4-Triazol-3-yl)phenyl)benzamide (152):**^{13}C NMR (101 MHz, CD_3OD)

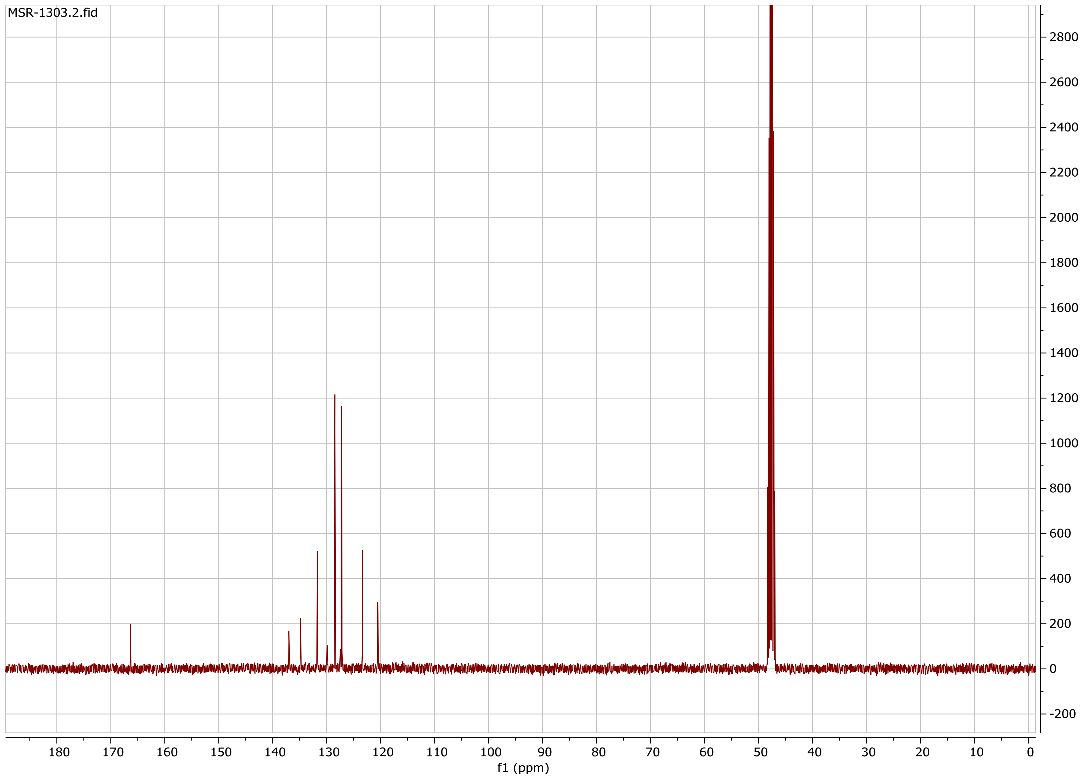

***N*-(4-Chloro-2-(1*H*-1,2,4-triazol-3-yl)phenyl)benzamide (153):** ^{1}H NMR (400 MHz, CD_3OD)

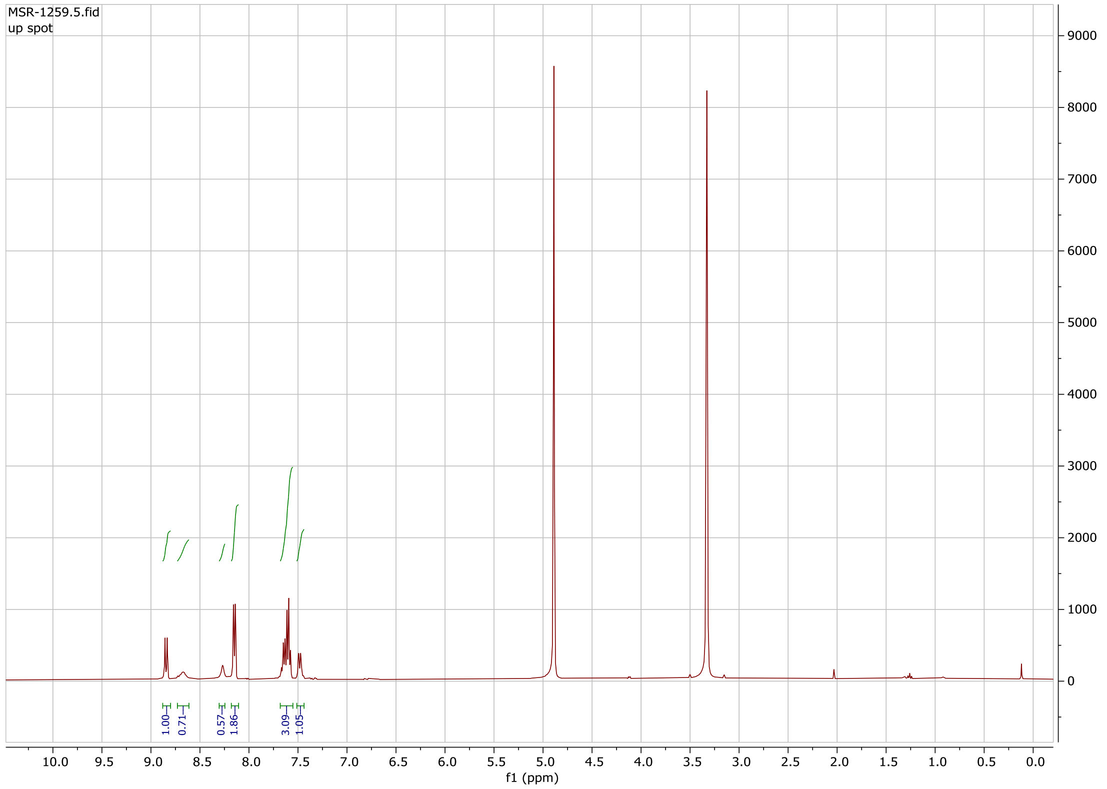

***N*-(4-Chloro-2-(1*H*-1,2,4-triazol-3-yl)phenyl)benzamide (153):** ^{13}C NMR (101 MHz, CD_3OD)

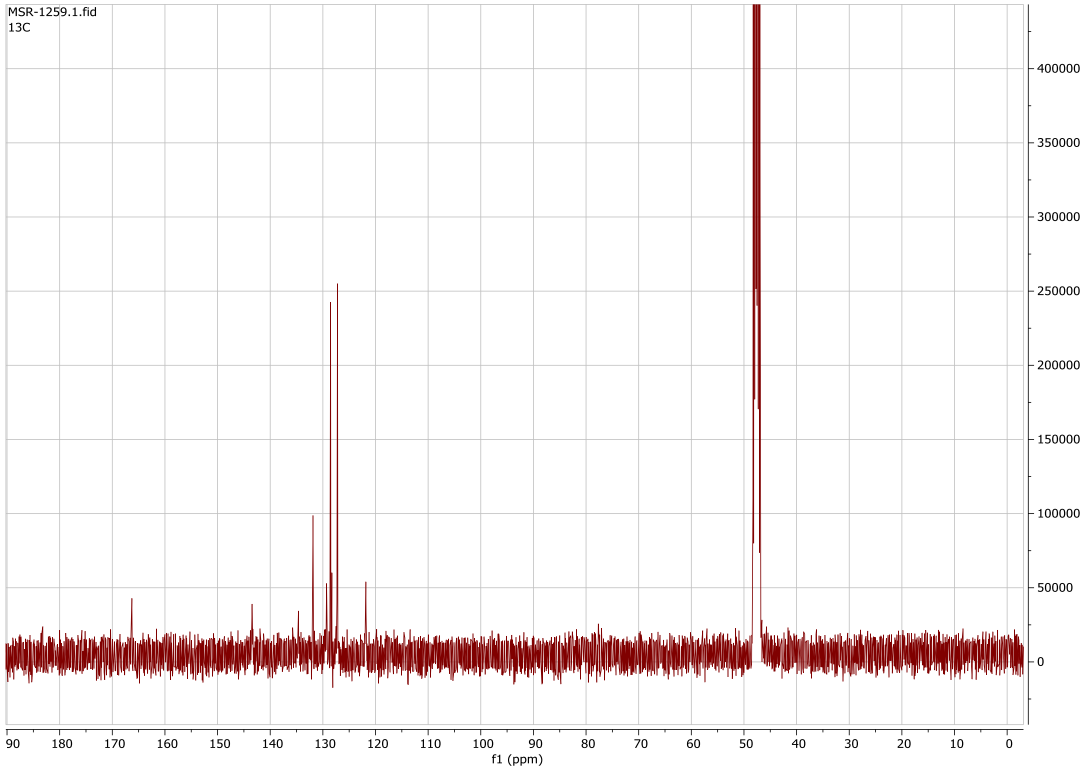

***N*-(2-Chloro-6-(1*H*-1,2,4-triazol-3-yl)phenyl)benzamide (154):** ^{1}H NMR (400 MHz, $CDCl_3$)

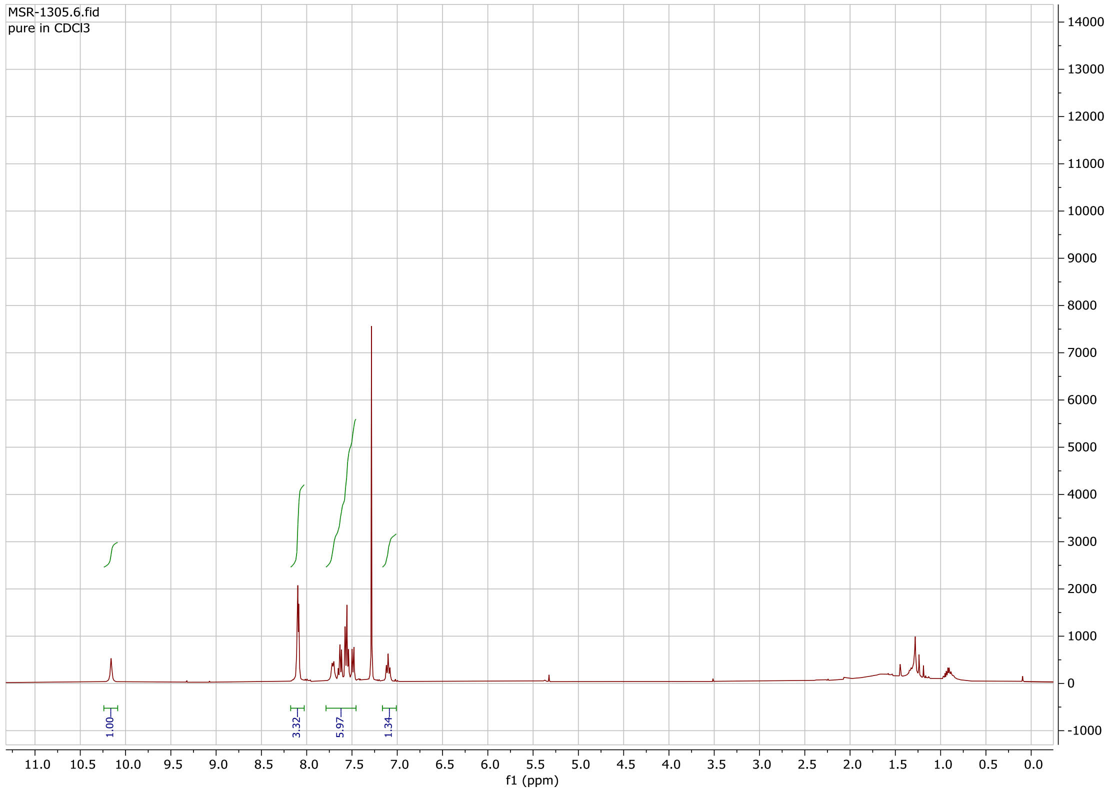

***N*-(2-Chloro-6-(1*H*-1,2,4-triazol-3-yl)phenyl)benzamide (154):** ^{13}C NMR (101 MHz, $CDCl_3$)

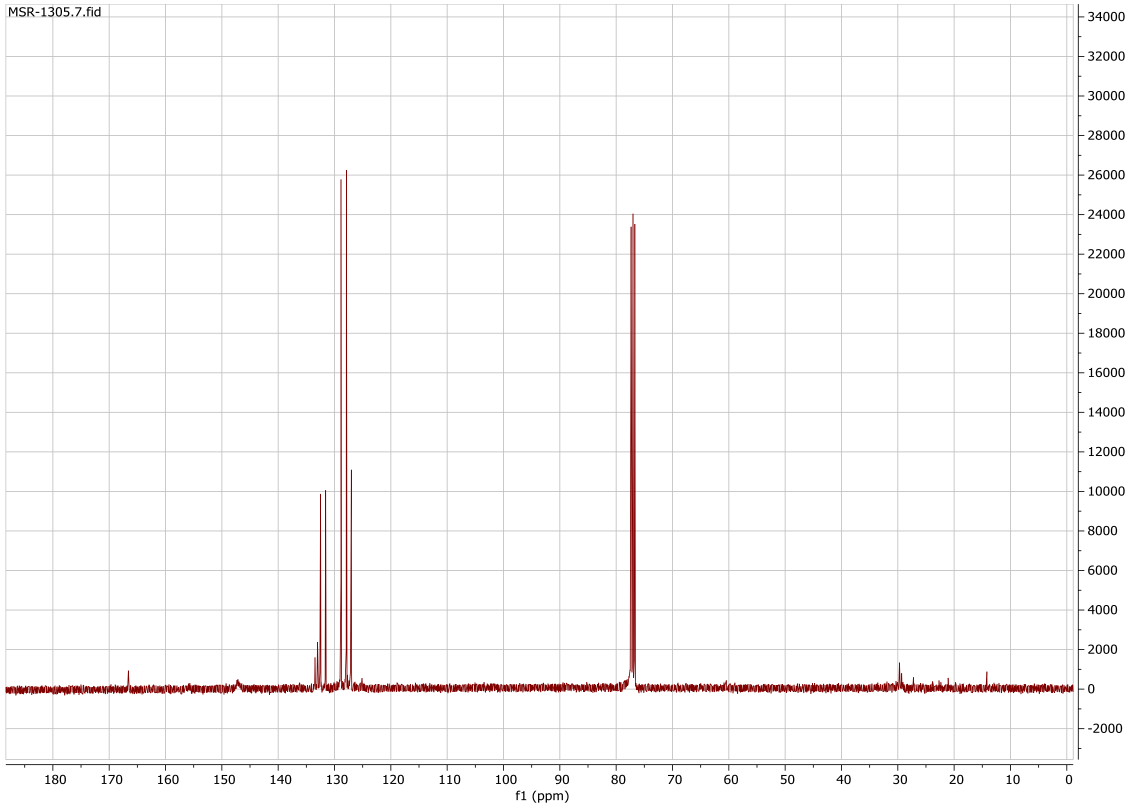

4-Bromo-3-methyl-2-(1*H*-1,2,4-triazol-3-yl)aniline (155): ^{1}H NMR (400 MHz, CD_3OD)

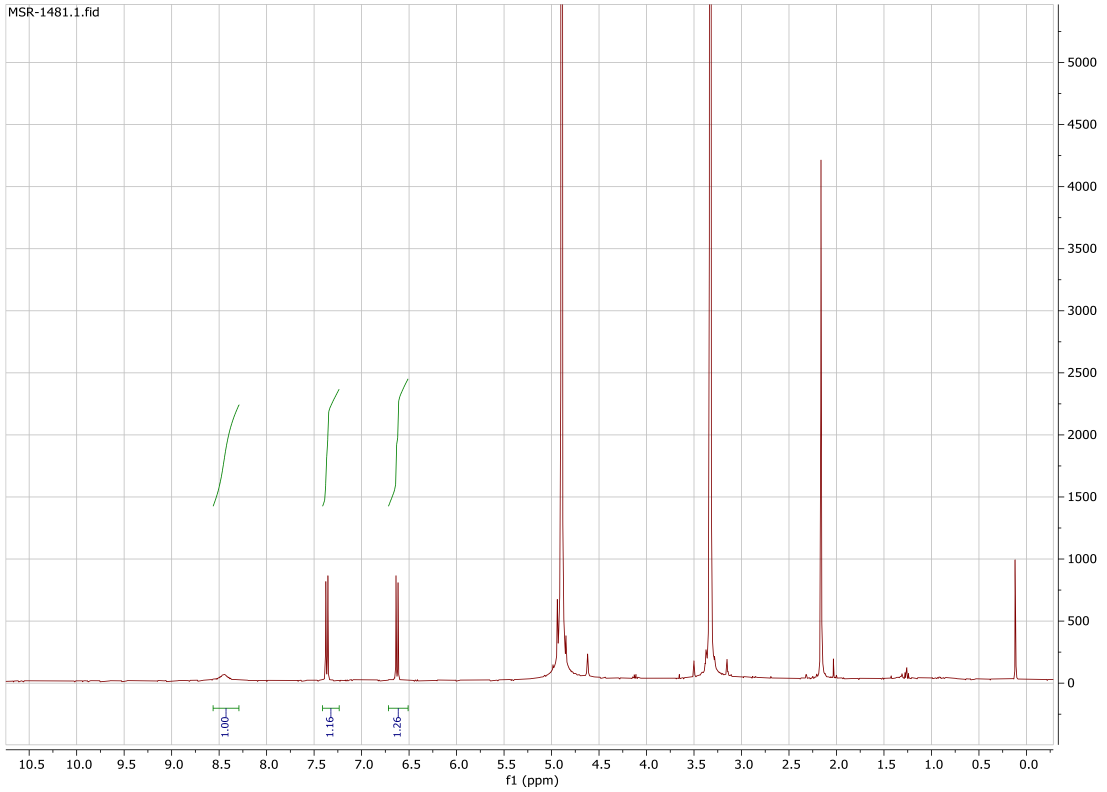

4-Bromo-3-methyl-2-(1*H*-1,2,4-triazol-3-yl)aniline (155): ^{13}C NMR (101 MHz, CD_3OD)

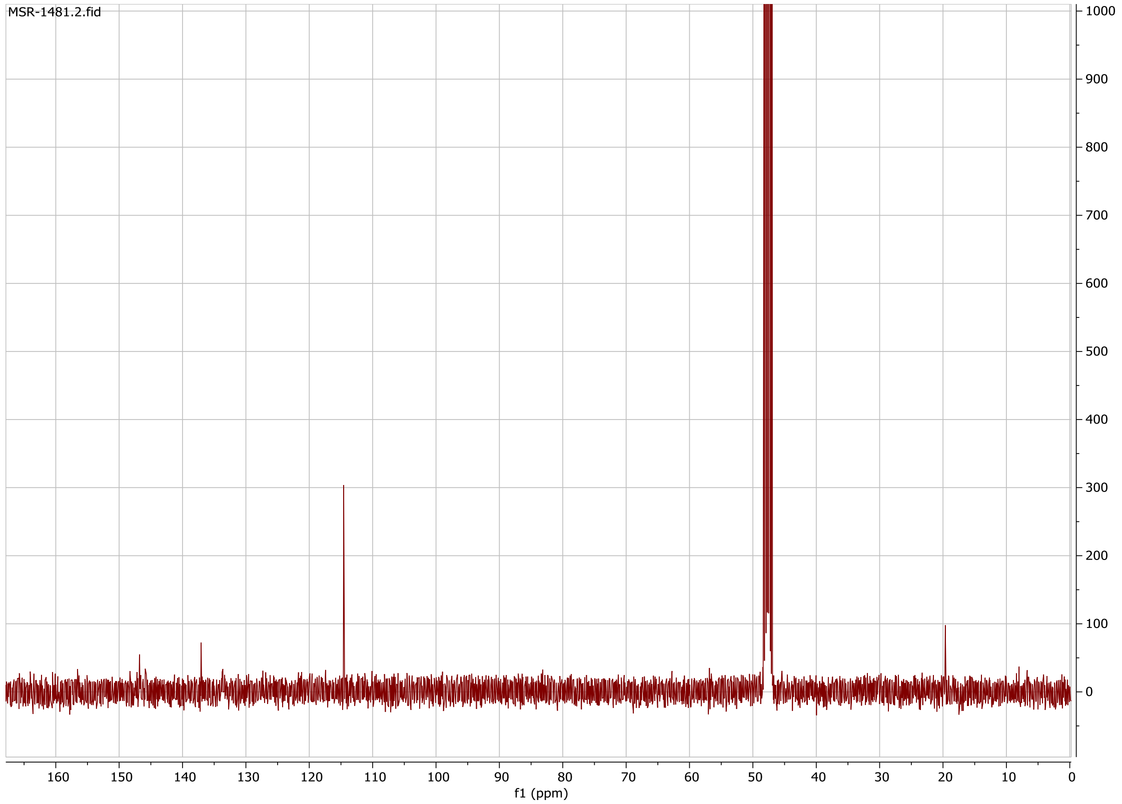

4-Chloro-3-methyl-2-(1*H*-1,2,4-triazol-3-yl)aniline (156):^{1}H NMR (400 MHz, CD_3OD)

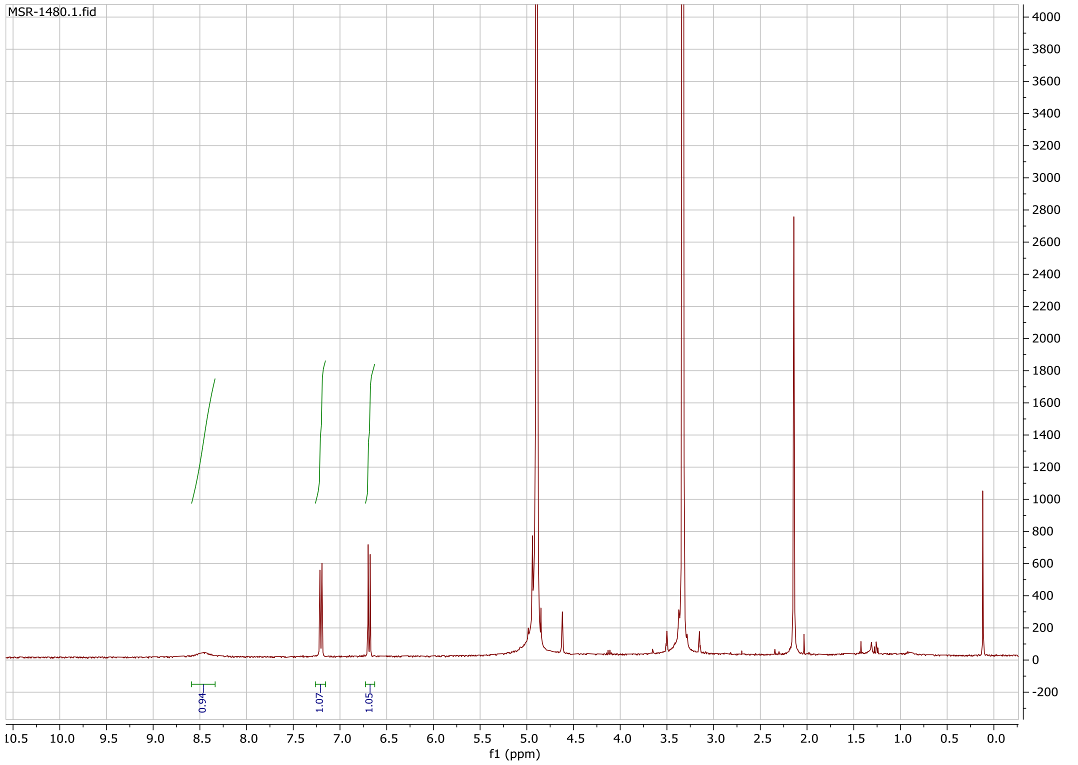

4-Chloro-3-methyl-2-(1*H*-1,2,4-triazol-3-yl)aniline (156):^{13}C NMR (101 MHz, CD_3OD)

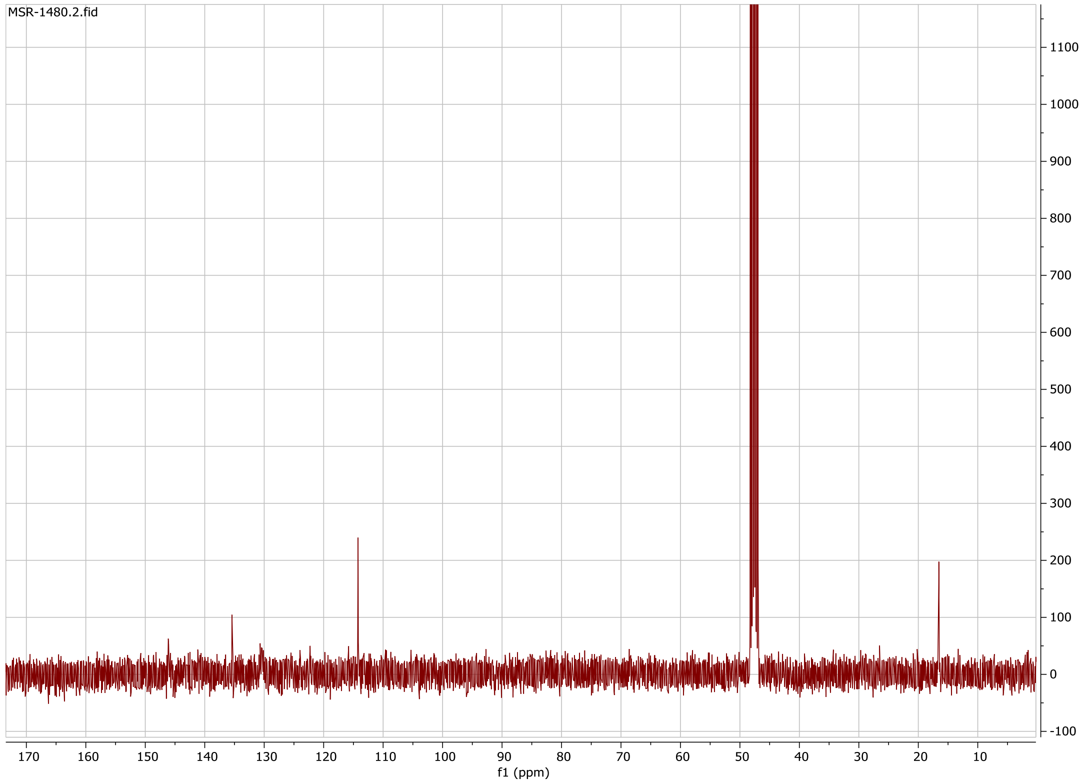

4-Chloro-2-(1*H*-1,2,4-triazol-3-yl)benzene-1,3-diamine (157):^{1}H NMR (400 MHz, CD_3OD)

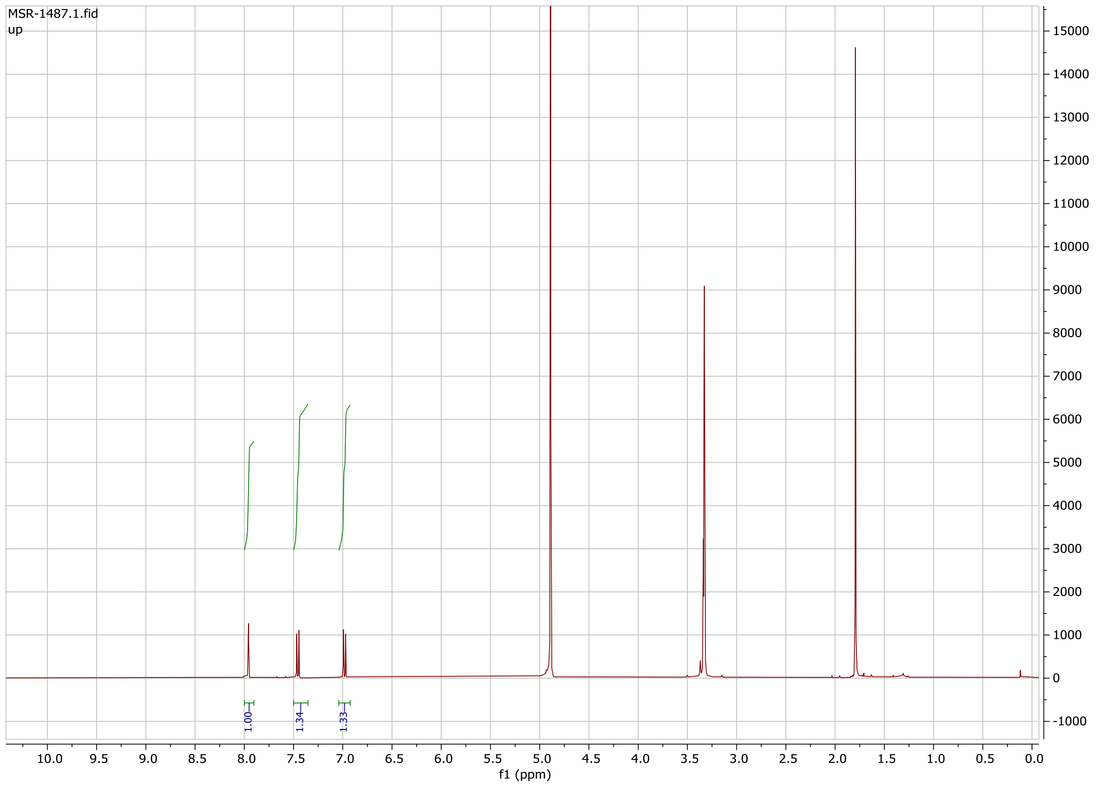

4-Chloro-2-(1*H*-1,2,4-triazol-3-yl)benzene-1,3-diamine (157):^{13}C NMR (101 MHz, CD_3OD)

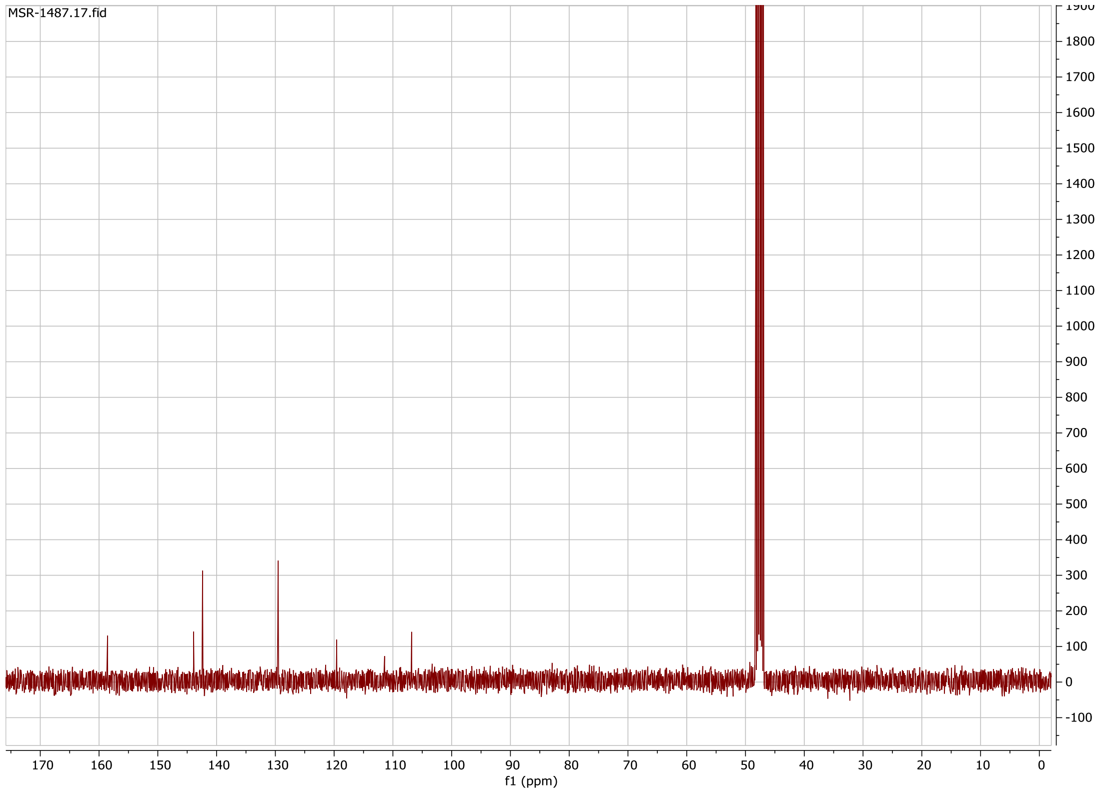

4-(1*H*-1,2,4-Triazol-3-yl)pyridine (158):^{1}H NMR (400 MHz, $CDCl_3$)

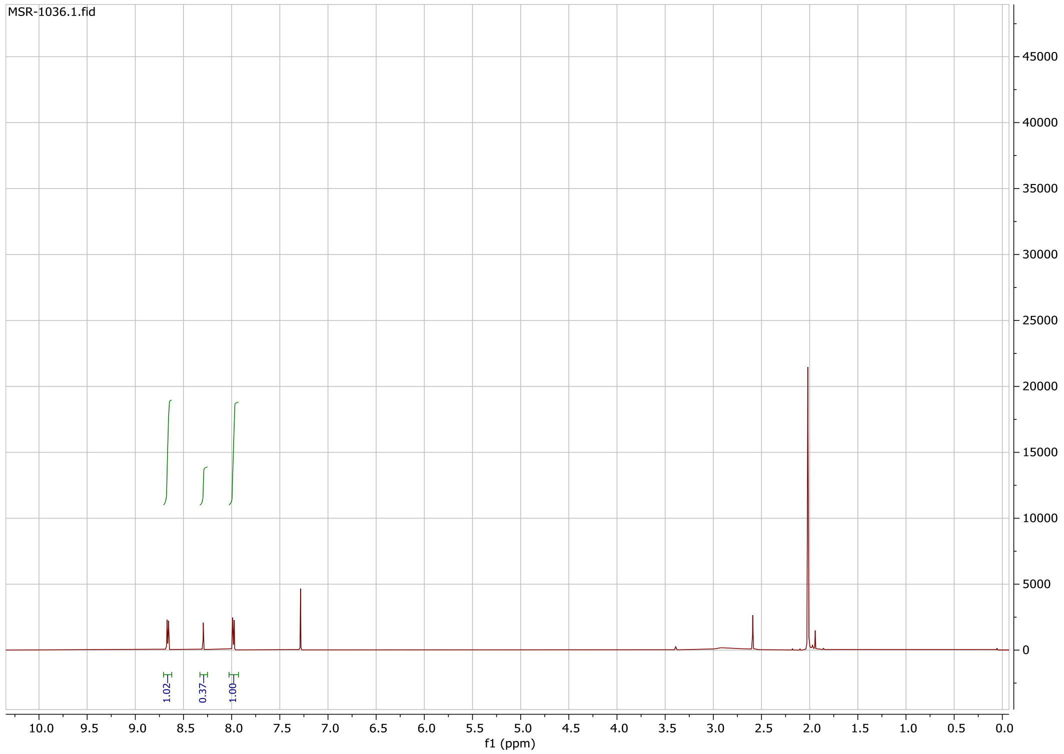

4-(1*H*-1,2,4-Triazol-3-yl)pyridine (158): ^{13}C NMR (101 MHz, CD_3OD)

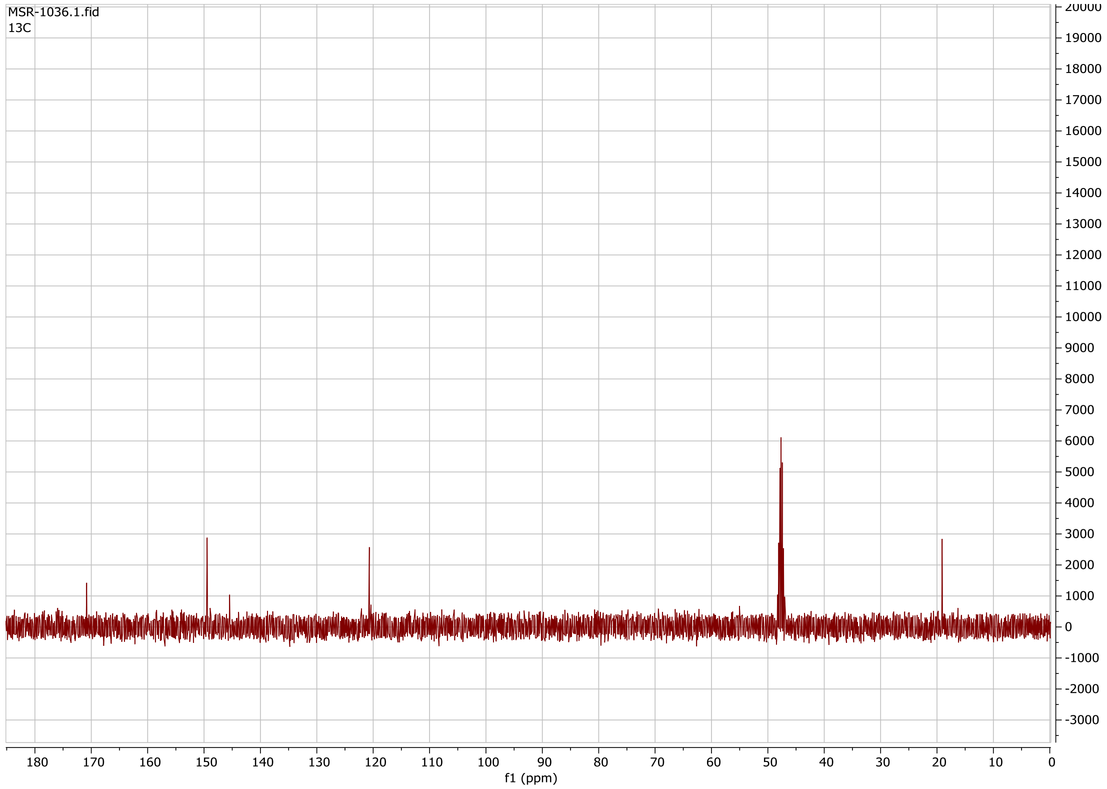

2-(1*H*-1,2,4-Triazol-3-yl)pyridine **(159):**^{1}H NMR (400 MHz, $CDCl_3$)

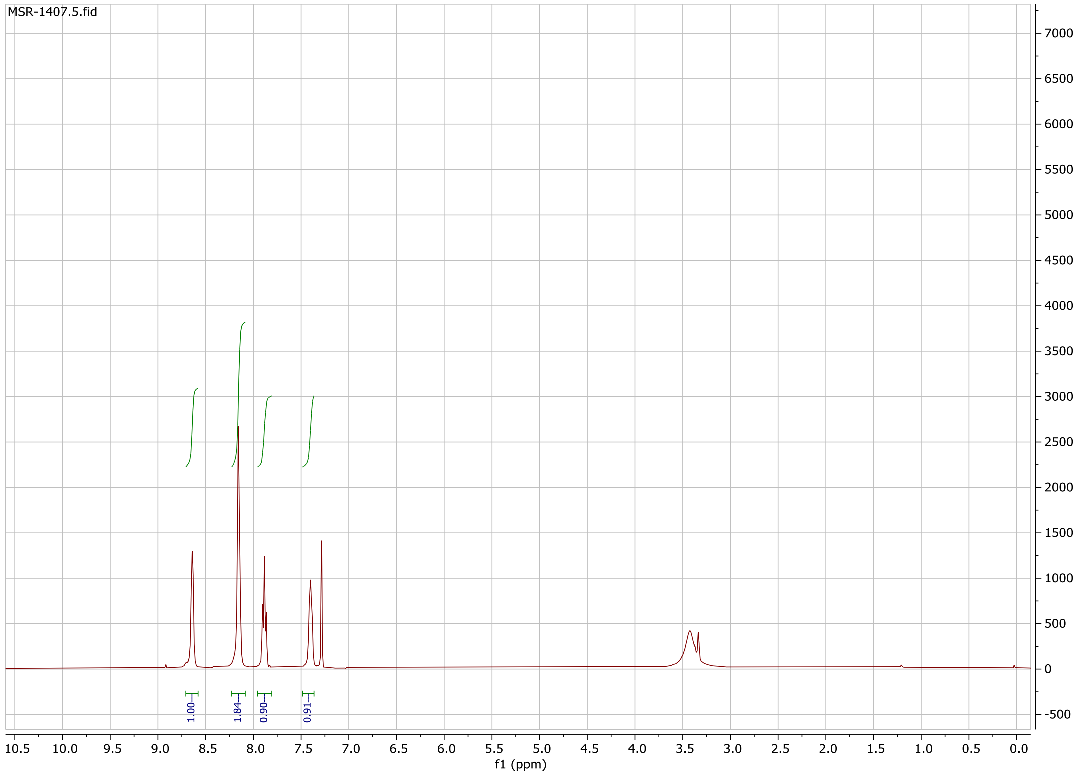

2-(1*H*-1,2,4-Triazol-3-yl)pyridine **(159):**^{13}C NMR (101 MHz, CD_3OD)

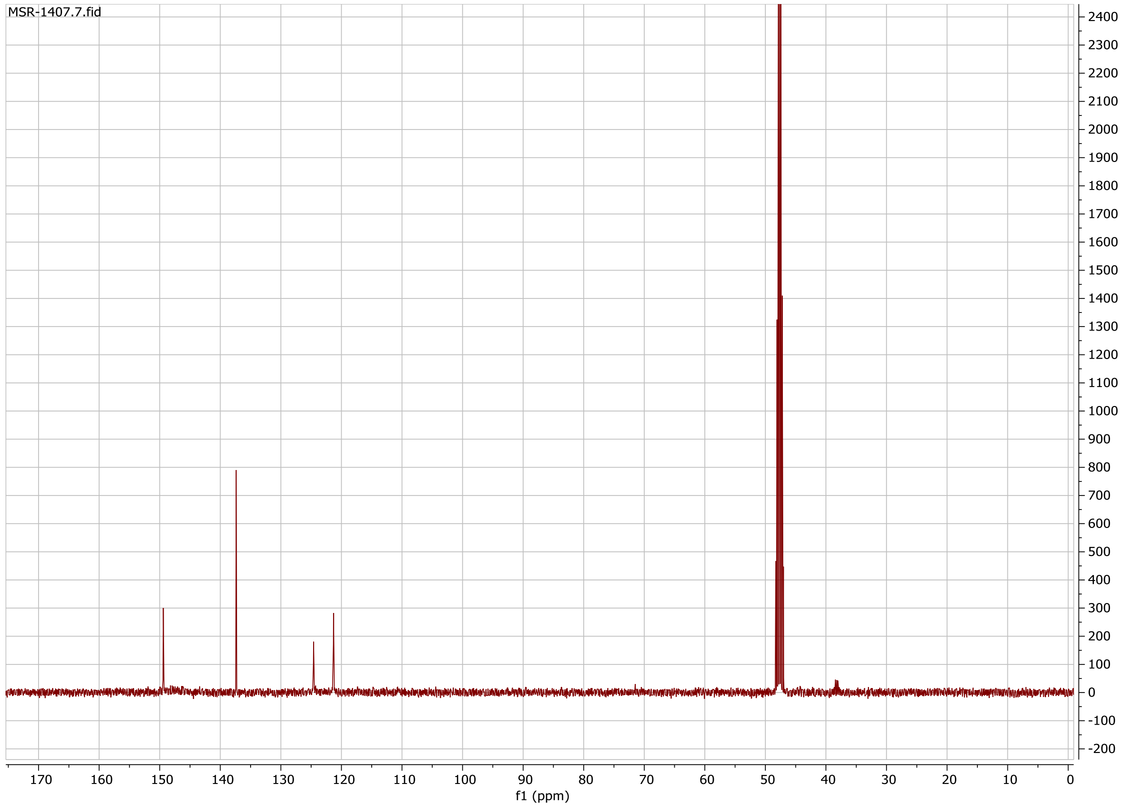

2-(1*H*-1,2,4-Triazol-3-yl)pyridine-3-amine (160):^{1}H NMR (400 MHz, CD_3OD)

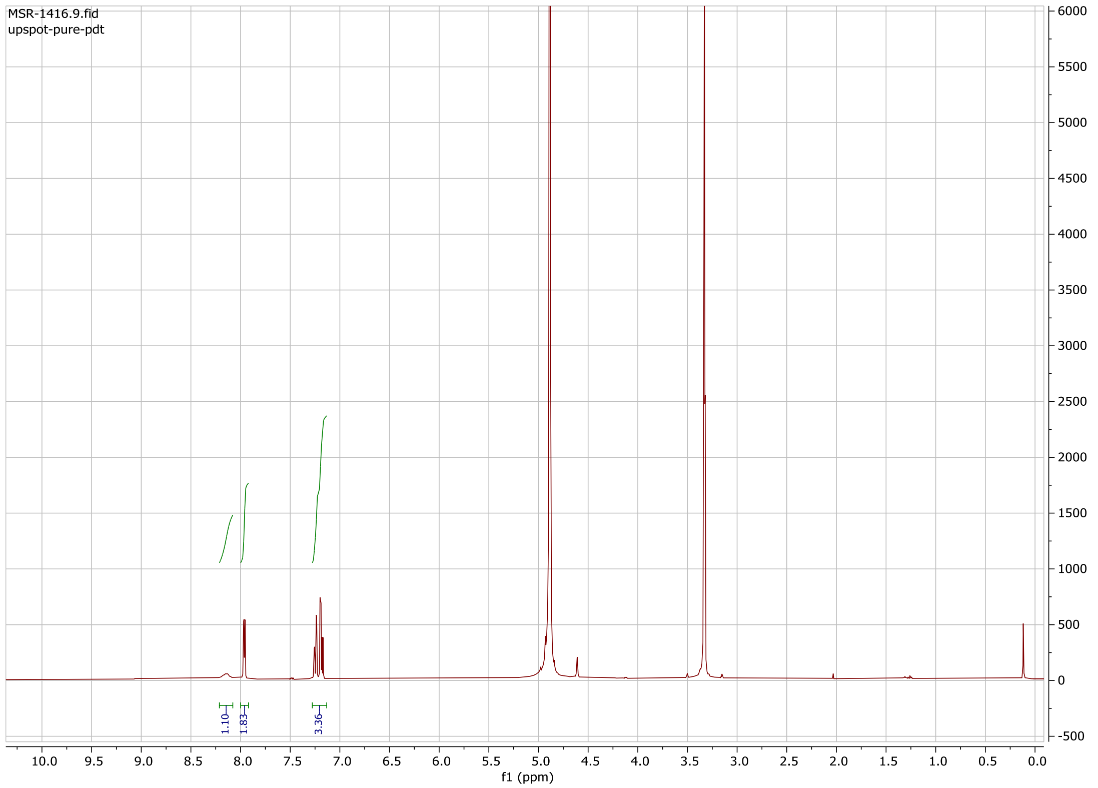

2-(1*H*-1,2,4-Triazol-3-yl)pyridine-3-amine (160):^{13}C NMR (101 MHz, CD_3OD)

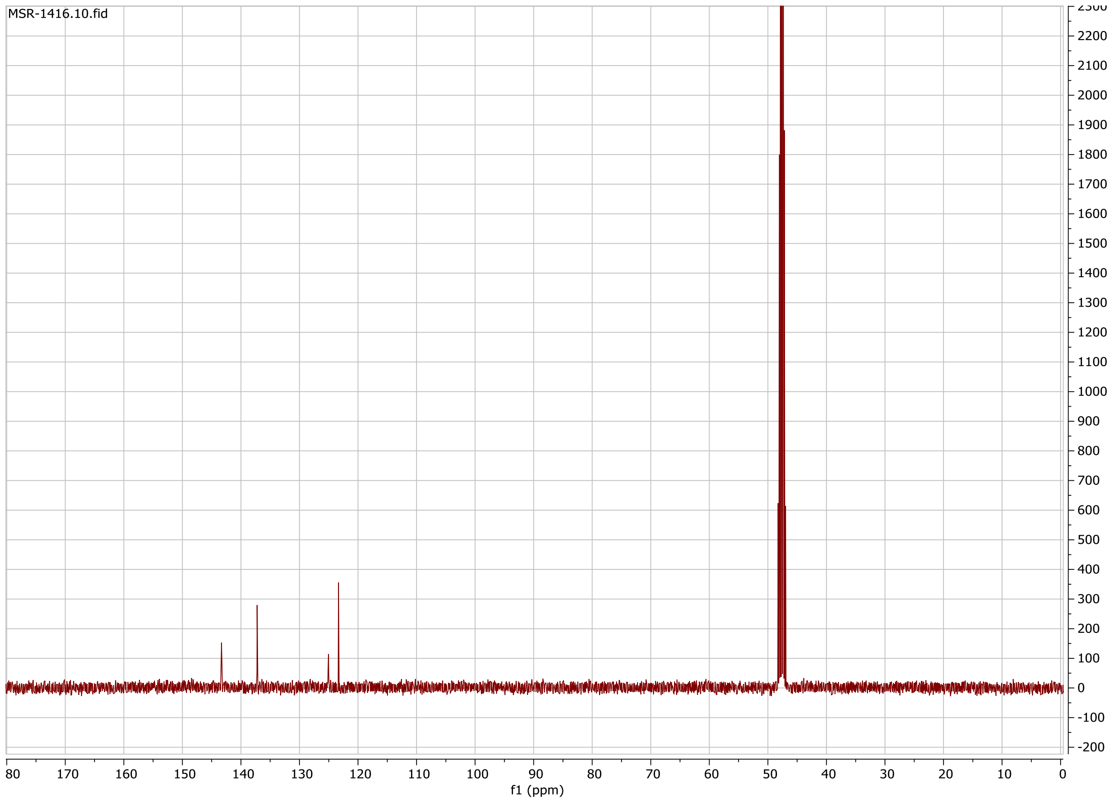

2-Chloro-6-(1*H*-1,2,4-triazol-3-yl)pyrazine (161):^{1}H NMR (400 MHz, CD_3OD)

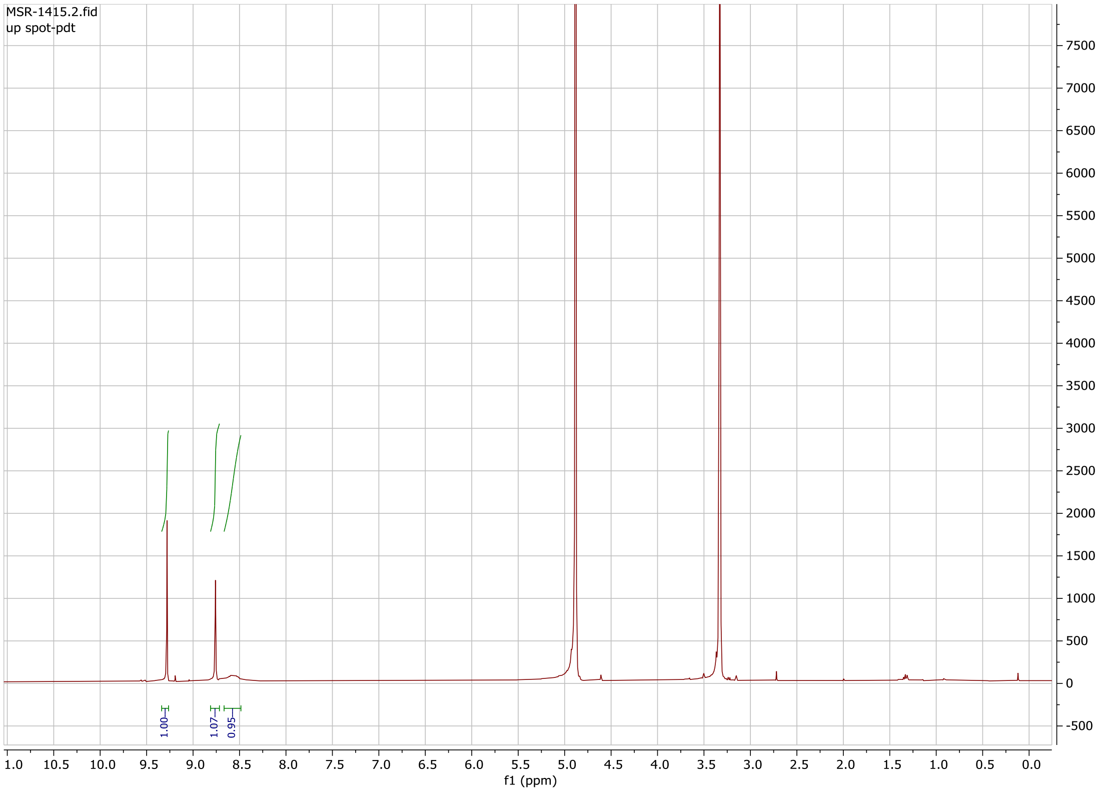

2-Chloro-6-(1*H*-1,2,4-triazol-3-yl)pyrazine (161):^{13}C NMR (101 MHz, CD_3OD)

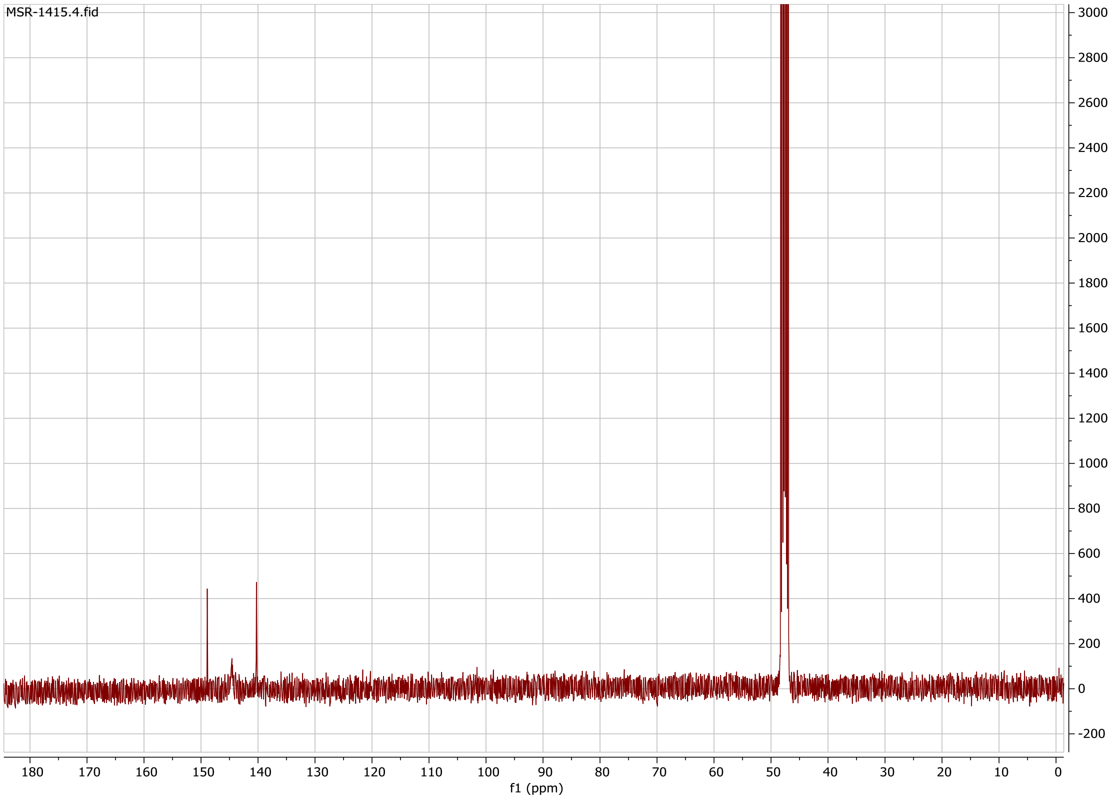

2-(1*H*-1,2,4-Triazol-3-yl)thiophen-3-amine (162):^{1}H NMR (400 MHz, CD_3OD)

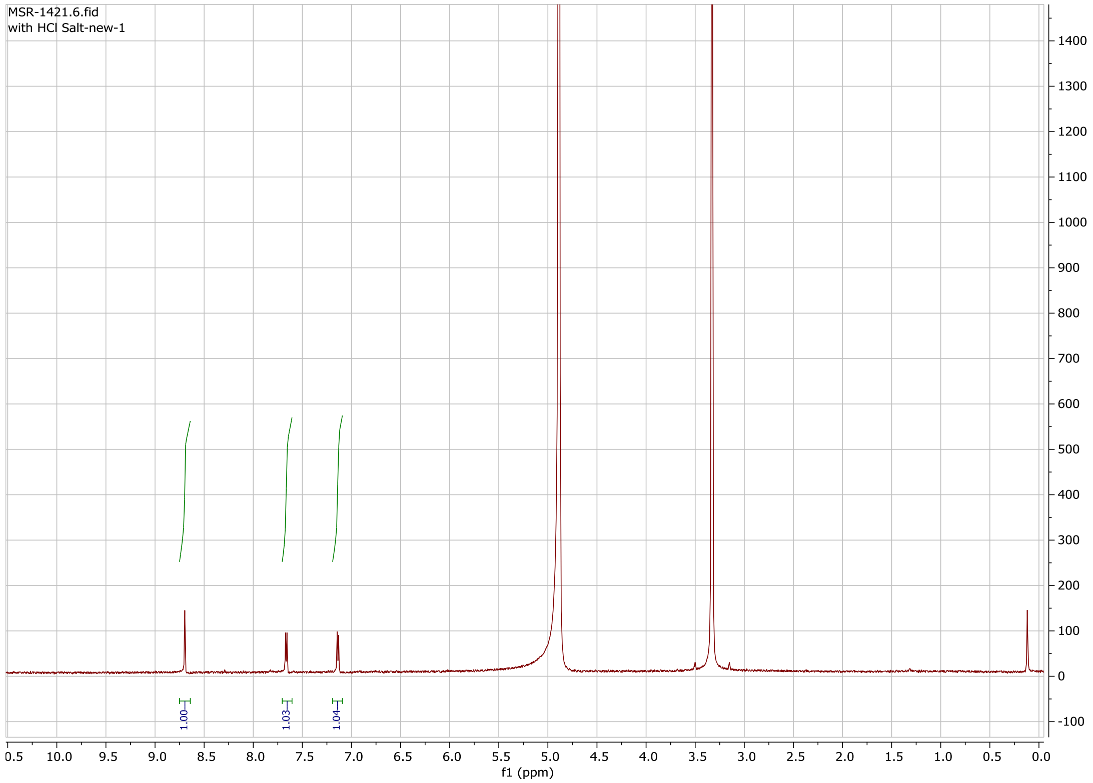

2-(1*H*-1,2,4-Triazol-3-yl)thiophen-3-amine (162):^{13}C NMR (101 MHz, CD_3OD)

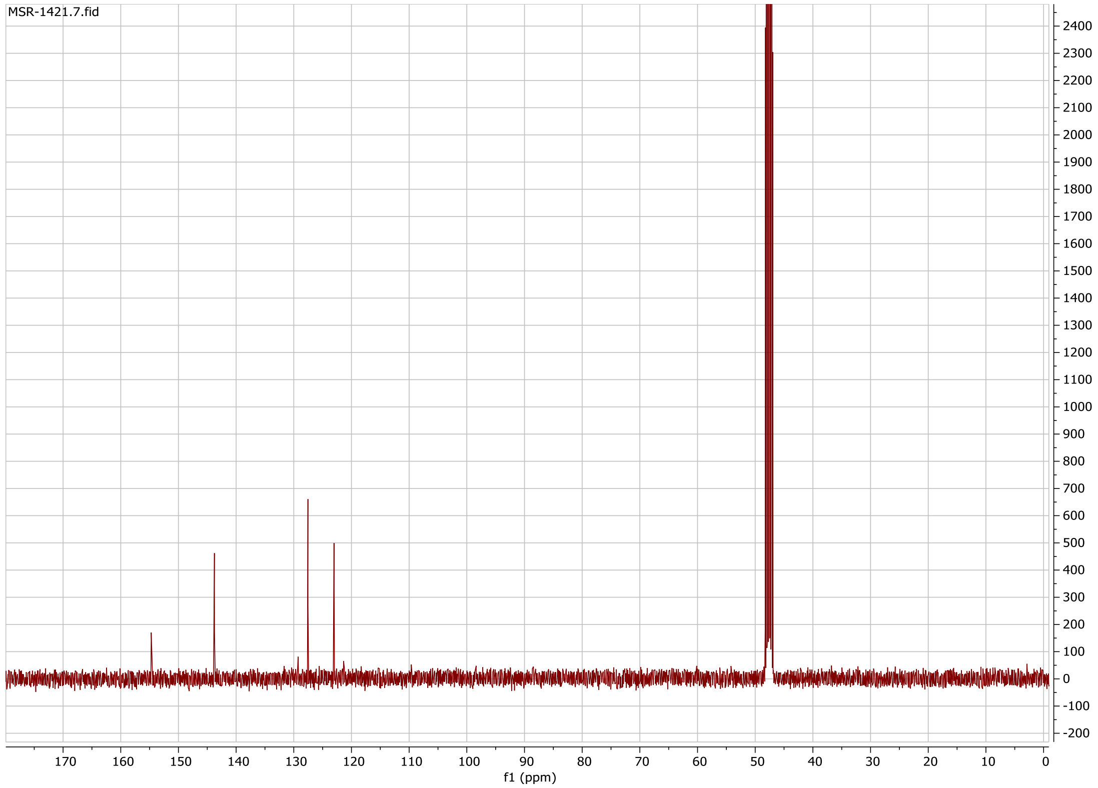

***N*-(4-Bromophenyl)-4*H*-1,2,4-triazol-3-amine (166):** ^{1}H NMR (400 MHz, $CDCl_3$)

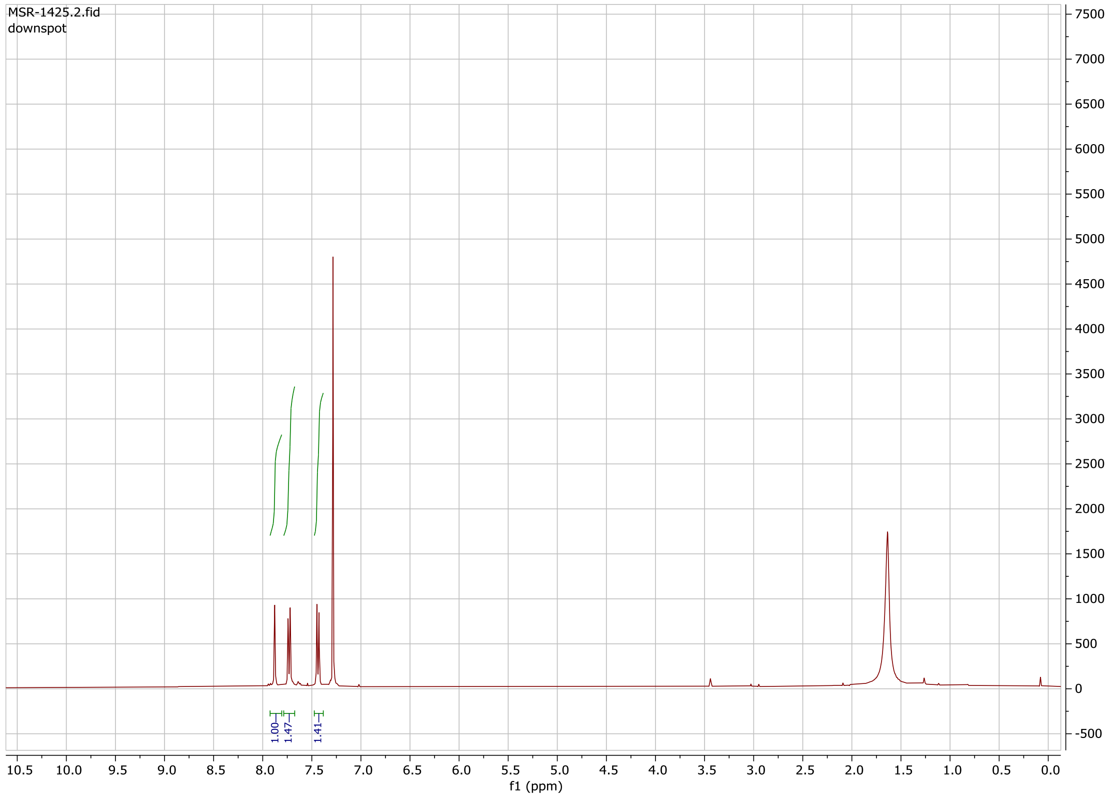

***N*-(4-Bromophenyl)-4*H*-1,2,4-triazol-3-amine (166):** ^{13}C NMR (101 MHz, CD_3OD)

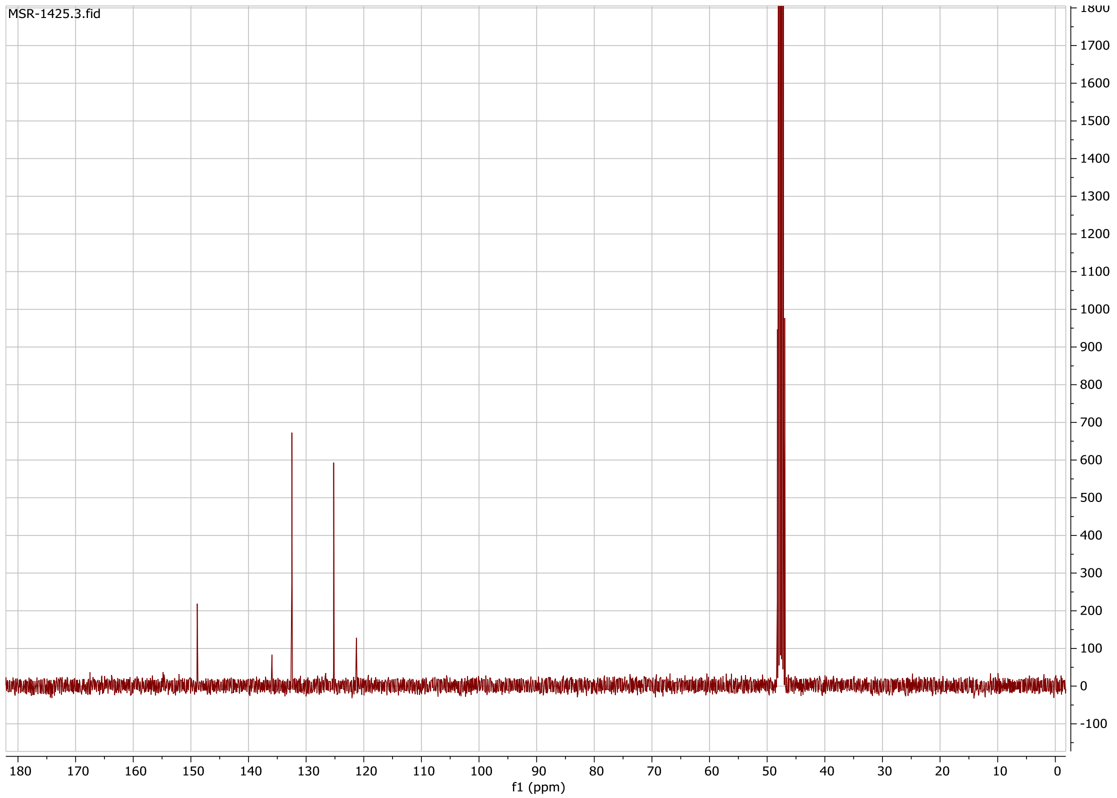

2-((1,9a-dihydrobenzo[4,5]thiazolo[2,3-*c*][1,2,4]triazol-3-yl)thio)acetamide (168): ^{1}H NMR (400 MHz, $CDCl_3$)

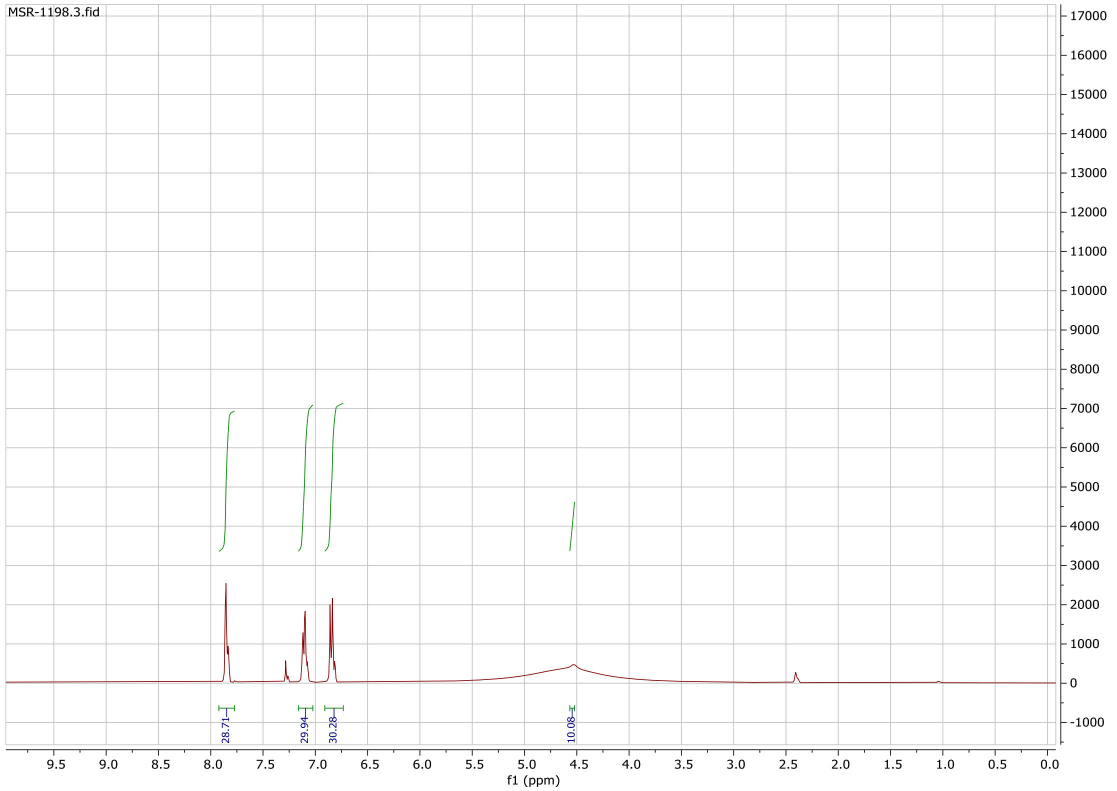

2-((1,9a-dihydrobenzo[4,5]thiazolo[2,3-*c*][1,2,4]triazol-3-yl)thio)acetamide (168): ^{13}C NMR (101 MHz, $CDCl_3$:CD_3OD)

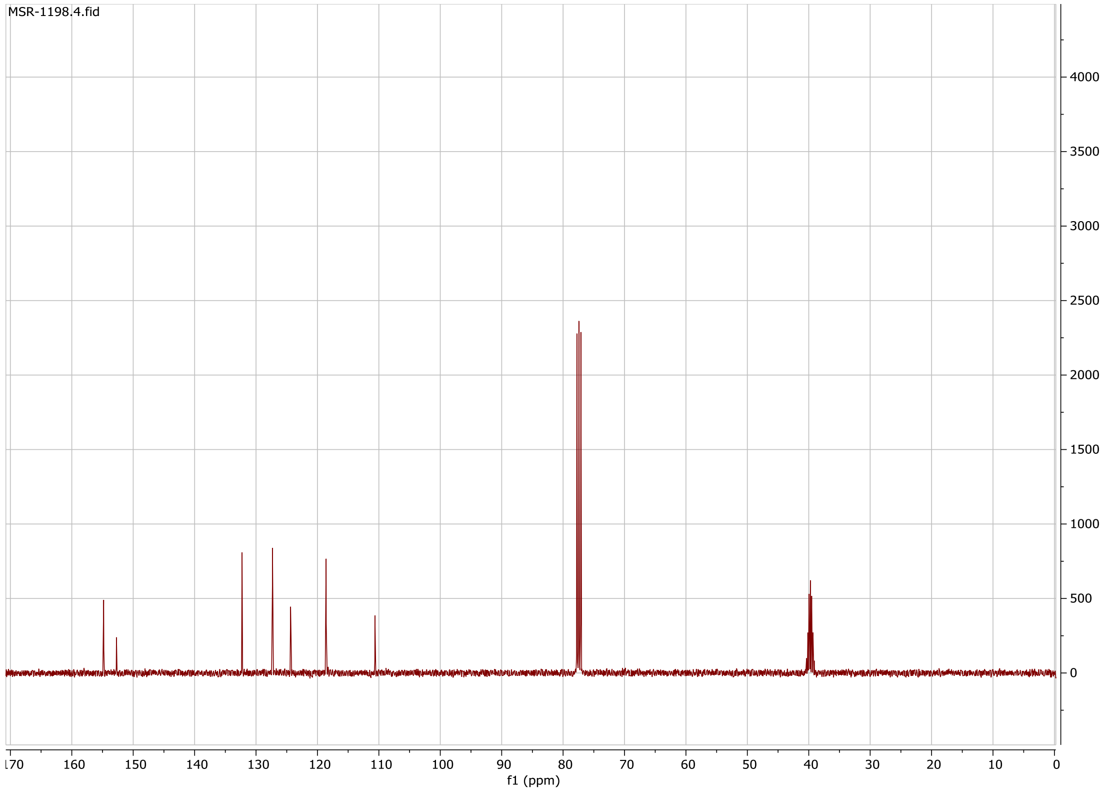

References

(1) Röhrig, U. F.; Majjigapu, S. R.; Reynaud, A.; Pojer, F.; Dilek, N.; Reichenbach, P.; Ascencao, K.; Irving, M.; Coukos, G.; Vogel, P.; Michielin, O.; Zoete, V. Azole-Based Indoleamine 2,3-Dioxygenase 1 (IDO1) Inhibitors. *J. Med. Chem.* **2021**, *64*, 2205–2227.

(2) Alexandre, J. A. C.; Swan, M. K.; Latchem, M. J.; Boyall, D.; Pollard, J. R.; Hughes, S. W.; Westcott, J. New 4-Amino-1,2,3-Triazole Inhibitors of Indoleamine 2,3-Dioxygenase Form a Long-Lived Complex with the Enzyme and Display Exquisite Cellular Potency. *ChemBioChem* **2018**, *19*, 552–561.

(3) Röhrig, U. F.; Majjigapu, S. R.; Caldelari, D.; Dilek, N.; Reichenbach, P.; Ascencao, K.; Irving, M.; Coukos, G.; Vogel, P.; Zoete, V.; Michielin, O. 1,2,3-Triazoles as inhibitors of indoleamine 2,3-dioxygenase 2 (IDO2). *Bioorg. Med. Chem. Lett.* **2016**, *26*, 4330–3.

(4) Röhrig, U. F.; Majjigapu, S. R.; Grosdidier, A.; Bron, S.; Stroobant, V.; Pilotte, L.; Colau, D.; Vogel, P.; Van den Eynde, B. J.; Zoete, V.; Michielin, O. Rational design of 4-aryl-1,2,3-triazoles for indoleamine 2,3-dioxygenase 1 inhibition. *J. Med. Chem.* **2012**, *55*, 5270–5290.
